# Remote generation of N–N axial chirality through asymmetric hydrophosphinylation/hydroamination of maleimides

**DOI:** 10.1039/d5sc06360d

**Published:** 2025-09-26

**Authors:** Yu-Li Sun, Lei Dai, Kun Zhu, Qingqin Huang, Yushuang Chen, Zugen Wu, Yixin Lu

**Affiliations:** a Department Joint School of National University of Singapore and Tianjin University, International Campus of Tianjin University Binhai New City Fuzhou 350207 China; b Department of Chemistry, National University of Singapore 117543 Singapore chmlyx@nus.edu.sg

## Abstract

Despite the significance of N–N axially chiral compounds in medicinal chemistry and asymmetric catalysis, there are limited reports on the asymmetric synthesis of N–N axially chiral compounds that also possess a phosphorus- or nitrogen-containing carbon stereogenic center. In this study, we developed organocatalytic hydrophosphinylation and hydroamination reactions of maleimides, enabling simultaneous creation of N–N axial chirality alongside a remote phosphorus or nitrogen-containing carbon chiral centre. These processes formed products in high yields with excellent enantioselectivities. The reported method represents an effective approach to construct structurally demanding chiral molecular scaffolds containing multiple chiral elements.

## Abstract 2

Organocatalytic hydrophosphinylation and hydroamination reactions of maleimides have been developed, enabling simultaneous creation of N–N axially chirality alongside a remote phosphorus or nitrogen-containing carbon central chirality.

## Graphical abstract

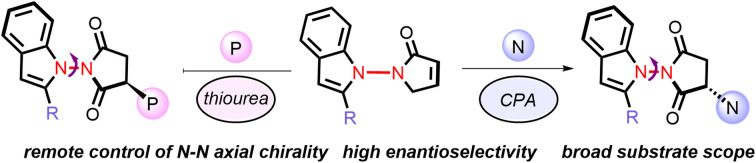

## Introduction

**Scheme 1:**
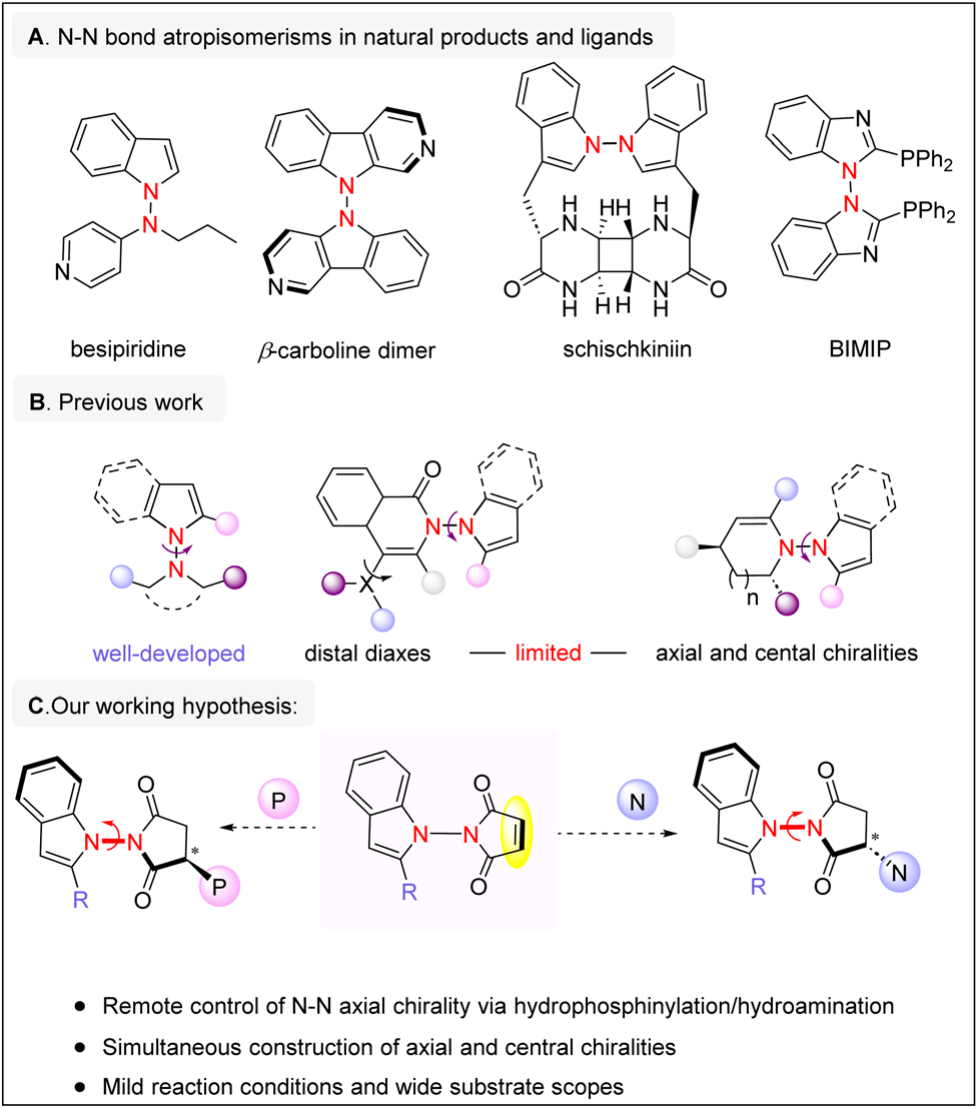
Background and our working hypothesis.

Axially chiral molecules are commonly found in biologically active compounds, natural products, and pharmaceuticals.^1^ As a result, the enantioselective synthesis of such molecules has been a long focus of synthetic chemistry over the past few decades. Although considerable achievements have been made in the atroposelective construction of C–C and C–N axial chirality,^2^ the asymmetric synthesis of molecules containing an N–N chiral axis has received relatively limited attention, despite their promising potentials. Notably, axially chiral compounds bearing an N–N axis are present in various bioactive molecules, such as besipiridine, β-carboline dimers, and schischkiniin.^3,4^ Moreover, the N–N axial chirality is also featured in chiral phosphine ligands, such as bimip,^5^ which have been successfully applied in asymmetric catalysis (Scheme 1A). Given the significance of these aforementioned molecules, there is an urgent need for the development of more efficient synthetic methods. N–N axially chiral compounds can generally be divided into three categories: those with N–N axial chirality only, those with distal diaxes and those with both axial and central chirality (Scheme 1B). Since our initial report on the atroposelective synthesis of N–N axially chiral compounds in 2021,^6^ good progress has been made in developing methods for constructing N–N axial chirality, utilizing either organocatalytic^7^ or metal-catalyzed strategies.^8,9^ Nevertheless, achieving precise stereochemical control in molecules with multiple chiral centers remains a significant challenge, as both diastereoselectivity and enantioselectivity must be addressed. As a result, there are still relatively few examples of atroposelective syntheses targeting N–N axially chiral compounds from the latter two structural classes. Recently, several fascinating reports have described the synthesis of axially chiral compounds possessing both N–N axial chirality and central chirality *via* cyclization reactions.^10,11^ Simultaneous control of multiple chiral elements—including both axial and central chirality—represents a promising approach in atroposelective synthesis.^12^ We therefore set out to design an effective process for the construction of N–N axial chirality in conjunction with the creation of central chiral centers.

In our working hypothesis, we considered employing maleimide substrates that possess an N–N axis. In our thinking, three major challenges needed to be addressed: First, effective catalyst–substrate interaction is essential for promoting desymmetrization. Second, the catalytic system must remotely control the N–N axial chirality while simultaneously establishing central chirality, thereby achieving high diastereoselectivity. Third, given the relatively low rotational barrier of N–N axial chirality, mild reaction conditions are necessary to prevent racemization during the reaction process. As part of our continuous interest in axial chirality and atroposelective synthesis,^13^ herein we document organocatalytic hydrophosphinylation and hydroamination reactions of prochiral N–N axially chiral molecules bearing a maleimide moiety, enabling the simultaneous construction of N–N axial chirality together with a remote phosphorus- or nitrogen-containing chiral center (Scheme 1C).

## Results and discussion

### Optimization of reaction conditions for hydrophosphinylation

To start our investigation, we choose 1-(2-phenyl-1*H* in-dol-1-yl)-1*H*-pyrrole-2,5-dione 1a and diphenylphosphine oxide 2a as the model substrates to evaluate their reactivity under various conditions (Table 1). In the absence of an organocatalyst, the desired racemic product 3a was obtained in 26% yield with 6:1 dr (entry 1). A range of bifunctional thiourea catalysts were then screened, which successfully suppressed the background reaction (entries 2–7). Urea catalysts with analogous structures produced similar results. Notably, catalyst C6 was the best catalyst, affording the desired product 3a in 83% yield with 73% ee and 12 : 1 dr (entry 7). Notably, the observed enhancement in diastereoselectivity indicates that the catalyst plays a crucial role in diastereoselective induction. When 5 Å molecular sieves (MS) were added, the ee value was improved to 78% (entry 8) (see the SI for results with additional additives). Increasing the catalyst loading under these conditions further improved the enantiomeric excess to 82% (entry 9). Conducting the reaction at lower concentration and reduced temperature resulted in further improvements, yielding the desired product 3a in 85% yield with 92% ee and a 12 : 1 dr (entry 12).^14^

**Table 1:** Optimization of the reaction conditions^a^

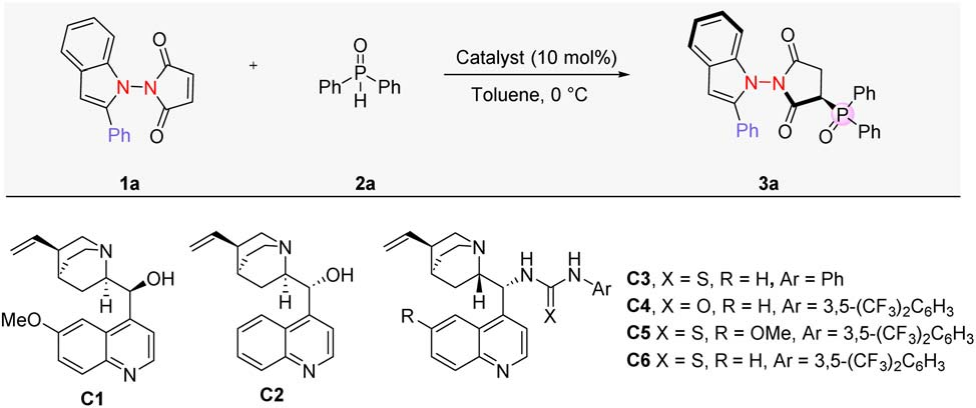					
Entry	Cat.	Additive	Yield^b^ (%)	ee (%)^c^	dr^d^
1^e^	—	—	26	0	6 : 1
2	C1	—	74	7	7 : 1
3	C2	—	87	15	10 : 1
4	C3	—	60	63	7 : 1
5	C4	—	69	70	10 : 1
6	C5	—	80	73	10 : 1
7	C6	—	83	73	12 : 1
8	C6	5 Å MS	81	78	12 : 1
9^f^	C6	5 Å MS	73	82	12 : 1
10^f^^,^^g^	C6	5 Å MS	96	83	12 : 1
11^f^^,^^g^^,^^h^	C6	5 Å MS	76	92	12 : 1
12^i^	C6	5 Å MS	85	92	12 : 1

a Reaction conditions: 1a (0.025 mmol), 2a (0.03 mmol), and catalyst (10 mol%) in toluene (0.25 mL) at 0 °C for 12 h.

b Isolated yield.

c ee determined by chiral HPLC analysis.

d dr determined by crude ^1^H NMR analysis.

e Room temperature.

f With 0.2 equiv. catalyst.

g 0.5 mL toluene.

h At −30 °C.

i Reaction conditions: 1a (0.1 mmol), 2a (0.12 mmol), 5 Å MS (100 mg) and C6 (20 mol%) in toluene (2 mL) at −30 °C for 24 h.

**Scheme 2:**
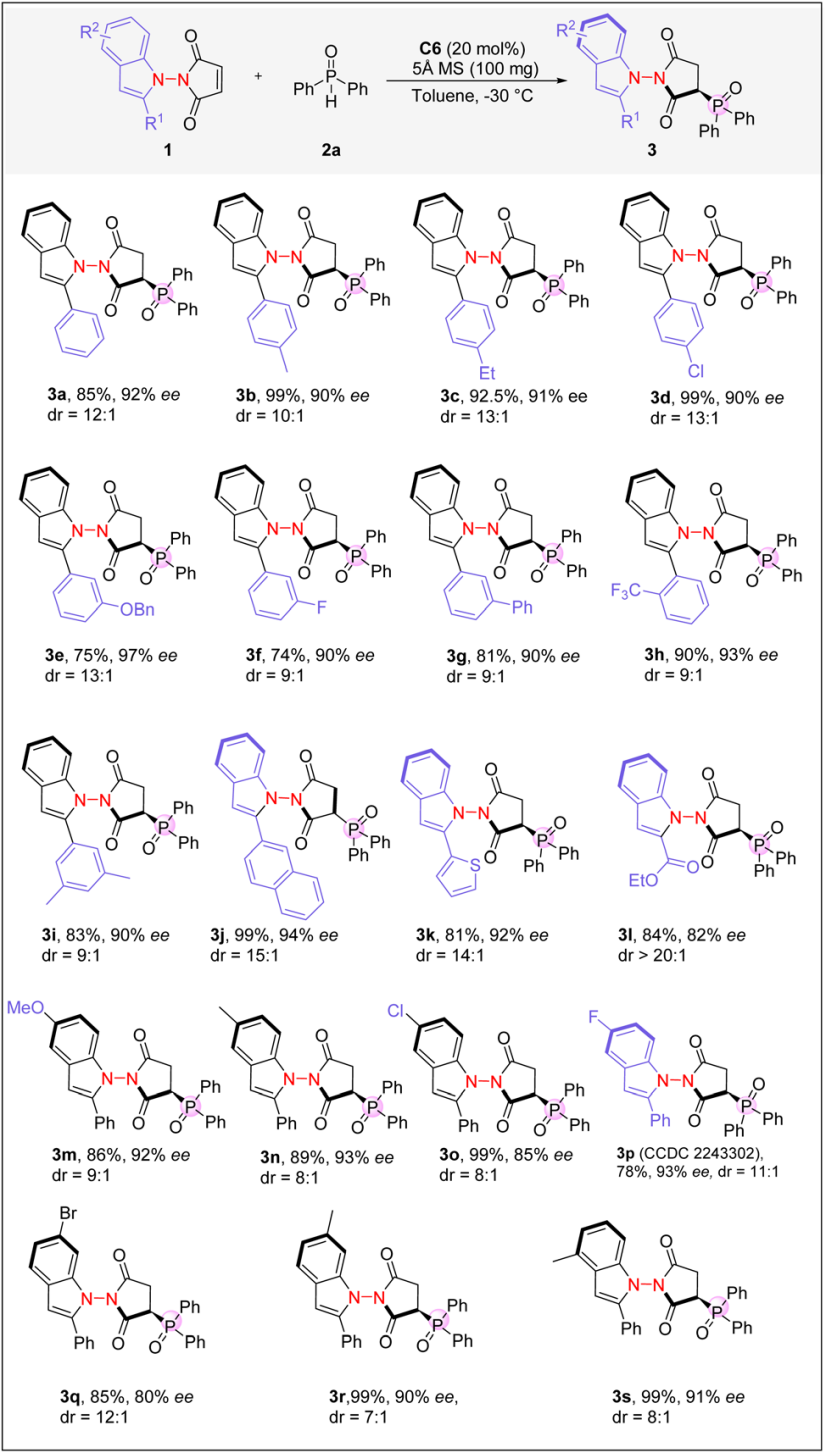
Reaction scope of indoles. Reaction conditions: 1 (0.1 mmol), 2a (0.12 mmol), C6 (20 mol%) in toluene (2 mL), and 5 Å MS (100 mg) at −30 °C for 24 h.

With the optimal reaction conditions established, we next investigated the scope of the reaction with various maleimide derivatives and phosphine oxides. As depicted in Scheme 2, imides 1 bearing various indole substructures were good substrates, yielding the corresponding axially chiral compounds 3 in high yields and with excellent enantioselectivities. The electron-withdrawing and electron-donating groups at the *ortho*-, *meta*-, and *para*-positions of the 2-phenyl-substituted indoles were well-tolerated, and the desired products with excellent enantioselectivities and diastereoselectivities were attainable (3b–3i). 1-Naphthyl and 2-thiophenyl groups could also be introduced into the C2-position of indole, resulting in excellent enantioselectivities (3j and 3k). The presence of an ester group in the indole moiety was also well-tolerated (3l). Notably, 3l was formed with a much higher dr, suggesting that the ester group may play an important role in differentiating diastereoselective pathways. Imides containing indole moieties with different substitution patterns were next examined. The 4-, 5-, or 6-substituted indoles were all found to be suitable, forming the corresponding products with good to excellent enantioselectivities and diastereoselectivities (3m–3s). The absolute configuration of the products was assigned based on the X-ray crystallographic analysis of 3p (see the SI).

**Scheme 3:**
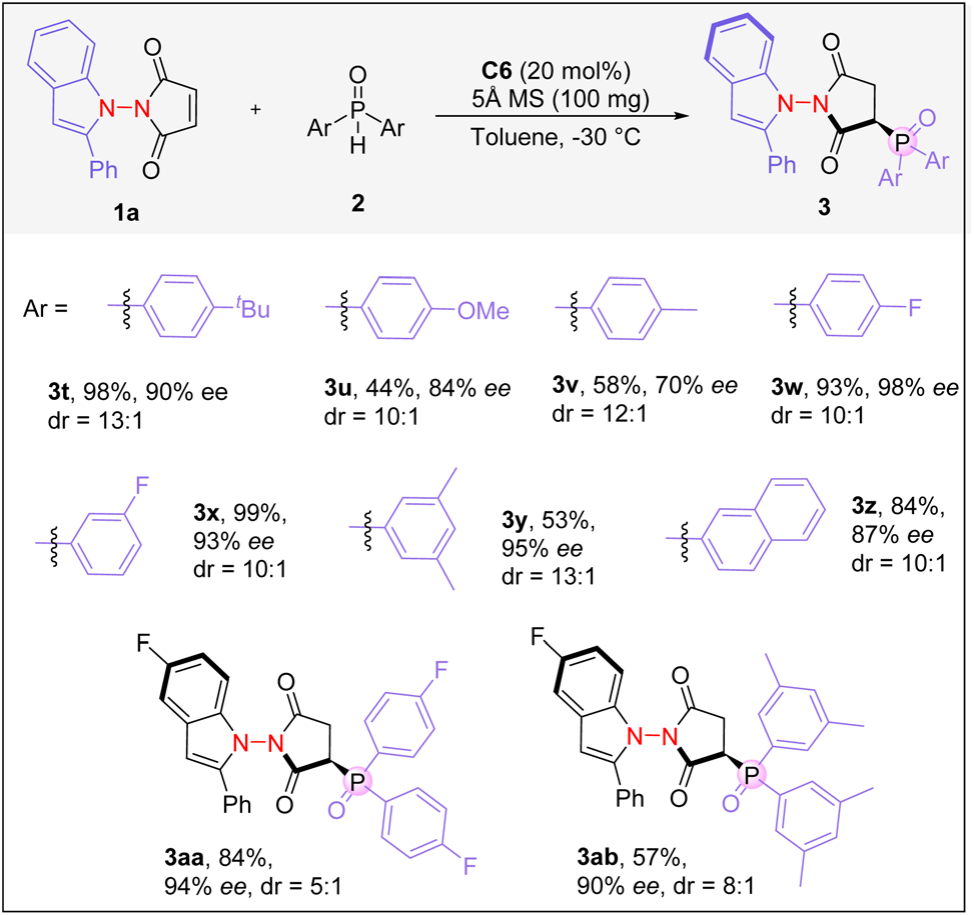
Reaction scope of diaryl phosphine oxides. Reaction conditions: 1a (0.1 mmol), 2 (0.12 mmol), C6 (20 mol%) in toluene (2 mL), and 5 Å MS (100 mg) at −30 °C for 24 h.

Next, we investigated the scope of diaryl phosphine oxides (Scheme 3). When a bulky *tert*-butyl group was present on the aryl moiety of phosphine oxide, the desired product was obtained in 98% yield, with 90% ee and a diastereomeric ratio of 13 : 1 (3t). Phosphine oxides containing electron-donating group substituted phenyl rings worked less efficiently (3u and 3v). Phosphine oxides bearing a *para*- or *meta*-fluoro-substituted phenyl group performed very well, forming the corresponding products in excellent yields with excellent enantioselectivities and very high dr ratios (3w and 3x). Furthermore, a number of other phosphine oxides with different aryl groups could all be utilized, and good results were obtained (3y–3ab), demonstrating generality of the hydrophosphinylation strategy in creating N–N axially chiral compounds.

Subsequently, we investigated the use of *N*-nucleophiles to simultaneously construct a nitrogen-containing carbon stereogenic center as well as the N–N axially chiral axis. We chose benzyl amines as the nucleophile and investigated the targeted transformation in the presence of various organic catalysts (Table 2). In the absence of a catalyst, treating imides 1a and 1v with benzylamine afforded the corresponding amination products in low yields. Substrate 1a gave products with poor diastereoselectivity, whereas 1v delivered a dr of 10 : 1 (entries 1 and 2). Furthermore, by utilizing imide 1a in the best catalytic system established earlier for the hydrophosphinylation reaction, the amination product 5a′ was obtained in a good yield, but in a racemic form with very low diastereoselectivity (entry 3). Using imide 1v markedly improved the dr, although the enantioselectivity remained very poor (entry 4), suggesting that the substrate structure strongly influences the diastereoselectivity of the hydroamination reaction. Attempts to improve enantioselectivity of the reaction by employing various bifunctional tertiary amine catalysts were unsuccessful (see the SI). We then turned to utilizing chiral phosphoric acids (CPAs), as they are tremendously powerful in asymmetric catalysis.^15^ After some screening (entries 5–8), CP5 was identified as the optimal catalyst, affording the desired amination product 5a in high yield with good enantioselectivity and decent diastereoselectivity (entry 9).

**Table 2:** Optimization of the reaction conditions^a^

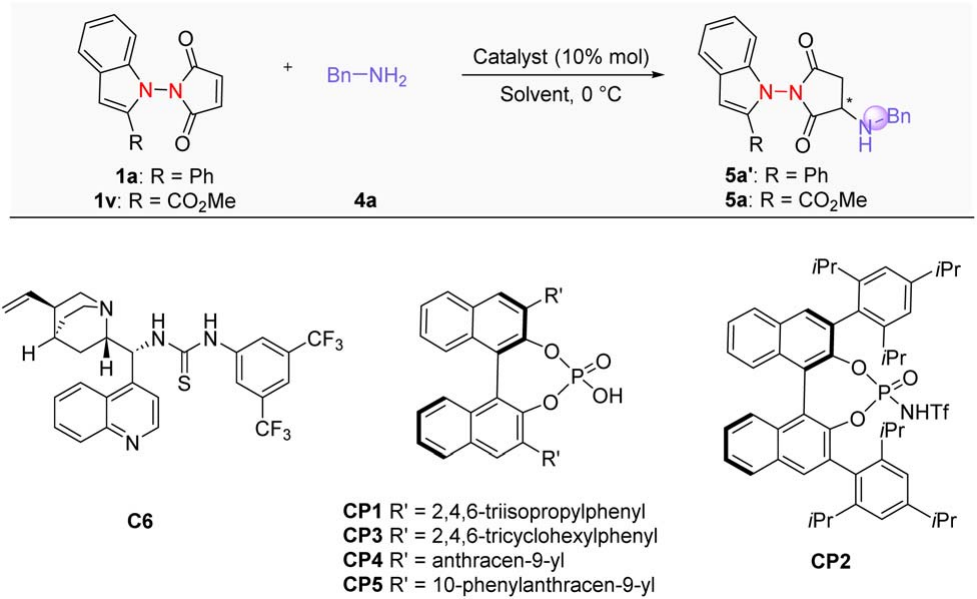					
Entry	1	Cat.	Yield (%)^b^	ee (%)^c^	dr^d^
1	1a	—	29	0	1.5 : 1
2	1v	—	32	0	10 : 1
3	1a	C6	83	0	1.2 : 1
4	1v	C6	80	2	10 : 1
5	1v	CP1	79	44	11.5 : 1
6	1v	CP2	72	7	9 : 1
7	1v	CP3	82	63	7 : 1
8	1v	CP4	74	47	4.5 : 1
9	1v	CP5	77	83	4 : 1

a Reaction conditions: 1v (0.025 mmol), 4a (0.025 mmol), catalyst (10 mol%) in toluene (0.25 mL), and 5 Å MS(25 mg) at 0 °C for 3 h.

b Isolated yield.

c ee determined by chiral HPLC analysis.

d dr determined by crude ^1^H NMR analysis.

**Scheme 4:**
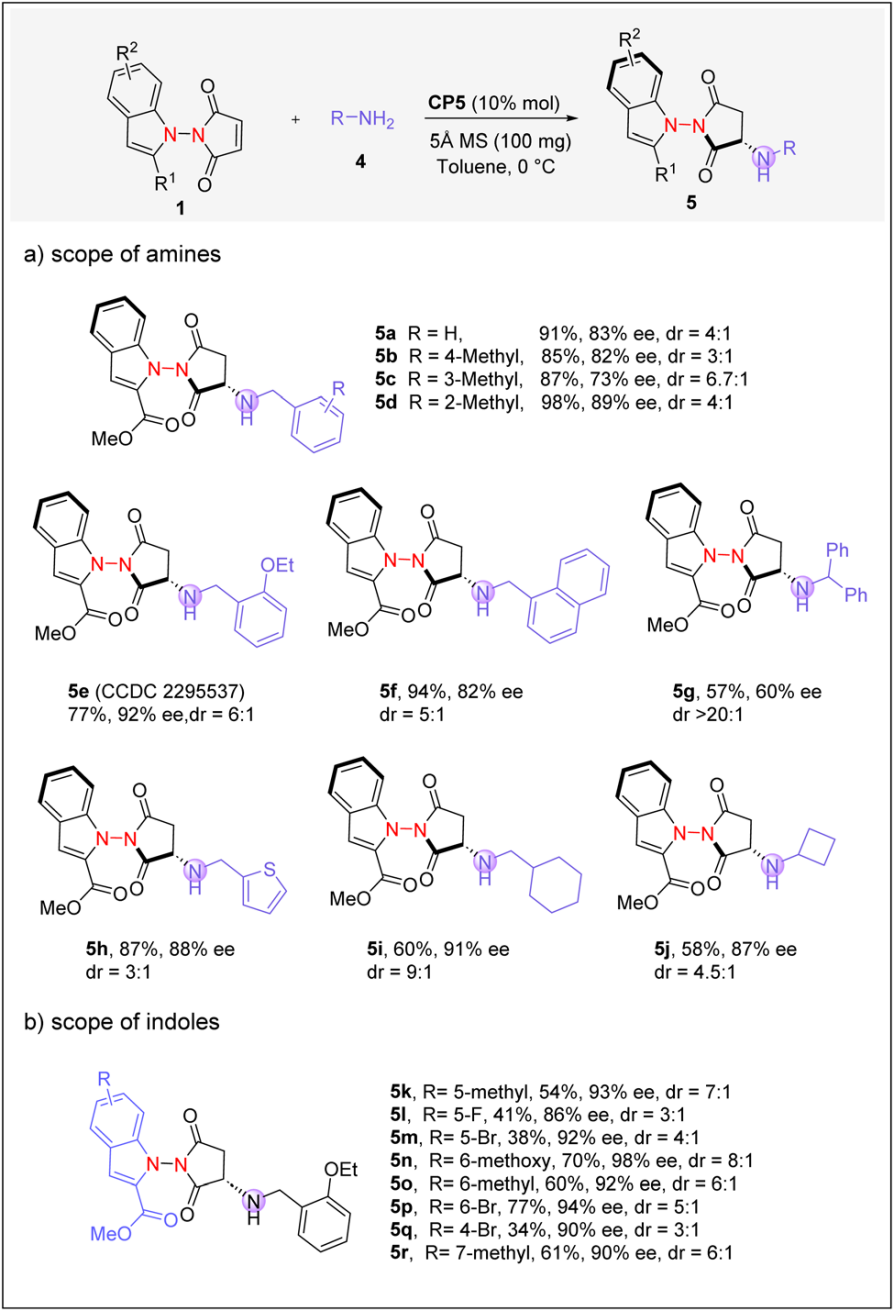
Reaction scope of hydroamination. [a] Reaction conditions: 1 (0.1 mmol), 4 (0.11 mmol), CP5 (10 mol%) in toluene (2 mL), and 5 Å molecular sieves (100 mg) at 0 °C for 3 h.

With the optimal conditions in hand, we proceeded to explore the substrate scope of this hydroamination reaction (Scheme 4). Monosubstituted benzylamines bearing *ortho*-, *meta*-, or *para*-methyl groups yielded the corresponding products in excellent yields with good stereoselectivities (5b–5d). Benzylamine bearing an electron-donating ethoxyl substituent was also suitable, and the absolute configuration of the corresponding hydroamination product was confirmed by X-ray crystallography (5e). Furthermore, benzylamines containing a naphthyl or a thiophenyl group were well-tolerated, and high enantioselectivity was attainable (5f and 5h). However, benzyl amine bearing a diphenyl substituent is a less ideal substrate (5g). Interestingly, the phenyl group in benzyl amines could be replaced by alkyl substituents, and high enantiomeric selectivities were well-maintained (5i and 5j). We also investigated the applicability of substrates bearing various indole substructures to this amination reaction, and the desired products were generally obtained with high ee values (5k–5r).

**Scheme 5:**
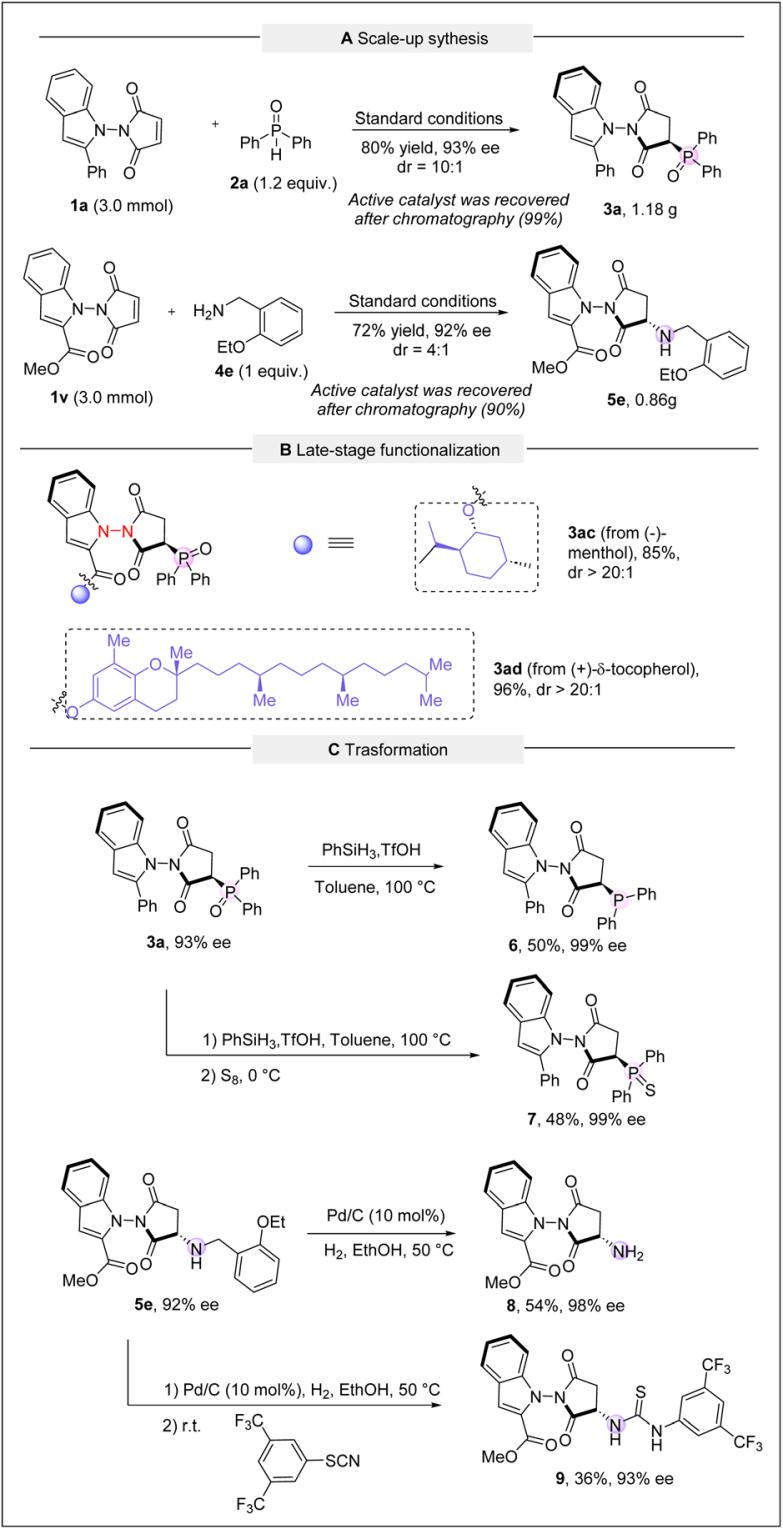
Synthetic applications.

We next evaluated the practicality and synthetic applications of the methods developed (Scheme 5). First, the scale-up experiments were carried out, and the desired products 3a and 5e were obtained in good yields with excellent enantioselectivities (Scheme 5A). It is noteworthy that over 90% of the catalyst could be recovered, and the recycled catalyst remained effective, making the process economical. When the catalyst loading for hydrophosphinylation was reduced to 10 mol%, the yield was slightly reduced to 77% while ee and dr values were well-maintained. Additionally, we investigated the late-stage functionalization of natural products such as (–)-menthol and (+)-δ-tocopherol using our reaction conditions. Both substrates afforded the desired products 3ac and 3ad in good yields with excellent regio- and diastereoselectivities, highlighting the versatility of this method for applications in medicinal chemistry and drug discovery (Scheme 5B). We also synthetically elaborated the products. For example, treatment of compound 3a with phenylsilane and triflic acid yielded the reduced product 6 in moderate yield with 99% ee. Phosphine oxide could be converted to phosphine sulfide 7 with 99% ee. In addition, the benzyl moiety on 5e could be readily cleaved to yield primary amine 8, and the corresponding thiourea 9 was also derived. Notably, both transformations proceeded smoothly without erosion of enantiomeric purity (Scheme 5C).

**Scheme 6:**
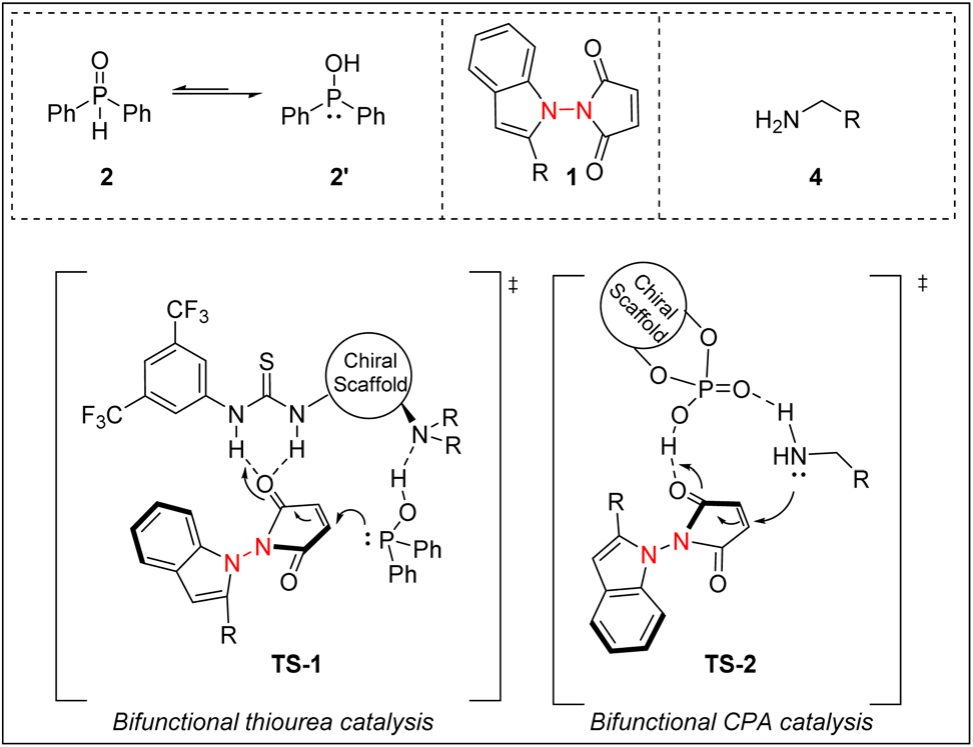
Proposed mechanism.

Based on our experimental results and related reports in the literature,^16^ a plausible reaction mechanism is proposed (Scheme 6). In the hydrophosphinylation reaction, bifunctional thiourea catalyst C6 simultaneously interacts with phosphine 2′ and α,β-unsaturated ketone substrate 1 through a weak H-bonding interaction to form TS-1, producing the desired phosphinylation product. The hydroamination cycle follows a similar pattern. Both amine 4 and α,β-unsaturated ketone 1 are activated by the bifunctional chiral phosphoric acid CP5*via* H-bonding interactions to form the intermediate *via*TS-2, leading to the formation of the desired amination product. Notably in both cases, the creation of the remote central phosphorus or nitrogen-containing chiral centers also simultaneously generated the N–N axial chirality.

## Conclusions

In summary, we have developed asymmetric organocatalytic hydrophosphinylation and hydroamination reactions of maleimides, enabling the efficient synthesis of N–N axially chiral compounds with excellent yields and stereoselectivities under mild reaction conditions. Notably, the products feature N–N axial chirality alongside a remote phosphorus- or nitrogen-containing carbon chiral center. Moreover, these N–N axially chiral compounds have potential applications as valuable ligands or organocatalysts in asymmetric catalysis. We believe this method offers broad utility for the synthesis of new N–N axially chiral molecular architectures bearing multiple chiral elements. Investigations of their applications in asymmetric catalysis are currently ongoing in our laboratory.

## Author contributions

Y. S. designed and carried out the experiments. K. Z., L. D., Q. H., Y. C., and Z. W. participated in the synthesis of substrates. Y. S. and Y. L. conceived the project and wrote the manuscript. Y. L. supervised the project.

## Conflicts of interest

The authors declare no conflicts of interest.

## Supplementary Material

SC-OLF-D5SC06360D-s001

SC-OLF-D5SC06360D-s002

## Data Availability

All experimental procedures, characterization, and copies of NMR spectra for all new compounds can be found in the SI. Supplementary information is available. See DOI: https://doi.org/10.1039/d5sc06360d. CCDC 2243302 and 2295537 contain the supplementary crystallographic data for this paper.^17*a*,*b*^
