# Supplementary material for: Remote generation of N–N axial chirality through asymmetric hydrophosphinylation/hydroamination of maleimides
Source: Chem Sci. 2025 Sep 26;16(42):20042–7. doi: 10.1039/d5sc06360d (PMC12486558; doi:10.1039/d5sc06360d)
Supplement: SC-016-D5SC06360D-s001 [file SC-016-D5SC06360D-s001.pdf]

Supplementary Information (SI) for Chemical Science.
This journal is © The Royal Society of Chemistry 2025

Electronic Supplementary Information For

Remote Generation of N–N Axial Chirality through Asymmetric Hydrophosphinylation/Hydroamination of Maleimides

Yu-Li Sun,[†,§] Lei Dai,[§] Kun Zhu,[§] Qingqin Huang,[†,§] Yushuang Chen,[§] Zugen Wu,[§] Yixin Lu[*,†,§]

†Joint School of National University of Singapore and Tianjin University, International Campus of Tianjin University, Binhai New City, Fuzhou, Fujian, 350207, China.

§Department of Chemistry, National University of Singapore, 3 Science Drive 3, 117543, Singapore.

*Corresponding author: chmlyx@nus.edu.sg

Table of Contents

General Information S3

Reaction Optimization S4

Representative procedures S8

Characterization Data of Products S9

References S74

X-Ray Data S74

NMR Spectra S78

General information

Unless otherwise specified, all reagents and solvents were purchased from commercial suppliers (Sigma Aldrich and BLD) and used without further purification. Flash column chromatography was performed using silica gel (200-300 mesh). NMR spectra were recorded on Bruker DPX 400 spectrometer at 400 MHz for ^{1}H NMR, 100 MHz for ^{13}C NMR, 376 MHz for ^{19}F NMR, or on a Bruker AMX500 spectrometer at 500 MHz for ^{1}H NMR, 125 MHz for ^{13}C NMR, 470 MHz for ^{19}F NMR. NMR chemical shifts were determined relative to CDCl3 (δ H = 7.26 ppm, δ C = 77.16 ppm). 1H NMR Spectroscopy splitting patterns were designated as singlet (s), doublet (d), triplet (t), quartet (q). Splitting patterns that could not be interpreted or easily visualized were designated as multiplet (m). The racemic sample was prepared by Et_3N catalysis. Catalysts were synthesized by following previously reported Procedures.[1] Optical rotations were measured using an Anton Paar MCP-100 polarimeter. Enantiomeric excesses were determined by HPLC analysis on a chiral stationary phase using a Chiralpak column (AD-H, IC and IE) with hexanes/iPrOH as the eluent. HRMS spectra of all compounds were recorded on a Bruker micrOTOFQ II (ESI). Optical rotations were measured using an Anton Paar MCP-100 polarimeter.

Reaction Optimization

Optimizing of reaction condition:

Table S1. The Screen of Catalyst in the reaction.[a]

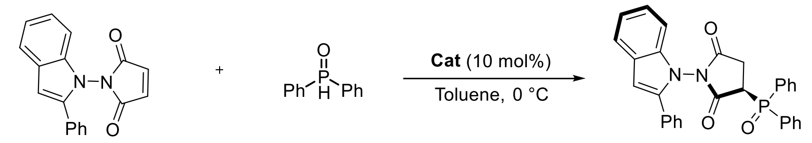

1a **2a** **3a**

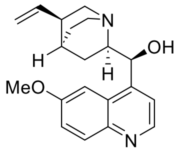

C1
74%, 7% *ee*

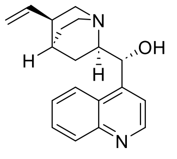

C2
87%, 15% *ee*

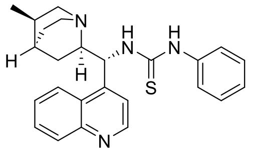

C3
60%, 63% *ee*

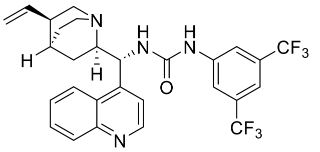

C4
69%, 70% *ee*

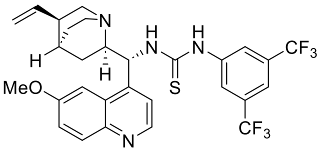

C5
80%, 73% *ee*

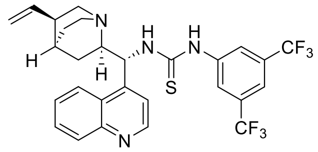

C6
83%, 73% *ee*

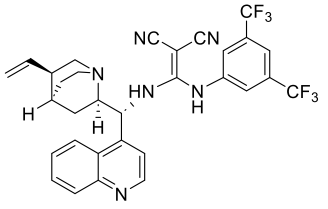

C7
81%, 50% *ee*

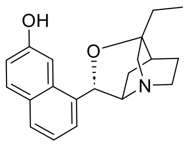

C8
60%, 4% *ee*

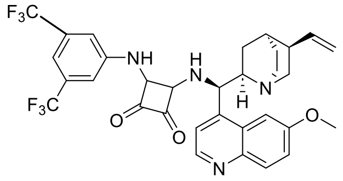

C9
89%, -37% *ee*

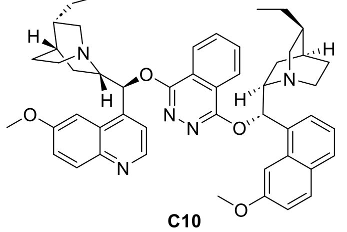

C10
55%, -35% *ee*

[a] Reaction conditions: **1a** (0.025 mmol), **2a** (1.2 eq.), and Catalyst (10 mol%), Toluene (2.0 ml), N_2, 12 h.

Table S2. The Screen of Additives in the reaction.[a]

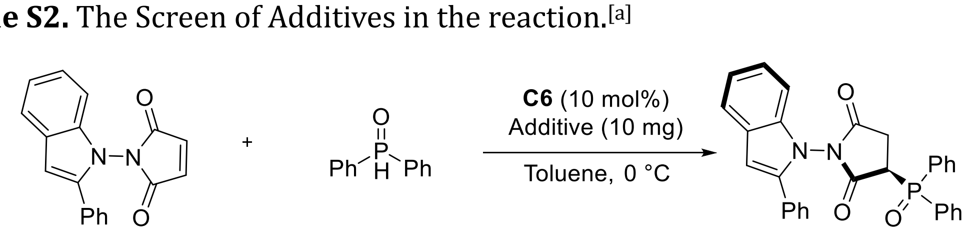

1a **2a** **3a**

Entry	Catalyst	additive	Yield (%)[b]	ee (%)[c]	dr[d]

1		-	26	0	-
2	**C6**	-	83	73	10:1
3	**C6**	4Å	60	77	10:1
4	**C6**	5Å	81	78	12:1
5	**C6**	3Å	82	73	12:1
6	**C6**	13X	73	75	7:1
7	**C6**	$MgSO_4$	91	64	10:1

[a] Reaction conditions: **1a** (0.025 mmol), **2a** (1.2 eq.), and Catalyst (10 mol%), Toluene (0.25 mL), N_2, 12 h. [b] Isolate yield. [c] Determined by HPLC analysis. [d] Determined by crude ^{1}H-NMR analysis.

Table S3. The Screen of the amount of additive and catalyst in the reaction.[a]

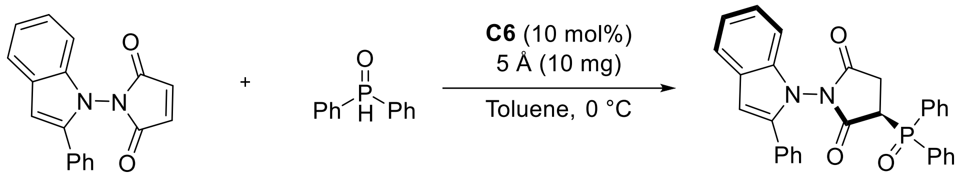

1a **2a** **3a**

Entry	additive	Yield (%)[b]	ee (%)[c]	d.r.[d]
1	-	83	73	12:1
2	5Å	81	78	12:1
3[e]	5Å	73	82	12:1
4[f]	5Å	89	80	12:1
5[e, g]	5Å	73	83	12:1
6[e, g, h]	5Å	96	83	12:1

[a] Reaction condition:**1a** (0.025 mmol), **2a** (1.2 eq.), and Catalyst (10 mol%), Toluene (0.25 mL), N_2, 12 h. [b] Isolate yield. [c] Determined by chiral HPLC analysis. [d] Determined by crude ^{1}H-NMR analysis. [e] 0.2 equiv catalyst. [f] 0.3 equiv catalyst. [g] With the addition of 5Å MS (25 mg). [h] 0.5 mL toluene.

Table S4. The Screen of the amount of Temp and Solvent in the reaction.[a]

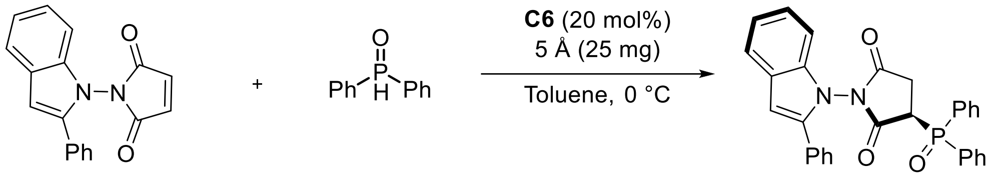

1a **2a** **3a**

Entry	Slovent	Temp (°C)	Yield (%)[b]	ee (%)[c]	d.r. [d]
1	toluene	0	96	83	12:1

2	toluene	-10	96	89	12:1
3	toluene	-20	97	91	12:1
4	toluene	-30	76	92	12:1
5	toluene	-40	68	91	12:1
6	$PhCF_3$	-30	60	90	10:1
7	CH_3CN	-30	42	53	10:1
8	$CHCl_3$	-30	69	45	10:1
9[e]	toluene	-30	85	92	12:1

Reaction condition:**1a** (0.025 mmol), **2a** (1.2 eq.), and Catalyst (20 mol%), Toluene (0.25 mL), 5Å MS (25 mg), N_2, 12 h. [b] Isolate yield. [c] Determined by chiral HPLC analysis. [d] Determined by crude ^{1}H-NMR analysis. [e] Reaction condition:**1a** (0.1 mmol), **2a** (1.2 eq.), and Catalyst (20 mol%), Toluene (2 mL), 5Å MS (100 mg), -30 °C, N_2, 24 h.

Table S5. The Catalysts evaluated in the reaction.[a]

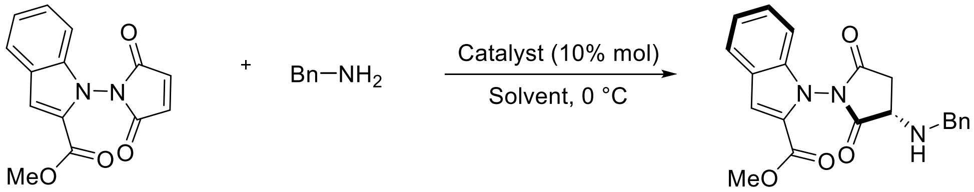

1v **4a** **5a**

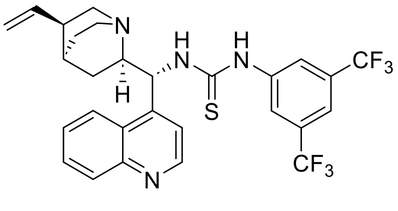

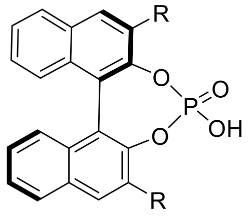

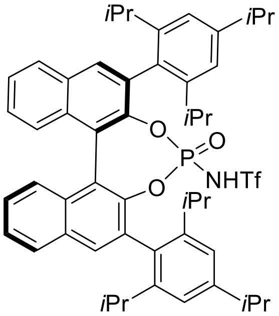

C6
80%
2 % ee, 12:1 dr

CP2 72%
7 % ee, 9:1 dr

CP1 R = 2,4,6-triisopropylphenyl 79%, 44 % ee, 11.5:1 dr
CP3 R = 2,4,6-tricyclohexylphenyl 82%, 63 % ee, 7:1 dr
CP4 R = anthracen-9-yl 74%, 47% ee, 4.5:1 dr
CP5 R = 10-phenylanthracen-9-yl 77%, 83% ee, 4:1 dr

[a] Reaction condition:**1a** (0.025 mmol), **2a** (1.0 eq.), and Catalyst (10 mol%), Toluene (0.5 mL), 5Å MS (25 mg), N_2, 3 h.

Table S6. The Screen of the amount of Temp and Solvent in the reaction.[a]

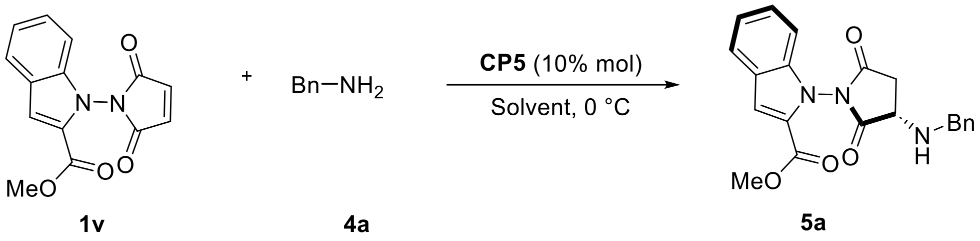

Entry	Temp (°C)	Slovent	Additive	Yield[b] (%)	ee[c]	d.r.[d]
1	0	toluene	5 Å	77	83	4:1
2	0	Et_2O	5 Å	75	71	4:1
3	0	DCE	5 Å	76	36	3:1
4	0	Mesitylene	5 Å	75	81.5	5.5:1
5	0	CCl_4	5 Å	76	80	6.7:1
6	10	toluene	5 Å	78	81	4:1
7	-10	toluene	5 Å	76	83	4:1
8	0	toluene	4 Å	72	82	4:1
9	0	toluene	3 Å	73	83	4:1

[a] Reaction condition:**1a** (0.025 mmol), **2a** (1.0 eq.), and Catalyst (10 mol%), Toluene (0.5 mL), 5Å MS (25 mg), N_2, 3 h. [b] Isolate yield. [c] Determined by chiral HPLC analysis. [d] Determined by ^{1}H-NMR analysis.

Figure S1. Examples of hydrophosphinylation, limitations.

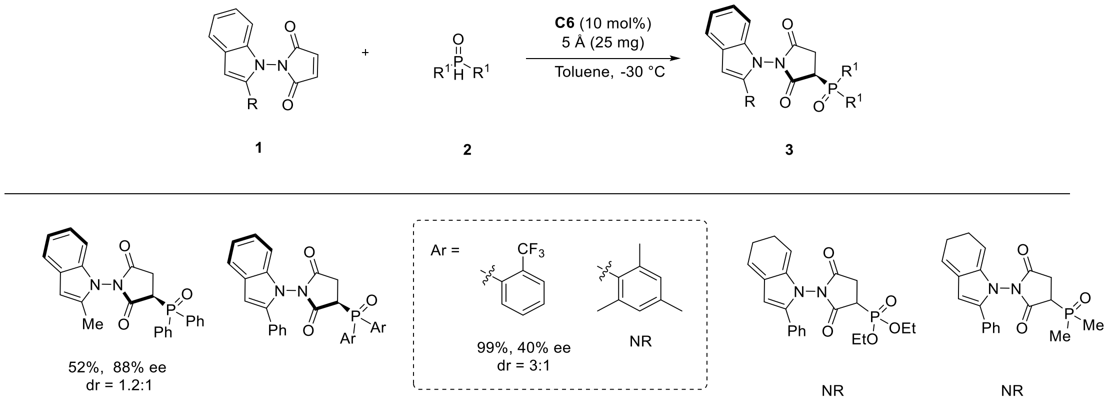

[a] Reaction condition:**1** (0.025 mmol), **2** (1.2 eq.), and Catalyst (10 mol%), Toluene (0.5 mL), 5Å MS (25 mg), N_2, 12 h. [b] Isolate yield. [c] Deter mined by chiral HPLC analysis. [d] Determined by ^{1}H-NMR analysis.

Figure S2. Examples of hydroamination, limitations.

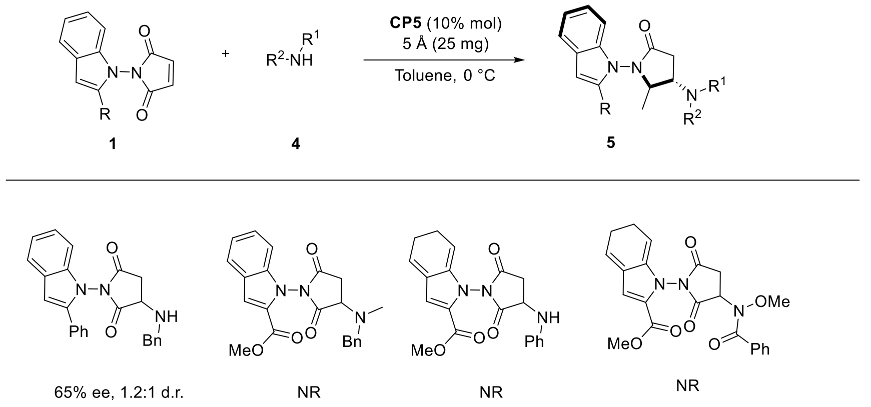

[a] Reaction condition:**1** (0.025 mmol), **4** (1.0 eq.), and Catalyst (10 mol%), Toluene (0.5 mL), 5Å MS (25 mg), N_2, 3 h. [b] Isolate yield. [c] Determined by chiral HPLC analysis. [d] Determined by ^{1}H-NMR analysis.

Representative procedures

Preparation of substrate 1[2-3]:

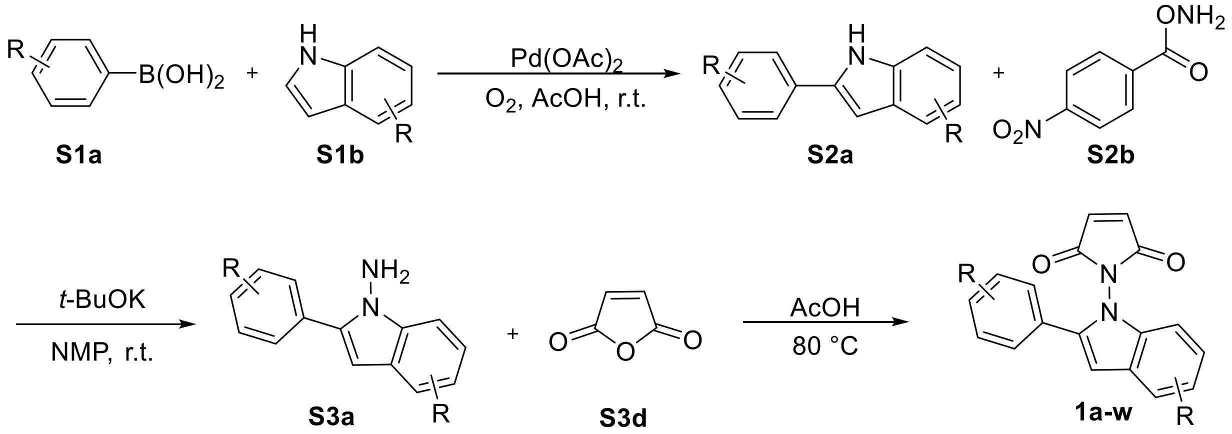

Representative Procedure A:

Procedure 1: Indole **S1a** (1.0 eq.), boronic acid (1.5 eq.), $Pd(OAc)_2$ (5 mol%) ware added in a Schlenk. The resulting mixture was degassed twice and filled of O_2, and acetic acid (3M) added. The reaction mixture was stirred at r.t. for 24 h. After the completion of the reaction which was indicated by TLC, the solvent was removed under reduced pressure. Then the crude mixture was diluted with EtOAc and washed with saturated $NaHCO_3$ (2x30 mL). the organic layer was dried with

anhydrous Na_2SO_4 and then concentrated under reduced pressure. The residue was purified through flash column chromatography on silica gel (hexane/DCM = 10/1)to afford intermediate product **S2a**.

Procedure 2: To a solution of **S2a** in N-methylpyrrolidone (NMP, 7 M) was added *t*-BuOK (1.2 eq., 1M in THF) and the reaction mixture was stirred at r.t. for 0.5 h. Then, a solution of **S2b** (1.2 eq.) was added to the mixture, which was stirred at r.t. for another2 h. After the completion of the reaction which was indicated by TLC, the reaction mixture was quenched with H_2O and the aqueous layer was extracted with EtOAc (3×10 mL). The combined organic layer was dried over anhydrous Na_2SO_4 and then concentrated under reduced pressure. The residue was purified through flash column chromatography on silica gel (hexane/ethyl acetate = 20/1) to afford intermediate product **S3a**.

Procedure 3: To a solution of **S3a** in AcOH (NMP, 7 M) was added **S3b** (2.0 eq.) and the reaction mixture was stirred at 110 °C for 8-12 h. The reaction mixture was quenched with $NaHCO_3$ and the aqueous layer was extracted with EtOAc after the reaction cool down. The organic layer was dried with anhydrous Na_2SO_4 and then concentrated under reduced pressure. The residue was purified through flash column chromatography on silica gel (hexane/ethyl acetate = 10/1)to afford pure product **1**.

Characterization Data of Products

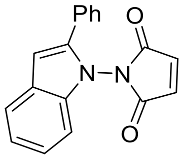

1-(2-Phenyl-1H-indol-1-yl)-1H-pyrrole-2,5-dione (1a)

A yellow solid. **^{1}H NMR** (500 MHz, $CDCl_3$) δ 7.68-7.64 (m, 1H), 7.43 (dd, *J* = 8.0, 1.6 Hz, 2H), 7.41-7.35 (m, 3H), 7.27-7.21 (m, 2H), 7.06 (d, *J* = 7.4 Hz, 1H), 6.81 (d, *J* = 1.2 Hz, 2H), 6.75 (s, 1H). **^{13}C NMR** (125 MHz, $CDCl_3$) δ 166.8, 141.6, 138.1, 133.3, 130.5, 128.9, 128.8, 128.4, 127.2, 123.8, 122.4, 121.6, 108.8, 103.7. **HRMS-EI (m/z):** Calcd for $[C_{18}H_{12}N_2O_2, M]^+$: 288.0899; found 288.0885.

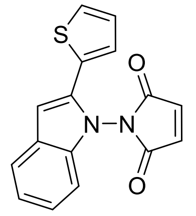

1-(2-(Thiophen-2-yl)-1H-indol-1-yl)-1H-pyrrole-2,5-dione (1b)

A yellow solid. **^{1}H NMR** (500 MHz, $CDCl_3$) δ 7.67-7.62 (m, 1H), 7.33 (m, 1H), 7.26-7.20 (m, 2H), 7.18-7.16 (m, 1H), 7.09-7.03 (m, 2H), 6.92 (s, 2H), 6.87 (s, 1H). **^{13}C NMR** (126 MHz, $CDCl_3$) δ 166.5, 137.6, 134.0, 133.4, 131.1, 127.8, 126.7, 126.6, 126.4, 124.0, 122.3, 121.4, 108.4, 103.5, 77.3, 77.1, 76.8. **HRMS-EI (m/z):** Calcd for [$C_{16}H_{10}N_2O_2S$, M]$^+$: 294.0463, found 294.0462.

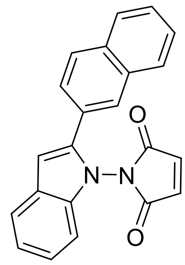

1-(2-(Naphthalen-2-yl)-1H-indol-1-yl)-1H-pyrrole-2,5-dione (1c)

A yellow solid. **^{1}H NMR** (500 MHz, $CDCl_3$) δ 7.93 (s, 1H), 7.90-7.84 (m, 3H), 7.75-7.68 (m, 1H), 7.58 (m, 1H), 7.53 (m, 2H), 7.34-7.24 (m, 2H), 7.12 (d, J = 7.5 Hz, 1H), 6.89 (s, 1H), 6.80 (s, 2H). **^{13}C NMR** (125 MHz, $CDCl_3$) δ 166.8, 141.7, 138.4, 133.4, 133.3, 128.7, 128.4, 127.9, 127.6, 127.3, 126.8, 126.7, 125.9, 123.9, 122.5, 121.6, 108.8, 104.2. **HRMS-EI (m/z):** Calcd for [$C_{22}H_{14}N_2O_2$, M]$^+$: 338.1055, found 338.1051.

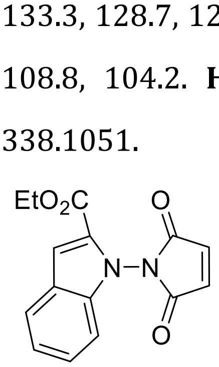

Ethyl 1-(2,5-dioxo-2,5-dihydro-1H-pyrrol-1-yl)-1H-indole-2-carboxylate (1d)

A yellow solid. **^{1}H NMR** (500 MHz, $CDCl_3$) δ 7.75 (d, J = 7.8 Hz, 1H), 7.48 (s, 1H), 7.43 (t, J = 7.7 Hz, 1H), 7.28 (t, J = 7.7 Hz, 2H), 6.98 (s, 2H), 4.31 (q, J = 7.1 Hz, 2H), 1.35 (t, J = 7.1 Hz, 3H). **^{13}C NMR** (125 MHz, $CDCl_3$) δ 167.2, 160.4, 139.4, 133.9, 127.1, 126.0, 124.5, 123.2, 122.7, 112.0, 109.2, 61.2, 14.3. **HRMS-EI (m/z):** Calcd

for [$C_{15}H_{12}N_2O_4$, M]$^+$: 284.0797, found 284.0789.

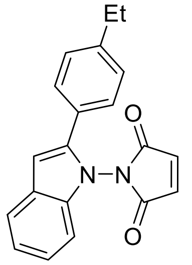

1-(2-(4-Ethylphenyl)-1H-indol-1-yl)-1H-pyrrole-2,5-dione (1e)

A yellow solid. **^{1}H NMR** (500 MHz, $CDCl_3$) δ 7.71-7.63 (m, 1H), 7.41-7.36 (m, 2H), 7.26 (m, 4H), 7.13-7.04 (m, 1H), 6.75 (s, 2H), 2.70 (q, J = 7.6 Hz, 1H), 1.28 (t, *J* = 7.6 Hz, 2H). **^{13}C NMR** (125 MHz, $CDCl_3$) δ 166.8, 145.0, 141.7, 138.0, 133.1, 128.4, 128.2, 127.7, 127.2, 123.5, 122.3, 121.4, 108.7, 103.2, 28.7, 15.4. **HRMS-EI (m/z):** Calcd for [$C_{20}H_{16}N_2O_2$, M]$^+$: 316.1212, found 316.1207.

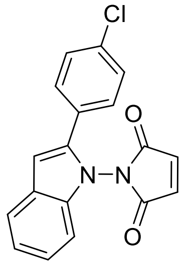

1-(2-(4-Chlorophenyl)-1H-indol-1-yl)-1H-pyrrole-2,5-dione (1f)

A yellow solid. **^{1}H NMR** (500 MHz, $CDCl_3$) δ 7.63-7.52 (m, 1H), 7.28 (s, 4H), 7.16 (m, 3H), 6.97 (d, *J* = 7.3 Hz, 1H), 6.73 (s, 2H), 6.66 (s, 1H). **^{13}C NMR** (125 MHz, $CDCl_3$) δ 166.8, 140.3, 138.2, 134.9, 133.3, 129.6, 129.2, 128.9, 127.1, 124.1, 122.6, 108.8, 104.1. **HRMS-EI (m/z):** Calcd for [$C_{18}H_{11}ClN_2O_2$, M]$^+$: 322.0509, found 322.0507.

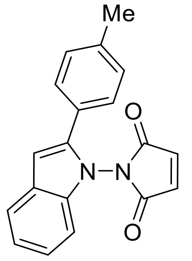

1-(2-(p-Tolyl)-1H-indol-1-yl)-1H-pyrrole-2,5-dione (1g)

A yellow solid. **^{1}H NMR** (400 MHz, $CDCl_3$) δ 7.60-7.52 (m, 1H), 7.23 (d, J = 8.1 Hz, 2H), 7.20-7.07 (m, 5H), 7.02-6.94 (m, 1H), 6.73 (s, 2H), 6.66-6.61 (m, 1H), 2.29 (s, 3H). **^{13}C NMR** (125 MHz, $CDCl_3$) δ 166.8, 141.6, 138.8, 138.1, 133.2, 129.6, 128.2,

127.5, 127.3, 123.6, 122.3, 121.4, 108.7, 103.2, 21.4. **HRMS-EI (m/z):** Calcd for [$C_{19}H_{14}N_2O_2$, M]$^+$: 302.1055, found 302.1063.

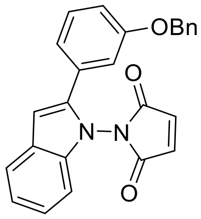

1-(2-(3-(Benzyloxy)phenyl)-1H-indol-1-yl)-1H-pyrrole-2,5-dione (1h)

A yellow solid. ^{1}**H NMR** (400 MHz, $CDCl_3$) δ 7.61-7.53 (m, 1H), 7.38-7.30 (m, 3H), 7.26-7.11 (m, 5H), 7.03-6.86 (m, 4H), 6.68 (s, 2H), 6.65 (d, *J* = 0.7 Hz, 1H).4.96 (s, 3H). ^{13}**C NMR** (100 MHz, $CDCl_3$) δ 166.8, 159.1, 141.4, 138.2, 136.8, 133.2, 131.7, 130.0, 128.8, 128.2, 127.6, 127.2, 123.8, 122.4, 121.6, 120.9, 115.9, 114.2, 108.8, 103.9, 70.1. **MS-EI (m/z):** Calcd for [$C_{25}H_{18}N_2O_3$, H+M]$^+$: 395.1390, found 395.1389.

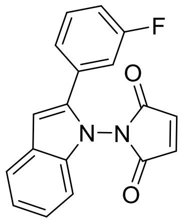

1-(2-(3-Fluorophenyl)-1H-indol-1-yl)-1H-pyrrole-2,5-dione (1i)

A yellow solid. ^{1}**H NMR** (500 MHz, $CDCl_3$) δ 7.69 (d, *J* = 7.2 Hz, 1H), 7.38 (m, *J* = 7.9, 6.0 Hz, 1H), 7.30-7.25 (m, 2H), 7.23 (d, *J* = 7.7 Hz, 1H), 7.20-7.15 (m, 1H), 7.08 (m, *J* = 8.2, 3.0 Hz, 2H), 6.85 (s, 2H), 6.81 (s, 1H). ^{13}**C NMR** (125 MHz, $CDCl_3$) δ 166.7, 163.9, 140.2, 138.2, 133.3, 132.5, 130.6 (d, *J* = 8.4 Hz), 127.0, 124.2, 123.9, 122.6, 121.7, 115.7 (d, *J* = 21.1 Hz), 115.2 (d, *J* = 22.6 Hz), 108.8, 104.4. ^{19}**F NMR** (471 MHz, CDCl3) δ -111.94. **HRMS-EI (m/z):** Calcd for [$C_{18}H_{11}FN_2O_2$, M]$^+$: 306.0805, found 306.0804.

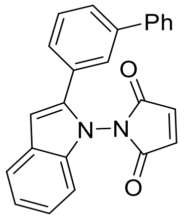

1-(2-([1,1'-Biphenyl]-3-yl)-1H-indol-1-yl)-1H-pyrrole-2,5-dione (1j)

A yellow solid. **^{1}H NMR** (400 MHz, $CDCl_3$) δ 7.60 (s, 1H), 7.53 (m, *J* = 6.9, 4.0 Hz, 1H), 7.36-7.21 (m, 5H), 7.17-7.06 (m, 3H), 6.96 (dt, *J* = 6.4, 3.2 Hz, 1H), 6.68 (s, 1H), 6.58 (s, 1H). **^{13}C NMR** (100 MHz, $CDCl_3$) δ 166.8, 141.7, 141.5, 140.3, 138.1, 133.2, 130.9, 129.3, 129.0, 127.8, 127.4, 127.14, 127.1, 127.04, 127.0, 108.8, 103.8. **HRMS-EI (m/z):** Calcd for $[C_{24}H_{16}N_2O_2, M]^+$: 364.1212, found 364.1209.

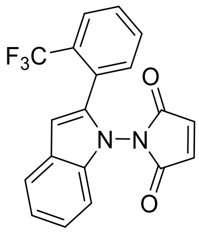

1-(2-(2-(Trifluoromethyl)phenyl)-1H-indol-1-yl)-1H-pyrrole-2,5-dione (1k)

^{1}H NMR (500 MHz, $CDCl_3$) δ 7.70 (d, *J* = 6.6 Hz, 1H), 7.59 (d, *J* = 7.1 Hz, 1H), 7.41 (m, 3H), 7.20-7.13 (m, 2H), 6.93 (d, *J* = 7.7 Hz, 1H), 6.71 (s, 1H), 6.67 (s, 2H). **^{13}C NMR** (100 MHz, $CDCl_3$) δ 166.4, 136.8, 136.1, 133.1, 132.5, 131.3, 130.5 (d, *J* = 30.4 Hz), 129.2,128.6 (d, *J* = 1.7 Hz), 126.8 (d, *J* = 5.3 Hz), 126.4, 124.1, 122.2, 123.7 (d, *J* = 272.3 Hz), 121.8, 108.4, 106.3 (d, *J* = 2.9 Hz). **^{19}F NMR** (377 MHz, CDCl3) δ -57.50. **HRMS-EI (m/z):** Calcd for $[C_{19}H_{11}F_3N_2O_2, M]^+$: 356.0773, found 356.0770.

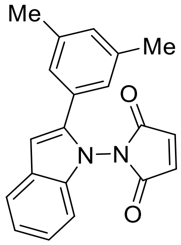

1-(2-(3,5-Dimethylphenyl)-1H-indol-1-yl)-1H-pyrrole-2,5-dione (1l)

^{1}H NMR (400 MHz, $CDCl_3$) δ 7.54 (dt, *J* = 6.7, 3.9 Hz, 1H), 7.13 (ddd, *J* = 8.9, 4.0, 2.7 Hz, 2H), 6.96 (d, *J* = 8.9 Hz, 2H), 6.68 (s, 1H), 6.62 (s, 1H), 2.21 (s, 4H). **^{13}C NMR** (100 MHz, $CDCl_3$) δ 166.8, 141.9, 138.4 133.18, 130.5, 127.2, 123.6, 121.4, 108.7, 103.4, 21.4. **HRMS-EI (m/z):** Calcd for $[C_{20}H_{16}N_2O_2, M]^+$: 316.1212, found 316.1208.

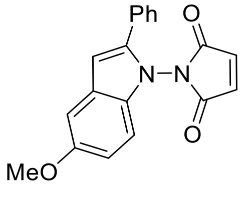

1-(5-Methoxy-2-phenyl-1H-indol-1-yl)-1H-pyrrole-2,5-dione (1m)

^{1}H NMR (500 MHz, $CDCl_3$) δ 7.46-7.32 (m, 5H), 7.12 (d, *J* = 2.3 Hz, 1H), 6.97 (d, *J* = 8.8 Hz, 1H), 6.89 (dd, *J* = 8.8, 2.3 Hz, 1H), 6.76 (s, 2H), 6.69 (s, 1H), 3.86 (s, 3H). **^{13}C NMR** (125 MHz, $CDCl_3$) δ 166.9, 156.0, 142.5, 133.2, 130.5, 128.9, 128.7, 128.2, 127.9, 113.5, 109.6, 103.7, 103.6, 56.0. **HRMS-EI (m/z):** Calcd for [$C_{19}H_{14}N_2O_3$, M]$^+$: 318.1004, found 318.1005.

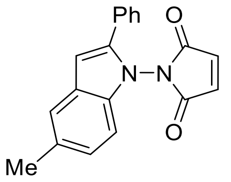

1-(5-Methyl-2-phenyl-1H-indol-1-yl)-1H-pyrrole-2,5-dione (1n)

^{1}H NMR (400 MHz, $CDCl_3$) δ 7.44 (d, *J* = 8.0 Hz, 1H), 7.33 (dd, *J* = 7.9, 1.8 Hz, 2H), 7.31-7.25 (m, 3H), 6.98-6.94 (m, 1H), 6.76 (s, 1H), 6.71 (s, 2H), 6.61 (d, *J* = 0.7 Hz, 1H), 2.35 (s, 3H). **^{13}C NMR** (100 MHz, $CDCl_3$) δ 166.8, 140.8, 138.5, 133.8, 133.1, 130.5, 128.8, 128.5, 128.1, 124.9, 124.0, 121.1, 108.7, 103.4, 21.8. **HRMS-EI (m/z):** Calcd for [$C_{19}H_{14}N_2O_2$, M]$^+$: 302.1055, found 302.1052.

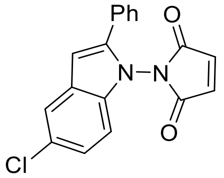

1-(5-Chloro-2-phenyl-1H-indol-1-yl)-1H-pyrrole-2,5-dione (1o)

^{1}H NMR (500 MHz, $CDCl_3$) δ 7.63 (s, 1H), 7.48-7.31 (m, 6H), 7.20 (d, *J* = 8.4 Hz, 1H), 6.99 (d, *J* = 8.5 Hz, 1H), 6.81 (s, 2H), 6.69 (s, 1H). **^{13}C NMR** (125 MHz, $CDCl_3$) δ 166.5, 142.9, 136.5, 133.3, 129.9, 129.1, 129.0, 128.4, 128.3, 128.0, 124.0, 121.0, 109.9, 103.1. **HRMS-EI (m/z):** Calcd for [$C_{18}H_{11}ClN_2O_2$, M]$^+$: 322.0509, found 322.0506.

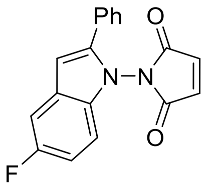

1-(5-Fluoro-2-phenyl-1H-indol-1-yl)-1H-pyrrole-2,5-dione (1p)

^{1}H NMR (500 MHz, $CDCl_3$) δ 7.43-7.37 (m, 5H), 7.32-7.29 (m, 1H), 6.99 (dd, *J* = 7.5, 1.7 Hz, 2H), 6.81 (s, 2H), 6.71 (s, 1H). **^{13}C NMR** (125 MHz, $CDCl_3$) δ 166.7, 160.3,

158.4, 143.3, 134.5, 133.3, 130.1, 129.0 (d, *J* = 10.1 Hz), 128.3, 127.8 (d, *J* = 10.4 Hz), 111.9 (d, *J* = 26.5 Hz), 109.6 (d, *J* = 9.7 Hz), 106.8 (d, *J* = 24.2 Hz), 103.6 (d, *J* = 4.2 Hz). **^{19}F NMR** (471 MHz, CDCl3) δ -121.73. **HRMS-EI (m/z):** Calcd for [$C_{18}H_{11}FN_2O_2$, M]$^+$: 306.0805, found 306.0803.

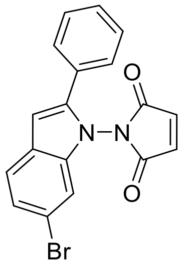

1-(6-Bromo-2-phenyl-1H-indol-1-yl)-1H-pyrrole-2,5-dione (1q)

^{1}H NMR (400 MHz, $CDCl_3$) δ 7.45-7.41 (m, 1H), 7.33-7.29 (m, 5H), 7.25 (dd, *J* = 8.4, 1.7 Hz, 1H), 7.16-7.14 (m, 1H), 6.73 (s, 2H), 6.63 (d, *J* = 0.9 Hz, 1H). **^{13}C NMR** (100 MHz, $CDCl_3$) δ 166.5, 142.2, 138.8, 133.3, 129.9, 129.1, 129.0, 128.4, 126.1, 125.8, 122.7, 117.2, 112.0, 103.5. **HRMS-EI (m/z):** Calcd for [$C_{18}H_{11}BrN_2O_2$, M]$^+$: 366.0004, found 366.0012.

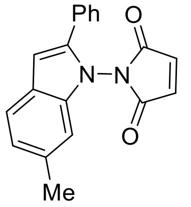

1-(6-Methyl-2-phenyl-1H-indol-1-yl)-1H-pyrrole-2,5-dione (1r)

^{1}H NMR (400 MHz, $CDCl_3$) δ 7.37-7.31 (m, 3H), 7.30-7.24 (m, 3H), 6.97 (d, *J* = 8.3 Hz, 1H), 6.86 (d, *J* = 8.3 Hz, 1H), 6.66 (s, 2H), 6.58 (s, 1H), 2.36 (s, 3H). **^{13}C NMR** (100 MHz, $CDCl_3$) δ 166.8, 141.7, 136.5, 133.1, 131.7, 130.5, 128.8, 128.6, 128.1, 127.4, 125.1, 121.3, 108.4, 103.3, 21.5. **HRMS-EI (m/z):** Calcd for [$C_{19}H_{14}N_2O_2$, M]$^+$: 302.1055, found 302.1050.

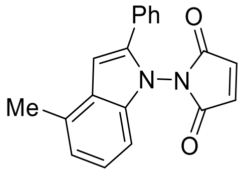

1-(4-Methyl-2-phenyl-1H-indol-1-yl)-1H-pyrrole-2,5-dione (1s)

^{1}H NMR (500 MHz, $CDCl_3$) δ 7.44 (dt, *J* = 8.1, 2.1 Hz, 2H), 7.41-7.34 (m, 3H), 7.18-

7.13 (m, 1H), 7.03 (d, *J* = 7.2 Hz, 1H), 6.90 (d, *J* = 8.1 Hz, 1H), 6.81-6.75 (m, 3H), 2.59 (s, 3H). **^{13}C NMR** (125 MHz, $CDCl_3$) δ 166.8, 140.9, 137.8, 133.2, 131.2, 130.6, 128.9, 128.7, 128.3, 126.9, 123.9, 122.7, 106.3, 102.2, 18.5. **HRMS-EI (m/z):** Calcd for [$C_{19}H_{14}N_2O_2$, M]$^+$: 302.1055, found 302.1053.

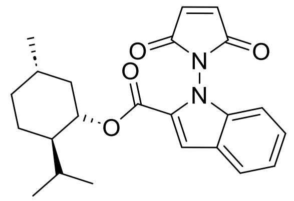

(1*S*,2*R*,5*S*)-2-Isopropyl-5-methylcyclohexyl 1-(2,5-dioxo-2,5-dihydro-1H-pyrrol-

1-yl)-1H-indole-2-carboxylate (1t)

^{1}H NMR (500 MHz, $CDCl_3$) δ 7.75 (d, *J* = 8.1 Hz, 1H), 7.48 (s, 1H), 7.42 (d, *J* = 7.7 Hz, 1H), 7.28 (dd, J = 7.6, 4.8 Hz, 2H), 6.94 (s, 2H), 4.89 (td, *J* = 10.9, 4.3 Hz, 1H), 2.08 (d, *J* = 11.8 Hz, 1H), 1.94 (td, J = 6.9, 2.4 Hz, 1H), 1.73 (d, *J* = 10.6 Hz, 2H), 1.56-1.46 (m, 2H), 1.15-1.04 (m, 2H), 0.94 (d, *J* = 6.5 Hz, 7H), 0.80 (d, *J* = 6.9 Hz, 3H). **^{13}C NMR** (125 MHz, $CDCl_3$) δ 167.1, 167.0, 160.0, 139.3, 133.7, 126.9, 126.3, 124.5, 123.1, 122.6, 111.7, 109.1, 75.2, 47.2, 40.8, 34.2, 31.5, 26.4, 23.6, 22.0, 20.7, 16.5. **HRMS-EI (m/z):** Calcd for [$C_{23}H_{26}N_2O_4$, M]$^+$: 394.1887, found 394.1885.

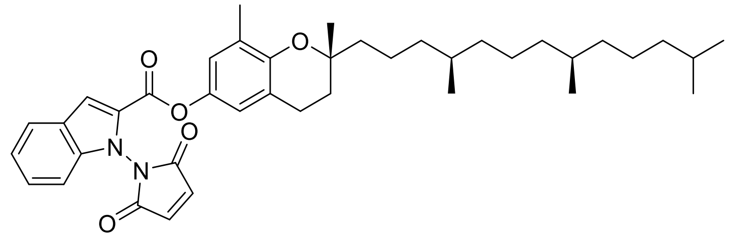

(R)-2,8-Dimethyl-2-((4R,8R)-4,8,12-trimethyltridecyl)chroman-6-yl 1-(2,5-dioxo-

(R)-2,5-Dihydro-1H-pyrrol-1-yl)-1H-indole-2-carboxylate (1u)

^{1}H NMR (400 MHz, $CDCl_3$) δ 7.78 (d, *J* = 7.9 Hz, 1H), 7.64 (s, 1H), 7.45 (s, 1H), 7.29 (d, *J* = 8.2 Hz, 2H), 6.91 (s, 2H), 6.76 (d, *J* = 2.4 Hz, 1H), 6.71 (d, *J* = 2.6 Hz, 1H), 2.74 (s, 2H), 2.17 (s, 3H), 1.78 (m, 2H), 1.65-1.48 (m, 4H), 1.46-1.29 (m, 10H), 1.27-1.03 (m, 10H), 0.95-0.84 (m, 14H). **^{13}C NMR** (125 MHz, $CDCl_3$) δ 167.1, 159.7, 150.2, 141.7, 139.7, 133.9, 127.6, 127.5, 125.4, 124.5, 123.4, 122.9, 121.3, 121.2, 119.3,

113.0, 109.3, 76.3, 40.3, 39.5, 37.6, 37.4, 32.9, 32.8, 31.0, 28.1, 24.9, 24.6, 24.4, 22.9, 22.8, 22.5, 21.1, 19.9, 19.8, 16.3. **HRMS-EI (m/z):** Calcd for [$C_{40}H_{52}N_2O_5$, M]$^+$: 641.3949, found 641.395.

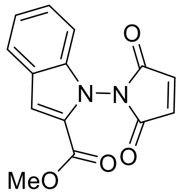

Methyl 1-(2,5-dioxo-2,5-dihydro-1H-pyrrol-1-yl)-1H-indole-2-carboxylate(1v)

^{1}H NMR (400 MHz, $CDCl_3$) δ 7.73 (d, J = 7.8 Hz, 1H), 7.46 – 7.41 (m, 1H), 7.41 (s, 11H), 7.28 (s, 1H), 7.24 (s, 1H), 6.98 (s, 2H), 3.83 (s, 3H). **^{13}C NMR** (100 MHz, $CDCl_3$) δ 167.2, 161.0, 139.5, 133.9, 127.2, 125.5, 124.5, 123.3, 122.8, 112.1, 109.3, 52.1. **HRMS-EI (m/z):** Calcd for [$C_{14}H_{10}N_2O_4$, M]$^+$: 270.0635; found: 270.064.

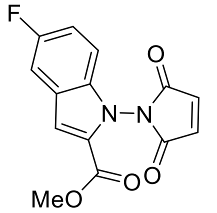

Methyl 1-(2,5-dioxo-2,5-dihydro-1H-pyrrol-1-yl)-5-fluoro-1H-indole-2-carboxylate(1w)

^{1}H NMR (400 MHz, $CDCl_3$) δ 7.41-7.34 (m, 2H), 7.24-7.12 (m, 2H), 7.00 (s, 2H), 3.84 (s, 3H). **^{13}C NMR** (100 MHz, $CDCl_3$) δ 167.1, 160.7, 136.0, 134.0, 126.9, 124.9, 116.2 (d, *J* = 27.0 Hz), 111.6 (d, *J* = 5.1 Hz), 110.5 (d, *J* = 9.5 Hz), 107.9 (d, *J* = 23.9 Hz), 52.3. **^{19}F NMR** (377 MHz, $CDCl_3$) δ -120.52. **HRMS-EI (m/z):** Calcd for [$C_{14}H_9FN_2O_4$, M]$^+$: 288.0541; found: 288.0542.

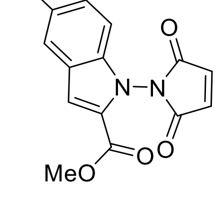

Methyl 5-bromo-1-(2,5-dioxo-2,5-dihydro-1H-pyrrol-1-yl)-1H-indole-2-carboxylate(1x)

^{1}H NMR (400 MHz, $CDCl_3$) δ 7.80 (d, *J* = 1.5 Hz, 1H), 7.42 (dd, *J* = 8.8, 1.7 Hz, 1H), 7.27 (s, 1H), 7.07 (d, *J* = 8.8 Hz, 1H), 6.92 (s, 2H), 3.76 (s, 3H). **^{13}C NMR** (100 MHz, $CDCl_3$) δ 167.0, 160.6, 138.1, 134.0, 130.2, 126.5, 126.0, 125.7, 115.9, 111.0, 110.9, 52.3. **HRMS-EI (m/z):** Calcd for [$C_{14}H_9BrN_2O_4$, M]$^+$: 347.974; found: 347.9743.

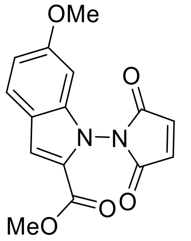

Methyl 1-(2,5-dioxo-2,5-dihydro-1H-pyrrol-1-yl)-6-methoxy-1H-indole-2-carboxylate (1y)

^{1}H NMR (400 MHz, $CDCl_3$) δ 7.57 (d, *J* = 8.8 Hz, 1H), 7.36 (d, *J* = 0.9 Hz, 1H), 7.10 - 6.93 (m, 2H), 6.89 (dd, *J* = 8.8, 2.2 Hz, 1H), 6.72-6.60 (m, 1H), 3.82 (s, 1H), 3.80 (s, 3H). **^{13}C NMR** (100 MHz, $CDCl_3$) δ 167.3, 160.9, 160.4, 140.9, 134.0, 124.2, 118.5, 114.0, 112.4, 55.7, 51.9. **HRMS-EI (m/z):** Calcd for [$C_{15}H_{12}N_2O_5$, M]$^+$: 300.0741; found: 300.0746.

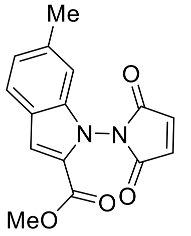

Methyl 1-(2,5-dioxo-2,5-dihydro-1H-pyrrol-1-yl)-6-methyl-1H-indole-2-carboxylate (1z)

^{1}H NMR (400 MHz, $CDCl_3$) δ 7.60 (d, J = 8.2 Hz, 1H), 7.43-7.35 (m, 1H), 7.09 (d, J = 8.2 Hz, 1H), 7.05 (s, 1H), 6.96 (s, 2H), 3.82 (s, 3H), 2.46 (s, 3H). **^{13}C NMR** (100 MHz, $CDCl_3$) δ 167.2, 161.0, 139.9, 137.8, 133.8, 124.9, 124.9, 122.9, 122.3, 112.0, 108.9, 51.9, 22.1. **HRMS-EI (m/z):** Calcd for [$C_{15}H_{12}N_2O_4$, M]$^+$: 284.0792; found: 284.0794.

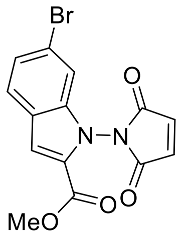

Methyl 6-bromo-1-(2,5-dioxo-2,5-dihydro-1H-pyrrol-1-yl)-1H-indole-2-carboxylate(1aa)

^{1}H NMR (400 MHz, $CDCl_3$) δ 7.57 (d, *J* = 8.5 Hz, 1H), 7.44 (s, 1H), 7.37 (d, *J* = 0.9 Hz, 11H), 7.35 (d, *J* = 1.7 Hz, 1H), 6.99 (s, 2H), 3.82 (s, 3H). **^{13}C NMR** (100 MHz, $CDCl_3$) δ 67.0, 160.6, 140.0, 134.0, 126.4, 126.0, 124.5, 123.3, 121.2, 112.5, 111.8, 52.2. **HRMS-EI (m/z):** Calcd for [$C_{14}H_9BrN_2O_4$, M]$^+$: 347.974; found: 347.9741.

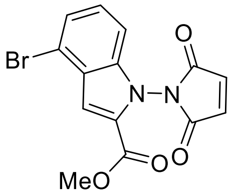

Methyl 4-bromo-1-(2,5-dioxo-2,5-dihydro-1H-pyrrol-1-yl)-1H-indole-2-carboxylate(1ab)

^{1}H NMR (400 MHz, $CDCl_3$) δ 7.48 (s, 1H), 7.46-7.43 (m, 1H), 7.22 (dd, J = 15.0, 7.8 Hz, 2H), 6.98 (s, 2H), 3.85 (s, 3H). **^{13}C NMR** (100 MHz, $CDCl_3$) δ166.9, 160.7, 139.7, 134.0, 127.9, 126.0, 125.7, 125.4, 117.4, 111.8, 108.5, 52.3. **HRMS-EI (m/z):** Calcd for [$C_{14}H_9BrN_2O_4$, M]$^+$: 347.974; found: 347.9746.

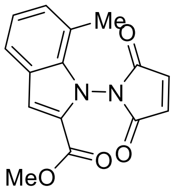

Methyl 1-(2,5-dioxo-2,5-dihydro-1H-pyrrol-1-yl)-7-methyl-1H-indole-2-Carboxylate(1ac)

^{1}H NMR (400 MHz, $CDCl_3$) δ 7.70-7.49 (m, 1H), 7.42 (s, 1H), 7.13 (d, *J* = 5.8 Hz, 1H), 6.98 (s, 1H), 3.82 (s, 2H), 2.39 (s, 3H). **^{13}C NMR** (126 MHz, $CDCl_3$) δ 167.5, 161.0, 138.0, 134.0, 129.5, 125.2, 122.7, 121.4, 121.1, 112.8, 52.0, 17.0. **HRMS-EI (m/z):** Calcd for [$C_{15}H_{12}N_2O_4$, M]$^+$: 284.0792; found 284.0795.

General procedure A

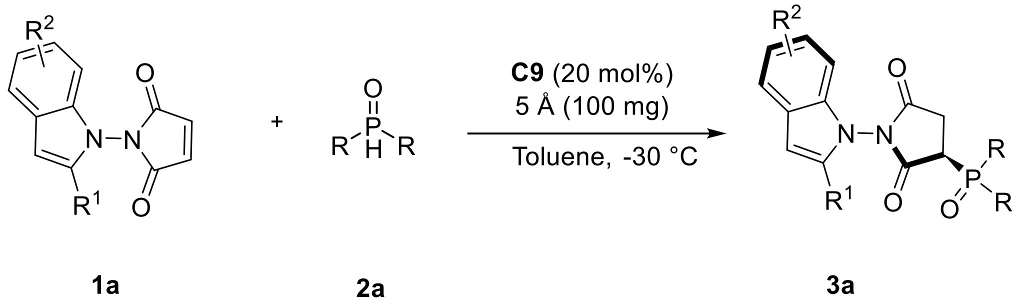

To a mixture of **1a** (0.1 mmol, 1.00 equiv.), **C9** (0.02 mmol, 20 mol%), 5 Å (100 mg) in toluene (1.00 mL) in a dry tube was added **2a** (0.12 mmol, 1.20 equiv.) in toluene (1.00 mL) dropwise under argon atmosphere at -30 °C. After stirring for 24h the reaction was diluted with dichloromethane, filtered and collect the filtrate. Evaporation on the rotavapor gave the crude product, then purification by column.

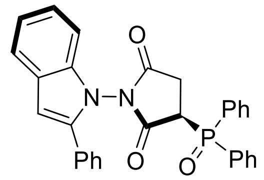

(*P,R*)-3-(Diphenylphosphoryl)-1-(2-phenyl-1H-indol-1-yl)pyrrolidine-2,5-dione (3a)

Prepared according to the general procedure A in 85% yield as a white solid. **^{1}H NMR** (500 MHz, $CDCl_3$) δ 7.95-7.87 (m, 2H), 7.86-7.80 (m, 2H), 7.64-7.55 (m, 5H), 7.49 (dt, *J* = 7.7, 3.9 Hz, 2H), 7.45 (dd, *J* = 7.7, 1.6 Hz, 2H), 7.42-7.36 (m, 3H), 7.25-7.19 (m, 2H), 7.04 (d, *J* = 8.0 Hz, 1H), 6.72 (s, 1H), 3.94 (m, 1H), 3.29 (m, 1H), 3.02-2.82 (m, 1H). **^{13}C NMR** (125 MHz, $CDCl_3$) δ 171.1, 169.5 (d, *J* = 4.5 Hz), 140.8, 137.4, 133.1 (d, *J* = 2.7 Hz), 133.0 (d, *J* = 2.6 Hz), 131.9 (d, *J* = 9.9 Hz), 131.2 (d, *J* = 9.7 Hz), 130.4, 129.4, 129.3, 129.1, 129.0, 128.9, 128.7, 128.2, 127.1, 124.1, 122.3, 121.1, 109.6, 104.0, 40.9 (d, *J* = 59.6 Hz), 28.3. **^{31}P NMR:** (162 MHz, $CDCl_3$) δ 30.53. **HRMS (ESI):** m/z Calcd for $[C_{30}H_{24}N_2O_3P, M+H]^+$: 491.1519; Found: 491.1518. $[\alpha]_D^{25}$ = +21.4 (c = 1.8, $CHCl_3$). **HPLC:** 92% ee. (HPLC condition: Chiralpak AD column, *n*-hexane/*i*-PrOH = 70:30, 1 mL/min, 220 nm, major enantiomer t_r = 7.53 min, minor enantiomer t_r = 8.73 min.)

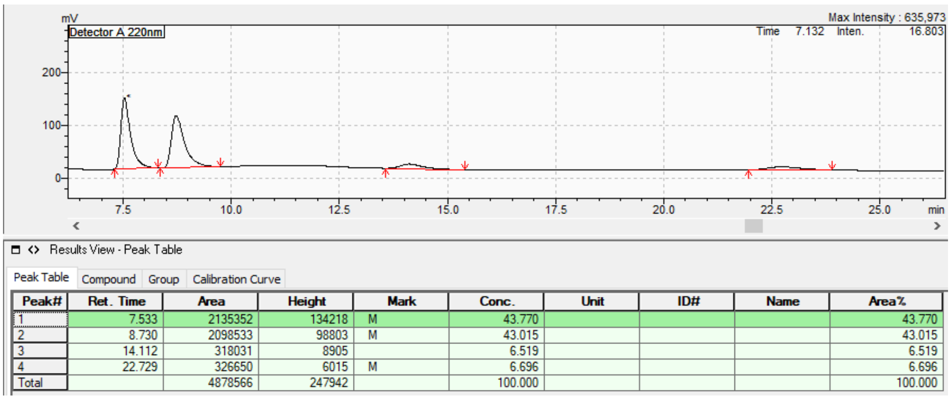

Peak#	Ret. Time	Area	Height	Mark	Conc.	Unit	ID#	Name	Area%
1	7.533	2135352	134218	M	43.770				43.770
2	8.730	2098533	98803	M	43.015				43.015
3	14.112	318031	8905		6.519				6.519
4	22.729	326650	6015	M	6.696				6.696
Total		4878566	247942		100.000				100.000

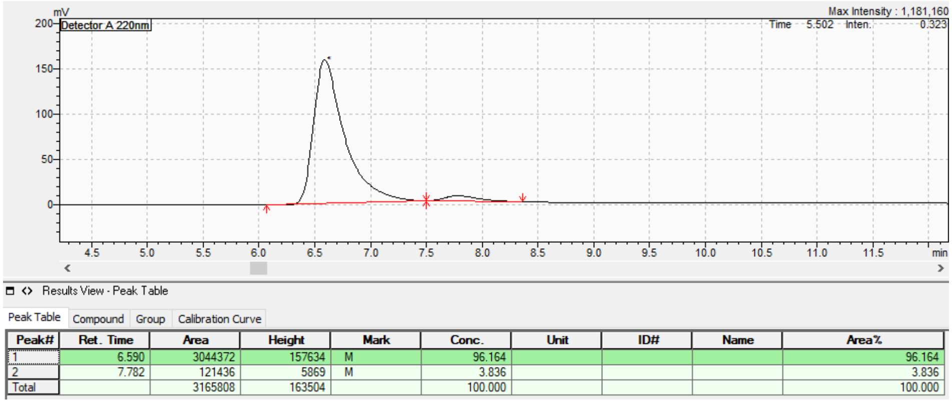

Peak#	Ret. Time	Area	Height	Mark	Conc.	Unit	ID#	Name	Area%
1	6.590	3044372	157634	M	96.164				96.164
2	7.782	121436	5869	M	3.836				3.836
Total		3165808	163504		100.000				100.000

The enantiomerisation barrier was obtained by kinetic of racemisation of an enantiomer. The slope of the first order kinetic line gives the racemisation constant ($k_{racemisation}$ =2 * $k_{enantiomerisation}$). Eyring equation gives the enantiomerisation barrier ($\Delta G^{\ddagger}_{enaniomerization}$) from enantiomerisation constant ($k_{enantiomerisation}$), R = 8.31451 $J.K^{-1}mol^{-1}$, h = 6.62608 * 10^{-34} Js and kB = 1.38066 *10^{-23} J/K. de values were determined by HPLC.

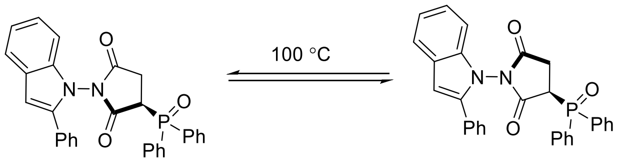

3a **3a'**

$$\Delta G^{\ddagger}\ \text{enantiomerization} = RT \times \ln \frac{K_B \times T}{h k_{\text{enantiomerisation}}}$$

Table S7. Racemization of **3a** in toluene at 100 ℃.

t	de
0	82
0.5	82
1	78
2	75
4	69

6	67

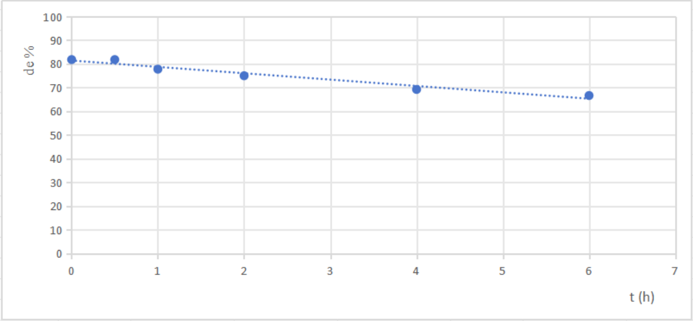

Figure S3. Racemization of **3a** in toluene at 100 ℃.

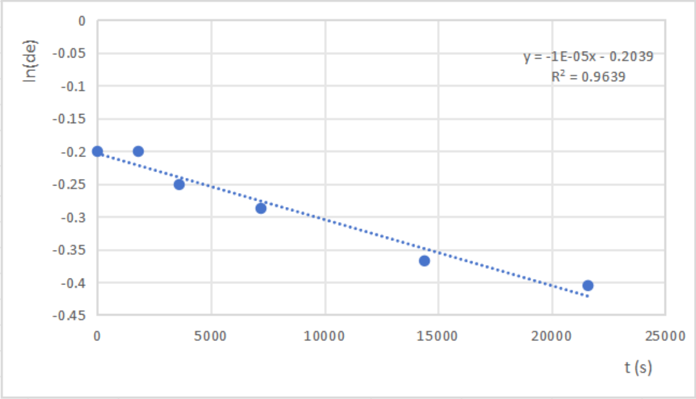

Figure S4. The plot of ln(de) vs time of **3a** at 100 ℃.

T=373.15 K (toluene); k_{rac}= 1E-05; $t_{1/2}$=ln2/K=12.2 h; $\Delta G^{\neq}$ = 30.0 kcal/mol

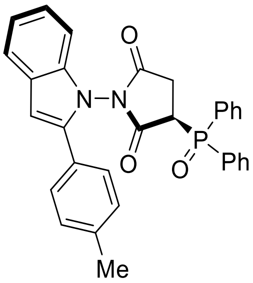

(*P*,*R*)-3-(Diphenylphosphoryl)-1-(2-(p-tolyl)-1H-indol-1-yl)pyrrolidine-2,5-dione (3b)

Prepared according to the general procedure A in 99% yield as a colorless oil. **^{1}H NMR** (500 MHz, $CDCl_3$) δ 7.96-7.90 (m, 2H), 7.83-7.77 (m, 2H), 7.61-7.53 (m, 5H), 7.49-7.44 (m, 2H), 7.31-7.28 (m, 2H), 7.19-7.15 (m, 4H), 6.92 (m, 1H), 6.66 (d, *J* = 0.8 Hz, 1H), 3.92 (td, *J* = 10.9, 3.1 Hz, 1H), 3.29 (td, *J* = 18.1, 16.9, 3.2 Hz, 1H), 2.95 (dt, *J* = 19.4, 10.1 Hz, 1H), 2.36 (s, 3H). **^{13}C NMR** (125 MHz, $CDCl_3$) δ 171.1, 169.5 (d, *J* = 4.3 Hz), 140.9, 138.7, 137.3, 133.1, 131.9 (d, *J* = 9.9 Hz), 131.3 (d, *J* = 9.6 Hz),

129.6, 129.4, 129.3, 129.1, 129.0, 128.1, 127.7, 127.5, 127.1, 123.9, 122.2, 121.0, 109.4, 103.5, 41.0 (d, *J* = 60.0 Hz), 28.3, 21.4. **^{31}P NMR:** (162 MHz, $CDCl_3$) δ 30.65. **HRMS (ESI):** m/z Calcd for $[C_{31}H_{25}N_2O_3P, M+H]^+$: 505.1676; Found: 505.1675. $[\alpha]^{25}_{D}$ = +17.18 (c = 2.2, $CHCl_3$). **HPLC:** 90% ee. (HPLC condition: Chiralpak AD column, *n*-hexane/*i*-PrOH = 65:35, 1 mL/min, 220 nm, major enantiomer t_r = 7.3 min, minor enantiomer t_r = 8.1 min.)

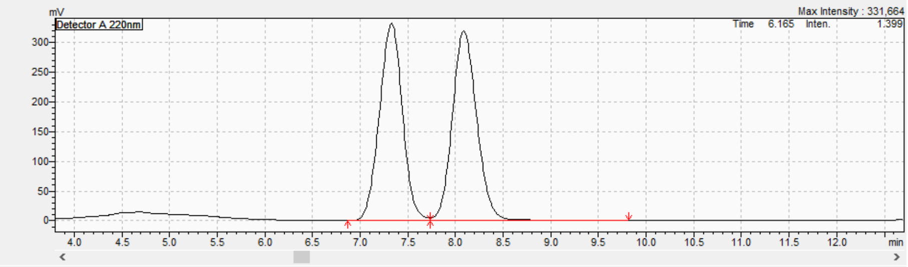

Results View - Peak Table

Peak Table | Compound | Group | Calibration Curve

Peak#	Ret. Time	Area	Height	Mark	Conc.	Unit	ID#	Name	Area%
1	7.330	5557327	330998		49.535				49.535
2	8.087	5661753	318378	V	50.465				50.465
Total		11219080	649376		100.000				100.000

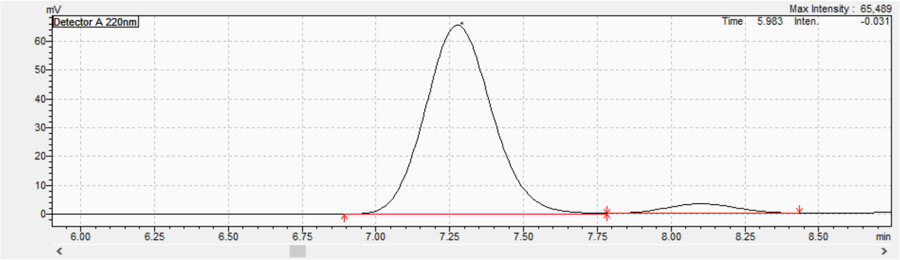

Results View - Peak Table

Peak Table | Compound | Group | Calibration Curve

Peak#	Ret. Time	Area	Height	Mark	Conc.	Unit	ID#	Name	Area%
1	7.277	1031644	65452	M	95.032				95.032
2	8.101	53926	3260	M	4.968				4.968
Total		1085570	68712		100.000				100.000

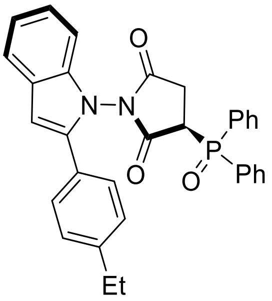

(*P*,*R*)-3-(Diphenylphosphoryl)-1-(2-(4-ethylphenyl)-1H-indol-1-yl)pyrrolidine-2,5-dione (3c)

Prepared according to the general procedure A in 92.5% yield as a colorless oil. **^{1}H NMR** (500 MHz, $CDCl_3$) δ 7.94 (dd, J = 6.8, 5.3 Hz, 2H), 7.87-7.78 (m, 2H), 7.65- 7.49 (m, 5H), 7.47 (m, 2H), 7.37-7.27 (m, 2H), 7.23-7.15 (m, 4H), 6.93 (m, 1H), 6.66 (s, 1H), 3.92 (m, 1H), 3.29 (m, 1H), 2.96 (m, 1H), 2.66 (d, *J* = 7.6 Hz, 2H), 1.25 (t, *J* = 7.6

Hz, 3H). **^{13}C NMR** (125 MHz, $CDCl_3$) δ 171.1, 169.5, 144.9, 140.9, 137.4, 133.1, 131.9 (d, J = 9.9 Hz), 131.3 (d, J = 9.7 Hz), 129.4, 129.3, 129.1, 129.0, 128.7, 128.4, 128.1, 127.8, 127.2, 123.9, 122.3, 121.0, 109.5, 103.6, 41.0 (d, J = 59.7 Hz), 28.7, 28.3, 15.4. **^{31}P NMR:** (202 MHz, $CDCl_3$) δ 30.48. **HRMS (ESI):** m/z Calcd for $[C_{32}H_{27}N_2O_3P, M+H]^+$: 519.1832; Found: 519.1830. $[\alpha]_D^{25}$ = +234.93 (c = 2.2, $CHCl_3$). **HPLC:** 91% ee. (HPLC condition: Chiralpak AD column, *n*-hexane/*i*-PrOH = 75:25, 1 mL/min, 220 nm, major enantiomer t_r = 10.1 min, minor enantiomer t_r = 11.8 min.)

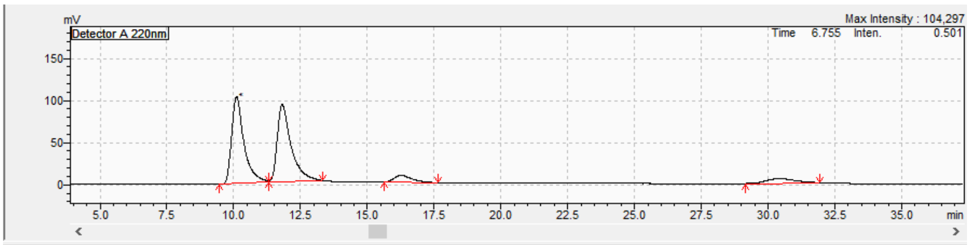

Results View - Peak Table

Peak Table | Compound | Group | Calibration Curve

Peak#	Ret. Time	Area	Height	Mark	Conc.	Unit	ID#	Name	Area%
1	10.110	3322381	102771	M	44.601				44.601
2	11.817	3332572	92179	V M	44.738				44.738
3	16.258	398069	8432	M	5.344				5.344
4	30.415	396122	5338	M	5.318				5.318
Total		7449145	208720		100.000				100.000

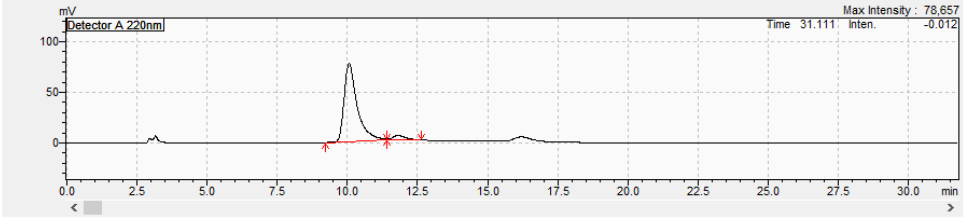

Results View - Peak Table

Peak Table | Compound | Group | Calibration Curve

Peak#	Ret. Time	Area	Height	Mark	Conc.	Unit	ID#	Name	Area%
1	10.063	2444610	76927	M	95.516				95.516
2	11.807	114760	3857	M	4.484				4.484
Total		2559370	80783		100.000				100.000

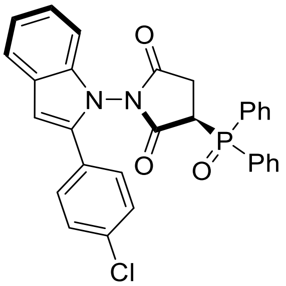

(*P*,*R*)-1-(2-(4-Chlorophenyl)-1H-indol-1-yl)-3-(diphenylphosphoryl)pyrrolidine-2,5-dione (3d)

Prepared according to the general procedure A in 99% yield as a colorless oil. **^{1}H NMR** (500 MHz, $CDCl_3$) δ 7.96-7.90 (m, 1H), 7.80 (dd, J = 12.2, 7.3 Hz, 2H), 7.72-

7.53 (m, 6H), 7.47 (dt, J = 7.5, 3.7 Hz, 2H), 7.34 (s, 3H), 7.23-7.16 (m, 2H), 7.06 (d, J = 8.0 Hz, 1H), 6.69 (s, 1H), 3.94 (td, J = 10.2, 3.4 Hz, 1H), 3.26 (m, 1H), 2.94 (m, 1H). **^{13}C NMR** (125 MHz, $CDCl_3$) δ 171.0, 169.4, 139.6, 137.5, 134.8, 133.2(d, *J* = 2.8 Hz), 133.1 (d, *J* = 2.8 Hz), 131.9, 131.8, 131.3, 131.2, 129.5, 129.4, 129.3, 129.2, 129.1, 129.0, 128.7 (d, *J* = 6.2 Hz), 127,0, 124.4, 122.5, 121.2, 109.7, 104.4, 40.9 (d, *J* = 59.5 Hz), 28.4. **^{31}P NMR:** (162 MHz, $CDCl_3$) δ 30.79. **HRMS (ESI):** m/z Calcd for [$C_{30}H_{22}ClN_2O_3P$, M+H]$^+$: 525.1129; Found: 525.1129. $[\alpha]_D^{25}$ = +6.21 (c = 2.4, $CHCl_3$). **HPLC:** 90% ee. (HPLC condition: Chiralpak AD column, *n*-hexane/*i*-PrOH = 85:15, 1 mL/min, 220 nm, major enantiomer t_r = 23.2 min, minor enantiomer t_r = 20.7 min.)

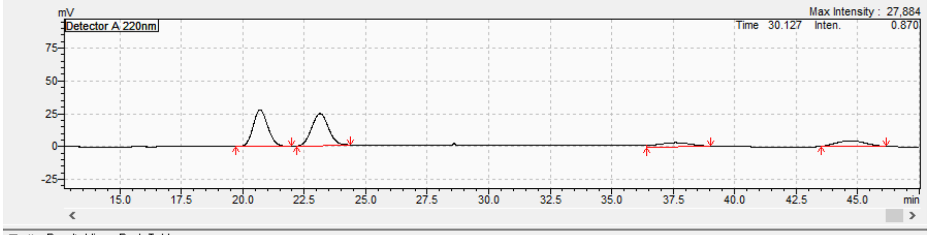

Peak#	Ret. Time	Area	Height	Mark	Conc.	Unit	ID#	Name	Area%
1	20.701	1170084	27911		39.351				39.351
2	23.142	1151931	24408	M	38.740				38.740
3	37.602	300916	3193	M	10.120				10.120
4	44.702	350550	4319	M	11.789				11.789
Total		2973481	59832		100.000				100.000

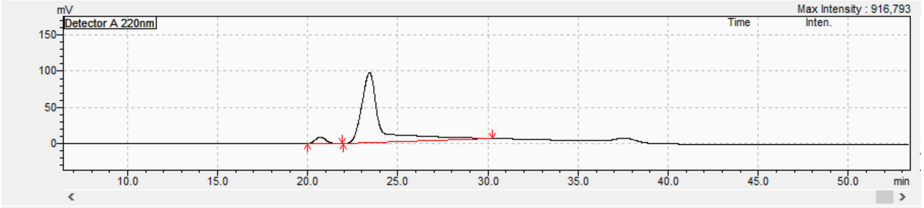

Peak#	Ret. Time	Area	Height	Mark	Conc.	Unit	ID#	Name	Area%
1	20.694	382784	9301	M	5.140				5.140
2	23.430	7064051	96787	M	94.860				94.860
Total		7446835	106089		100.000				100.000

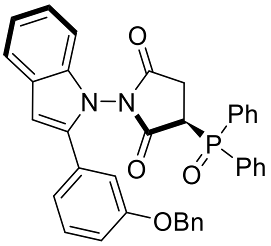

(*P*,*R*)-1-(2-(3-(Benzyloxy)phenyl)-1H-indol-1-yl)-3-(diphenylphosphoryl-)pyrrolidine-2,5-dione (3e)

Prepared according to the general procedure A in 75% yield as a colorless oil. **^{1}H NMR** (500 MHz, $CDCl_3$) δ 8.00-7.90 (m, 2H), 7.81 (dd, *J* = 11.7, 7.0 Hz, 2H), 7.68-

7.53 (m, 5H), 7.49 (m, 2H), 7.42 (m, 4H), 7.35 (m, 1H), 7.27-7.14 (m, 2H), 7.07 (s, 1H), 7.04-6.98 (m, 2H), 6.93 (d, *J* = 6.1 Hz, 1H), 6.71 (s, 1H), 5.09 (s, 2H), 3.84 (m, 1H), 3.33-3.24 (m, 1H), 2.92-2.70 (m, 1H). **^{13}C NMR** (125 MHz, $CDCl_3$) δ 171.1, 169.5, 159.0, 140.6, 137.5, 136.9, 133.1, 133.1, 132.0 (d, *J* = 9.9 Hz), 131.8, 131.4 (d, *J* = 9.7 Hz), 130.4, 130.1, 129.3 (d, *J* = 12.4 Hz), 129.01 (d, *J* = 12.5 Hz), 128.8, 128.2, 127.8, 127.5, 127.0, 124.2, 122.4, 121.1, 120.7, 115.8, 114.0, 109.5, 104.2, 69.9, 41.0 (d, *J* = 59.9 Hz), 28.3. **^{31}P NMR:** (162 MHz, $CDCl_3$) δ 30.60. **HRMS (ESI):** m/z Calcd for $[C_{37}H_{29}N_2O_4P, M+H]^+$: 597.1938; Found: 597.1935. $[\alpha]_D^{25}$ = +20.5 (c = 1.37, $CHCl_3$). **HPLC:** 97% ee. (HPLC condition: Chiralpak AD column, *n*-hexane/*i*-PrOH = 60:40, 1 mL/min, 220 nm, major enantiomer t_r = 9.7 min, minor enantiomer t_r = 41.4 min.)

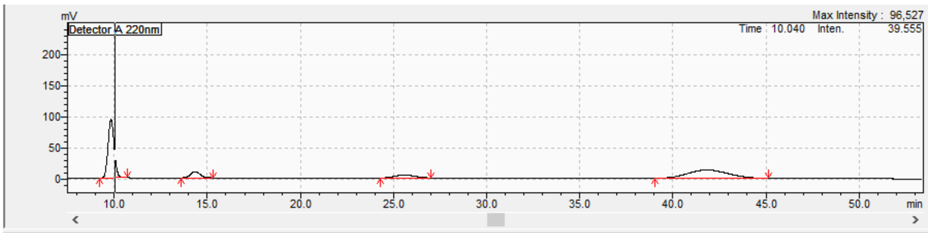

Results View - Peak Table

Peak Table | Compound | Group | Calibration Curve

Peak#	Ret. Time	Area	Height	Mark	Conc.	Unit	ID#	Name	Area%
1	9.826	2140898	93541	M	43.675				43.675
2	14.352	356334	9643	M	7.269				7.269
3	25.584	343884	4579	M	7.015				7.015
4	41.812	2060737	13460	M	42.040				42.040
Total		4901854	121223		100.000				100.000

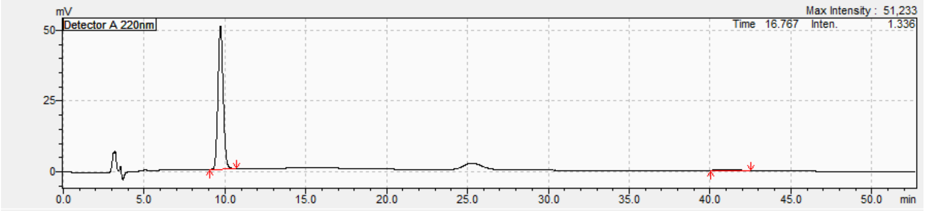

Results View - Peak Table

Peak Table | Compound | Group | Calibration Curve

Peak#	Ret. Time	Area	Height	Mark	Conc.	Unit	ID#	Name	Area%
1	9.721	1129842	50259	M	98.533				98.533
2	41.097	16818	183	M	1.467				1.467
Total		1146660	50441		100.000				100.000

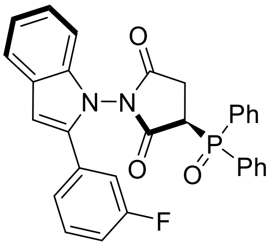

(*P*,*R*)-3-(Diphenylphosphoryl)-1-(2-(3-fluorophenyl)-1H-indol-1-yl)pyrrolidine-2,5-dione (3f)

Prepared according to the general procedure A in 74% yield as a colorless oil. **^{1}H NMR** (500 MHz, $CDCl_3$) δ 7.95-7.88 (m, 2H), 7.84-7.77 (m, 2H), 7.65-7.53 (m, 6H), 7.50-7.45 (m, 2H), 7.36-7.30 (m, 1H), 7.20 (m, 3H), 7.13 (dt, J = 9.7, 2.0 Hz, 1H), 7.04 (m, 2H), 6.73 (s, 1H), 3.96 (m, 1H), 3.29 (m, 1H), 2.97 (m, 1H). **^{13}C NMR** (125 MHz, $CDCl_3$) δ 171.0 (d, J = 3.0 Hz), 169.4 (d, J = 4.6 Hz), 164.1, 161.7, 139.4 (d, J = 2.5 Hz), 137.6, 133.2 (d, J = 2.9 Hz), 133.1 (d, J = 2.9 Hz), 132.5 (d, J = 8.4 Hz), 131.9 (d, J = 10.0 Hz), 131.3 (d, J = 9.7 Hz), 130.6 (d, J = 8.5 Hz), 129.4 (d, J = 12.5 Hz), 129.1 (d, J = 12.6 Hz), 128.5 (d, J = 5.4 Hz), 126.9, 124.55, 123.8 (d, J = 2.9 Hz), 122.5, 121.3, 115.7 (d, J = 21.1 Hz), 115.1 (d, J = 22.6 Hz), 109.7, 104.7, 41.0 (d, J = 59.5 Hz), 28.4. **^{19}F NMR** (377 MHz, $CDCl_3$) δ -111.71. **^{31}P NMR** (162 MHz, $CDCl_3$) δ 30.84. **HRMS (ESI):** m/z Calcd for $[C_{30}H_{22}FN_2O_3P, M+H]^+$: 509.1425; Found: 509.1425. $[\alpha]_D^{25}$ = +17.05 (c = 1.8, $CHCl_3$). **HPLC:** 90% ee. (HPLC condition: Chiralpak AD column, *n*-hexane/*i*-PrOH = 65:35, 1 mL/min, 220 nm, major enantiomer t_r = 7.8 min, minor enantiomer t_r = 9.1 min.)

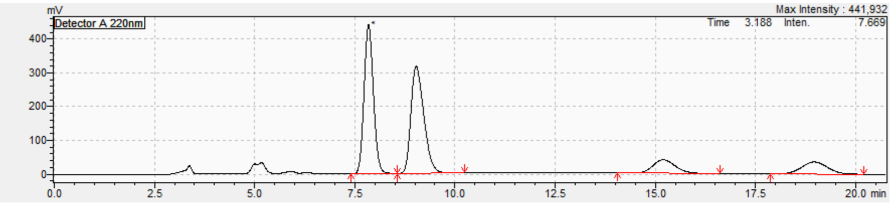

Results View - Peak Table

Peak Table | Compound | Group | Calibration Curve

Peak#	Ret. Time	Area	Height	Mark	Conc.	Unit	ID#	Name	Area%
1	7.845	7169464	440760		40.886				40.886
2	9.030	7176038	317211	V	40.923				40.923
3	15.195	1593837	40204		9.089				9.089
4	18.967	1596081	35691		9.102				9.102
Total		17535420	833865		100.000				100.000

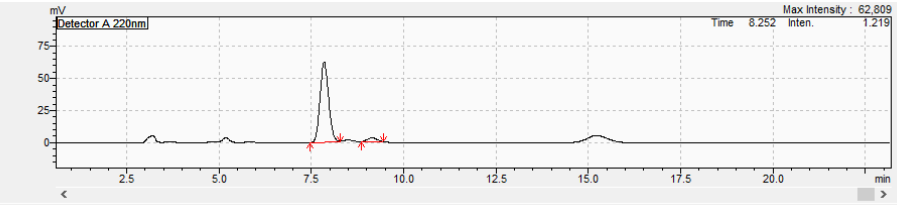

Results View - Peak Table

Peak Table | Compound | Group | Calibration Curve

Peak#	Ret. Time	Area	Height	Mark	Conc.	Unit	ID#	Name	Area%
1	7.845	1016838	62588	M	94.932				94.932
2	9.145	54280	2903	M	5.068				5.068
Total		1071118	65491		100.000				100.000

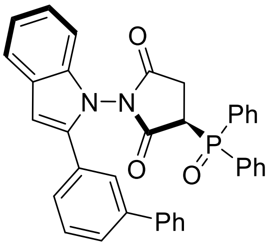

(*P*,*R*)-1-(2-([1,1'-Biphenyl]-3-yl)-1H-indol-1-yl)-3-(diphenylphosphoryl)-pyrrolidine-2,5-dione (3g)

Prepared according to the general procedure A in 81% yield as a colorless oil. **^{1}H NMR** (500 MHz, $CDCl_3$) δ 7.98-7.89 (m, 2H), 7.86-7.79 (m, 2H), 7.70-7.44 (m, 15H), 7.42-7.35 (m, 2H), 7.23 (td, *J* = 7.0, 1.4 Hz, 2H), 7.01 (t, *J* = 6.8 Hz, 1H), 6.79 (d, *J* = 8.7 Hz, 1H), 3.91 (m, 1H), 3.32 (m, 1H), 2.95 (m, 1H). **^{13}C NMR** (125 MHz, $CDCl_3$) δ 171.1, 169.5, 141.7, 140.8, 140.2, 137.5, 133.1, 131.9 (d, *J* = 9.9 Hz), 131.3, 131.0, 130.4, 129.4 (d, *J* = 4.3 Hz), 129.3, 129.1, 129.0, 128.6 (d, *J* = 12.0 Hz), 127.8, 127.61 (d, *J* = 12.6 Hz), 127.3, 127.1, 127.0, 127.0, 126.9, 124.2, 122.4, 121.1, 109.6, 104.2, 41.0 (d, *J* = 59.8 Hz), 28.3. **^{31}P NMR:** (202 MHz, $CDCl_3$) δ 30.64. **HRMS (ESI):** m/z Calcd for $[C_{36}H_{27}N_2O_3P, M+H]^+$: 567.1832; Found: 567.1831.

$[\alpha]_D^{25}$ = +4.04 (c = 2.4, $CHCl_3$). **HPLC:** 90% ee. (HPLC condition: Chiralpak AD column, *n*-hexane/*i*-PrOH = 70:30, 1 mL/min, 220 nm, major enantiomer t_r = 13.2 min, minor enantiomer t_r = 11.6 min.)

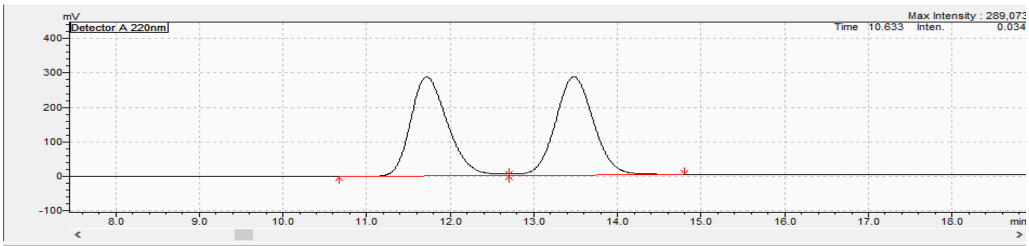

Results View - Peak Table

Peak Table | Compound | Group | Calibration Curve

Peak#	Ret. Time	Area	Height	Mark	Conc.	Unit	ID#	Name	Area%
1	11.718	8771044	286864	M	49.350				49.350
2	13.480	9002184	285202	V M	50.650				50.650
Total		17773228	572066		100.000				100.000

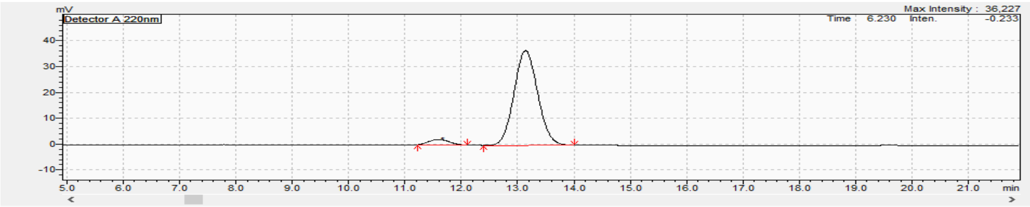

Results View - Peak Table

Peak Table | Compound | Group | Calibration Curve

Peak#	Ret. Time	Area	Height	Mark	Conc.	Unit	ID#	Name	Area%
1	11.597	52358	2086	M	4.664				4.664
2	13.141	1070287	36692	M	95.336				95.336
Total		1122644	38778		100.000				100.000

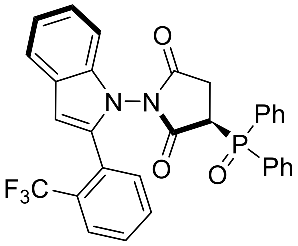

(*P*,*R*)-3-(Diphenylphosphoryl)-1-(2-(2-(trifluoromethyl)phenyl)-1H-indol-1-yl)pyrrolidine-2,5-dione (3h)

Prepared according to the general procedure A in 90% yield as a colorless oil. **^{1}H NMR** (500 MHz, $CDCl_3$) δ 7.97-7.86 (m, 2H), 7.83-7.76 (m, 3H), 7.62-7.54 (m, 5H), 7.50-7.44 (m, 5H), 7.24-7.16 (m, 2H), 7.03 (t, *J* = 7.1 Hz, 1H), 6.76 (d, *J* = 5.4 Hz, 1H), 3.89 (m, 1H), 3.29-3.14 (m, 1H), 2.87 (m, 1H). **^{13}C NMR** (125 MHz, $CDCl_3$) δ 170.7, 136.3, 135.1, 133.1 (d, *J* = 8.6 Hz), 132.4, 132.1, 131.9, 131.8, 131.6 (d, *J* = 10.0 Hz), 131.3, 131.2, 131.1 (d, *J* = 9.5 Hz), 130.5, 130.2, 129.2 (q, *J* = 37.3, 13.2 Hz), 128.6, 126.9 (d, *J* = 5.1 Hz), 126.2, 124.5, 123.6 (q, *J* = 274.0 Hz), 122.1, 121.3, 109.3, 108.3, 106.6, 40.9 (d, *J* = 59.6 Hz), 28.3. **^{31}P NMR:** (162 MHz, $CDCl_3$) δ 30.96. **^{19}F NMR** (377 MHz, $CDCl_3$) δ -57.31. **HRMS (ESI):** m/z Calcd for $[C_{31}H_{22}F_3N_2O_3P, M+H]^+$: 559.1393; Found: 559.1393. $[\alpha]_D^{25}$ = +25.02 (c = 2.33, $CHCl_3$). **HPLC:** 93% ee. (HPLC condition: Chiralpak AD column, *n*-hexane/*i*-PrOH = 70:30, 1 mL/min, 220 nm, major enantiomer t_r = 18.9 min, minor enantiomer t_r = 10.7 min.)

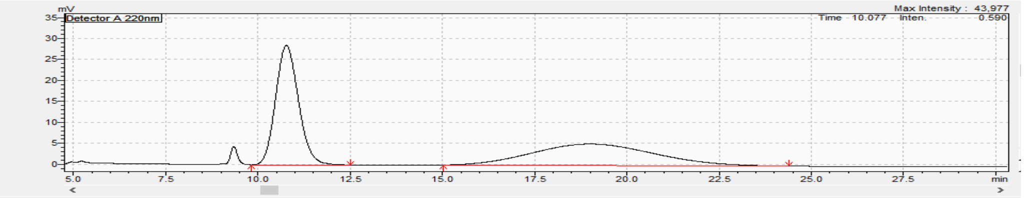

Results View - Peak Table

Peak Table | Compound | Group | Calibration Curve

Peak#	Ret. Time	Area	Height	Mark	Conc.	Unit	ID#	Name	Area%
1	10.770	1229397	28526		51.041				51.041
2	19.017	1179256	5106		48.959				48.959
Total		2408653	33633		100.000				100.000

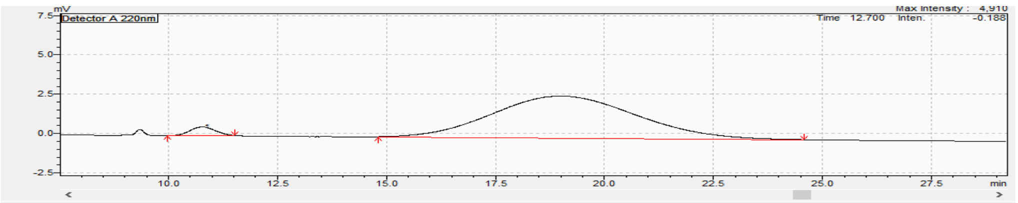

Results View - Peak Table

Peak Table | Compound | Group | Calibration Curve

Peak#	Ret. Time	Area	Height	Mark	Conc.	Unit	ID#	Name	Area%
1	10.777	22353	553	M	3.529				3.529
2	18.960	611113	2663	M	96.471				96.471
Total		633466	3216		100.000				100.000

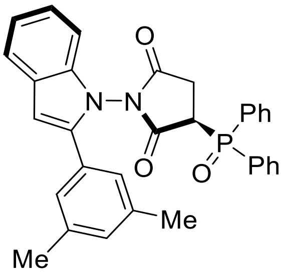

(*P*,*R*)-1-(2-(3,5-Dimethylphenyl)-1H-indol-1-yl)-3-(diphenylphosphoryl)-pyrrolidine-2,5-dione (3i)

Prepared according to the general procedure A in 83% yield as a colorless oil. **^{1}H NMR** (500 MHz, $CDCl_3$) δ 7.89-7.80 (m, 2H), 7.73-7.66 (m, 2H), 7.54-7.44 (m, 5H), 7.38 (td, *J* = 7.7, 3.3 Hz, 2H), 7.08 (m, 2H), 6.95 (m, 2H), 6.90 (s, 1H), 6.84-6.76 (m, 1H), 6.57 (s, 1H), 3.82 (m, 1H), 3.23 (m, 1H), 2.93-2.82 (m, 1H), 2.22 (s, 6H). **^{13}C NMR** (125 MHz, $CDCl_3$) δ 171.1 (d, *J* = 3.0 Hz), 169.4 (d, *J* = 4.3 Hz), 141.1, 138.4, 137.4, 133.1 (d, *J* = 3.1 Hz), 131.9 (d, *J* = 10.0 Hz), 131.3 (d, *J* = 9.7 Hz), 130.5, 130.3, 129.4, 129.3, 129.1, 129.0, 128.5, 127.1, 125.9, 123.9, 122.2, 121.0, 109.5, 103.7, 41.0 (d, *J* = 59.8 Hz), 28.4, 21.5 (d, *J* = 6.7 Hz). **^{31}P NMR:** (162 MHz, $CDCl_3$) δ 30.61. **HRMS (ESI):** m/z Calcd for $[C_{32}H_{27}N_2O_3P, M+H]^+$: 519.1832; Found: 519.1833. $[\alpha]_D^{25}$ = +25 (c = 1.8, $CHCl_3$). **HPLC:** 90% ee. (HPLC condition: Chiralpak AD-H column, *n*-hexane/*i*-PrOH = 70:30, 0.8 mL/min, 220 nm, major enantiomer t_r = 10.96 min, minor enantiomer t_r = 10.06 min.)

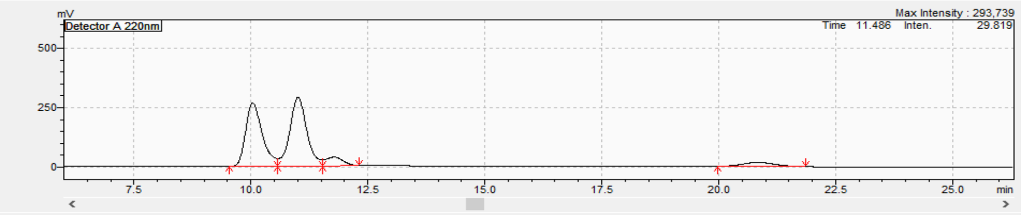

Results View - Peak Table

Peak Table | Compound | Group | Calibration Curve

Peak#	Ret. Time	Area	Height	Mark	Conc.	Unit	ID#	Name	Area%
1	10.038	6703543	268528	M	42.422				42.422
2	11.008	7346232	290735	V M	46.489				46.489
3	11.780	951143	36566	V M	6.019				6.019
4	20.834	801262	16651	M	5.071				5.071
Total		15802180	612481		100.000				100.000

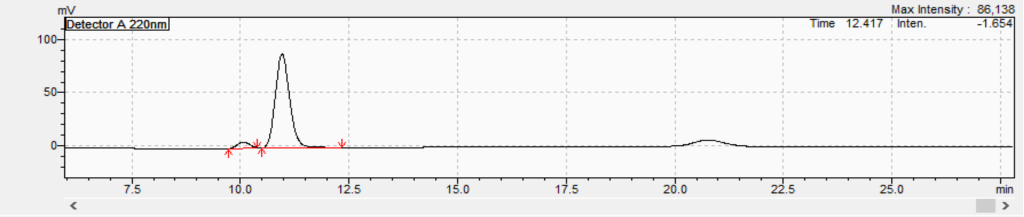

Results View - Peak Table

Peak Table | Compound | Group | Calibration Curve

Peak#	Ret. Time	Area	Height	Mark	Conc.	Unit	ID#	Name	Area%
1	10.069	112530	5636	M	5.216				5.216
2	10.960	2044943	88012	M	94.784				94.784
Total		2157472	93648		100.000				100.000

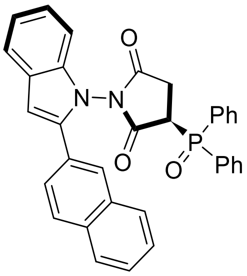

(*P*,*R*)-3-(Diphenylphosphoryl)-1-(2-(naphthalen-2-yl)-1H-indol-1-

yl)pyrrolidine-2,5-dione (3j)

Prepared according to the general procedure A in 99% yield as a coloress oil. **^{1}H NMR** (500 MHz, $CDCl_3$) δ 7.85 (m, 8H), 7.60-7.45 (m, 11H), 7.33-7.23 (m, 3H), 7.10 (d, *J* = 6.8 Hz, 3H), 6.82 (s, 3H), 3.81 (m, 1H), 3.26 (t, *J* = 17.8 Hz, 1H), 2.86 (m, 1H).**^{13}C NMR** (125 MHz, $CDCl_3$) δ 171.2, 169.4 (d, J = 4.5 Hz), 140.9, 137.7, 133.3, 133.1, 133.0, 131.8 (d, *J* = 9.9 Hz), 131.2 (d, *J* = 9.6 Hz), 130.3, 129.3 (d, *J* = 12.4 Hz), 129.0 (d, *J* = 12.5 Hz), 128.6, 128.4, 127.9, 127.8, 127.5, 127.2, 126.8, 126.7, 125.8, 125.2, 124.2, 123.7, 122.4, 121.1, 109.7, 104.5, 40.8 (d, *J* = 59.7 Hz), 28.3. **^{31}P NMR:** (162 MHz, $CDCl_3$) δ 30.77. **HRMS (ESI):** m/z Calcd for $[C_{34}H_{25}N_2O_3P, M+H]^+$: 541.1676; Found: 541.1678. $[\alpha]_D^{25}$ = 5.5 (c = 2.2, $CHCl_3$). **HPLC:** 93.5% ee. (HPLC condition: Chiralpak AD column, *n*-hexane/*i*-PrOH = 75:25, 1 mL/min, 220 nm, major enantiomer t_r = 13.1 min, minor enantiomer t_r = 17.1 min.)

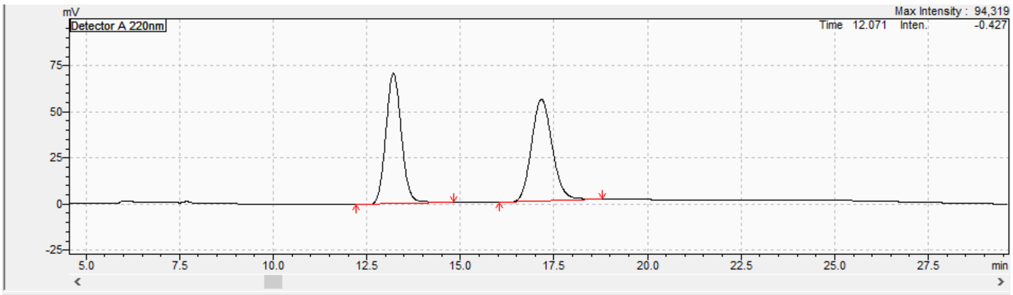

Results View - Peak Table

Peak Table | Compound | Group | Calibration Curve

Peak#	Ret. Time	Area	Height	Mark	Conc.	Unit	ID#	Name	Area%
1	13.213	2041032	70799	M	48.915				48.915
2	17.166	2131617	55292	M	51.085				51.085
Total		4172649	126091		100.000				100.000

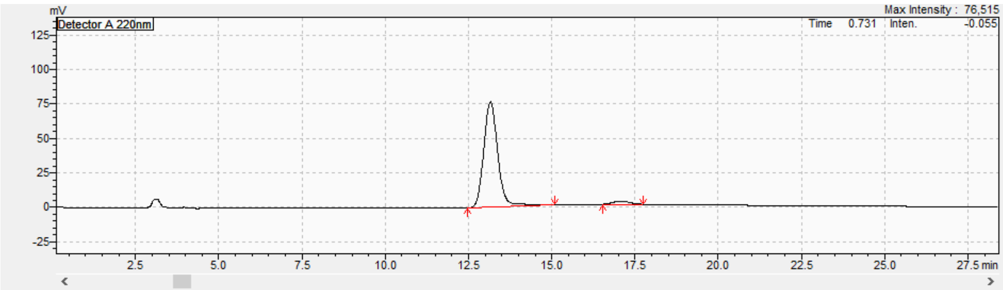

Results View - Peak Table

Peak Table | Compound | Group | Calibration Curve

Peak#	Ret. Time	Area	Height	Mark	Conc.	Unit	ID#	Name	Area%
1	13.160	2242125	76351		96.559				96.559
2	17.077	79903	2280	M	3.441				3.441
Total		2322029	78631		100.000				100.000

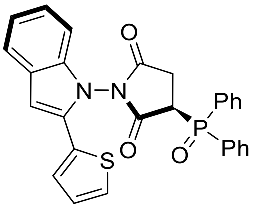

(*P*,*R*)-3-(Diphenylphosphoryl)-1-(2-(thiophen-2-yl)-1H-indol-1-yl)pyrrolidine-2,5-dione (3k)

Prepared according to the general procedure A in 81% yield as a colorless oil. **^{1}H NMR** (500 MHz, $CDCl_3$) δ 8.02 – 7.93 (m, 2H), 7.82 (m, 2H), 7.70 (m, 1H), 7.66-7.44 (m, 8H), 7.30 (dd, *J* = 5.1, 1.0 Hz, 1H), 7.22-7.10 (m, 3H), 7.04 (m, 1H), 6.94-6.86 (m, 1H), 6.81 (s, 1H), 4.07 (m, 1H), 3.37 (m, 1H), 3.25-3.03 (m, 1H). **^{13}C NMR** (125 MHz, $CDCl_3$) δ 170.9, 169.4, 137.0, 133.1, 133.1, 132.7, 131.9 (d, *J* = 9.9 Hz), 131.3 (d, *J* = 9.7 Hz), 130.8 (d, *J* = 11.5 Hz), 130.4, 129.4 (d, *J* = 12.5 Hz), 129.1 (d, *J* = 12.5 Hz), 128.7, 127.9, 126.6, 126.5, 124.4, 122.4, 121.1, 109.2, 104.0, 41.1 (d, *J* = 59.5 Hz), 28.5. **^{31}P NMR:** (162 MHz, $CDCl_3$) δ 30.60. **HRMS (ESI):** m/z Calcd for $[C_{28}H_{21}N_2O_3PS, M + H]^+$: 497.1083; Found: 497.1082. $[\alpha]_D^{25}$ = +24,2 (c = 1.5, $CHCl_3$). **HPLC:** 92 % ee. (HPLC condition: Chiralpak AD column, n-hexane/i-PrOH = 70:30, 1 mL/min, 220 nm, major enantiomer t_r = 14.7 min, minor enantiomer t_r = 9.9 min.)

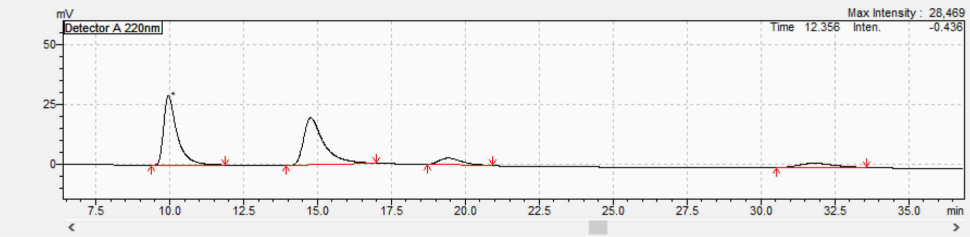

Results View - Peak Table

Peak Table | Compound | Group | Calibration Curve

Peak#	Ret. Time	Area	Height	Mark	Conc.	Unit	ID#	Name	Area%
1	9.961	908965	28844	M	42.400				42.400
2	14.776	938264	19620	M	43.767				43.767
3	19.421	148315	2758	M	6.918				6.918
4	31.782	148227	1788	M	6.914				6.914
Total		2143771	53010		100.000				100.000

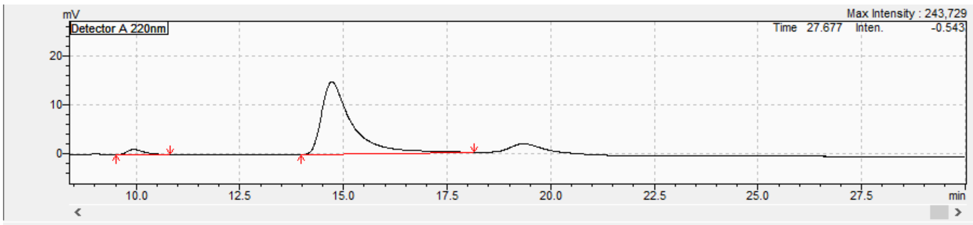

Results View - Peak Table

Peak Table | Compound | Group | Calibration Curve

Peak#	Ret. Time	Area	Height	Mark	Conc.	Unit	ID#	Name	Area%
1	9.935	31076	1085	M	3.927				3.927
2	14.716	760180	14823	M	96.073				96.073
Total		791256	15908		100.000				100.000

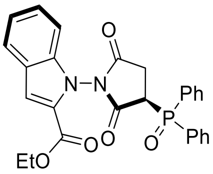

Ethyl (*P*,*R*)-1-(3-(diphenylphosphoryl)-2,5-dioxopyrrolidin-1-yl)-1H-indole-

2-Carboxylate (3l)

Prepared according to the general procedure A in 84% yield as a colorless oil.

^{1}H NMR (500 MHz, $CDCl_3$) δ 8.00 (m, 2H), 7.91-7.83 (m, 2H), 7.67-7.55 (m, 5H), 7.50 (dm, 2H), 7.38 (d, *J* = 0.8 Hz, 1H), 7.34-7.27 (m, 1H), 7.23-7.17 (m, 1H), 6.96-6.75 (m, 1H), 4.30-4.13 (m, 2H), 3.42 (m, 1H), 3.36-3.10 (m, 1H), 1.33 (m, 3H). **^{13}C NMR** (125 MHz, $CDCl_3$) δ 171.1, 169.6, 160.5, 138.0, 133.1, 133.0, 132.0 (d, *J* = 9.9 Hz), 131.4 (d, *J* = 9.8 Hz), 130.7, 129.8, 129.3 (d, *J* = 12.4 Hz), 129.1 (d, *J* = 12.5 Hz), 127.2, 125.2, 124.4, 123.0, 122.7, 111.9, 109.5, 61.09, 41.2 (d, *J* = 60.4 Hz), 28.6, 14.3. **^{31}P NMR:** (162 MHz, $CDCl_3$) δ 30.25. **HRMS (ESI):** m/z Calcd for [$C_{27}H_{23}N_2O_5P$, M+H]$^+$: 487.1417; Found: 487.1416. $[\alpha]_D^{25}$ = +191 (c = 1.10, $CHCl_3$). **HPLC:** 82% ee. (HPLC condition: Chiralpak AD column, *n*-hexane/*i*-PrOH = 85:15, 1 mL/min, 220 nm, major enantiomer t_r = 28.6 min, minor enantiomer t_r = 21.0 min.)

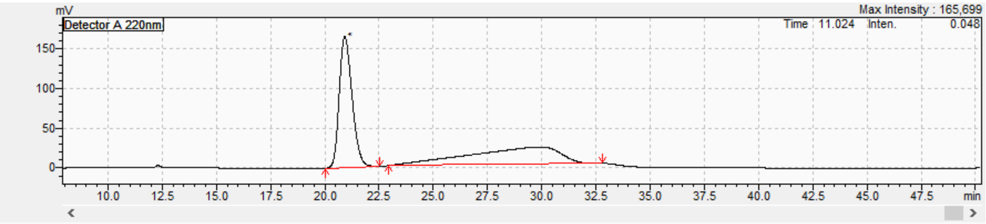

Results View - Peak Table

Peak Table | Compound | Group | Calibration Curve

Peak#	Ret. Time	Area	Height	Mark	Conc.	Unit	ID#	Name	Area%
1	20.917	6793388	165073		51.997				51.997
2	29.915	6271619	21622	M	48.003				48.003
Total		13065008	186696		100.000				100.000

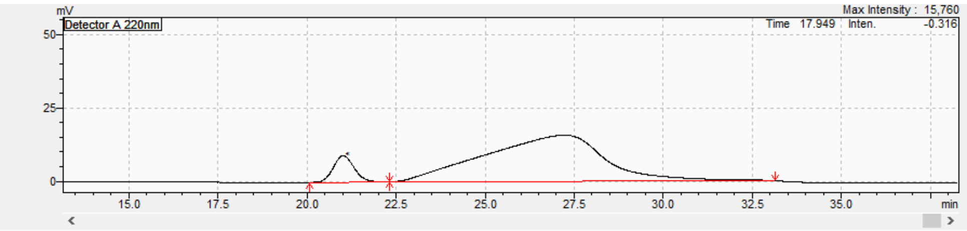

Results View - Peak Table

Peak Table | Compound | Group | Calibration Curve

Peak#	Ret. Time	Area	Height	Mark	Conc.	Unit	ID#	Name	Area%
1	21.017	365070	9044	M	8.994				8.994
2	27.229	3693921	15723	M	91.006				91.006
Total		4058991	24767		100.000				100.000

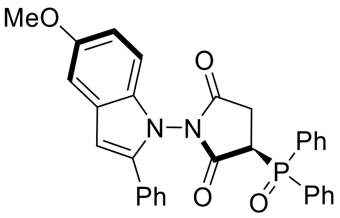

(*P*,*R*)-3-(Diphenylphosphoryl)-1-(5-methoxy-2-phenyl-1H-indol-1-yl)pyrrolidine-2,5-dione (3m)

Prepared according to the general procedure A in 86% yield as a colorless oil. **^{1}H NMR** (500 MHz, $CDCl_3$) δ 7.97-7.86 (m, 2H), 7.83-7.77 (m, 2H), 7.66-7.50 (m, 5H), 7.47 (m, 2H), 7.44-7.31 (m, 6H), 7.05 (dd, *J* = 9.0, 2.0 Hz, 1H), 6.88-6.81 (m, 2H), 6.65-6.60 (m, 1H), 3.94-3.86 (m, 1H), 3.82 (d, *J* = 6.2 Hz, 3H), 3.31-3.20 (m, 1H), 2.92 (m, 1H). **^{13}C NMR** (125 MHz, $CDCl_3$) δ 171.13, 169.54, 156.00, 141.74, 133.07 (d, *J* = 4.6 Hz), 132.65, 131.88 (d, *J* = 9.9 Hz), 131.28 (d, *J* = 9.7 Hz), 130.52, 130.36, 129.52, 129.33 (d, *J* = 12.4 Hz), 129.15, 129.07 (d, *J* = 12.6 Hz), 128.90, 128.67, 128.07, 127.82 (d, *J* = 4.0 Hz), 113.78, 110.50, 103.97, 103.30, 55.96, 40.94 (d, *J* = 59.6 Hz), 28.31. **^{31}P NMR:** (162 MHz, $CDCl_3$) δ 30.62. **HRMS (ESI):** m/z Calcd for $[C_{31}H_{26}N_2O_4P, M+H]^+$: 521.1625; Found: 521.1623. $[\alpha]_D^{25}$ = +21.35 (c = 2.0, $CHCl_3$). **HPLC:** 92% ee. (HPLC condition: Chiralpak AD column, *n*-hexane/*i*-PrOH = 70:30, 1 mL/min, 220 nm, major enantiomer t_r = 12.2 min, minor enantiomer t_r = 18.9 min.)

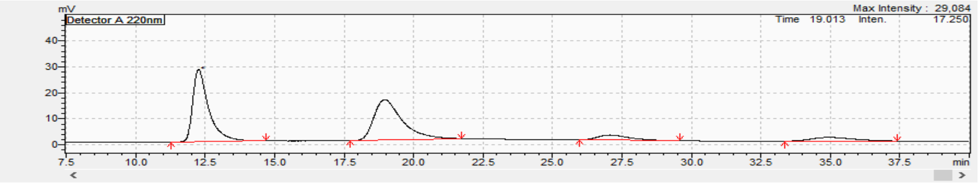

Results View - Peak Table

Peak Table | Compound | Group | Calibration Curve

Peak#	Ret. Time	Area	Height	Mark	Conc.	Unit	ID#	Name	Area%
1	12.263	1113899	27889		43.990				43.990
2	18.958	1113852	15571	M	43.988				43.988
3	27.069	153498	1940		6.062				6.062
4	34.964	150944	1426	M	5.961				5.961
Total		2532193	46827		100.000				100.000

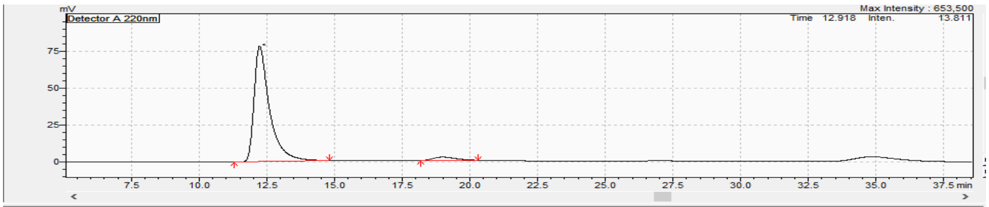

Results View - Peak Table

Peak Table | Compound | Group | Calibration Curve

Peak#	Ret. Time	Area	Height	Mark	Conc.	Unit	ID#	Name	Area%
1	12.235	3109015	78250	M	95.774				95.774
2	18.992	137199	2322	M	4.226				4.226
Total		3246214	80572		100.000				100.000

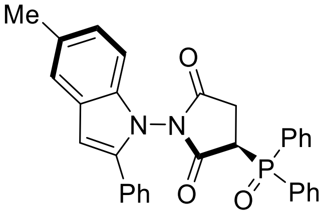

(*P*,*R*)-3-(Diphenylphosphoryl)-1-(5-methyl-2-phenyl-1H-indol-1-yl)pyrrolidine-2,5-dione (3n)

Prepared according to the general procedure A in 89% yield as a colorless oil. **^{1}H**

NMR (400 MHz, $CDCl_3$) δ 7.83 (m, 2H), 7.74-7.66 (m, 2H), 7.52-7.43 (m, 4H), 7.39 (dd, *J* = 7.8, 3.4 Hz, 2H), 7.28 (m, 6H), 6.90 (dd, *J* = 8.3, 1.1 Hz, 1H), 6.67 (d, *J* = 8.3 Hz, 1H), 6.52 (d, *J* = 0.8 Hz, 1H), 3.80 (m, 1H), 3.18 (m, 1H), 2.84 (m, 1H), 2.32 (s, 3H). **^{13}C NMR** (100 MHz, $CDCl_3$) δ 171.1, 169.5, 141.0, 135.9, 133.1 (d, *J* = 4.2 Hz), 132.0 (d, *J* = 9.9 Hz), 131.7, 131.4 (d, *J* = 9.7 Hz), 130.6, 129.4, 129.3, 129.2 (d, *J* = 2.2 Hz), 129.0, 128.9, 128.6, 128.1, 127.8, 127.4, 125.5, 120.9, 109.2, 103.8, 41.0 (d, *J* = 60.0 Hz), 28.3, 21.6. **^{31}P NMR:** (162 MHz, $CDCl_3$) δ 30.50. **HRMS (ESI):** m/z Calcd for $[C_{31}H_{25}N_2O_3P, M+H]^+$: 505.1676; Found: 505.1674. $[\alpha]_D^{25}$ = +23.13 (c =1.50, $CHCl_3$). **HPLC:** 93% ee. (HPLC condition: Chiralpak AD column, *n*-hexane/*i*-PrOH = 80:20, 0.8 mL/min, 220 nm, major enantiomer t_r = 42.3 min, minor enantiomer t_r = 45.1 min.)

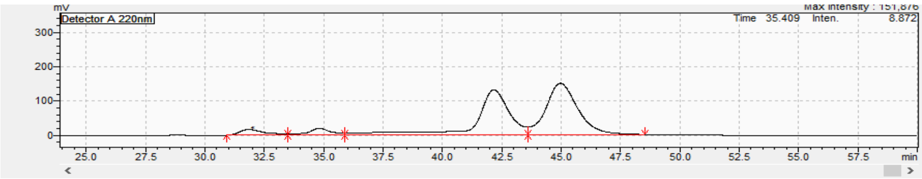

Results View - Peak Table

Peak Table | Compound | Group | Calibration Curve

Peak#	Ret. Time	Area	Height	Mark	Conc.	Unit	ID#	Name	Area%
1	31.848	1252104	16514	M	4.289				4.289
2	34.818	1363988	19931	V M	4.672				4.672
3	42.166	12601570	130752	V M	43.163				43.163
4	44.967	13977549	149813	V M	47.876				47.876
Total		29195211	317010		100.000				100.000

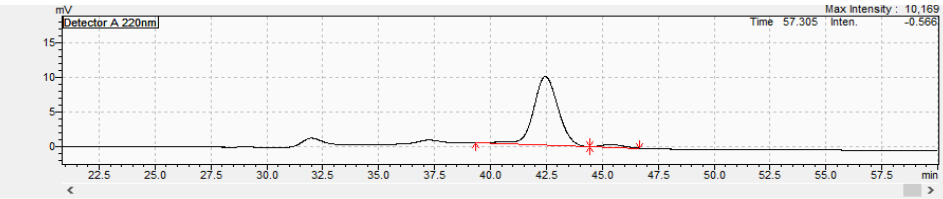

Results View - Peak Table

Peak Table | Compound | Group | Calibration Curve

Peak#	Ret. Time	Area	Height	Mark	Conc.	Unit	ID#	Name	Area%
1	42.456	761546	9997	M	96.573				96.573
2	45.397	27024	396	M	3.427				3.427
Total		788570	10393		100.000				100.000

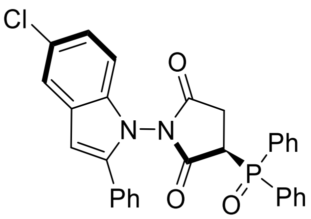

(*P*,*R*)-1-(5-Chloro-2-phenyl-1H-indol-1-yl)-3-(diphenylphosphoryl)pyrrolidine-2,5-dione (3o)

Prepared according to the general procedure A in 99% yield as a colorless oil. **^{1}H NMR** (500 MHz, $CDCl_3$) δ 7.86-7.80 (m, 2H), 7.75-7.69 (m, 2H), 7.58-7.35 (m, 5H),

7.27 (s, 2H), 7.18 (d, *J* = 2.2 Hz, 2H), 7.16-7.09 (m, 3H), 7.00-6.93 (m, 1H), 6.61 (d, *J* = 0.8 Hz, 1H), 3.86 (m, 1H), 3.19 (m, 1H), 2.92-2.81 (m, 1H). **^{13}C NMR** (125 MHz, $CDCl_3$) δ 171.02 (d, *J* = 2.9 Hz), 169.46 (d, *J* = 4.7 Hz), 139.58, 137.54, 134.82, 133.16 (d, *J* = 2.7 Hz), 133.08 (d, *J* = 2.8 Hz), 131.84 (d, *J* = 9.9 Hz), 131.22 (d, *J* = 9.6 Hz), 129.50, 129.45, 129.32, 129.17, 129.11, 128.99, 128.96, 126.96, 124.42, 122.51, 121.16, 109.67, 40.93 (d, *J* = 59.5 Hz), 28.35. **^{31}P NMR:** (162 MHz, $CDCl_3$) δ 30.79. **HRMS (ESI):** m/z Calcd for $[C_{30}H_{23}ClN_2O_3P, M+H]^+$: 525.1129; Found: 525.1130. $[\alpha]_D^{25}$ = +6.38 (c = 1.6, $CHCl_3$). **HPLC:** 85% ee. (HPLC condition: Chiralpak AD column, *n*-hexane/*i*-PrOH = 70:30, 1 mL/min, 220 nm, major enantiomer t_r = 19.5 min, minor enantiomer t_r = 2 min.)

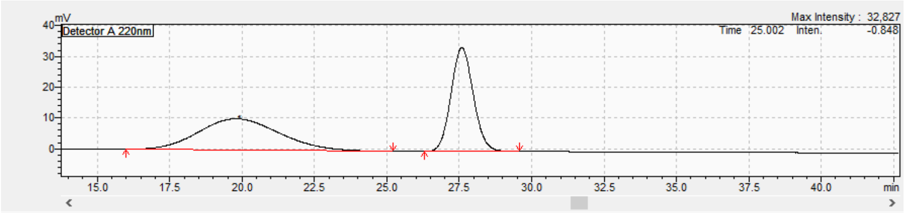

Results View - Peak Table

Peak Table | Compound | Group | Calibration Curve

Peak#	Ret. Time	Area	Height	Mark	Conc.	Unit	ID#	Name	Area%
1	19.769	1998871	10185		52.467				52.467
2	27.587	1810872	33735		47.533				47.533
Total		3809743	43919		100.000				100.000

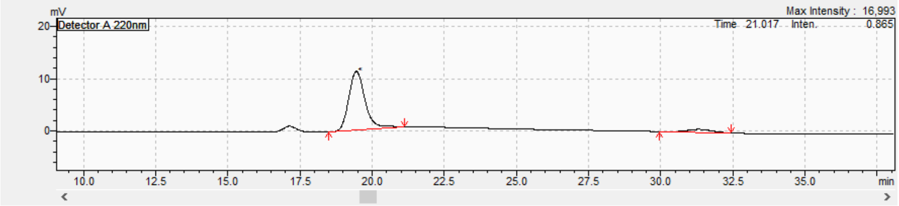

Results View - Peak Table

Peak Table | Compound | Group | Calibration Curve

Peak#	Ret. Time	Area	Height	Mark	Conc.	Unit	ID#	Name	Area%
1	19.454	440199	11272	M	92.500				92.500
2	31.304	35690	585	M	7.500				7.500
Total		475889	11856		100.000				100.000

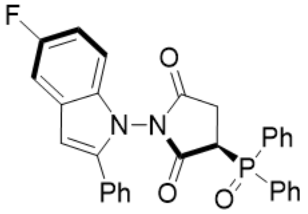

(*P*,*R*)-3-(Diphenylphosphoryl)-1-(5-fluoro-2-phenyl-1H-indol-1-yl)pyrrolidine-2,5-dione (3p)

Prepared according to the general procedure A in 78% yield as a white solid. **^{1}H NMR** (500 MHz, $CDCl_3$) δ 7.95-7.85 (m, 2H), 7.82-7.76 (m, 2H), 7.63-7.53 (m, 4H),

7.47 (m, 2H), 7.42-7.33 (m, 5H), 7.23 (dd, *J* = 9.1, 2.4 Hz, 1H), 7.04 (dd, *J* = 8.8, 4.3 Hz, 1H), 6.96 (m, 1H), 6.65 (s, 1H), 3.92 (m, 1H), 3.24 (m, 1H), 2.92 (m, 1H). **^{13}C NMR** (125 MHz, $CDCl_3$) δ 171.0, 169.4 (d, *J* = 4.8 Hz), 160.3, 158.5, 142.5, 133.9, 133.2 (d, *J* = 2.7 Hz), 133.1 (d, *J* = 2.7 Hz), 131.8 (d, *J* = 10.0 Hz), 131.2 (d, *J* = 9.7 Hz), 130.13, 129.4 (d, *J* = 12.5 Hz), 129.1, 129.0 (d, *J* = 2.6 Hz), 129.0, 128.6, 128.18, 127.7 (d, *J* = 10.5 Hz), 112.2 (d, *J* = 26.3 Hz), 110.6 (d, *J* = 9.6 Hz), 106.4 (d, *J* = 24.3 Hz), 103.8 (d, *J* = 4.1 Hz), 40.9 (d, *J* = 59.2 Hz), 28.4. **^{31}P NMR:** (162 MHz, $CDCl_3$) δ 31.08. **^{19}F NMR** (471 MHz, $CDCl_3$) δ -121.83. **HRMS (ESI):** m/z Calcd for [$C_{30}H_{22}FN_2O_3P$, M+H]$^+$: 509.1425; Found: 509.1425. $[\alpha]_D^{25}$ = +10.0 (c = 1.1, $CHCl_3$). **HPLC:** 93% ee. (HPLC condition: Chiralpak AD column, *n*-hexane/*i*-PrOH = 70:30, 1 mL/min, 220 nm, major enantiomer t_r = 10.2 min, minor enantiomer t_r = 13.0 min.)

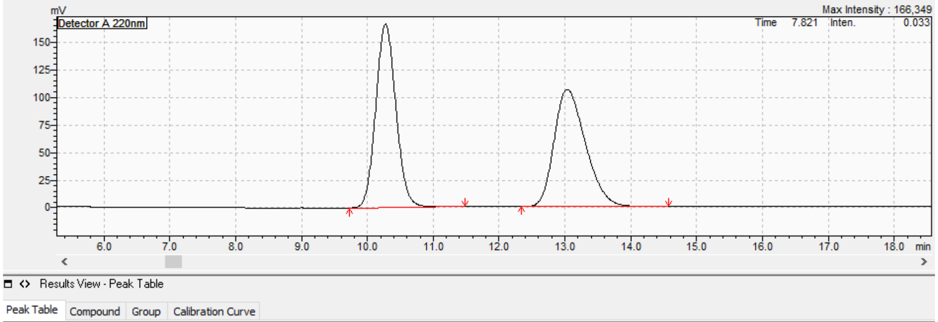

Peak#	Ret. Time	Area	Height	Mark	Conc.	Unit	ID#	Name	Area%
1	10.275	3538310	166086		50.143				50.143
2	13.034	3518117	106168		49.857				49.857
Total		7056427	272254		100.000				100.000

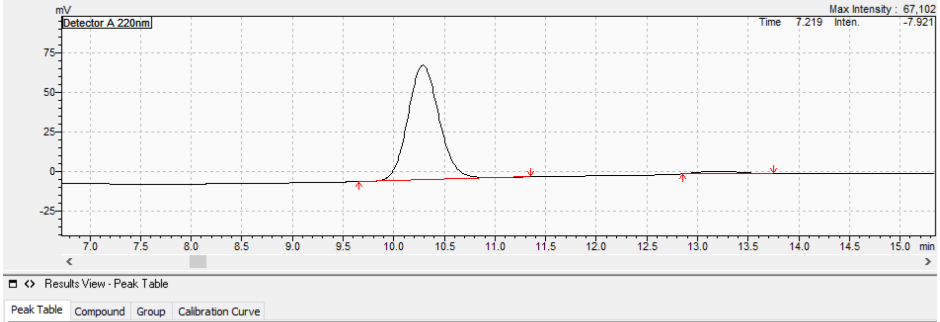

Peak#	Ret. Time	Area	Height	Mark	Conc.	Unit	ID#	Name	Area%
1	10.289	1555383	72471	M	97.149				97.149
2	13.202	45647	1671	M	2.851				2.851
Total		1601030	74142		100.000				100.000

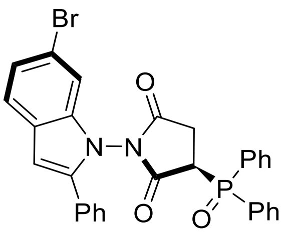

(*P*,*R*)-1-(6-Bromo-2-phenyl-1H-indol-1-yl)-3-(diphenylphosphoryl)pyrrolidine-2,5-dione (3q)

Prepared according to the general procedure A in 85% yield as a colorless oil. **^{1}H NMR** (500 MHz, $CDCl_3$) δ 7.88-7.77 (m, 2H), 7.75-7.66 (m, 2H), 7.58-7.39 (m, 7H), 7.37-7.27 (m, 4H), 7.23-7.13 (m, 2H), 7.09 (s, 1H), 6.58-6.51 (m, 1H), 3.85-3.73 (m, 1H), 3.25-3.12 (m, 1H), 2.85 (m, 1H). **^{13}C NMR** (125 MHz, $CDCl_3$) δ 170.9 (d, *J* = 3.2 Hz), 169.4, 141.3, 137.9, 133.7 (d, *J* = 2.7 Hz), 133.2, 131.9, 131.8, 131.4, 131.3, 129.9, 129.4, 129.2 (d, *J* = 2.2 Hz), 129.1 (d, *J* = 6.9 Hz), 129.0, 128.2, 126.0, 125.8, 122.4, 117.4, 112.5, 103.8, 41.2 (d, *J* = 59.3 Hz), 28.3. **^{31}P NMR:** (162 MHz, $CDCl_3$) δ 30.16. **HRMS (ESI):** m/z Calcd for $[C_{30}H_{22}BrN_2O_3P, M+H]^+$: 569.0624; Found: 569.0625. $[\alpha]_D^{25}$ = +39.63 (c = 2.2, $CHCl_3$). **HPLC:** 80% ee. (HPLC condition: Chiralpak IE column, *n*-hexane/*i*-PrOH = 80:20, 1 mL/min, 220 nm, major enantiomer t_r = 19.6 min, minor enantiomer t_r = 17.6 min.)

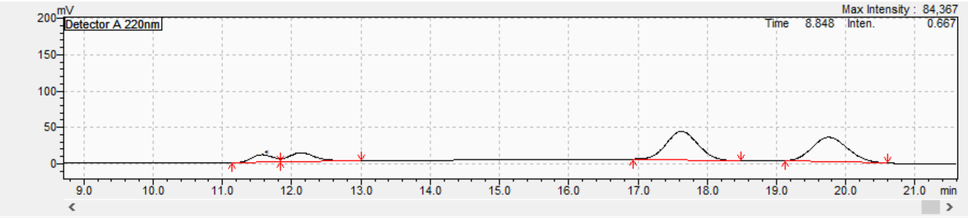

Results View - Peak Table

Peak Table | Compound | Group | Calibration Curve

Peak#	Ret. Time	Area	Height	Mark	Conc.	Unit	ID#	Name	Area%
1	11.574	240780	10666		7.913				7.913
2	12.134	334337	12432	V	10.988				10.988
3	17.627	1247731	39294	M	41.006				41.006
4	19.753	1219936	33829	M	40.093				40.093
Total		3042784	96221		100.000				100.000

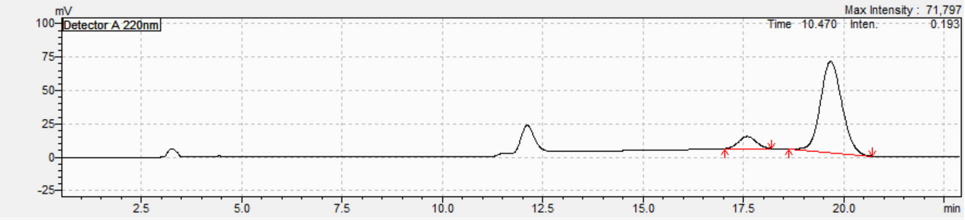

Results View - Peak Table

Peak Table | Compound | Group | Calibration Curve

Peak#	Ret. Time	Area	Height	Mark	Conc.	Unit	ID#	Name	Area%
1	17.598	278155	9064	M	9.900				9.900
2	19.673	2531529	68242	M	90.100				90.100
Total		2809684	77306		100.000				100.000

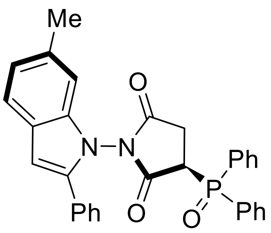

(*P*,*R*)-3-(Diphenylphosphoryl)-1-(6-methyl-2-phenyl-1H-indol-1-yl)pyrrolidine-2,5-dione (3r)

Prepared according to the general procedure A in 99% yield as a colorless oil.

^{1}H NMR (500 MHz, $CDCl_3$) δ 7.92 (dd, *J* = 11.9, 7.9 Hz, 2H), 7.82 (dd, *J* = 12.1, 7.9 Hz, 2H), 7.64-7.58 (m, 2H), 7.55 (dt, *J* = 7.1, 3.7 Hz, 2H), 7.50-7.44 (m, 3H), 7.38 (td, *J* = 16.7, 15.6, 7.6 Hz, 5H), 7.02 (d, *J* = 7.9 Hz, 1H), 6.81 (s, 1H), 6.65 (s, 1H), 3.90 (td, *J* = 12.3, 11.5, 3.2 Hz, 1H), 3.35-3.23 (m, 1H), 2.95 (dt, *J* = 19.1, 9.7 Hz, 1H), 2.46 (s, 3H). **^{13}C NMR** (125 MHz, $CDCl_3$) δ 171.1, 169.5, 140.1, 137.8, 134.1, 133.1 (d, *J* = 3.9 Hz), 132.0 (d, *J* = 9.8 Hz), 131.3 (d, *J* = 9.7 Hz), 130.6, 129.3, 129.2, 129.0, 128.9, 128.9, 128.5, 128.1, 127.8, 125.0, 124.1, 120.8, 109.4, 103.9, 40.9 (d, *J* = 60.4 Hz), 28.3, 22.1. **^{31}P NMR:** (162 MHz, $CDCl_3$) δ 30.21. **HRMS (ESI):** m/z Calcd for $[C_{31}H_{25}N_2O_3P, M+H]^+$: 505.1676; Found: 505.1675. $[\alpha]_D^{25}$ = +32.78 (c = 2.3, $CHCl_3$). **HPLC:** 90% ee. (HPLC condition: Chiralpak AD column, *n*-hexane/*i*-PrOH = 70:30, 1 mL/min, 220 nm, major enantiomer t_r = 7.3 min, minor enantiomer t_r = 14.1 min.)

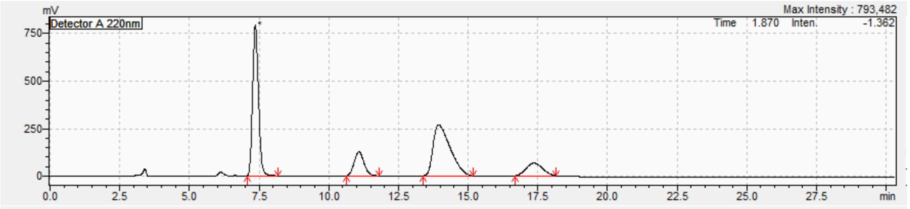

Results View - Peak Table

Peak Table | Compound | Group | Calibration Curve

Peak#	Ret. Time	Area	Height	Mark	Conc.	Unit	ID#	Name	Area%
1	7.367	11642472	791983	M	40.207				40.207
2	11.079	3137334	126569	M	10.835				10.835
3	13.944	11410616	268147	M	39.406				39.406
4	17.360	2766057	67047	M	9.552				9.552
Total		28956478	1253747		100.000				100.000

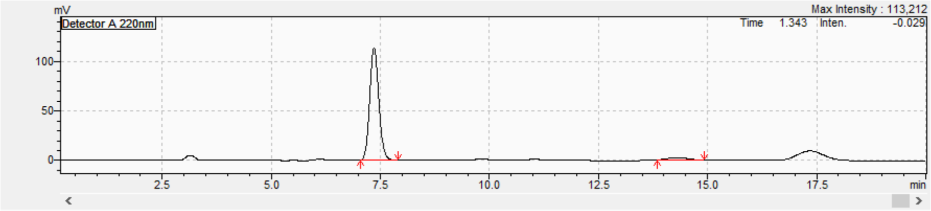

Results View - Peak Table

Peak Table | Compound | Group | Calibration Curve

Peak#	Ret. Time	Area	Height	Mark	Conc.	Unit	ID#	Name	Area%
1	7.354	1646635	112962	M	94.925				94.925
2	14.299	88039	2643	M	5.075				5.075
Total		1734674	115605		100.000				100.000

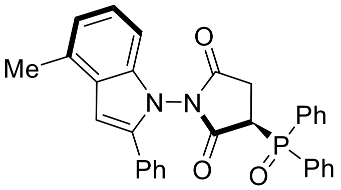

(*P*,*R*)-3-(Diphenylphosphoryl)-1-(4-methyl-2-phenyl-1H-indol-1-yl)pyrrolidine-2,5-dione (3s)

Prepared according to the general procedure A in 99% yield as a colorless oil. **^{1}H NMR** (500 MHz, $CDCl_3$) δ 7.98-7.86 (m, 2H), 7.83-7.73 (m, 2H), 7.63-7.53 (m, 4H), 7.49-7.35 (m, 7H), 7.13-7.06 (m, 1H), 7.00-6.94 (m, 1H), 6.77-6.74 (m, 1H), 6.73 (d, *J* = 0.8 Hz, 1H), 3.92 (td, *J* = 11.2, 3.4 Hz, 1H), 3.30 (ddd, *J* = 19.9, 16.8, 3.5 Hz, 1H), 2.95 (dt, *J* = 19.3, 10.1 Hz, 1H), 2.54 (s, 3H). **^{13}C NMR** (125 MHz, $CDCl_3$) δ 171.1, 169.5 (d, *J* = 4.3 Hz), 140.1, 137.1, 133.1, 131.9 (d, *J* = 9.9 Hz), 131.3 (d, *J* = 9.7 Hz), 130.6 (d, *J* = 2.6 Hz), 129.9, 129.4, 129.3, 129.1, 129.0, 128.9, 128.6, 128.2, 127.9, 126.8, 124.2, 122.7, 107.0, 102.6, 41.0 (d, *J* = 60.0 Hz), 28.3, 18.4. **^{31}P NMR:** (162 MHz, $CDCl_3$) δ 30.51. **HRMS (ESI):** m/z Calcd for $[C_{31}H_{25}N_2O_3P, M+H]^+$: 505.1676; Found: 505.1673. $[\alpha]_D^{25}$ = +19.19 (c = 2.1, $CHCl_3$). **HPLC:** 91% ee. (HPLC condition: Chiralpak AD column, *n*-hexane/*i*-PrOH = 65:35, 1 mL/min, 220 nm, major enantiomer t_r = 6.0 min, minor enantiomer t_r = 6.9 min.)

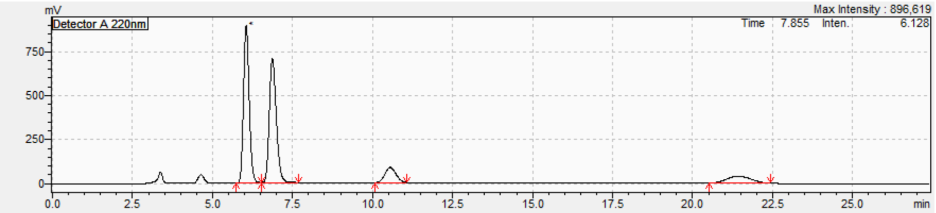

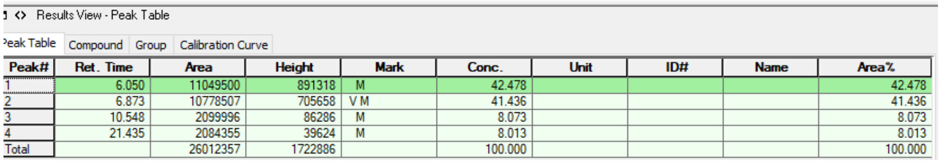

Peak#	Ret. Time	Area	Height	Mark	Conc.	Unit	ID#	Name	Area%
1	6.050	11049500	891318	M	42.478				42.478
2	6.873	10778507	705658	V M	41.436				41.436
3	10.548	2099996	86286	M	8.073				8.073
4	21.435	2084355	39624	M	8.013				8.013
Total		26012357	1722886		100.000				100.000

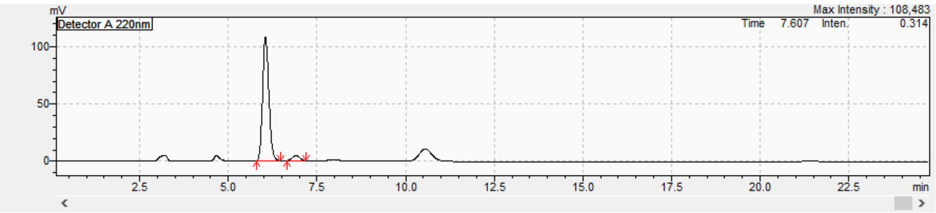

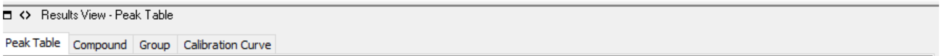

Peak#	Ret. Time	Area	Height	Mark	Conc.	Unit	ID#	Name	Area%
1	6.049	1330471	107917	M	95.444				95.444
2	6.903	63509	4390	M	4.556				4.556
Total		1393979	112307		100.000				100.000

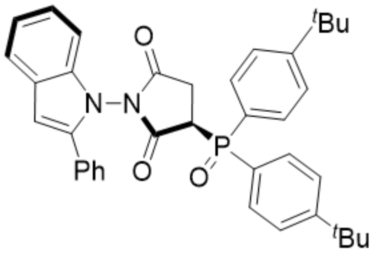

(*P*,*R*)-3-(Bis(4-(tert-butyl)phenyl)phosphoryl)-1-(2-phenyl-1H-indol-1yl)-pyrrolidine-2,5-dione (3t)

Prepared according to the general procedure A in 98% yield as a colorless oil. **^{1}H NMR** (500 MHz, $CDCl_3$) δ 7.81 (m, 4H), 7.63-7.56 (m, 3H), 7.54-7.50 (m, 2H), 7.45 (dd, *J* = 8.0, 1.5 Hz, 2H), 7.42-7.35 (m, 3H), 7.29 (d, *J* = 3.7 Hz, 2H), 7.22 (s, 1H), 6.73 (s, 1H), 3.91 (m, 1H), 3.24 (m, 1H), 2.93 (m, 1H), 1.35 (d, *J* = 9.2 Hz, 18H). **^{13}C NMR** (125 MHz, $CDCl_3$) δ 171.3, 169.6 (d, *J* = 4.7 Hz), 156.6 (d, *J* = 17.0 Hz), 140.7, 137.5, 131.8 (d, *J* = 10.3 Hz), 131.1 (d, *J* = 10.0 Hz), 130.5, 129.2, 128.9, 128.7, 128.22, 127.1, 126.3 (d, *J* = 12.6 Hz), 126.0 (d, *J* = 12.8 Hz), 125.4, 124.1, 122.3, 121.1, 109.7, 103.9, 41.0 (d, *J* = 59.6 Hz), 35.2 (d, *J* = 4.7 Hz), 31.1 (d, *J* = 1.7 Hz), 28.6. **^{31}P NMR:** (162 MHz, $CDCl_3$) δ 30.97. **HRMS (ESI):** m/z Calcd for $[C_{38}H_{39}N_2O_3P, M+H]^+$: 603.2771; Found: 603.2773. $[\alpha]_D^{25}$ = +143.52 (c = 2.4, $CHCl_3$). **HPLC:** 90% ee. (HPLC condition: Chiralpak AD column, *n*-hexane/*i*-PrOH = 70:30, 1 mL/min, 220 nm, major enantiomer t_r = 47.4 min, minor enantiomer t_r = 35.2 min.)

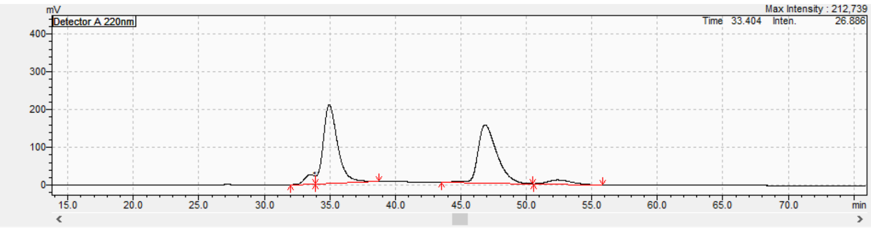

Results View - Peak Table

Peak Table | Compound | Group | Calibration Curve

Peak#	Ret. Time	Area	Height	Mark	Conc.	Unit	ID#	Name	Area%
1	33.522	1261258	24497	M	3.760				3.760
2	34.956	15815363	207677	V M	47.146				47.146
3	46.855	15085369	153094		44.970				44.970
4	52.395	1383343	11128		4.124				4.124
Total		33545334	396396		100.000				100.000

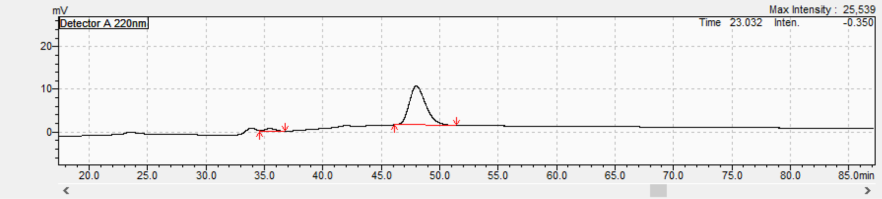

Results View - Peak Table

Peak Table | Compound | Group | Calibration Curve

Peak#	Ret. Time	Area	Height	Mark	Conc.	Unit	ID#	Name	Area%
1	35.422	46062	700	M	4.864				4.864
2	47.999	900892	9110		95.136				95.136
Total		946954	9810		100.000				100.000

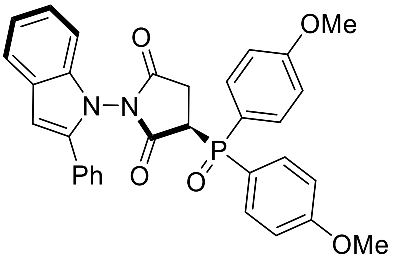

(*P*,*R*)-3-(Bis(4-methoxyphenyl)phosphoryl)-1-(2-phenyl-1H-indol-1-yl)pyrrolidine-2,5-dione (3u)

Prepared according to the general procedure A in 44% yield as a colorless oil. **^{1}H NMR** (500 MHz, $CDCl_3$) δ 7.86 (dd, *J* = 11.5, 8.7 Hz, 2H), 7.72-7.64 (m, 2H), 7.60-7.55 (m, 1H), 7.42-7.35 (m, 5H), 7.16 (m, 2H), 7.04 (dd, *J* = 8.9, 2.4 Hz, 2H), 6.96 (dd, *J* = 8.8, 2.4 Hz, 2H), 6.77 (dd, *J* = 6.1, 2.3 Hz, 1H), 6.69 (s, 1H), 3.85 (d, *J* = 9.2 Hz, 6H), 3.82 (d, *J* = 2.6 Hz, 1H), 3.33 (m, 1H), 2.94 (m, 1H). **^{13}C NMR** (125 MHz, $CDCl_3$) δ 171.3, 170.0, 163.3, 140.8, 137.4, 134.0 (d, *J* = 11.2 Hz), 133.3 (d, *J* = 11.2 Hz), 130.5, 129.1, 128.9, 128.7, 128.2, 127.9, 127.1, 123.9, 122.3, 121.1, 114.8 (d, *J* = 13.6 Hz), 114.7 (d, *J* = 13.6 Hz), 109.5, 104.0, 55.6 (d, *J* = 8.2 Hz), 41.5 (d, *J* = 59.7 Hz), 28.3. **^{31}P NMR:** (162 MHz, $CDCl_3$) δ 30.18. **HRMS (ESI):** m/z Calcd for $[C_{32}H_{27}N_2O_5P, M+H]^+$: 551.1730; Found: 551.1729. $[\alpha]_D^{25}$ = +22.4 (c = 1.0, $CHCl_3$). **HPLC:** 84% ee. (HPLC condition: Chiralpak AD column, *n*-hexane/*i*-PrOH = 70:30, 1 mL/min, 220 nm, major enantiomer t_r = 14.5 min, minor enantiomer t_r = 11.8 min.)

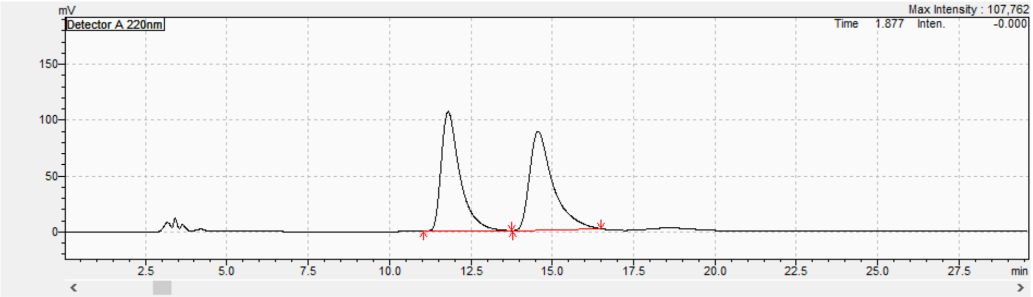

Results View - Peak Table

Peak Table | Compound | Group | Calibration Curve

Peak#	Ret. Time	Area	Height	Mark	Conc.	Unit	ID#	Name	Area%
1	11.801	4280413	107080		49.246				49.246
2	14.563	4411431	88513	M	50.754				50.754
Total		8691844	195593		100.000				100.000

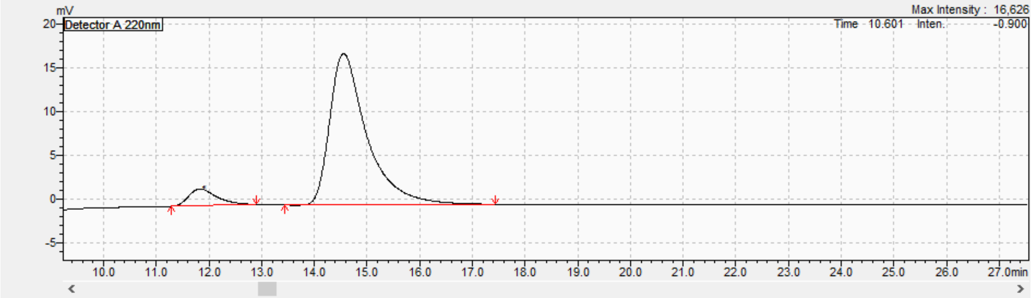

Results View - Peak Table

Peak Table | Compound | Group | Calibration Curve

Peak#	Ret. Time	Area	Height	Mark	Conc.	Unit	ID#	Name	Area%
1	11.826	71290	1918	M	7.765				7.765
2	14.555	846856	17286	M	92.235				92.235
Total		918146	19205		100.000				100.000

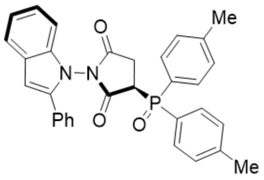

(*P*,*R*)-3-(Di-p-tolylphosphoryl)-1-(2-phenyl-1H-indol-1-yl)pyrrolidine-2,5-dione (3v)

Prepared according to the general procedure A in 58% yield as a colorless oil. **^{1}H NMR** (500 MHz, $CDCl_3$) δ 7.80 (dd, *J* = 11.9, 8.1 Hz, 2H), 7.66 (dd, *J* = 12.0, 8.1 Hz, 2H), 7.60-7.55 (m, 1H), 7.43-7.34 (m, 7H), 7.27 (d, *J* = 9.4 Hz, 2H), 7.19-7.15 (m, 2H), 6.97-6.92 (m, 1H), 6.69 (s, 1H), 3.86 (ddd, *J* = 11.3, 10.1, 3.4 Hz, 1H), 3.29 (ddd, *J* = 19.5, 16.6, 3.5 Hz, 1H), 2.92 (dt, *J* = 19.3, 10.1 Hz, 1H), 2.42 (d, *J* = 6.5 Hz, 6H). **^{13}C NMR** (125 MHz, $CDCl_3$) δ 171.1, 169.1, 143.7, 140.7, 137.3, 131.8, 131.2 (d, *J* = 10.1 Hz), 130.4, 129.9 (d, *J* = 12.8 Hz), 129.7 (d, *J* = 12.9 Hz), 129.0, 128.8, 128.6, 128.1, 127.0, 126.3, 125.4, 123.8, 122.2, 121.0, 109.5, 103.9, 41.1 (d, *J* = 59.4 Hz), 28.3, 21.7 (d, *J* = 7.3 Hz). **^{31}P NMR:** (162 MHz, $CDCl_3$) δ 30.85. **HRMS (ESI):** m/z Calcd for $[C_{32}H_{27}N_2O_3P, M+H]^+$= 519.1832; Found: 519.1831. $[\alpha]_D^{25}$ = +12.75 (c =

1.2, $CHCl_3$). **HPLC:** 70% ee. (HPLC condition: Chiralpak AD column, *n*-hexane/*i*-PrOH = 70:30, 1 mL/min, 220 nm, major enantiomer t_r = 18.2 min, minor enantiomer t_r = 15.7 min.)

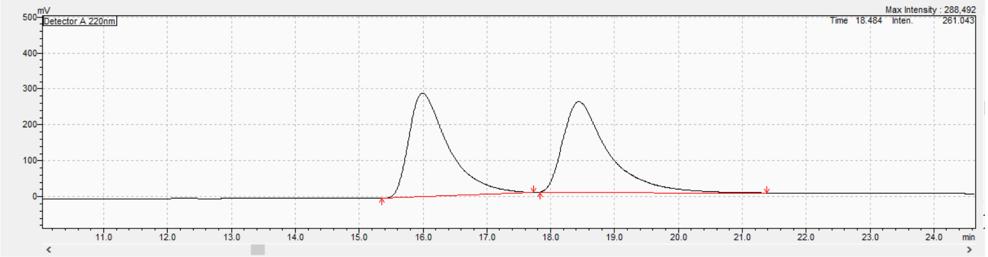

Results View - Peak Table

Peak Table | Compound | Group | Calibration Curve

Peak#	Ret. Time	Area	Height	Mark	Conc.	Unit	ID#	Area%
1	15.995	12459728	289085		50.291			50.291
2	18.440	12315459	252162	M	49.709			49.709
Total		24775187	541247		100.000			100.000

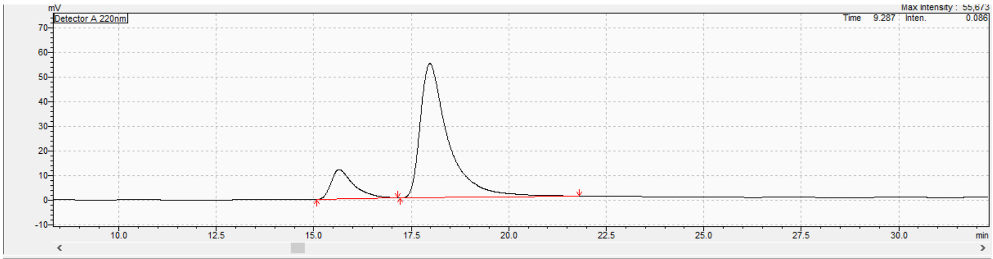

Results View - Peak Table

Peak Table | Compound | Group | Calibration Curve

Peak#	Ret. Time	Area	Height	Mark	Conc.	Unit	ID#	Area%
1	15.652	484162	11946	M	15.160			15.160
2	17.982	2709571	54554		84.840			84.840
Total		3193734	66500		100.000			100.000

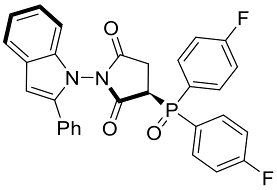

(*P*,*R*)-3-(Bis(4-fluorophenyl)phosphoryl)-1-(2-phenyl-1H-indol-1-yl)pyrrolidine-2,5-dione (3w)

Prepared according to the general procedure A in 93% yield as a colorless oil. **^{1}H NMR** (500 MHz, $CDCl_3$) δ 7.97-7.88 (m, 2H), 7.81-7.70 (m, 2H), 7.62-7.54 (m, 1H), 7.45-7.33 (m, 6H), 7.23-7.12 (m, 5H), 6.85-6.78 (m, 1H), 6.72-6.68 (m, 1H), 3.90-3.81 (m, 1H), 3.33-3.22 (m, 1H), 2.95 (m, 1H). **^{13}C NMR** (125 MHz, $CDCl_3$) δ 170.2 (d, *J* = 170.6 Hz), 165.8 (d, *J* = 239.8 Hz), 140.9, 137.4, 134.6 (d, *J* = 9.4 Hz), 134.0 (d, *J* = 9.3 Hz), 130.4, 129.0, 128.8, 128.2, 127.7, 127.2, 124.0, 122.5, 121.25, 117.1 (dd, *J* = 20.3, 12.4 Hz), 116.70 (dd, *J* = 20.2, 12.4 Hz), 109.2, 104.1, 41.1 (d, *J* = 61.4 Hz), 28.0. **^{31}P NMR** (162 MHz, $CDCl_3$) δ 29.34. **^{19}F NMR** (471 MHz, $CDCl_3$) δ -103.81, -104.01. **HRMS (ESI):** m/z Calcd for $[C_{30}H_{21}F_2N_2O_3P, M+H]^+$: 527.1331; Found:

527.1331. $[\alpha]_D^{25}$ = +29.55 (c = 2.17, $CHCl_3$). **HPLC:** 98% ee. (HPLC condition: Chiralpak AD column, *n*-hexane/*i*-PrOH = 70:30, 1 mL/min, 220 nm, major enantiomer t_r = min, minor enantiomer t_r = min.)

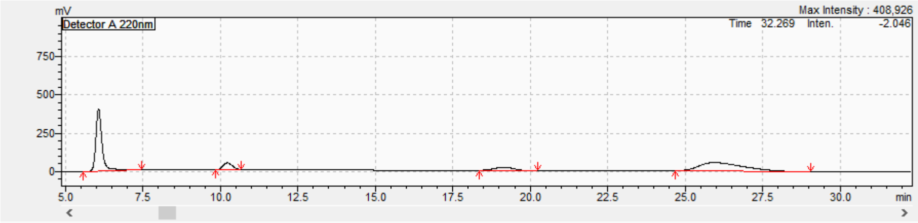

Results View - Peak Table

Peak Table | Compound | Group | Calibration Curve

Peak#	Ret. Time	Area	Height	Mark	Conc.	Unit	ID#	Name	Area%
1	6.065	5566181	404450	M	42.977				42.977
2	10.225	960718	45392	M	7.418				7.418
3	19.141	961152	21865	M	7.421				7.421
4	25.946	5463543	54626	M	42.184				42.184
Total		12951593	526333		100.000				100.000

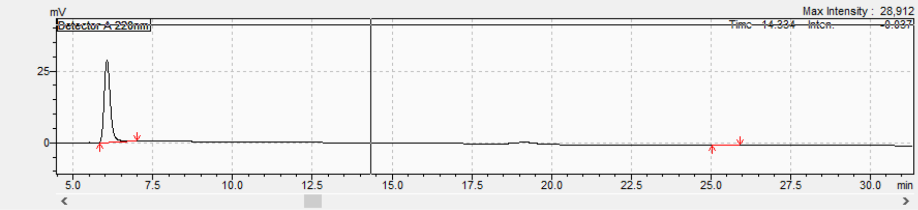

Results View - Peak Table

Peak Table | Compound | Group | Calibration Curve

Peak#	Ret. Time	Area	Height	Mark	Conc.	Unit	ID#	Name	Area%
1	6.056	380415	28779	M	99.008				99.008
2	25.921	3812	84	M	0.992				0.992
Total		384227	28863		100.000				100.000

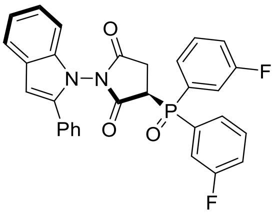

(*P*,*R*)-3-(Bis(3-fluorophenyl)phosphoryl)-1-(2-phenyl-1H-indol-1-yl)pyrrolidine-2,5-dione (3x)

Prepared according to the general procedure A in 99% yield as a colorless oil. **^{1}H NMR** (500 MHz, $CDCl_3$) δ 7.71-7.55 (m, 5H), 7.50-7.47 (m, 1H), 7.43-7.36 (m, 5H), 7.31 (m, 3H), 7.23-7.16 (m, 2H), 7.02 (d, *J* = 7.8 Hz, 1H), 6.73-6.70 (m, 1H), 3.94 – 3.82 (m, 1H), 3.30-3.17 (m, 1H), 2.96 (m, 1H). **^{13}C NMR** (125 MHz, $CDCl_3$) δ 169.80 (d, *J* = 206.2 Hz), 162.89 (d, *J* = 286.9 Hz), 140.84, 137.46, 131.64 (dd, *J* = 14.2, 7.6 Hz), 131.26 (dd, *J* = 14.6, 7.3 Hz), 130.39, 129.61, 129.18, 128.97, 128.81, 128.19, 127.89, 127.61 (d, *J* = 6.2 Hz), 127.18, 126.89 (d, *J* = 6.0 Hz), 124.20, 123.67, 122.52, 121.59, 121.24, 120.68 (dd, *J* = 21.0, 14.4 Hz), 118.80 (dd, *J* = 22.9, 10.6 Hz), 118.32 (dd, *J* = 23.0, 10.3 Hz), 109.33, 104.19, 40.80 (d, *J* = 61.8 Hz), 28.13. **^{31}P NMR:** (162

MHz, $CDCl_3$) δ 28.69. **HRMS (ESI):** m/z Calcd for $[C_{30}H_{21}F_2N_2O_3P, M+H]^+$: 527.1331; Found: 527.1334. $[\alpha]_D^{25}$ = +20.54 (c = 2.4, $CHCl_3$). **HPLC:** 93% ee. (HPLC condition: Chiralpak AD column, *n*-hexane/*i*-PrOH = 70:30, 0.8 mL/min, 220 nm, major enantiomer t_r = 8.0 min, minor enantiomer t_r = 13.0 min.)

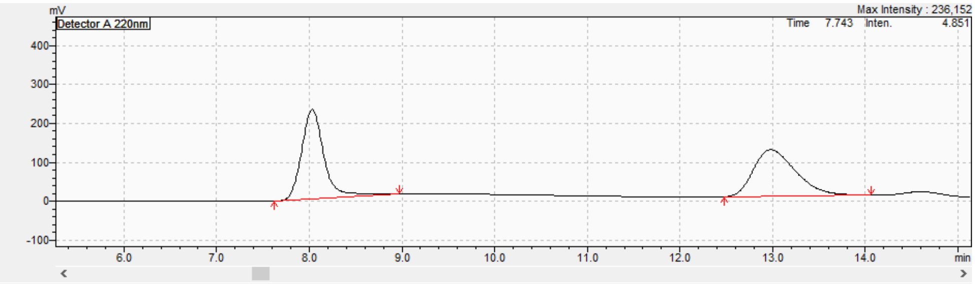

Results View - Peak Table

Peak Table | Compound | Group | Calibration Curve

Peak#	Ret. Time	Area	Height	Mark	Conc.	Unit	ID#	Name	Area%
1	8.033	3849787	229741	M	50.115				50.115
2	12.978	3832054	120153	M	49.885				49.885
Total		7681840	349893		100.000				100.000

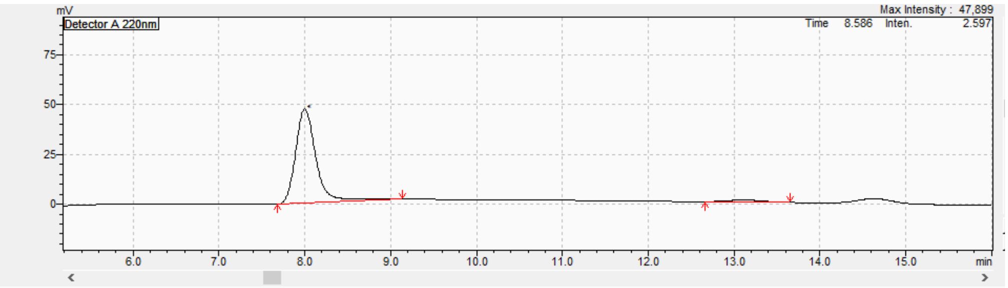

Results View - Peak Table

Peak Table | Compound | Group | Calibration Curve

Peak#	Ret. Time	Area	Height	Mark	Conc.	Unit	ID#	Name	Area%
1	8.001	780745	47401	M	96.492				96.492
2	13.111	28380	995	M	3.508				3.508
Total		809126	48396		100.000				100.000

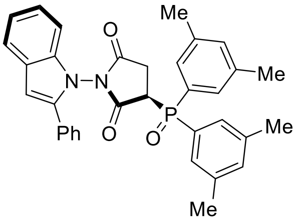

(*P*,*R*)-3-(Bis(3,5-dimethylphenyl)phosphoryl)-1-(2-phenyl-1H-indol-1-yl)pyrrolidine-2,5-dione (3y)

Prepared according to the general procedure A in 53% yield as a colorless oil. **^{1}H NMR** (500 MHz, $CDCl_3$) δ 7.60-7.56 (m, 1H), 7.53 (d, *J* = 12.3 Hz, 2H), 7.44-7.35 (m, 7H), 7.24-7.16 (m, 4H), 6.96-6.89 (m, 1H), 6.70 (s, 1H), 3.90 (td, *J* = 10.3, 3.4 Hz, 1H), 3.28 (tdd, *J* = 21.0, 16.4, 4.3 Hz, 1H), 2.93 (dt, *J* = 19.3, 10.2 Hz, 1H), 2.37 (s, 6H), 2.29 (s, 6H). **^{13}C NMR** (125 MHz, $CDCl_3$) δ 171.3, 169.6, 140.8, 139.1 (d, *J* = 13.0 Hz), 138.9 (d, *J* = 13.2 Hz), 137.5, 134.8 (d, *J* = 13.4 Hz), 130.6, 129.6, 129.4 (d,

J = 10.0 Hz), 128.9, 128.8, 128.7 (d, J = 7.6 Hz), 128.2, 127.9, 127.1, 124.1, 122.3, 121.0, 109.5, 104.0, 41.1 (d, J = 58.6 Hz), 28.5, 21.5. **^{31}P NMR:** (162 MHz, $CDCl_3$) δ 30.98. **HRMS (ESI):** m/z Calcd for $[C_{34}H_{31}N_2O_3P, M+H]^+$: 547.2145; Found: 547.2144. $[\alpha]_D^{25}$ = +31.85 (c = 1.3, $CHCl_3$). **HPLC:** 95% ee. (HPLC condition: Chiralpak AD column, *n*-hexane/*i*-PrOH = 90:10, 1 mL/min, 220 nm, major enantiomer t_r = 12.5 min, minor enantiomer t_r = 13.8 min.)

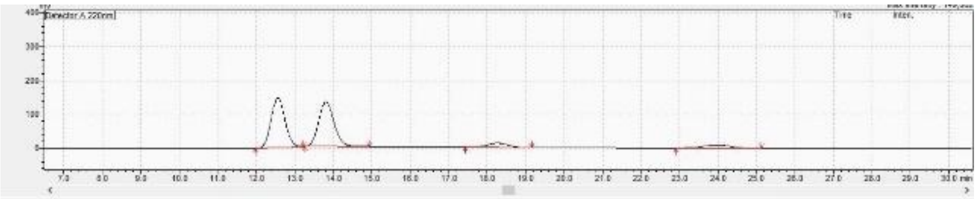

Peak#	Ret. Time	Area	Height	Mark	Conc.	Unit	ID#	Name	Area%
1	12.562	3664822	146053		44.549				44.549
2	13.810	3635596	130579	M	44.194				44.194
3	18.270	458008	12404		5.578				5.578
4	23.959	467213	9458	V	5.679				5.679
Total		8226459	298564		100.000				100.000

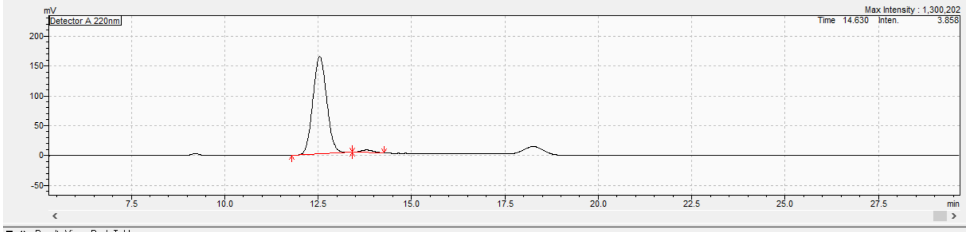

Peak#	Ret. Time	Area	Height	Mark	Conc.	Unit	ID#	Area%
1	12.543	4206478	163260	M	97.692			97.692
2	13.799	99390	4178	M	2.308			2.308
Total		4305868	167438		100.000			100.000

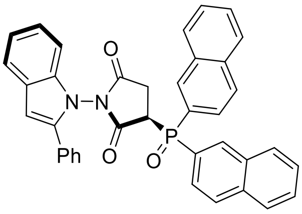

(*P*,*R*)-3-(Di(naphthalen-2-yl)phosphoryl)-1-(2-phenyl-1H-indol-1-yl)pyrrolidine-2,5-dione (3z)

Prepared according to the general procedure A in 84% yield as a white solid. **^{1}H NMR** (500 MHz, $CDCl_3$) δ 8.67 (d, J = 13.9 Hz, 1H), 8.51 (d, J = 14.1 Hz, 1H), 7.98 (d, J = 9.2 Hz, 2H), 7.93-7.85 (m, 5H), 7.77 (d, J = 9.1 Hz, 1H), 7.66-7.53 (m, 5H), 7.46-7.34 (m, 5H), 7.11 (s, 1H), 6.86 (s, 1H), 6.76 (s, 1H), 6.68 (s, 1H), 4.13 (t, J = 9.9 Hz, 1H), 3.43 (t, J = 17.7 Hz, 1H), 3.09-2.94 (m, 1H). **^{13}C NMR** (125 MHz, $CDCl_3$) δ 171.1, 169.7, 140.8, 137.4, 135.3, 135.1, 134.4 (d, J = 9.0 Hz), 134.1 (d, J = 8.8 Hz), 132.7,

132.6, 132.5, 130.5, 129.4, 129.4, 129.3, 129.2, 129.1, 128.9, 128.7, 128.2, 128.0 (d, *J* = 6.4 Hz), 127.8, 127.6, 127.4, 127.0, 126.6, 126.1 (d, *J* = 11.1 Hz), 125.3 (d, *J* = 10.9 Hz), 123.9, 122.3, 121.1, 109.4, 104.0, 41.2 (d, *J* = 59.6 Hz), 28.4. **^{31}P NMR:** (162 MHz, $CDCl_3$) δ 30.72. **HRMS (ESI):** m/z Calcd for [$C_{38}H_{27}N_2O_3P$, M+H]$^+$: 591.1832; Found: 591.1831. $[\alpha]_D^{25}$ = +127.7 (c = 1.48, $CHCl_3$). **HPLC:** 87% ee. (HPLC condition: Chiralpak AD column, *n*-hexane/*i*-PrOH = 50:50, 1 mL/min, 220 nm, major enantiomer t_r = 21.1 min, minor enantiomer t_r = 10.7 min.)

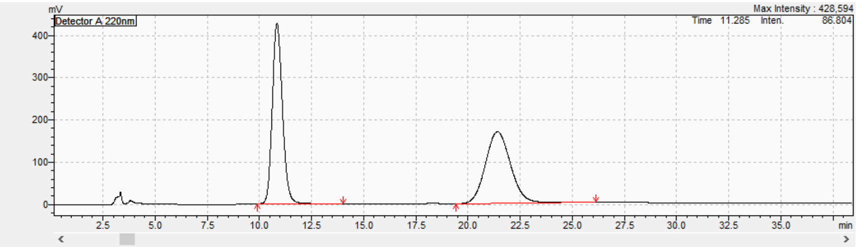

Results View - Peak Table

Peak Table | Compound | Group | Calibration Curve

Peak#	Ret. Time	Area	Height	Mark	Conc.	Unit	ID#	Name	Area%
1	10.845	14623070	427811		50.725				50.725
2	21.415	14205292	169487	M	49.275				49.275
Total		28828362	597298		100.000				100.000

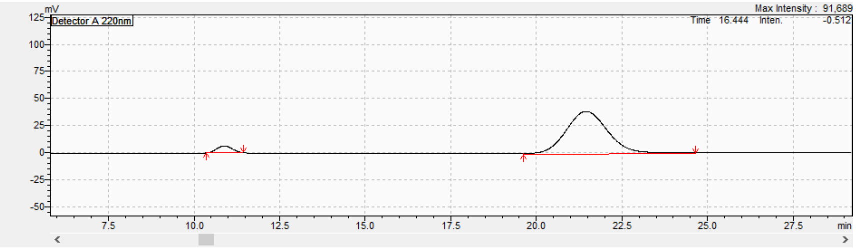

Results View - Peak Table

Peak Table | Compound | Group | Calibration Curve

Peak#	Ret. Time	Area	Height	Mark	Conc.	Unit	ID#	Name	Area%
1	10.877	202243	6522	M	5.570				5.570
2	21.452	3428431	39313	M	94.430				94.430
Total		3630674	45836		100.000				100.000

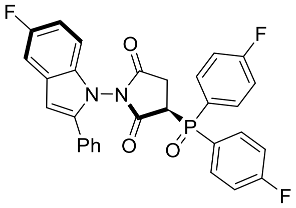

(*P*,*R*)-3-(Bis(4-fluorophenyl)phosphoryl)-1-(5-fluoro-2-phenyl-1H-indol-1-yl)pyrrolidine-2,5-dione (3aa)

Prepared according to the general procedure A in 84% yield as a colorless oil. **^{1}H NMR** (500 MHz, $CDCl_3$) δ 7.90 (m, 2H), 7.77 (m, 2H), 7.44-7.34 (m, 6H), 7.30-7.26 (m, 1H), 7.25-7.14 (m, 4H), 6.97-6.93 (m, 2H), 6.66 (s, 1H), 3.85 (m, 1H), 3.22 (m, 1H), 3.04-2.85 (m, 1H). **^{13}C NMR** (125 MHz, $CDCl_3$) δ 170.0 (d, J = 137.3 Hz), 165.8 (d, J = 256.4 Hz), 160.6, 158.3, 142.6, 134.5 (dd, J = 11.4, 9.1 Hz), 133.9 (t, J = 10.1

Hz), 130.1, 129.2, 129.0, 128.2, 127.90 (d, J = 6.6 Hz), 126.1, 125.3, 125.0, 124.3, 117.1 (dd, J = 21.6, 13.7 Hz), 116.7 (dd, J = 21.7, 13.8 Hz), 112.1 (d, J = 26.3 Hz), 110.4 (d, J = 9.7 Hz), 106.5 (d, J = 24.3 Hz), 104.0 (d, J = 4.3 Hz), 41.1 (d, J = 60.7 Hz), 28.2. **^{31}P NMR:** (162 MHz, $CDCl_3$) δ 27.02. **^{19}F NMR** (377 MHz, $CDCl_3$) δ -106.18 (d, *J* = 1.5 Hz), -106.47 (d, *J* = 1.2 Hz), -124.03. **HRMS (ESI):** m/z Calcd for $[C_{30}H_{20}F_3N_2O_3P, M+H]^+$: 545.1236; Found: 545.1232. $[\alpha]_D^{25}$ = +39.70 (c = 2.3, $CHCl_3$). **HPLC:** 93.5% ee. (HPLC condition: Chiralpak AD column, *n*-hexane/*i*-PrOH = 70:30, 1 mL/min, 220 nm, major enantiomer t_r = 6.5 min, minor enantiomer t_r = 15.0 min.)

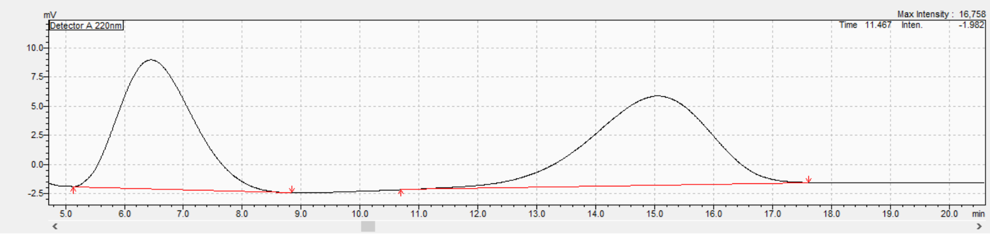

Peak#	Ret. Time	Area	Height	Mark	Conc.	Unit	ID#	Area%
1	6.449	969417	11057		47.154			47.154
2	15.051	1086428	7653	M	52.846			52.846
Total		2055845	18709		100.000			100.000

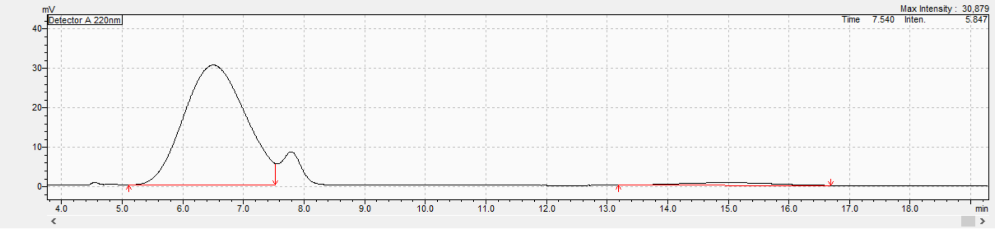

Peak#	Ret. Time	Area	Height	Mark	Conc.	Unit	ID#	Area%
1	6.504	2159445	30491	M	96.757			96.757
2	14.981	72381	676	M	3.243			3.243
Total		2231826	31167		100.000			100.000

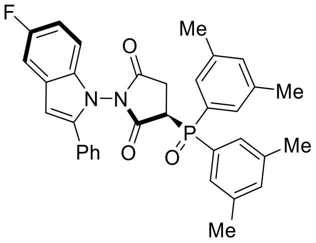

(*P*,*R*)-3-(Bis(3,5-dimethylphenyl)phosphoryl)-1-(5-fluoro-2-phenyl-1H-indol-1-yl)pyrrolidine-2,5-dione(3ab)

Prepared according to the general procedure A in 57% yield as a colorless oil. **^{1}H NMR** (400 MHz, $CDCl_3$) δ 7.42 (s, 1H), 7.38 (s, 1H), 7.36-7.23 (m, 8H), 7.15-7.08 (m, 3H), 6.96-6.89 (m, 1H), 6.86 (dd, *J* = 9.2, 2.4 Hz, 1H), 6.55 (d, *J* = 0.8 Hz, 1H), 3.84-

3.75 (m, 1H), 3.14 (m, 1H), 2.81 (m, 1H), 2.27 (s, 6H), 2.20 (s, 6H). **^{13}C NMR** (100 MHz, $CDCl_3$) δ 170.4 (d, *J* = 165.2 Hz), 159.4 (d, *J* = 236.8 Hz), 142.5, 139.2 (d, *J* = 13.0 Hz), 138.8 (d, *J* = 13.2 Hz), 134.8, 133.9, 130.4, 130.2, 129.6, 129.3, 129.2, 128.9, 128.7, 128.6, 128.2, 127.7, 127.6, 112.2 (d, *J* = 26.2 Hz), 110.7 (d, *J* = 9.7 Hz), 106.3 (d, *J* = 24.3 Hz), 103.8 (d, *J* = 4.2 Hz), 41.0 (d, *J* = 58.0 Hz), 28.6, 21.5. **^{31}P NMR:** (162 MHz, $CDCl_3$) δ 31.45. **^{19}F NMR** (377 MHz, CDCl3) δ -122.03. **HRMS (ESI):** m/z Calcd for $[C_{34}H_{30}FN_2O_3P, M+H]^+$: 565.2051; Found: 565.2049.

$[\alpha]_D^{25}$ = +16.53 (c = 1.3, $CHCl_3$). **HPLC:** 90% ee. (HPLC condition: Chiralpak OD column, *n*-hexane/*i*-PrOH = 95:5, 1 mL/min, 220 nm, major enantiomer t_r = 6.7 min, minor enantiomer t_r = 5.4 min.)

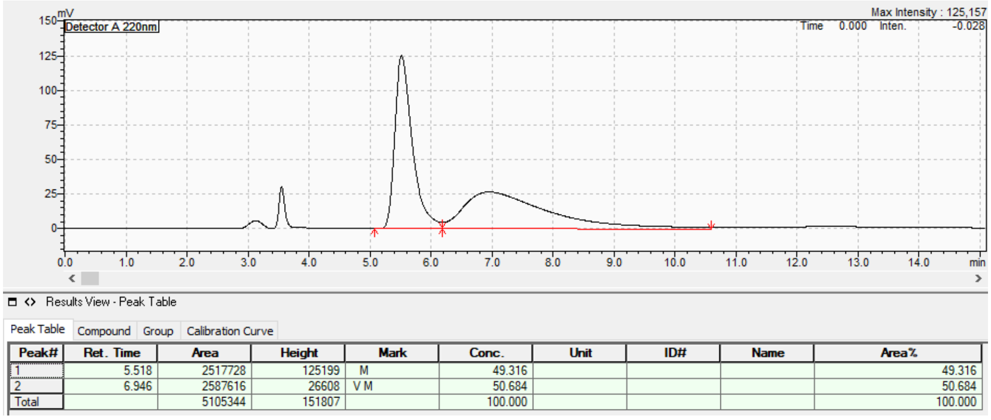

Peak#	Ret. Time	Area	Height	Mark	Conc.	Unit	ID#	Name	Area%
1	5.518	2517728	125199	M	49.316				49.316
2	6.946	2587616	26608	V M	50.684				50.684
Total		5105344	151807		100.000				100.000

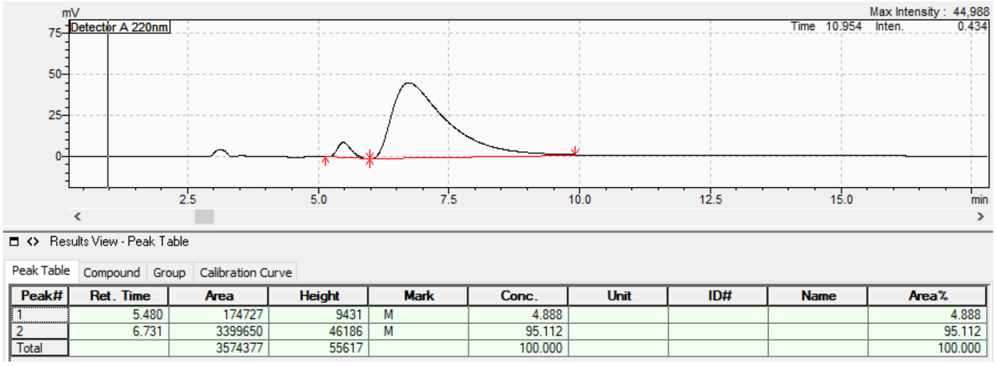

Peak#	Ret. Time	Area	Height	Mark	Conc.	Unit	ID#	Name	Area%
1	5.480	174727	9431	M	4.888				4.888
2	6.731	3399650	46186	M	95.112				95.112
Total		3574377	55617		100.000				100.000

General procedure B

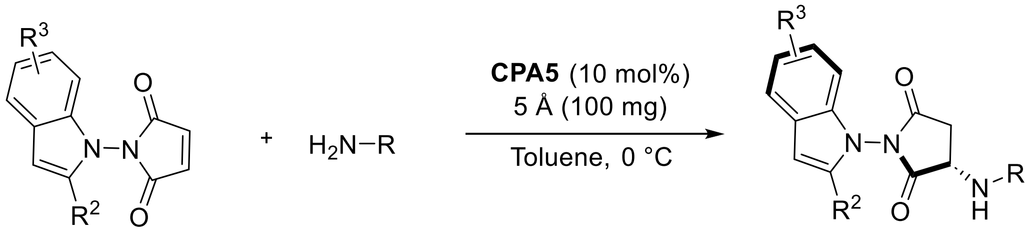

1 **4** **5**

To a mixture of **1a** (0.1 mmol, 1.00 equiv.), **CPA5** (0.02 mmol, 20 mol%), 5 Å molecular sieves (100 mg) in toluene (1.00 mL) in a dry tube was added **4a** (0.12 mmol, 1.20 equiv.) in toluene (1.00 mL) dropwise under argon atmosphere at 0 °C. After stirring for 3h the reaction was diluted with dichloromethane, filtered and collect the filtrate. Evaporation on the rotavapor gave the crude product, then purification by column.

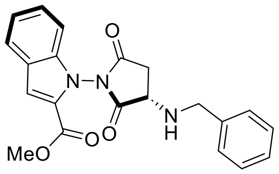

Methyl (*P*,*S*)-1-(3-(benzylamino)-2,5-dioxopyrrolidin-1-yl)-1H-indole-2-carboxylate(5a)

Prepared according to the general procedure B in 91% yield as a colorless oil. **^{1}H NMR** (500 MHz, $CDCl_3$) δ 7.72 (dt, *J* = 8.1, 1.0 Hz, 1H), 7.44 (d, *J* = 1.0 Hz, 1H), 7.42-7.40 (m, 3H), 7.39-7.35 (m, 4H), 7.32 (dd, *J* = 6.7, 2.0 Hz, 1H), 7.13 (dt, *J* = 8.2, 0.9 Hz, 1H), 4.09 (d, *J* = 6.3 Hz, 2H), 4.07-4.04 (m, 1H), 3.86 (s, 3H), 3.25 (dd, *J* = 18.3, 8.7 Hz, 1H), 2.88 (dd, *J* = 18.5, 5.3 Hz, 1H), 2.10 (s, 1H). **^{13}C NMR** (125 MHz, $CDCl_3$) δ 174.7, 171.9, 160.9, 138.9, 138.1, 128.8, 128.6, 127.7, 127.1, 125.2, 124.7, 122.8, 112.1, 109.0, 53.7, 52.2, 51.3, 35.7. **HRMS-EI (m/z):** Calcd for [$C_{21}H_{20}N_3O_4$, M+H]$^+$: 378.1448; found: 378.1454. $[\alpha]_D^{25}$ = -10.4 (c = 1, $CHCl_3$). **HPLC:** 83% ee. (HPLC condition: Chiralpak AD column, *n*-hexane/*i*-PrOH = 90:10, 1 mL/min, 220 nm, major enantiomer t_r = 20.80 min, minor enantiomer t_r = 25.95 min.)

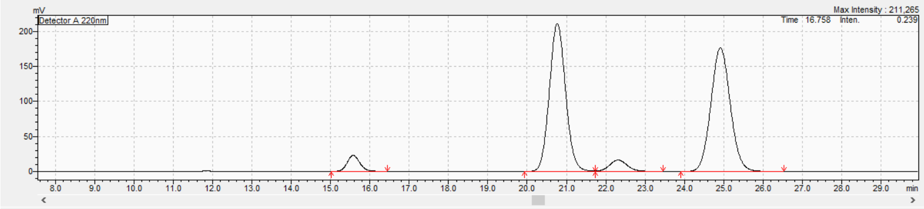

Results View - Peak Table

Peak Table | Compound | Group | Calibration Curve

Peak#	Ret. Time	Area	Height	Mark	Conc.	Unit	ID#	Name	Area%
1	15.576	504103	22796		3.839				3.839
2	20.768	6057160	211185		46.129				46.129
3	22.315	508137	16170	V	3.870				3.870
4	24.918	6061475	176571		46.162				46.162
Total		13130875	426721		100.000				100.000

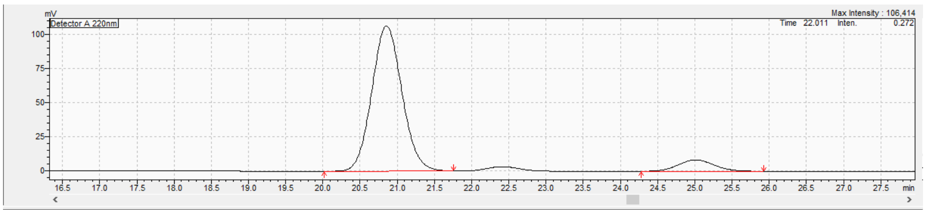

Results View - Peak Table

Peak Table | Compound | Group | Calibration Curve

Peak#	Ret. Time	Area	Height	Mark	Conc.	Unit	ID#	Name	Area%
1	20.856	2998635	106485	M	91.442				91.442
2	25.012	280648	8337	M	8.558				8.558
Total		3279283	114822		100.000				100.000

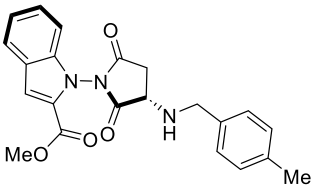

Methyl (*P*,*S*)-1-(3-((4-methylbenzyl)amino)-2,5-dioxopyrrolidin-1-yl)-1H-indole-2-carboxylate(5b)

Prepared according to the general procedure B in 83% yield as a colorless oil. **^{1}H NMR** (400 MHz, $CDCl_3$) δ 7.72 (d, *J* = 8.5 Hz, 1H), 7.43 (d, *J* = 0.9 Hz, 1H), 7.40-7.35 (m, 1H), 7.29 (d, *J* = 7.8 Hz, 2H), 7.18 (d, *J* = 7.8 Hz, 3H), 7.15-7.09 (m, 1H), 4.04 (d, *J* = 2.7 Hz, 2H), 3.95 (d, *J* = 4.1 Hz, 1H), 3.86 (s, 3H), 3.23 (dd, *J* = 18.3, 8.7 Hz, 1H), 2.87 (dd, *J* = 18.3, 5.1 Hz, 1H), 2.37 (d, *J* = 1.8 Hz, 3H), 2.12 (s, 1H). **^{13}C NMR** (100 MHz, $CDCl_3$) δ 174.7, 171.9, 160.9, 138.1, 137.3, 135.9, 129.5, 128.6, 127.1, 125.3, 124.7, 123.4, 122.7, 112.0, 109.0, 53.6, 52.2, 51.1, 35.8, 21.3. **HRMS-EI (m/z):** Calcd for $[C_{22}H_{22}N_3O_4, M+H]^+$: 392.1605; found: 392.1607. $[\alpha]_D^{25}$ = -8.0 (c = 0.68, $CHCl_3$). **HPLC:** 83% ee. (HPLC condition: Chiralpak AD column, *n*-hexane/*i*-PrOH = 90:10, 1 mL/min, 220 nm, major enantiomer t_r = 18.73 min, minor enantiomer t_r = 23.85 min.)

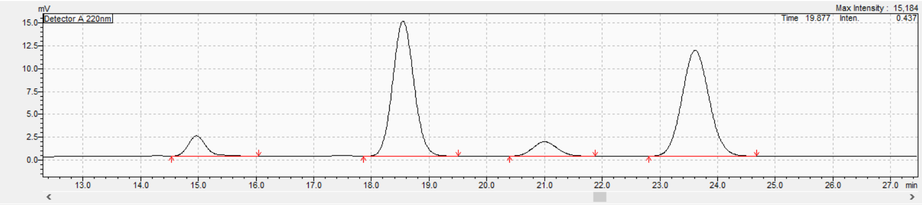

Results View - Peak Table

Peak Table | Compound | Group | Calibration Curve

Peak#	Ret. Time	Area	Height	Mark	Conc.	Unit	ID#	Name	Area%
1	14.968	48626	2217	V	5.723				5.723
2	18.548	377056	14744		44.377				44.377
3	21.001	46773	1583		5.505				5.505
4	23.611	377205	11564		44.395				44.395
Total		849661	30109		100.000				100.000

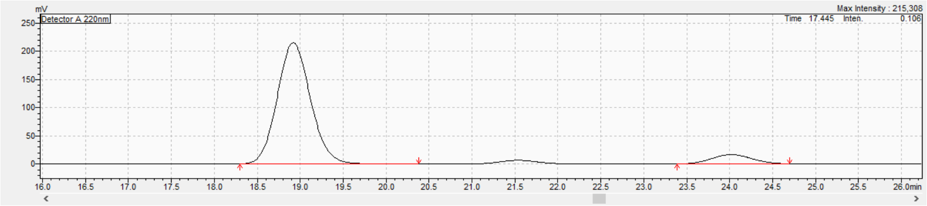

Results View - Peak Table

Peak Table | Compound | Group | Calibration Curve

Peak#	Ret. Time	Area	Height	Mark	Conc.	Unit	ID#	Name	Area%
1	18.920	5787568	214956	M	91.475				91.475
2	24.018	539363	16444	M	8.525				8.525
Total		6326931	231400		100.000				100.000

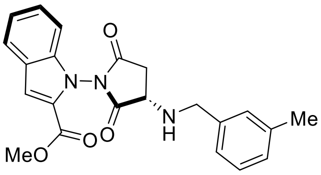

Methyl (*P*,*S*)-1-(3-((3-methylbenzyl)amino)-2,5-dioxopyrrolidin-1-yl)-1H-indole-2-carboxylate(5c)

Prepared according to the general procedure B in 87% yield as a colorless oil. **^{1}H NMR** (500 MHz, $CDCl_3$) δ 7.72 (dt, *J* = 8.0, 1.0 Hz, 1H), 7.43 (d, *J* = 1.0 Hz, 1H), 7.38 (ddd, *J* = 8.2, 7.0, 1.1 Hz, 1H), 7.25 – 7.18 (m, 4H), 7.14-7.09 (m, 2H), 4.07 (dd, *J* = 5.8, 2.9 Hz, 1H), 4.04 (d, *J* = 4.5 Hz, 2H), 3.86 (s, 3H), 3.25 (dd, *J* = 18.3, 8.7 Hz, 1H), 2.89 (dd, *J* = 18.4, 5.1 Hz, 1H), 2.37 (s, 3H), 2.09 (s, 1H). **^{13}C NMR** (126 MHz, $CDCl_3$) δ 174.7, 171.9, 160.9, 138.8, 138.5, 138.1, 129.4, 128.7, 128.4, 127.1, 125.7, 125.2, 124.7, 123.4, 122.8, 53.7, 52.2, 51.3, 35.7, 21.5. **HRMS-EI (m/z):** Calcd for $[C_{22}H_{22}N_3O_4, M+H]^+$: 392.1605; found: 392.1612. $[\alpha]_D^{25}$ = -8.4 (c = 1.36, $CHCl_3$). **HPLC:** 73% ee. (HPLC condition: Chiralpak AD column, *n*-hexane/*i*-PrOH = 90:10, 1 mL/min, 220 nm, major enantiomer t_r = 18.87 min, minor enantiomer t_r = 23.29 min.)

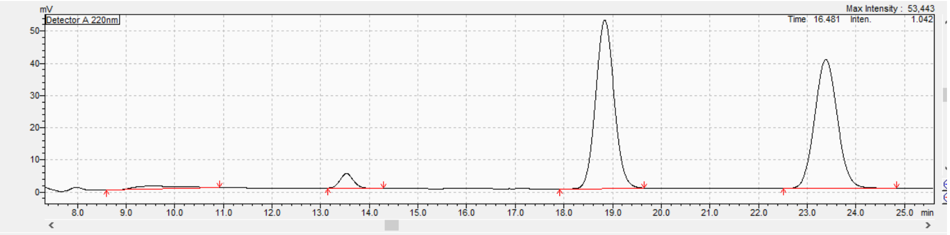

Results View - Peak Table

Peak Table | Compound | Group | Calibration Curve

Peak#	Ret. Time	Area	Height	Mark	Conc.	Unit	ID#	Name	Area%
1	9.515	59865	942	M	2.118				2.118
2	13.530	87604	4538		3.099				3.099
3	18.839	1377627	52303	M	48.737				48.737
4	23.383	1301586	40131	V	46.046				46.046
Total		2826681	97914		100.000				100.000

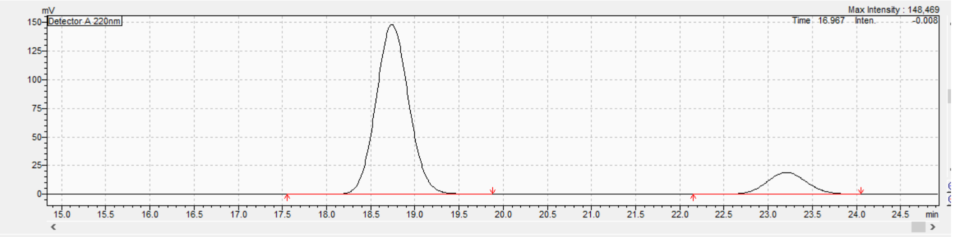

Results View - Peak Table

Peak Table | Compound | Group | Calibration Curve

Peak#	Ret. Time	Area	Height	Mark	Conc.	Unit	ID#	Name	Area%
1	18.741	3828094	148318	M	86.462				86.462
2	23.205	599387	19055	M	13.538				13.538
Total		4427481	167373		100.000				100.000

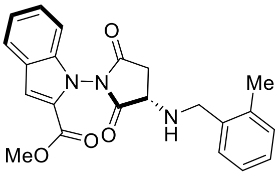

Methyl (*P*,*S*)-1-(3-((2-methylbenzyl)amino)-2,5-dioxopyrrolidin-1-yl)-1H-indole-2-carboxylate(5d)

Prepared according to the general procedure B in 98% yield as a colorless oil. **^{1}H NMR** (400 MHz, $CDCl_3$) δ 7.72 (dt, *J* = 8.0, 1.0 Hz, 1H), 7.44 (d, *J* = 0.9 Hz, 1H), 7.39 (ddt, *J* = 10.2, 6.6, 2.2 Hz, 2H), 7.24-7.17 (m, 4H), 7.15 (dd, *J* = 8.3, 1.0 Hz, 1H), 4.15-3.99 (m, 3H), 3.85 (s, 3H), 3.27 (dd, *J* = 18.4, 8.8 Hz, 1H), 2.93-2.85 (m, 1H), 2.41 (s, 3H), 2.04 (s, 1H). **^{13}C NMR** (100 MHz, $CDCl_3$) δ174.8, 171.9, 160.9, 138.1, 137.0, 130.7, 129.1, 128.0, 127.7, 127.1, 126.2, 125.2, 124.7, 123.4, 122.8, 112.0, 1099.0, 54.0, 52.1, 49.0, 35.6, 19.1. **HRMS-EI (m/z):** Calcd for $[C_{22}H_{22}N_3O_4, M+H]^+$: 392.1605; found: 392.1609. $[\alpha]_D^{25}$ = -8.9 (c = 0.78, $CHCl_3$). **HPLC:** 89% ee. (HPLC condition: Chiralpak AD column, *n*-hexane/*i*-PrOH = 90:10, 1 mL/min, 220 nm, major enantiomer t_r = 20.06 min, minor enantiomer t_r = 23.51 min.)

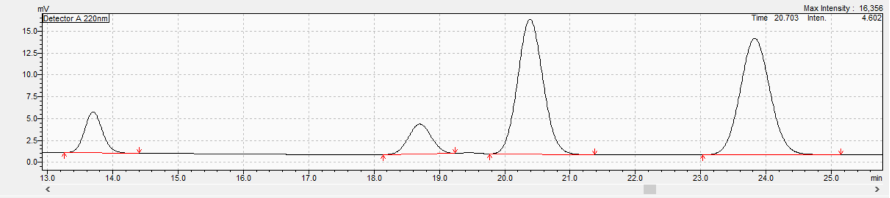

Results View - Peak Table

Peak Table | Compound | Group | Calibration Curve

Peak#	Ret. Time	Area	Height	Mark	Conc.	Unit	ID#	Name	Area%
1	13.700	89109	4704		8.772				8.772
2	18.700	83491	3412		8.219				8.219
3	20.387	417677	15433		41.116				41.116
4	23.829	425582	13327		41.894				41.894
Total		1015859	36877		100.000				100.000

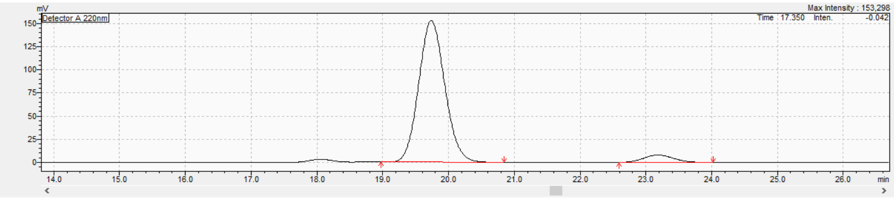

Results View - Peak Table

Peak Table | Compound | Group | Calibration Curve

Peak#	Ret. Time	Area	Height	Mark	Conc.	Unit	ID#	Name	Area%
1	19.738	4220934	152966	M	94.314				94.314
2	23.186	254489	8016	M	5.686				5.686
Total		4475423	160982		100.000				100.000

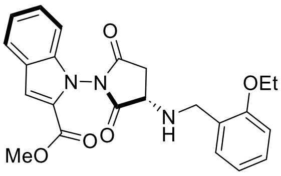

Methyl (*P*,*S*)-1-(3-((2-ethoxybenzyl)amino)-2,5-dioxopyrrolidin-1-yl)-1H-indole-2-carboxylate(5e)

Prepared according to the general procedure B in 77% yield as a white solid. **^{1}H NMR** (400 MHz, $CDCl_3$) δ 7.71 (d, *J* = 8.0 Hz, 1H), 7.43 (s, 1H), 7.39-7.34 (m, 1H), 7.29 (d, *J* = 8.0 Hz, 2H), 7.24 (d, *J* = 7.6 Hz, 1H), 7.10 (s, 1H), 6.94 (t, *J* = 7.4 Hz, 1H), 6.90 (d, *J* = 8.4 Hz, 1H), 4.18-4.07 (m, 3H), 4.06-4.01 (m, 1H), 3.94 (d, *J* = 13.6 Hz, 1H), 3.86 (s, 3H), 3.24 (dd, *J* = 18.1, 8.4 Hz, 1H), 2.96 (dd, *J* = 18.0, 5.5 Hz, 1H), 2.53 (s, 1H), 1.45 (t, *J* = 6.9 Hz, 3H). **^{13}C NMR** (100 MHz, $CDCl_3$) δ 174.5, 172.0, 130.4, 129.1, 127.0, 126.9, 124.7, 123.3, 122.7, 120.6, 112.0, 111.5, 109.0, 63.8, 53.4, 52.2, 47.2, 35.6, 15.0. **HRMS-EI (m/z):** Calcd for [$C_{23}H_{24}N_3O_5$, M+H]$^+$: 422.171; found: 422.1711. $[\alpha]_D^{25}$ = +1.64 (c =1.0 , $CHCl_3$). **HPLC:** 92% ee. (HPLC condition: Chiralpak AD column, *n*-hexane/*i*-PrOH = 90:10, 1 mL/min, 220 nm, major enantiomer t_r = 22.89 min, minor enantiomer t_r = 26.18 min.)

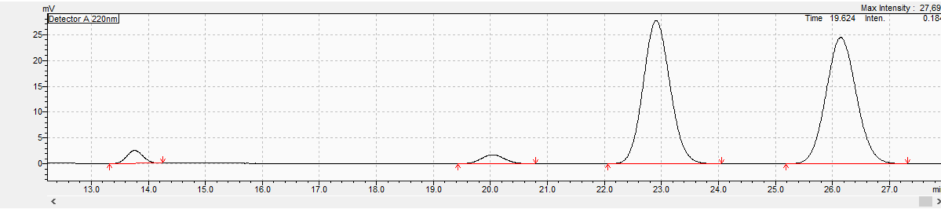

Results View - Peak Table

Peak Table | Compound | Group | Calibration Curve

Peak#	Ret. Time	Area	Height	Mark	Conc.	Unit	ID#	Name	Area%
1	13.761	49450	2499		2.585				2.585
2	20.041	49687	1746		2.598				2.598
3	22.909	907786	27637		47.458				47.458
4	26.148	905884	24379		47.359				47.359
Total		1912806	56260		100.000				100.000

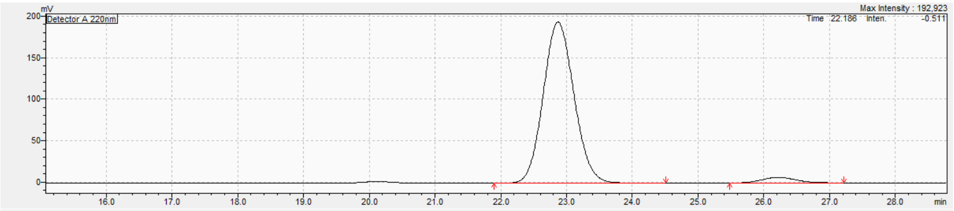

Results View - Peak Table

Peak Table | Compound | Group | Calibration Curve

Peak#	Ret. Time	Area	Height	Mark	Conc.	Unit	ID#	Name	Area%
1	22.871	6311262	193950		96.183				96.183
2	26.222	250460	6770	M	3.817				3.817
Total		6561722	200719		100.000				100.000

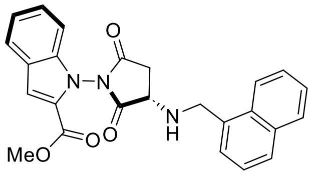

Methyl (*P*,*S*)-1-(3-((naphthalen-1-ylmethyl)amino)-2,5-dioxopyrrolidin-1-yl)-1H-indole-2-carboxylate(5f)

Prepared according to the general procedure B in 94% yield as a colorless oil. **^{1}H NMR** (500 MHz, $CDCl_3$) δ 8.24 (d, *J* = 8.2 Hz, 1H), 7.91 – 7.87 (m, 1H), 7.83 (d, *J* = 8.5 Hz, 1H), 7.76 – 7.69 (m, 1H), 7.61 – 7.49 (m, 4H), 7.48 – 7.44 (m, 2H), 7.39 (ddd, *J* = 8.2, 7.1, 1.0 Hz, 1H), 7.18 – 7.11 (m, 1H), 4.56 (d, *J* = 51.5 Hz, 2H), 4.15 (dt, *J* = 7.1, 5.0 Hz, 1H), 3.84 (s, 3H), 3.26 (dd, *J* = 18.4, 8.8 Hz, 1H), 2.91 (dd, *J* = 18.4, 5.1 Hz, 1H), 2.14 (s, 1H). **^{13}C NMR** (125 MHz, $CDCl_3$) δ 174.9, 171.9, 160.9, 138.1, 134.6, 134.1, 132.0, 131.0, 128.9, 128.5, 127.1, 126.9, 126.4, 126.0, 125.5, 125.2,124.7, 124.0, 123.4, 122.8, 112.1, 109.0, 54.0, 52.2, 48.9, 35.5. **HRMS-EI (m/z):** Calcd for [$C_{25}H_{22}N_3O_4$, M+H]$^+$: 428.1605; found: 428.161. $[\alpha]_D^{25}$ = -8.20 (c = 1.0, $CHCl_3$). **HPLC:** 82% ee. (HPLC condition: Chiralpak IE column, *n*-hexane/*i*-PrOH = 90:10, 1 mL/min, 220 nm, major enantiomer t_r = 17.45 min, minor enantiomer t_r = 20.41 min.)

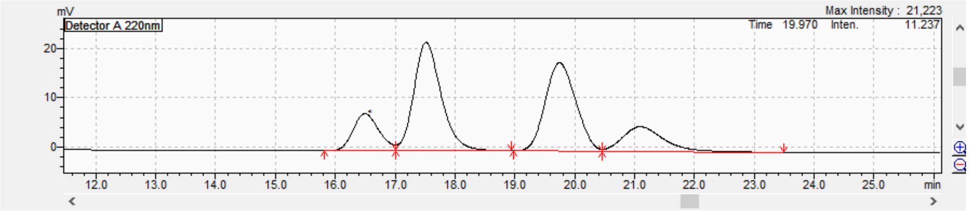

Results View - Peak Table

Peak Table | Compound | Group | Calibration Curve

Peak#	Ret. Time	Area	Height	Mark	Conc.	Unit	ID#	Name	Area%
1	16.496	227667	7550		12.590				12.590
2	17.517	701976	22011	V	38.821				38.821
3	19.744	634760	17911		35.104				35.104
4	21.091	243846	5040	V	13.485				13.485
Total		1808249	52512		100.000				100.000

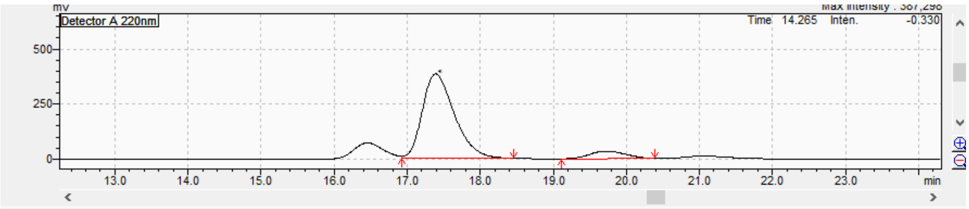

Results View - Peak Table

Peak Table | Compound | Group | Calibration Curve

Peak#	Ret. Time	Area	Height	Mark	Conc.	Unit	ID#	Name	Area%
1	17.390	11762041	385294	M	90.976				90.976
2	19.728	1166660	34253	M	9.024				9.024
Total		12928701	419547		100.000				100.000

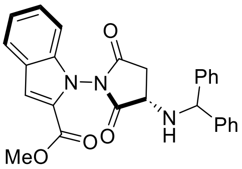

Methyl (*P*,*S*)-1-(3-(benzhydrylamino)-2,5-dioxopyrrolidin-1-yl)-1H-indole-2-carboxylate(5g)

Prepared according to the general procedure B in 57% yield as a colorless oil. **^{1}H NMR** (400 MHz, $CDCl_3$) δ 7.71 (d, *J* = 8.0 Hz, 1H), 7.54-7.49 (m, 2H), 7.48-7.44 (m, 2H), 7.42 (d, *J* = 0.8 Hz, 1H), 7.39-7.31 (m, 5H), 7.30-7.27 (m, 1H), 7.25-7.20 (m, 2H), 7.13-7.04 (m, 1H), 5.47 (s, 1H), 4.00 (dd, *J* = 8.8, 5.2 Hz, 1H), 3.81 (s, 3H), 3.19 (dd, *J* = 18.4, 8.8 Hz, 1H), 2.87 (dd, *J* = 18.4, 5.2 Hz, 1H). **^{13}C NMR** (100 MHz, $CDCl_3$) δ143.2, 142.2, 128.9, 128.8, 128.0, 127.8, 127.7, 127.5, 127.1, 123.4, 122.7, 112.0, 109.0, 65.1, 52.6, 52.1, 36.5. **HRMS-EI (m/z):** Calcd for $[C_{27}H_{24}N_3O_4, M+H]^+$: 454.1761; found: 454.1761. $[\alpha]_D^{25}$ = -5.95 (c = 1.0, $CHCl_3$). **HPLC:** 58% ee. (HPLC condition: Chiralpak AD column, *n*-hexane/*i*-PrOH = 95:5, 1 mL/min, 220 nm, major enantiomer t_r = 29.51 min, minor enantiomer t_r = 34.30 min.)

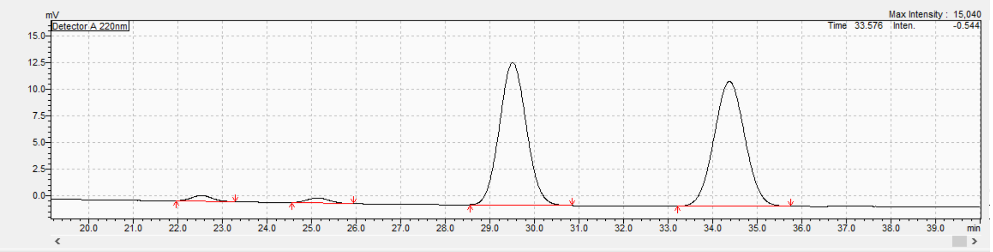

Results View - Peak Table

Peak Table | Compound | Group | Calibration Curve

Peak#	Ret. Time	Area	Height	Mark	Conc.	Unit	ID#	Name	Area%
1	22.520	16727	528		1.438				1.438
2	25.132	16563	477		1.424				1.424
3	29.511	566316	13426	V	48.685				48.685
4	34.375	563619	11744		48.453				48.453
Total		1163225	26175		100.000				100.000

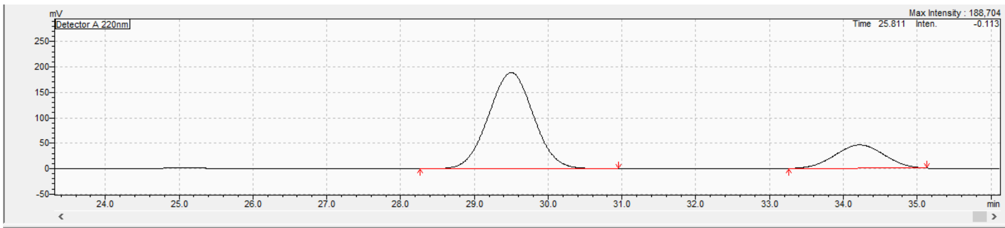

Results View - Peak Table

Peak Table | Compound | Group | Calibration Curve

Peak#	Ret. Time	Area	Height	Mark	Conc.	Unit	ID#	Name	Area%
1	29.500	7984072	188833		78.765				78.765
2	34.221	2152483	46019	M	21.235				21.235
Total		10136555	234852		100.000				100.000

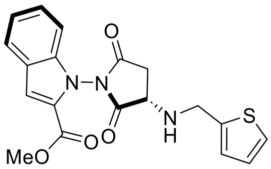

Methyl (*P*,*S*)-1-(2,5-dioxo-3-((thiophen-2-ylmethyl)amino)pyrrolidin-1-yl)-1H-indole-2-carboxylate(5h)

Prepared according to the general procedure B in 87% yield as a colorless oil. **^{1}H NMR** (400 MHz, $CDCl_3$) δ 7.72 (dd, *J* = 8.0, 1.0 Hz, 1H), 7.43 (d, *J* = 1.0 Hz, 1H), 7.41-7.32 (m, 1H), 7.30-7.27 (m, 2H),7.14-7.09 (m, 1H), 7.03 (dd, *J* = 3.5, 1.1 Hz, 1H), 7.00-6.93 (m, 1H), 4.30 (dd, *J* = 1.8, 0.9 Hz, 2H), 4.10 (dd, *J* = 8.7, 5.1 Hz, 1H), 3.86 (s, 3H), 3.27 (dd, *J* = 18.4, 8.8 Hz, 1H), 2.87 (dd, *J* = 18.4, 5.0 Hz, 1H), 2.20 (s, 1H). **^{13}C NMR** (100 MHz, $CDCl_3$) δ 174.6, 171.8, 160.9, 142.5, 138.1, 127.1, 127.0, 126.1, 125.3, 125.2, 124.7, 123.4, 112.1, 109.0, 53.3, 52.2, 46.1, 35.7. **HRMS-EI (m/z):** Calcd for $[C_{19}H_{18}N_3O_4S, M+H]^+$: 384.1013; found: 384.1013. $[\alpha]_D^{25}$ = -14.69 (c = 0.81, $CHCl_3$). **HPLC:** 88% ee. (HPLC condition: Chiralpak AD column, *n*-hexane/*i*-PrOH = 90:10, 1 mL/min, 220 nm, major enantiomer t_r = 23.62 min, minor enantiomer t_r = 27.95 min.)

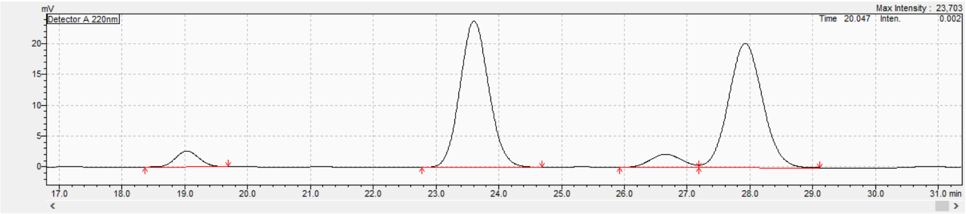

Results View - Peak Table

Peak Table | Compound | Group | Calibration Curve

Peak#	Ret. Time	Area	Height	Mark	Conc.	Unit	ID#	Name	Area%
1	19.033	67054	2586		4.031				4.031
2	23.605	758871	23799		45.616				45.616
3	26.658	78079	2179	M	4.693				4.693
4	27.924	759594	20229	V M	45.660				45.660
Total		1663598	48793		100.000				100.000

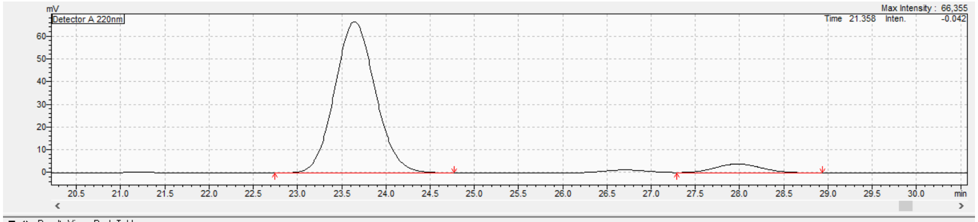

Results View - Peak Table

Peak Table | Compound | Group | Calibration Curve

Peak#	Ret. Time	Area	Height	Mark	Conc.	Unit	ID#	Name	Area%
1	23.645	2121633	66538		93.834				93.834
2	27.978	139421	3844		6.166				6.166
Total		2261054	70382		100.000				100.000

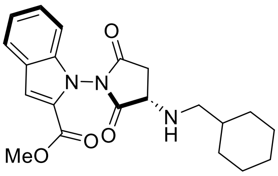

Methyl (*P*,*S*)-1-(3-((cyclohexylmethyl)amino)-2,5-dioxopyrrolidin-1-yl)-1H-indole-2-carboxylate(5i)

Prepared according to the general procedure B in 60% yield as a colorless oil. **^{1}H NMR** (500 MHz, $CDCl_3$) δ 7.71 (dt, *J* = 8.0, 0.8 Hz, 1H), 7.42 (d, *J* = 0.9 Hz, 1H), 7.41-7.35 (m, 1H), 7.25-7.22 (m, 1H), 7.13 (dd, *J* = 8.4, 0.8 Hz, 1H), 4.06 (dd, *J* = 8.5, 5.1 Hz, 1H), 3.85 (s, 2H), 3.29 (dd, *J* = 18.3, 8.7 Hz, 1H), 2.87 (dd, *J* = 18.3, 5.1 Hz, 1H), 2.77 (dd, *J* = 11.4, 6.7 Hz, 1H), 2.59-2.51 (m, 0H), 1.86-1.65 (m, 7H), 1.34-1.08 (m, 5H), 0.97 (qd, *J* = 12.3, 3.3 Hz, 3H). **^{13}C NMR** (125 MHz, $CDCl_3$) δ 174.6, 172.0, 160.8, 138.1, 127.1, 124.7, 123.4, 122.7, 112.0, 109.9, 109.0, 55.1, 54.0, 52.1, 38.3, 35.6, 31.4, 31.4, 26.7, 26.1. **HRMS-EI (m/z):** Calcd for $[C_{21}H_{26}N_3O_4, M+H]^+$: 384.1918; found: 384.1919. $[\alpha]_D^{25}$ = -18.87 (c = 1.15, $CHCl_3$). **HPLC:** 90% ee. (HPLC condition: Chiralpak AD column, *n*-hexane/*i*-PrOH = 90:10, 1 mL/min, 220 nm, major enantiomer t_r = 16.87 min, minor enantiomer t_r = 22.47 min.)

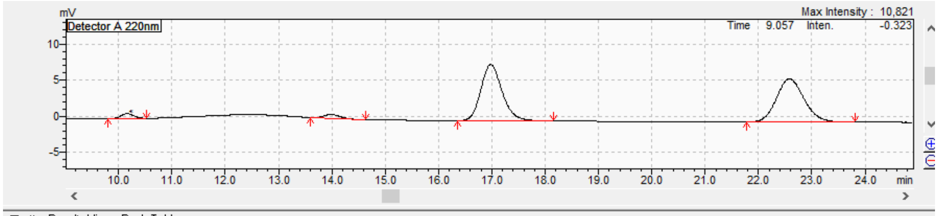

Results View - Peak Table

Peak Table | Compound | Group | Calibration Curve

Peak#	Ret. Time	Area	Height	Mark	Conc.	Unit	ID#	Name	Area%
1	10.158	11799	695		2.609				2.609
2	13.986	11005	526	M	2.433				2.433
3	16.979	214991	7778		47.535				47.535
4	22.582	214486	5978		47.423				47.423
Total		452280	14976		100.000				100.000

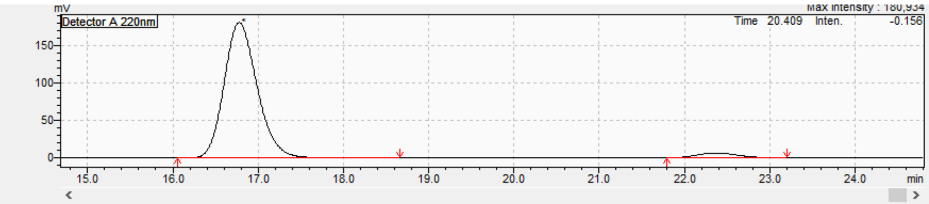

Results View - Peak Table

Peak Table | Compound | Group | Calibration Curve

Peak#	Ret. Time	Area	Height	Mark	Conc.	Unit	ID#	Name	Area%
1	16.779	4881212	181102		95.475				95.475
2	22.368	231362	6729	M	4.525				4.525
Total		5112574	187831		100.000				100.000

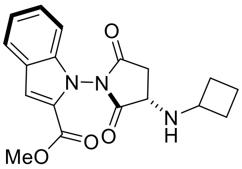

Methyl (*P*,*S*)-1-(3-(cyclobutylamino)-2,5-dioxopyrrolidin-1-yl)-1H-indole-2-carboxylate(5j)

Prepared according to the general procedure B in 58% yield as a colorless oil. **^{1}H NMR** (500 MHz, $CDCl_3$) δ 7.73-7.70 (m, 1H), 7.42 (d, *J* = 0.9 Hz, 1H), 7.38 (ddd, *J* = 8.3, 7.2, 1.0 Hz, 1H), 7.24-7.21 (m, 0H), 7.14-7.10 (m, 1H), 4.05 (dd, *J* = 8.6, 5.4 Hz, 0H), 3.86 (s, 1H), 3.58-3.51 (m, 0H), 3.27 (dd, *J* = 18.2, 8.6 Hz, 0H), 2.86 (dd, *J* = 18.2, 5.4 Hz, 0H), 2.29 (tdd, *J* = 13.6, 6.0, 4.2 Hz, 2H), 1.89 – 1.63 (m, 1H). **^{13}C NMR** (100 MHz, $CDCl_3$) δ 174.7, 171.9, 160.8, 138.1, 127.1, 125.3, 124.7, 123.4, 122.7, 112.0, 109.0, 53.2, 52.7, 52.2, 36.2, 31.6, 31.3, 14.8. **HRMS-EI (m/z):** Calcd for $[C_{18}H_{20}N_3O_4, M+H]^+$: 342.1448; found: 342.1451. $[\alpha]_D^{25}$ = -13.9 (c = 1.0, $CHCl_3$). **HPLC:** 87% ee. (HPLC condition: Chiralpak AD column, *n*-hexane/*i*-PrOH = 90:10, 1 mL/min, 220 nm, major enantiomer t_r = 18.85 min, minor enantiomer t_r = 21.45 min.)

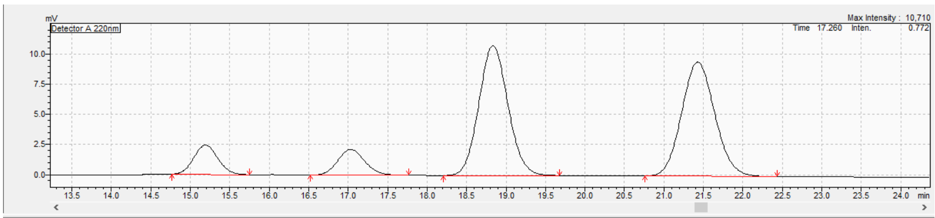

Results View - Peak Table

Peak Table | Compound | Group | Calibration Curve

Peak#	Ret. Time	Area	Height	Mark	Conc.	Unit	ID#	Name	Area%
1	15.191	51316	2427		7.819				7.819
2	17.033	51366	2140		7.827				7.827
3	18.834	276422	10776		42.120				42.120
4	21.428	277167	9474		42.234				42.234
Total		656272	24817		100.000				100.000

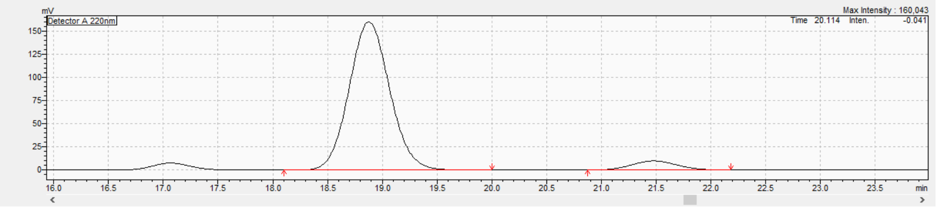

Results View - Peak Table

Peak#	Ret. Time	Area	Height	Mark	Conc.	Unit	ID#	Name	Area%
1	18.878	4122400	160063	M	93.505				93.505
2	21.477	286371	9883	M	6.495				6.495
Total		4408771	169946		100.000				100.000

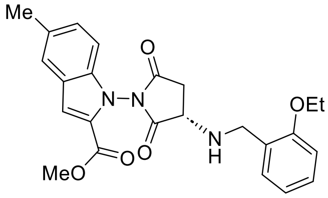

Methyl (*P*,*S*)-1-(3-((2-ethoxybenzyl)amino)-2,5-dioxopyrrolidin-1-yl)-5-methyl-1H-indole-2-carboxylate(5k)

Prepared according to the general procedure B in 54% yield as a colorless oil. **^{1}H NMR** (400 MHz, $CDCl_3$) δ 7.47 (dq, *J* = 2.6, 1.3 Hz, 1H), 7.33 (d, *J* = 1.0 Hz, 1H), 7.30 – 7.27 (m, 2H), 7.18 (dd, *J* = 8.6, 1.6 Hz, 1H), 6.99 (dt, *J* = 8.5, 0.9 Hz, 1H), 6.94 (td, *J* = 7.4, 1.1 Hz, 1H), 6.91 – 6.85 (m, 1H), 4.13 (s, 1H), 4.10 – 4.06 (m, 2H), 4.01 (dd, *J* = 8.4, 5.5 Hz, 1H), 3.92 (d, *J* = 13.7 Hz, 1H), 3.84 (s, 3H), 3.21 (dd, *J* = 18.0, 8.4 Hz, 1H), 2.94 (dd, *J* = 18.1, 5.5 Hz, 1H), 2.43 (s, 3H), 2.24 (s, 1H), 1.45 (t, *J* = 7.0 Hz, 3H). **^{13}C NMR** (100 MHz, $CDCl_3$) δ 174.7, 172.1 160.7, 157.5, 136.5, 132.2, 130.4, 129.3, 129.09, 128.9, 126.8, 124.9, 122.7, 120.6, 111.6, 111.6, 108.6, 63.8, 53.4, 52.1, 47.22, 35.69, 21.46, 15.03. **HRMS-EI (m/z):** Calcd for $[C_{24}H_{26}N_3O_5, M+H]^+$: 436.1867; found: 436.1872. $[\alpha]_D^{25}$ = +10.77 (c = 1.2, $CHCl_3$). **HPLC:** 93% ee. (HPLC condition: Chiralpak AD column, *n*-hexane/*i*-PrOH = 90:10, 1 mL/min, 220 nm, major enantiomer t_r = 25.91 min, minor enantiomer t_r = 27.38 min.)

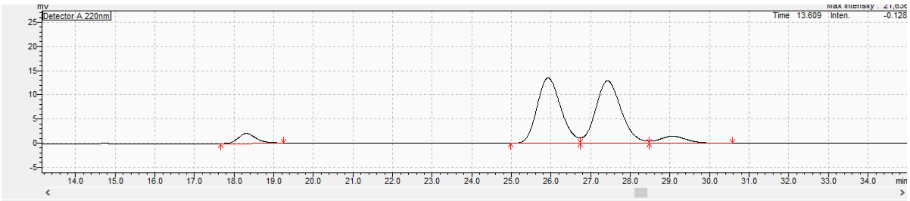

Results View - Peak Table

Peak#	Ret. Time	Area	Height	Mark	Conc.	Unit	ID#	Name	Area%
1	18.308	63036	2039		4.957				4.957
2	25.924	563174	13522		44.283				44.283
3	27.426	577219	12924	V	45.388				45.388
4	29.068	68320	1444	SV	5.372				5.372
Total		1271749	29928		100.000				100.000

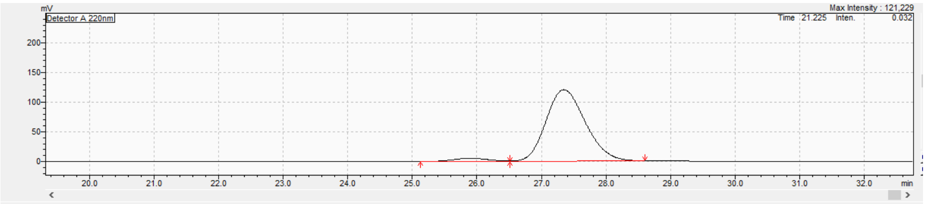

Results View - Peak Table

Peak Table | Compound | Group | Calibration Curve

Peak#	Ret. Time	Area	Height	Mark	Conc.	Unit	ID#	Name	Area%
1	25.903	182729	5056		3.482				3.482
2	27.348	5065552	120285	V	96.518				96.518
Total		5248281	125341		100.000				100.000

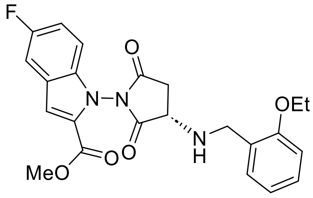

Methyl (*P*,*S*)-1-(3-((2-ethoxybenzyl)amino)-2,5-dioxopyrrolidin-1-yl)-5-fluoro-1H-indole-2-carboxylate(5l)

Prepared according to the general procedure B in 41% yield as a colorless oil. **^{1}H NMR** (400 MHz, $CDCl_3$) δ 7.37 (d, *J* = 0.9 Hz, 1H), 7.34 (d, *J* = 1.8 Hz, 2H), 7.22 (d, *J* = 1.6 Hz, 1H), 7.11 (dd, *J* = 9.0, 2.4 Hz, 1H), 7.06 – 7.02 (m, 1H), 6.98 – 6.88 (m, 2H), 4.09 (dd, *J* = 7.0, 1.0 Hz, 2H), 4.03 – 3.96 (m, 1H), 3.91 (s, 1H), 3.86 (s, 3H), 3.79 (d, *J* = 12.5 Hz, 1H), 3.22 (dd, *J* = 18.1, 8.4 Hz, 1H), 2.95 (dd, *J* = 18.1, 5.5 Hz, 1H), 2.05 (s, 1H), 1.45 (t, *J* = 7.0 Hz, 3H). **^{13}C NMR** (125 MHz, $CDCl_3$) δ 174.6, 172.90, 160.3 (d, J = 28.0 Hz), 157.9 (d, J = 102.9 Hz), 140.0, 134.5, 130.4, 129.1, 126.8, 125.1, 122.2, 120.6, 115.9 (d, J = 26.8 Hz), 113.3, 111.6, 110.1 (d, J = 9.4 Hz), 108.0 (d, J = 24.0 Hz), 63.8, 53.4, 52.3, 47.3, 35.8, 15.0. **HRMS-EI (m/z):** Calcd for [$C_{23}H_{23}FN_3O_5$, M+H]$^+$: 440.1616; found: 440.162. $[\alpha]_D^{25}$ = +3.146 (c = 0.89, $CHCl_3$). **HPLC:** 86% ee. (HPLC condition: Chiralpak IB column, *n*-hexane/*i*-PrOH = 90:10, 1 mL/min, 220 nm, major enantiomer t_r = 14.80 min, minor enantiomer t_r = 18.25 min.)

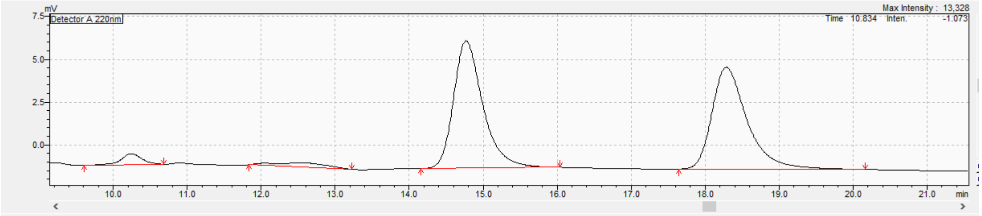

Results View - Peak Table

Peak Table | Compound | Group | Calibration Curve

Peak#	Ret. Time	Area	Height	Mark	Conc.	Unit	ID#	Name	Area%
1	10.244	13125	654		3.065				3.065
2	12.528	12517	267	M	2.923				2.923
3	14.769	205252	7444		47.933				47.933
4	18.287	197312	5956		46.079				46.079
Total		428205	14320		100.000				100.000

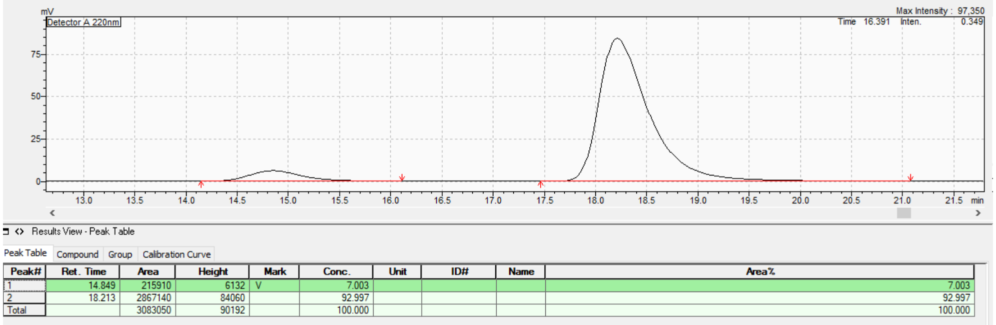

Peak#	Ret. Time	Area	Height	Mark	Conc.	Unit	ID#	Name	Area%
1	14.849	215910	6132	V	7.003				7.003
2	18.213	2867140	84060		92.997				92.997
Total		3083050	90192		100.000				100.000

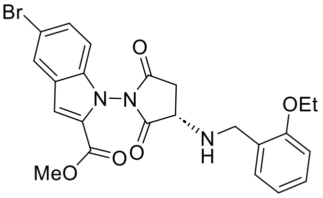

Methyl (*P*,*S*)-5-bromo-1-(3-((2-ethoxybenzyl)amino)-2,5-dioxopyrrolidin-1-yl)-1H-indole-2-carboxylate(5m)

Prepared according to the general procedure B in 38% yield as a colorless oil. **^{1}H NMR** (400 MHz, $CDCl_3$) δ 7.85 (dd, *J* = 1.8, 0.6 Hz, 1H), 7.44 (dd, *J* = 8.8, 1.8 Hz, 1H), 7.34 (d, *J* = 0.9 Hz, 1H), 7.23 – 7.14 (m, 0H), 7.05 – 6.82 (m, 3H), 4.13 – 4.07 (m, 2H), 4.04 – 3.97 (m, 1H), 3.92 (d, *J* = 13.7 Hz, 1H), 3.86 (s, 3H), 3.79 (d, *J* = 10.7 Hz, 1H), 3.22 (dd, *J* = 18.1, 8.4 Hz, 1H), 2.94 (dd, *J* = 18.1, 5.5 Hz, 1H), 2.04 (s, 0H), 1.45 (t, *J* = 7.0 Hz, 3H). **^{13}C NMR** (100 MHz, $CDCl_3$) δ 174.5, 171.8, 160.3, 157.5, 136.6, 130.4, 129.9, 129.2, 126.7, 126.4, 126.2, 125.7, 120.6, 115.8, 111., 110.9, 110.6, 63.8, 53.4, 52.34, 47.3, 35.8, 15.0. **HRMS-EI (m/z):** Calcd for [$C_{23}H_{23}BrN_3O_5$, M+H]$^+$: 500.0816; found: 500.0818. $[\alpha]_D^{25}$ = +5.208 (c = 0.96, $CHCl_3$). **HPLC:** 90% ee. (HPLC condition: Chiralpak AD column, *n*-hexane/*i*-PrOH = 90:10, 1 mL/min, 220 nm, major enantiomer t_r = min, minor enantiomer t_r = min.)

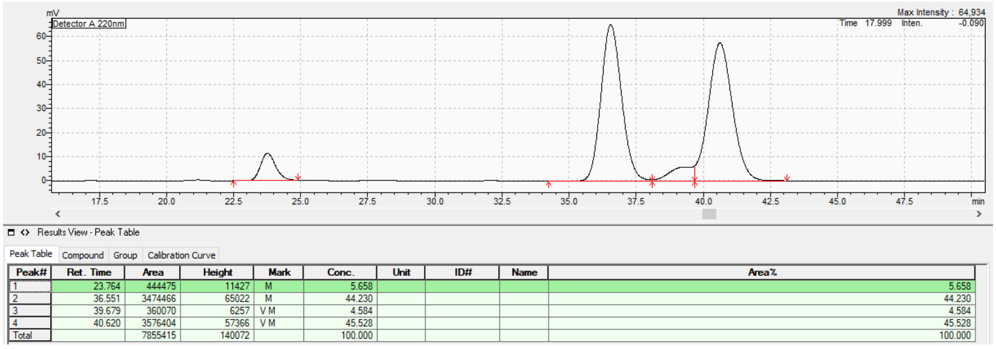

Peak#	Ret. Time	Area	Height	Mark	Conc.	Unit	ID#	Name	Area%
1	23.764	444475	11427	M	5.658				5.658
2	36.551	3474466	65022	M	44.230				44.230
3	39.679	360070	6257	V M	4.584				4.584
4	40.620	3576404	57366	V M	45.528				45.528
Total		7855415	140072		100.000				100.000

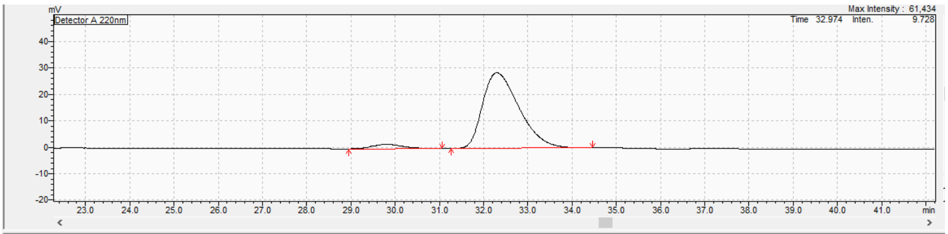

Results View - Peak Table

Peak Table | Compound | Group | Calibration Curve

Peak#	Ret. Time	Area	Height	Mark	Conc.	Unit	ID#	Name	Area%
1	29.802	77985	1634		4.549				4.549
2	32.300	1636455	28529		95.451				95.451
Total		1714441	30163		100.000				100.000

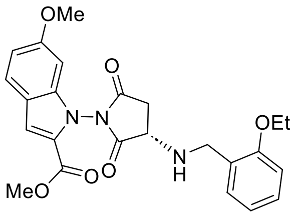

Methyl (*P*,*S*)-1-(3-((2-ethoxybenzyl)amino)-2,5-dioxopyrrolidin-1-yl)-6-methoxy-1H-indole-2-carboxylate(5n)

Prepared according to the general procedure B in 70% yield as a colorless oil. **^{1}H NMR** (400 MHz, $CDCl_3$) δ 7.57 (dd, *J* = 8.8, 0.5 Hz, 1H), 7.36 (d, *J* = 0.9 Hz, 1H), 7.33 – 7.30 (m, 1H), 7.26 (s, 1H), 6.95 (ddd, *J* = 21.5, 7.8, 1.1 Hz, 2H), 6.90 (dd, *J* = 8.8, 2.2 Hz, 1H), 6.63 (d, *J* = 2.2 Hz, 1H), 4.13 (d, *J* = 7.0 Hz, 2H), 4.11 (d, *J* = 3.6 Hz, 1H), 4.09 – 4.02 (m, 1H), 3.91 (d, *J* = 13.3 Hz, 1H), 3.84 (s, 3H), 3.81 (s, 3H), 3.22 (dd, *J* = 17.9, 8.5 Hz, 1H), 2.94 (dd, *J* = 17.8, 4.9 Hz, 1H), 2.19 (s, 1H), 1.49 (t, *J* = 7.0 Hz, 3H). **^{13}C NMR** (100 MHz, $CDCl_3$) δ 174.4, 172.0, 160.9, 160.5, 157.4, 139.6, 130.5, 129.4, 126.2, 124.2, 120.7, 118.7, 114.1, 113.4, 112.5, 111.6, 91.4, 63.8, 55.9, 54.0, 51.8, 47.7, 34.9, 15.0. **HRMS-EI (m/z):** Calcd for $[C_{24}H_{26}N_3O_6, M+H]^+$: 452.1816; found: 452.1815. $[\alpha]_D^{25}$ = - 24.744(c = 1.56, $CHCl_3$). **HPLC:** 98% ee. (HPLC condition: Chiralpak AD column, *n*-hexane/*i*-PrOH = 90:10, 1 mL/min, 220 nm, major enantiomer t_r = 16.36 min, minor enantiomer t_r = 20.79 min.)

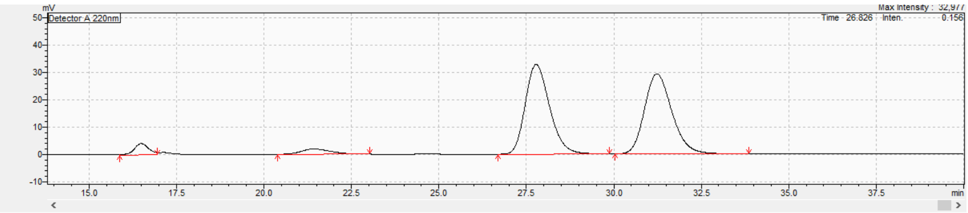

Results View - Peak Table

Peak Table | Compound | Group | Calibration Curve

Peak#	Ret. Time	Area	Height	Mark	Conc.	Unit	ID#	Name	Area%
1	16.492	118184	4077	M	3.467				3.467
2	21.430	112405	1921		3.298				3.298
3	27.781	1584692	32810		46.490				46.490
4	31.231	1593366	29282	S	46.745				46.745
Total		3408648	68091		100.000				100.000

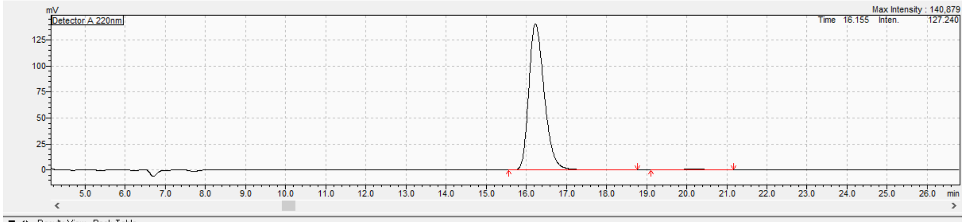

Results View - Peak Table

Peak Table | Compound | Group | Calibration Curve

Peak#	Ret. Time	Area	Height	Mark	Conc.	Unit	ID#	Name	Area%
1	16.229	3777251	140912	S	98.983				98.983
2	20.158	38807	956		1.017				1.017
Total		3816059	141868		100.000				100.000

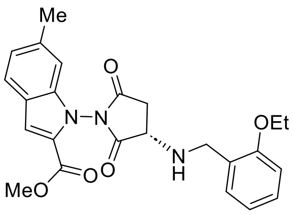

Methyl (*P*,*S*)-1-(3-((2-ethoxybenzyl)amino)-2,5-dioxopyrrolidin-1-yl)-6-methyl-1H-indole-2-carboxylate(5o)

Prepared according to the general procedure B in 60% yield as a colorless oil. **^{1}H NMR** (400 MHz, $CDCl_3$) δ 7.57 (d, *J* = 8.2 Hz, 1H), 7.37 (d, *J* = 1.0 Hz, 1H), 7.28 (d, *J* = 1.6 Hz, 2H), 7.08 – 7.03 (m, 1H), 6.94 (td, *J* = 7.4, 1.1 Hz, 1H), 6.91 – 6.87 (m, 2H), 4.13 (d, *J* = 11.5 Hz, 1H), 4.11 – 4.07 (m, 2H), 4.01 (dd, *J* = 8.4, 5.5 Hz, 1H), 3.92 (d, *J* = 13.7 Hz, 1H), 3.84 (s, 3H), 3.22 (dd, *J* = 18.1, 8.4 Hz, 1H), 2.95 (dd, *J* = 18.1, 5.5 Hz, 1H), 2.42 (s, 3H), 2.05 (s, 1H), 1.46 (t, *J* = 6.9 Hz, 3H). **^{13}C NMR** (100 MHz, $CDCl_3$) δ 174.7, 172.1, 160.8, 157.5, 138.5, 137.7, 130.4, 129.3, 129.1, 126.9, 124.7, 123.0, 122.6, 120.6, 112.0, 111.6, 108.7, 63.8, 53.4, 52.1, 47.3, 35.7, 22.2, 15.00. **HRMS-EI (m/z):** Calcd for $[C_{24}H_{26}N_3O_5, M+H]^+$: 436.1867; found: 436.187.

$[\alpha]_D^{25}$ = +17.46 (c = 1.3 , $CHCl_3$). **HPLC:** 92% ee. (HPLC condition: Chiralpak AD column, *n*-hexane/*i*-PrOH = 95:5, 1 mL/min, 220 nm, major enantiomer t_r = 37.28 min, minor enantiomer t_r = 39.30 min.)

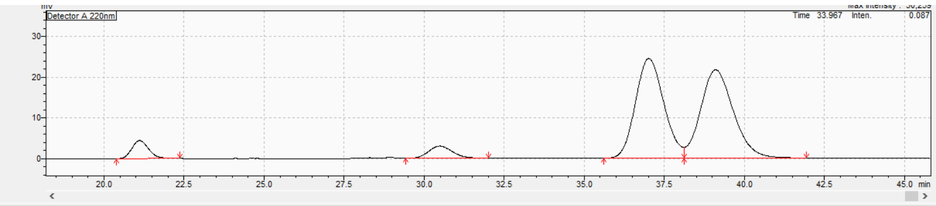

Results View - Peak Table

Peak Table | Compound | Group | Calibration Curve

Peak#	Ret. Time	Area	Height	Mark	Conc.	Unit	ID#	Name	Area%
1	21.107	161336	4442		4.778				4.778
2	30.490	158467	3023		4.693				4.693
3	37.019	1514451	24530		44.853				44.853
4	39.104	1542234	21724	V	45.676				45.676
Total		3376488	53720		100.000				100.000

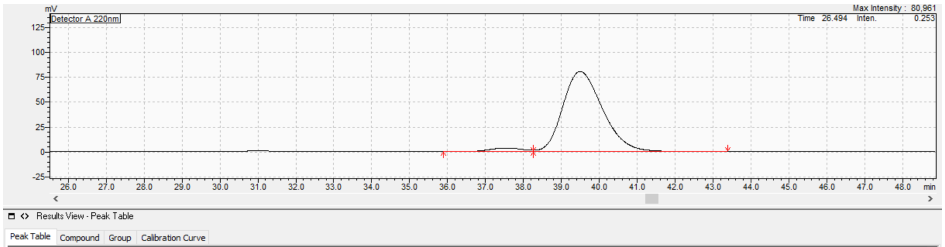

Peak#	Ret. Time	Area	Height	Mark	Conc.	Unit	ID#	Name	Area%
1	37.536	224083	3709		3.680				3.680
2	39.495	5865162	80556	V	96.320				96.320
Total		6089244	84265		100.000				100.000

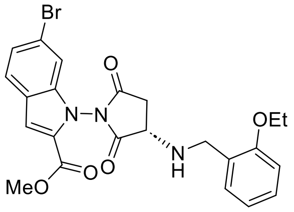

Methyl (*P*,*S*)-6-bromo-1-(3-((2-ethoxybenzyl)amino)-2,5-dioxopyrrolidin-1-yl)-1H-indole-2-carboxylate(5p)

Prepared according to the general procedure B in 77% yield as a colorless oil. **^{1}H NMR** (400 MHz, $CDCl_3$) δ 7.56 (d, *J* = 8.5 Hz, 1H), 7.36 (dd, *J* = 7.2, 0.9 Hz, 1H), 7.33 (dd, *J* = 8.4, 1.7 Hz, 1H), 7.30 – 7.27 (m, 3H), 6.95 (td, *J* = 7.5, 1.2 Hz, 1H), 6.90 (dd, *J* = 8.1, 1.1 Hz, 1H), 4.13 (d, *J* = 7.4 Hz, 1H), 4.11 – 4.09 (m, 2H), 4.03 (dd, *J* = 8.4, 5.5 Hz, 1H), 3.92 (d, *J* = 13.7 Hz, 1H), 3.85 (s, 3H), 3.23 (dd, *J* = 18.1, 8.4 Hz, 1H), 2.95 (dd, *J* = 18.1, 5.5 Hz, 1H), 2.23 (s, 1H), 1.46 (t, *J* = 7.0 Hz, 3H).. **^{13}C NMR** (100 MHz, $CDCl_3$) δ 174.5, 171.8, 160.4, 157.4, 138.6, 130.4, 129.2, 126.7, 126.4, 126.0, 124.6, 123.5, 121.0, 120.6, 112.2, 111.8, 111.6, 63.8, 53.4, 52.3, 47.3, 35.7, 15.0. **HRMS-EI (m/z):** Calcd for $[C_{23}H_{23}BrN_3O_5, M+H]^+$: 500.0816; found: 500.0816. $[\alpha]_D^{25}$ = +15.65 (c = 1.93, $CHCl_3$). **HPLC:** 94% ee. (HPLC condition: Chiralpak AD column, *n*-hexane/*i*-PrOH = 90:10, 1 mL/min, 220 nm, major enantiomer t_r = 21.22 min, minor enantiomer t_r = 27.15 min.)

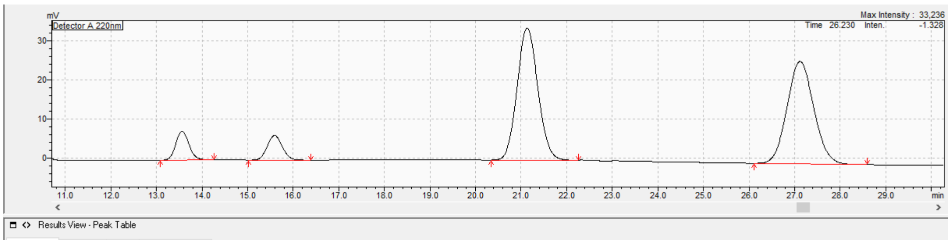

Peak#	Ret. Time	Area	Height	Mark	Conc.	Unit	ID#	Name	Area%
1	13.563	147942	7312		6.066				6.066
2	15.592	145848	6338		5.980				5.980
3	21.133	1074176	33816		44.043				44.043
4	27.118	1070960	26201		43.911				43.911
Total		2438926	73667		100.000				100.000

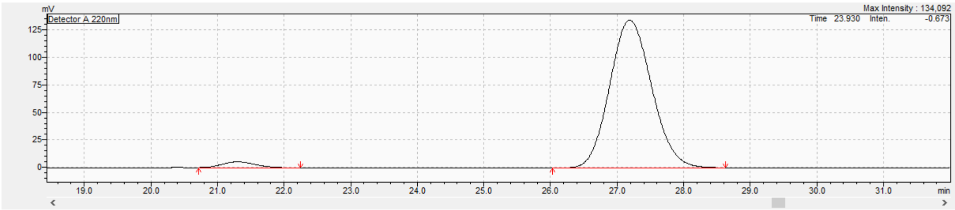

Results View - Peak Table

Peak Table | Compound | Group | Calibration Curve

Peak#	Ret. Time	Area	Height	Mark	Conc.	Unit	ID#	Name	Area%
1	21.297	185580	5343	M	3.046				3.046
2	27.190	5907653	134667	M	96.954				96.954
Total		6093233	140010		100.000				100.000

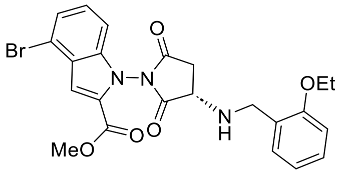

Methyl (*P*,*S*)-4-bromo-1-(3-((2-ethoxybenzyl)amino)-2,5-dioxopyrrolidin-1-yl)-1H-indole-2-carboxylate(5q)

Prepared according to the general procedure B in 34% yield as a colorless oil. **^{1}H NMR** (400 MHz, $CDCl_3$) δ 7.47 (d, *J* = 0.7 Hz, 1H), 7.41 (dd, *J* = 7.6, 0.7 Hz, 1H), 7.24 – 7.17 (m, 2H), 7.07 – 7.04 (m, 1H), 6.97 – 6.88 (m, 2H), 4.19 – 4.05 (m, 3H), 4.02 (dd, *J* = 8.3, 5.5 Hz, 1H), 3.93 (d, *J* = 13.7 Hz, 1H), 3.88 (s, 3H), 3.23 (dd, *J* = 18.1, 8.4 Hz, 1H), 2.96 (dd, *J* = 18.1, 5.5 Hz, 1H), 2.05 (s, 1H), 1.45 (t, *J* = 6.9 Hz, 3H). **^{13}C NMR** (100 MHz, $CDCl_3$) δ 174.4, 171.7, 130.4, 129.2, 127.8, 126.8, 125.7, 120.6, 111.8, 111.6, 108.2, 63.8, 53.4, 52.4, 47.3, 35.8, 15.0. **HRMS-EI (m/z):** Calcd for $[C_{23}H_{23}BrN_3O_5, M+H]^+$: 500.0816; found: 500.0817. $[\alpha]_D^{25}$ = +6.96 (c = 0.56, $CHCl_3$). **HPLC:** 90% ee. (HPLC condition: Chiralpak AD column, *n*-hexane/*i*-PrOH = 90:10, 1 mL/min, 220 nm, major enantiomer t_r = 19.35 min, minor enantiomer t_r = 20.86 min.)

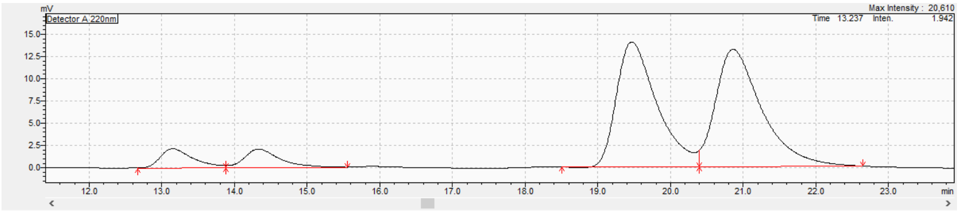

Results View - Peak Table

Peak Table | Compound | Group | Calibration Curve

Peak#	Ret. Time	Area	Height	Mark	Conc.	Unit	ID#	Name	Area%
1	13.151	69077	2185	V	5.460				5.460
2	14.332	70951	2092	V	5.608				5.608
3	19.468	544891	14035	M	43.067				43.067
4	20.863	580290	13188	V M	45.865				45.865
Total		1265209	31500		100.000				100.000

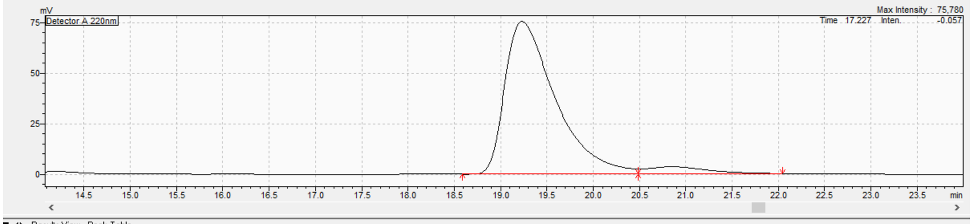

Results View - Peak Table

Peak Table | Compound | Group | Calibration Curve

Peak#	Ret. Time	Area	Height	Mark	Conc.	Unit	ID#	Name	Area%
1	19.232	2870265	75695	M	94.799				94.799
2	20.855	157474	3493	V M	5.201				5.201
Total		3027740	79188		100.000				100.000

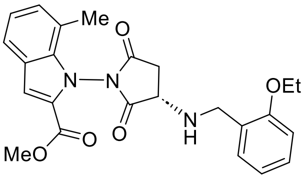

Methyl (*P*,*S*)-1-(3-((2-ethoxybenzyl)amino)-2,5-dioxopyrrolidin-1-yl)-7-methyl-1H-indole-2-carboxylate(5r)

Prepared according to the general procedure B in 61% yield as a colorless oil. **^{1}H NMR** (500 MHz, $CDCl_3$) δ 7.59 – 7.51 (m, 1H), 7.42 (s, 1H), 7.28 (d, *J* = 2.1 Hz, 1H), 7.14 – 7.06 (m, 2H), 6.95 – 6.92 (m, 1H), 6.90 (dd, *J* = 8.6, 1.1 Hz, 1H), 4.12 (d, *J* = 1.5 Hz, 1H), 4.10 – 4.08 (m, 2H), 3.99 – 3.93 (m, 1H), 3.84 (s, 3H), 3.18 (dd, *J* = 18.1, 8.5 Hz, 1H), 2.94 (dd, *J* = 18.1, 5.5 Hz, 1H), 2.33 (d, *J* = 0.7 Hz, 3H), 2.05 (s, 0H), 1.45 (t, *J* = 6.9 Hz, 3H). **^{13}C NMR** (126 MHz, $CDCl_3$) δ 175.2, 172.7, 160.7, 157.4, 136.9, 130.3, 129.3, 129.1, 127.0, 125.4, 125.3, 122.6, 121.4, 120.7, 120.6, 112.8, 111.6, 63.8, 53.5, 52.1, 47.3, 35.6, 17.3, 15.0. **HRMS-EI (m/z):** Calcd for [$C_{24}H_{26}N_3O_5$, M+H]$^+$: 436.1867; found: 436.1869. $[\alpha]_D^{25}$ = +9.36 (c = 1.4, $CHCl_3$). **HPLC:** 90% ee. (HPLC condition: Chiralpak AD column, *n*-hexane/*i*-PrOH = 90:10, 1 mL/min, 220 nm, major enantiomer t_r = 16.93 min, minor enantiomer t_r = 19.47 min.)

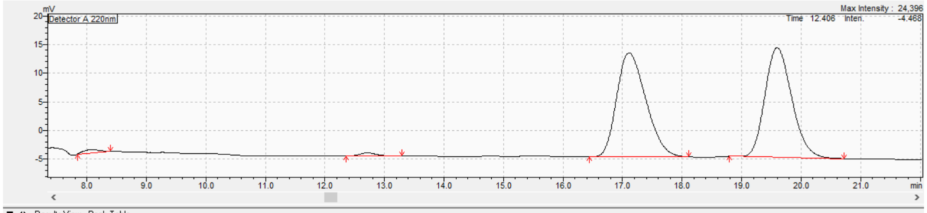

Results View - Peak Table

Peak Table | Compound | Group | Calibration Curve

Peak#	Ret. Time	Area	Height	Mark	Conc.	Unit	ID#	Name	Area%
1	8.090	10751	551	M	0.864				0.864
2	12.704	10961	550		0.881				0.881
3	17.109	620356	18183	M	49.882				49.882
4	19.597	601581	19149	M	48.372				48.372
Total		1243649	38433		100.000				100.000

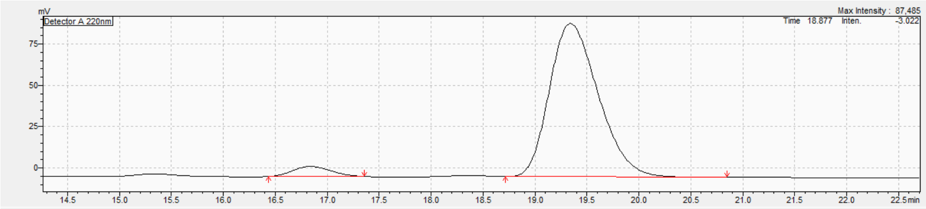

Results View - Peak Table

Peak Table | Compound | Group | Calibration Curve

Peak#	Ret. Time	Area	Height	Mark	Conc.	Unit	ID#	Name	Area%
1	16.838	155102	6077	M	4.858				4.858
2	19.341	3037388	92841		95.142				95.142
Total		3192491	98918		100.000				100.000

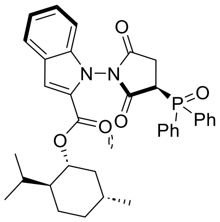

(*P*,1R,2S,5R)-2-Isopropyl-5-methylcyclohexyl 1-((R)-3-(diphenylphosphoryl)-2,5-dioxopyrrolidin-1-yl)-1H-indole-2-carboxylate (3ac)

^{1}H NMR (400 MHz, $CDCl_3$) δ 8.00 (m, 2H), 7.89 – 7.80 (m, 2H), 7.67 – 7.55 (m, 5H), 7.48 (m, 2H), 7.37 (d, *J* = 0.8 Hz, 1H), 7.33 – 7.27 (m, 1H), 7.21 – 7.16 (m, 1H), 6.89 – 6.78 (m, 1H), 4.79 (td, *J* = 10.9, 4.4 Hz, 1H), 4.21 (m, 1H), 3.54 – 3.38 (m, 1H), 3.27 (m, 1H), 2.05 (d, *J* = 12.0 Hz, 1H), 1.95 – 1.90 (m, 1H), 1.76 – 1.67 (m, 2H), 1.54 – 1.45 (m, 2H), 1.12 (d, *J* = 12.6 Hz, 2H), 0.93 – 0.87 (m, 7H), 0.75 (d, *J* = 6.9 Hz, 3H). **^{13}C NMR** (125 MHz, $CDCl_3$) δ 171.9 (d, J = 3.4 Hz), 169.4 (d, J = 4.3 Hz), 160.1, 138.0, 133.0 (d, J = 15.1 Hz), 131.9 (d, J = 9.9 Hz), 131.4 (d, J = 9.8 Hz), 130.6, 129.8, 129.3 (d, J = 12.4 Hz), 129.0 (d, J = 12.5 Hz), 127.0, 125.6, 124.4, 122.8 (d, J = 28.4 Hz), 111.7, 109.5, 75.3, 47.3, 41.5, 40.9, 34.3, 31.6, 28.5, 26.6, 23.8, 22.1, 20.8, 16.7. **^{31}P NMR:** (162 MHz, $CDCl_3$) δ 30.31. **HRMS (ESI):** m/z Calcd for $[C_{23}H_{27}N_2O_4P, M+H]^+$: 597.2513 ; Found: 597.2515.

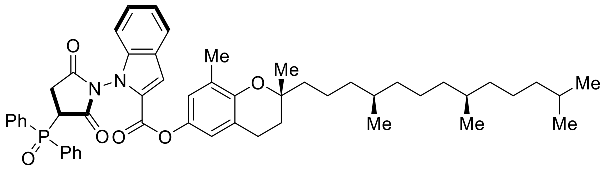

5

(*P*,*R*)-2,8-Dimethyl-2-((4R,8R)-4,8,12-trimethyltridecyl)chroman-6-yl 1-((S)-3-(diphenylphosphoryl)-2,5-dioxopyrrolidin-1-yl)-1H-indole-2-carboxylate (3ad)

^{1}H NMR (400 MHz, $CDCl_3$) δ 7.97 (dd, *J* = 11.4, 7.7 Hz, 2H), 7.84 (dd, *J* = 11.5, 7.9 Hz, 2H), 7.70 (d, *J* = 8.0 Hz, 1H), 7.64 – 7.55 (m, 5H), 7.53 – 7.48 (m, 2H), 7.38 – 7.33 (m, 1H), 7.23 (d, *J* = 7.8 Hz, 1H), 6.89 (d, *J* = 8.3 Hz, 1H), 6.75 (d, *J* = 2.4 Hz, 1H), 6.69 (d, *J* = 2.5 Hz, 1H), 4.21 – 4.13 (m, 1H), 3.46 – 3.33 (m, 1H), 3.27 – 3.13 (m, 1H), 2.73 (s, 2H), 2.16 (s, 3H), 1.76 (m, 2H), 1.60 – 1.51 (m, 3H), 1.36 (d, *J* = 46.5 Hz, 11H), 1.25 – 1.01 (m, 10H), 0.91 – 0.84 (m, 14H). **^{13}C NMR** (125 MHz, $CDCl_3$) δ 171.0, 169.4, 159.7, 150.3, 141.7, 138.4, 133.1, 132.9, 131.9 (d, *J* = 9.5 Hz), 131.3 (d, *J* = 9.4 Hz), 130.6, 129.8, 129.4, 129.3, 129.1, 129.0, 127.6 (d, *J* = 6.8 Hz), 124.6, 124.4, 123.1, 122.9, 121.3 (d, *J* = 4.7 Hz), 119.3, 113.1, 109.6, 41.2 (d, *J* = 60.3 Hz), 40.3, 39.5, 37.5, 37.4, 32.9, 32.8, 31.0, 28.5, 28.1, 24.9, 24.6, 24.4, 22.8 (d, *J* = 11.6 Hz), 22.5, 21.1, 19.9, 19.8, 16.3. **^{31}P NMR** (162 MHz, $CDCl_3$) δ 30.35. **HRMS (ESI):** m/z Calcd for $[C_{23}H_{27}N_2O_4P, M+H]^+$: 843.4497; Found: 843.4495.

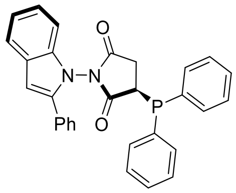

(*P*,*R*)-3-(Diphenylphosphaneyl)-1-(2-phenyl-1H-indol-1-yl)pyrrolidine-2,5-dione (6)

^{1}H NMR (500 MHz, $CDCl_3$) δ 7.59 (t, *J* = 7.4 Hz, 2H), 7.53 – 7.28 (m, 14H), 7.25 – 7.11 (m, 2H), 6.71 (s, 1H), 6.48 (d, *J* = 7.7 Hz, 1H), 3.73 (dt, *J* = 9.4, 3.2 Hz, 1H), 3.07 – 2.93 (m, 1H), 2.81 (ddd, *J* = 19.0, 11.8, 3.8 Hz, 1H). **^{13}C NMR** (125 MHz, $CDCl_3$) δ 173.1 (d, *J* = 7.4 Hz), 171.9 (d, *J* = 2.0 Hz), 141.1, 137.3, 134.3, 134.1, 133.5, 133.3, 130.6 130.4, 129.9, 129.3, 129.3, 129.0, 128.9, 128.7, 128.3, 127.3, 123.63, 122.2, 121.3, 109.0, 103.8, 36.8 (d, *J* = 27.5 Hz), 31. (d, *J* = 13.3 Hz). **^{31}P NMR** (162 MHz, $CDCl_3$) δ 1.71. **HRMS (ESI):** m/z Calcd for $[C_{30}H_{23}N_2O_2P, M+H]^+$: 475.1570; Found: 475.1558. $[\alpha]_D^{25}$ = +39.70 (c = 2.3, $CHCl_3$). **HPLC:** 99% ee. (HPLC condition: Chiralpak AD column, *n*-hexane/*i*-PrOH = 70:30, 1 mL/min, 220 nm, major enantiomer t_r = 9.0 min, minor enantiomer t_r = 14.0 min.)

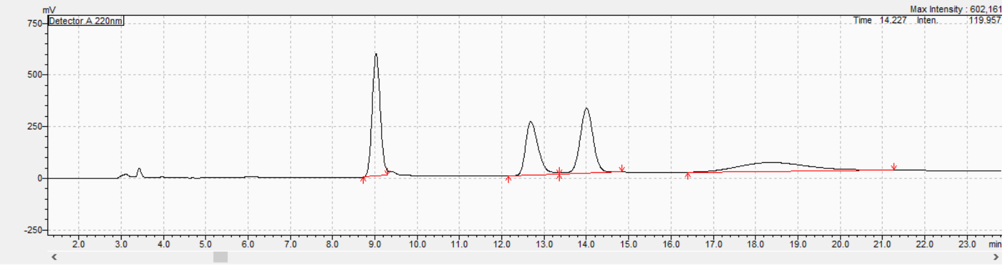

Results View - Peak Table

Peak Table | Compound | Group | Calibration Curve

Peak#	Ret. Time	Area	Height	Mark	Conc.	Unit	ID#	Area%
1	9.028	7767282	589501	M	31.101			31.101
2	12.684	5349372	257768	M	21.420			21.420
3	14.005	6808187	315672	V M	27.261			27.261
4	18.366	5049318	42893	M	20.218			20.218
Total		24974158	1205834		100.000			100.000

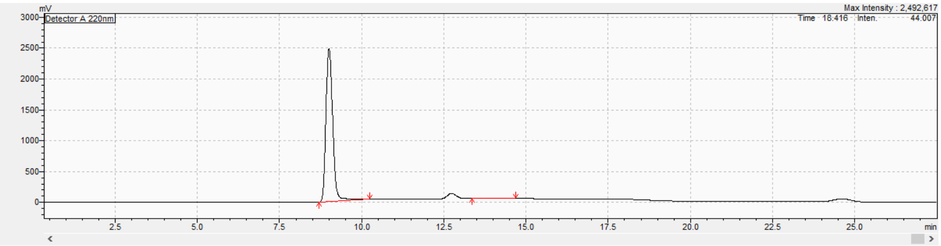

Results View - Peak Table

Peak Table | Compound | Group | Calibration Curve

Peak#	Ret. Time	Area	Height	Mark	Conc.	Unit	ID#	Area%
1	9.006	35942002	2480304		99.440			99.440
2	13.993	202453	9812	M	0.560			0.560
Total		36144455	2490116		100.000			100.000

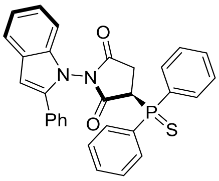

(*P*,*R*)-3-(Diphenylphosphorothioyl)-1-(2-phenyl-1H-indol-1-yl)pyrrolidine-2,5-dione(7)

^{1}H NMR (500 MHz, CDCl$_3$) δ 8.05 – 7.87 (m, 3H), 7.62-7.49 (m, 6H), 7.48 – 7.43 (m, 3H), 7.43-7.35 (m, 4H), 7.21 (dd, *J* = 6.8, 1.0 Hz, 2H), 6.69 (d, *J* = 0.9 Hz, 1H), 4.28 – 3.90 (m, 11H), 3.41-2.94 (m, 1H), 2.93-2.50 (m, 1H). **^{13}C NMR** (125 MHz, CDCl$_3$) δ 169.8 (d, *J* = 262.3 Hz), 164.6 (d, *J* = 18.0 Hz),140.6, 138.2, 137.5, 132.5, 132.3, 132.2, 131.5, 131.4, 129.2, 129.1, 128.8, 128.7, 128.5, 128.2, 123.9, 122.2, 120.9, 110.5, 104.0, 41.3 (d, *J* = 48.4 Hz), 29.3. **^{31}P NMR** (162 MHz, CDCl$_3$) δ 48.03. **HRMS (ESI):** m/z Calcd for [$C_{30}H_{24}N_2O_2PS$, M+H]$^+$: 475.1570; Found: 475.1558. $[\alpha]_D^{25}$ = -1.429 (c = 0.7, CHCl$_3$). **HPLC:** 99% ee. (HPLC condition: Chiralpak AD column, *n*-hexane/*i*-PrOH = 70:30, 1 mL/min, 220 nm, major enantiomer t_r = 7. min, minor enantiomer t_r = 19,5 min.)

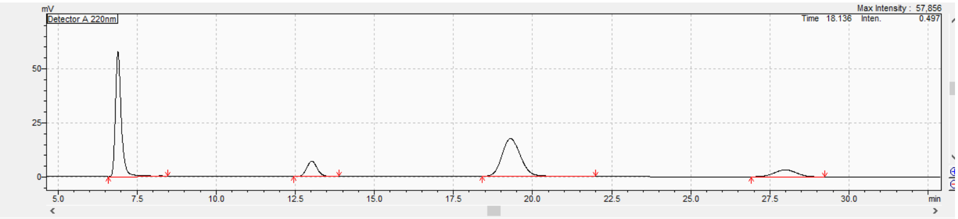

Results View - Peak Table

Peak Table | Compound | Group | Calibration Curve

Peak#	Ret. Time	Area	Height	Mark	Conc.	Unit	ID#	Name	Area%
1	6.901	740544	57633	S	41.584				41.584
2	13.021	162684	7129		9.135				9.135
3	19.304	717382	17473		40.284				40.284
4	27.992	160220	3231		8.997				8.997
Total		1780830	85467		100.000				100.000

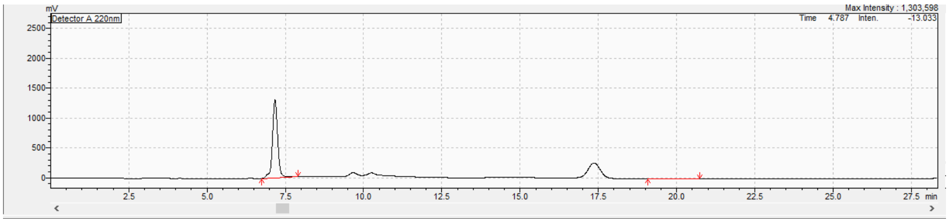

Results View - Peak Table

Peak Table | Compound | Group | Calibration Curve

Peak#	Ret. Time	Area	Height	Mark	Conc.	Unit	ID#	Name	Area%
1	7.174	15366952	1305396		99.598				99.598
2	19.852	62024	1586		0.402				0.402
Total		15428976	1306982		100.000				100.000

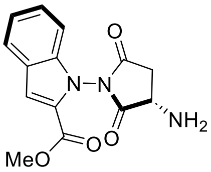

(*P*,*R*)- Methyl (*S*)-1-(3-amino-2,5-dioxopyrrolidin-1-yl)-1H-indole-2-carboxylate(8)

^{1}H NMR (400 MHz, $CDCl_3$) δ 7.62 (dt, *J* = 8.1, 1.0 Hz, 1H), 7.35-7.32 (m, 1H), 7.31-7.24 (m, 2H), 7.07-7.05 (m, 1H), 4.03 (dd, *J* = 9.0, 5.0 Hz, 1H), 3.76 (s, 3H), 3.31 (dd, *J* = 18.6, 9.0 Hz, 1H), 2.69 (dd, *J* = 18.6, 5.0 Hz, 1H), 1.66 (s, 2H). **HRMS (EI):** m/z Calcd for [$C_{14}H_{14}N_3O_4$, M+H]$^+$: 288.0979; Found: 288.0981. **HPLC:** 99% ee. (HPLC condition: Chiralpak AD column, *n*-hexane/*i*-PrOH = 90:10, 1 mL/min, 220 nm, major enantiomer t_r = 19.5 min, minor enantiomer t_r = 22.9 min.)

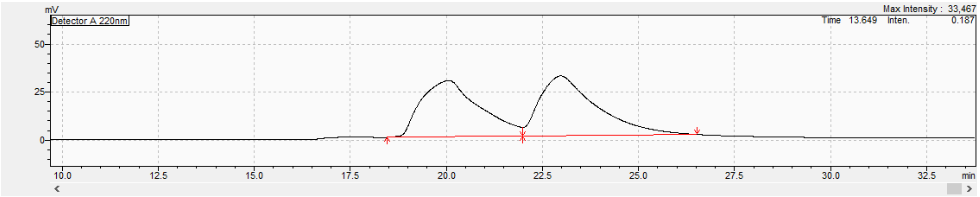

Results View - Peak Table

Peak Table | Compound | Group | Calibration Curve

Peak#	Ret. Time	Area	Height	Mark	Conc.	Unit	ID#	Name	Area%
1	20.055	3106404	29256	M	49.030				49.030
2	22.969	3229348	31172	V M	50.970				50.970
Total		6335752	60428		100.000				100.000

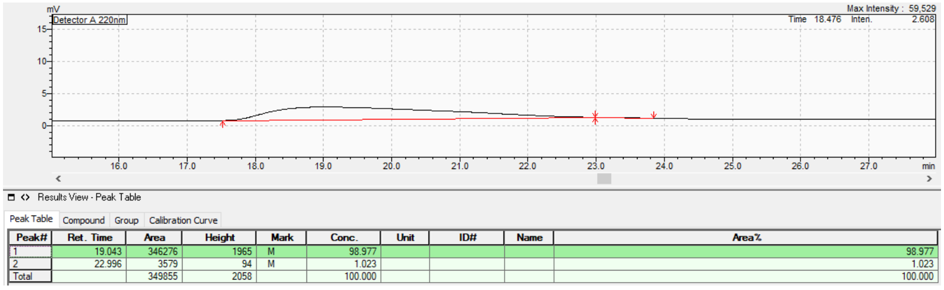

Peak#	Ret. Time	Area	Height	Mark	Conc.	Unit	ID#	Name	Area%
1	19.043	346276	1965	M	98.977				98.977
2	22.996	3579	94	M	1.023				1.023
Total		349855	2058		100.000				100.000

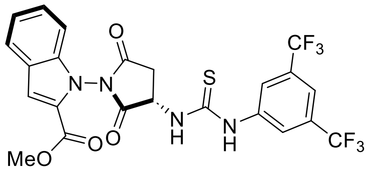

(*P*,*R*)- Methyl (*S*)-1-(3-(3-(3,5-bis(trifluoromethyl)phenyl)thioureido)-2,5-dioxopyrrolidin-1-yl)-1H-indole-2-carboxylate(9)

^{1}H NMR (400 MHz, $CDCl_3$) δ 9.19 (s, 1H), 8.73 (s, 1H), 7.93 (s, 1H), 7.89 – 7.88 (m, 2H), 7.79 (d, *J* = 23.9 Hz, 1H), 7.63 (d, *J* = 7.8 Hz, 1H), 7.48 – 7.40 (m, 1H), 7.39 – 7.34 (m, 2H), 7.21 – 7.16 (m, 1H), 4.76 (d, *J* = 9.2 Hz, 1H), 3.82 (s, 3H), 3.25 (d, *J* = 15.4 Hz, 1H), 2.93 (dd, *J* = 15.6, 9.6 Hz, 1H). **^{13}C NMR** (101 MHz, $CDCl_3$) δ 182.3, 172.2, 169.8, 162.0, 139.1, 134.1, 132.5 (d, *J* = 34.0 Hz), 129.0, 127.0, 125.8, 123.8, 123.5, 123.1, 122.4, 121.5, 111.5, 109.7, 56.7, 52.4, 35.7. **^{19}F NMR** (377 MHz, $CDCl_3$) δ -62.84. **HRMS (EI):** m/z Calcd for [$C_{23}H_{16}F_6N_4O_4S$, M+H]$^+$: 559.0869; Found: 559.0872. **HPLC:** 92% ee. (HPLC condition: Chiralpak AD column, *n*-hexane/*i*-PrOH = 90:10, 1 mL/min, 220 nm, major enantiomer t_r = 7.5 min, minor enantiomer t_r = 11.7 min.)

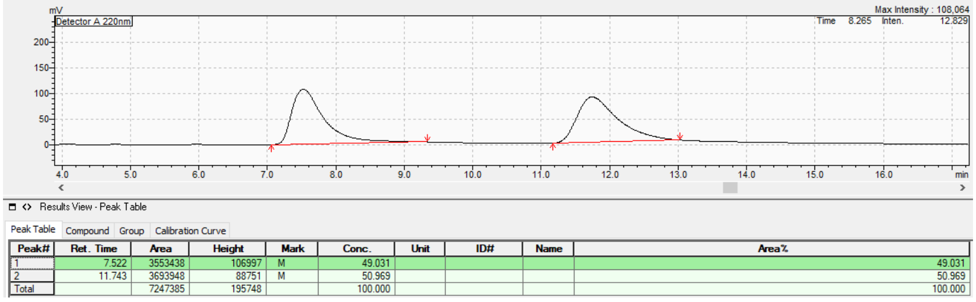

Peak#	Ret. Time	Area	Height	Mark	Conc.	Unit	ID#	Name	Area%
1	7.522	3553438	106997	M	49.031				49.031
2	11.743	3693948	88751	M	50.969				50.969
Total		7247385	195748		100.000				100.000

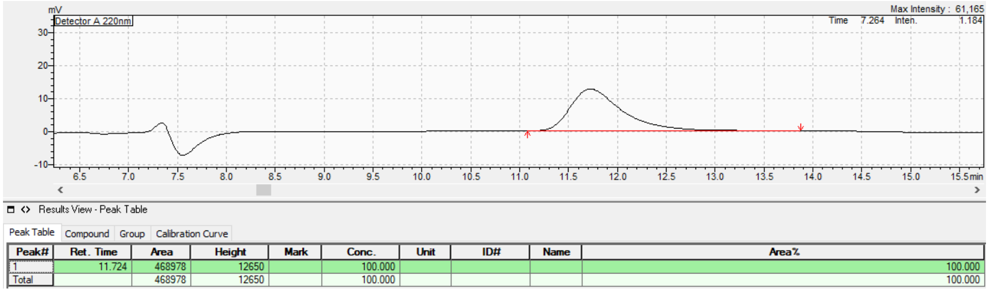

References

[1] Turnu, F.; lberto, A. L.; Cocco, A.; Porcu, S.; Frongia, A.; Sarais, G.; Secci, F. *Org. Lett*. **2019**, *21*, 7329 - 7332.

[2] Chen, K. -W.; Chen, Z. -H.; Yang, S.; Wu, S. -F.; Zhang, Y. -C.; Shi, F. *Angew. Chem. Int. Ed.* **2022**, *61*, e202116829.

[3] Mandal, R.; Emayavaramban, B.; Sundararaju, B. *Org. Lett*. **2018**, *20*, 2835 - 2838. Gu, X. -W.; Sun, Y. -L.; Xie, J. -L.; Wang, X. -B.; Xu, Z.; Yin, G. -W.; Li, L.; Yang, K. -F.; Xu, L. -W. *Nat. Commum*. **2020**, *11*,1-9.

X-Ray Data

Single Crystal Structure X-ray Analysis of 3p

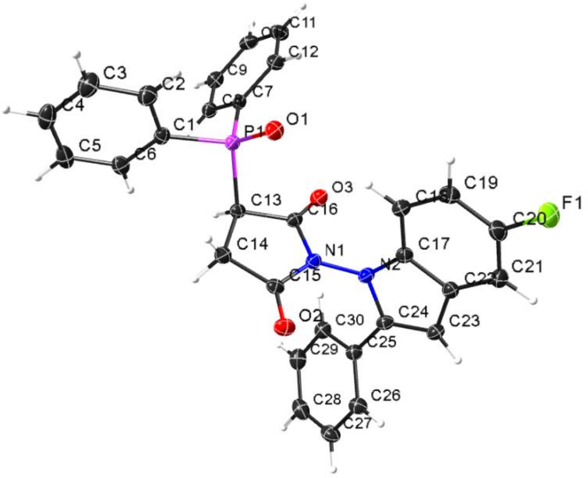

CCDC 2243302

Table 1. Sample and crystal data for M263.

Identification code	M263
Chemical formula	$C_{34}H_{30}FN_2O_5P$

Formula weight	596.57 g/mol
Temperature	100(2) K
Wavelength	0.71073 Å
Crystal size	0.138 x 0.162 x 0.209 mm
Crystal habit	colorless block
Crystal system	monoclinic
Space group	P 1 21 1
Unit cell dimensions	a = 9.0878(4) Å α = 90°
	b = 14.2865(8) Å β = 95.771(2)°
	c = 11.2346(6) Å γ = 90°
Volume	1451.23(13) Å3
Z	2
Density (calculated)	1.365 g/cm^3
Absorption coefficient	0.148 mm^{-1}
F(000)	624

Table 2. Data collection and structure refinement for M263.

Theta range for data collection	3.04 to 30.54°
Index ranges	-12<=h<=11, -20<=k<=20, -16<=l<=16
Reflections collected	47818
Independent reflections	8831 [R(int) = 0.0661]
Coverage of independent reflections	99.8%
Absorption correction	Multi-Scan
Structure solution technique	direct methods
Structure solution program	XT, VERSION 2018/2
Refinement method	Full-matrix least-squares on F^2
Refinement program	SHELXL-2019/1 (Sheldrick, 2019)
Function minimized	Σ w(Fo^2 - Fc^2)^2
Data / restraints / parameters	8831 / 1 / 390
Goodness-of-fit on F2	1.101

Final R indices	7917 data; I>2σ(I) R1 = 0.0395, wR^2 = 0.0880
	all data R1 = 0.0477, wR^2 = 0.0966
Weighting scheme	$w=1/[\sigma2(Fo^2)+(0.0366P)^2+0.3887P]$
	where $P=(Fo^2+2Fc^2)/3$
Absolute structure parameter	0.00(4)
Largest diff. peak and hole	0.461 and -0.244 $eÅ^{-3}$
R.M.S. deviation from mean	0.050 $eÅ^{-3}$

Single Crystal Structure X-ray Analysis of 5e

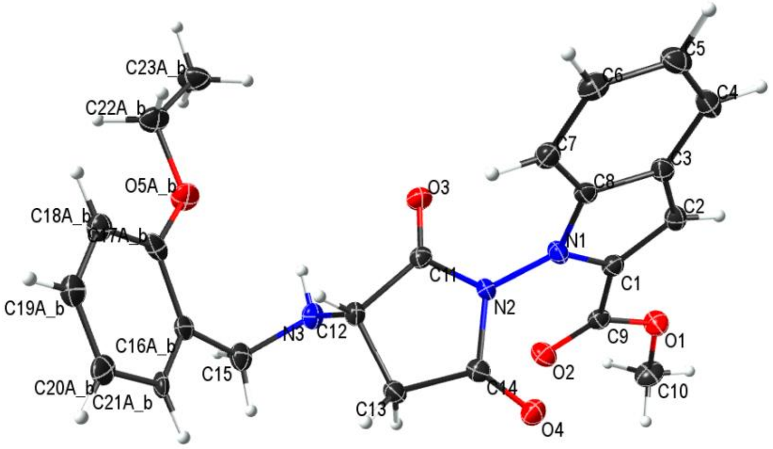

CCDC 2295537

Table 1. Sample and crystal data for N343.

Identification code	N343
Chemical formula	$C_{23}H_{25}N_3O_6$
Formula weight	439.46 g/mol
Temperature	100(2) K
Wavelength	1.54178 Å
Crystal size	0.054 x 0.168 x 0.324 mm
Crystal system	orthorhombic
Space group	P 21 21 21
Unit cell dimensions	a = 6.6376(2) Å α = 90°
	b = 12.5870(3) Å β = 90°

	c = 25.9116(7) Å γ = 90°
Volume	2164.85(10) Å3
Z	4
Density (calculated)	1.348 g/cm^3
Absorption coefficient	0.818 mm^{-1}
F(000)	928

Table 2. Data collection and structure refinement for N343.

Theta range for data collection	3.41 to 72.13°
Index ranges	-8<=h<=8, -15<=k<=15, -31<=l<=31
Reflections collected	87952
Independent reflections	4262 [R(int) = 0.0232]
Coverage of independent reflections	99.9%
Absorption correction	Multi-Scan
Structure solution technique	direct methods
Structure solution program	XT, VERSION 2018/2
Refinement method	Full-matrix least-squares on F^2
Refinement program	SHELXL-2019/1 (Sheldrick, 2019)
Function minimized	Σ w(Fo^2 - Fc^2)^2
Data / restraints / parameters	4262 / 295 / 374
Goodness-of-fit on F2	1.070
Δ/σmax	0.001
Final R indices	4256 data; I>2σ(I) R1 = 0.0238, wR2 = 0.0604
	all data R1 = 0.0239, wR2 = 0.0604
Weighting scheme	$w=1/[\sigma^2(Fo^2)+(0.0324P)^2+0.4297P]$
	Where $P=(Fo^2+2Fc^2)/3$
Absolute structure parameter	0.015(16)
Largest diff. peak and hole	0.115 and -0.194 eÅ^{-3}
R.M.S. deviation from mean	0.037 eÅ^{-3}

NMR Spectra

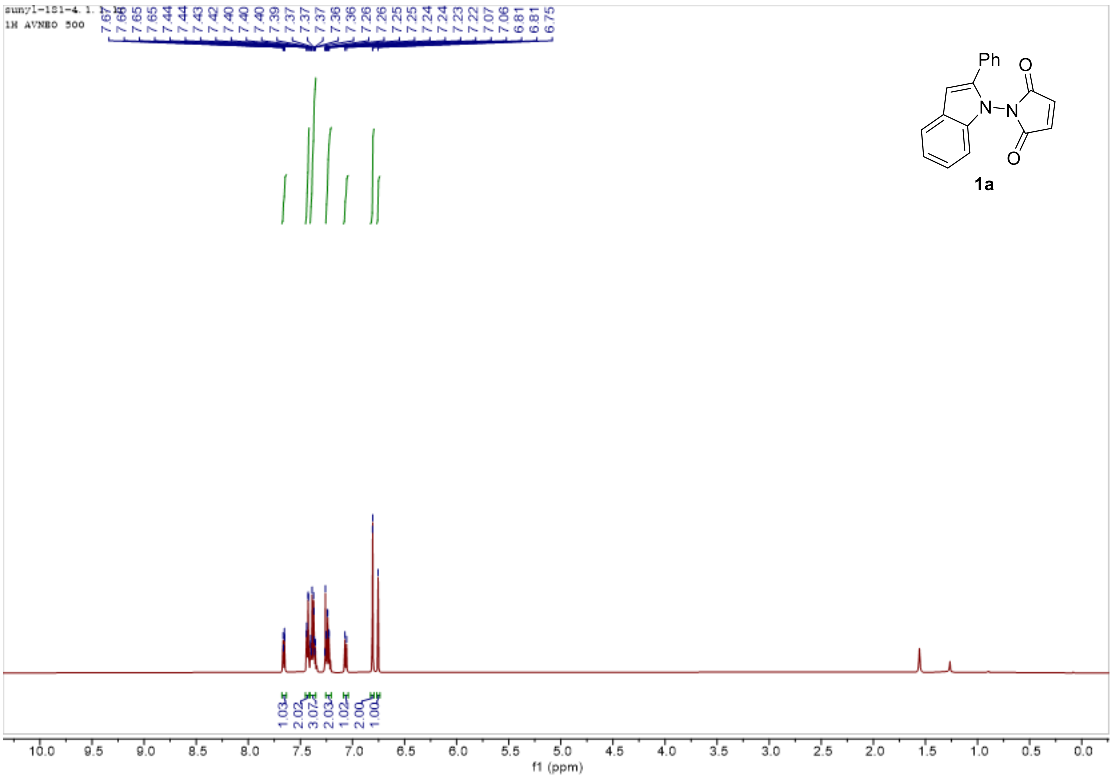

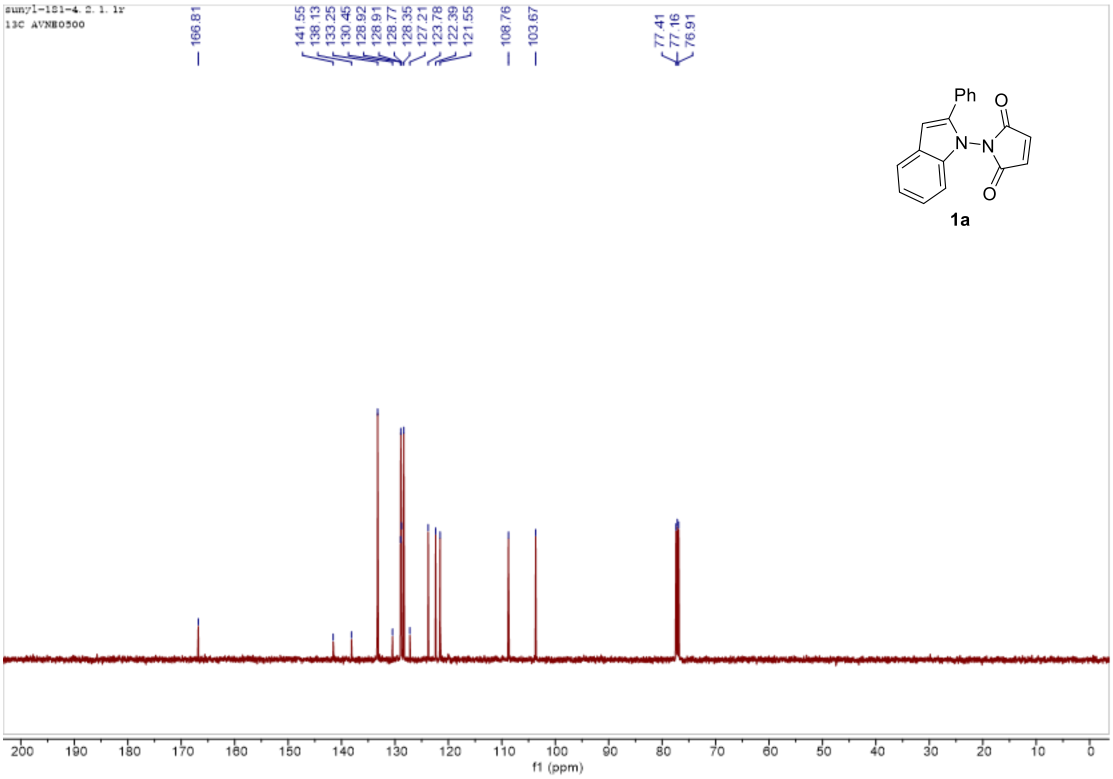

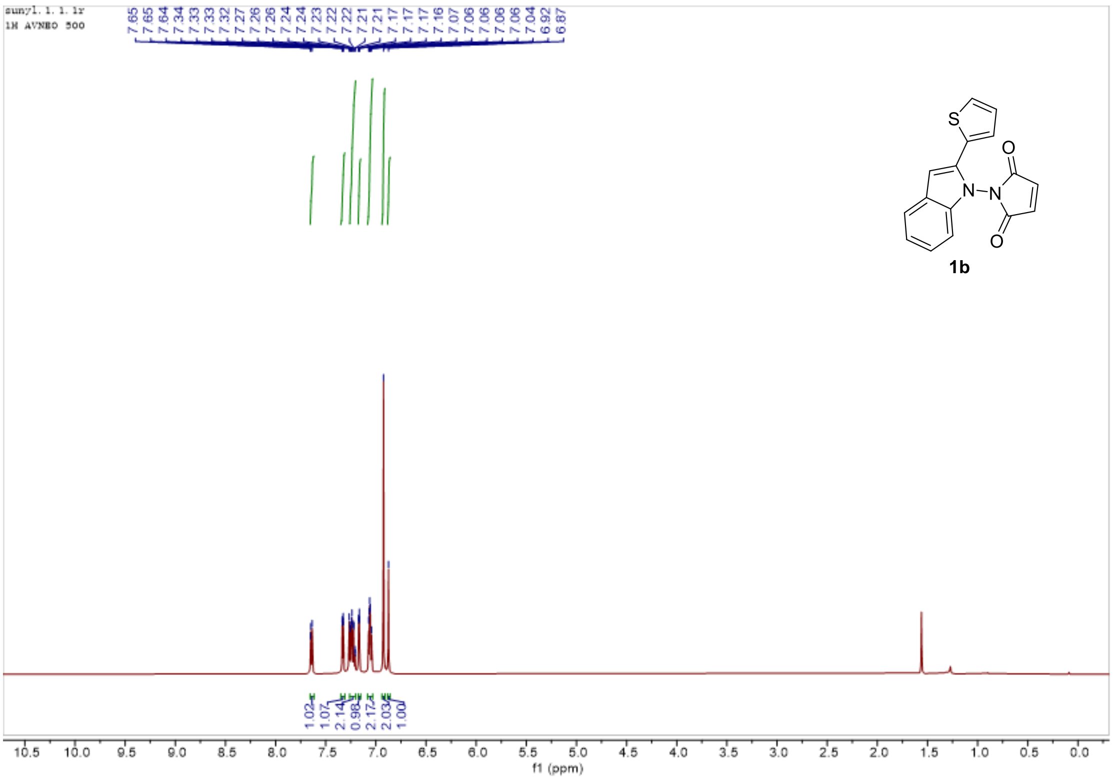
sunyl.1.1.1r
1H AVNEO 500
7.65
7.65
7.64
7.34
7.33
7.33
7.32
7.27
7.26
7.26
7.24
7.24
7.23
7.22
7.22
7.21
7.21
7.17
7.17
7.16
7.07
7.06
7.06
7.06
7.06
7.04
6.92
6.87
1b
1.02
1.07
2.14
0.98
2.17
2.03
1.00
f1 (ppm)

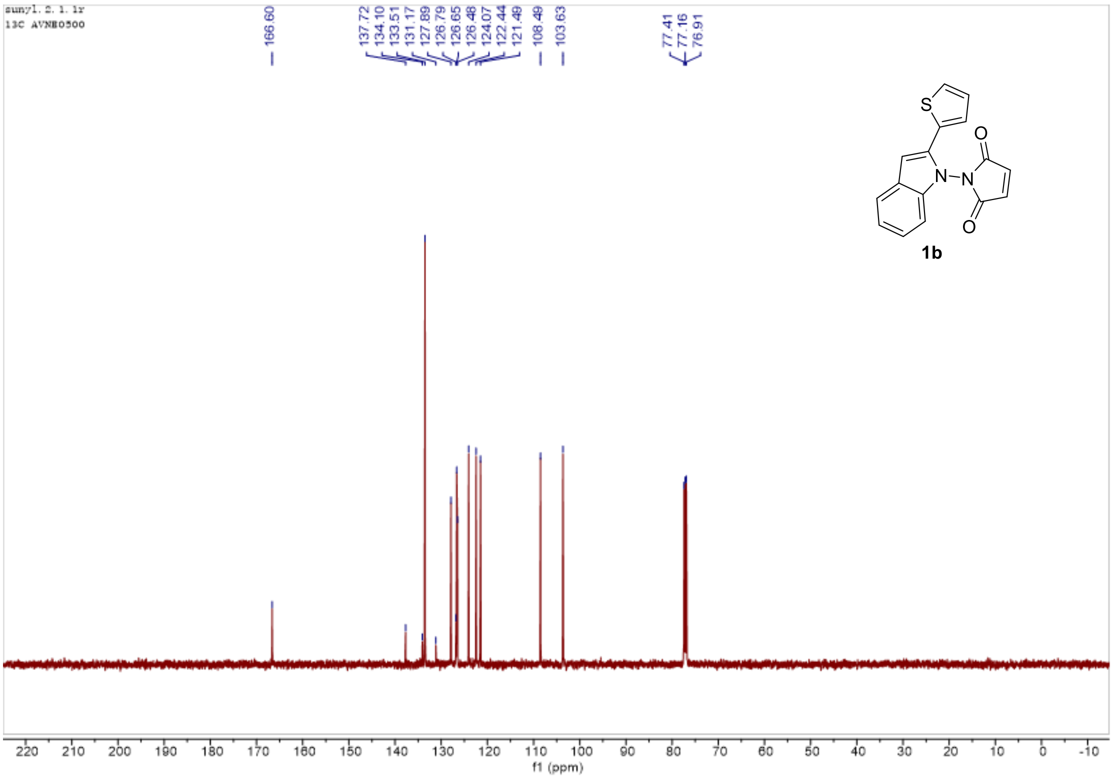
sunyl.2.1.1r
13C AVNEO500
166.60
137.72
134.10
133.51
131.17
127.89
126.79
126.65
126.48
124.07
122.44
121.49
108.49
103.63
77.41
77.16
76.91
1b
f1 (ppm)

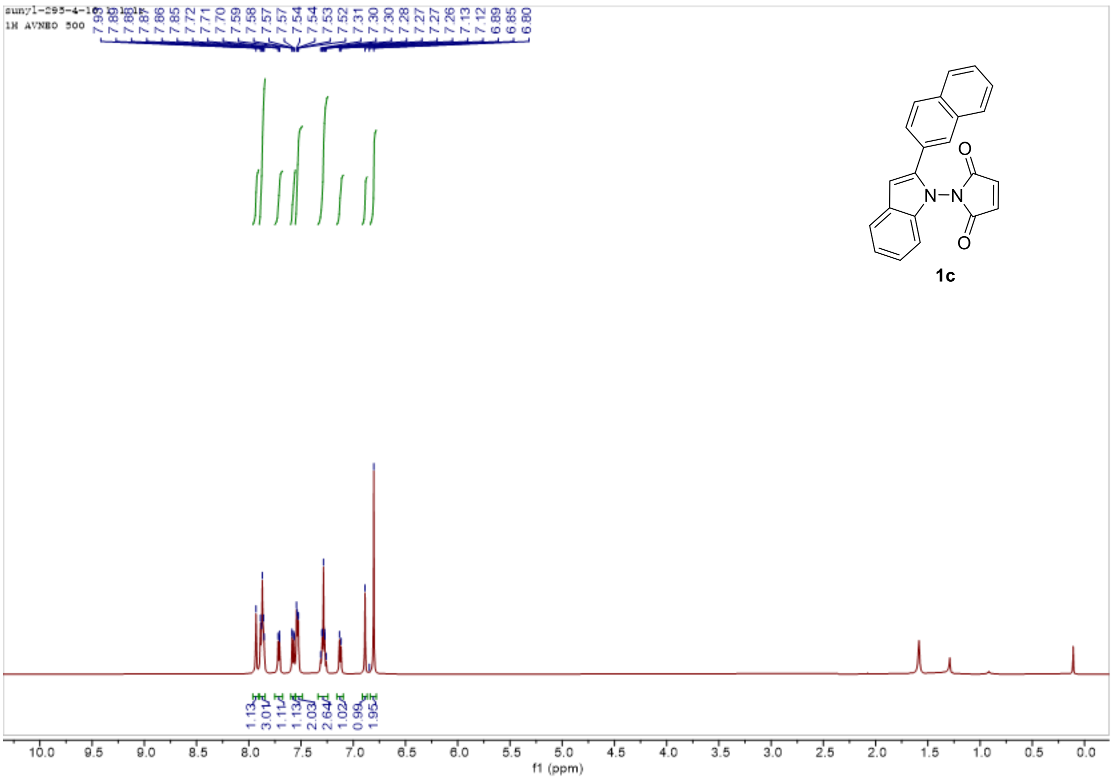
sunyl-293-4-16.1.fid
1H AVNEO 500
1c
f1 (ppm)

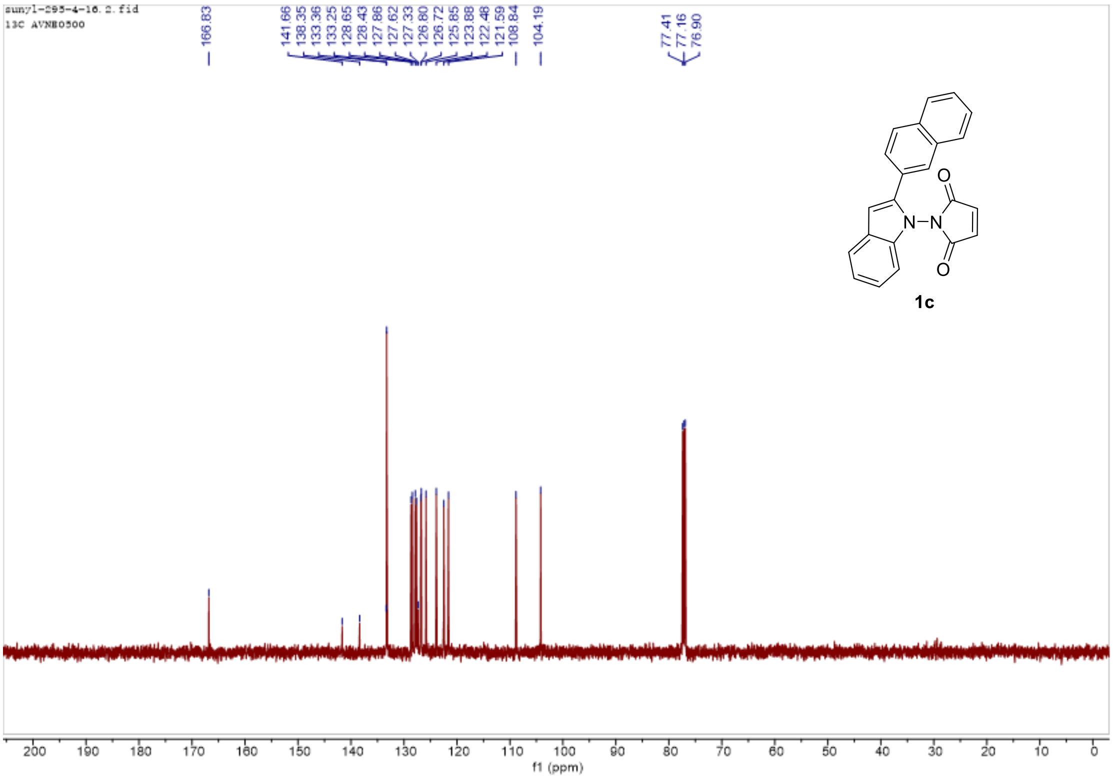
sunyl-293-4-16.2.fid
13C AVNEO500
166.83
141.66
138.35
133.36
133.25
128.65
128.43
127.86
127.62
127.33
126.80
126.72
125.85
123.88
122.48
121.59
108.84
104.19
77.41
77.16
76.90
1c
f1 (ppm)

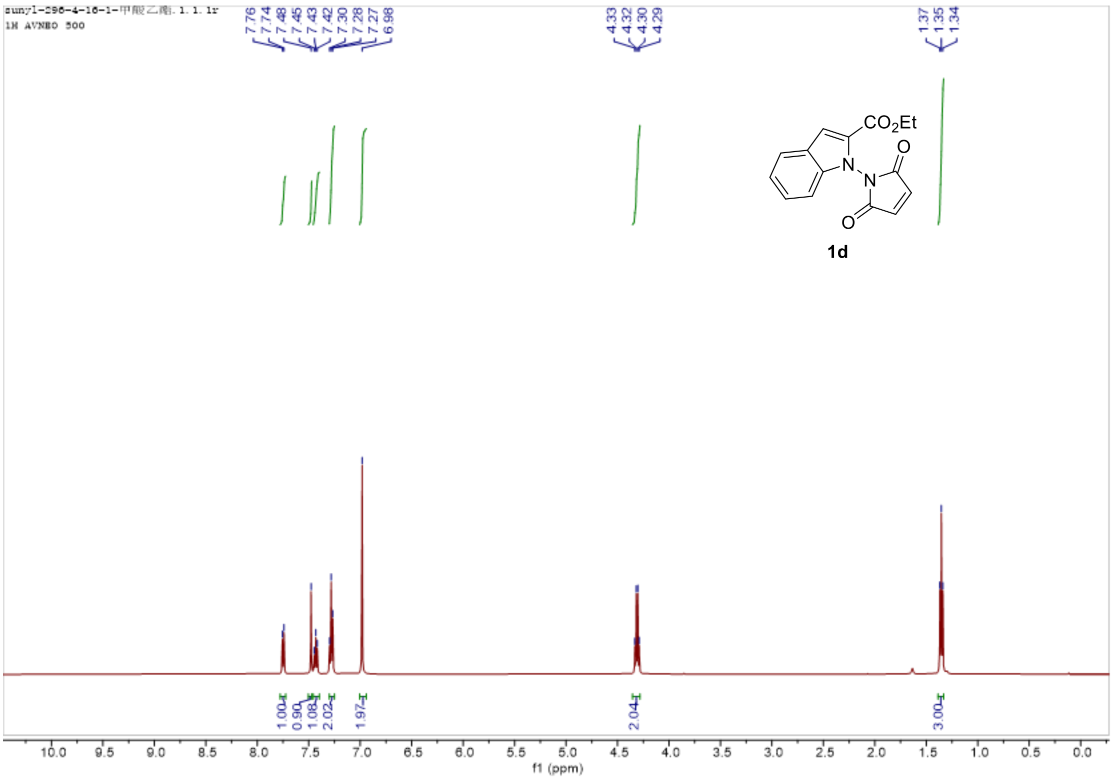
sunyl-296-4-16-1-甲酸乙酯.1.1.1r
1H AVNEO 500
7.76
7.74
7.48
7.45
7.43
7.42
7.30
7.28
7.27
6.98
4.33
4.32
4.30
4.29
1.37
1.35
1.34
CO2Et
N
N
O
O
1d
1.00
0.90
1.08
2.02
1.97
2.04
3.00
f1 (ppm)

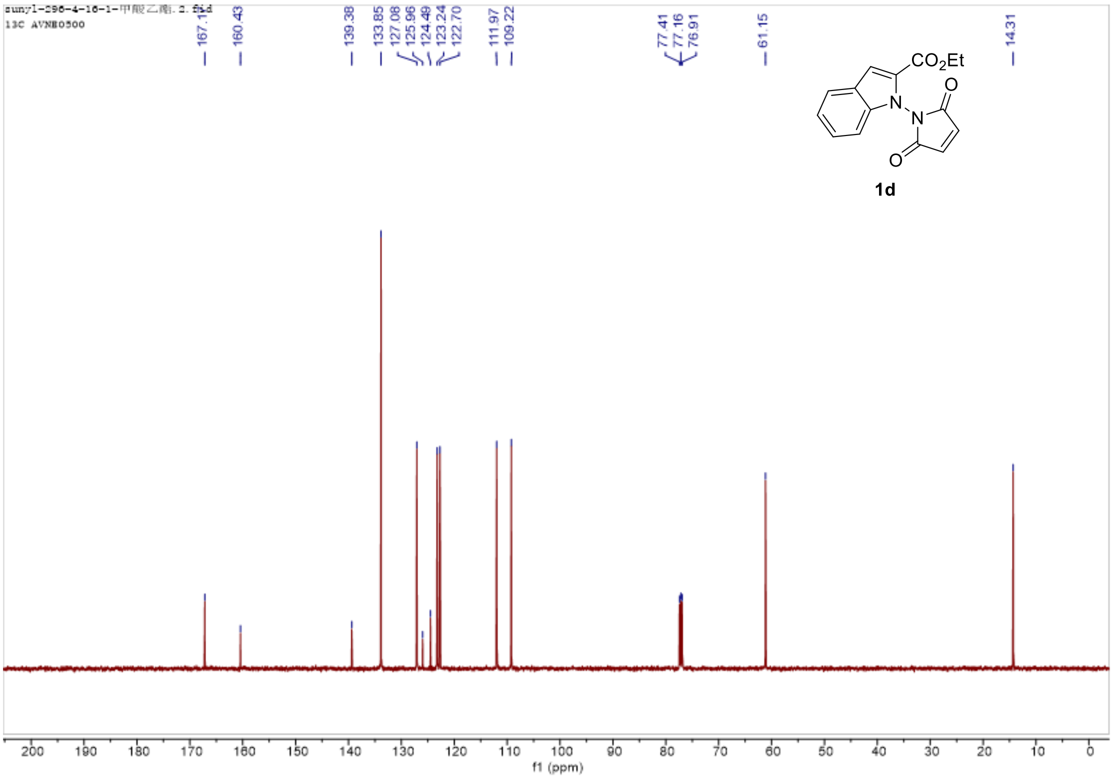
sunyl-296-4-16-1-甲酸乙酯.2.fid
13C AVNEO500
167.14
160.43
139.38
133.85
127.08
125.96
124.49
123.24
122.70
111.97
109.22
77.41
77.16
76.91
61.15
14.31
CO2Et
N
N
O
O
1d
f1 (ppm)

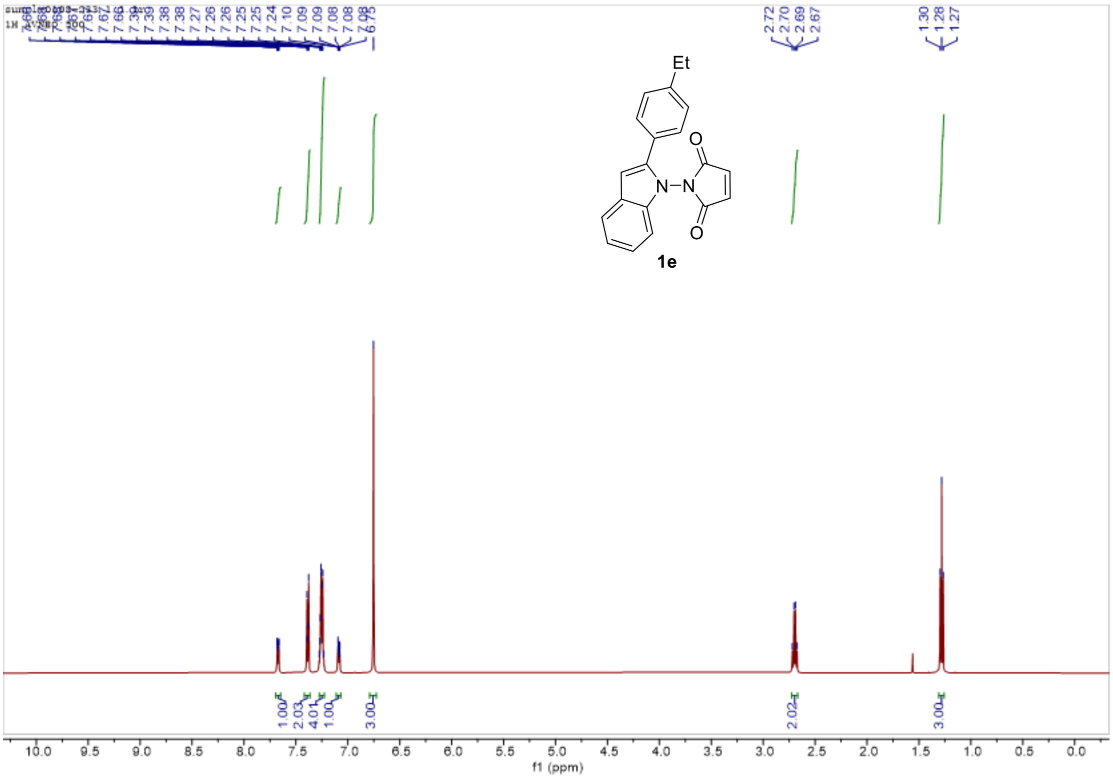

Et
O
N
N
O
1e
2.72
2.70
2.69
2.67
1.30
1.28
1.27
1.00
2.03
4.01
1.00
3.00
2.02
3.00
f1 (ppm)

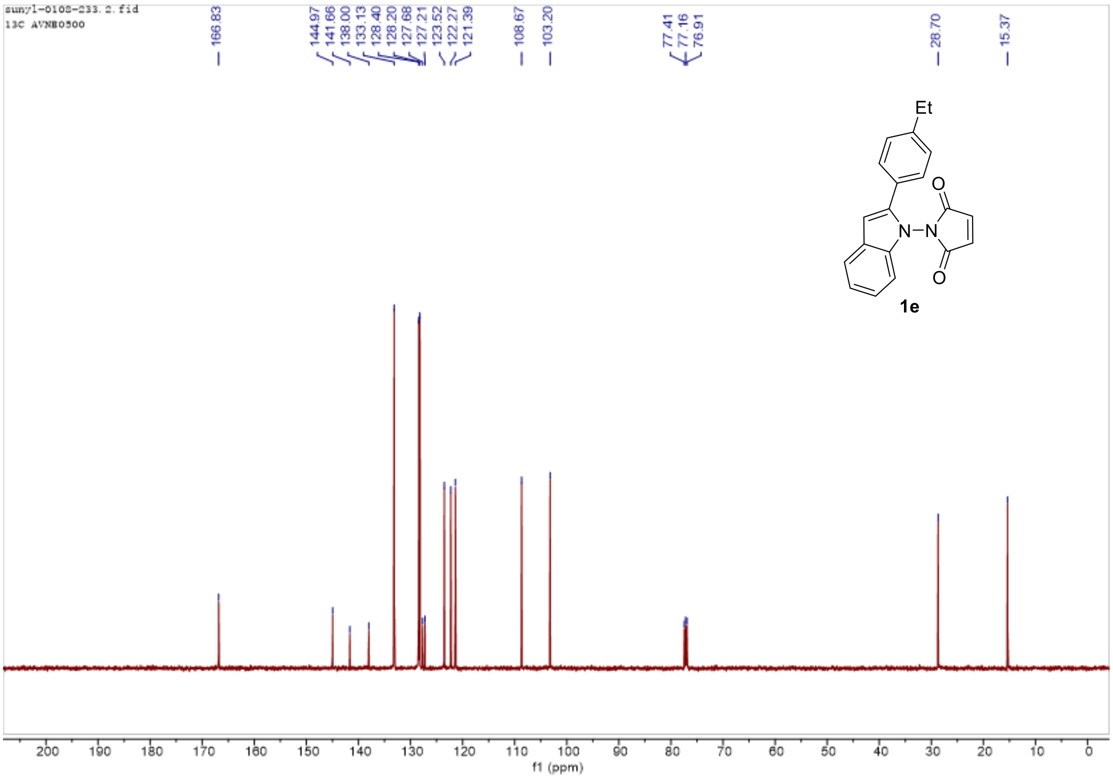

sunyl-0108-233.2.fid
13C AVNEO500
166.83
144.97
141.66
138.00
133.13
128.40
128.20
127.68
127.21
123.52
122.27
121.39
108.67
103.20
77.41
77.16
76.91
28.70
15.37
Et
O
N
N
O
1e
f1 (ppm)

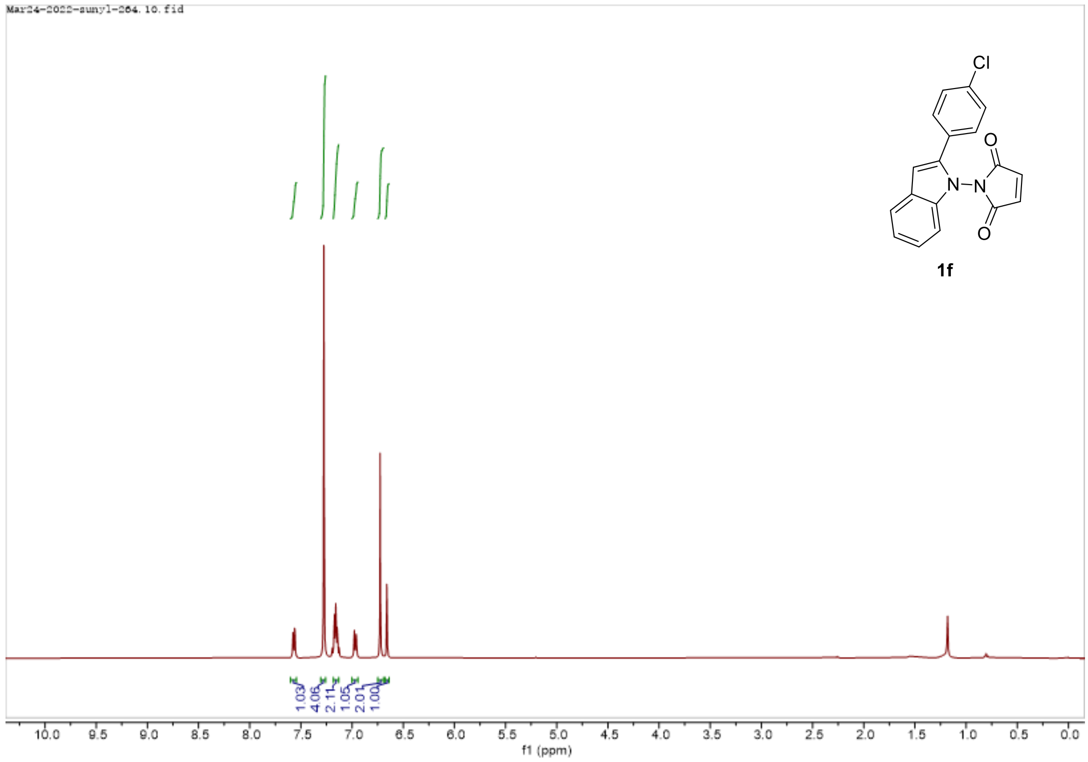
Mar24-2022-sunyl-264.10.fid
Cl
O
N
N
O
1f
1.03
4.06
2.11
1.05
2.01
1.00
10.0 9.5 9.0 8.5 8.0 7.5 7.0 6.5 6.0 5.5 5.0 4.5 4.0 3.5 3.0 2.5 2.0 1.5 1.0 0.5 0.0
f1 (ppm)

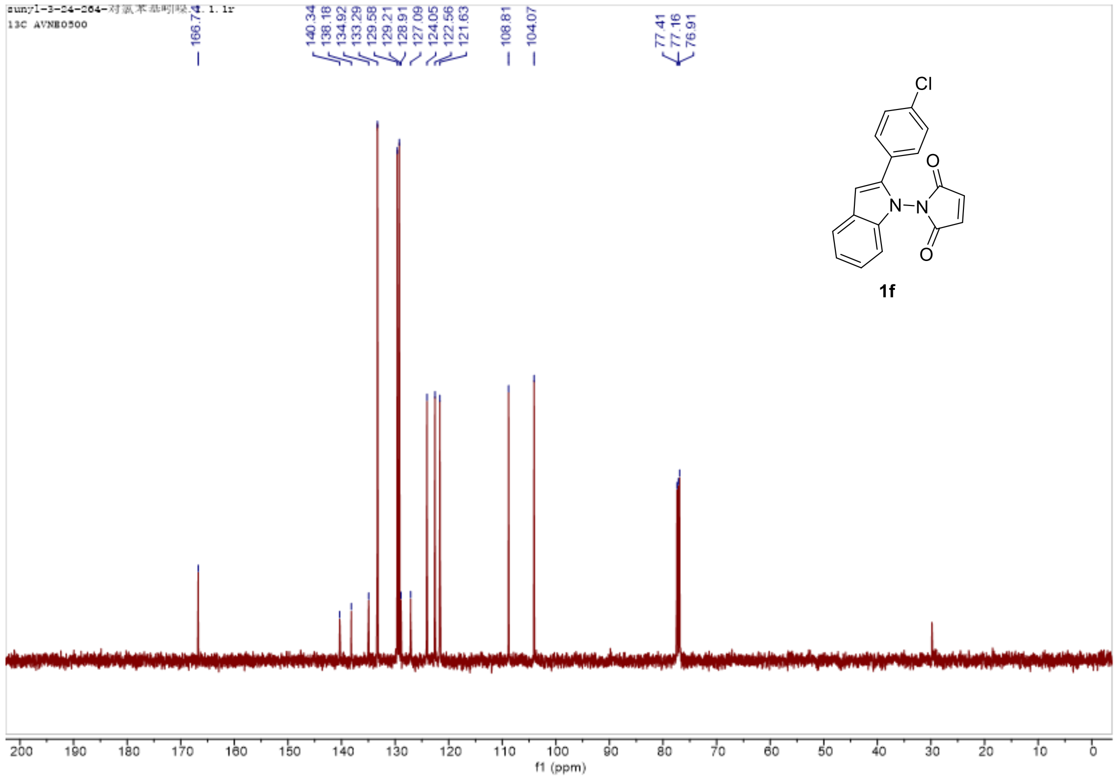
13C AVNE0500
166.74
140.34
138.16
134.92
133.29
129.58
129.21
128.91
127.09
124.05
122.56
121.63
108.81
104.07
77.41
77.16
76.91
Cl
O
N
N
O
1f
200 190 180 170 160 150 140 130 120 110 100 90 80 70 60 50 40 30 20 10 0
f1 (ppm)

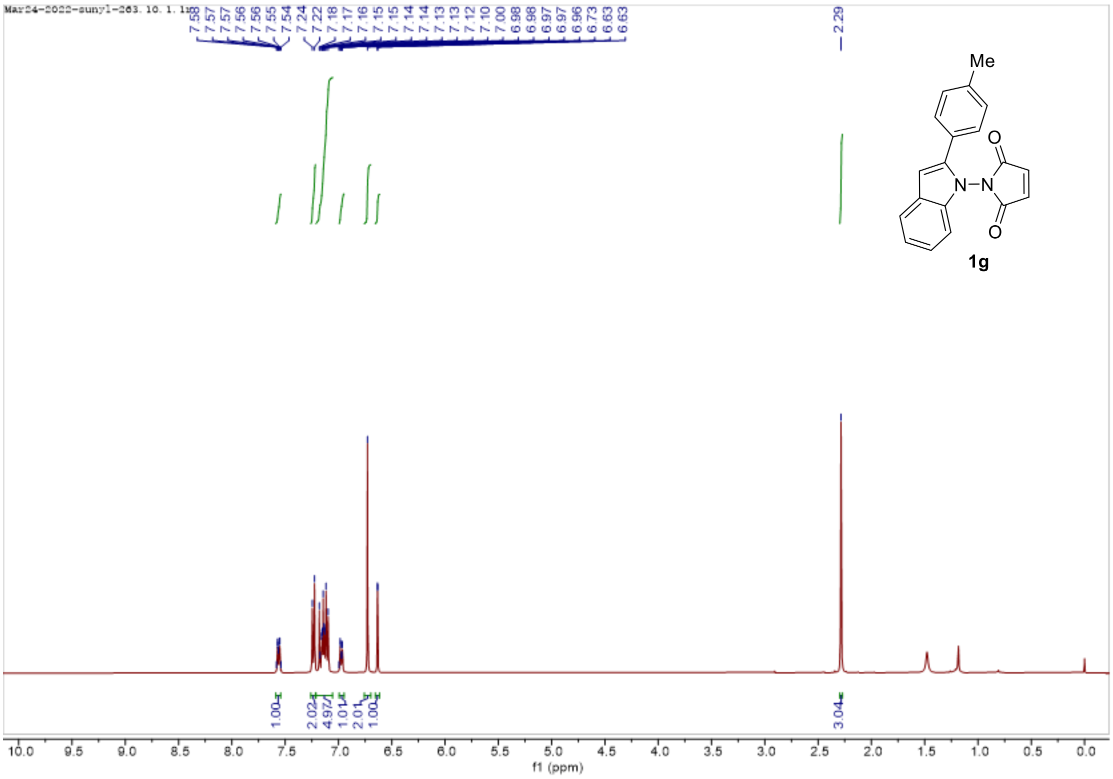
Mar24-2022-sunyl-263.10.1.1r
7.58
7.57
7.57
7.56
7.56
7.55
7.54
7.24
7.22
7.18
7.17
7.16
7.15
7.15
7.14
7.14
7.13
7.13
7.12
7.10
7.00
6.98
6.98
6.97
6.97
6.96
6.73
6.63
6.63
2.29
Me
O
N–N
O
1g
1.00
2.02
4.97
1.01
2.01
1.00
3.04
f1 (ppm)

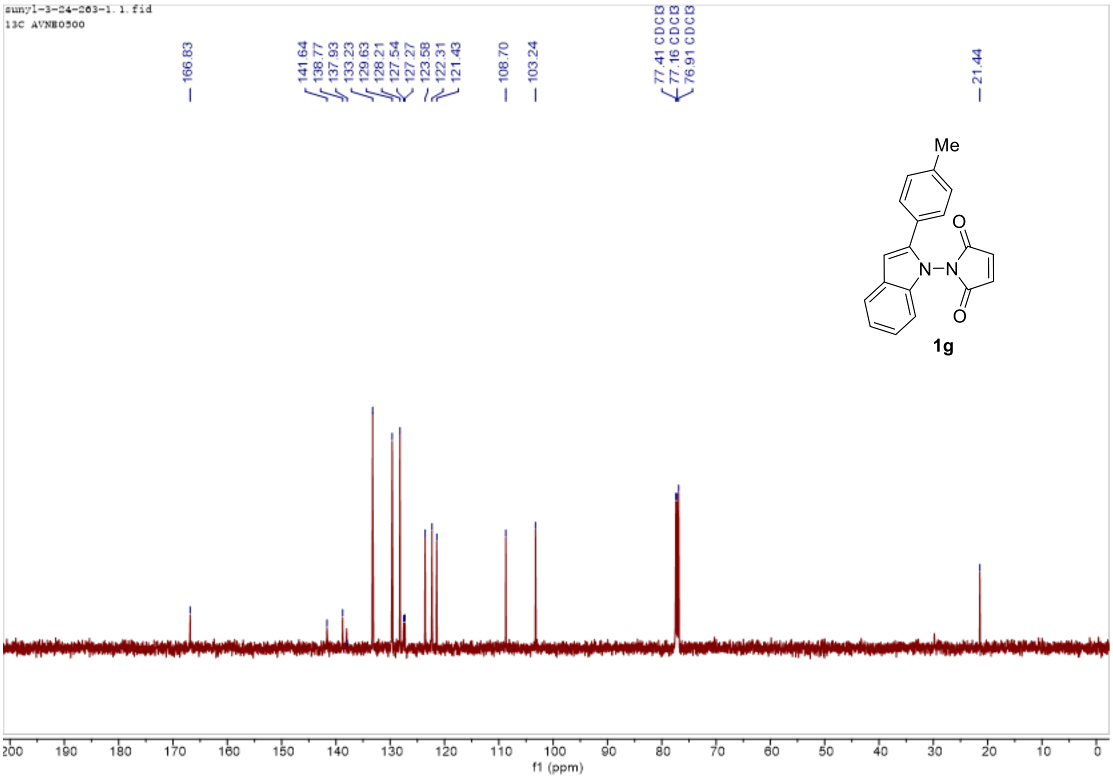
sunyl-3-24-263-1.1.fid
13C AVNEO500
166.83
141.64
138.77
137.93
133.23
129.63
128.21
127.54
127.27
123.58
122.31
121.43
108.70
103.24
77.41 CDCl3
77.16 CDCl3
76.91 CDCl3
21.44
Me
O
N–N
O
1g
f1 (ppm)

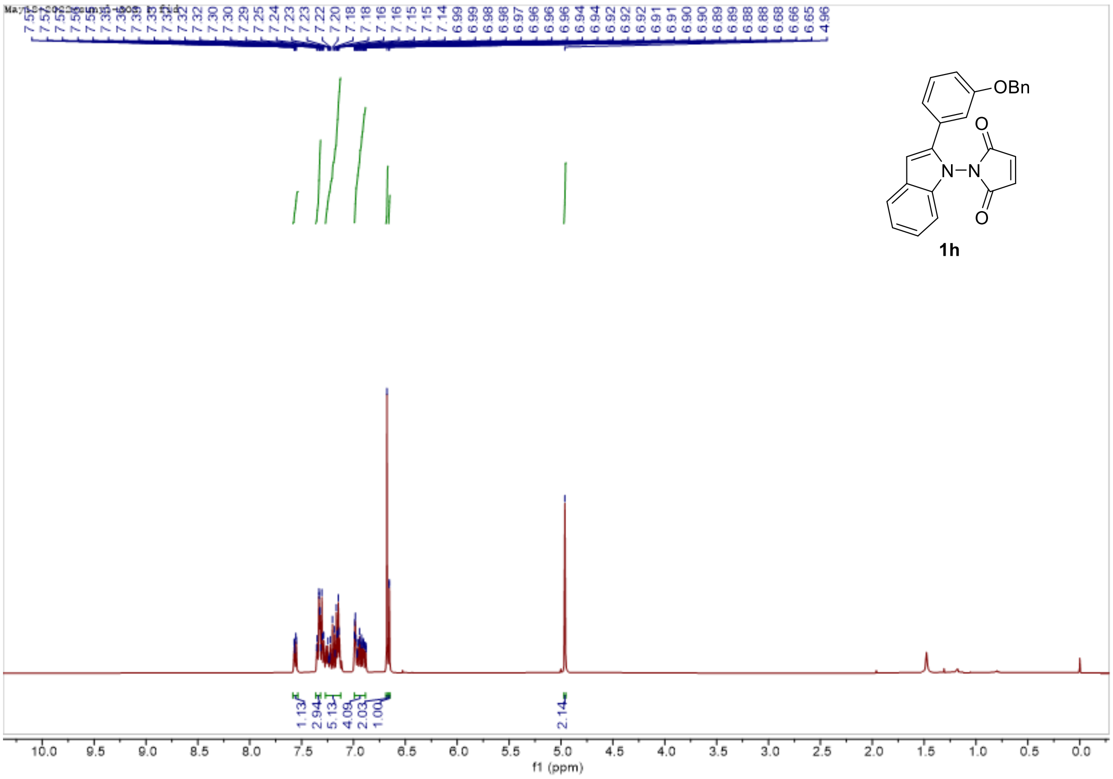
OBn
1h
1.13
2.94
5.13
4.09
2.03
1.00
2.14
f1 (ppm)

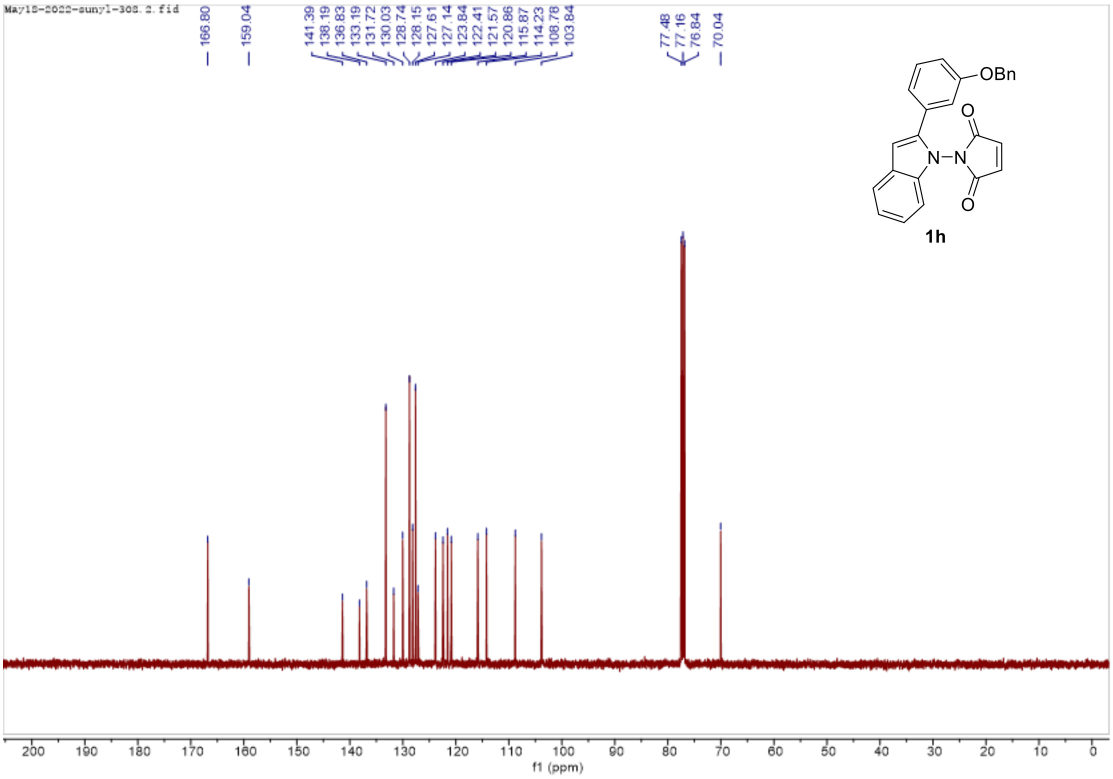
May18-2022-sunyl-308.2.fid
166.80
159.04
141.39
138.19
136.83
133.19
131.72
130.03
128.74
128.15
127.61
127.14
123.84
122.41
121.57
120.86
115.87
114.23
108.78
103.84
77.48
77.16
76.84
70.04
OBn
1h
f1 (ppm)

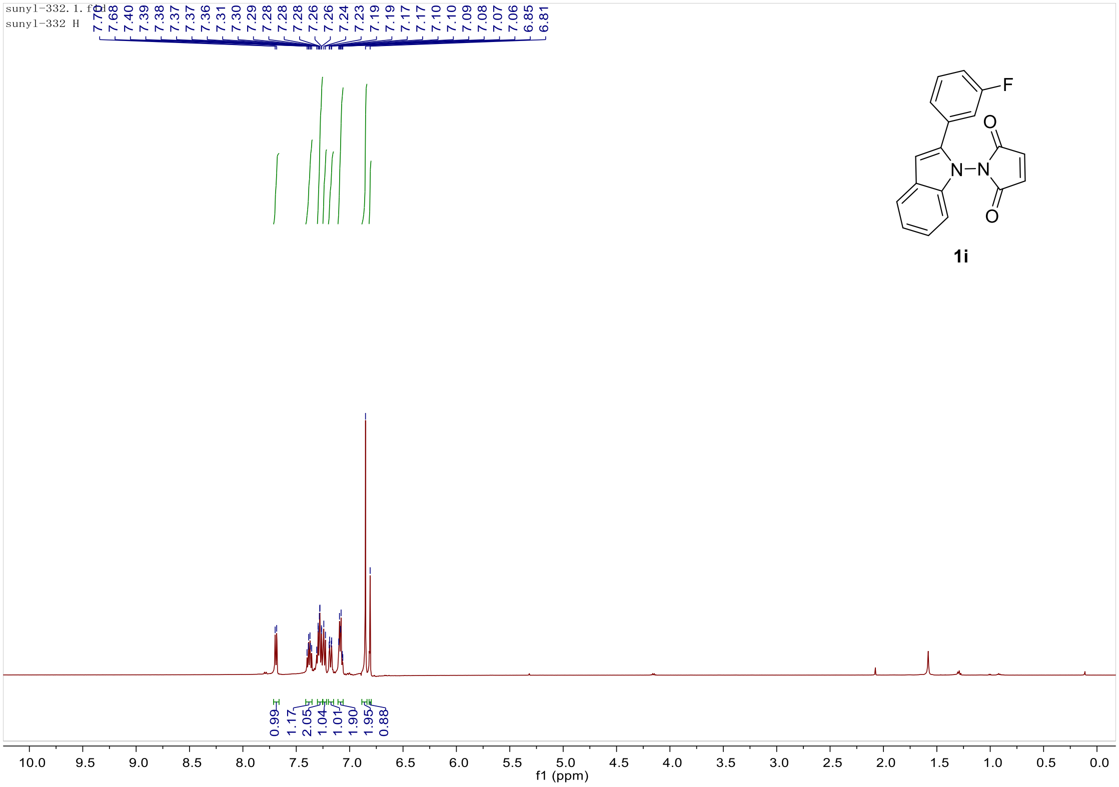
sunyl-332.1.fid
sunyl-332 H
7.70 7.68 7.40 7.39 7.38 7.37 7.37 7.36 7.31 7.30 7.29 7.28 7.28 7.28 7.26 7.26 7.24 7.23 7.19 7.17 7.17 7.10 7.10 7.09 7.08 7.07 7.06 6.85 6.81
0.99 1.17 2.05 1.04 1.01 1.90 1.95 0.88
F
O
N
N
O
1i
10.0 9.5 9.0 8.5 8.0 7.5 7.0 6.5 6.0 5.5 5.0 4.5 4.0 3.5 3.0 2.5 2.0 1.5 1.0 0.5 0.0
f1 (ppm)

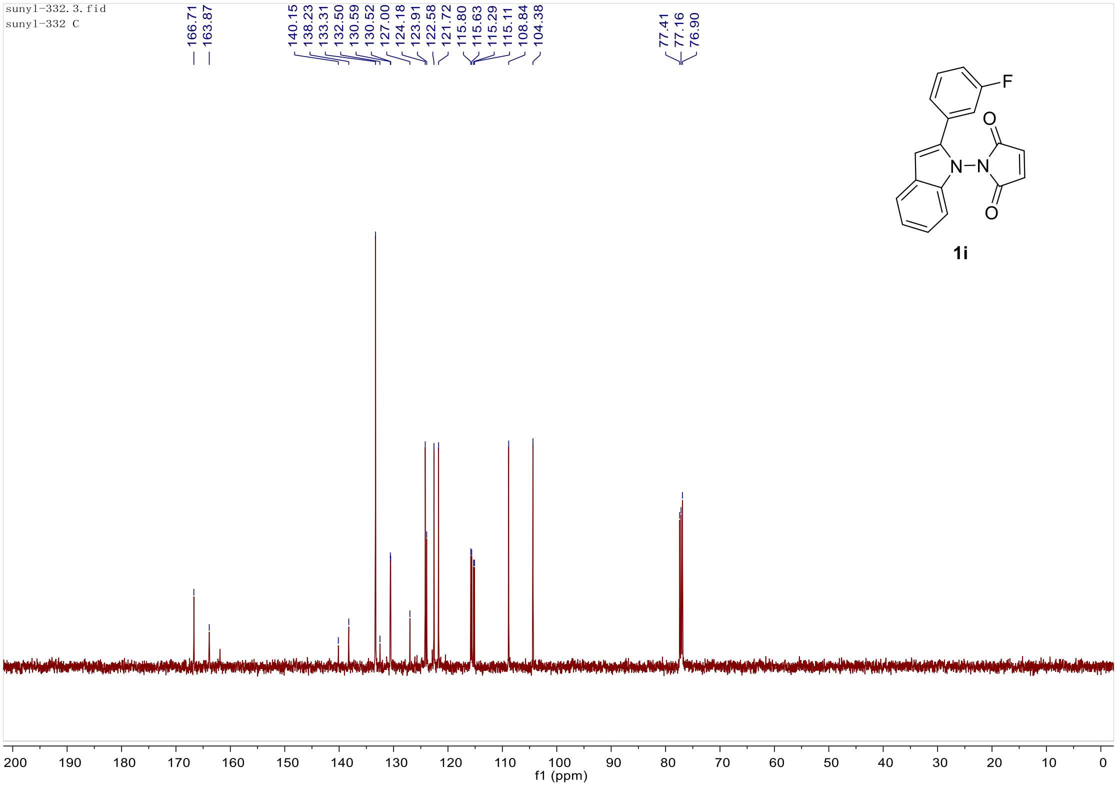
sunyl-332.3.fid
sunyl-332 C
166.71 163.87
140.15 138.23 133.31 132.50 130.59 130.52 127.00 124.18 123.91 122.58 121.72 115.80 115.63 115.29 115.11 108.84 104.38
77.41 77.16 76.90
F
O
N
N
O
1i
200 190 180 170 160 150 140 130 120 110 100 90 80 70 60 50 40 30 20 10 0
f1 (ppm)

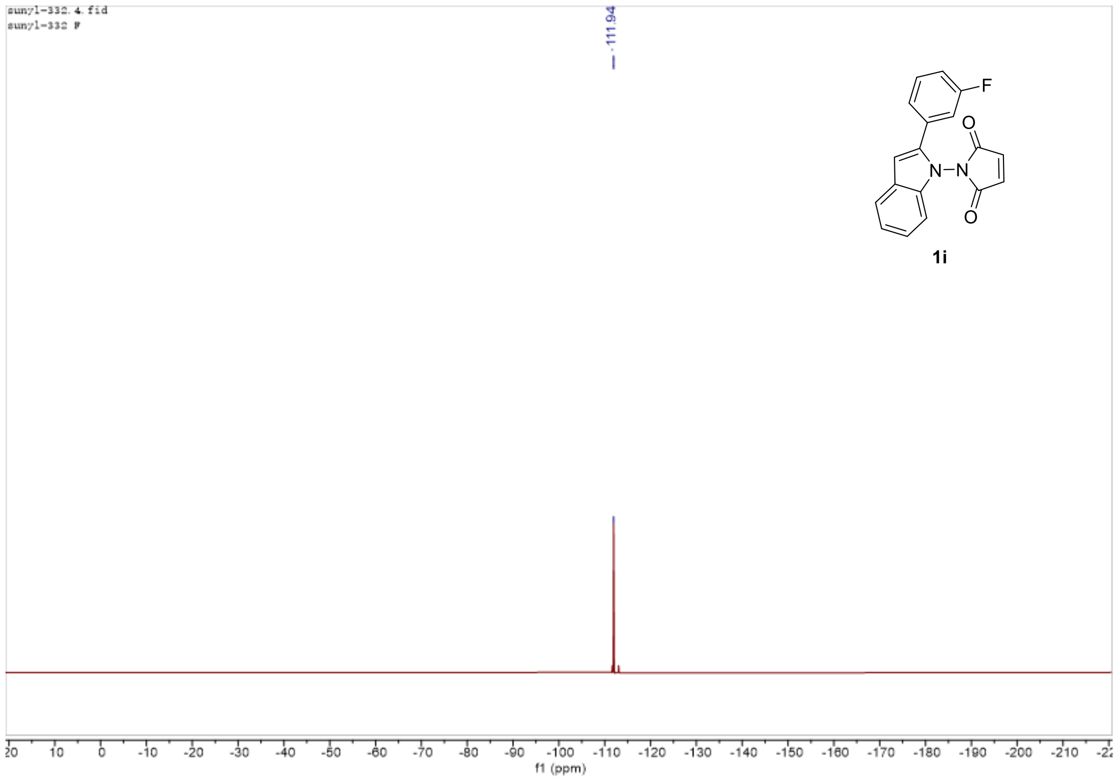

1i

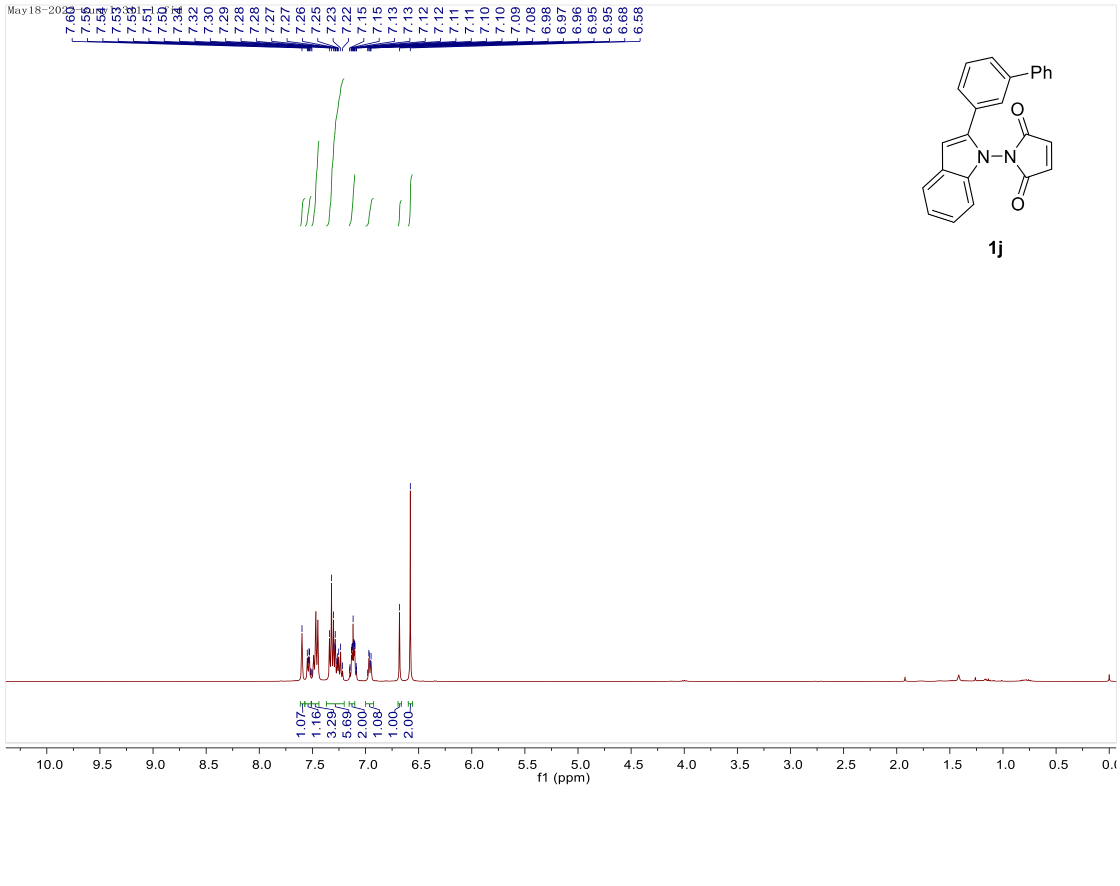

1j

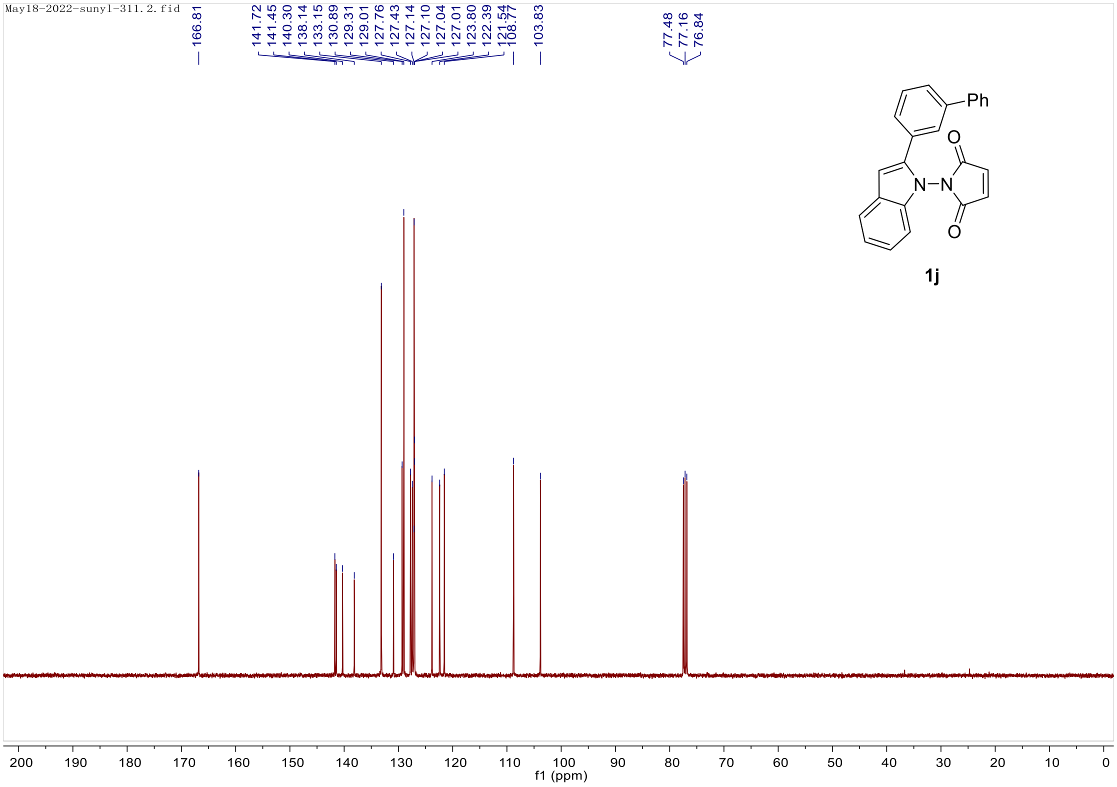
May18-2022-sunyl-311.2.fid
166.81
141.72
141.45
140.30
138.14
133.15
130.89
129.31
129.01
127.76
127.43
127.14
127.10
127.04
127.01
123.80
122.39
121.54
108.77
103.83
77.48
77.16
76.84
Ph
N
N
O
O
1j
200 190 180 170 160 150 140 130 120 110 100 90 80 70 60 50 40 30 20 10 0
f1 (ppm)

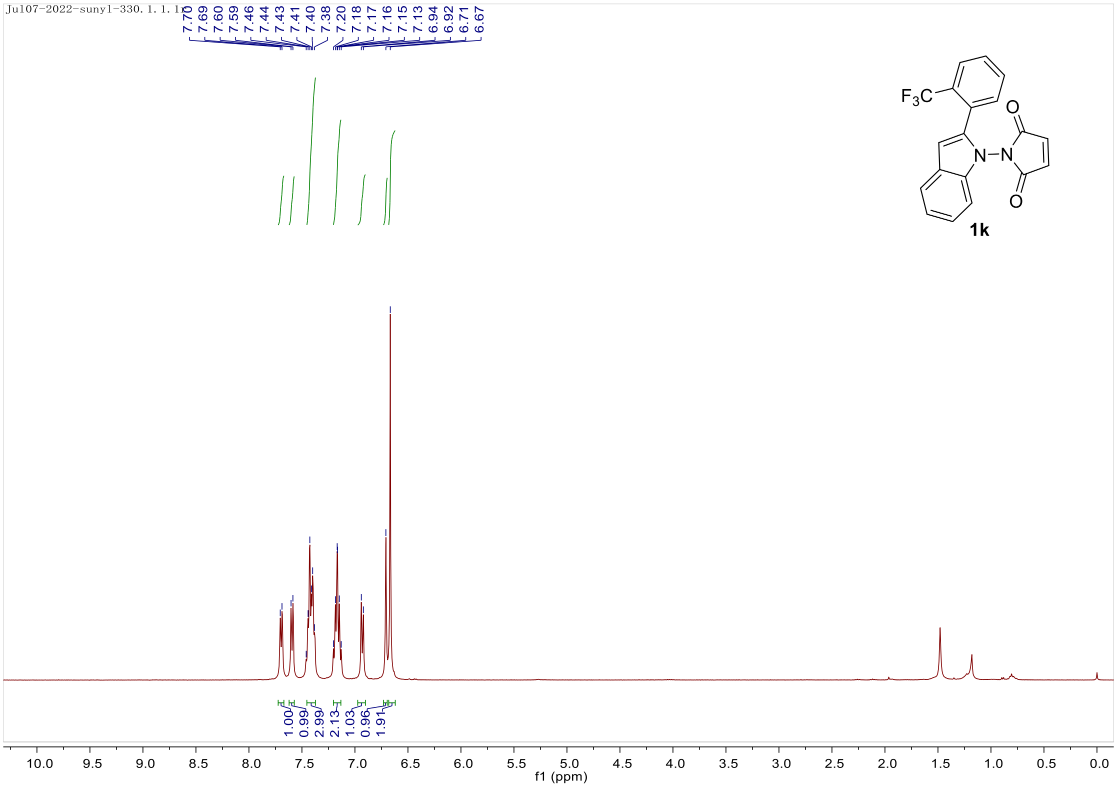
Jul07-2022-sunyl-330.1.1.1
7.70
7.69
7.60
7.59
7.46
7.44
7.43
7.41
7.40
7.38
7.20
7.18
7.17
7.16
7.15
7.13
6.94
6.92
6.71
6.67
F3C
N
N
O
O
1k
1.00
0.99
2.99
2.13
1.03
0.96
1.91
10.0 9.5 9.0 8.5 8.0 7.5 7.0 6.5 6.0 5.5 5.0 4.5 4.0 3.5 3.0 2.5 2.0 1.5 1.0 0.5 0.0
f1 (ppm)

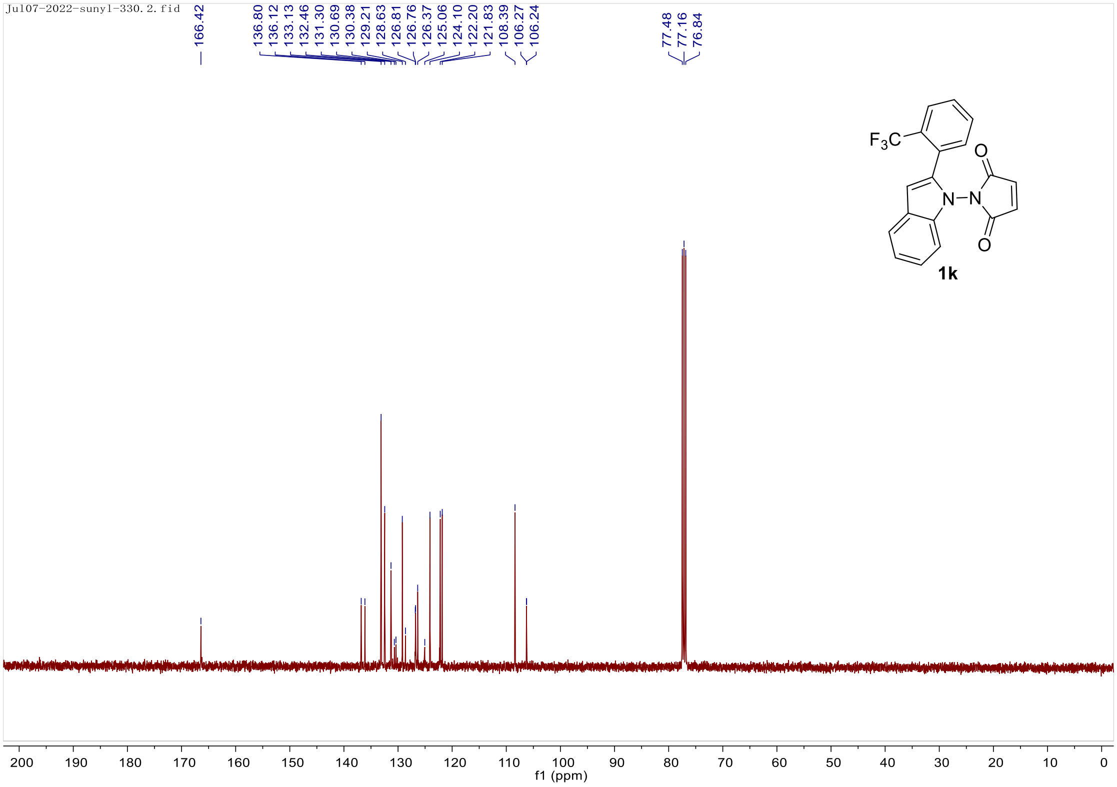

Jul07-2022-suny1-330.2.fid
166.42
136.80
136.12
133.13
132.46
131.30
130.69
130.38
129.21
128.63
126.81
126.76
126.37
125.06
124.10
122.20
121.83
108.39
106.27
106.24
77.48
77.16
76.84
F3C
1k
200 190 180 170 160 150 140 130 120 110 100 90 80 70 60 50 40 30 20 10 0
f1 (ppm)

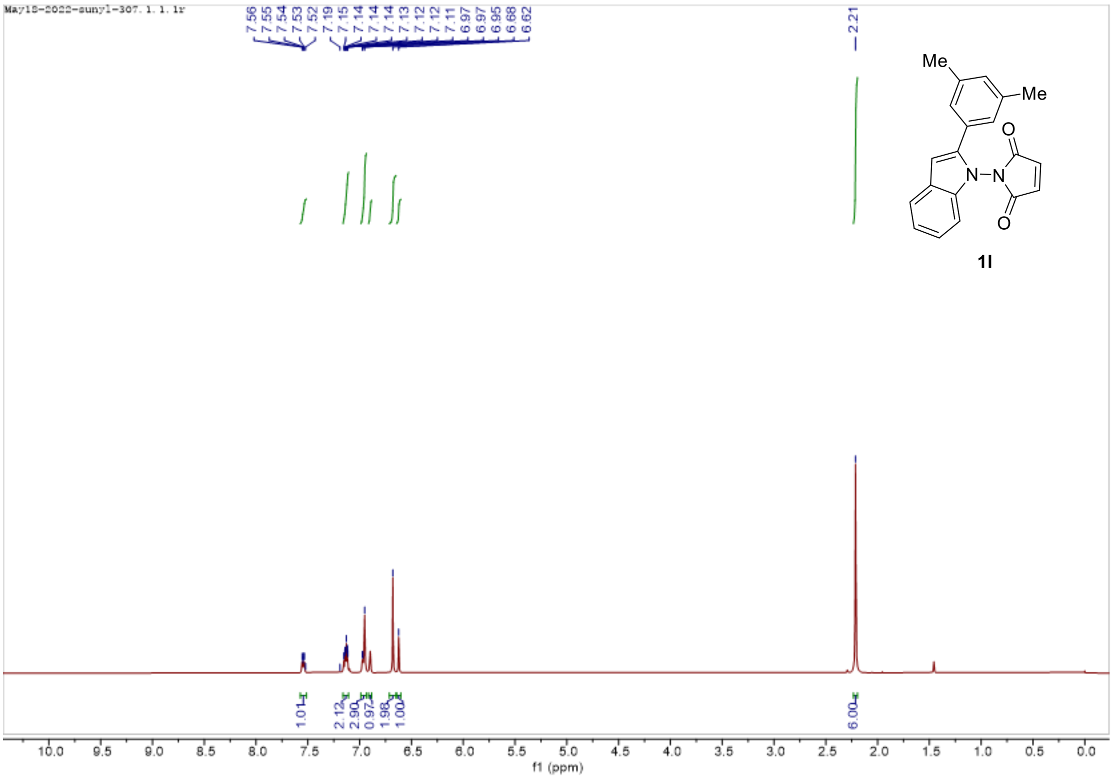

May15-2022-suny1-307.1.1.1r
7.56
7.55
7.54
7.53
7.52
7.19
7.15
7.14
7.14
7.13
7.12
7.12
7.11
6.97
6.97
6.95
6.68
6.62
2.21
Me
Me
1l
1.01
2.12
2.90
0.97
1.98
1.00
6.00
10.0 9.5 9.0 8.5 8.0 7.5 7.0 6.5 6.0 5.5 5.0 4.5 4.0 3.5 3.0 2.5 2.0 1.5 1.0 0.5 0.0
f1 (ppm)

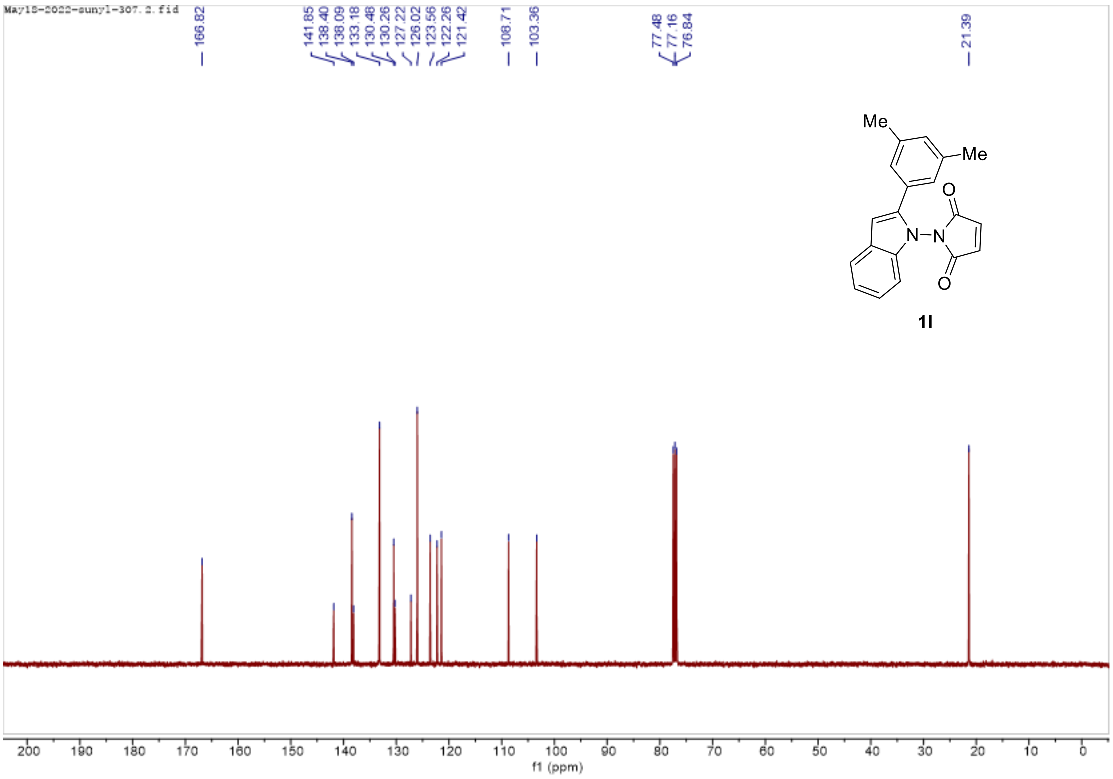
May18-2022-sunyl-307.2.fid
166.82
141.85 138.40 138.09 133.18 130.48 130.26 127.22 126.02 123.56 122.26 121.42
108.71
103.36
77.48 77.16 76.84
21.39
Me
Me
N–N
O
O
1l
f1 (ppm)

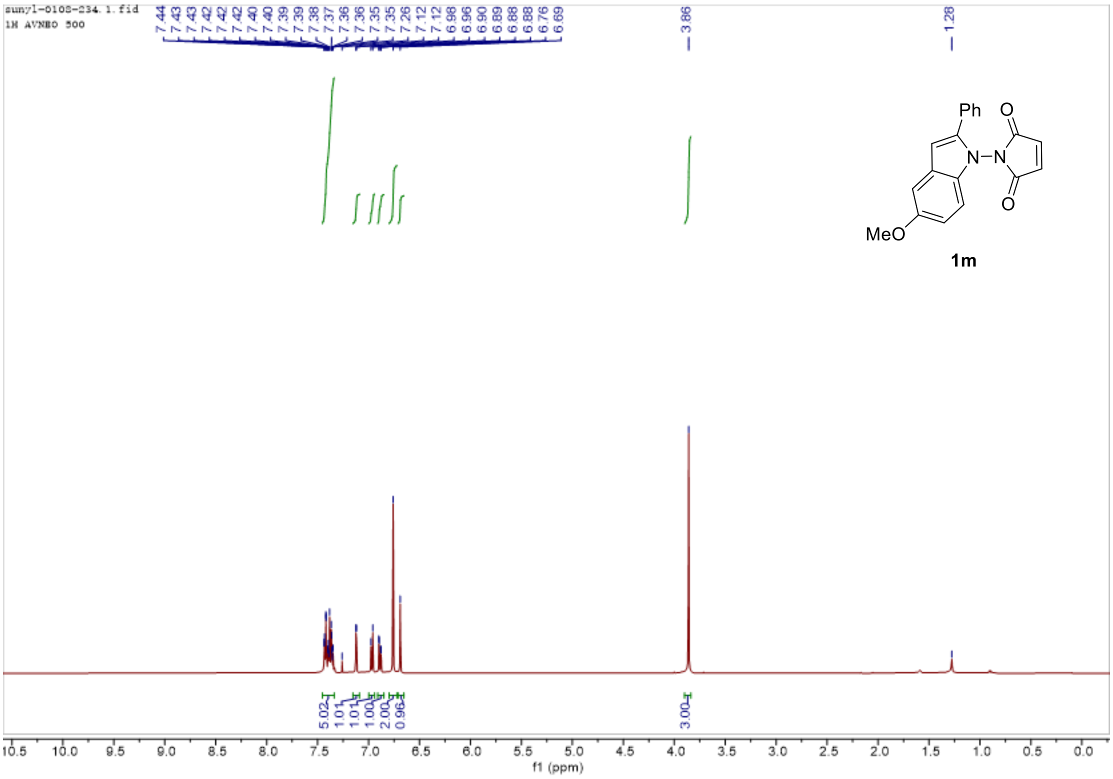
sunyl-0108-234.1.fid
1H AVNEO 500
7.44 7.43 7.43 7.42 7.42 7.42 7.40 7.40 7.39 7.39 7.38 7.37 7.36 7.36 7.35 7.35 7.26 7.12 7.12 6.98 6.96 6.90 6.89 6.88 6.88 6.76 6.69
3.86
1.28
Ph
O
N–N
O
MeO
1m
5.02 1.01 1.01 1.00 2.00 0.96
3.00
f1 (ppm)

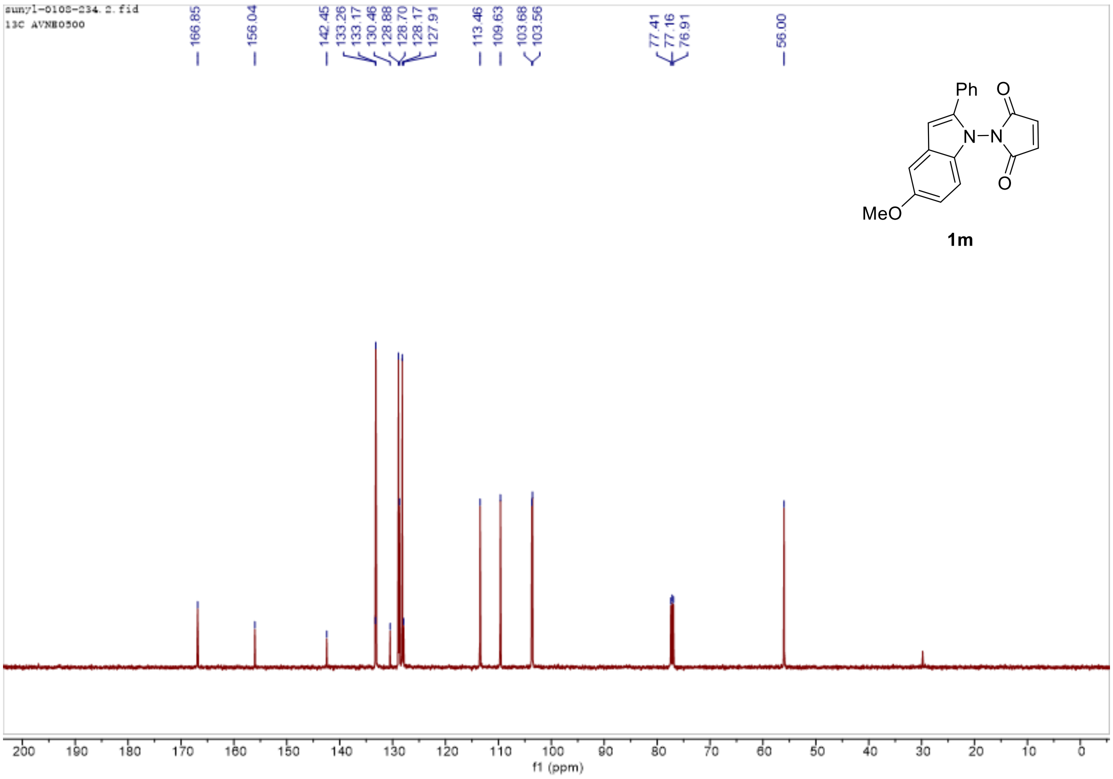

sunyl-0108-234.2.fid
13C AVNEO500
166.85
156.04
142.45
133.26
133.17
130.46
128.88
128.70
128.17
127.91
113.46
109.63
103.68
103.56
77.41
77.16
76.91
56.00
Ph
O
N
N
O
MeO
1m
200 190 180 170 160 150 140 130 120 110 100 90 80 70 60 50 40 30 20 10 0
f1 (ppm)

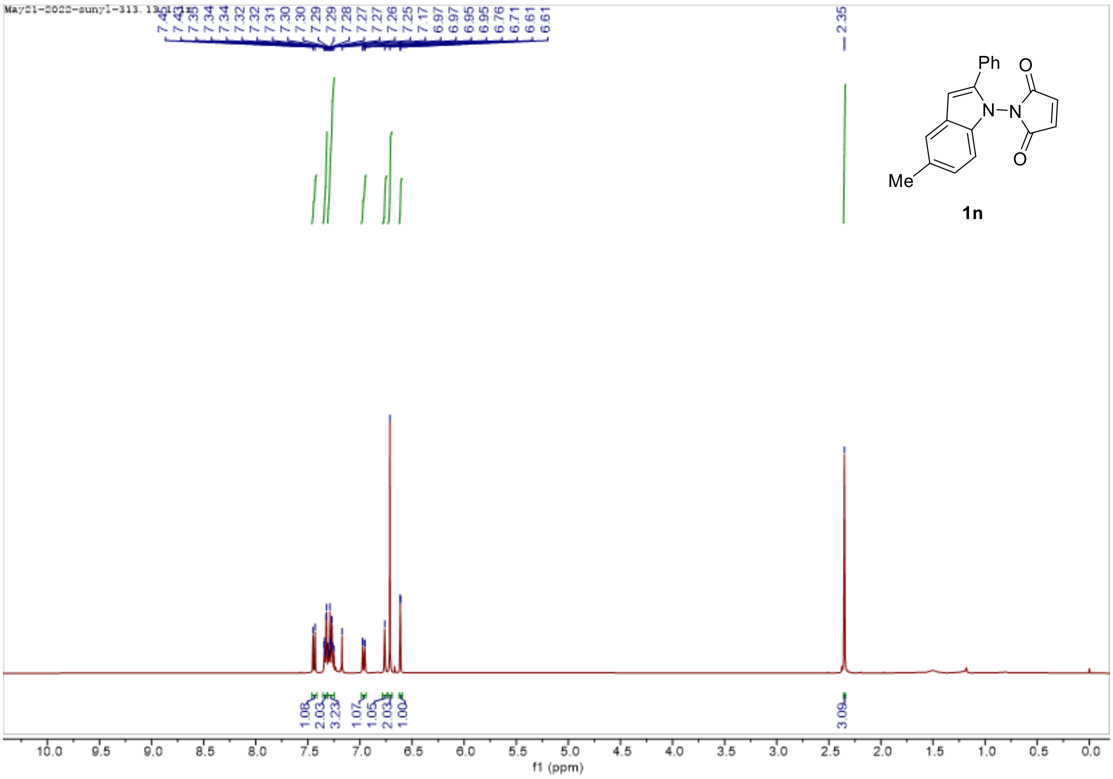

May21-2022-sunyl-313.
7.45 7.43 7.35 7.34 7.34 7.32 7.32 7.31 7.30 7.30 7.29 7.29 7.28 7.27 7.27 7.26 7.25 7.17 6.97 6.97 6.95 6.95 6.76 6.71 6.61 6.61
2.35
Ph
O
N
N
O
Me
1n
1.06
2.03
3.23
1.07
1.05
2.03
1.00
3.09
10.0 9.5 9.0 8.5 8.0 7.5 7.0 6.5 6.0 5.5 5.0 4.5 4.0 3.5 3.0 2.5 2.0 1.5 1.0 0.5 0.0
f1 (ppm)

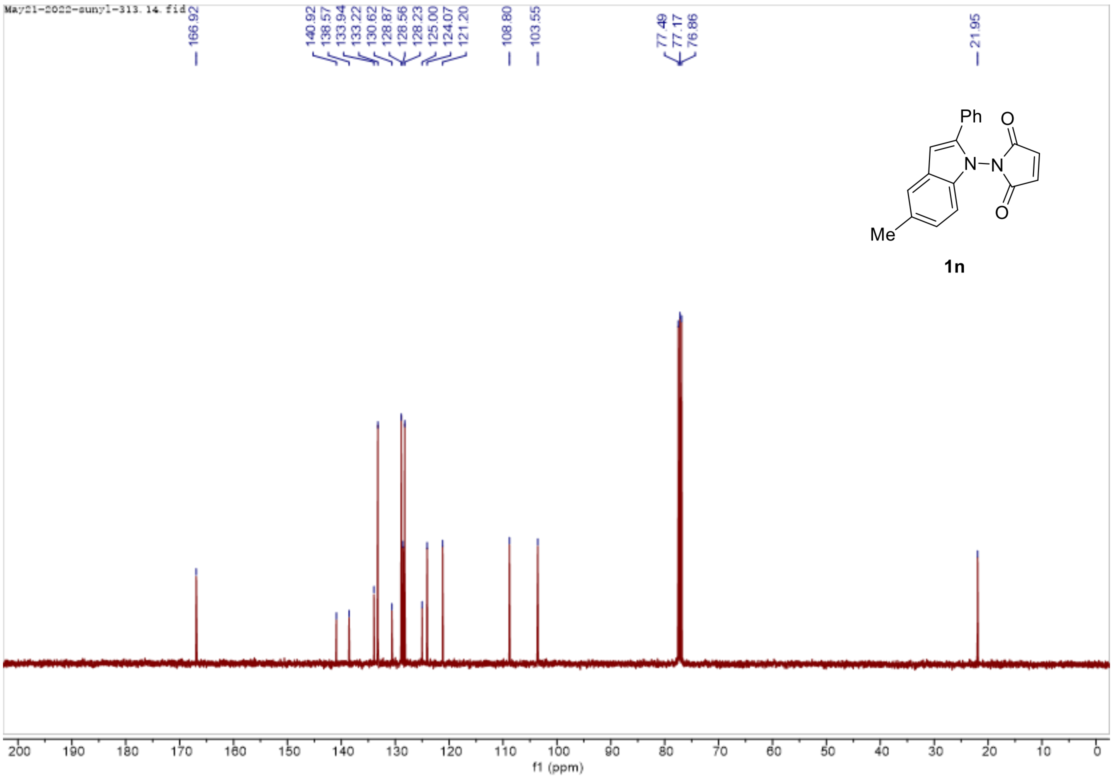
May21-2022-sunyl-313.14.fid
166.92
140.92
138.57
133.94
133.22
130.62
128.87
128.56
128.23
125.00
124.07
121.20
108.80
103.55
77.49
77.17
76.86
21.95
Ph
O
N–N
O
Me
1n
f1 (ppm)

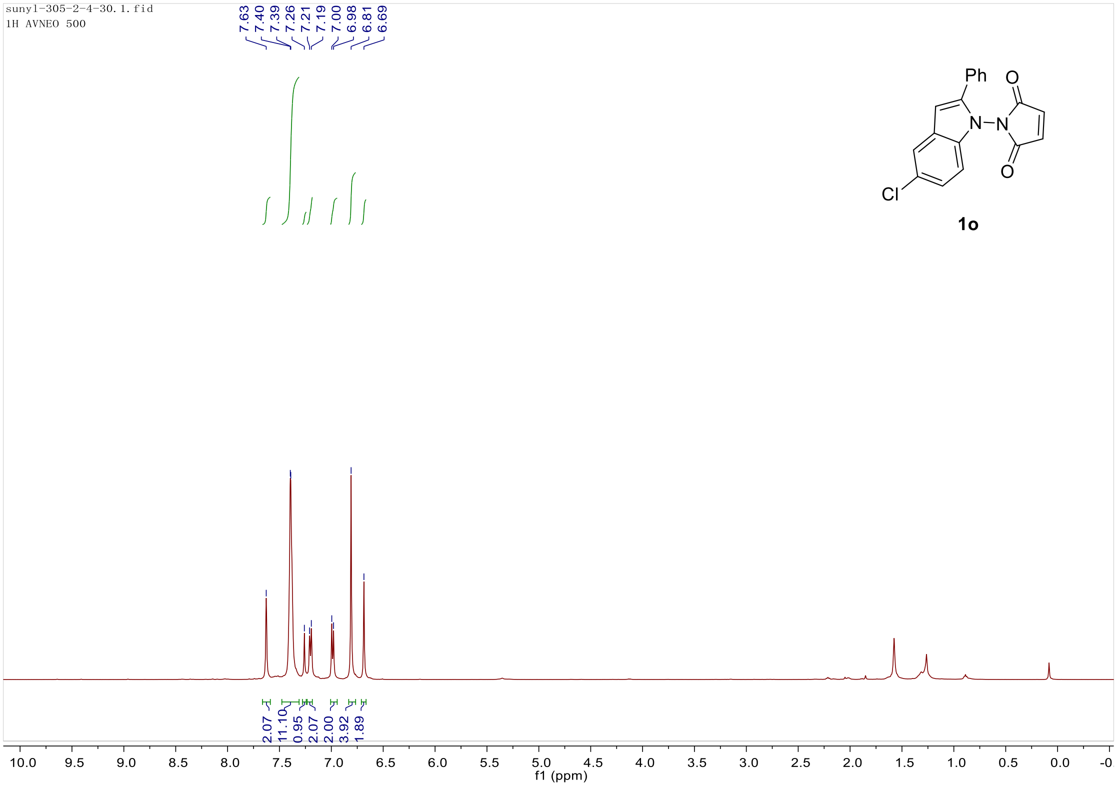
sunyl-305-2-4-30.1.fid
1H AVNEO 500
7.63
7.40
7.39
7.26
7.21
7.19
7.00
6.98
6.81
6.69
Ph
O
N–N
O
Cl
1o
2.07
11.10
0.95
2.07
2.00
3.92
1.89
f1 (ppm)

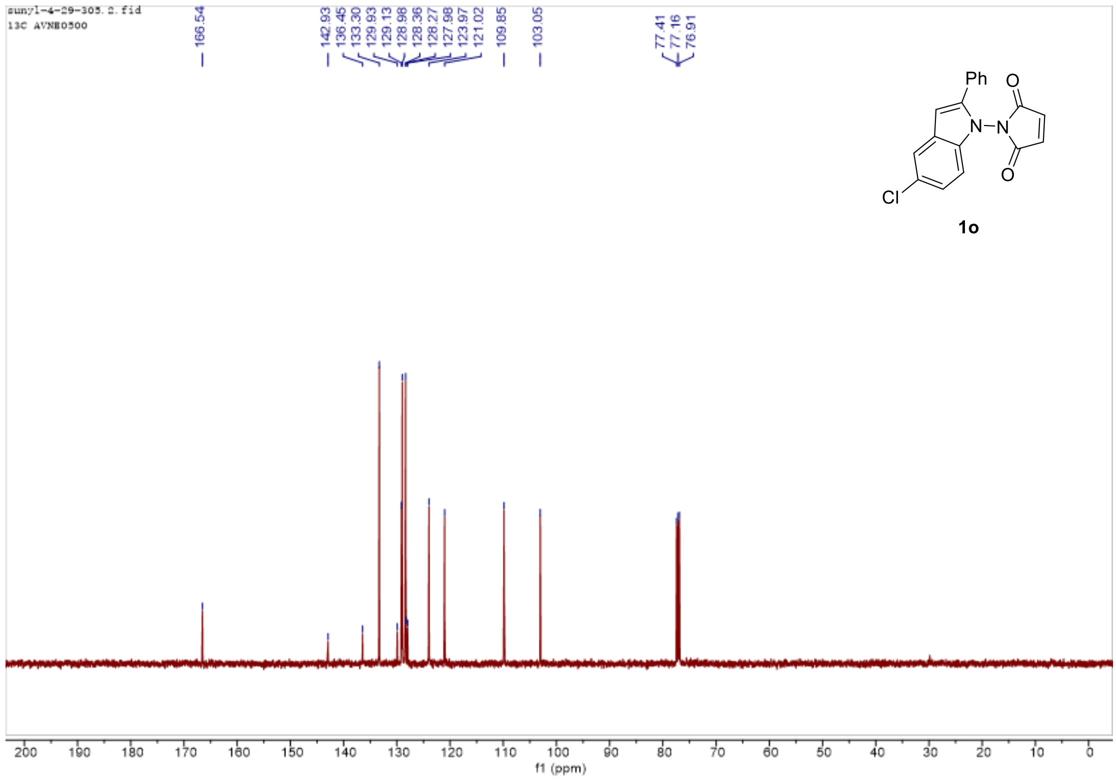

sunyl-4-29-305.2.fid
13C AVNEO500
166.54
142.93
136.45
133.30
129.93
129.13
128.98
128.36
128.27
127.98
123.97
121.02
109.85
103.05
77.41
77.16
76.91
Ph
O
N
N
O
Cl
200 190 180 170 160 150 140 130 120 110 100 90 80 70 60 50 40 30 20 10 0
f1 (ppm)

1o

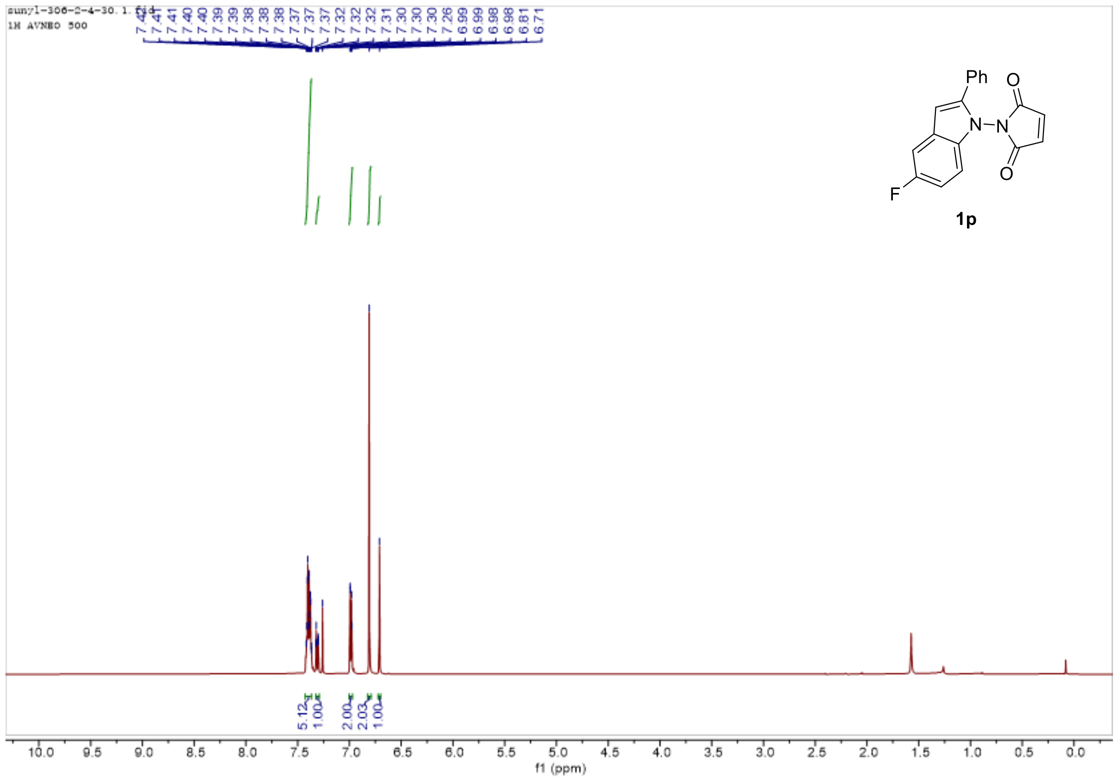

sunyl-306-2-4-30.1.fid
1H AVNEO 500
7.42
7.41
7.41
7.40
7.40
7.39
7.39
7.38
7.38
7.38
7.37
7.37
7.37
7.32
7.32
7.32
7.31
7.30
7.30
7.30
7.26
6.99
6.99
6.98
6.98
6.81
6.71
Ph
O
N
N
O
F
5.12
1.00
2.00
2.03
1.00
10.0 9.5 9.0 8.5 8.0 7.5 7.0 6.5 6.0 5.5 5.0 4.5 4.0 3.5 3.0 2.5 2.0 1.5 1.0 0.5 0.0
f1 (ppm)

1p

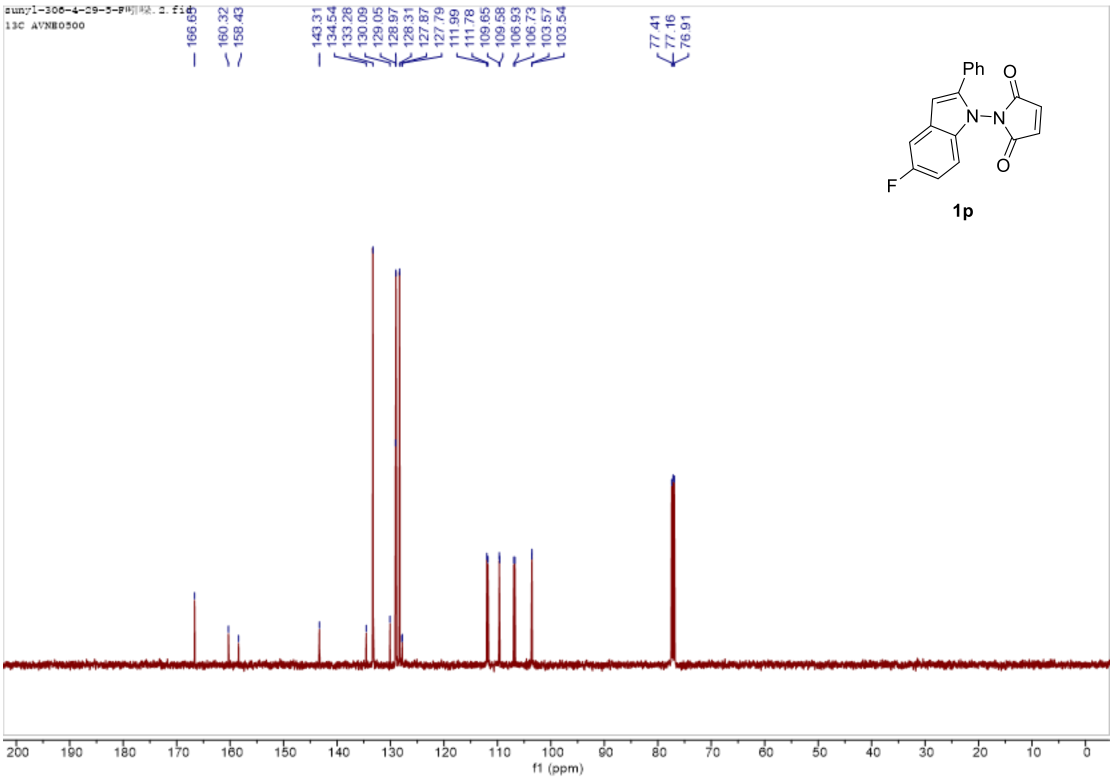
sunyl-306-4-29-5-F吲哚.2.fid
13C AVNEO500
166.65
160.32
158.43
143.31
134.54
133.28
130.09
129.05
128.97
128.31
127.87
127.79
111.99
111.78
109.65
109.58
106.93
106.73
103.57
103.54
77.41
77.16
76.91
Ph
O
N
N
O
F
200 190 180 170 160 150 140 130 120 110 100 90 80 70 60 50 40 30 20 10 0
f1 (ppm)

1p

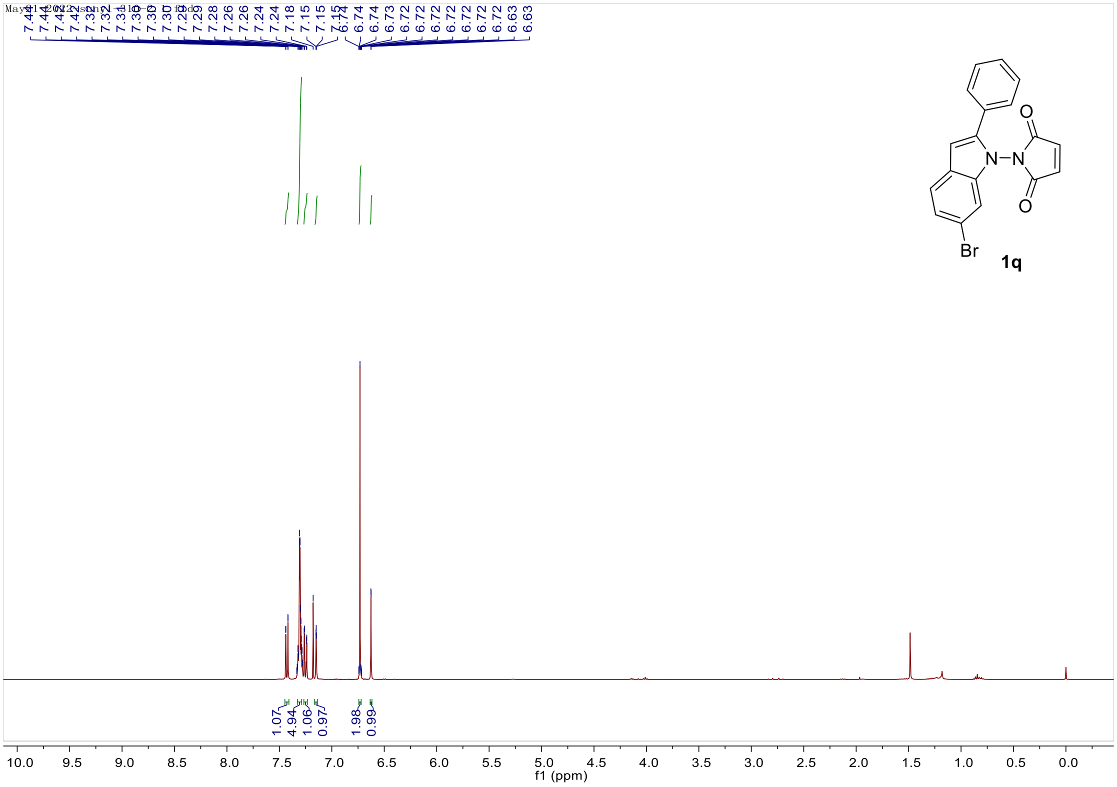
7.44
7.42
7.42
7.32
7.31
7.30
7.30
7.29
7.28
7.26
7.26
7.24
7.24
7.18
7.15
7.15
6.74
6.74
6.73
6.72
6.72
6.72
6.72
6.72
6.72
6.63
6.63
O
N
N
O
Br
1.07
4.94
1.06
0.97
1.98
0.99
10.0 9.5 9.0 8.5 8.0 7.5 7.0 6.5 6.0 5.5 5.0 4.5 4.0 3.5 3.0 2.5 2.0 1.5 1.0 0.5 0.0
f1 (ppm)

1q

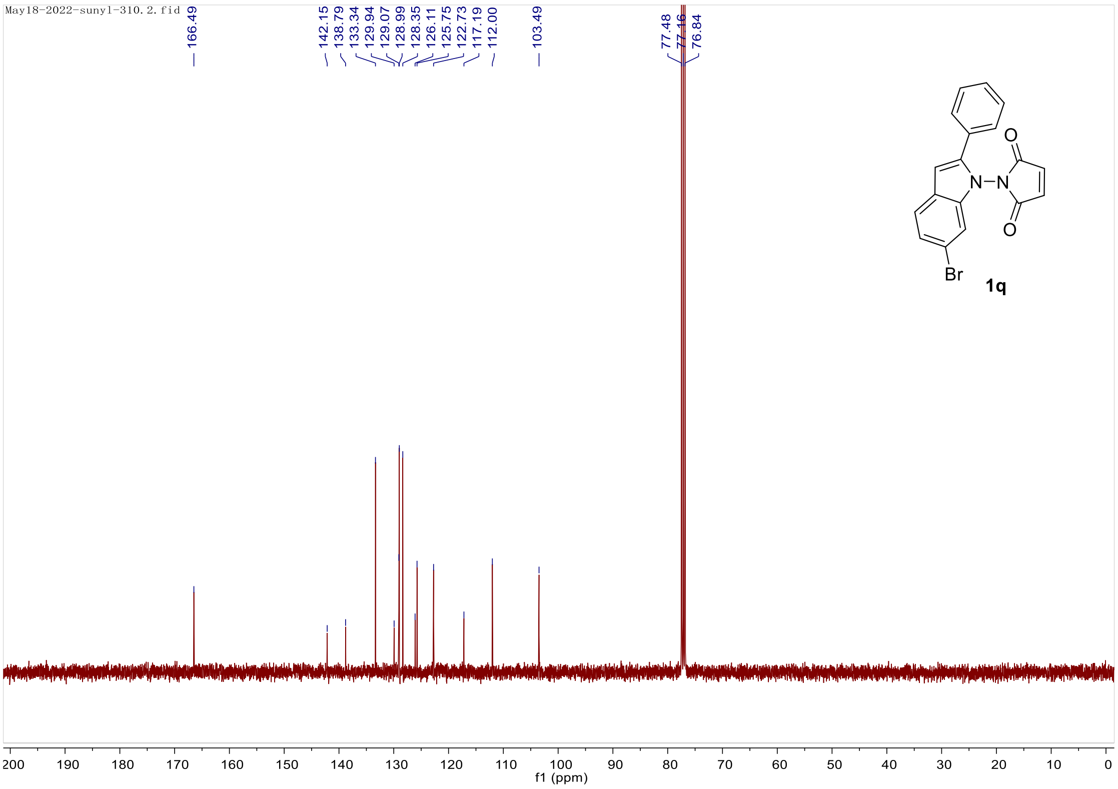
May18-2022-sunyl-310.2.fid
166.49
142.15
138.79
133.34
129.94
129.07
128.99
128.35
126.11
125.75
122.73
117.19
112.00
103.49
77.48
77.16
76.84
O
N
N
O
Br
1q
200
190
180
170
160
150
140
130
120
110
100
90
80
70
60
50
40
30
20
10
0
f1 (ppm)

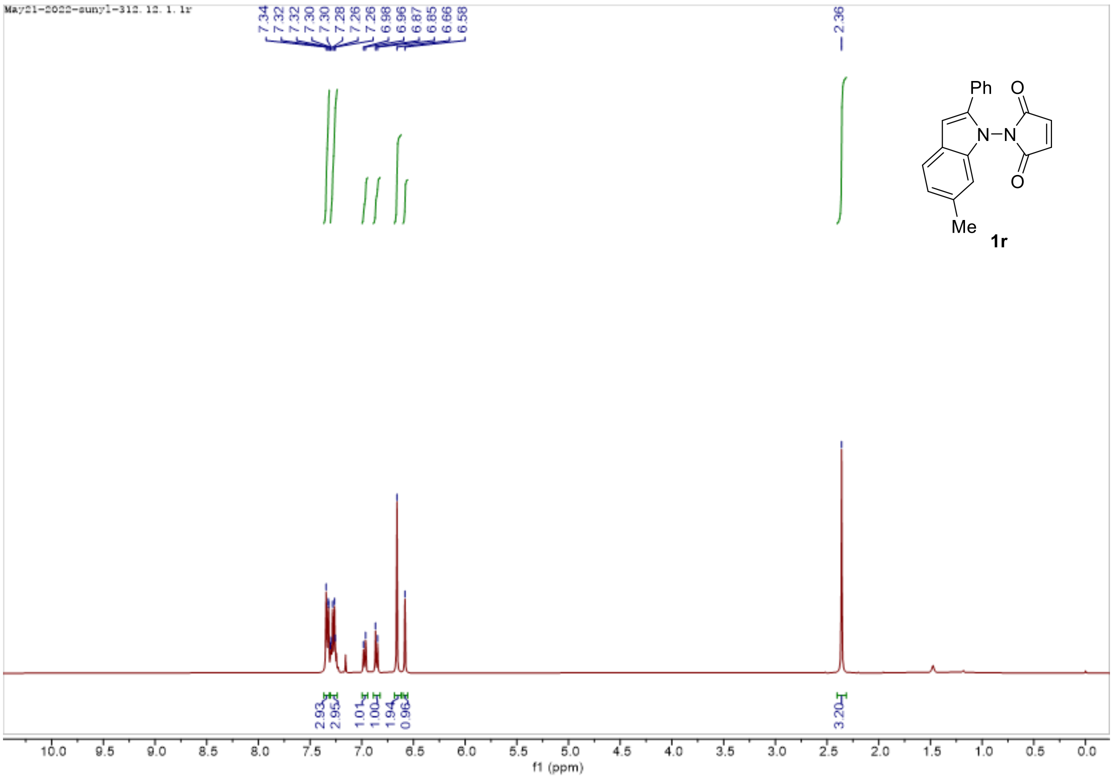
May21-2022-sunyl-312.12.1.1r
7.34
7.32
7.32
7.30
7.30
7.28
7.26
7.26
6.98
6.96
6.87
6.85
6.66
6.58
2.36
Ph
O
N
N
O
Me
1r
2.93
2.95
1.01
1.00
1.94
0.96
3.20
10.0
9.5
9.0
8.5
8.0
7.5
7.0
6.5
6.0
5.5
5.0
4.5
4.0
3.5
3.0
2.5
2.0
1.5
1.0
0.5
0.0
f1 (ppm)

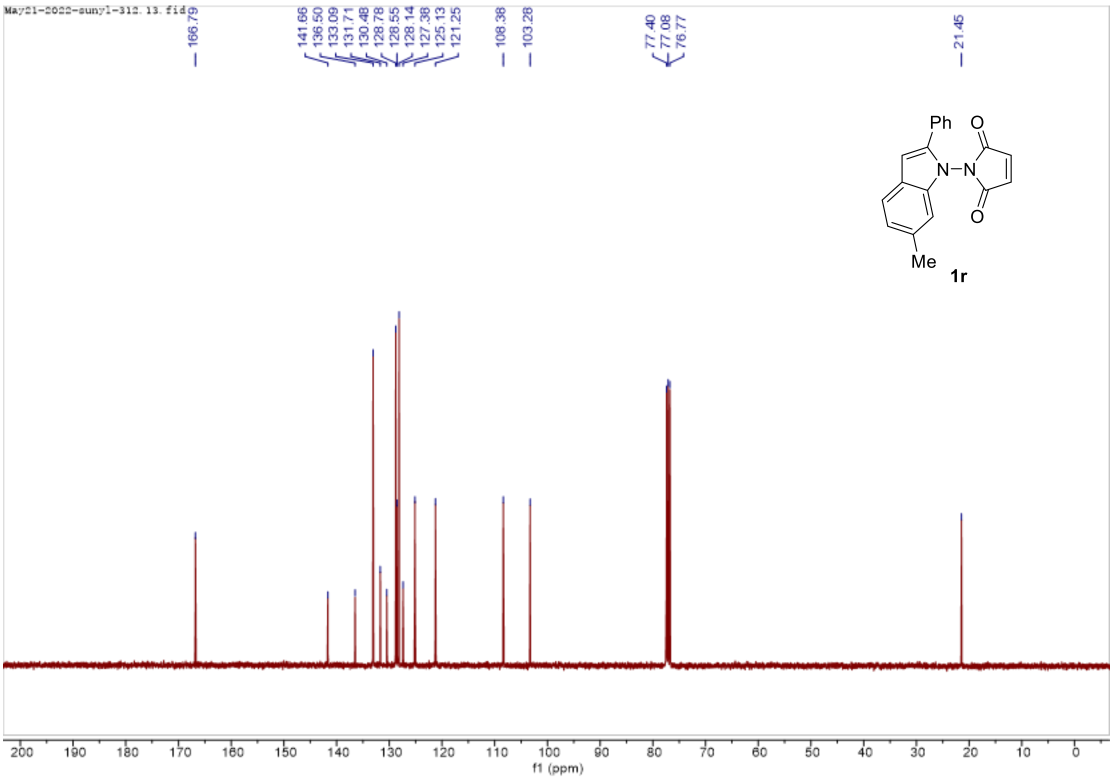
May21-2022-sunyl-312.13.fid
166.79
141.66
136.50
133.09
131.71
130.48
128.78
128.55
128.14
127.38
125.13
121.25
108.38
103.28
77.40
77.08
76.77
21.45
Ph
O
N
N
O
Me
1r
f1 (ppm)

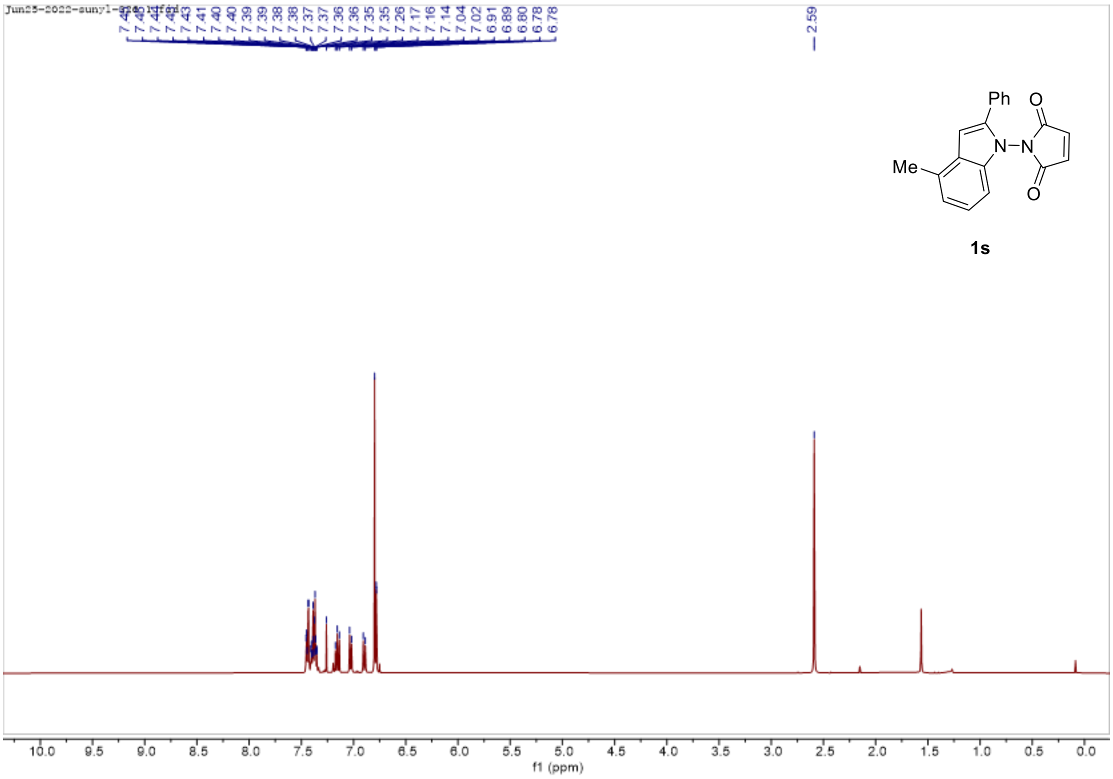
7.43
7.41
7.40
7.40
7.39
7.39
7.38
7.38
7.37
7.37
7.36
7.36
7.35
7.26
7.17
7.16
7.14
7.04
7.02
6.91
6.89
6.80
6.78
6.78
2.59
Ph
O
Me
N
N
O
1s
f1 (ppm)

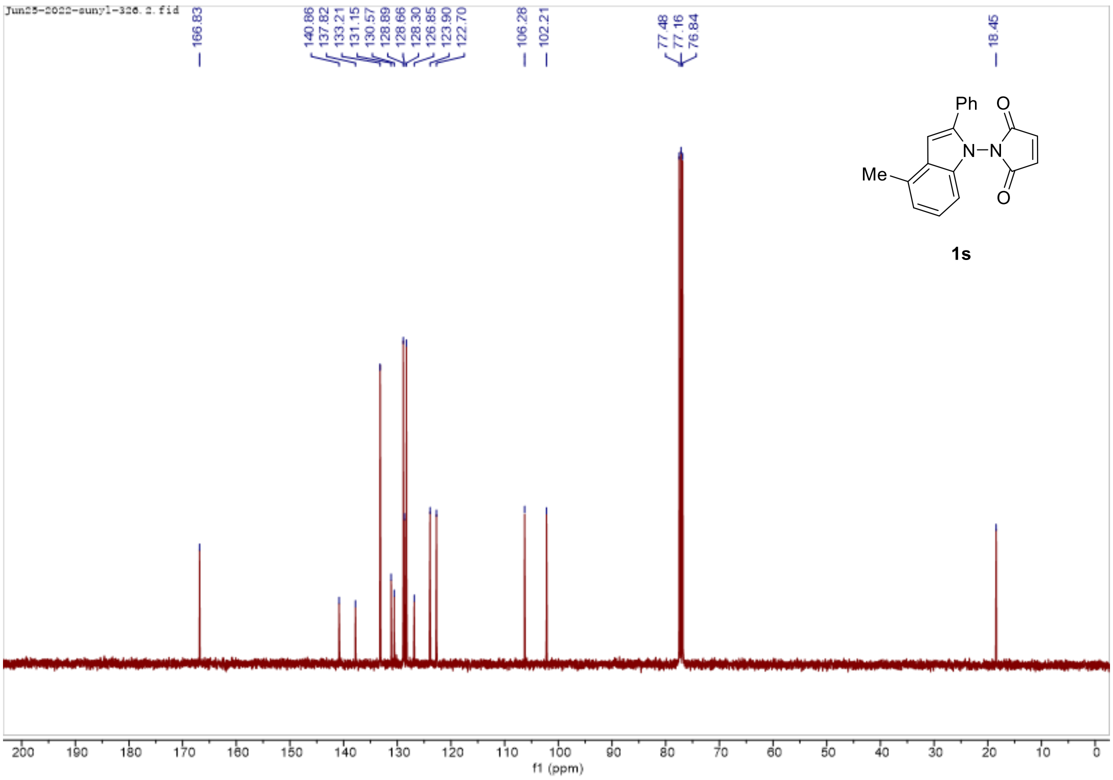
Jun25-2022-sunyl-326.2.fid
166.83
140.86
137.82
133.21
131.15
130.57
128.89
128.66
128.30
126.85
123.90
122.70
106.28
102.21
77.48
77.16
76.84
18.45
Ph
Me
N
O
1s
f1 (ppm)

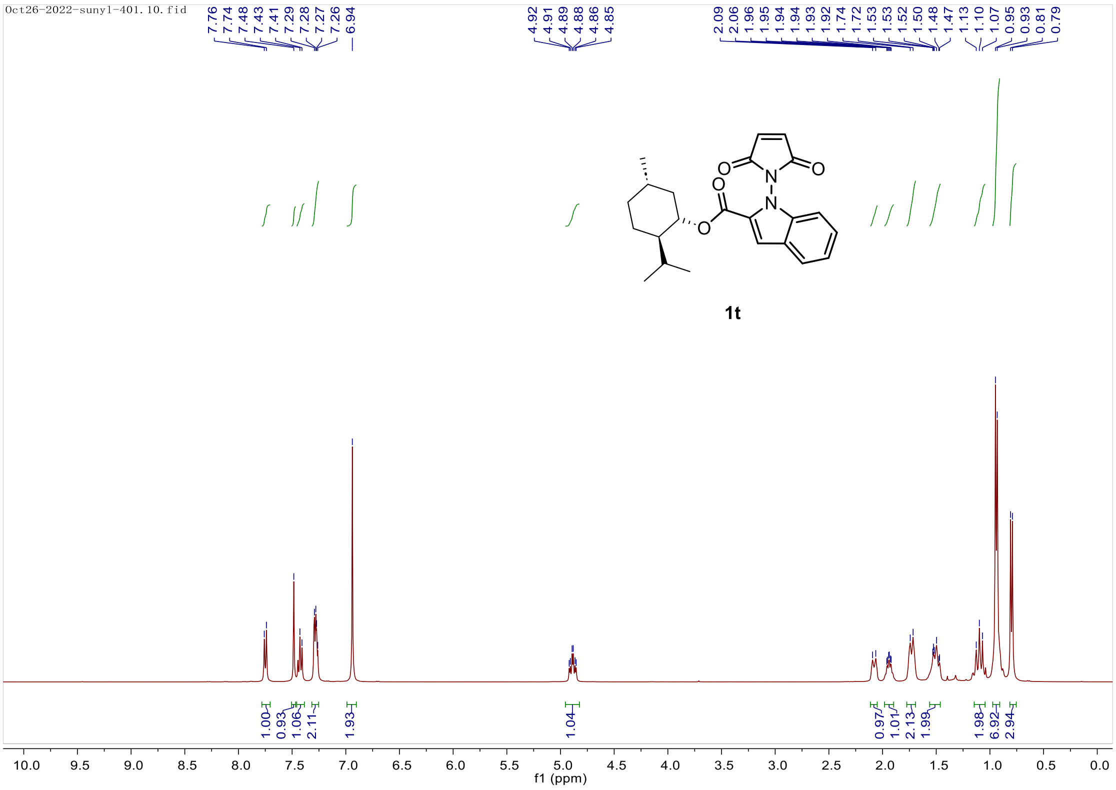
Oct26-2022-sunyl-401.10.fid
7.76
7.74
7.48
7.43
7.41
7.29
7.28
7.27
7.26
6.94
4.92
4.91
4.89
4.88
4.86
4.85
2.09
2.06
1.96
1.95
1.94
1.93
1.92
1.74
1.72
1.53
1.53
1.52
1.50
1.48
1.47
1.13
1.10
1.07
0.95
0.93
0.81
0.79
1t
1.00
0.93
1.06
2.11
1.93
1.04
0.97
1.01
2.13
1.99
1.98
6.92
2.94
f1 (ppm)

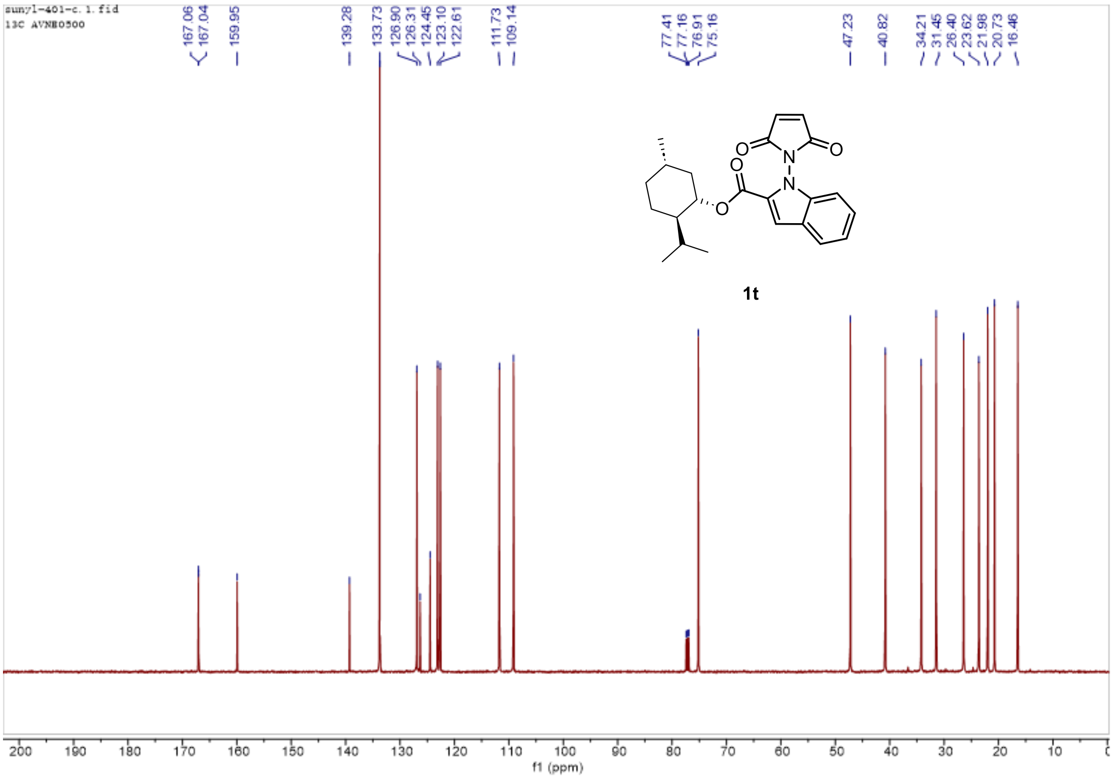

sunyl-401-c.1.fid
13C AVNEO500
167.06
167.04
159.95
139.28
133.73
126.90
126.31
124.45
123.10
122.61
111.73
109.14
77.41
77.16
76.91
75.16
47.23
40.82
34.21
31.45
26.40
23.62
21.98
20.73
16.46
1t
f1 (ppm)

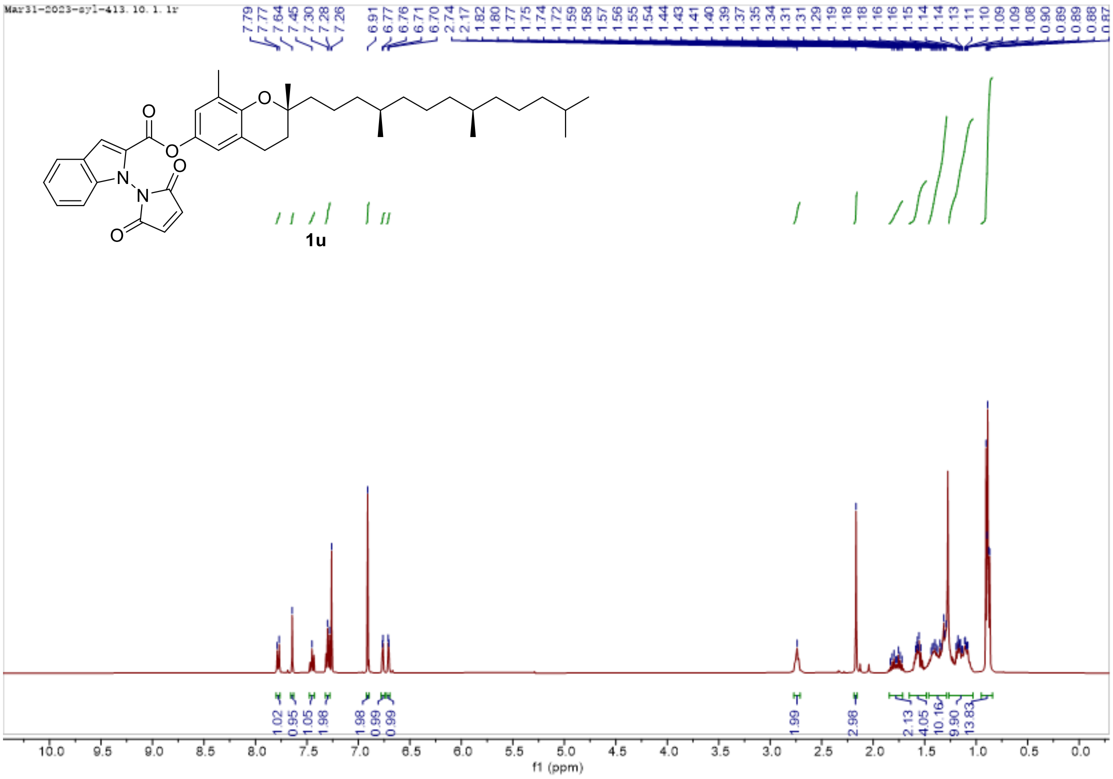

Mar31-2023-syl-413.10.1.1r
1u
1.02
0.95
1.05
1.98
1.98
0.99
0.99
1.99
2.98
2.13
4.05
10.16
9.90
13.83
f1 (ppm)

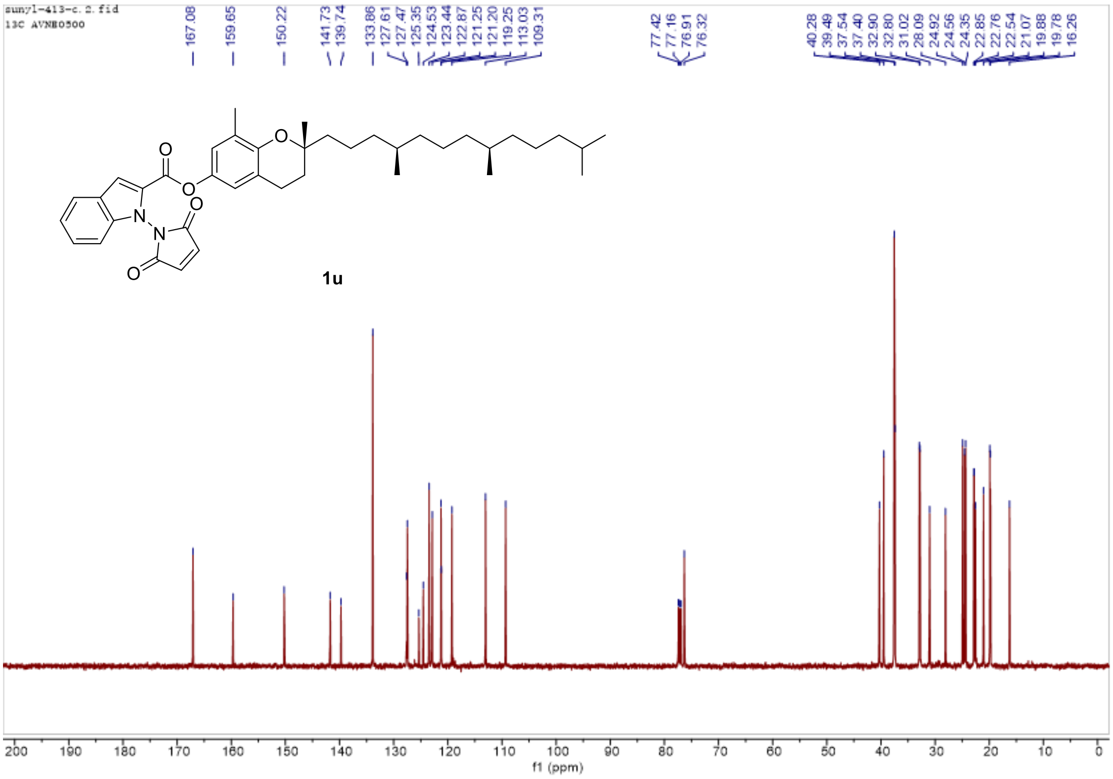
sunyl-413-c.2.fid
13C AVNEO500
167.08
159.65
150.22
141.73
139.74
133.86
127.61
127.47
125.35
124.53
123.44
122.87
121.25
121.20
119.25
113.03
109.31
77.42
77.16
76.91
76.32
40.28
39.49
37.54
37.40
32.90
32.80
31.02
28.09
24.92
24.56
24.35
22.85
22.76
22.54
21.07
19.88
19.78
16.26
1u
f1 (ppm)

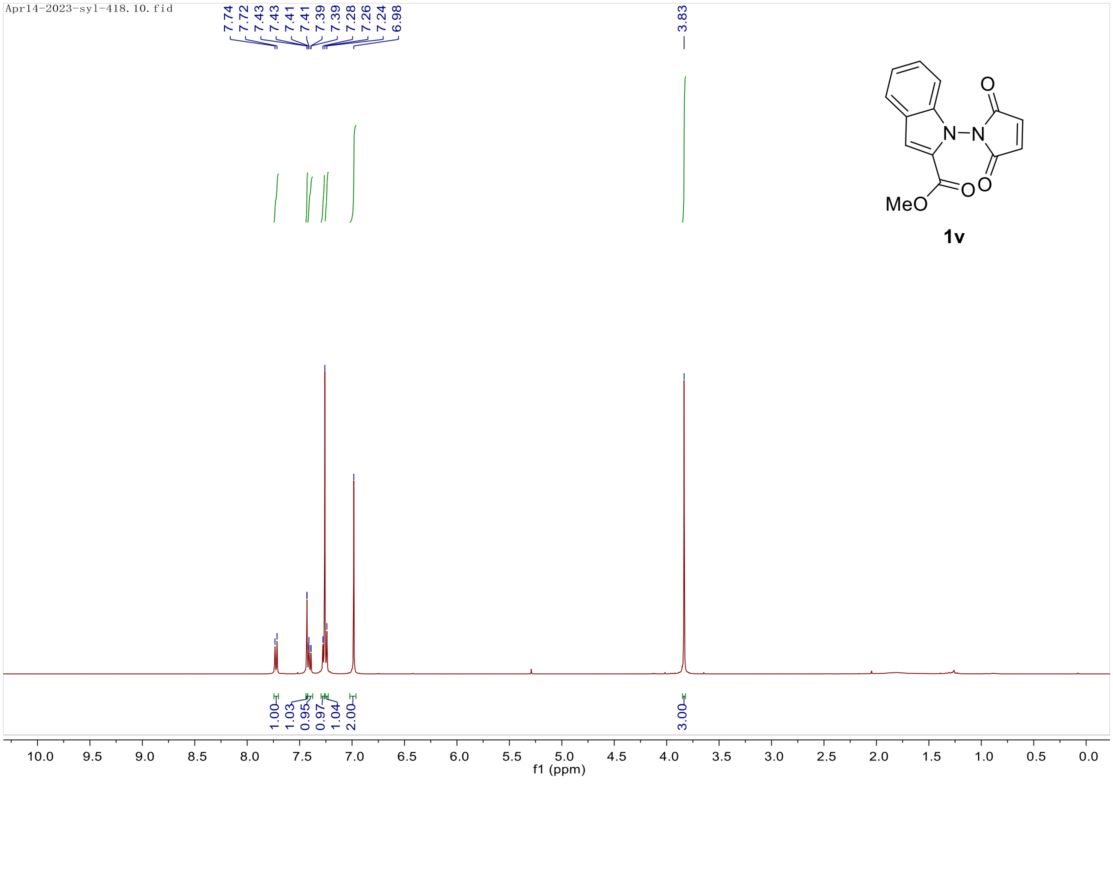
Apr14-2023-syl-418.10.fid
7.74
7.72
7.43
7.43
7.41
7.41
7.39
7.28
7.26
7.24
6.98
3.83
MeO
1v
1.00
1.03
0.95
0.97
1.04
2.00
3.00
f1 (ppm)

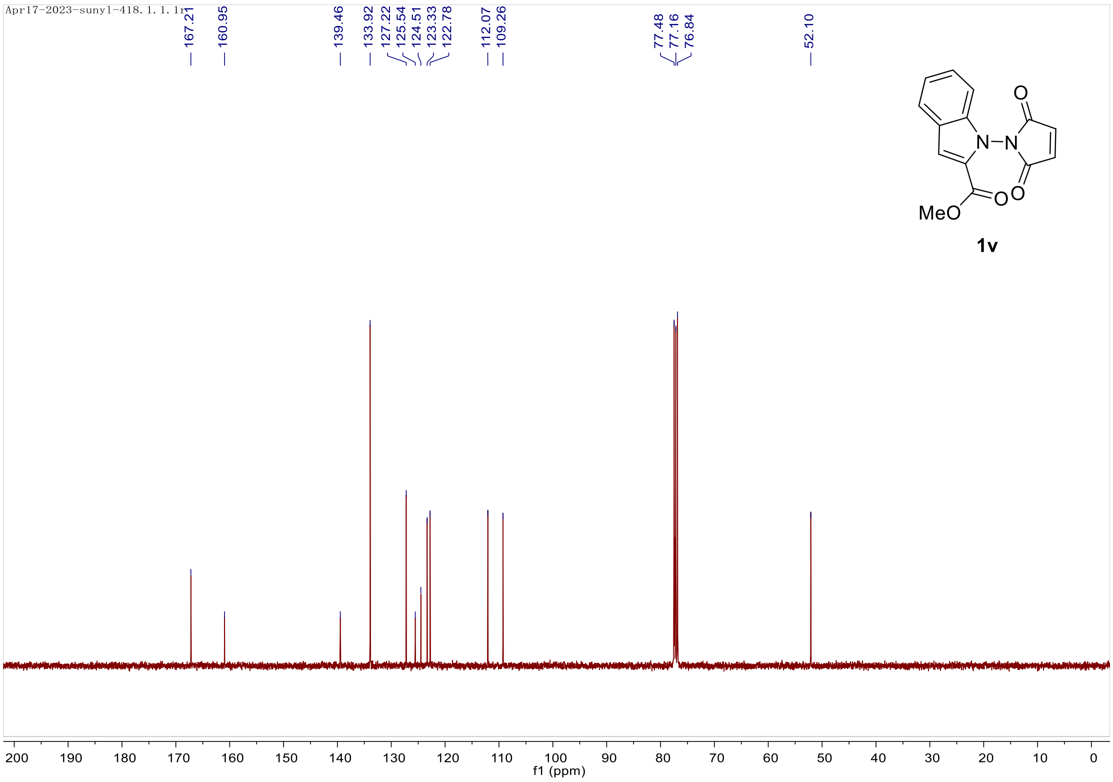
Apr17-2023-sunyl-418.1.1.1r
167.21
160.95
139.46
133.92
127.22
125.54
124.51
123.33
122.78
112.07
109.26
77.48
77.16
76.84
52.10
MeO
O
N
N
O
O
1v
200 190 180 170 160 150 140 130 120 110 100 90 80 70 60 50 40 30 20 10 0
f1 (ppm)

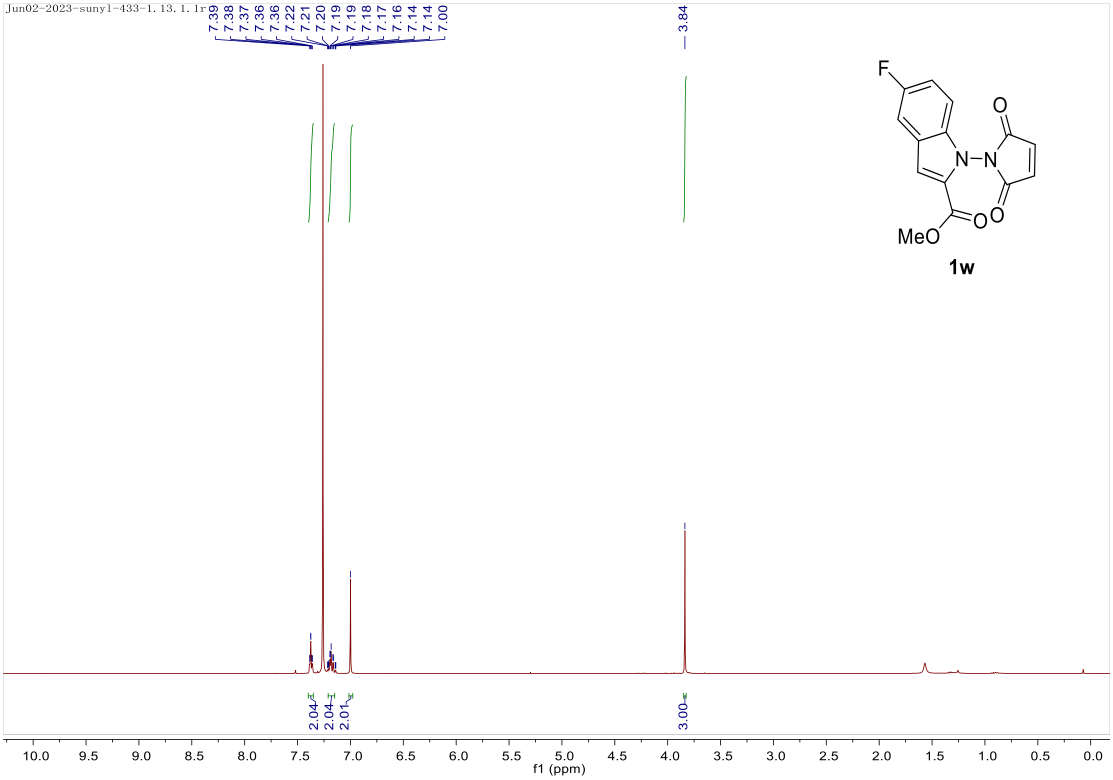
Jun02-2023-sunyl-433-1.13.1.1r
7.39
7.38
7.37
7.36
7.36
7.22
7.21
7.20
7.19
7.19
7.18
7.17
7.16
7.14
7.14
7.00
3.84
F
MeO
O
N
N
O
O
1w
2.04
2.04
2.01
3.00
10.0 9.5 9.0 8.5 8.0 7.5 7.0 6.5 6.0 5.5 5.0 4.5 4.0 3.5 3.0 2.5 2.0 1.5 1.0 0.5 0.0
f1 (ppm)

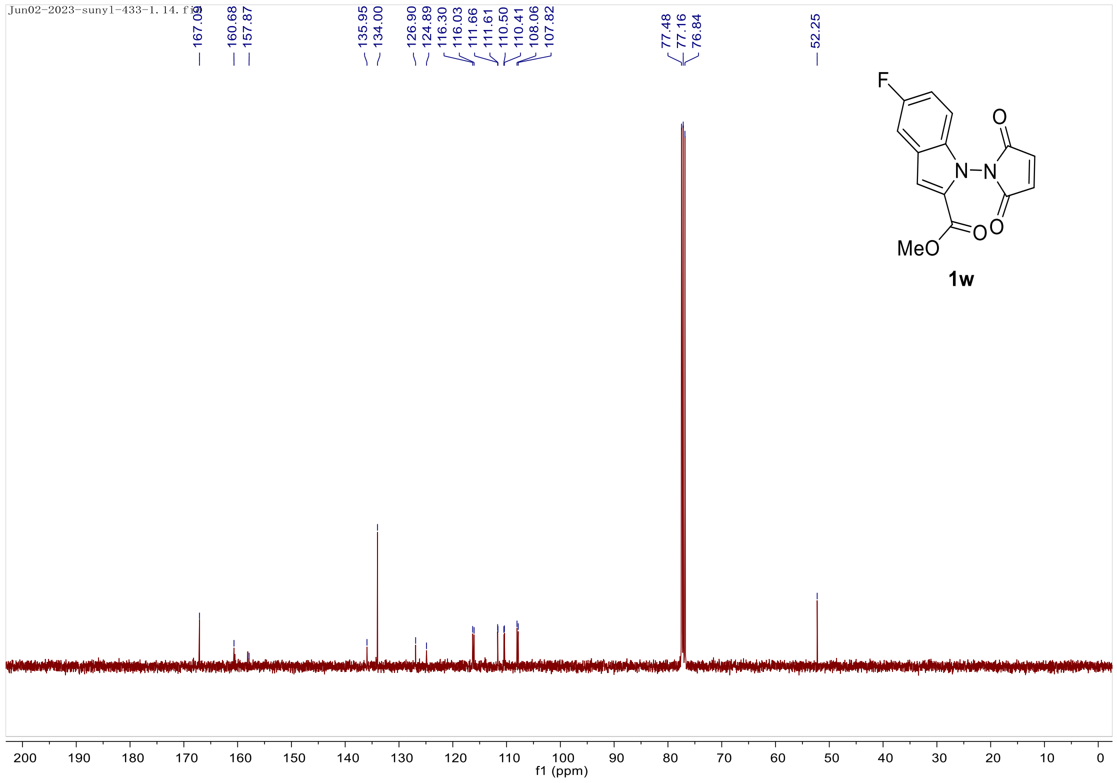
Jun02-2023-sunyl-433-1.14.fid
167.05
160.68
157.87
135.95
134.00
126.90
124.89
116.30
116.03
111.66
111.61
110.50
110.41
108.06
107.82
77.48
77.16
76.84
52.25
F
N
N
O
O
MeO
O
1w
f1 (ppm)

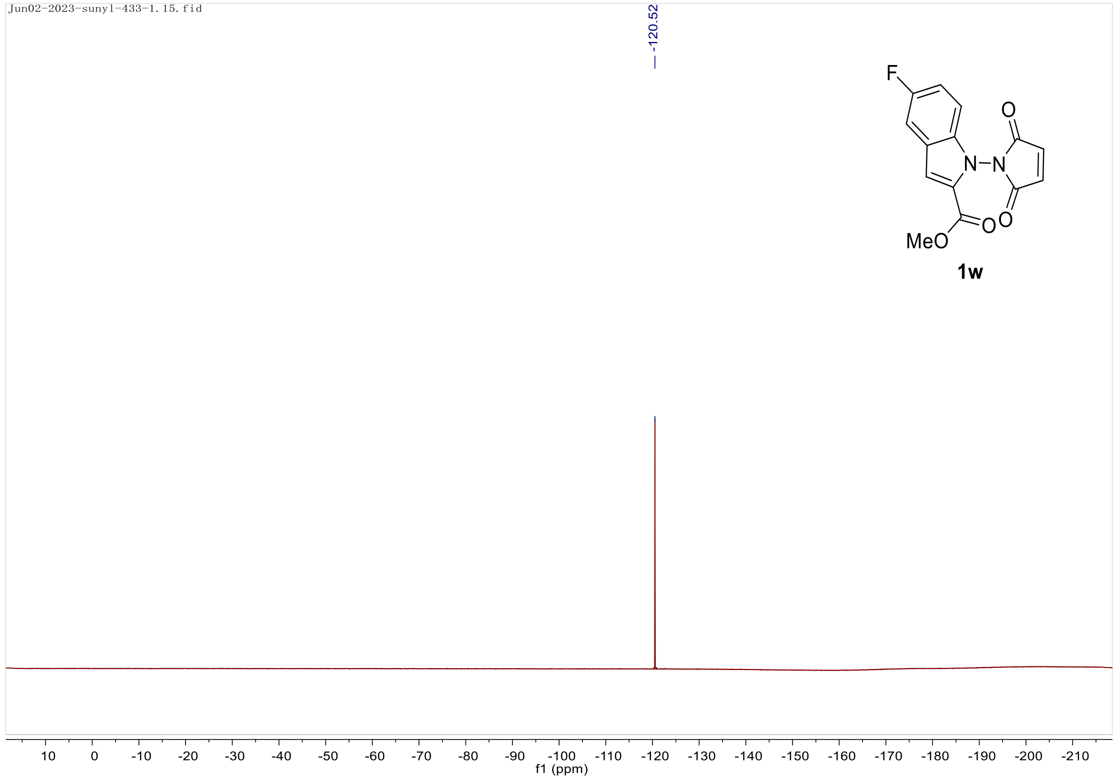
Jun02-2023-sunyl-433-1.15.fid
-120.52
F
N
N
O
O
MeO
O
1w
f1 (ppm)

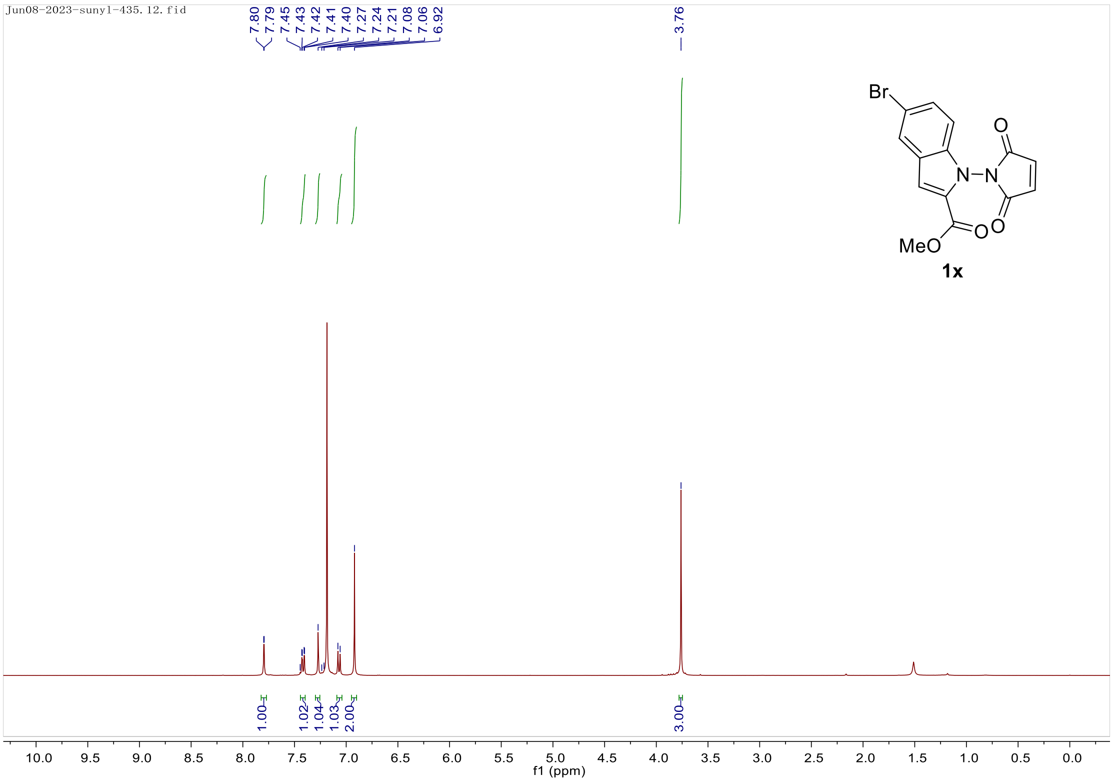
Jun08-2023-sunyl-435.12.fid
7.80
7.79
7.45
7.43
7.42
7.41
7.40
7.27
7.24
7.21
7.08
7.06
6.92
3.76
Br
N
N
O
O
O
MeO
1x
1.00
1.02
1.04
1.03
2.00
3.00
f1 (ppm)

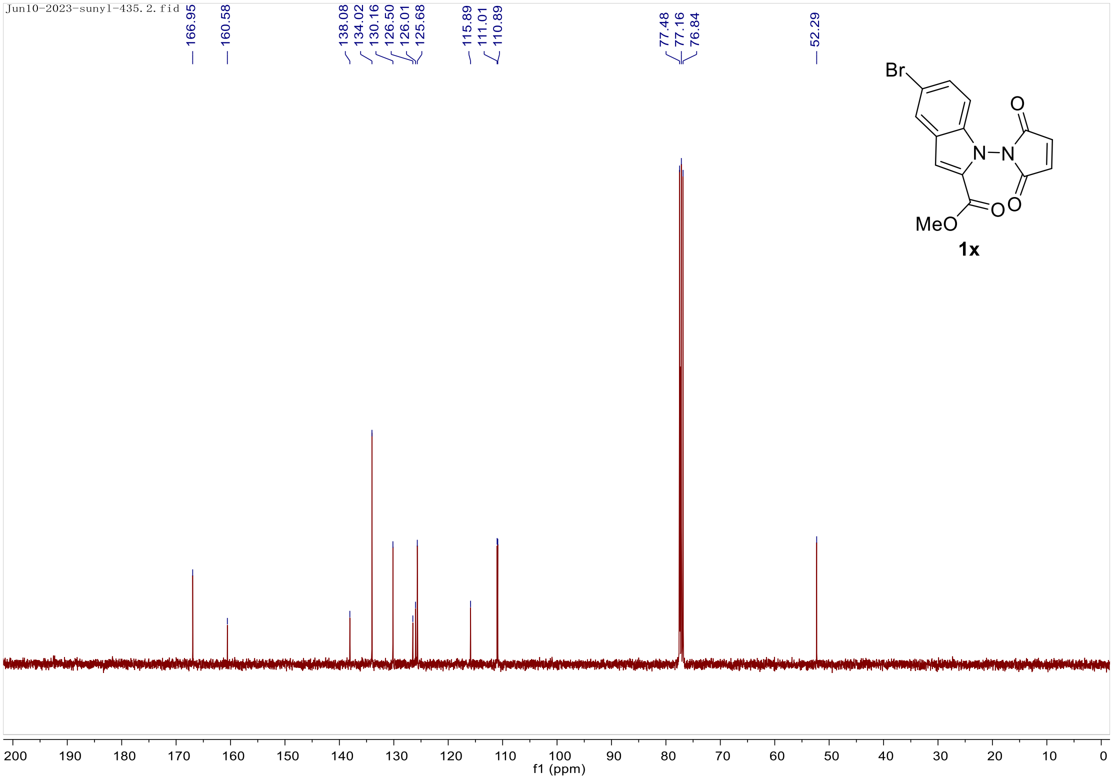
Jun10-2023-sunyl-435.2.fid
166.95
160.58
138.08
134.02
130.16
126.50
126.01
125.68
115.89
111.01
110.89
77.48
77.16
76.84
52.29
Br
N
N
O
O
O
MeO
1x
f1 (ppm)

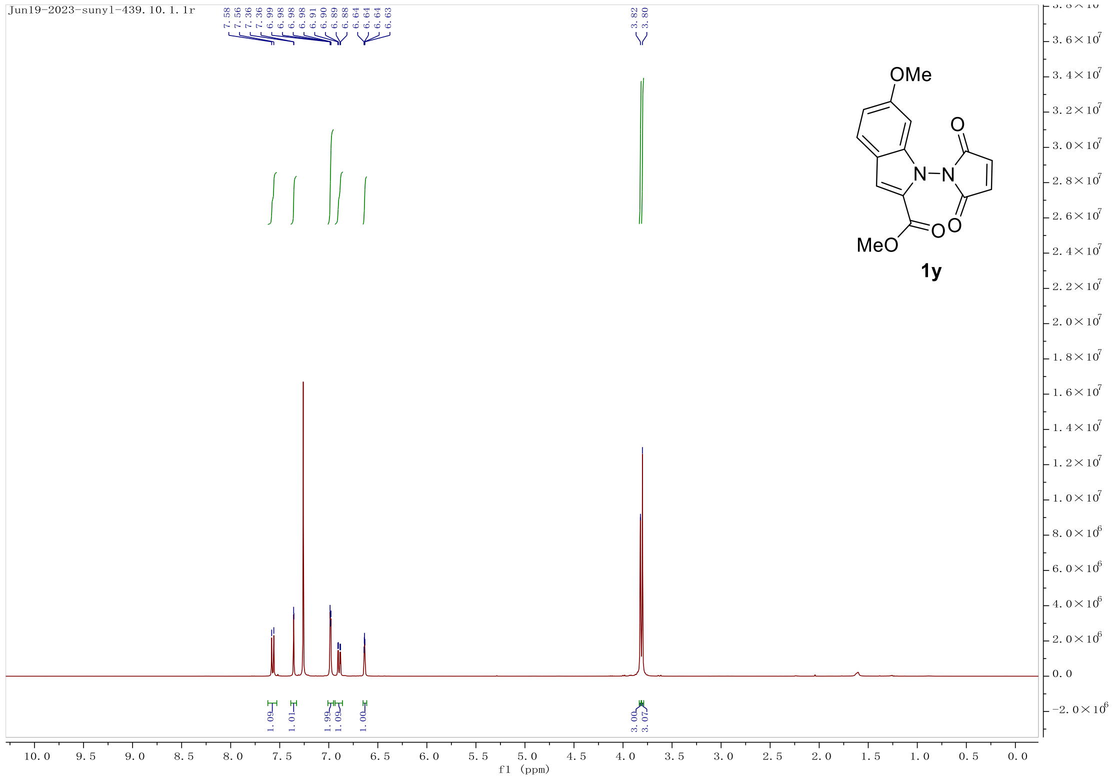

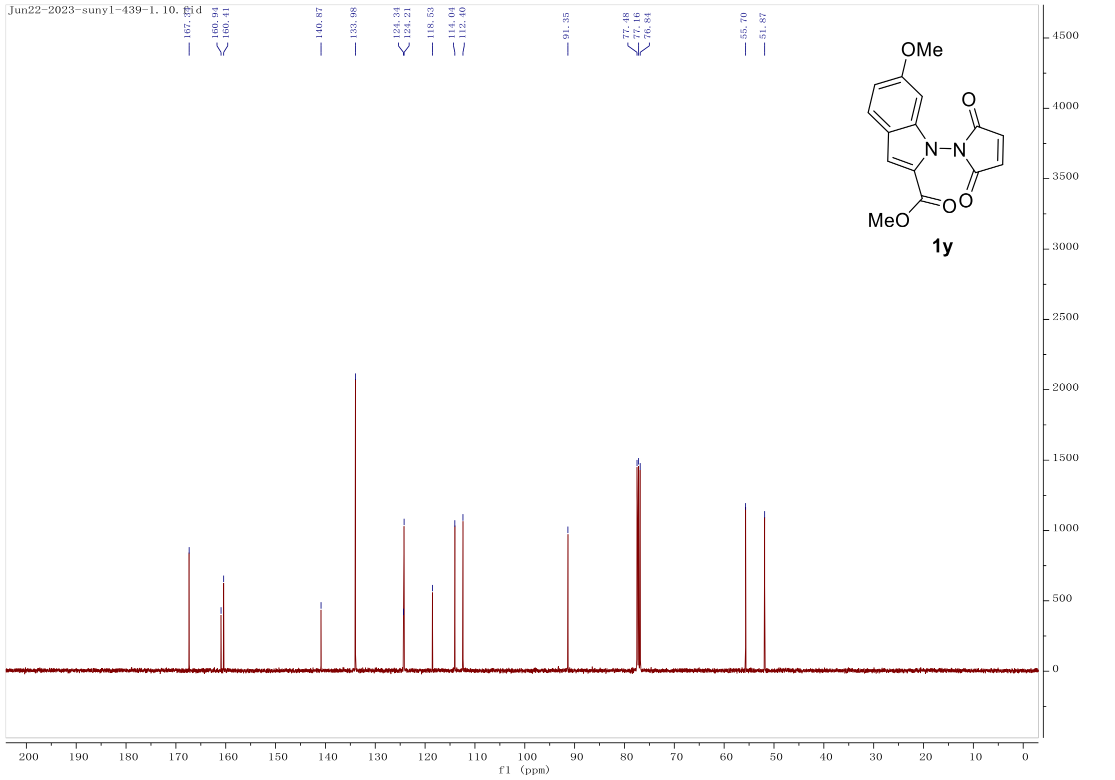

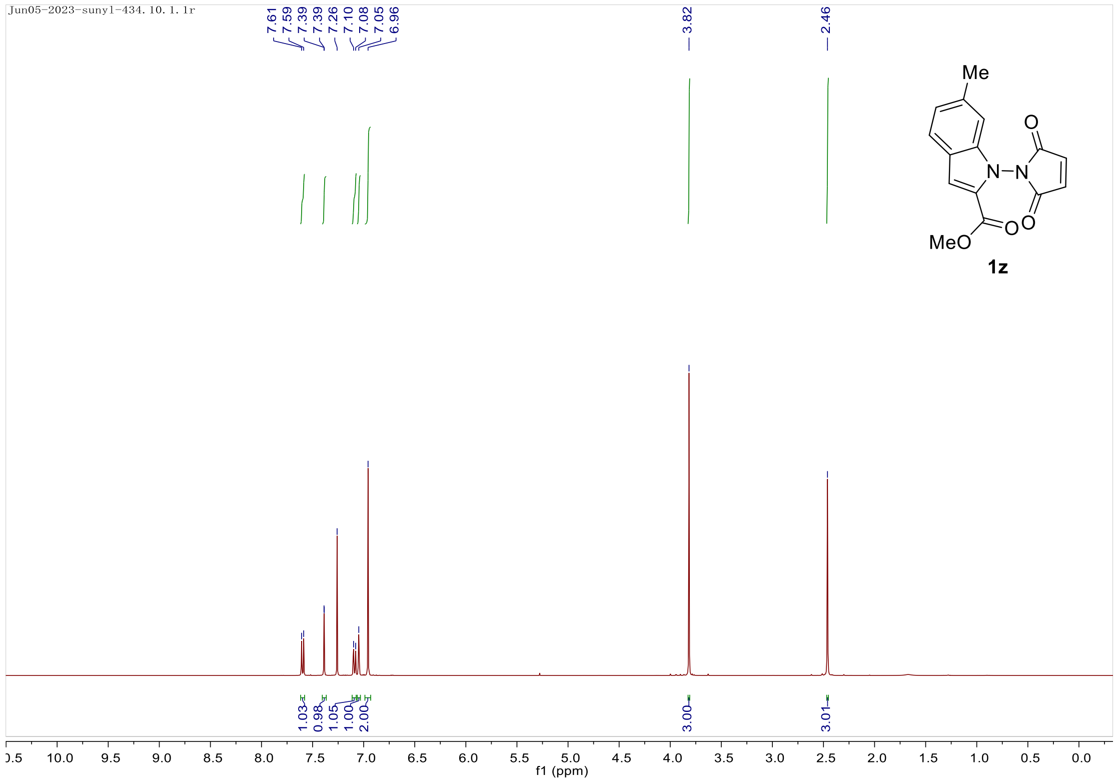
Jun05-2023-sunyl-434.10.1.1r
7.61
7.59
7.39
7.39
7.26
7.10
7.08
7.05
6.96
3.82
2.46
Me
O
N
N
O
O
MeO
1z
1.03
0.98
1.05
1.00
2.00
3.00
3.01
f1 (ppm)

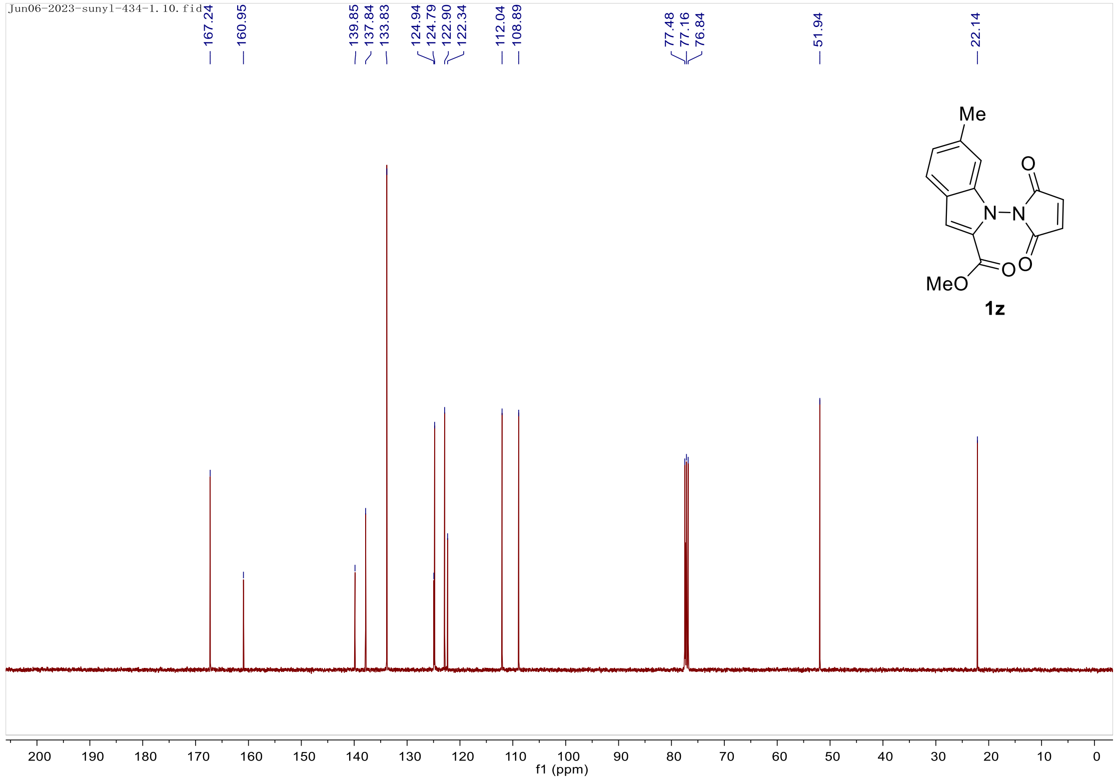
Jun06-2023-sunyl-434-1.10.fid
167.24
160.95
139.85
137.84
133.83
124.94
124.79
122.90
122.34
112.04
108.89
77.48
77.16
76.84
51.94
22.14
Me
O
N
N
O
O
MeO
1z
f1 (ppm)

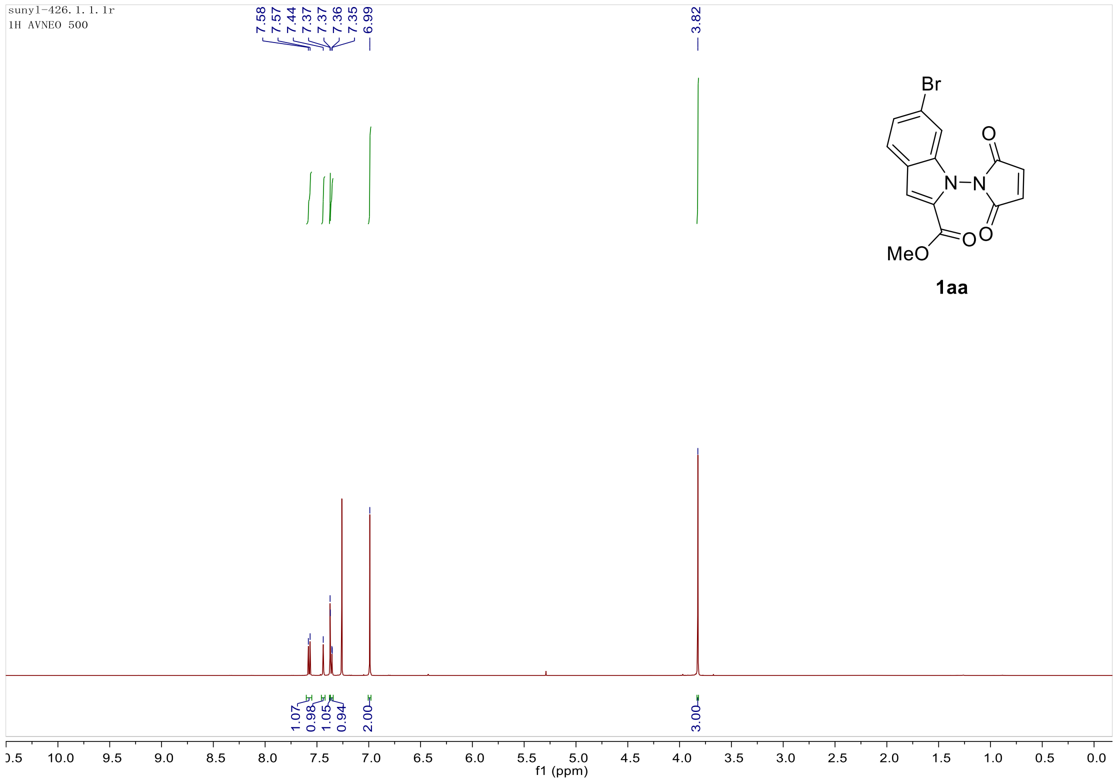
sunyl-426.1.1.1r
1H AVNEO 500
7.58
7.57
7.44
7.37
7.37
7.36
7.35
6.99
3.82
Br
N–N
O
O
MeO
O
1aa
1.07
0.98
1.05
0.94
2.00
3.00
f1 (ppm)

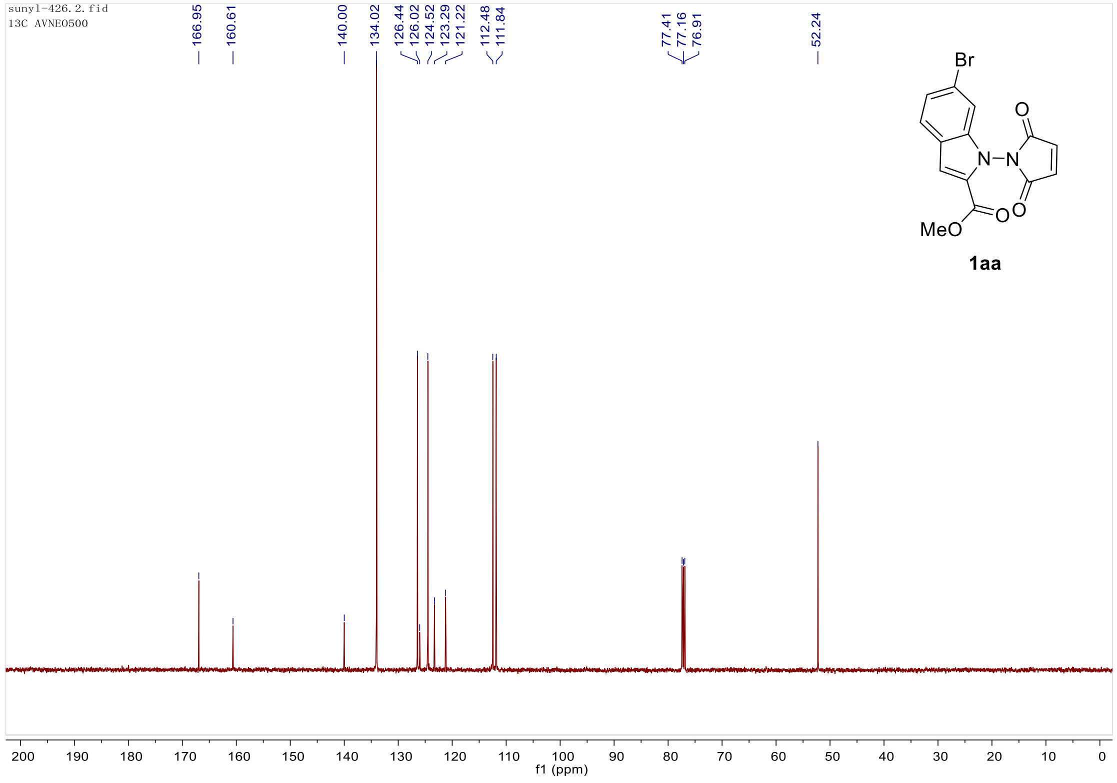
sunyl-426.2.fid
13C AVNEO500
166.95
160.61
140.00
134.02
126.44
126.02
124.52
123.29
121.22
112.48
111.84
77.41
77.16
76.91
52.24
Br
N–N
O
O
MeO
O
1aa
f1 (ppm)

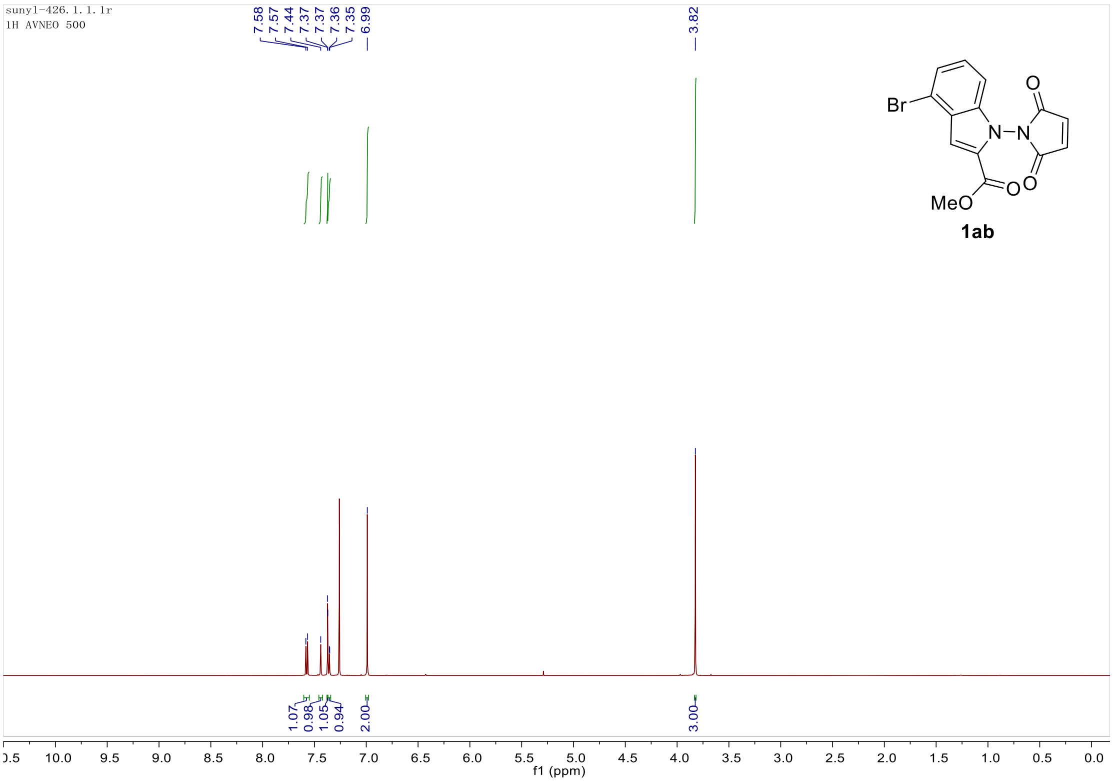
sunyl-426.1.1.1r
1H AVNEO 500
7.58
7.57
7.44
7.37
7.37
7.36
7.35
6.99
3.82
Br
N
N
O
O
MeO
O
1ab
1.07
0.98
1.05
0.94
2.00
3.00
f1 (ppm)

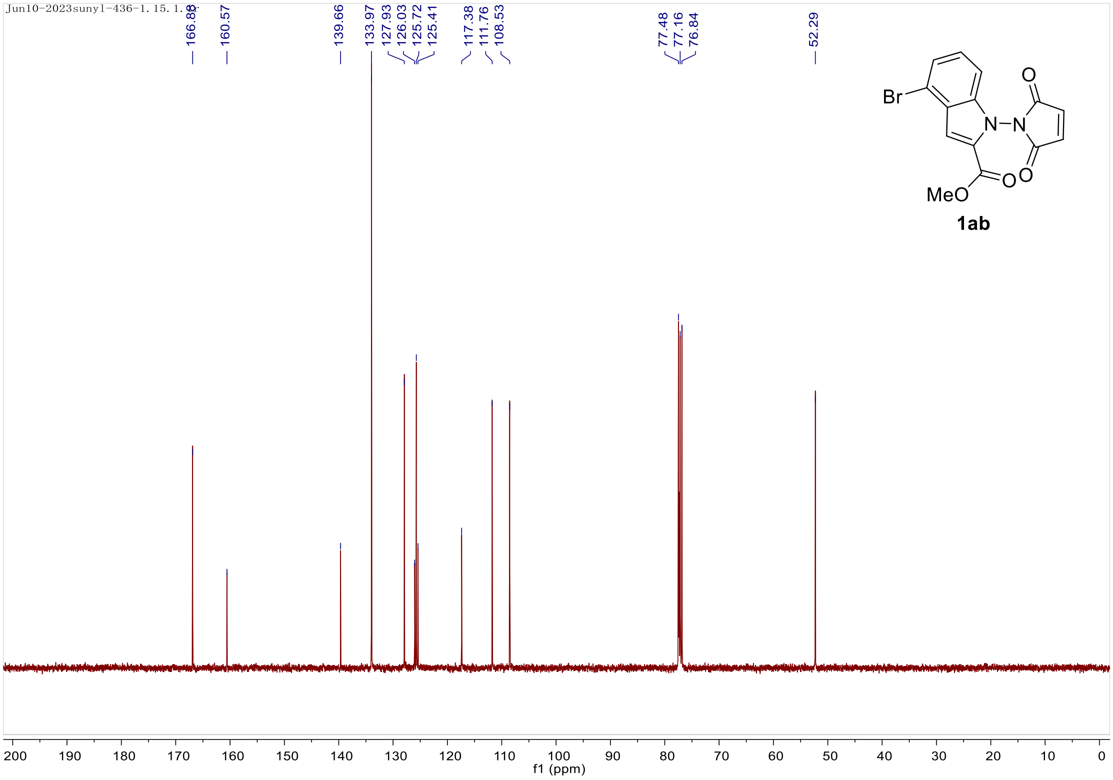
160.57
139.66
133.97
127.93
126.03
125.72
125.41
117.38
111.76
108.53
77.48
77.16
76.84
52.29
Br
N
N
O
O
MeO
O
1ab
f1 (ppm)

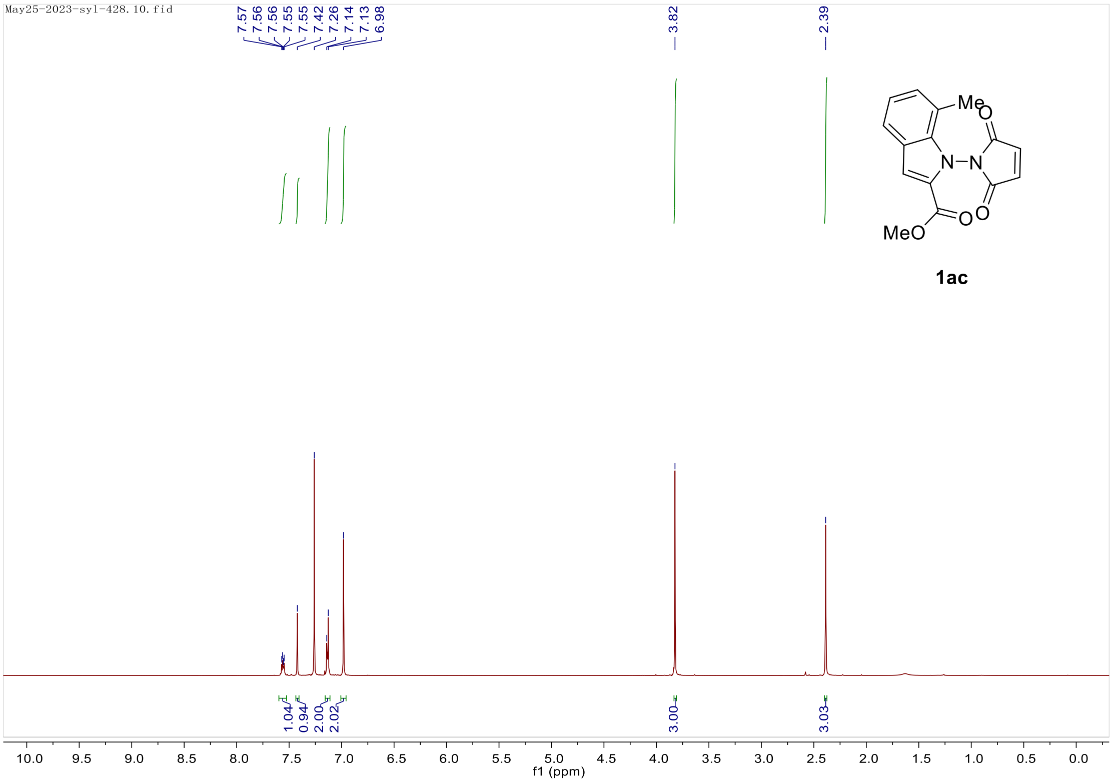

May25-2023-syl-428.10.fid
7.57
7.56
7.56
7.55
7.55
7.42
7.26
7.14
7.13
6.98
3.82
2.39
Me
O
N
N
O
O
MeO
1ac
1.04
0.94
2.00
2.02
3.00
3.03
10.0
9.5
9.0
8.5
8.0
7.5
7.0
6.5
6.0
5.5
5.0
4.5
4.0
3.5
3.0
2.5
2.0
1.5
1.0
0.5
0.0
f1 (ppm)

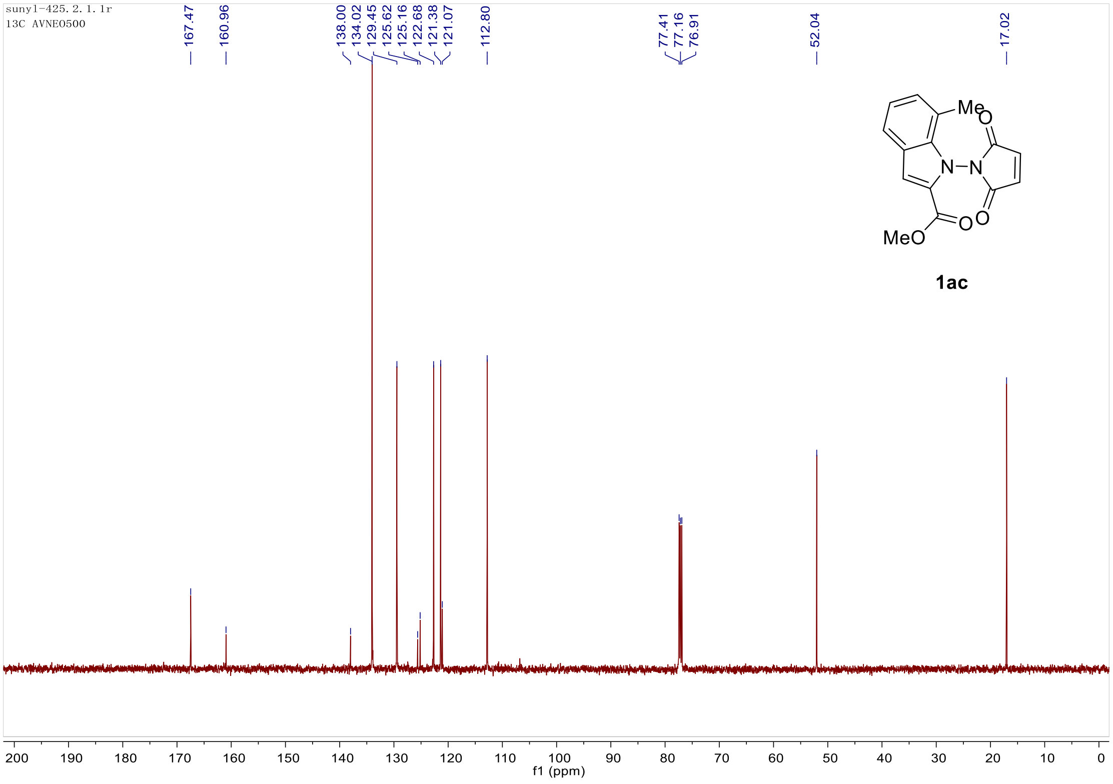

sunyl-425.2.1.1r
13C AVNEO500
167.47
160.96
138.00
134.02
129.45
125.62
125.16
122.68
121.38
121.07
112.80
77.41
77.16
76.91
52.04
17.02
Me
O
N
N
O
O
MeO
1ac
200
190
180
170
160
150
140
130
120
110
100
90
80
70
60
50
40
30
20
10
0
f1 (ppm)

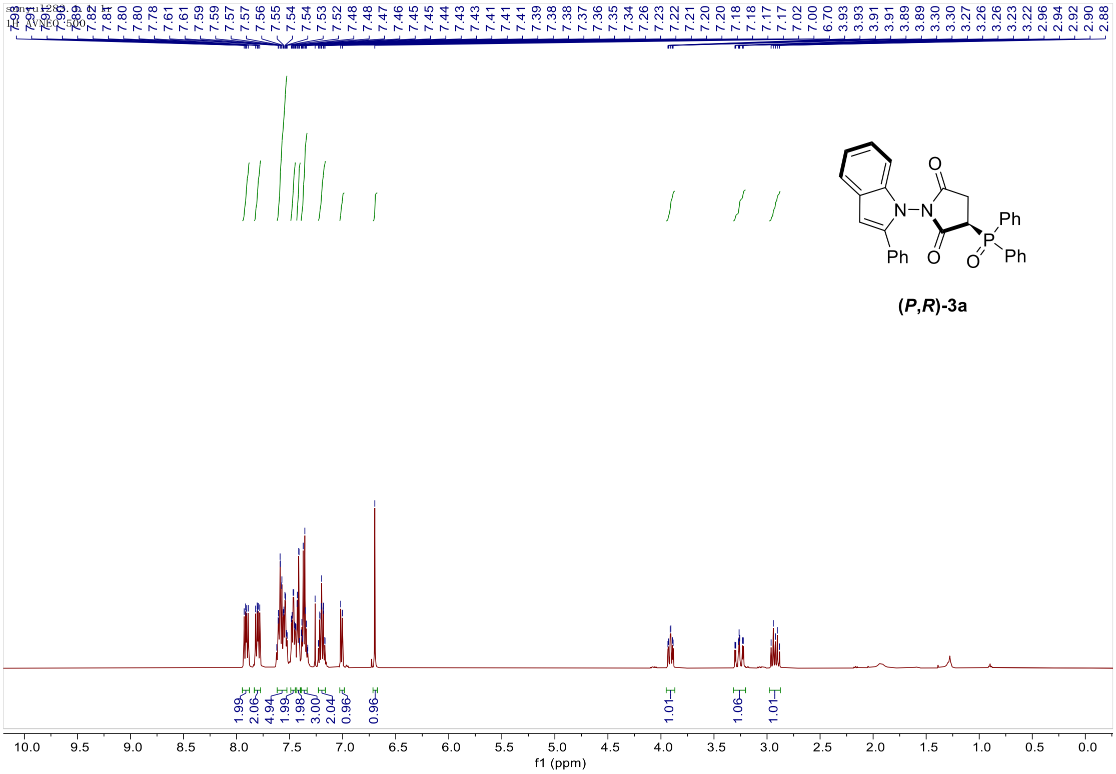
7.81 7.80 7.78 7.61 7.61 7.59 7.59 7.57 7.57 7.56 7.55 7.54 7.54 7.53 7.52 7.48 7.48 7.47 7.46 7.45 7.45 7.44 7.43 7.43 7.41 7.41 7.39 7.38 7.38 7.37 7.36 7.35 7.34 7.26 7.23 7.22 7.21 7.20 7.18 7.18 7.17 7.17 7.02 7.00 6.70 3.93 3.93 3.91 3.91 3.89 3.89 3.30 3.30 3.27 3.26 3.26 3.23 3.22 2.96 2.94 2.92 2.90 2.88
1.99 2.06 4.94 1.99 1.98 3.00 2.04 0.96 0.96 1.01 1.06 1.01
(P,R)-3a
f1 (ppm)

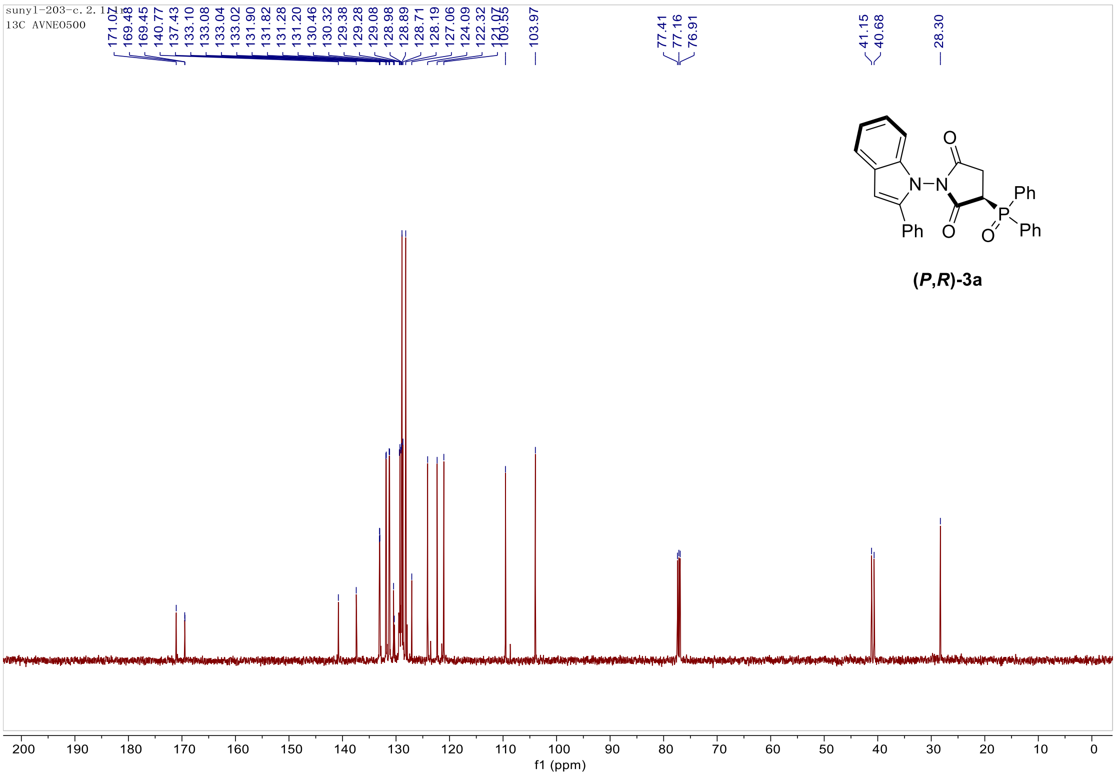
sunyl-203-c.2.1
13C AVNEO500
171.07 169.48 169.45 140.77 137.43 133.10 133.08 133.04 133.02 131.90 131.82 131.28 131.20 130.46 130.32 129.38 129.28 129.08 128.98 128.89 128.71 128.19 127.06 124.09 122.32 121.07 109.55 103.97 77.41 77.16 76.91 41.15 40.68 28.30
(P,R)-3a
f1 (ppm)

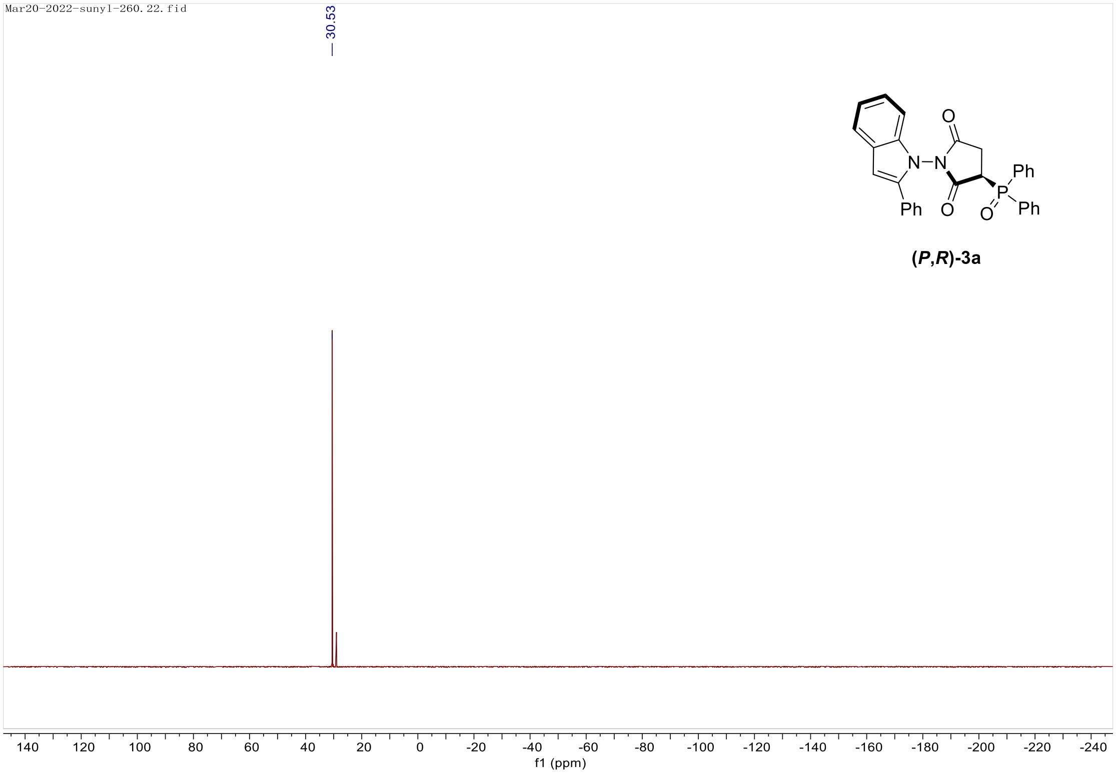
Mar20-2022-sunyl-260.22.fid
30.53
Ph
N–N
O
O
Ph
P
O
Ph
f1 (ppm)

(*P*,*R*)-3a

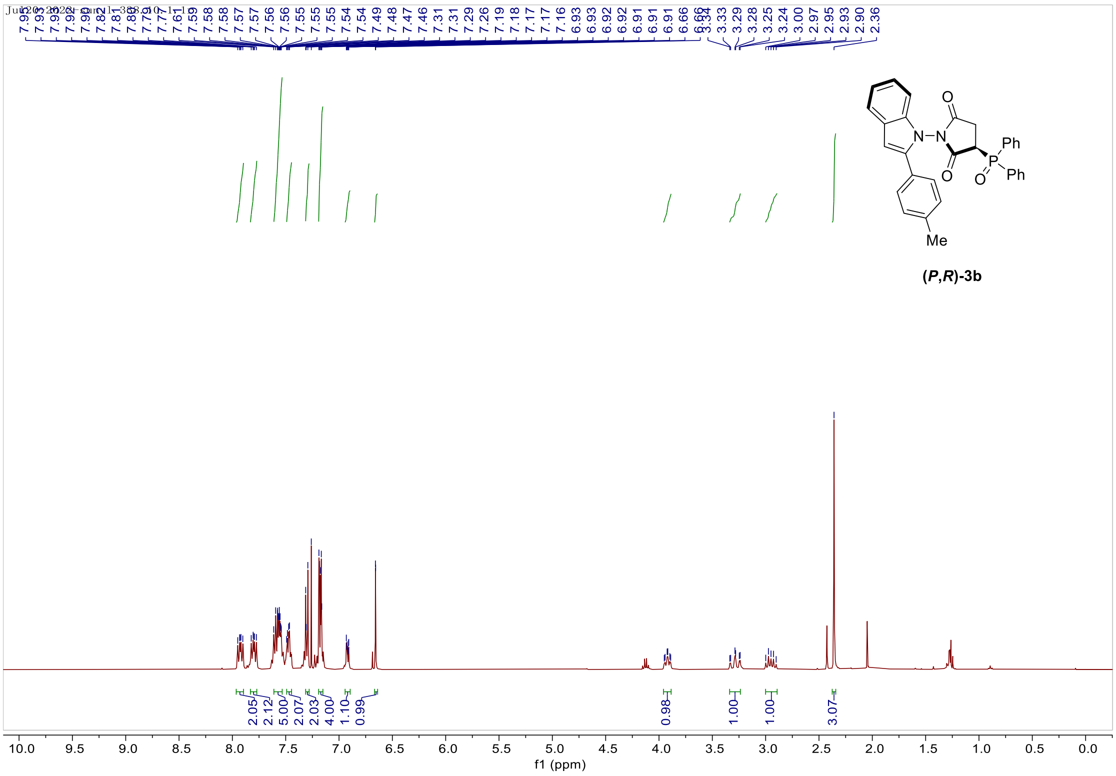
Me
Ph
Ph
2.05
2.12
5.00
2.07
2.03
4.00
1.10
0.99
0.98
1.00
1.00
3.07
f1 (ppm)

(*P*,*R*)-3b

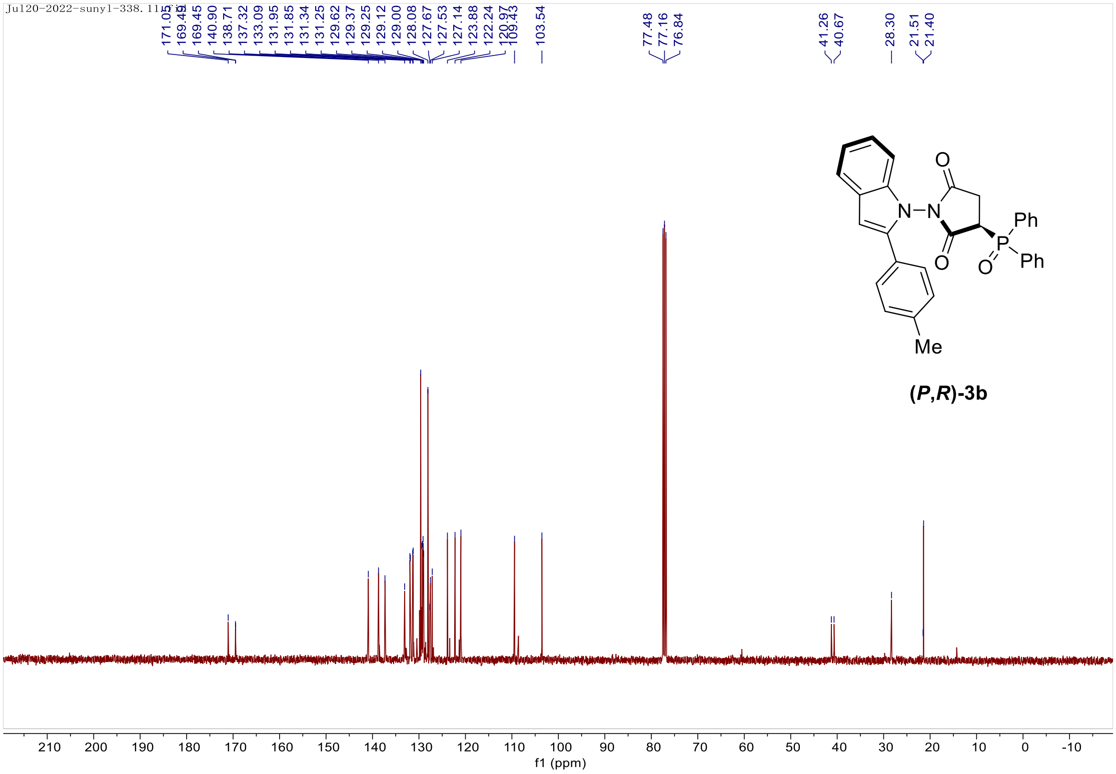
Jul20-2022-sunyl-338.11.fid
171.05
169.49
169.45
140.90
138.71
137.32
133.09
131.95
131.85
131.34
131.25
129.62
129.37
129.25
129.12
129.00
128.08
127.67
127.53
127.14
123.88
122.24
120.97
109.43
103.54
77.48
77.16
76.84
41.26
40.67
28.30
21.51
21.40
N
N
O
O
P
O
Ph
Ph
Me
(P,R)-3b
210
200
190
180
170
160
150
140
130
120
110
100
90
80
70
60
50
40
30
20
10
0
-10
f1 (ppm)

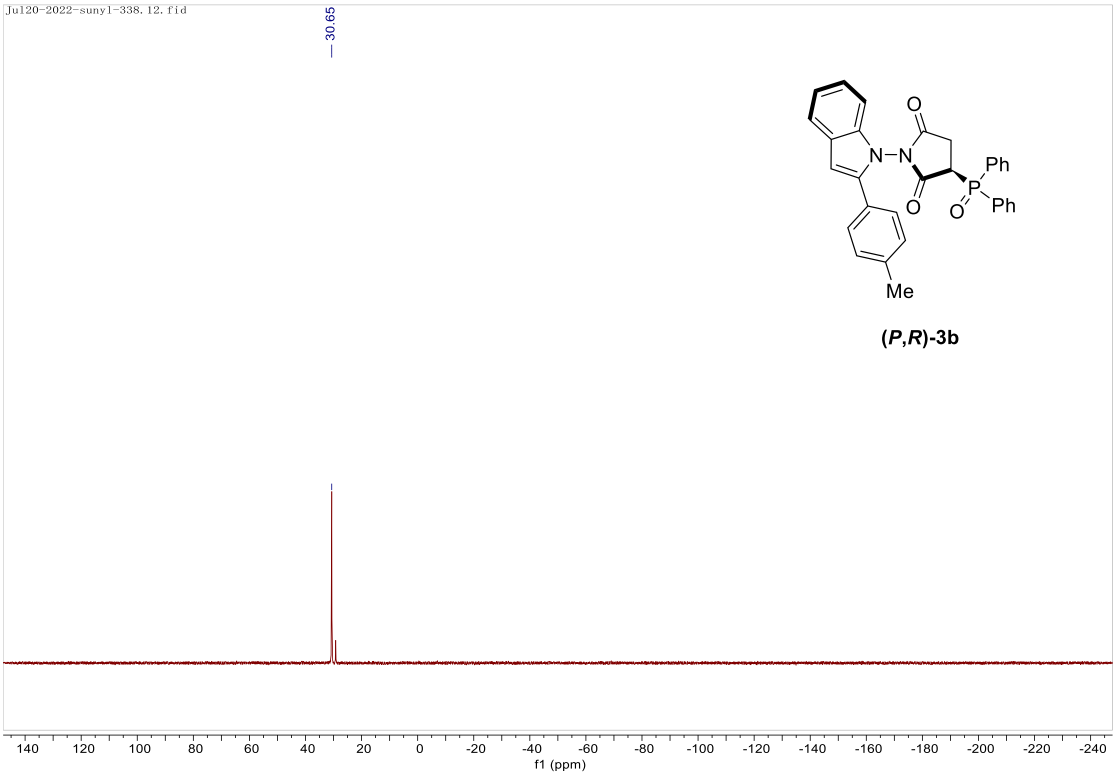
Jul20-2022-sunyl-338.12.fid
30.65
N
N
O
O
P
O
Ph
Ph
Me
(P,R)-3b
140
120
100
80
60
40
20
0
-20
-40
-60
-80
-100
-120
-140
-160
-180
-200
-220
-240
f1 (ppm)

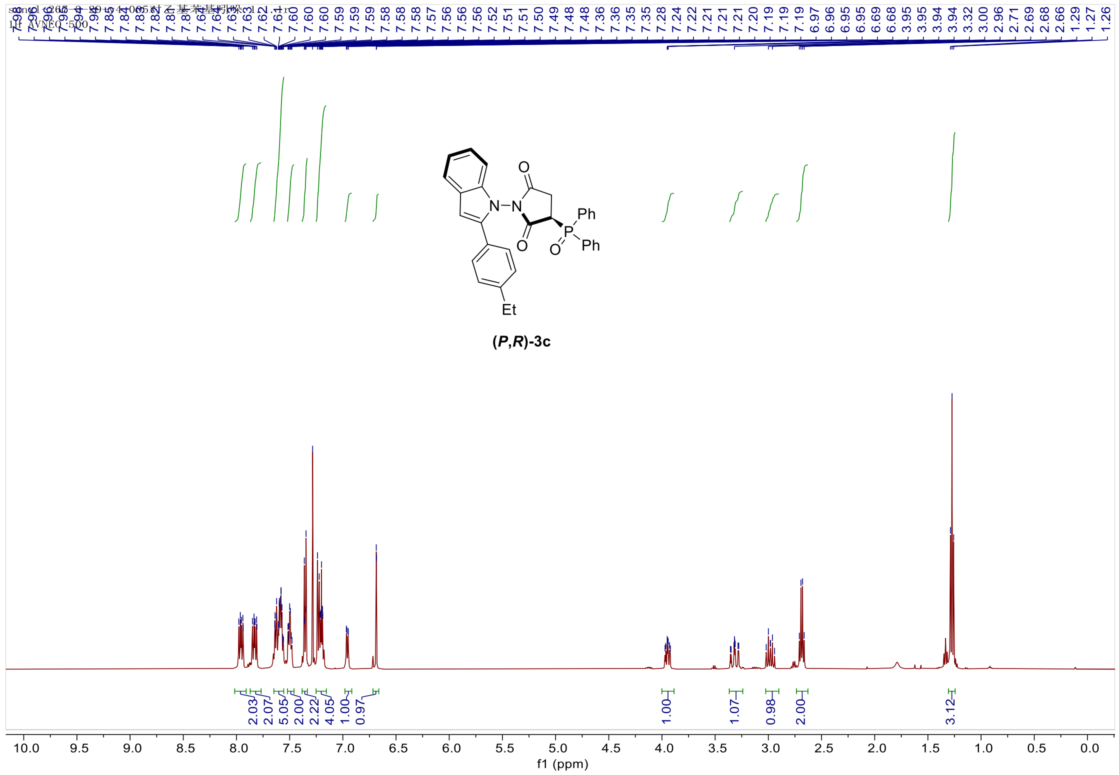

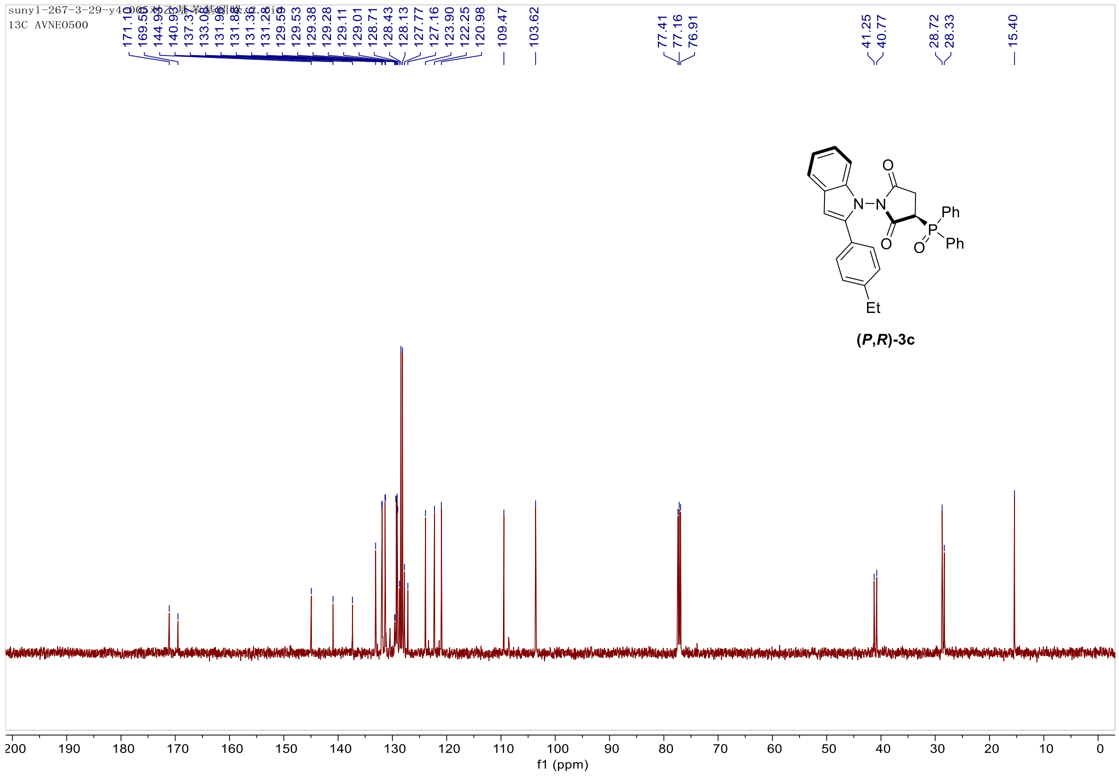

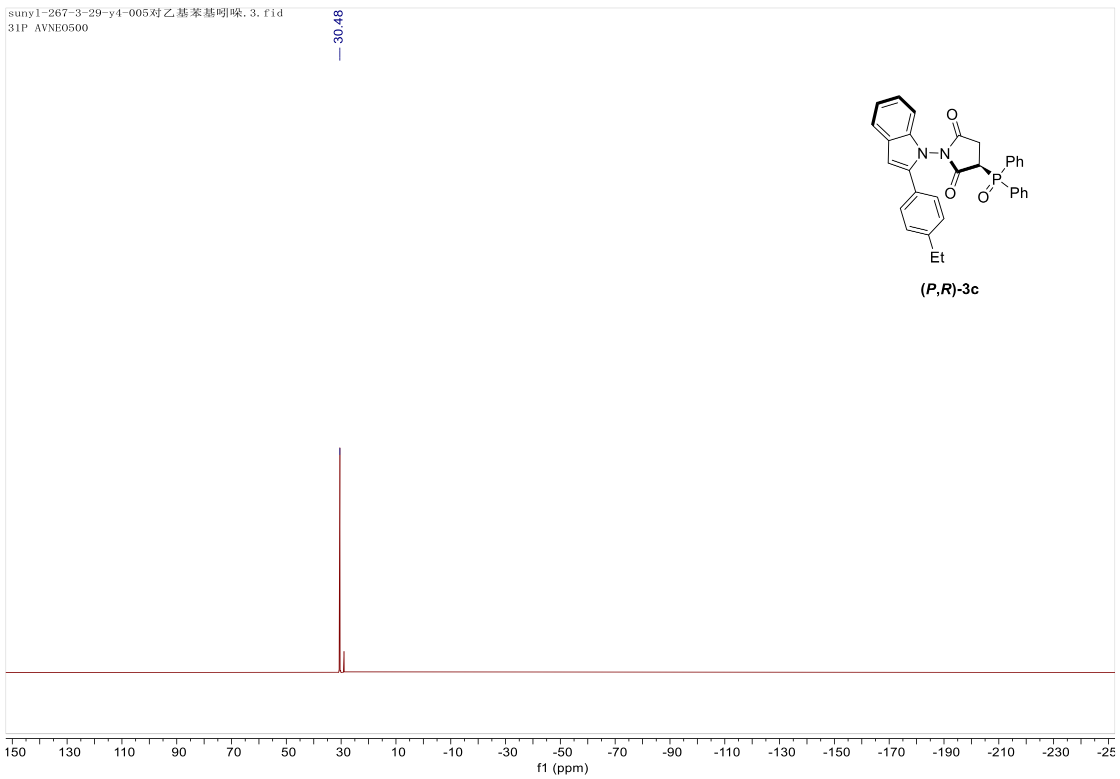
sunyl-267-3-29-y4-005对乙基苯基吲哚.3.fid
31P AVNEO500
30.48
Ph
Ph
Et
(P,R)-3c
f1 (ppm)

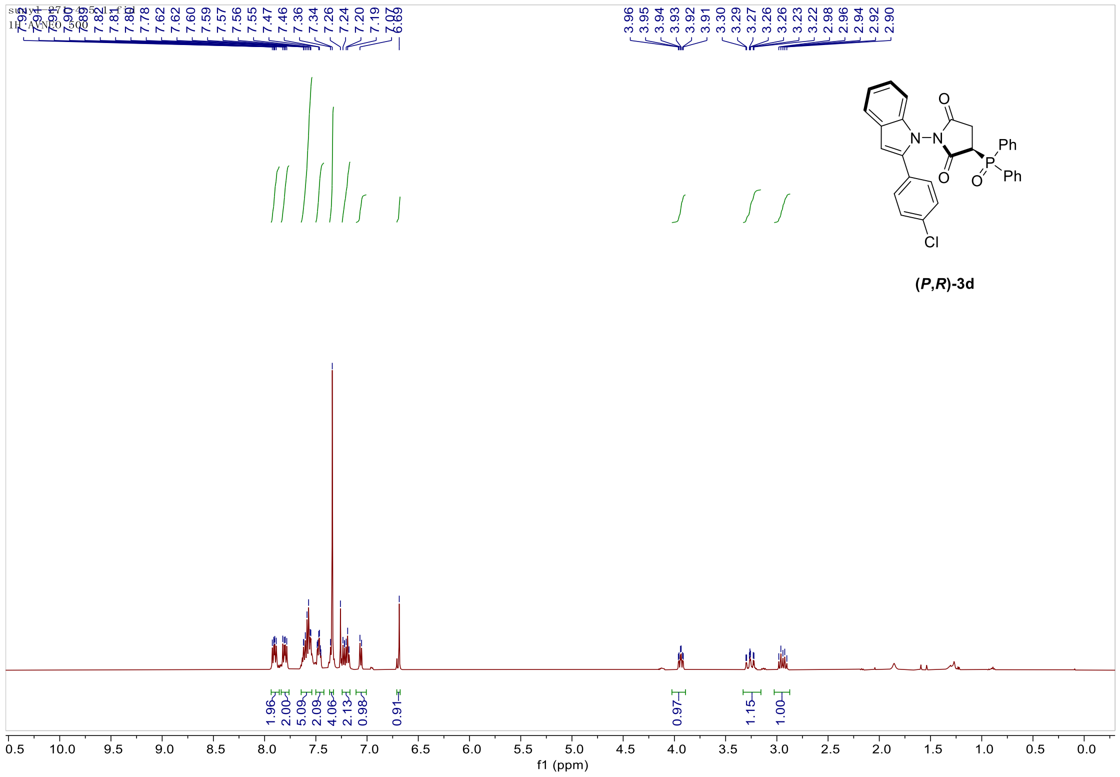
Ph
Ph
Cl
(P,R)-3d
f1 (ppm)

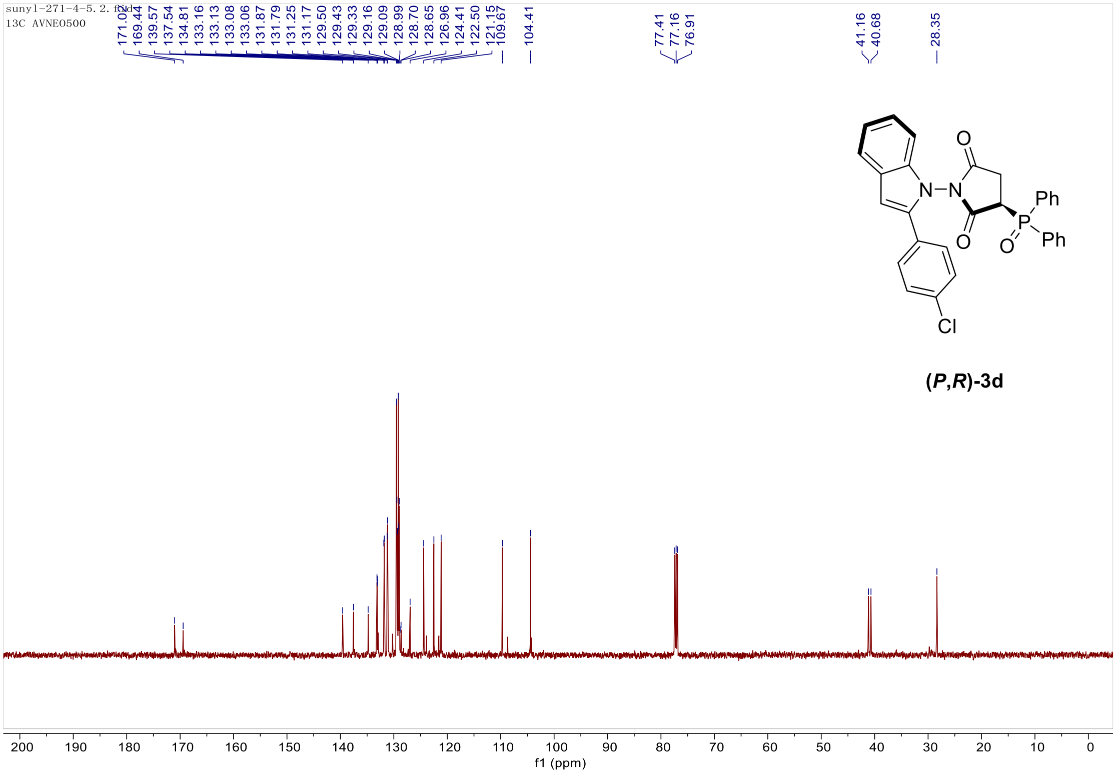
sunyl-271-4-5.2.fid
13C AVNEO500
171.02
169.44
139.57
137.54
134.81
133.16
133.13
133.08
133.06
131.87
131.79
131.25
131.17
129.50
129.43
129.33
129.16
129.09
128.99
128.70
128.65
126.96
124.41
122.50
121.15
109.67
104.41
77.41
77.16
76.91
41.16
40.68
28.35
(P,R)-3d
f1 (ppm)

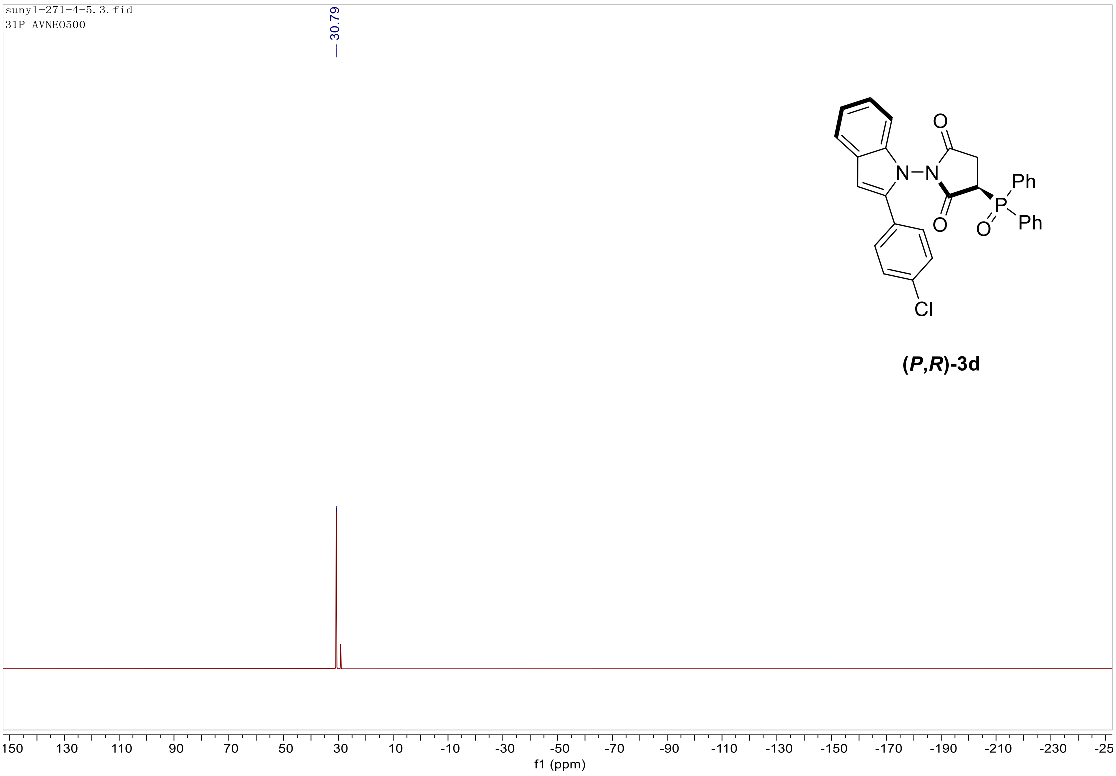
sunyl-271-4-5.3.fid
31P AVNEO500
30.79
(P,R)-3d
f1 (ppm)

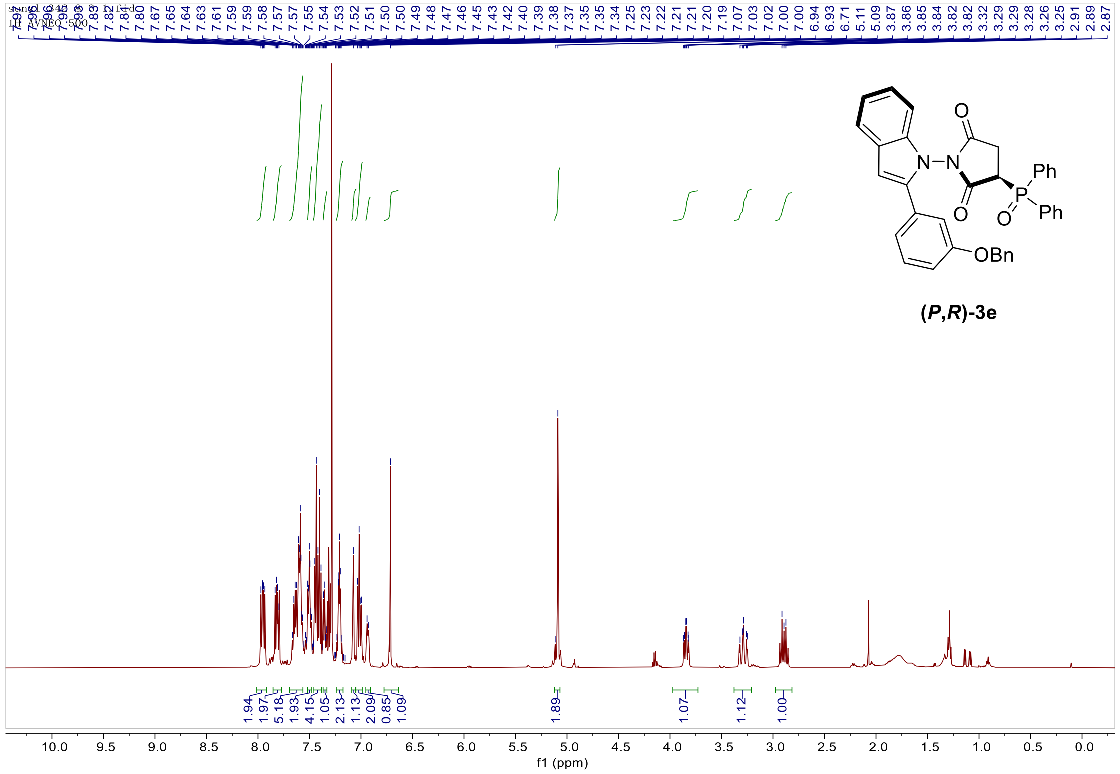
7.82 7.80 7.67 7.65 7.64 7.63 7.61 7.59 7.59 7.58 7.57 7.57 7.55 7.54 7.53 7.52 7.51 7.50 7.50 7.49 7.48 7.47 7.46 7.45 7.43 7.42 7.40 7.39 7.38 7.37 7.35 7.35 7.34 7.25 7.23 7.22 7.21 7.21 7.20 7.19 7.07 7.03 7.02 7.00 7.00 6.94 6.93 6.71 5.11 5.09 3.87 3.86 3.85 3.84 3.82 3.82 3.32 3.29 3.29 3.28 3.26 3.25 2.91 2.89 2.87
1.94 1.97 5.18 1.93 4.15 1.05 2.13 1.13 2.09 0.85 1.09 1.89 1.07 1.12 1.00
O
N
N
Ph
P
O
Ph
O
OBn
(P,R)-3e
f1 (ppm)
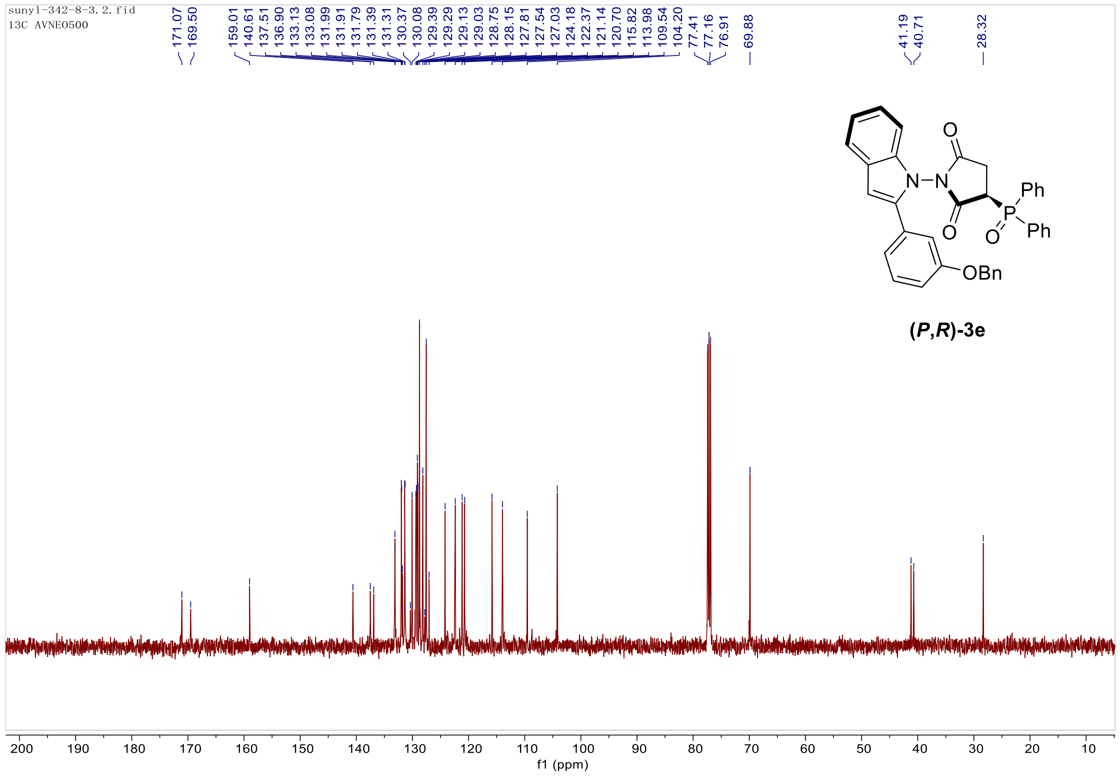
sunyl-342-8-3.2.fid
13C AVNEO500
171.07 169.50 159.01 140.61 137.51 136.90 133.13 133.08 131.99 131.91 131.79 131.39 131.31 130.37 130.08 129.39 129.29 129.13 129.03 128.75 128.15 127.81 127.54 127.03 124.18 122.37 121.14 120.70 115.82 113.98 109.54 104.20 77.41 77.16 76.91 69.88 41.19 40.71 28.32
O
N
N
Ph
P
O
Ph
O
OBn
(P,R)-3e
f1 (ppm)

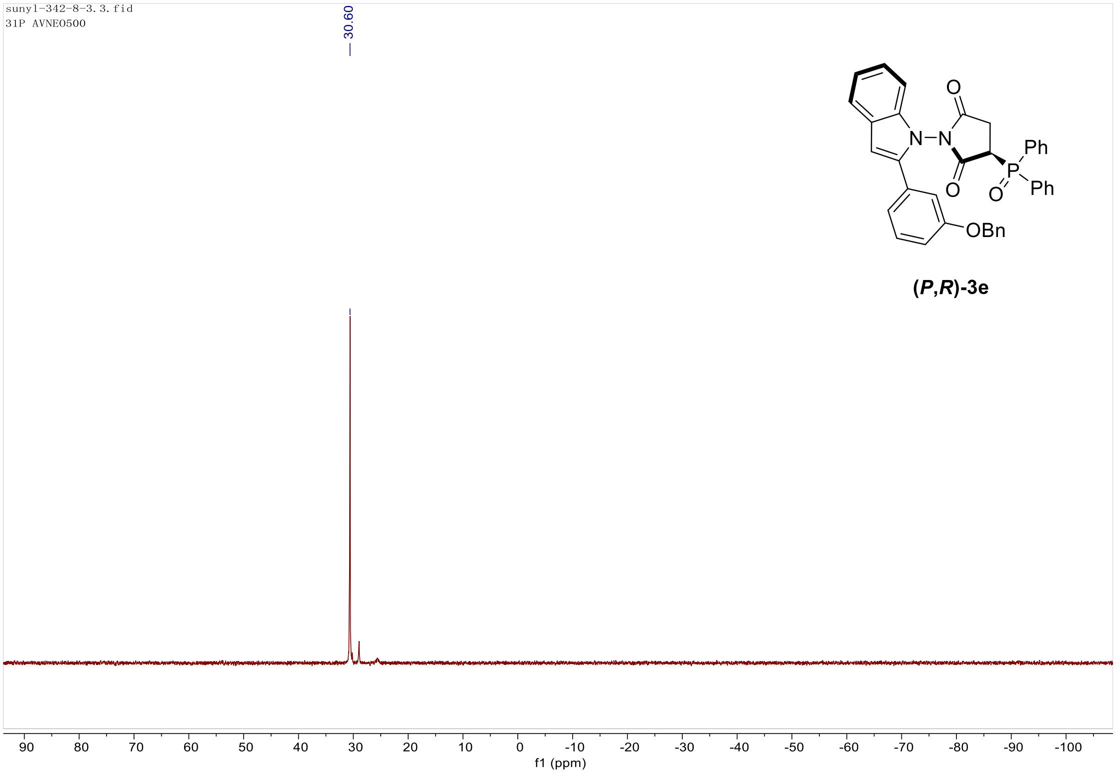
sunyl-342-8-3.3.fid
31P AVNEO500
30.60
(P,R)-3e
f1 (ppm)

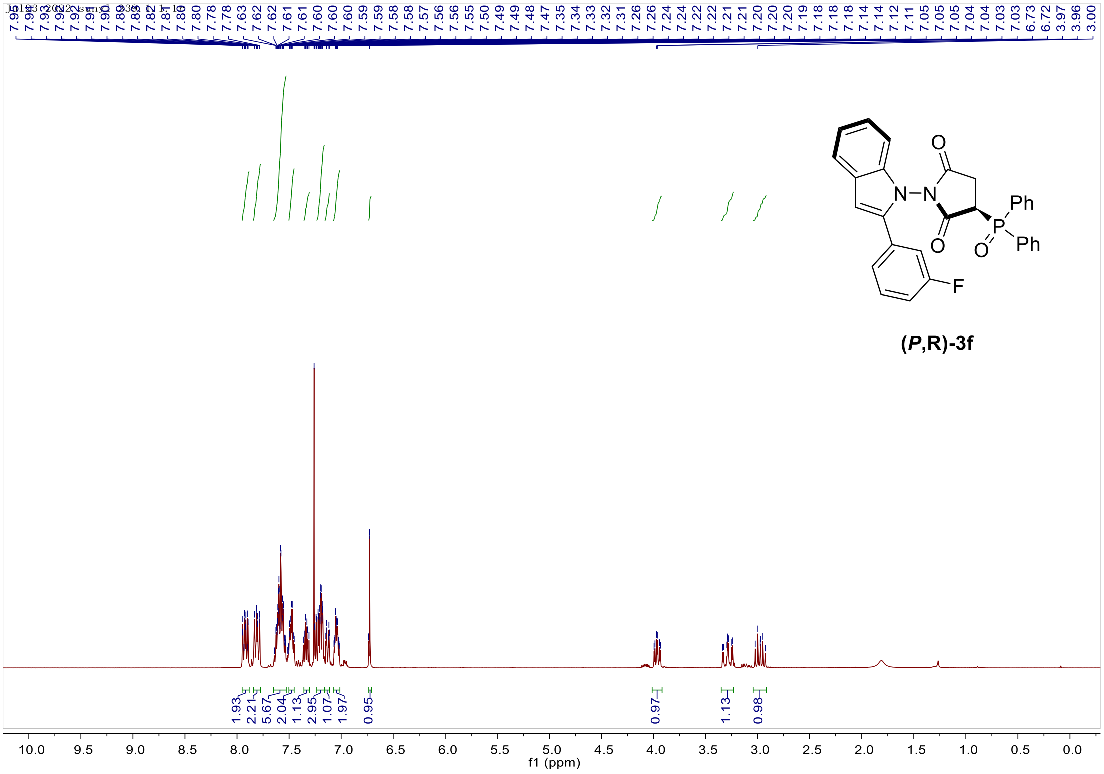
(P,R)-3f
f1 (ppm)

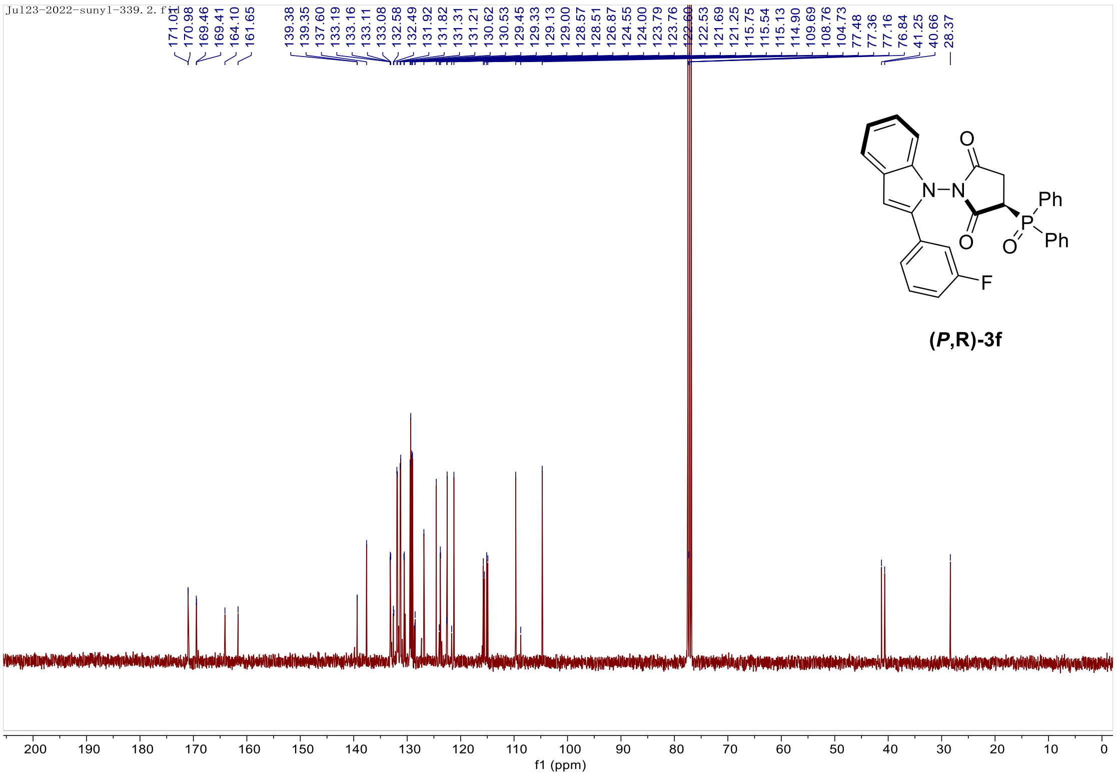
Jul23-2022-sunyl-339.2.fid
171.01
170.98
169.46
169.41
164.10
161.65
139.38
139.35
137.60
133.19
133.16
133.11
133.08
132.58
132.49
131.92
131.82
131.31
131.21
130.62
130.53
129.45
129.33
129.13
129.00
128.57
128.51
126.87
124.55
124.00
123.79
123.76
122.60
122.53
121.69
121.25
115.75
115.54
115.13
114.90
109.69
108.76
104.73
77.48
77.36
77.16
76.84
41.25
40.66
28.37
(P,R)-3f
f1 (ppm)

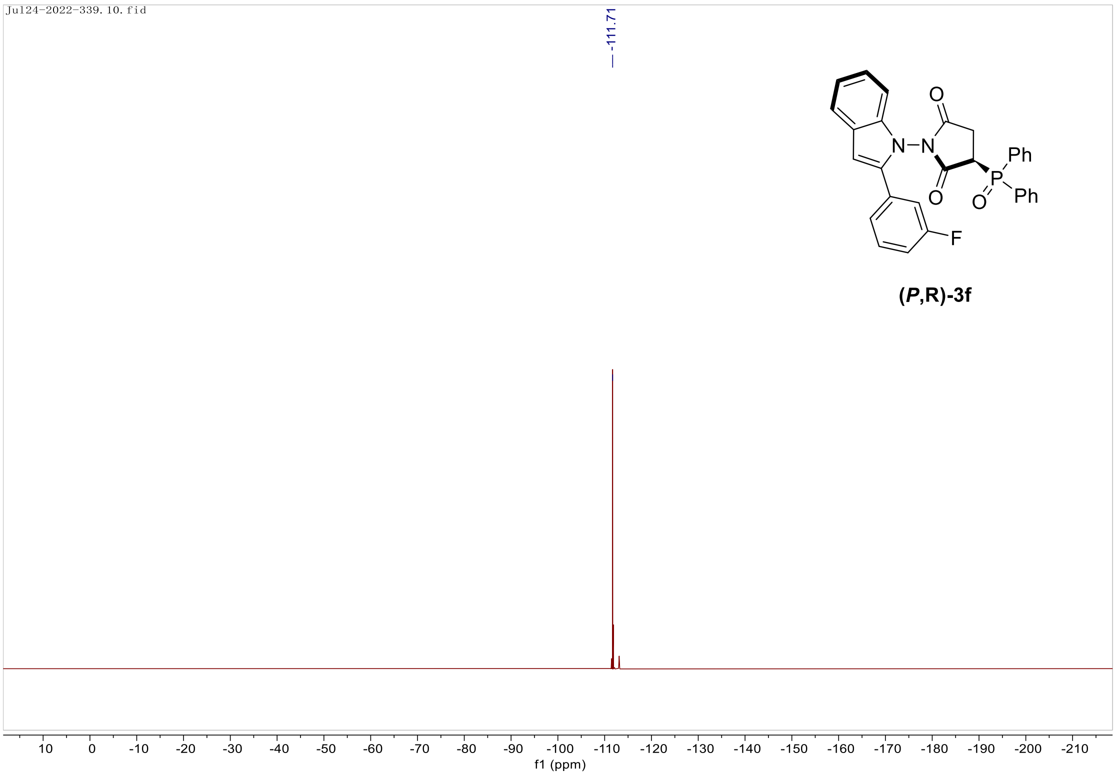
Jul24-2022-339.10.fid
-111.71
(P,R)-3f
f1 (ppm)

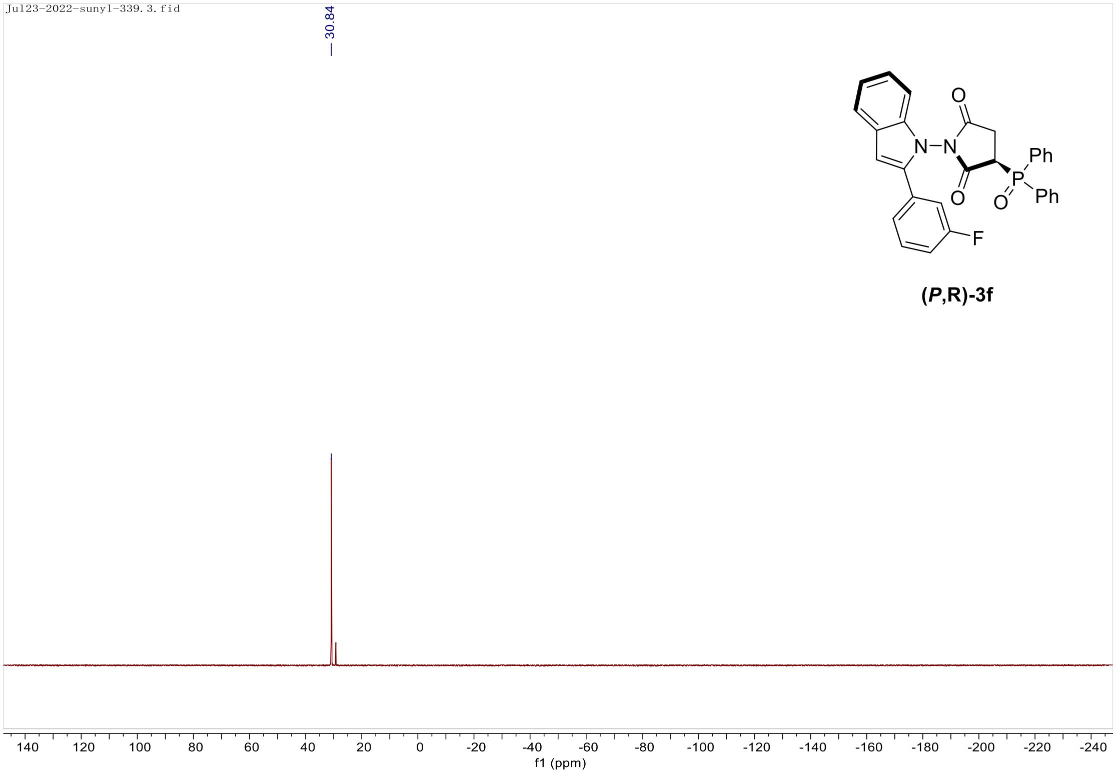
Jul23-2022-sunyl-339.3.fid
30.84
O
N
N
Ph
P
Ph
O
O
F
(P,R)-3f
f1 (ppm)

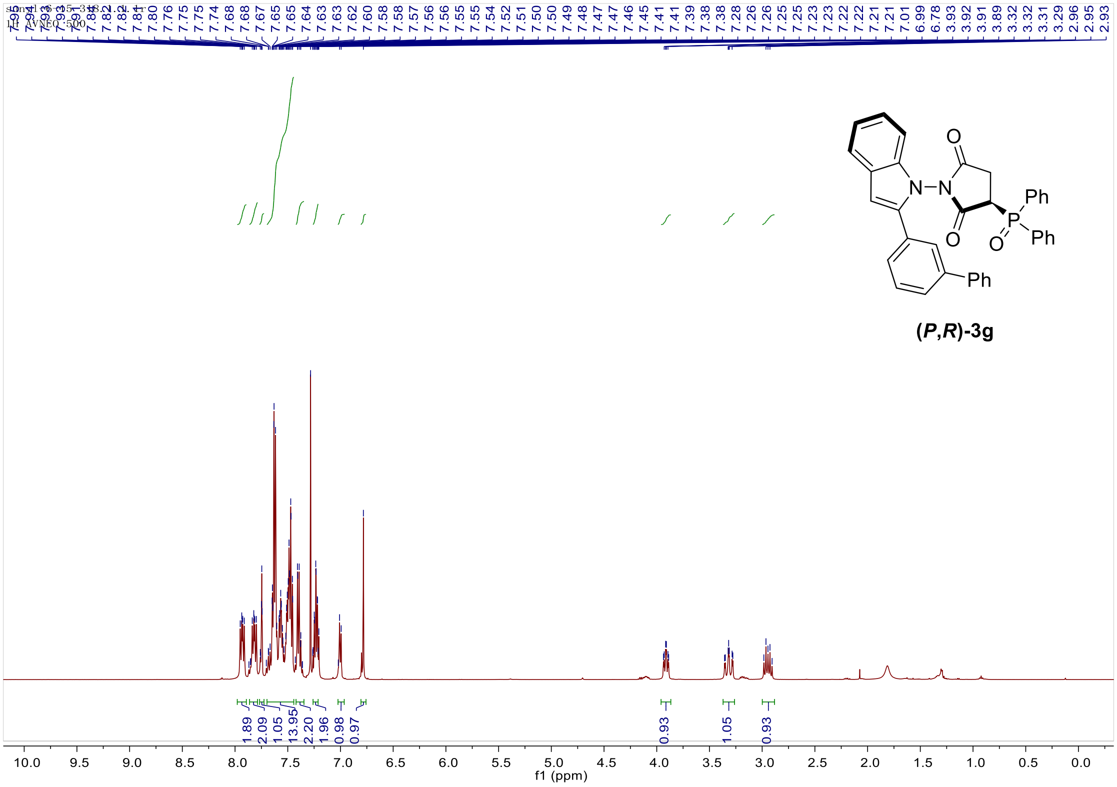
O
N
N
Ph
P
Ph
O
O
Ph
(P,R)-3g
1.89
2.09
1.05
13.95
2.20
1.96
0.98
0.97
0.93
1.05
0.93
f1 (ppm)

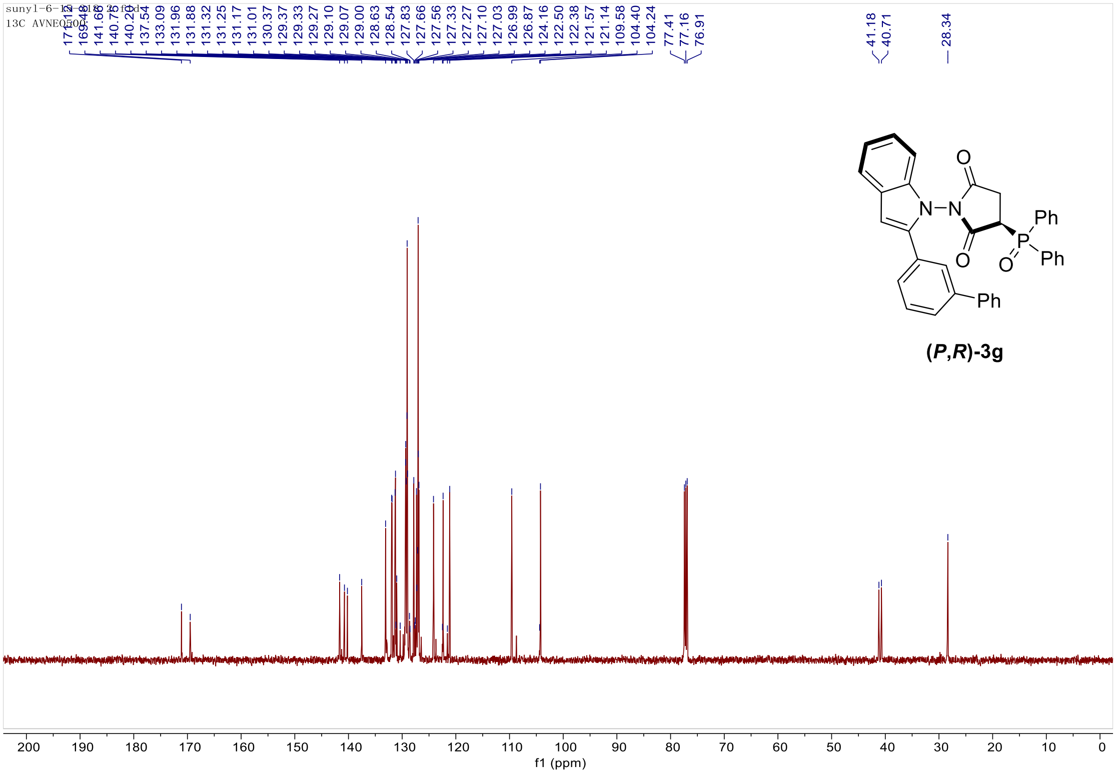
13C AVNEO500
141.66
140.76
140.20
137.54
133.09
131.96
131.88
131.32
131.25
131.17
131.01
130.37
129.37
129.33
129.27
129.10
129.07
129.00
128.63
128.54
127.83
127.66
127.56
127.33
127.27
127.10
127.03
126.99
126.87
124.16
122.50
122.38
121.57
121.14
109.58
104.40
104.24
77.41
77.16
76.91
41.18
40.71
28.34
N
N
O
O
Ph
P
O
Ph
Ph
(P,R)-3g
200 190 180 170 160 150 140 130 120 110 100 90 80 70 60 50 40 30 20 10 0
f1 (ppm)

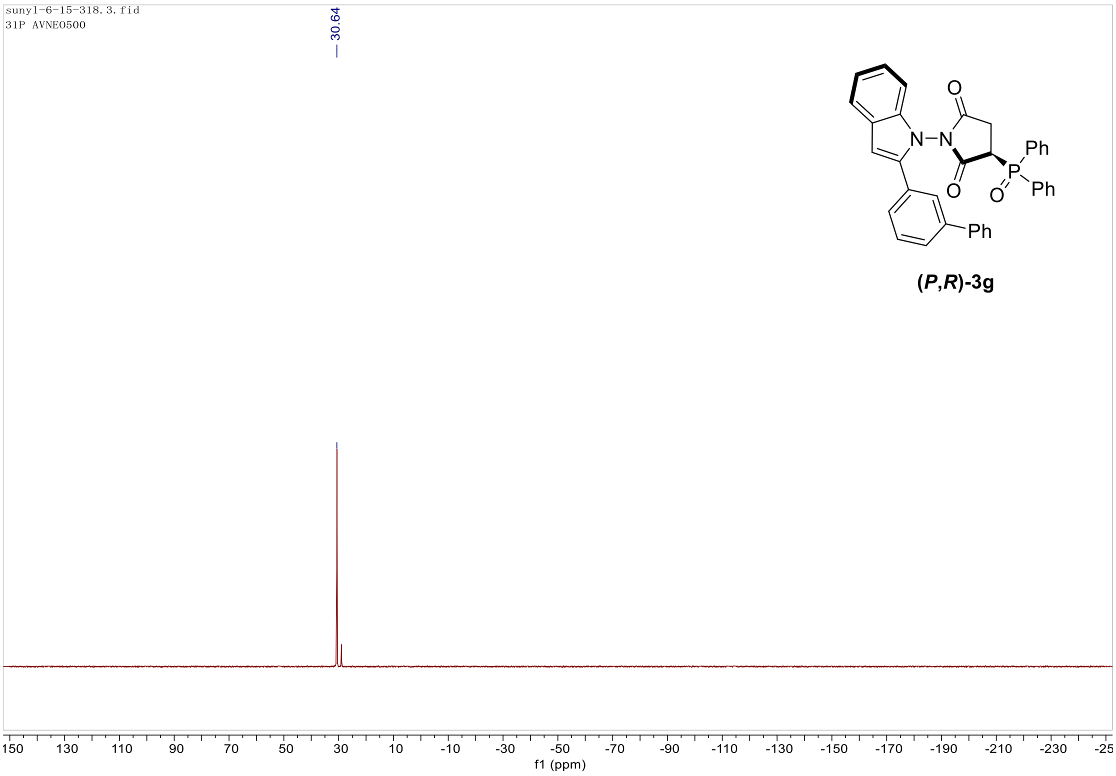
sunyl-6-15-318.3.fid
31P AVNEO500
30.64
N
N
O
O
Ph
P
O
Ph
Ph
(P,R)-3g
150 130 110 90 70 50 30 10 -10 -30 -50 -70 -90 -110 -130 -150 -170 -190 -210 -230 -25
f1 (ppm)

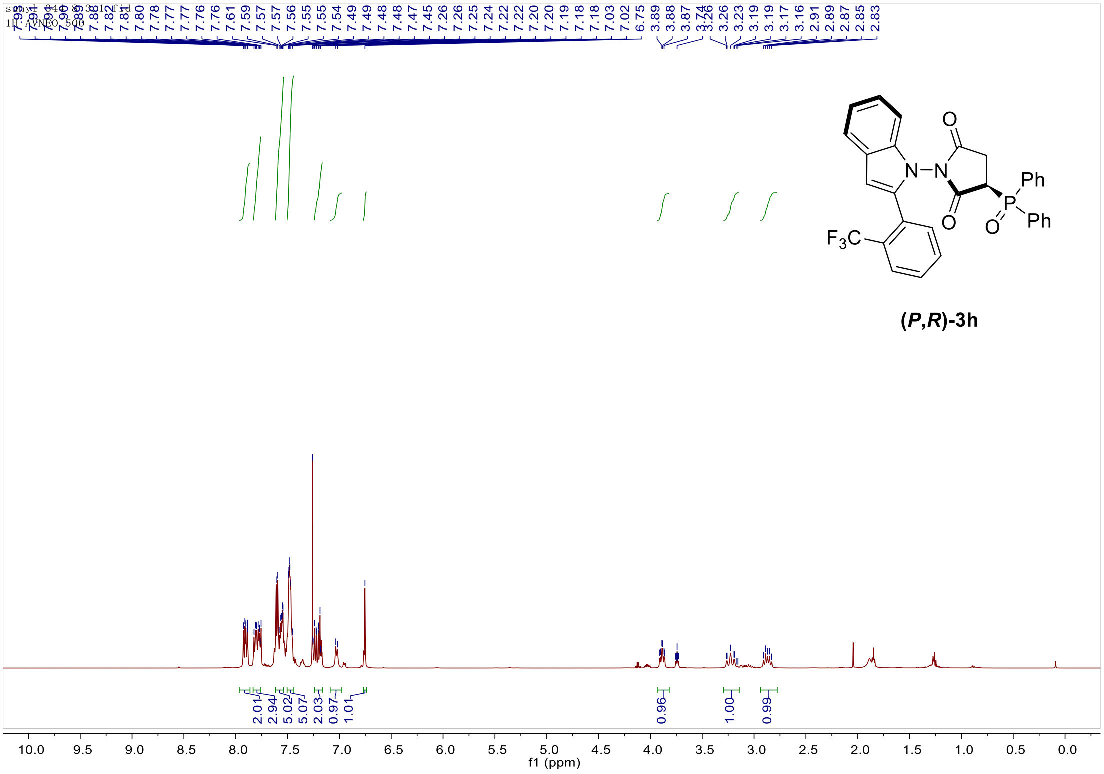
(P,R)-3h
f1 (ppm)

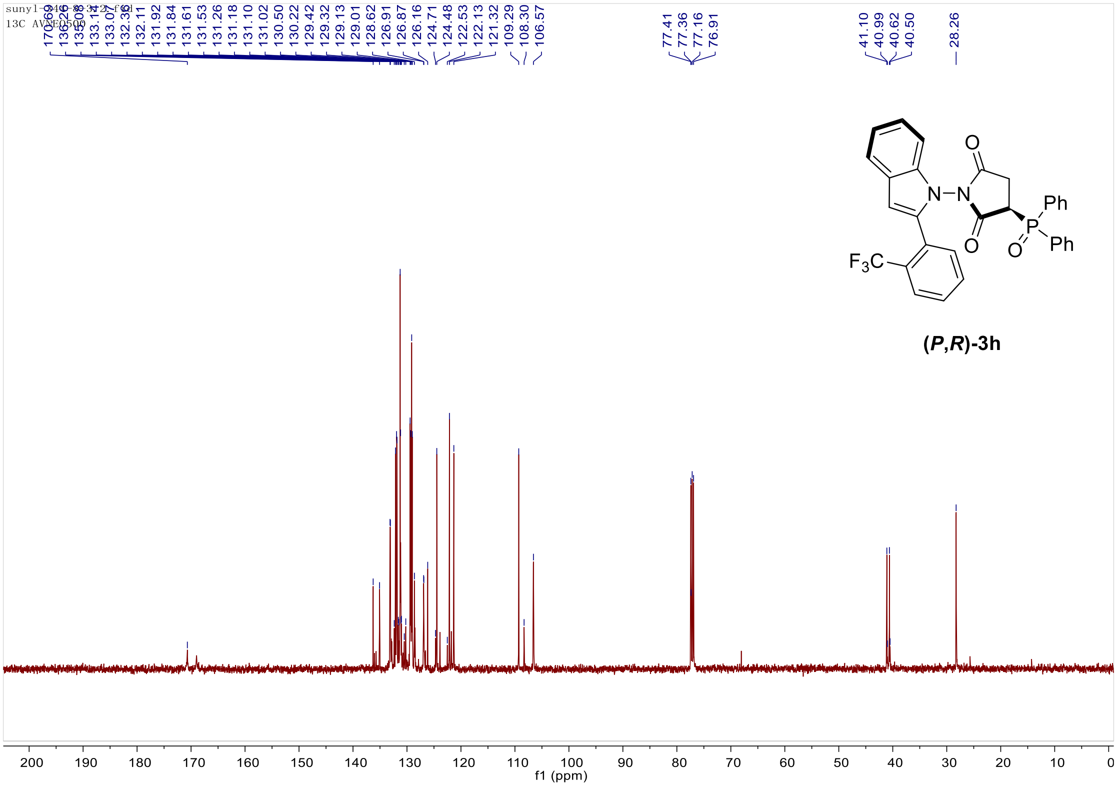
(P,R)-3h
f1 (ppm)

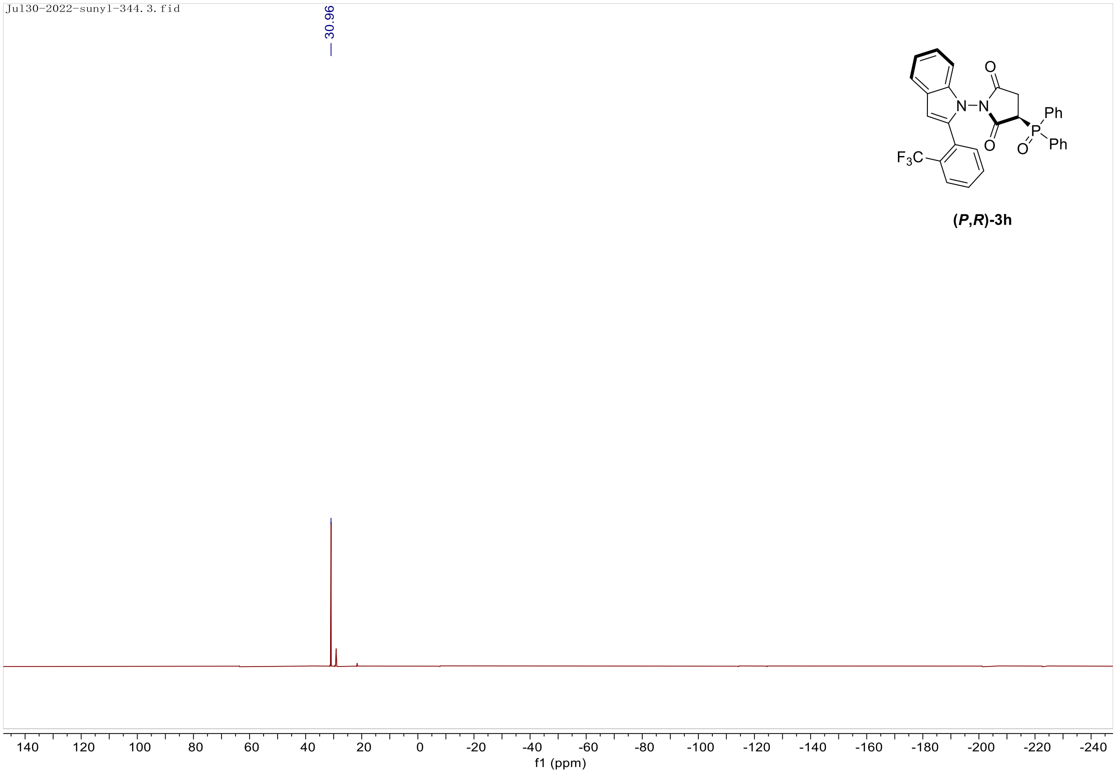
Jul30-2022-sunyl-344.3.fid
30.96
N
N
O
O
P
Ph
Ph
O
F3C
(P,R)-3h
140 120 100 80 60 40 20 0 -20 -40 -60 -80 -100 -120 -140 -160 -180 -200 -220 -240
f1 (ppm)

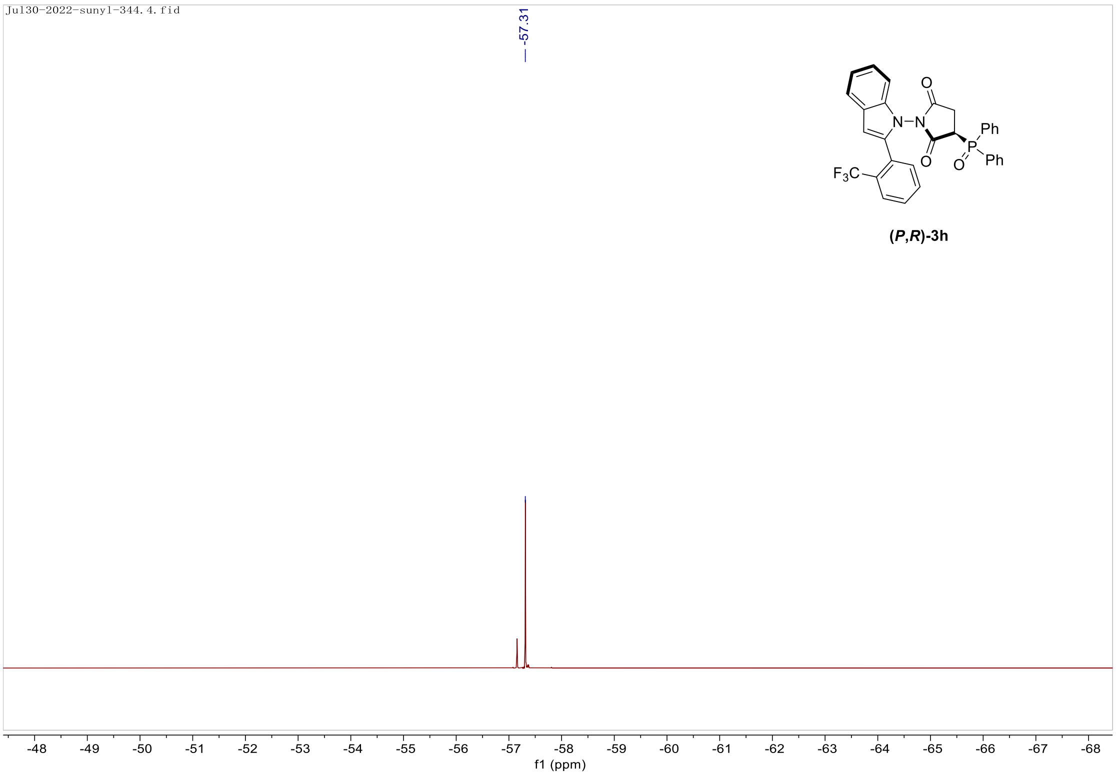
Jul30-2022-sunyl-344.4.fid
-57.31
N
N
O
O
P
Ph
Ph
O
F3C
(P,R)-3h
-48 -49 -50 -51 -52 -53 -54 -55 -56 -57 -58 -59 -60 -61 -62 -63 -64 -65 -66 -67 -68
f1 (ppm)

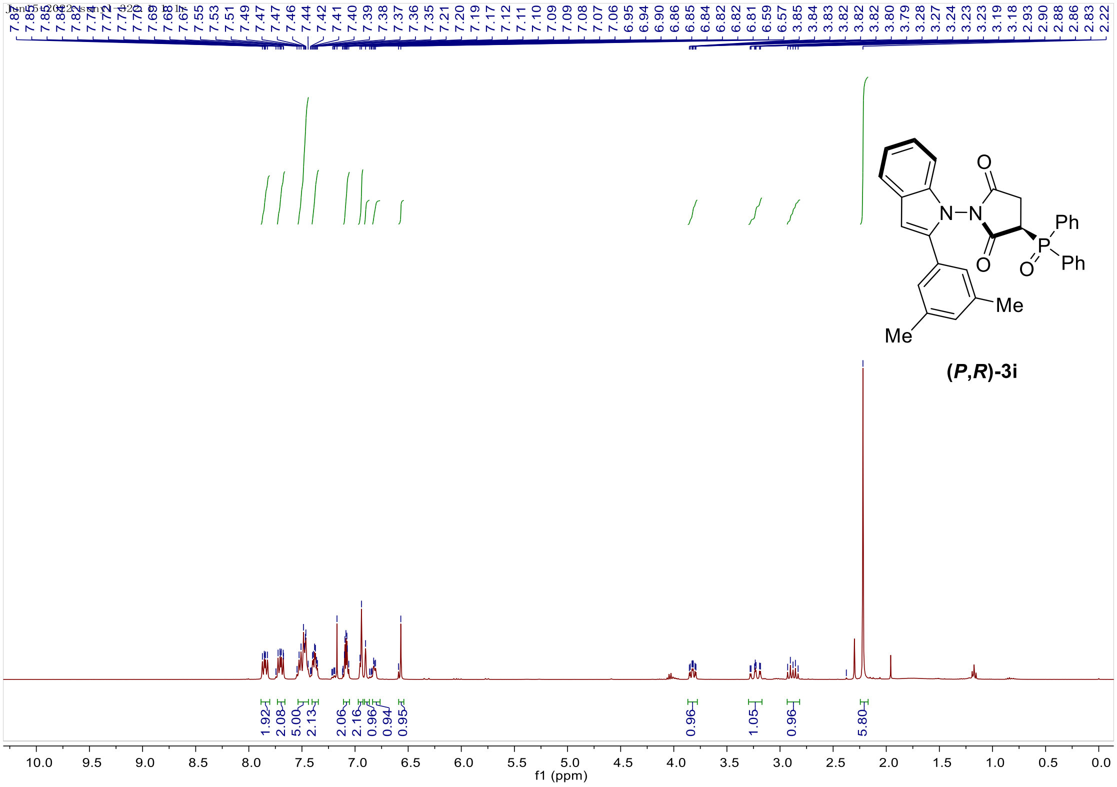

7.85 7.85 7.84 7.82 7.74 7.72 7.70 7.69 7.68 7.67 7.55 7.53 7.51 7.49 7.47 7.47 7.46 7.44 7.42 7.41 7.40 7.39 7.38 7.37 7.36 7.35 7.21 7.20 7.19 7.17 7.12 7.11 7.10 7.09 7.09 7.08 7.07 7.06 6.95 6.94 6.90 6.86 6.85 6.84 6.82 6.82 6.81 6.59 6.57 3.85 3.84 3.83 3.82 3.82 3.82 3.80 3.79 3.28 3.27 3.24 3.23 3.23 3.19 3.18 2.93 2.90 2.88 2.86 2.83 2.22
O
N
N
O
Ph
P
O
Ph
Me
Me
(P,R)-3i
1.92 2.08 5.00 2.13 2.06 2.16 0.96 0.94 0.95 0.96 1.05 0.96 5.80
10.0 9.5 9.0 8.5 8.0 7.5 7.0 6.5 6.0 5.5 5.0 4.5 4.0 3.5 3.0 2.5 2.0 1.5 1.0 0.5 0.0
f1 (ppm)

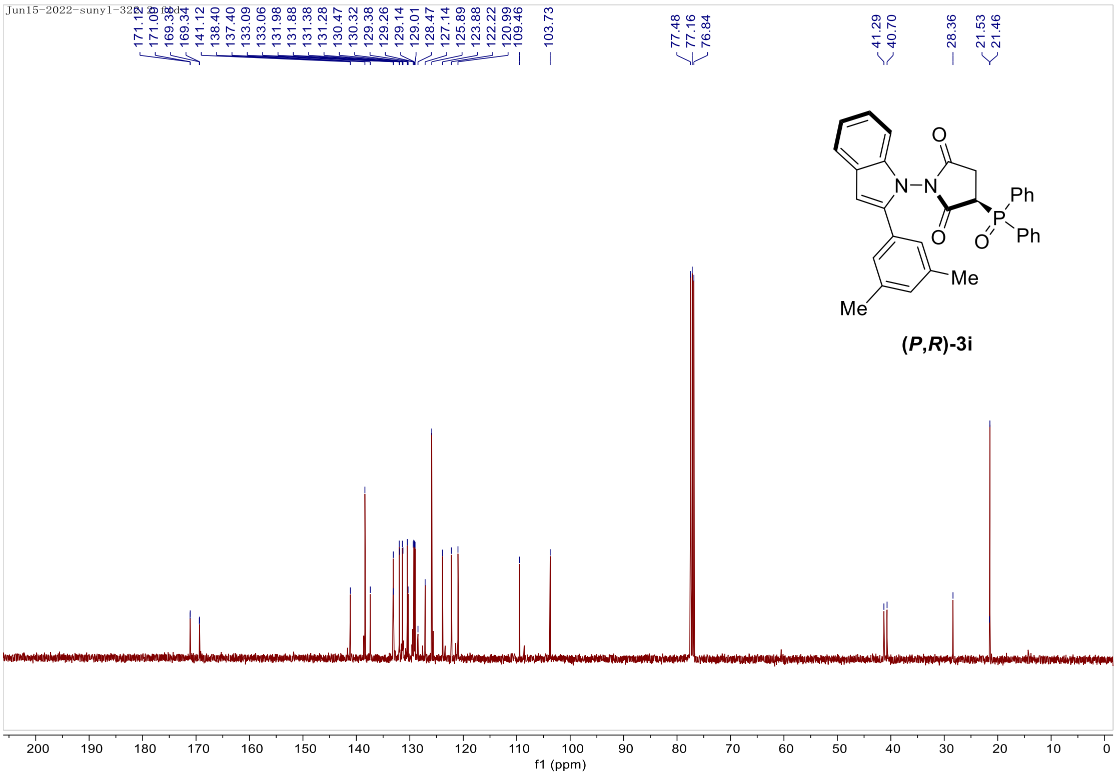

Jun15-2022-suny1-322
171.12 171.09 169.38 169.34 141.12 138.40 137.40 133.09 133.06 131.98 131.88 131.38 131.28 130.47 130.32 129.38 129.26 129.14 129.01 128.47 127.14 125.89 123.88 122.22 120.99 109.46 103.73 77.48 77.16 76.84 41.29 40.70 28.36 21.53 21.46
O
N
N
O
Ph
P
O
Ph
Me
Me
(P,R)-3i
200 190 180 170 160 150 140 130 120 110 100 90 80 70 60 50 40 30 20 10 0
f1 (ppm)

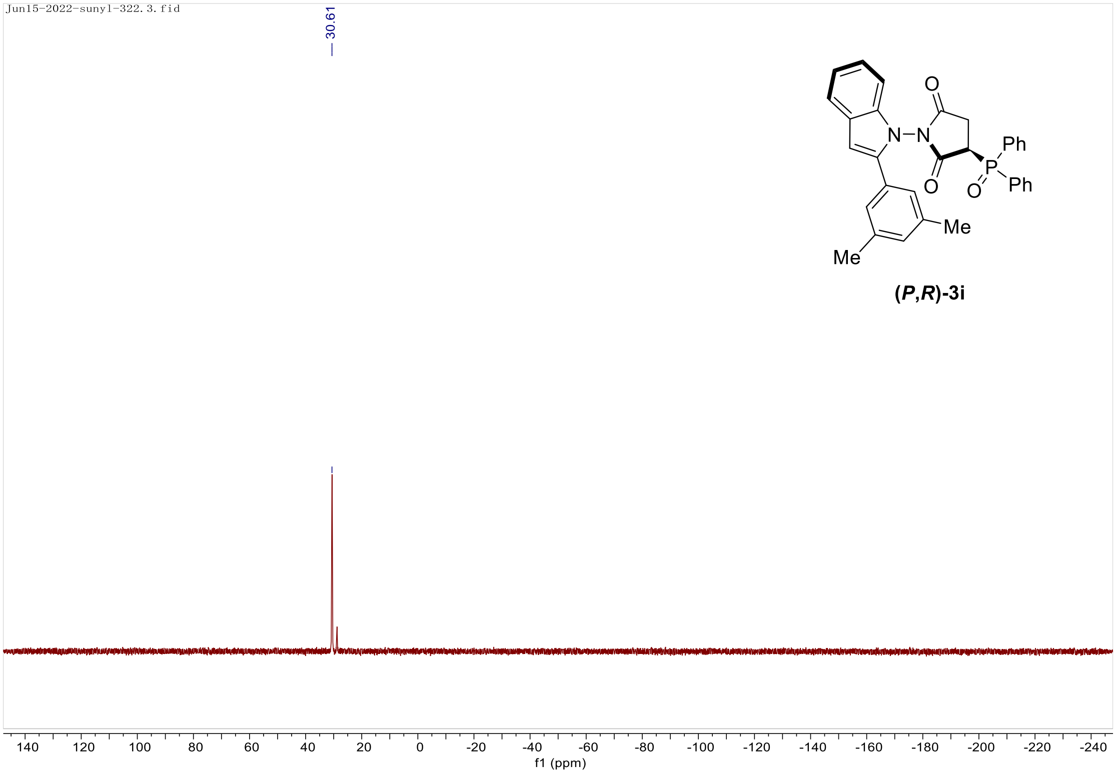
Jun15-2022-sunyl-322.3.fid
30.61
Ph
Ph
Me
Me
f1 (ppm)

(*P*,*R*)-3i

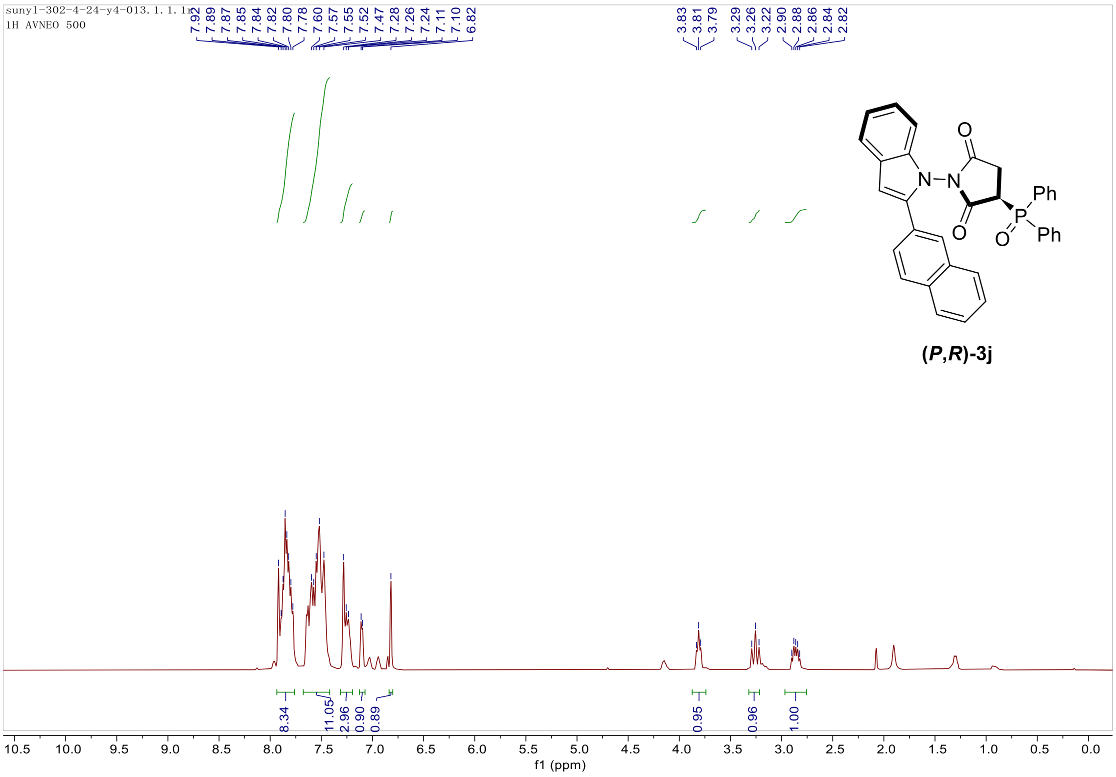
sunyl-302-4-24-y4-013.1.1.1r
1H AVNEO 500
7.92
7.89
7.87
7.85
7.84
7.82
7.80
7.78
7.60
7.57
7.55
7.52
7.47
7.28
7.26
7.24
7.11
7.10
6.82
3.83
3.81
3.79
3.29
3.26
3.22
2.90
2.88
2.86
2.84
2.82
Ph
Ph
8.34
11.05
2.96
0.90
0.89
0.95
0.96
1.00
f1 (ppm)

(*P*,*R*)-3j

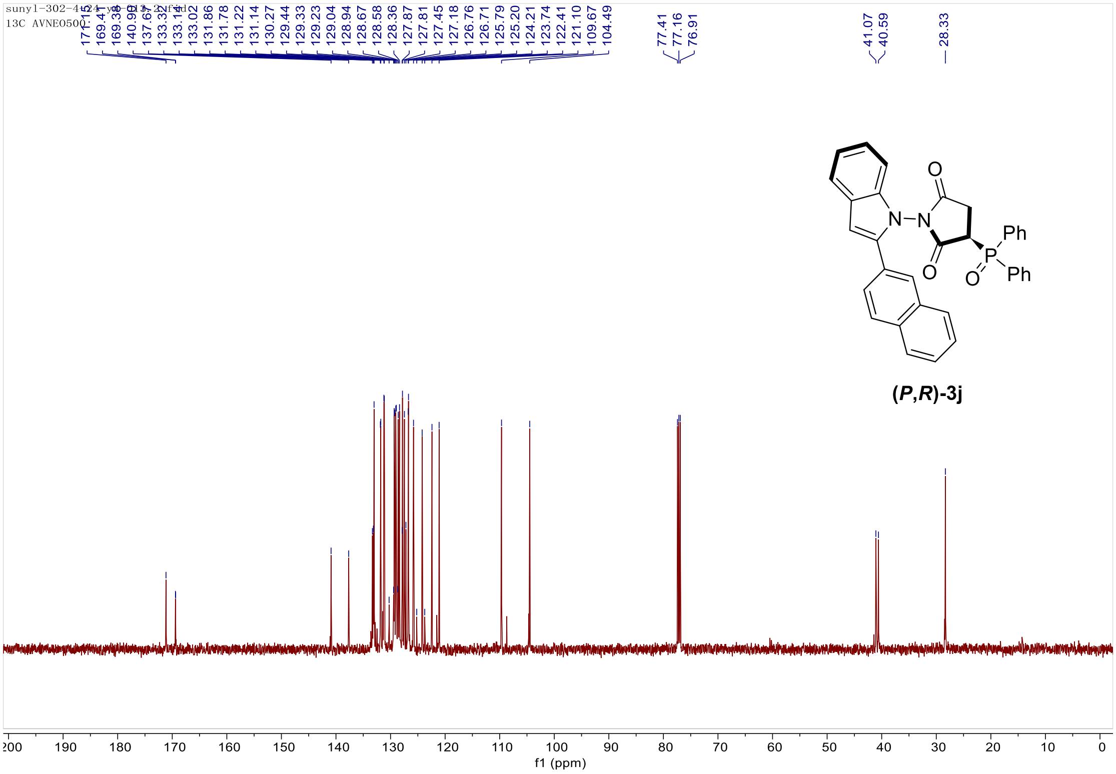
13C AVNEO500
171.15 169.41 169.38 140.99 137.67 133.32 133.14 133.02 131.86 131.78 131.22 131.14 130.27 129.44 129.33 129.23 129.04 128.94 128.67 128.58 128.36 127.87 127.81 127.45 127.18 126.76 126.71 125.79 125.20 124.21 123.74 122.41 121.10 109.67 104.49
77.41 77.16 76.91
41.07 40.59
28.33
O
N
N
Ph
P
O
O
Ph
(P,R)-3j
f1 (ppm)

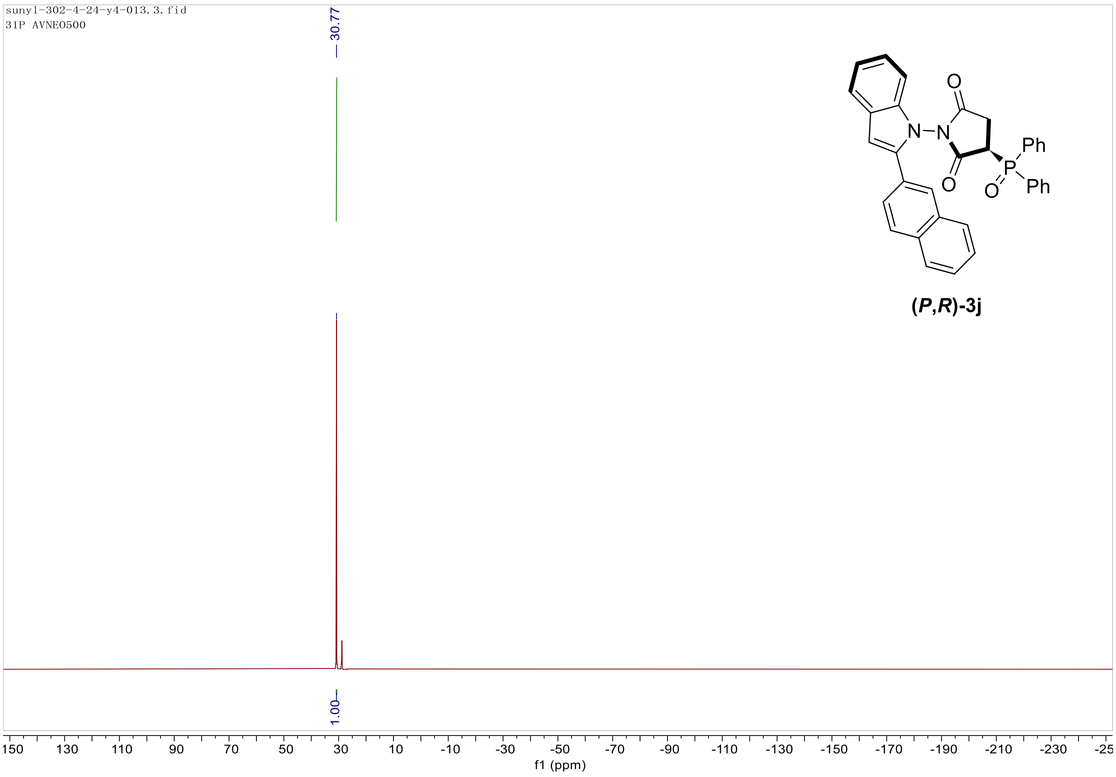
sunyl-302-4-24-y4-013.3.fid
31P AVNEO500
30.77
O
N
N
Ph
P
O
O
Ph
(P,R)-3j
1.00
f1 (ppm)

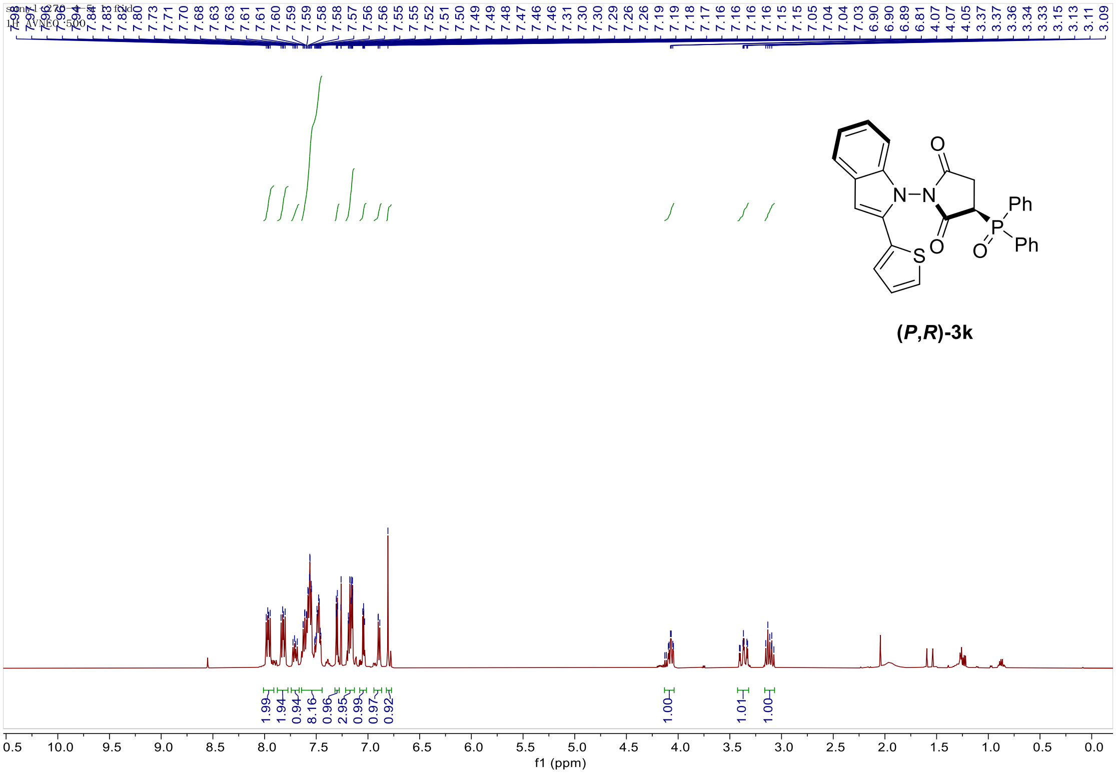

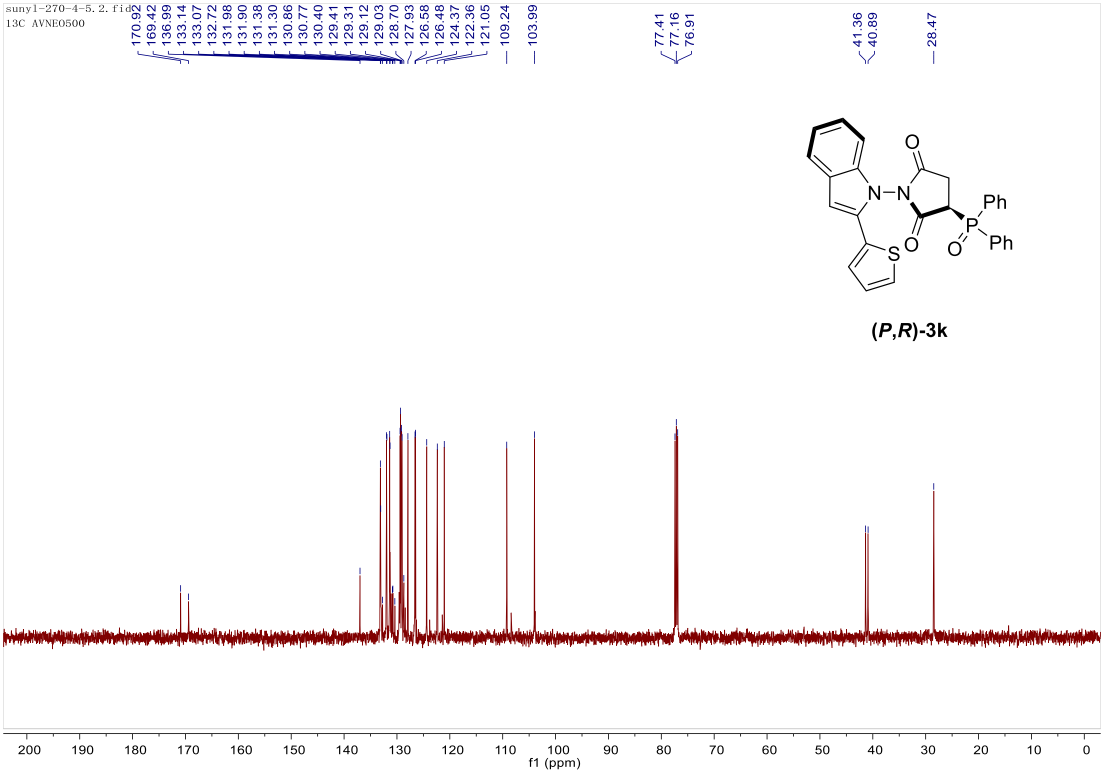

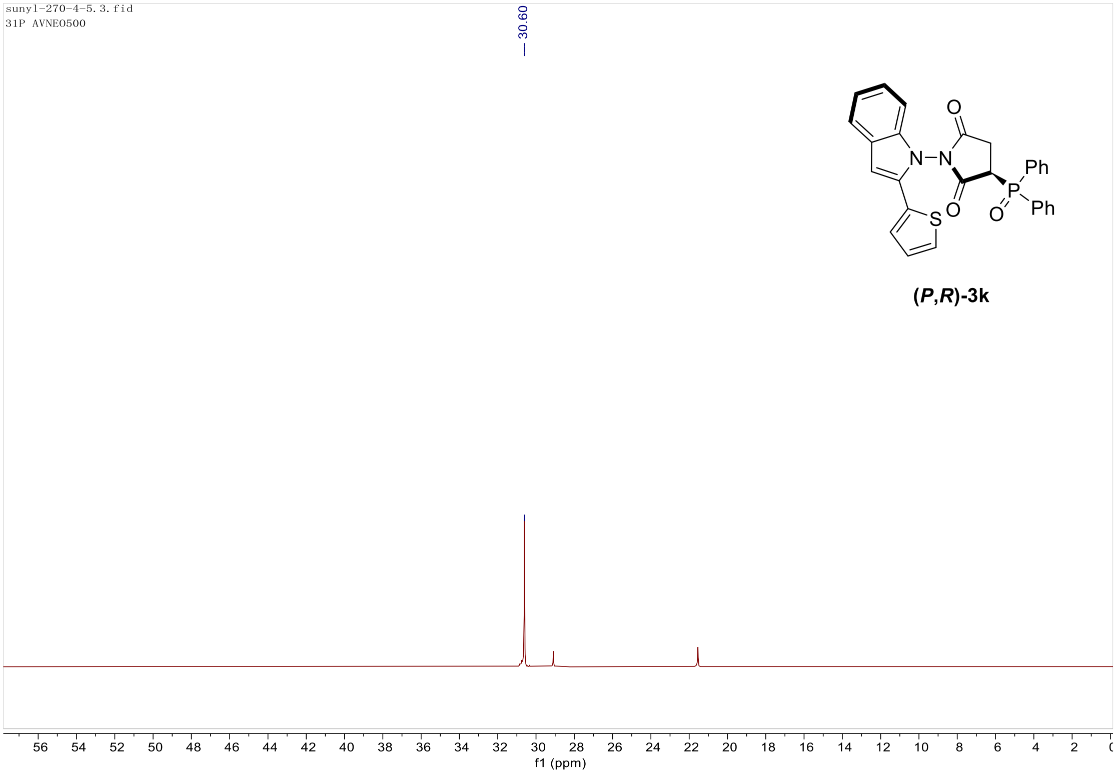
sunyl-270-4-5.3.fid
31P AVNEO500
30.60
(P,R)-3k
f1 (ppm)

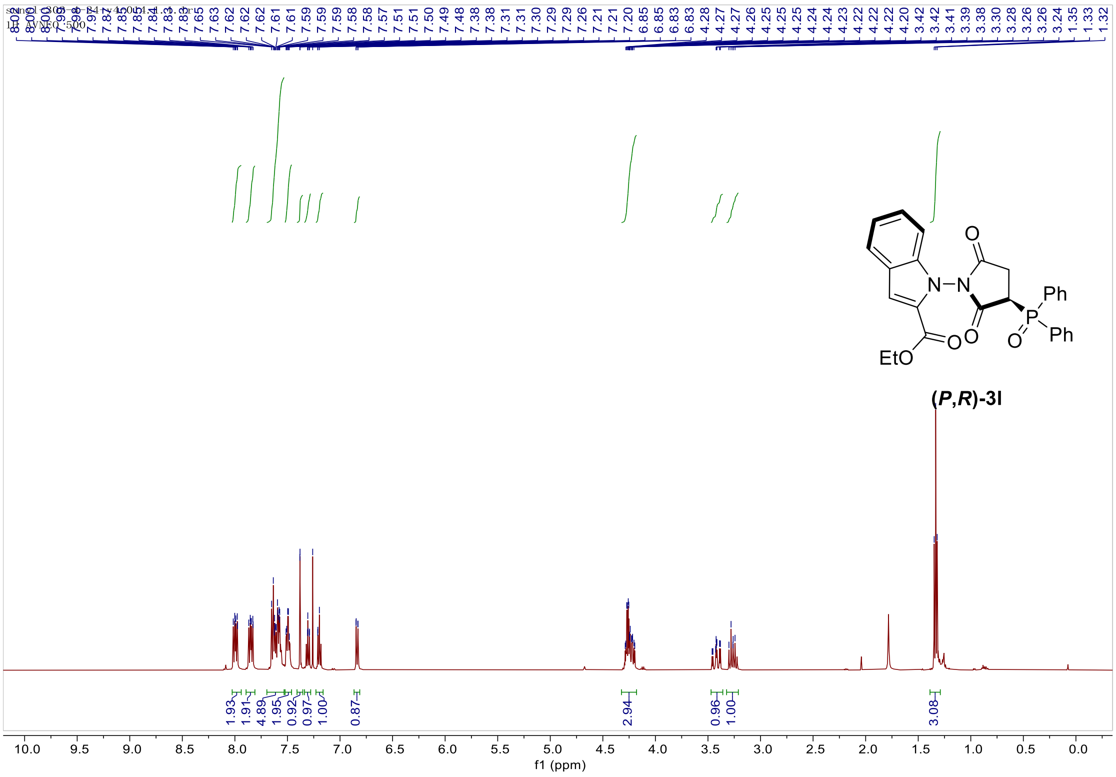
8.02 8.00 8.00 7.99 7.98 7.97 7.87 7.85 7.85 7.84 7.83 7.83 7.65 7.63 7.62 7.62 7.61 7.61 7.59 7.59 7.58 7.58 7.57 7.51 7.51 7.50 7.49 7.48 7.38 7.38 7.31 7.30 7.29 7.29 7.26 7.21 7.21 7.20 6.85 6.85 6.83 6.83 4.28 4.27 4.27 4.26 4.25 4.25 4.24 4.24 4.23 4.22 4.22 4.22 4.20 3.42 3.42 3.41 3.39 3.38 3.30 3.28 3.26 3.24 1.35 1.33 1.32
(P,R)-3l
1.93 1.91 4.89 1.95 0.92 0.97 1.00 0.87 2.94 0.96 1.00 3.08
f1 (ppm)

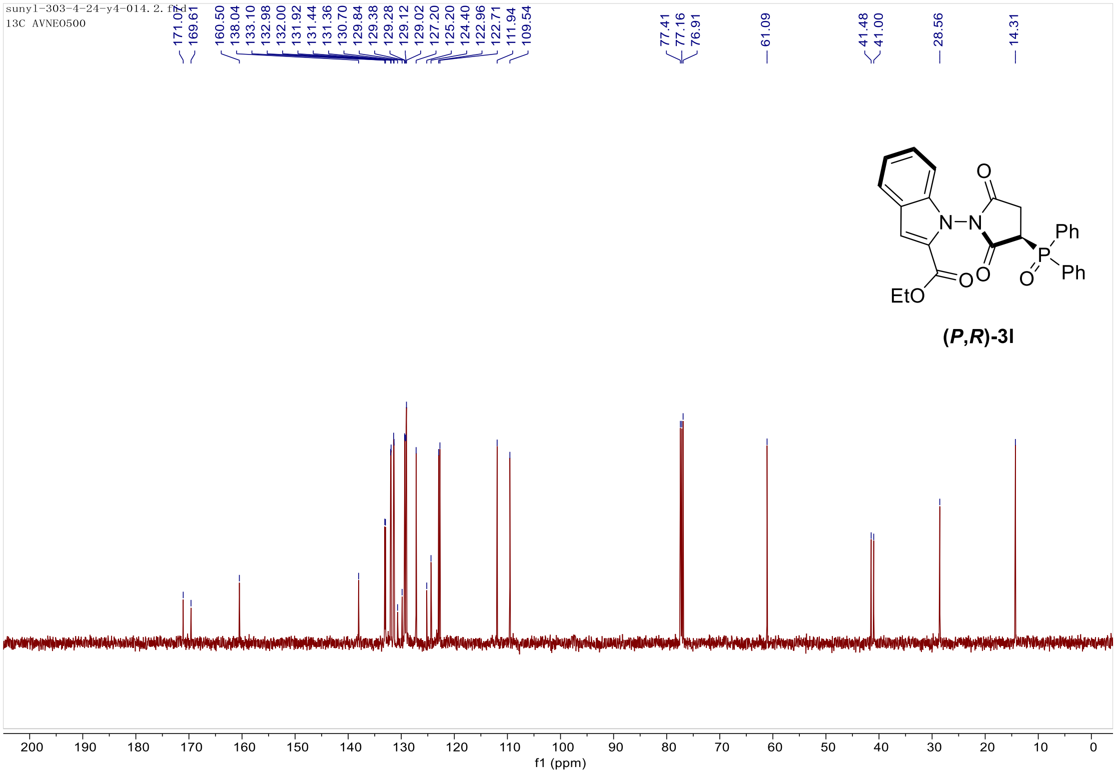
sunyl-303-4-24-y4-014.2.fid
13C AVNEO500
171.07
169.61
160.50
138.04
133.10
132.98
132.00
131.92
131.44
131.36
130.70
129.84
129.38
129.28
129.12
129.02
127.20
125.20
124.40
122.96
122.71
111.94
109.54
77.41
77.16
76.91
61.09
41.48
41.00
28.56
14.31
Ph
P
Ph
O
N
N
O
O
O
EtO
(P,R)-3l
f1 (ppm)

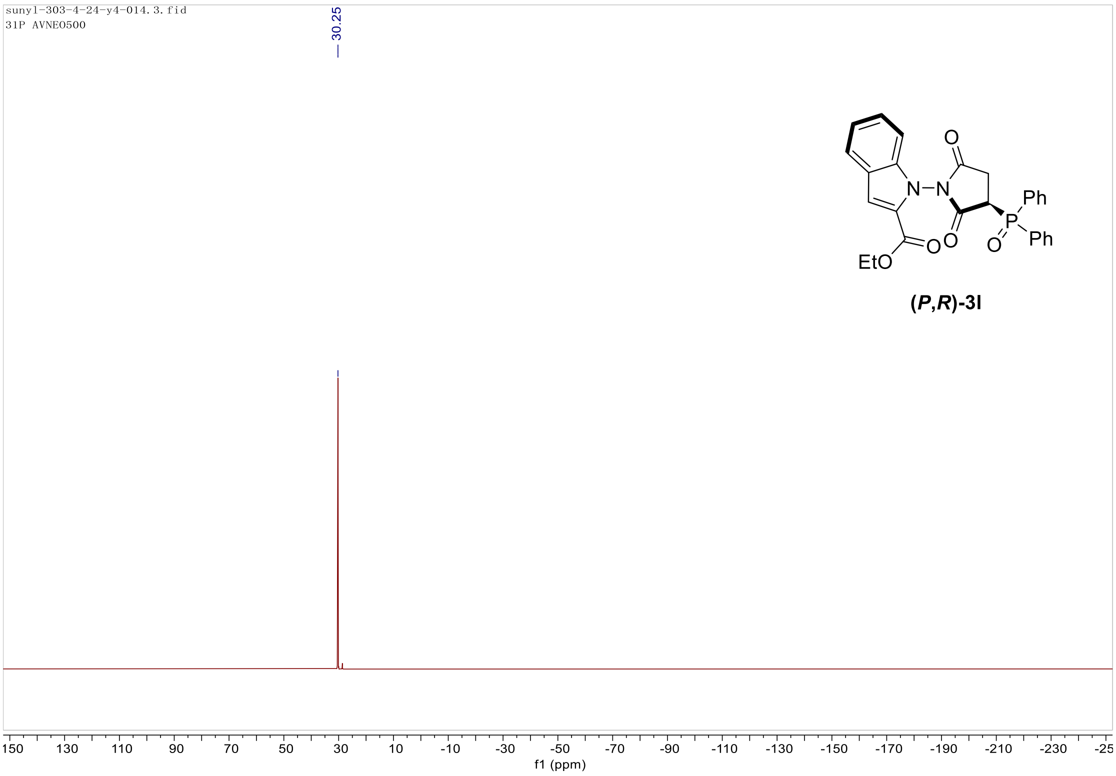
sunyl-303-4-24-y4-014.3.fid
31P AVNEO500
30.25
Ph
P
Ph
O
N
N
O
O
O
EtO
(P,R)-3l
f1 (ppm)

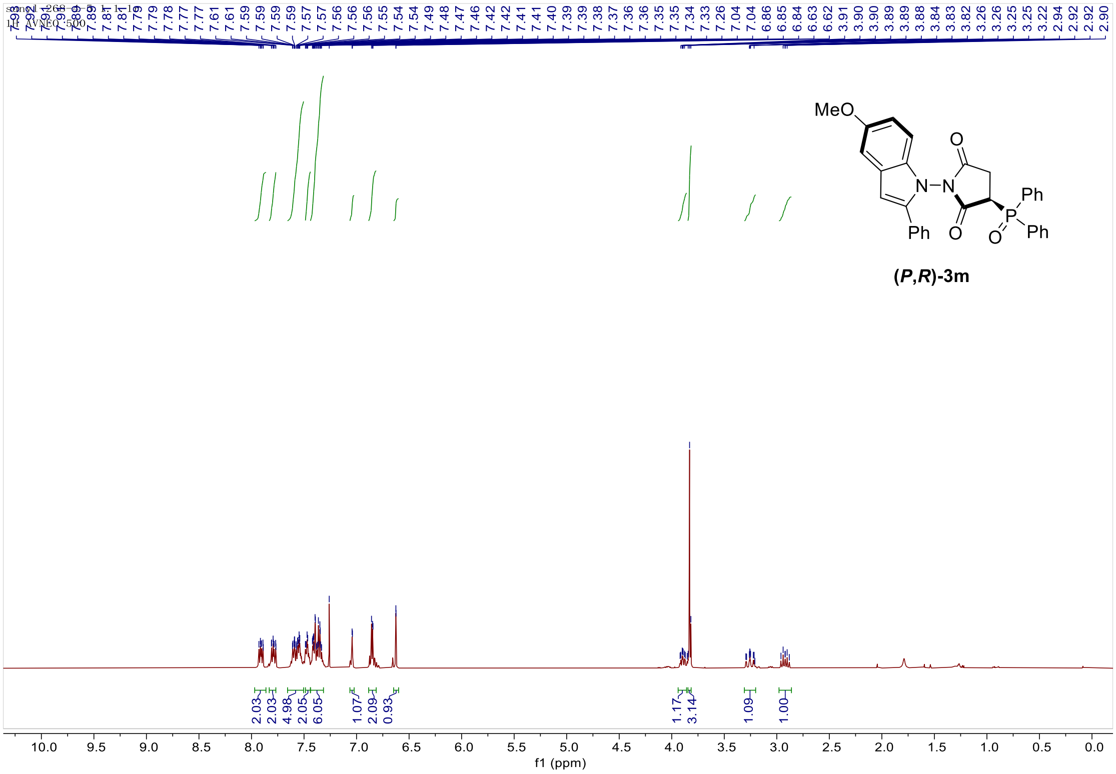

MeO
N–N
O
Ph
P
Ph
Ph
O
O
(P,R)-3m
2.03
2.03
4.98
2.05
6.05
1.07
2.09
0.93
1.17
3.14
1.09
1.00
f1 (ppm)

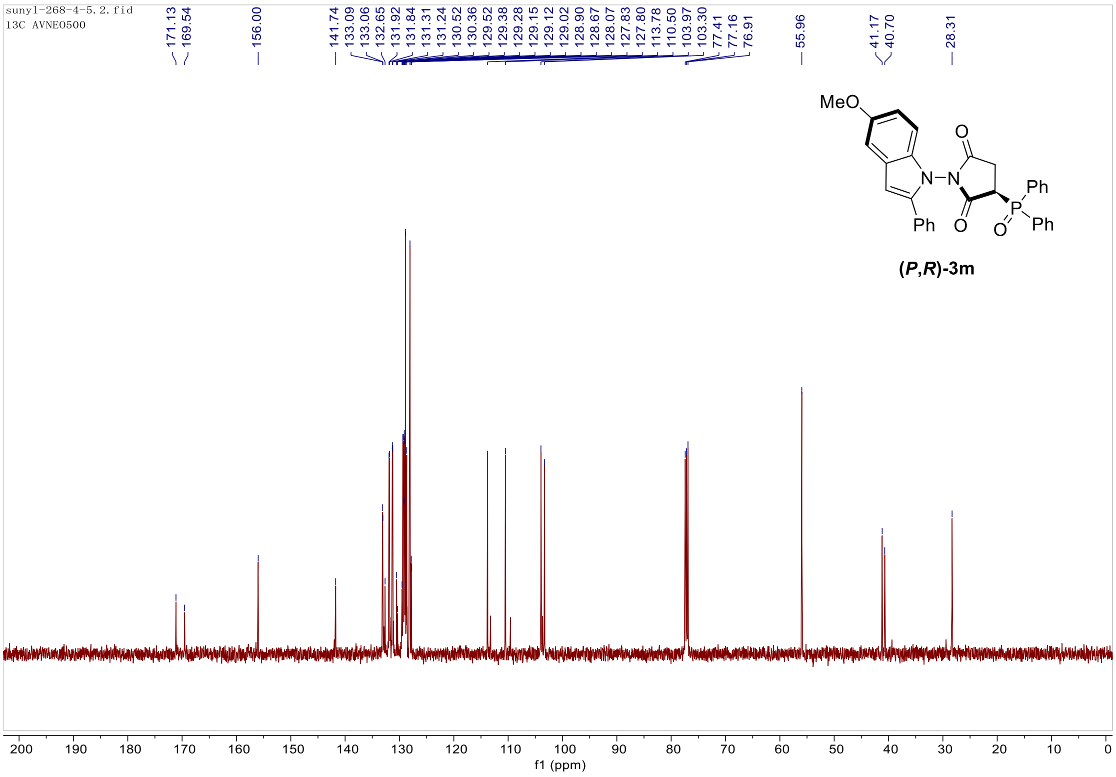

sunyl-268-4-5.2.fid
13C AVNEO500
171.13
169.54
156.00
141.74
133.09
133.06
132.65
131.92
131.84
131.31
131.24
130.52
130.36
129.52
129.38
129.28
129.15
129.12
129.02
128.90
128.67
128.07
127.83
127.80
113.78
110.50
103.97
103.30
77.41
77.16
76.91
55.96
41.17
40.70
28.31
MeO
N–N
O
Ph
P
Ph
Ph
O
O
(P,R)-3m
f1 (ppm)

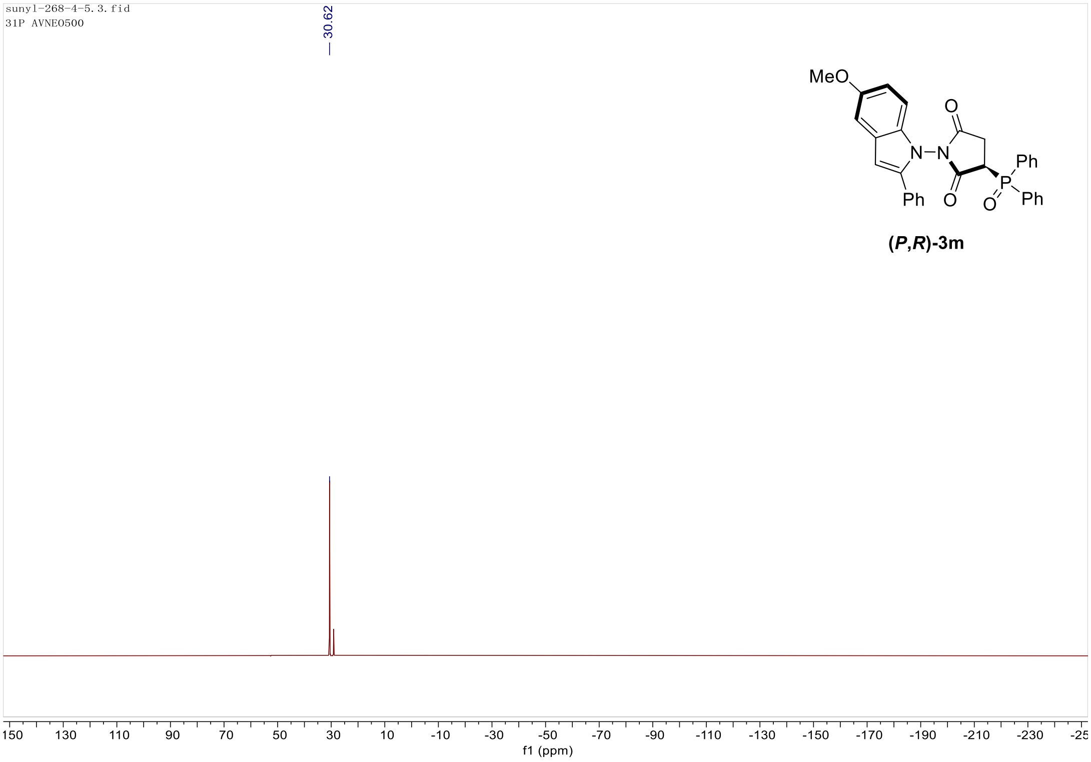
sunyl-268-4-5.3.fid
31P AVNE0500
30.62
MeO
N
N
O
O
P
O
Ph
Ph
Ph
(P,R)-3m
f1 (ppm)

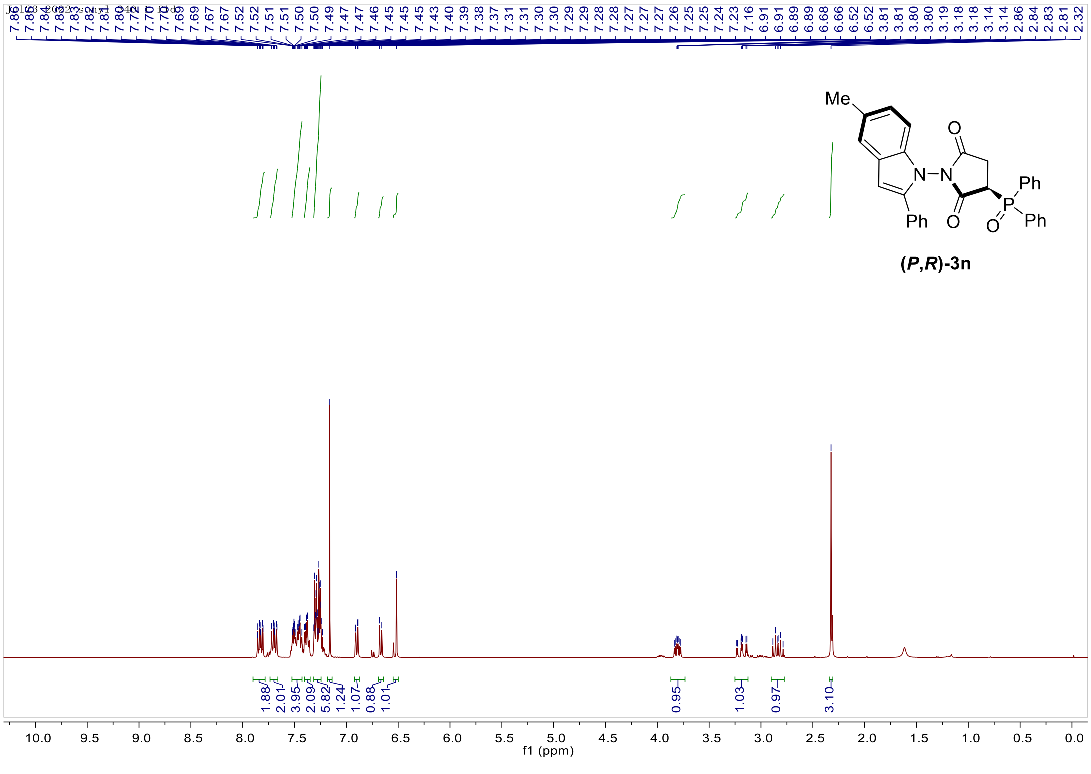
Me
N
N
O
O
P
O
Ph
Ph
Ph
(P,R)-3n
1.88
2.01
3.95
2.09
5.82
1.24
1.07
0.88
1.01
0.95
1.03
0.97
3.10
f1 (ppm)

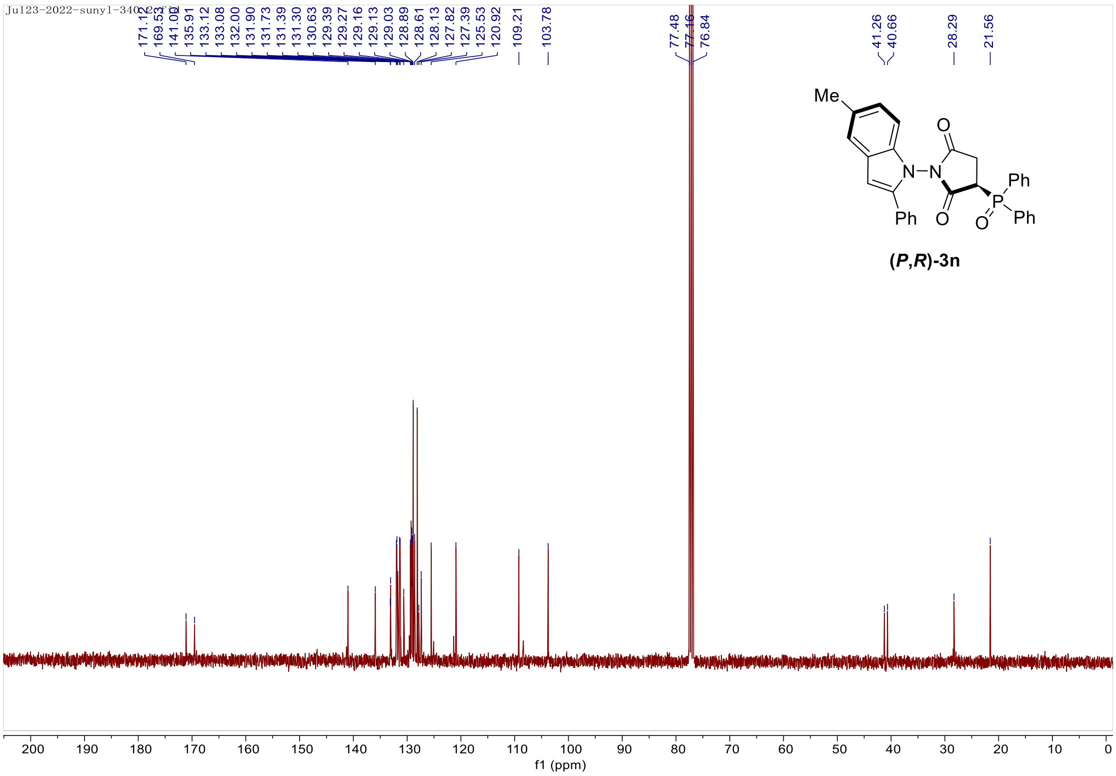
Jul23-2022-sunyl-340.2.fid
171.12
169.53
141.02
135.91
133.12
133.08
132.00
131.90
131.73
131.39
131.30
130.63
129.39
129.27
129.16
129.13
129.03
128.89
128.61
128.13
127.82
127.39
125.53
120.92
109.21
103.78
77.48
77.16
76.84
41.26
40.66
28.29
21.56
Me
N
N
O
O
Ph
P
O
Ph
Ph
(P,R)-3n
200
190
180
170
160
150
140
130
120
110
100
90
80
70
60
50
40
30
20
10
0
f1 (ppm)

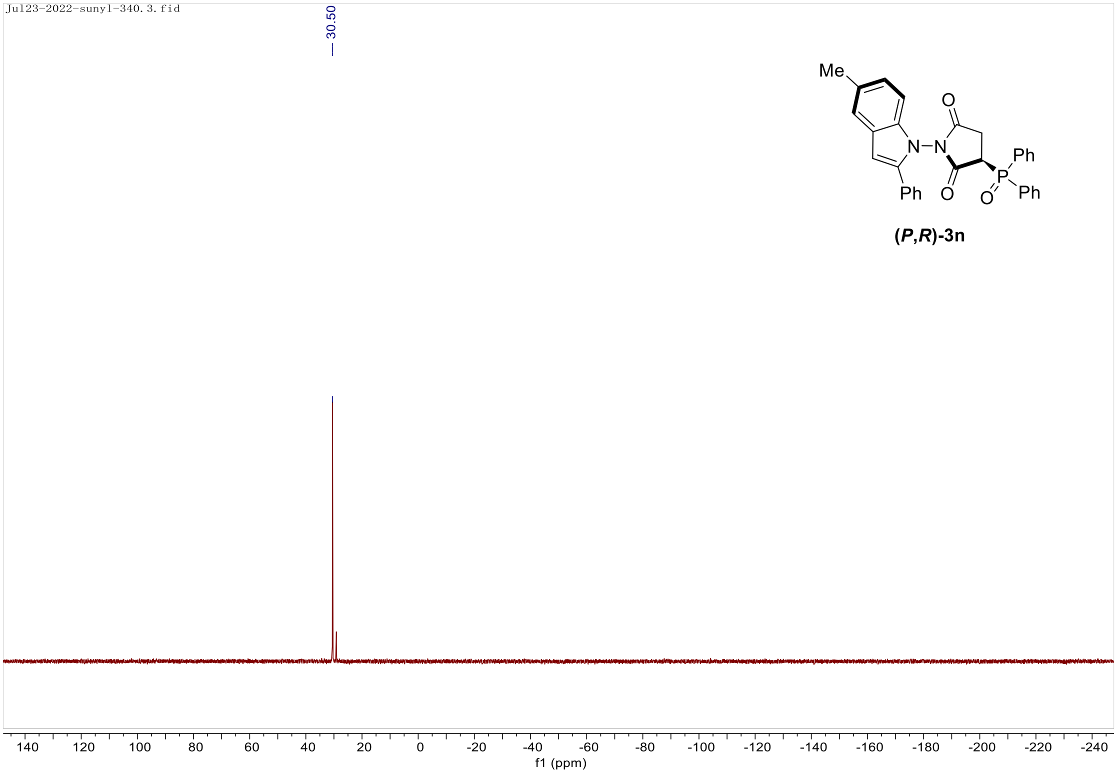
Jul23-2022-sunyl-340.3.fid
30.50
Me
N
N
O
O
Ph
P
O
Ph
Ph
(P,R)-3n
140
120
100
80
60
40
20
0
-20
-40
-60
-80
-100
-120
-140
-160
-180
-200
-220
-240
f1 (ppm)

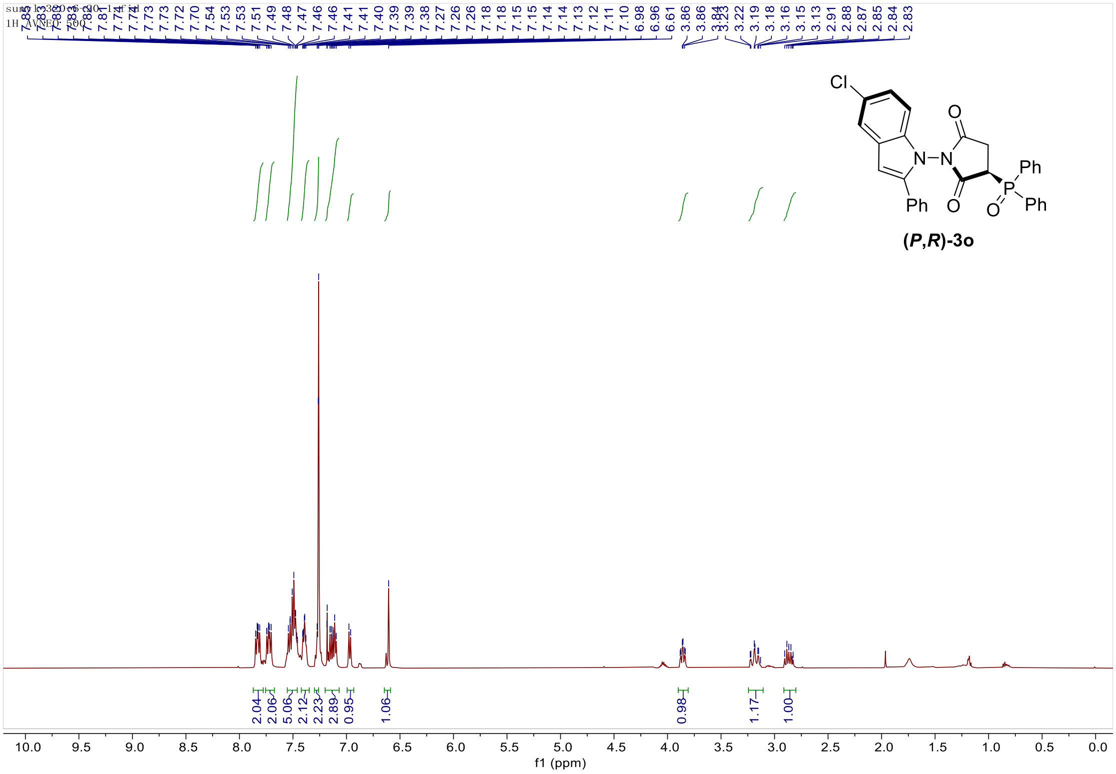
(P,R)-3o
f1 (ppm)

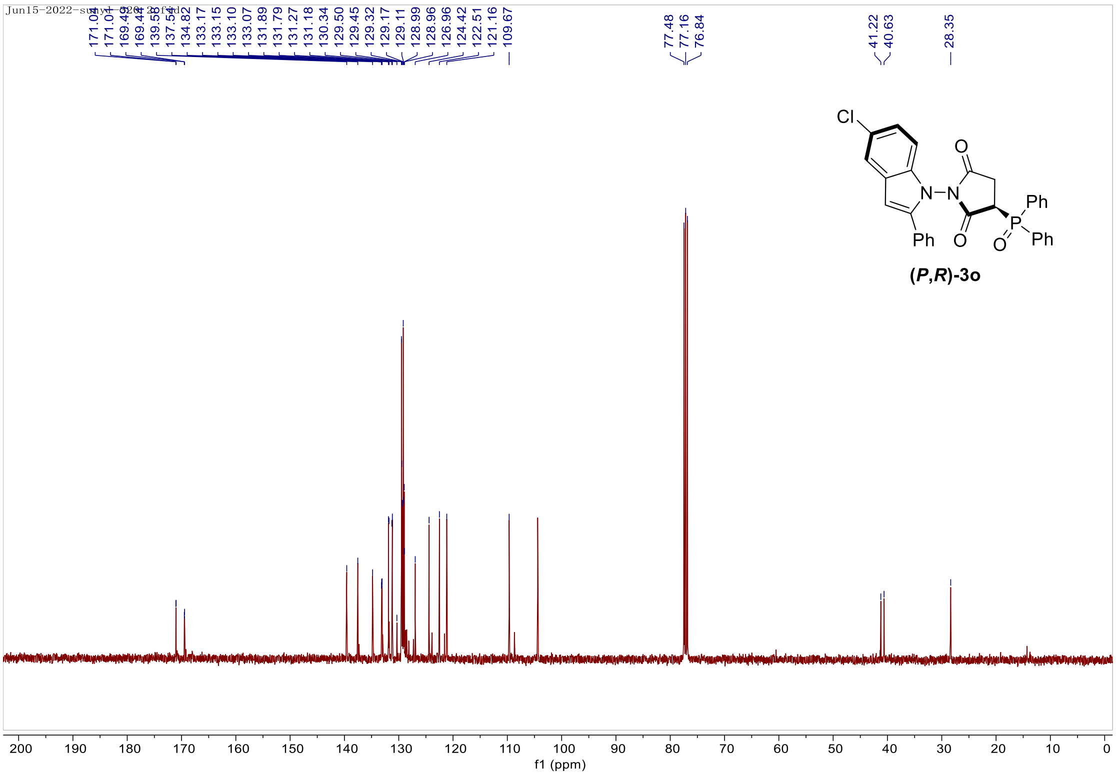
(P,R)-3o
f1 (ppm)

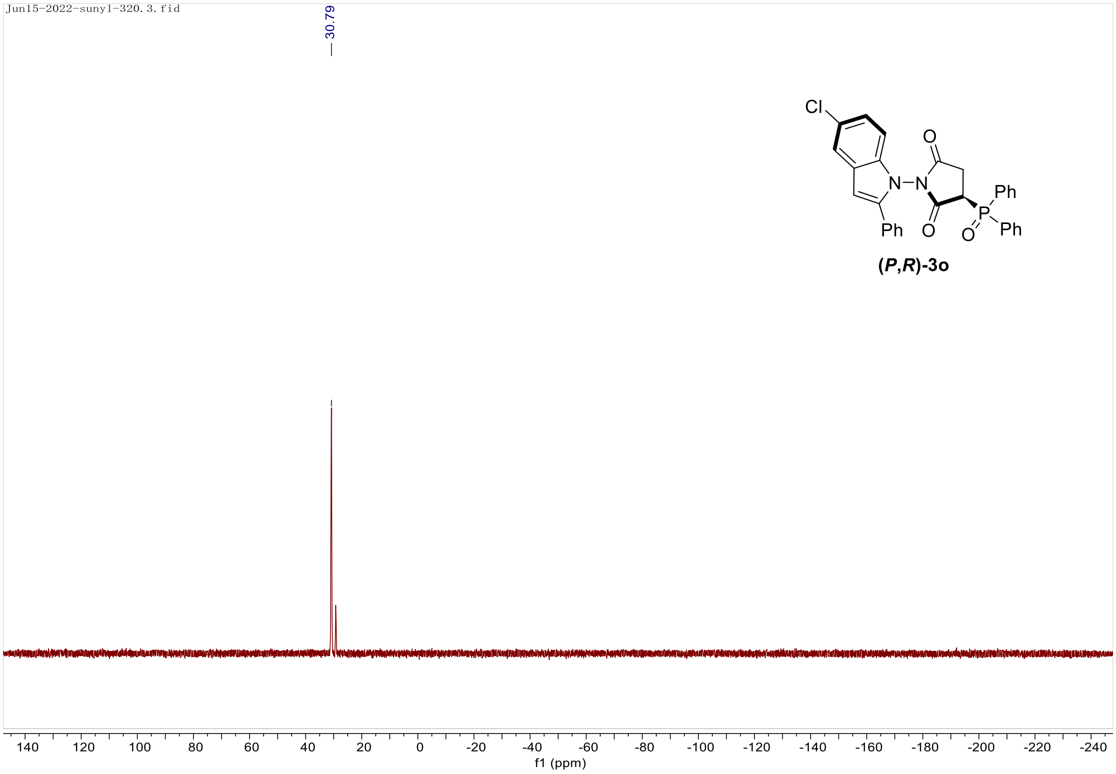
Jun15-2022-sunyl-320.3.fid
30.79
Cl
N–N
O
O
Ph
P
Ph
O
Ph
(P,R)-3o
f1 (ppm)

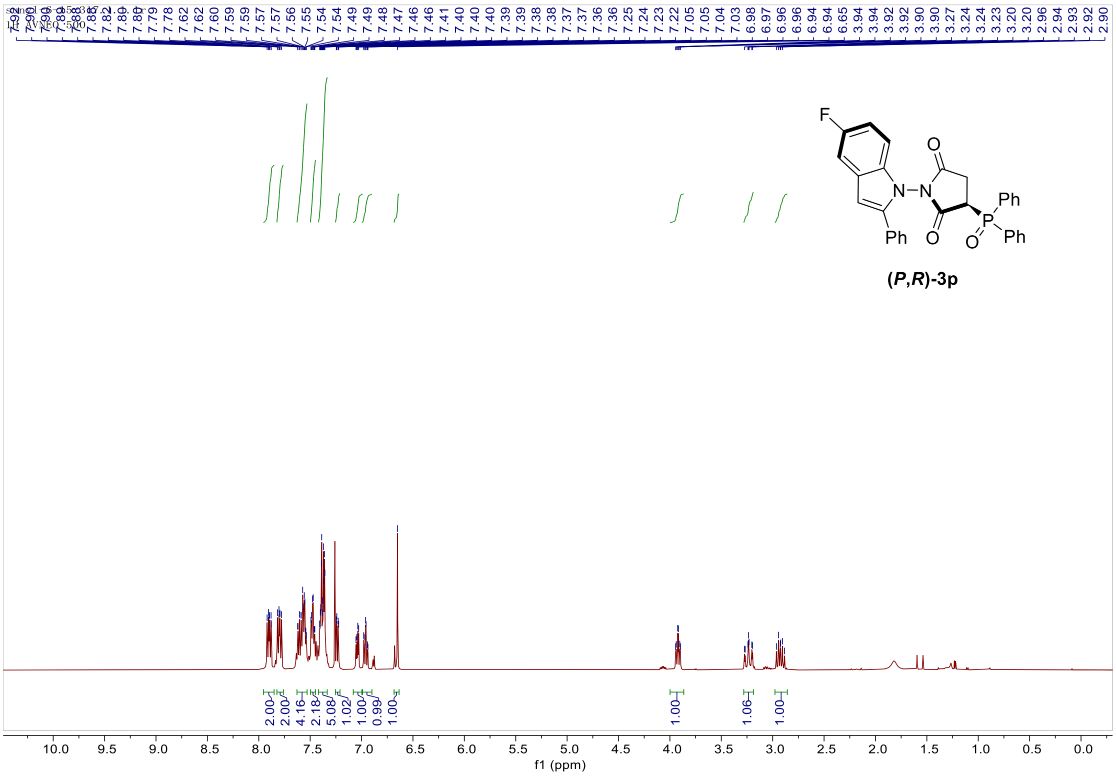
F
N–N
O
O
Ph
P
Ph
O
Ph
(P,R)-3p
2.00
2.00
4.16
2.18
5.08
1.02
1.00
0.99
1.00
1.00
1.06
1.00
f1 (ppm)

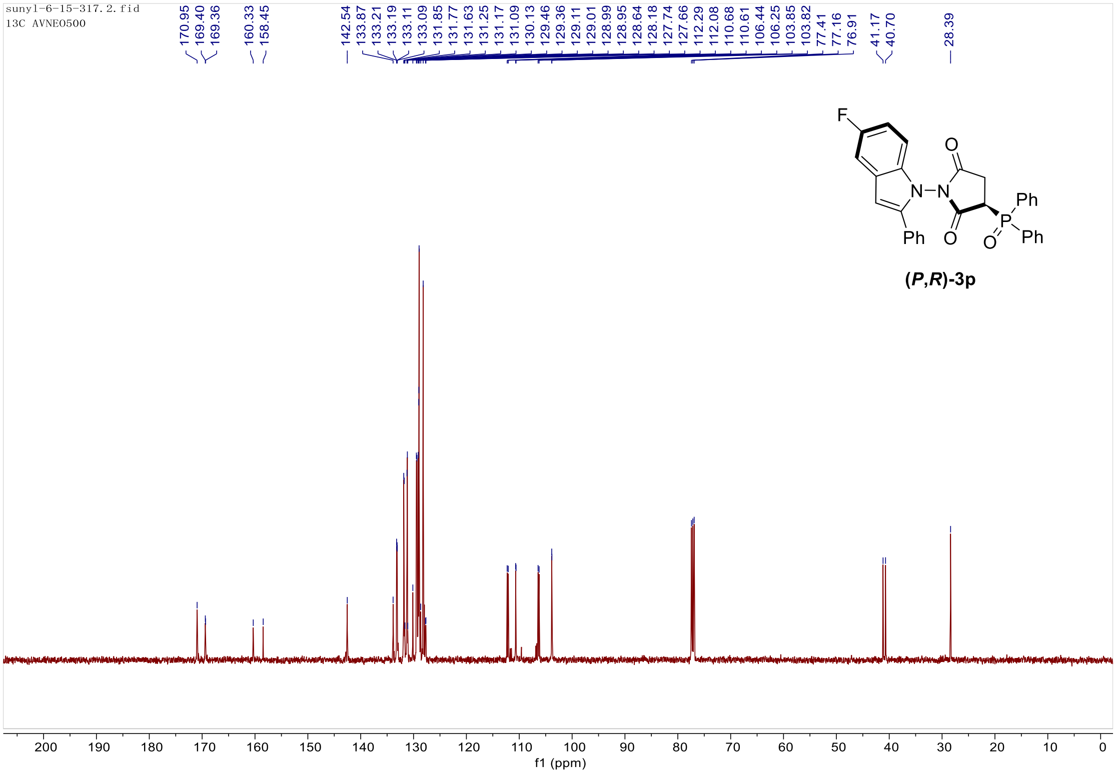
sunyl-6-15-317.2.fid
13C AVNEO500
170.95
169.40
169.36
160.33
158.45
142.54
133.87
133.21
133.19
133.11
133.09
131.85
131.77
131.63
131.25
131.17
131.09
130.13
129.46
129.36
129.11
129.01
128.99
128.95
128.64
128.18
127.74
127.66
112.29
112.08
110.68
110.61
106.44
106.25
103.85
103.82
77.41
77.16
76.91
41.17
40.70
28.39
F
N
N
O
O
Ph
P
O
Ph
Ph
(*P*,*R*)-3p
200 190 180 170 160 150 140 130 120 110 100 90 80 70 60 50 40 30 20 10 0
f1 (ppm)

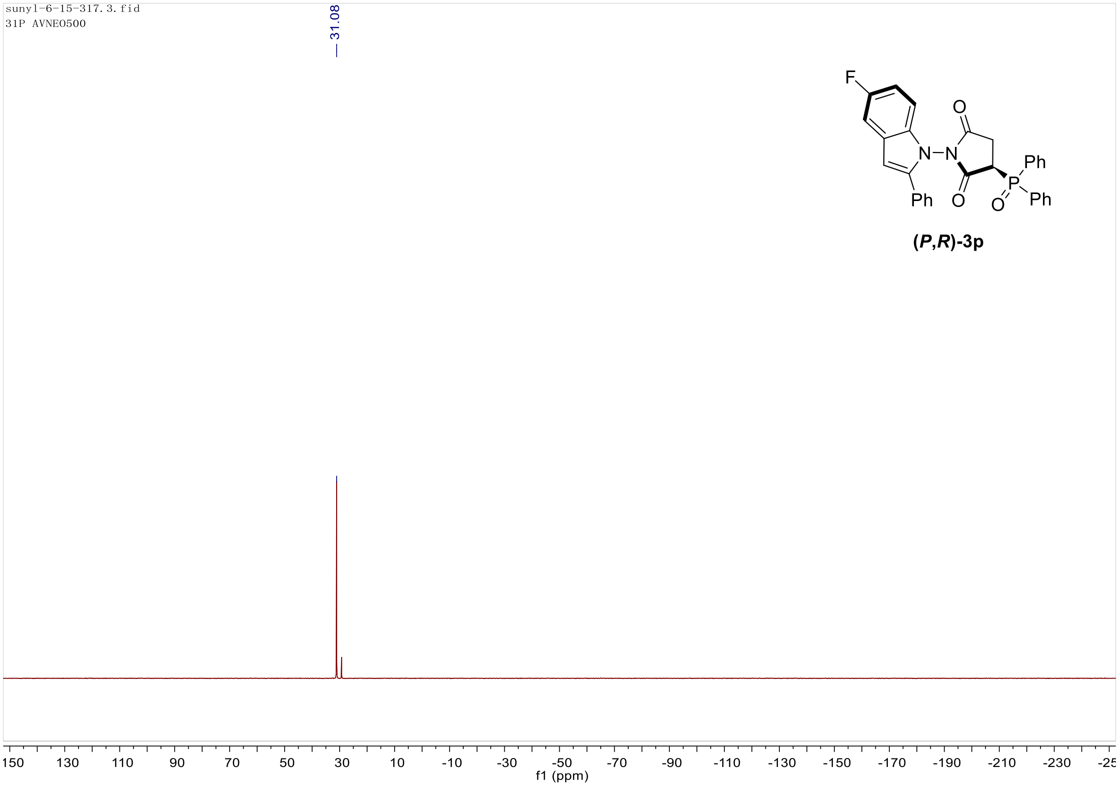
sunyl-6-15-317.3.fid
31P AVNEO500
31.08
F
N
N
O
O
Ph
P
O
Ph
Ph
(*P*,*R*)-3p
150 130 110 90 70 50 30 10 -10 -30 -50 -70 -90 -110 -130 -150 -170 -190 -210 -230
f1 (ppm)

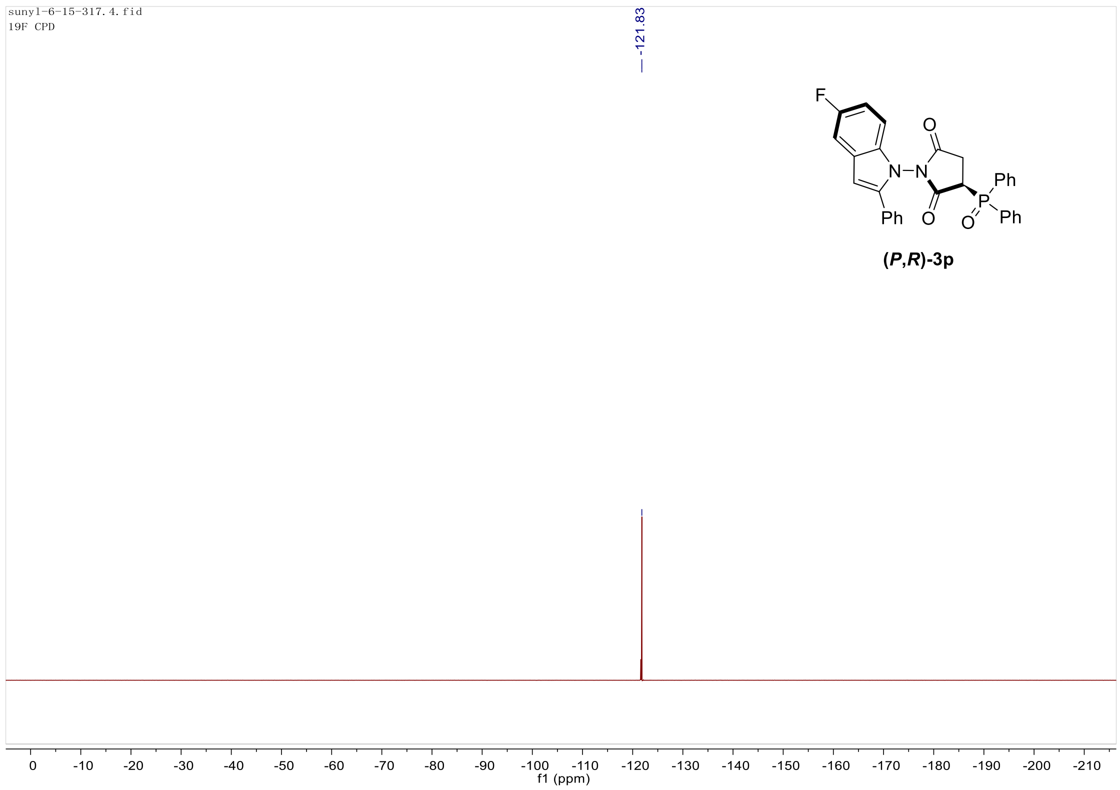
sunyl-6-15-317.4.fid
19F CPD
-121.83
F
N
N
O
O
Ph
P
O
Ph
Ph
(P,R)-3p
f1 (ppm)

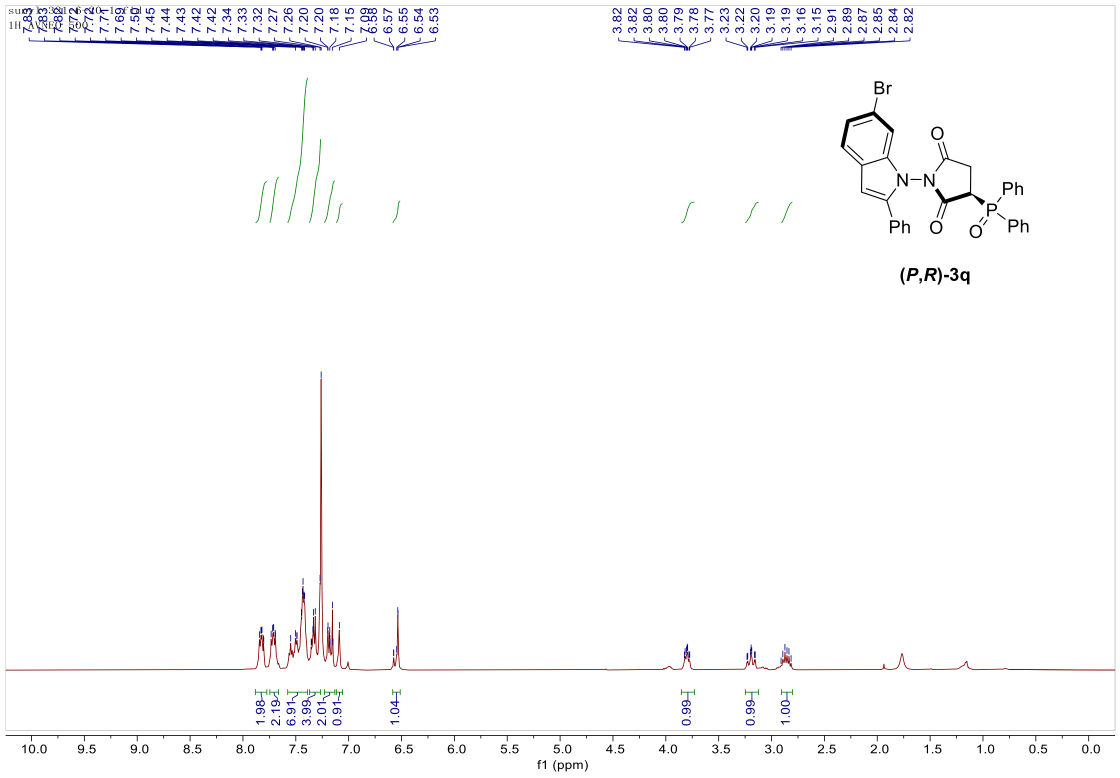
Br
N
N
O
O
Ph
P
O
Ph
Ph
(P,R)-3q
f1 (ppm)

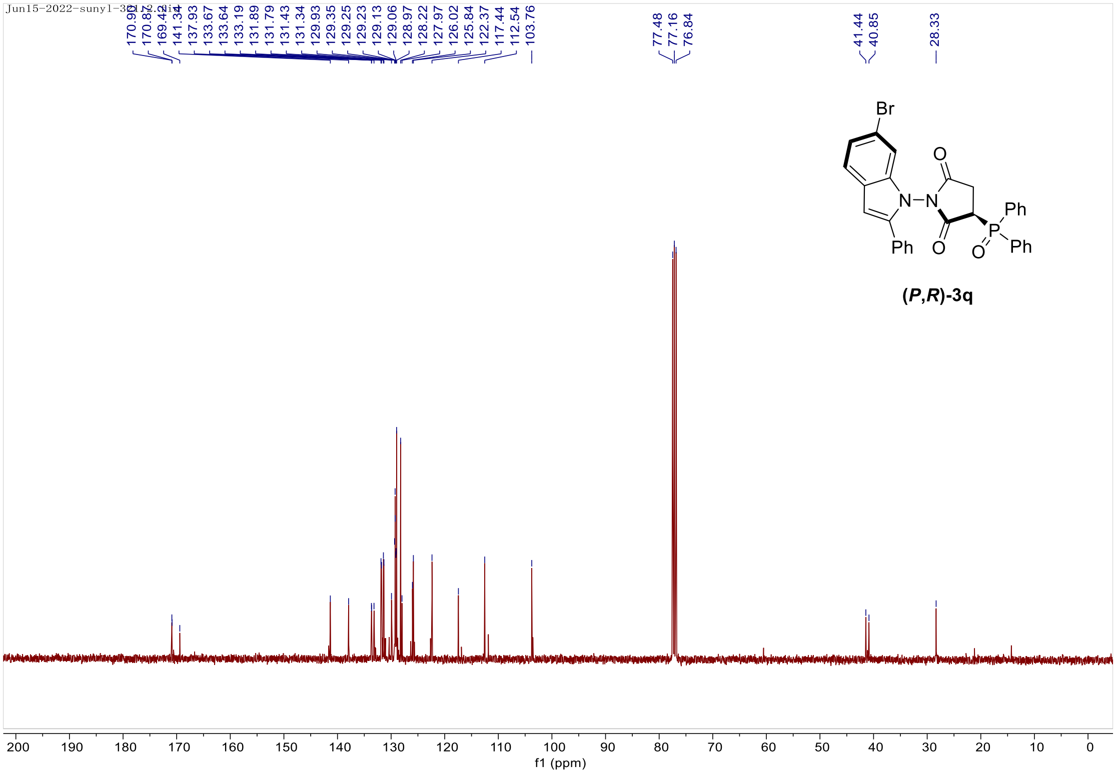
Jun15-2022-sunyl-321
170.92
170.87
169.42
141.34
137.93
133.67
133.64
133.19
131.89
131.79
131.43
131.34
129.93
129.35
129.25
129.23
129.13
129.06
128.97
128.22
127.97
126.02
125.84
122.37
117.44
112.54
103.76
77.48
77.16
76.84
41.44
40.85
28.33
Br
O
N
N
Ph
Ph
O
P
O
Ph
(P,R)-3q
f1 (ppm)

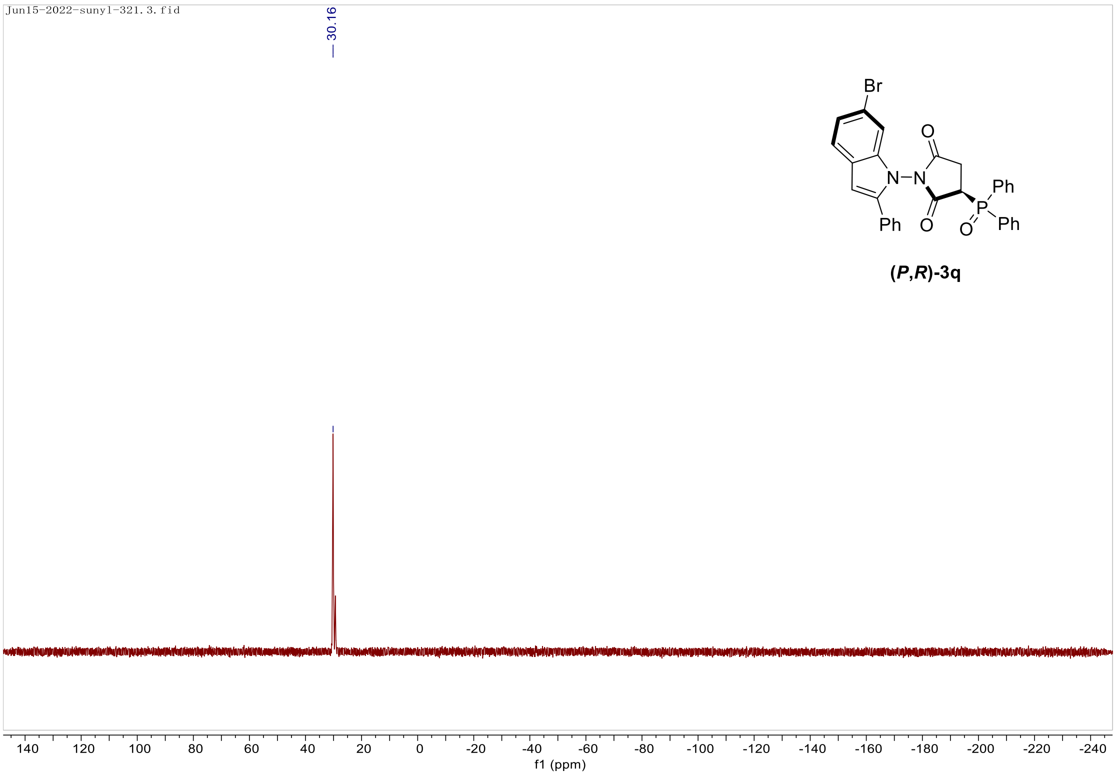
Jun15-2022-sunyl-321.3.fid
30.16
Br
O
N
N
Ph
Ph
O
P
O
Ph
(P,R)-3q
f1 (ppm)

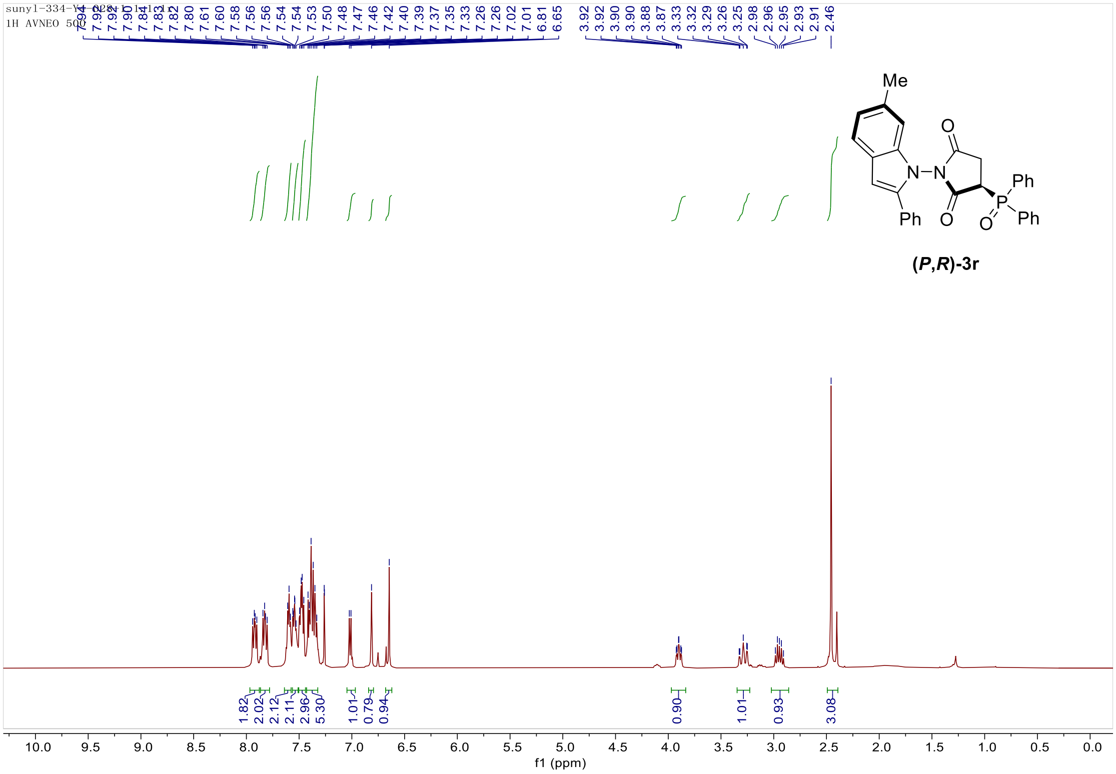
suny1-334-Y4-028-1.1.fid
1H AVNEO 500
(P,R)-3r
Me
Ph
f1 (ppm)

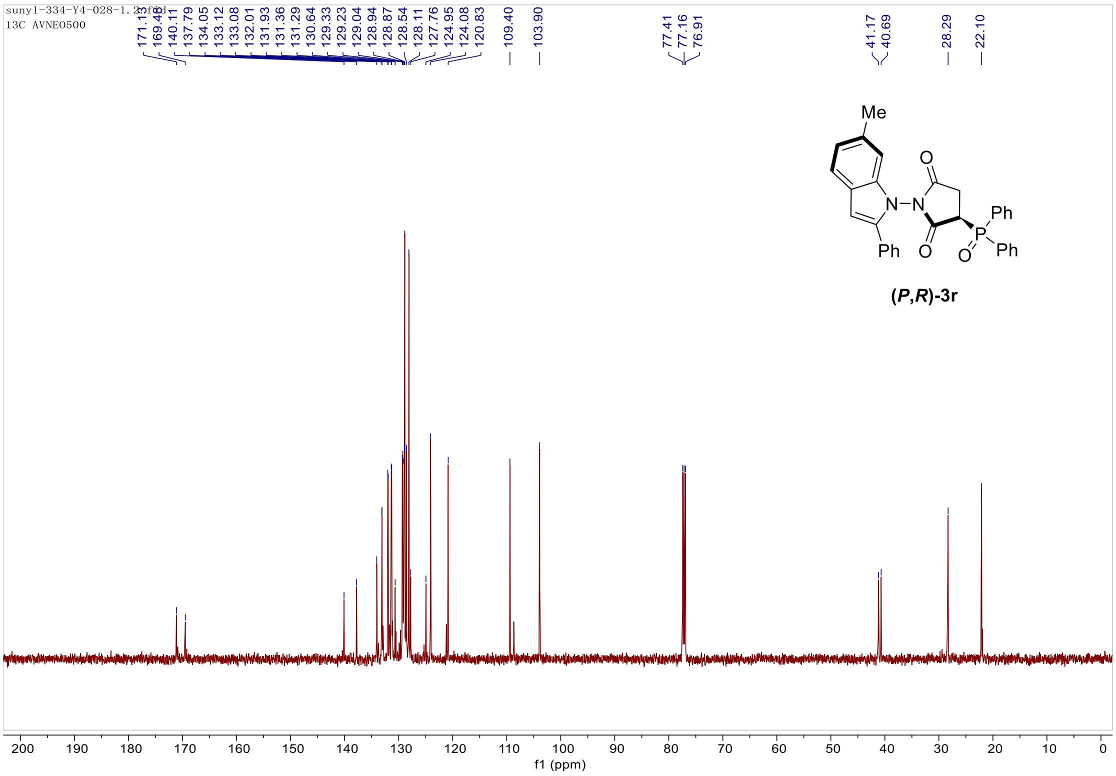
suny1-334-Y4-028-1.2.fid
13C AVNEO500
(P,R)-3r
Me
Ph
f1 (ppm)

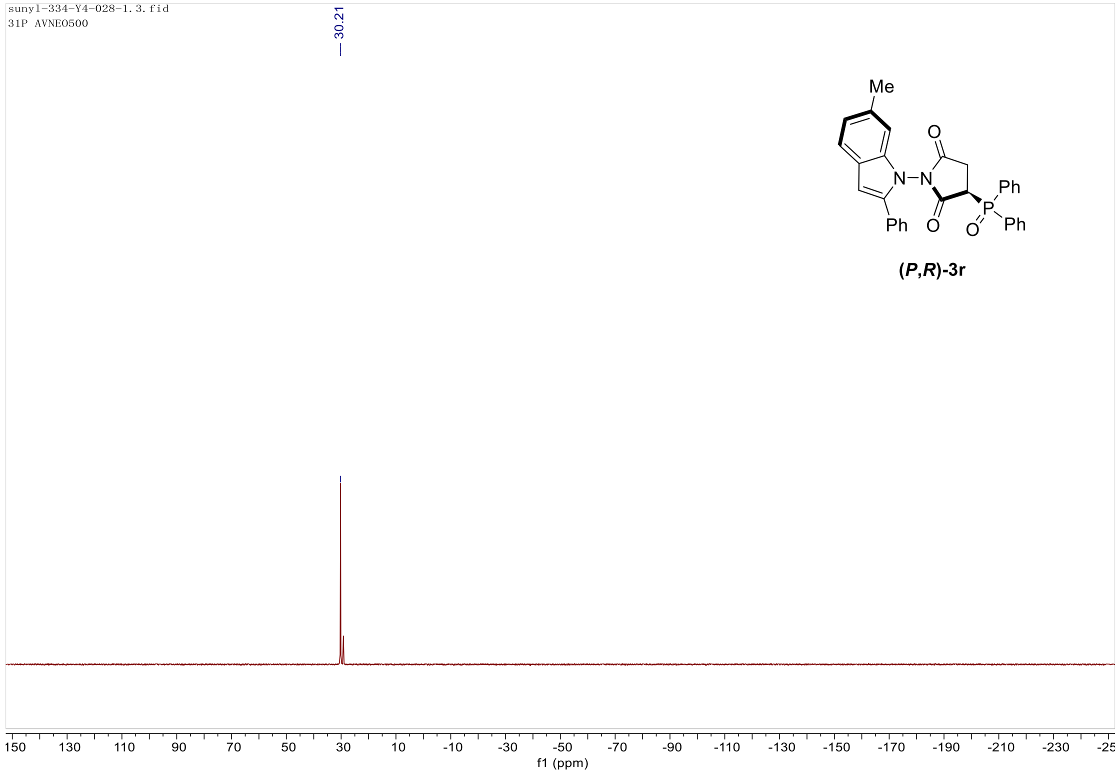
sunyl-334-Y4-028-1.3.fid
31P AVNEO500
30.21
(P,R)-3r
f1 (ppm)

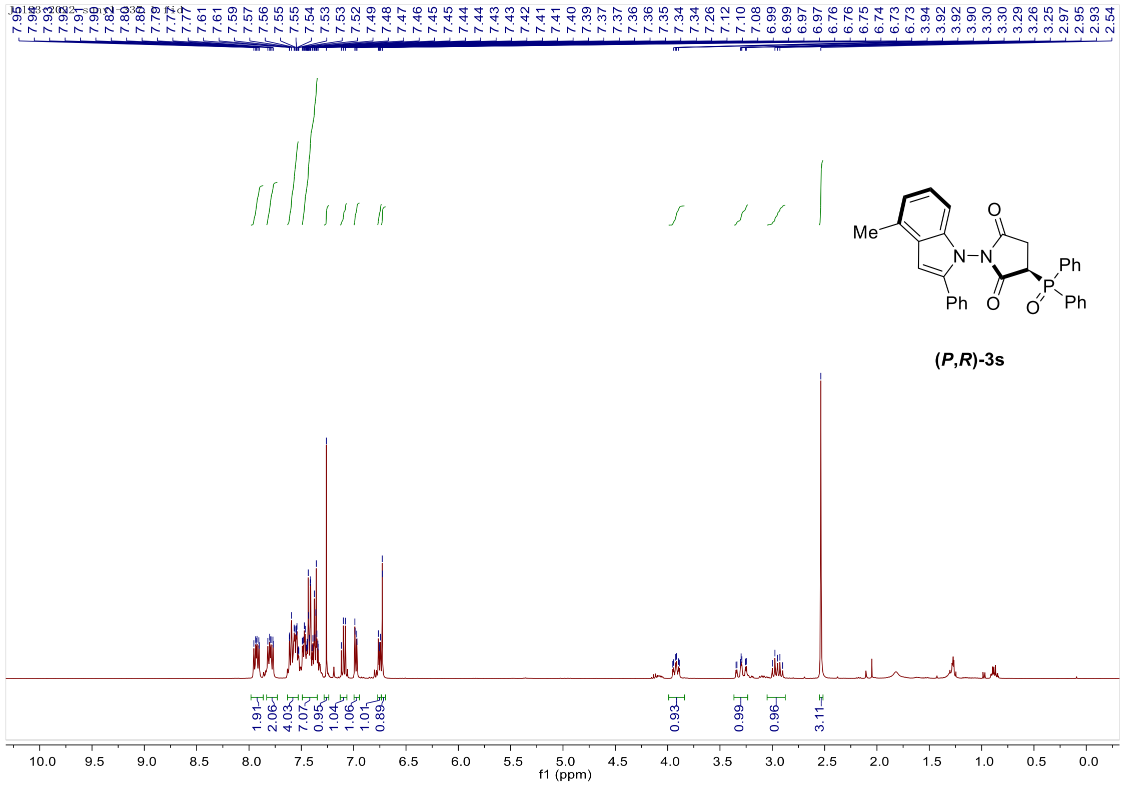
(P,R)-3s
f1 (ppm)

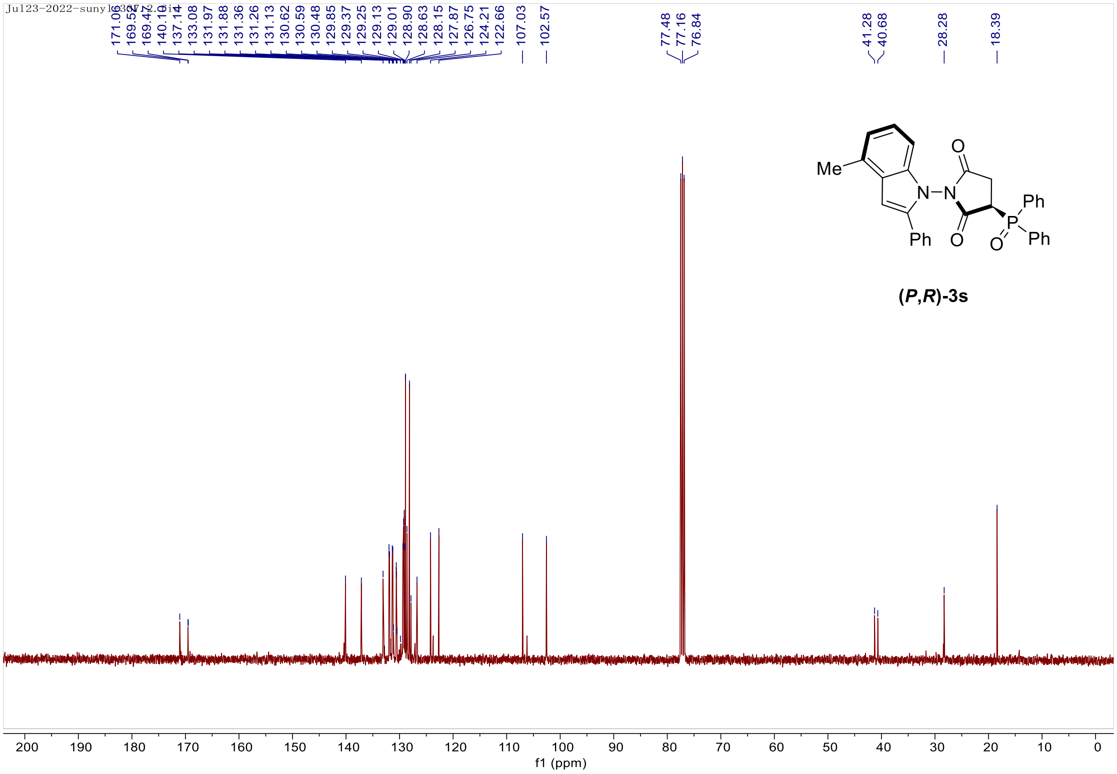
Jul23-2022-sunyl-337
171.06
169.52
169.47
140.10
137.14
133.08
131.97
131.88
131.36
131.26
131.13
130.62
130.59
130.48
129.85
129.37
129.25
129.13
129.01
128.90
128.63
128.15
127.87
126.75
124.21
122.66
107.03
102.57
77.48
77.16
76.84
41.28
40.68
28.28
18.39
Me
N
N
O
O
P
O
Ph
Ph
Ph
(P,R)-3s
f1 (ppm)

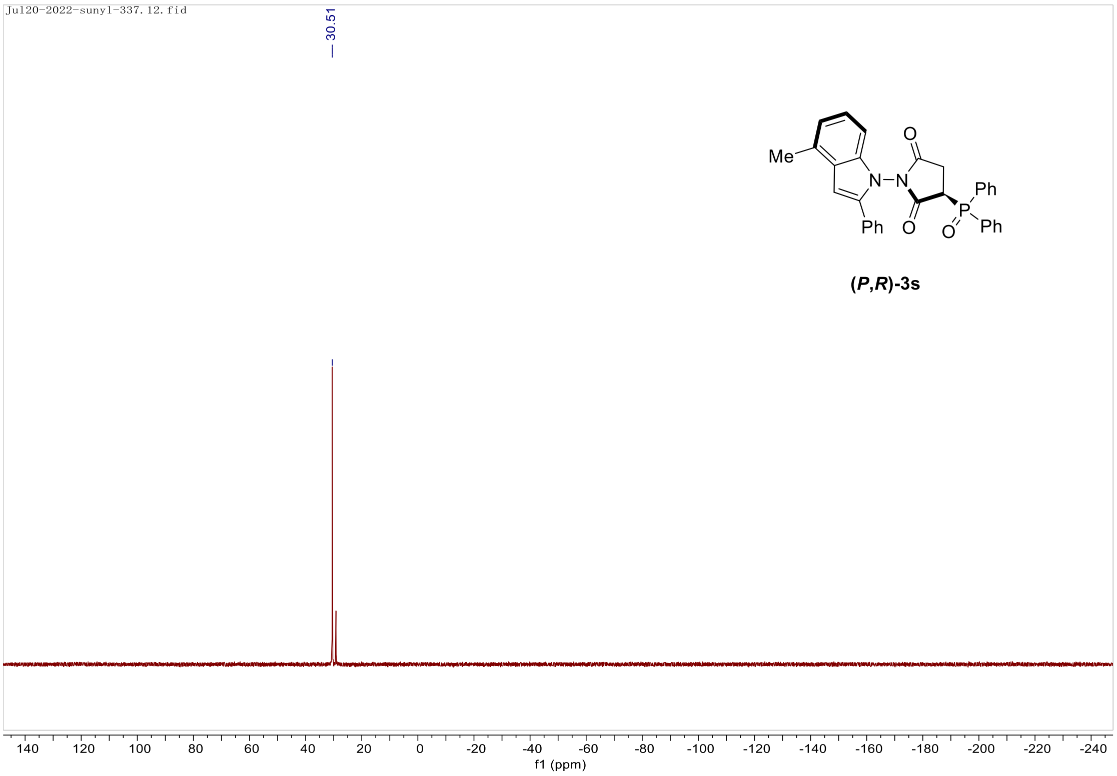
Jul20-2022-sunyl-337.12.fid
30.51
Me
N
N
O
O
P
O
Ph
Ph
Ph
(P,R)-3s
f1 (ppm)

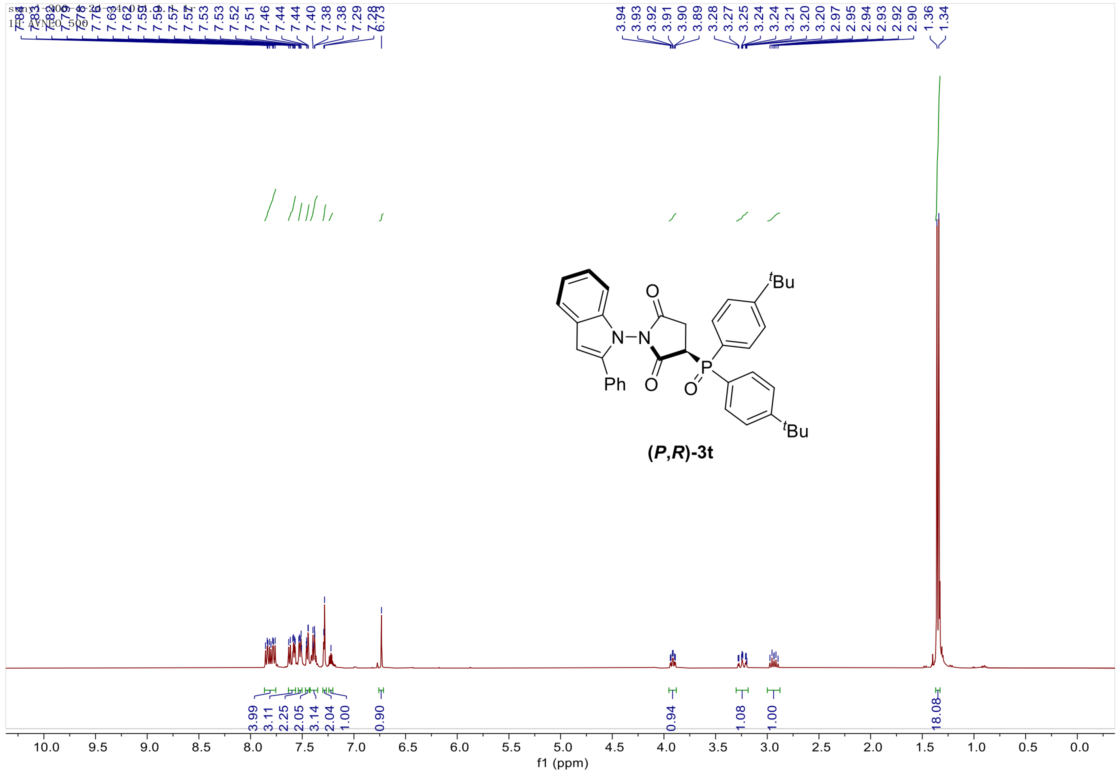
(P,R)-3t
f1 (ppm)

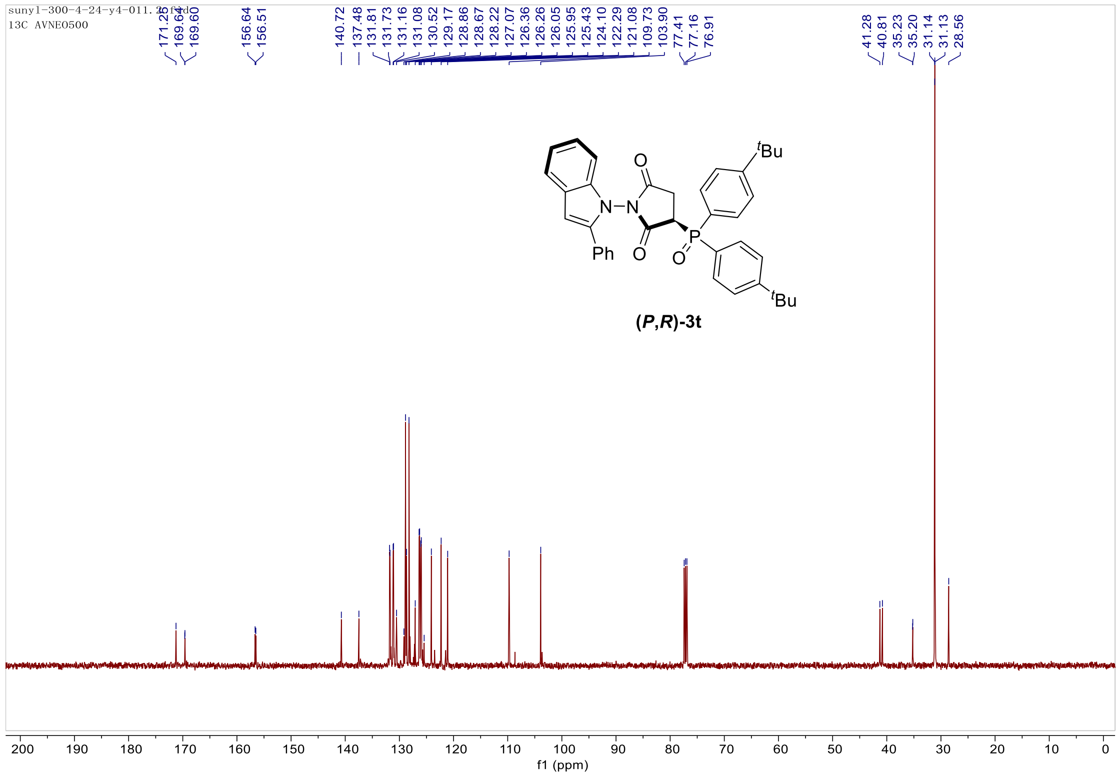
13C AVNE0500
(P,R)-3t
f1 (ppm)

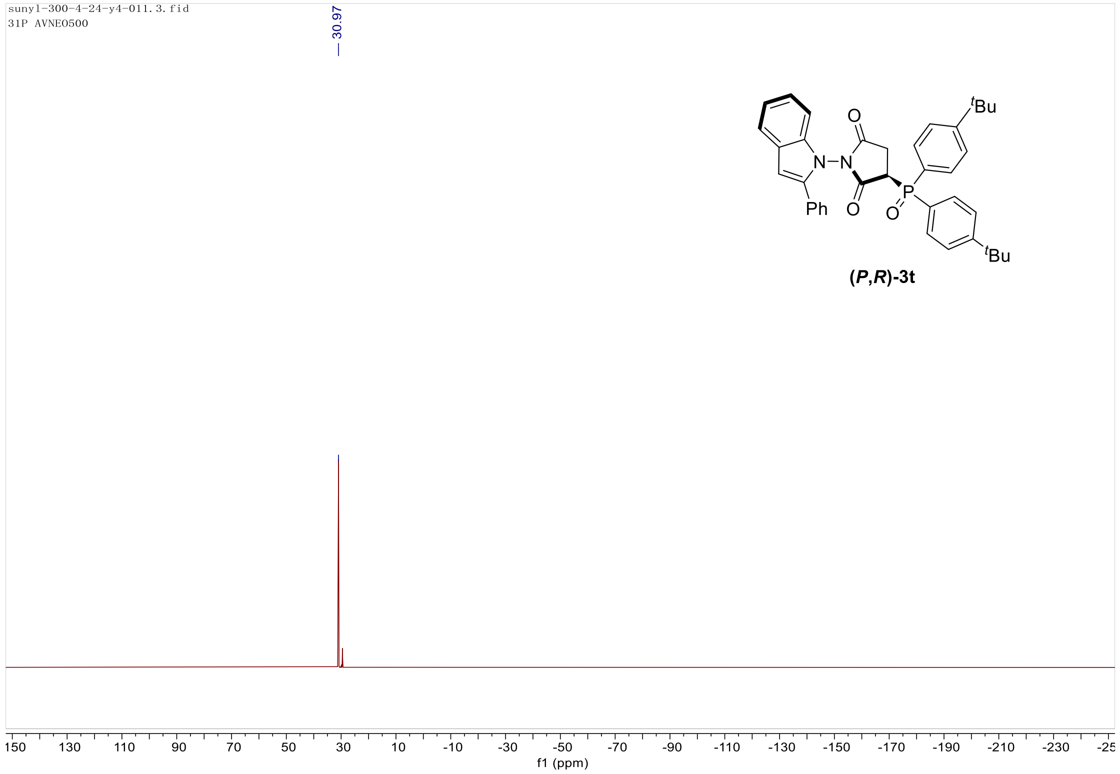

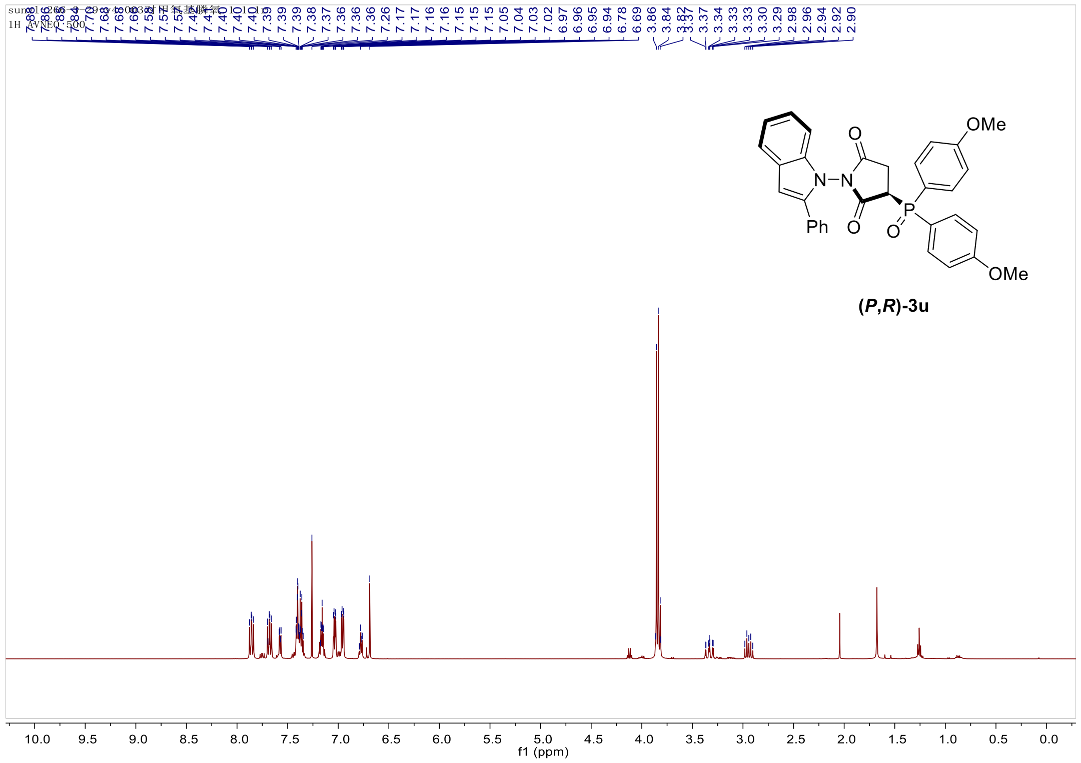

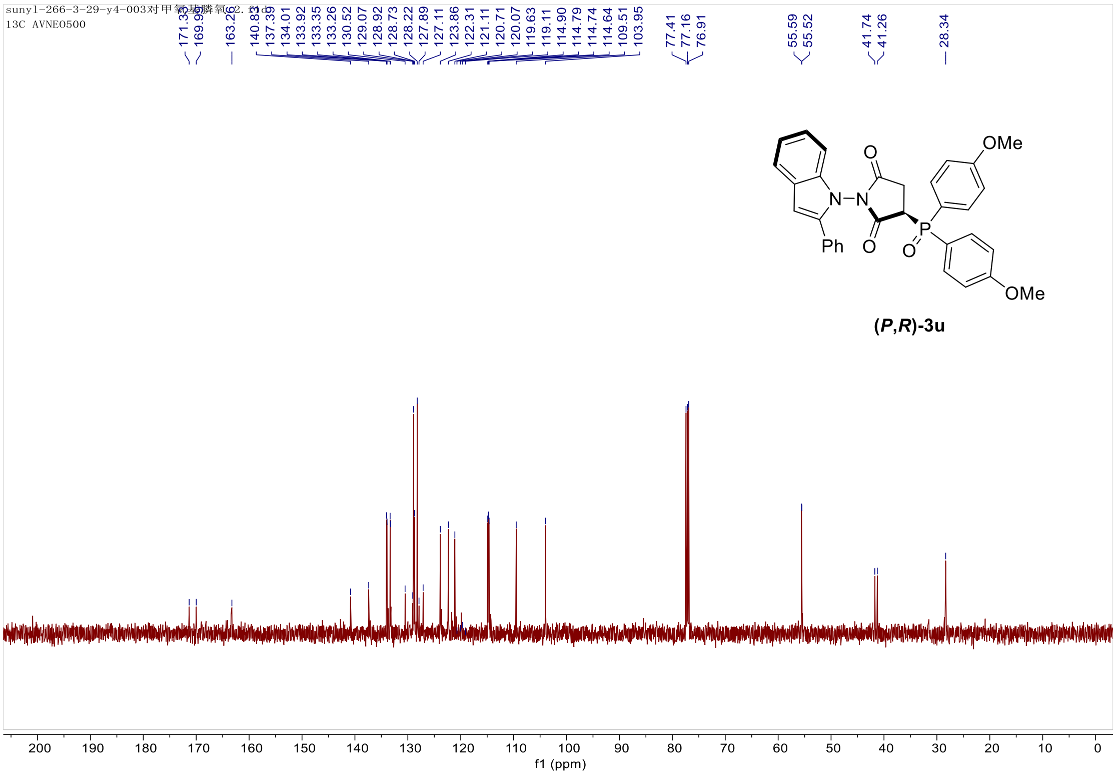
sunyl-266-3-29-y4-003对甲氧基膦氧.2.fid
13C AVNEO500
171.36
169.99
163.26
140.83
137.39
134.01
133.92
133.35
133.26
130.52
129.07
128.92
128.73
128.22
127.89
127.11
123.86
122.31
121.11
120.71
120.07
119.63
119.11
114.90
114.79
114.74
114.64
109.51
103.95
77.41
77.16
76.91
55.59
55.52
41.74
41.26
28.34
OMe
N–N
O
O
P
O
Ph
OMe
(P,R)-3u
f1 (ppm)

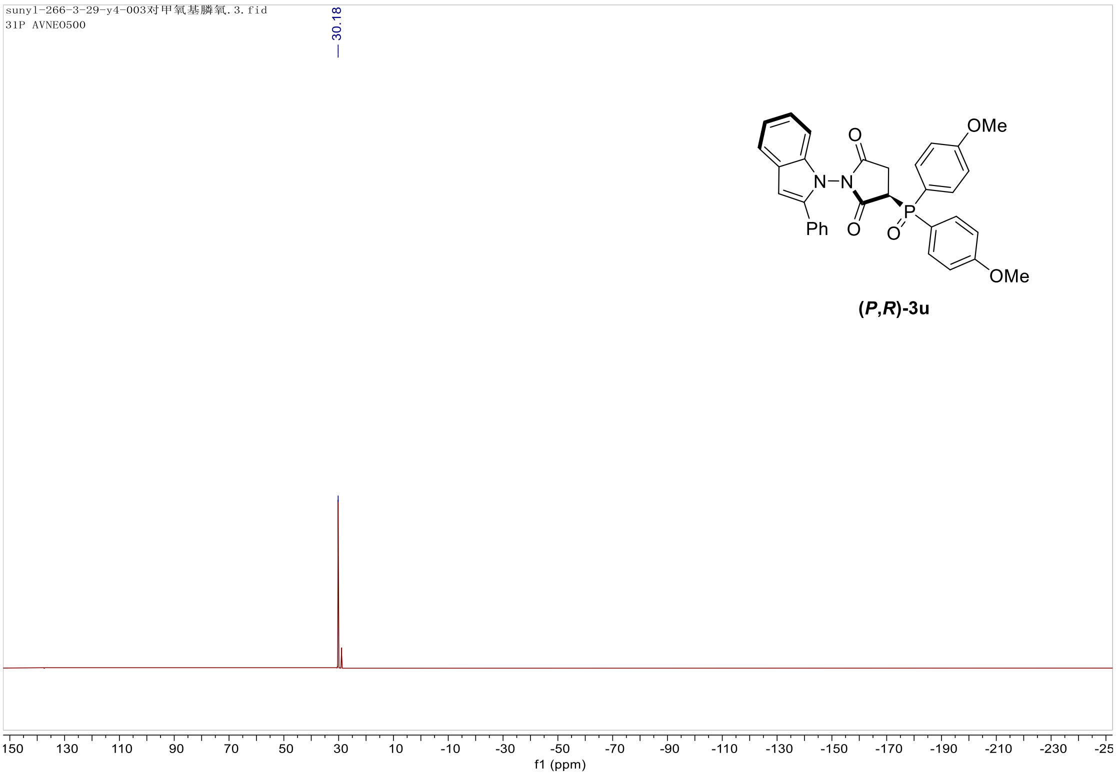
sunyl-266-3-29-y4-003对甲氧基膦氧.3.fid
31P AVNEO500
30.18
OMe
N–N
O
O
P
O
Ph
OMe
(P,R)-3u
f1 (ppm)

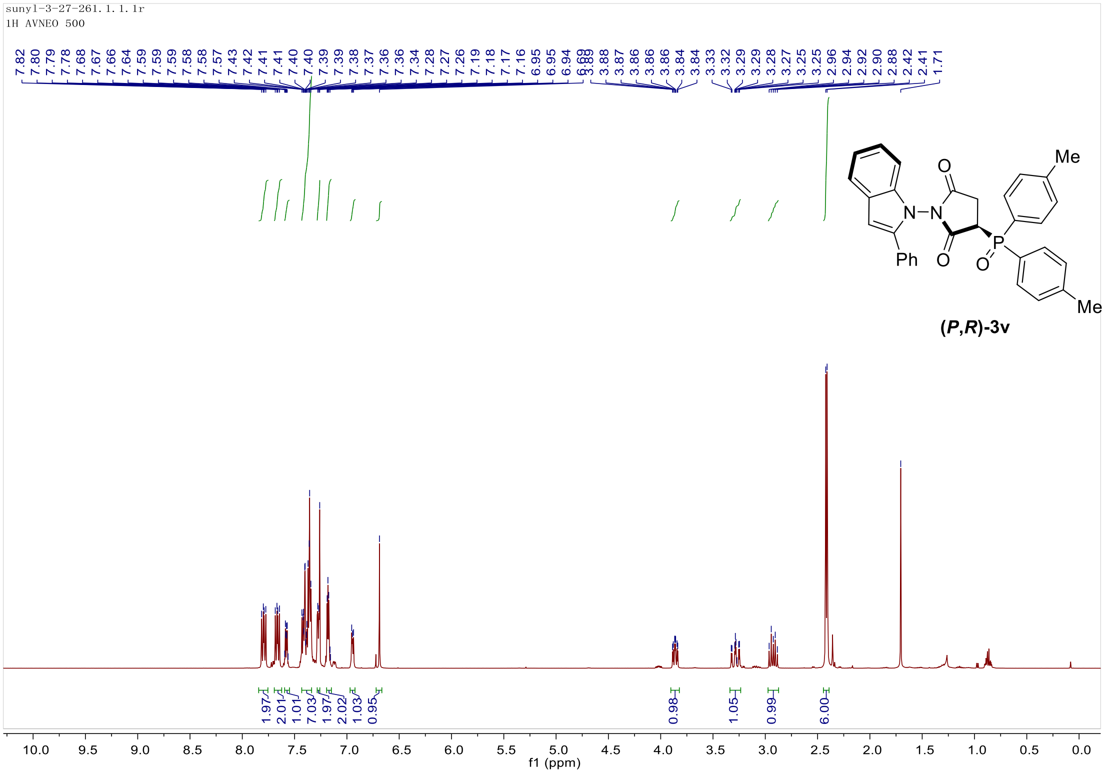

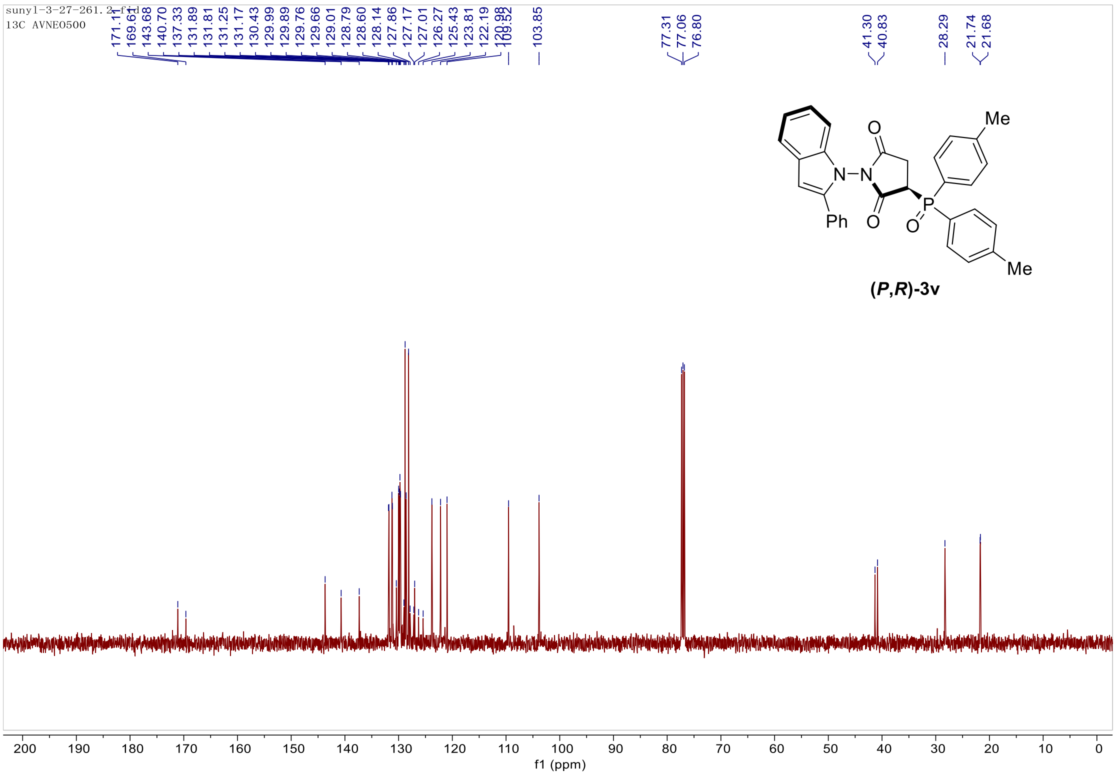

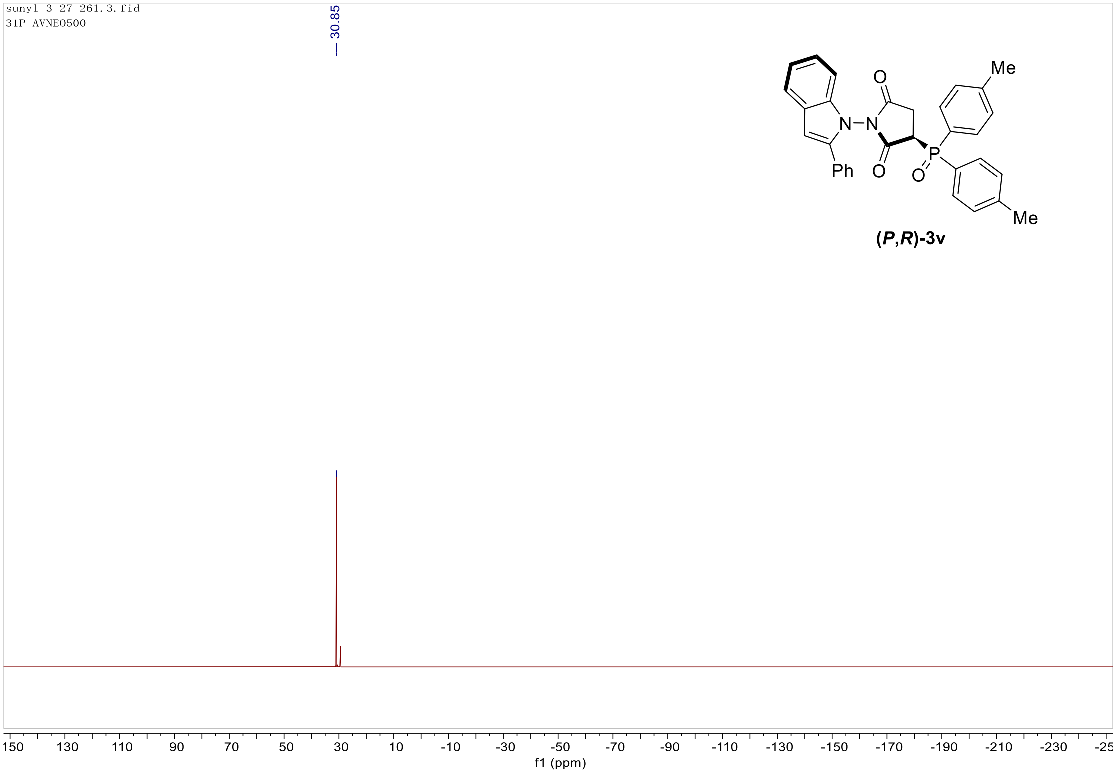
sunyl-3-27-261.3.fid
31P AVNEO500
30.85
Me
N
N
O
O
P
O
Ph
Me
(P,R)-3v
f1 (ppm)

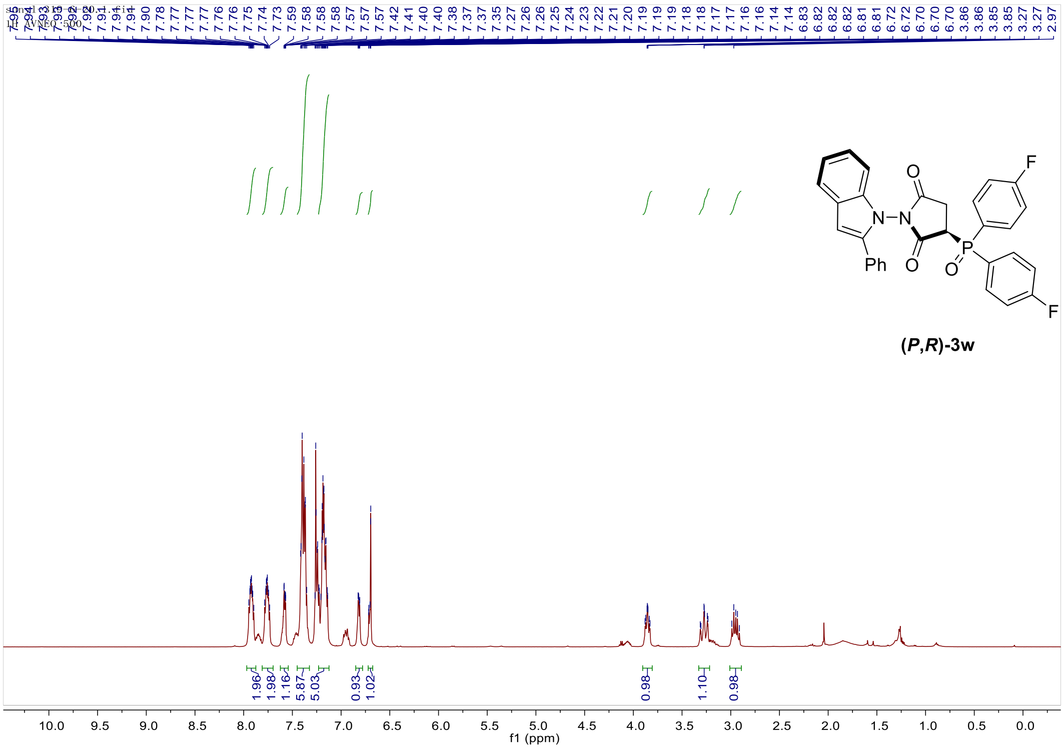
F
N
N
O
O
P
O
Ph
F
(P,R)-3w
1.96
1.98
1.16
5.87
5.03
0.93
1.02
0.98
1.10
0.98
f1 (ppm)

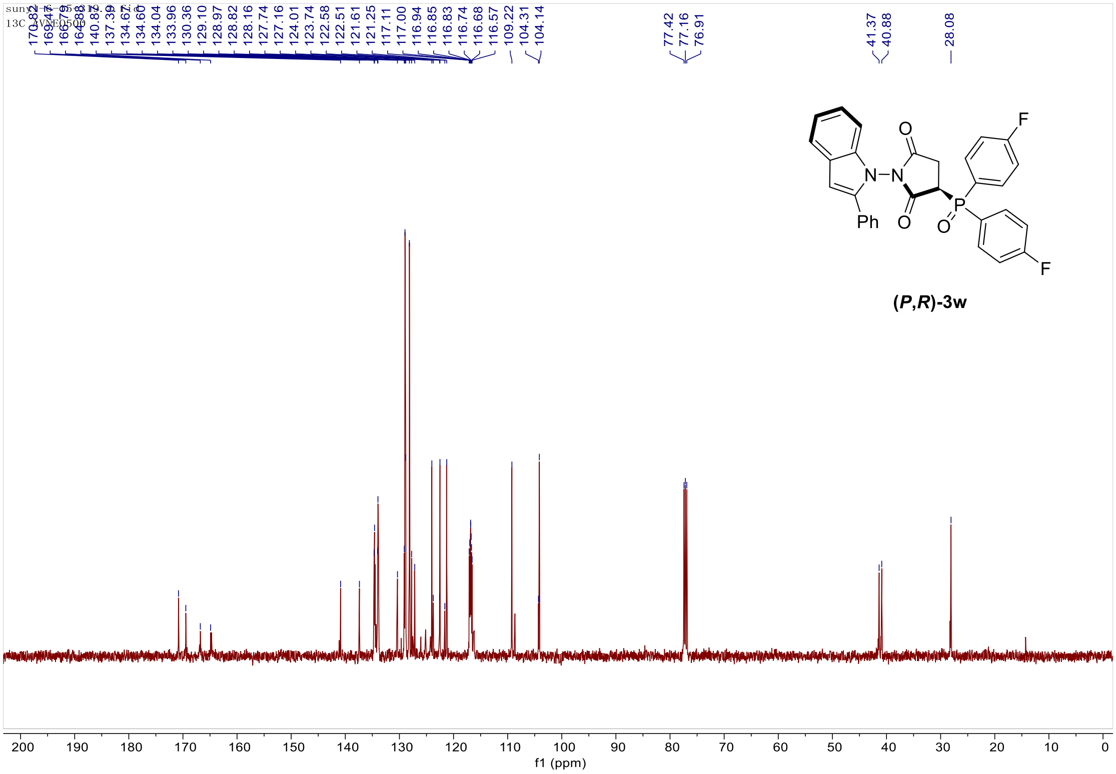
13C AVNEO500
170.82
169.47
166.79
164.83
140.87
137.39
134.67
134.60
134.04
133.96
130.36
129.10
128.97
128.82
128.16
127.74
127.16
124.01
123.74
122.58
122.51
121.61
121.25
117.11
117.00
116.94
116.85
116.83
116.74
116.68
116.57
109.22
104.31
104.14
77.42
77.16
76.91
41.37
40.88
28.08
Ph
F
F
(P,R)-3w
f1 (ppm)

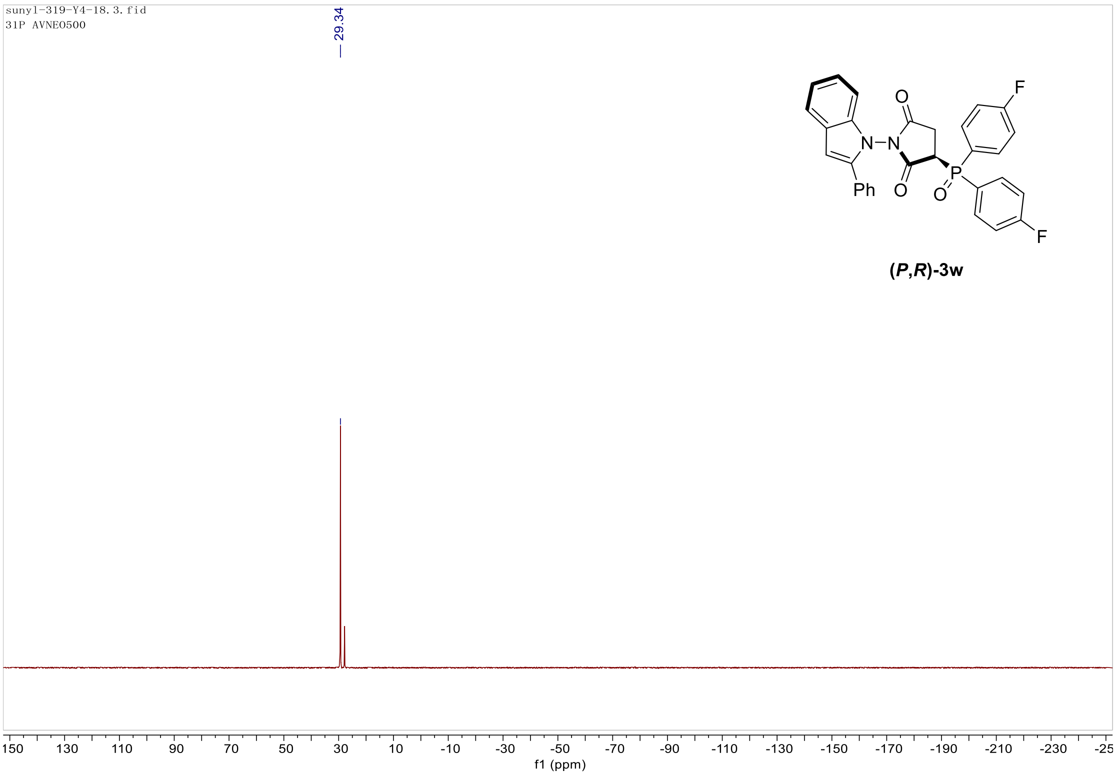
sunyl-319-Y4-18.3.fid
31P AVNEO500
29.34
Ph
F
F
(P,R)-3w
f1 (ppm)

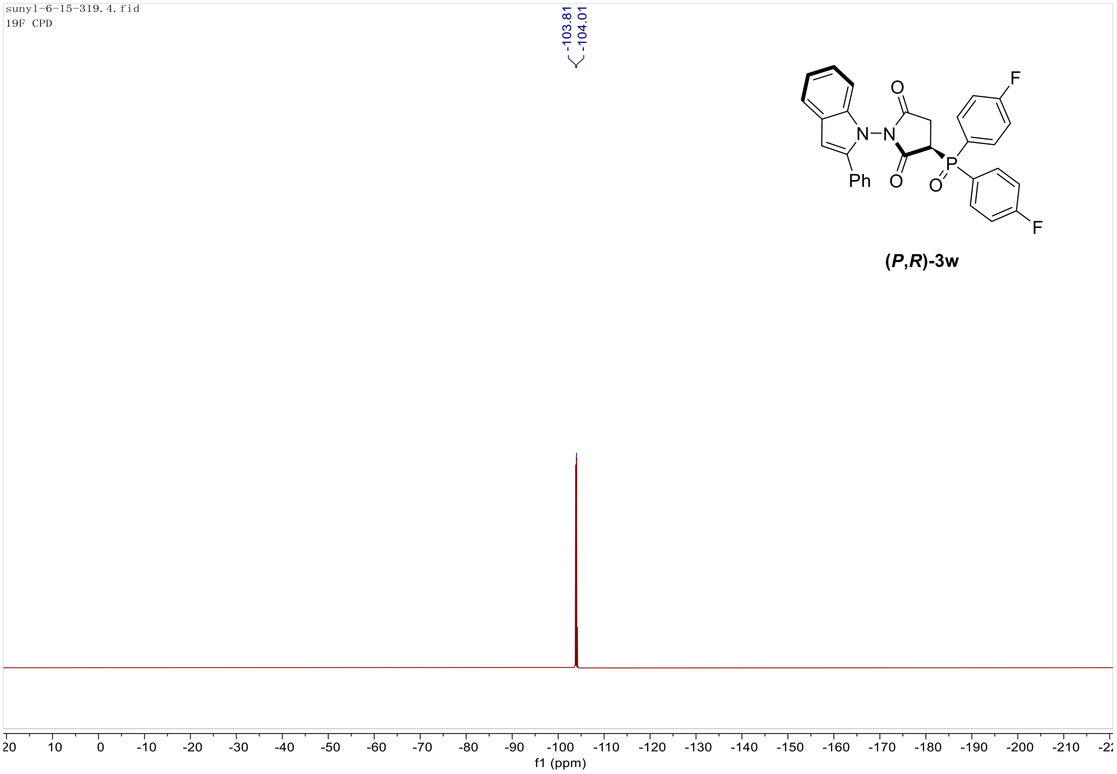
sunyl-6-15-319.4.fid
19F CPD
-103.81
-104.01
(P,R)-3w
f1 (ppm)

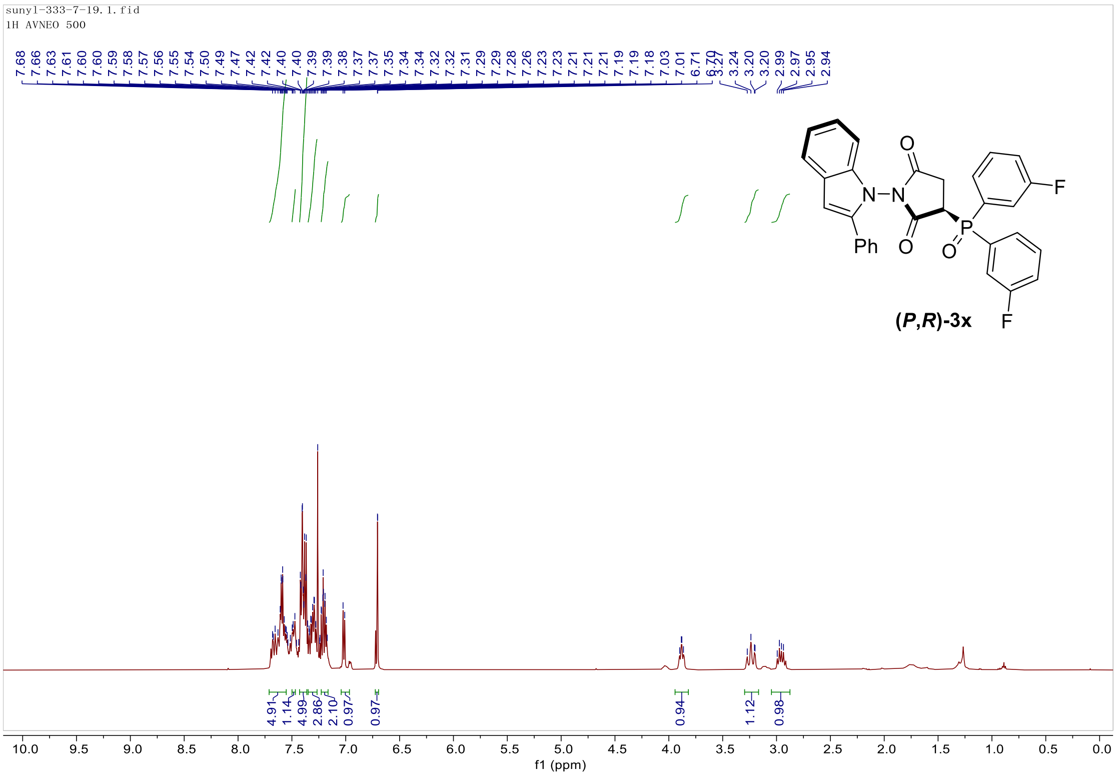
sunyl-333-7-19.1.fid
1H AVNEO 500
4.91
1.14
4.99
2.86
2.10
0.97
0.97
0.94
1.12
0.98
(P,R)-3x
f1 (ppm)

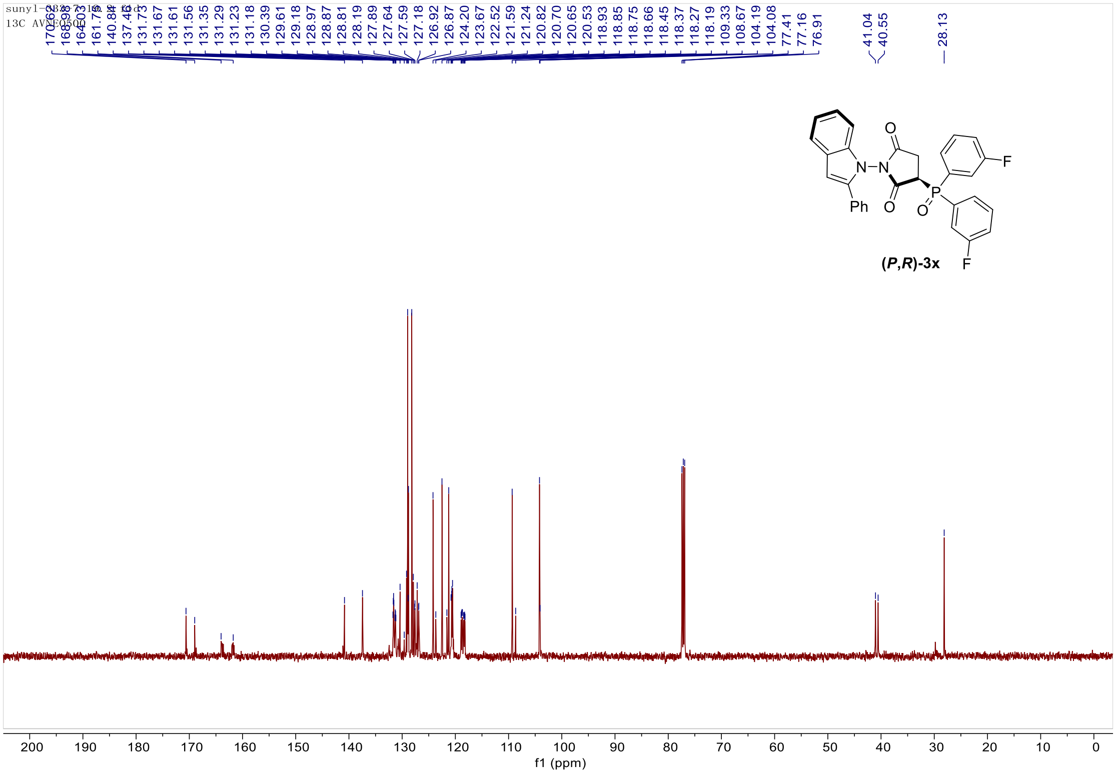
13C
170.62 166.98 164.03 161.79 140.84 137.46 131.73 131.67 131.61 131.56 131.35 131.29 131.23 131.18 130.39 129.61 129.18 128.97 128.87 128.81 128.19 127.89 127.64 127.59 127.18 126.92 126.87 124.20 123.67 122.52 121.59 121.24 120.82 120.70 120.65 120.53 118.93 118.85 118.75 118.66 118.45 118.37 118.27 118.19 109.33 108.67 104.19 104.08 77.41 77.16 76.91
41.04
40.55
28.13
(P,R)-3x
f1 (ppm)

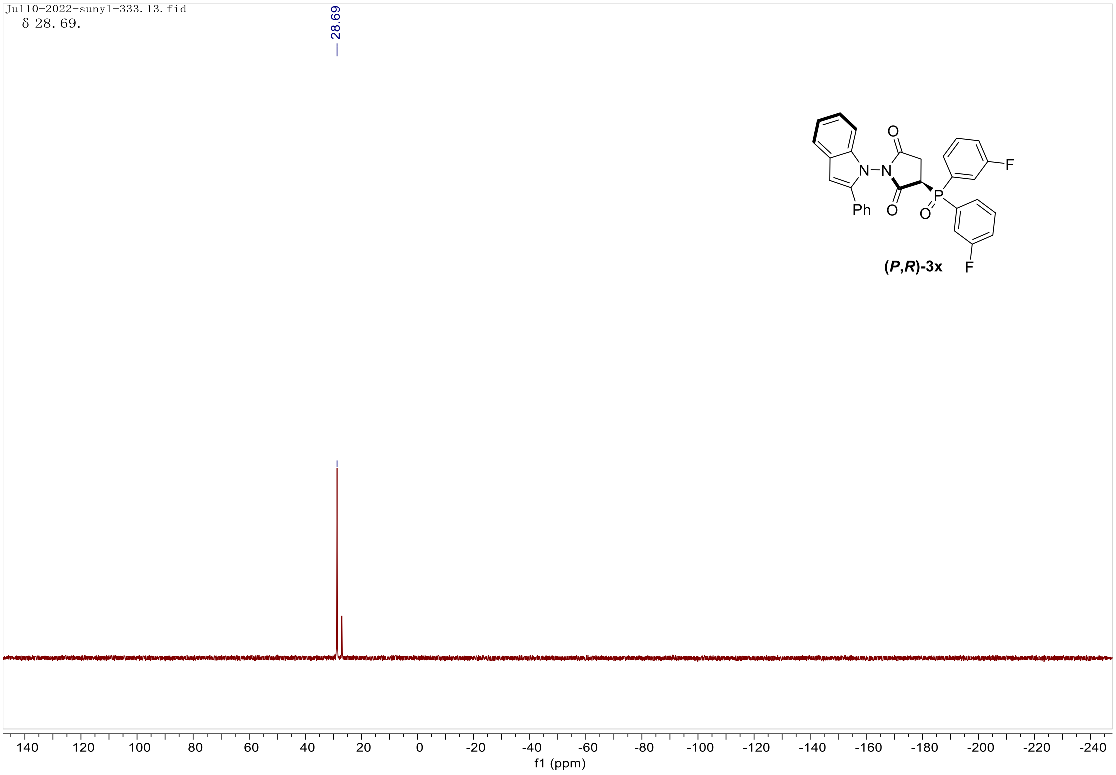
Jul10-2022-sunyl-333.13.fid
δ 28.69.
28.69
(P,R)-3x
f1 (ppm)

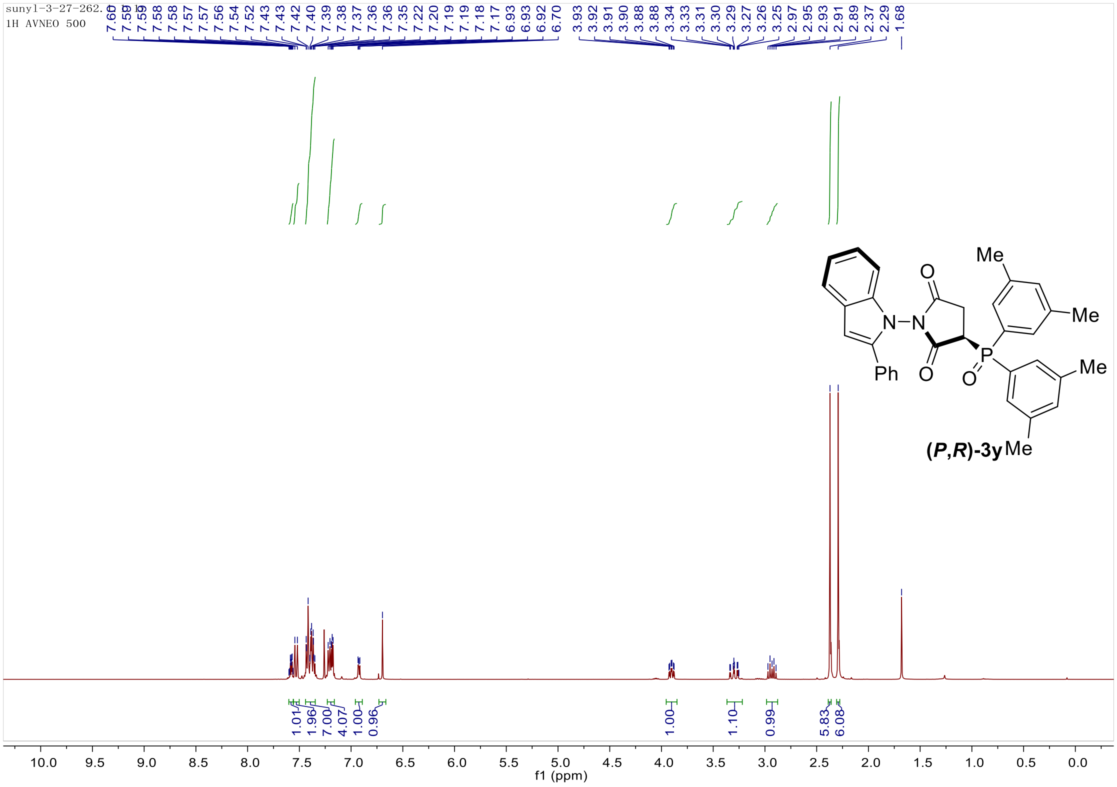
sunyl-3-27-262.
1H AVNEO 500
7.60 7.59 7.59 7.58 7.57 7.56 7.54 7.52 7.43 7.42 7.40 7.39 7.38 7.37 7.36 7.35 7.22 7.20 7.19 7.19 7.18 7.17 6.93 6.93 6.92 6.70 3.93 3.92 3.91 3.90 3.88 3.88 3.34 3.33 3.31 3.30 3.29 3.27 3.26 3.25 2.97 2.95 2.93 2.91 2.89 2.37 2.29 1.68
1.01 1.96 7.00 4.07 1.00 0.96 1.00 1.10 0.99 5.83 6.08
f1 (ppm)
Me
Me
Me
Me
Ph
O
O
O
N
N
P
(P,R)-3y

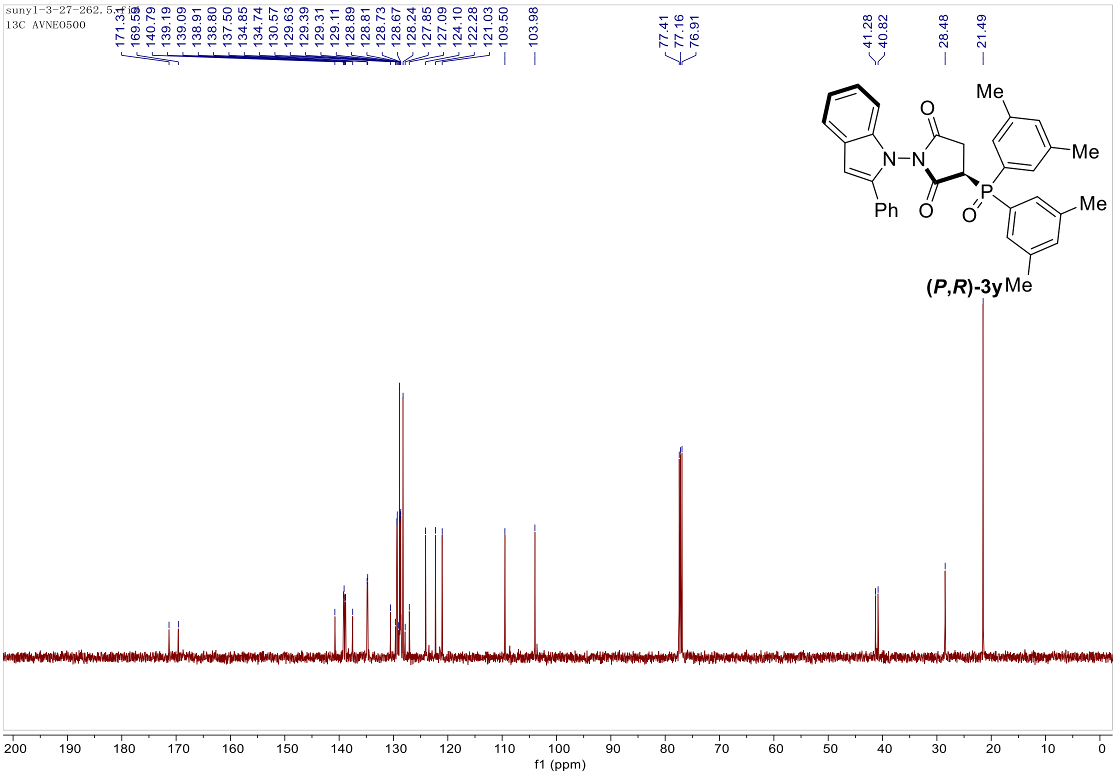
sunyl-3-27-262.
13C AVNEO500
171.34 169.59 140.79 139.19 139.09 138.91 138.80 137.50 134.85 134.74 130.57 129.63 129.39 129.31 129.11 128.89 128.81 128.73 128.67 128.24 127.85 127.09 124.10 122.28 121.03 109.50 103.98 77.41 77.16 76.91 41.28 40.82 28.48 21.49
f1 (ppm)
Me
Me
Me
Me
Ph
O
O
O
N
N
P
(P,R)-3y

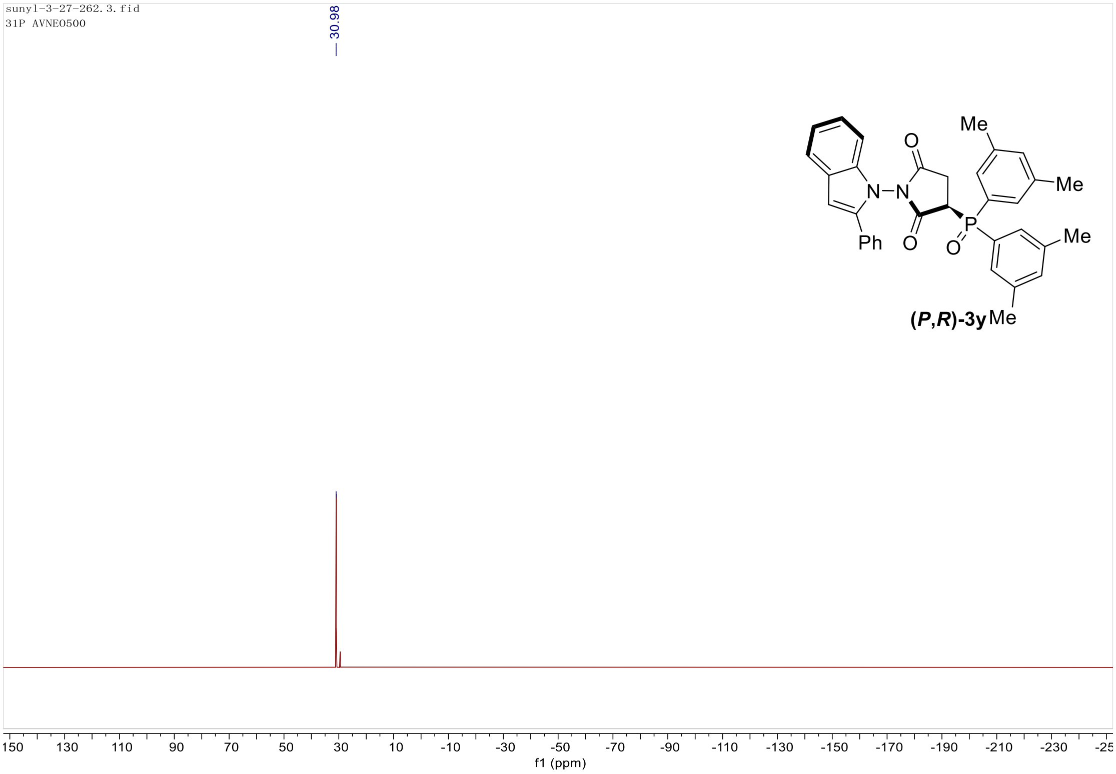
sunyl-3-27-262.3.fid
31P AVNEO500
30.98
Me
O
N–N
Me
Me
Ph
O
P
O
Me
(P,R)-3y
f1 (ppm)

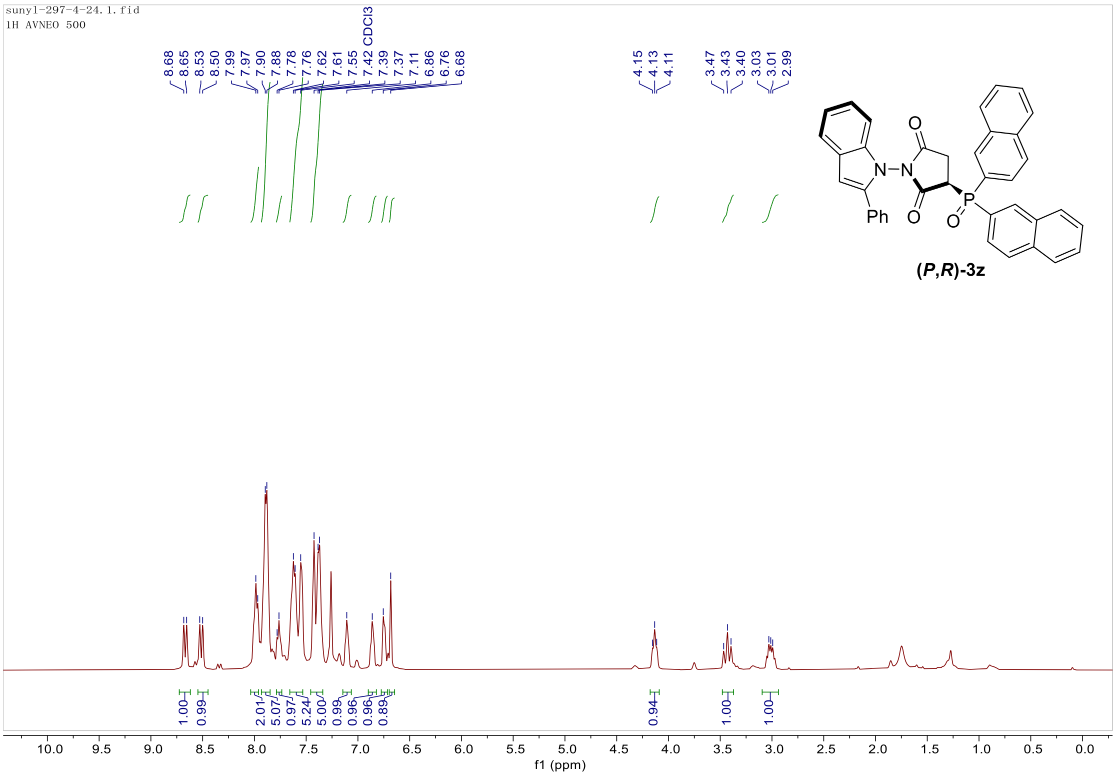
sunyl-297-4-24.1.fid
1H AVNEO 500
CDCl3
8.68
8.65
8.53
8.50
7.99
7.97
7.90
7.88
7.78
7.76
7.62
7.61
7.55
7.42
7.39
7.37
7.11
6.86
6.76
6.68
4.15
4.13
4.11
3.47
3.43
3.40
3.03
3.01
2.99
O
N–N
Ph
O
P
O
(P,R)-3z
1.00
0.99
2.01
5.07
0.97
5.24
5.00
0.99
0.96
0.96
0.89
0.94
1.00
1.00
f1 (ppm)

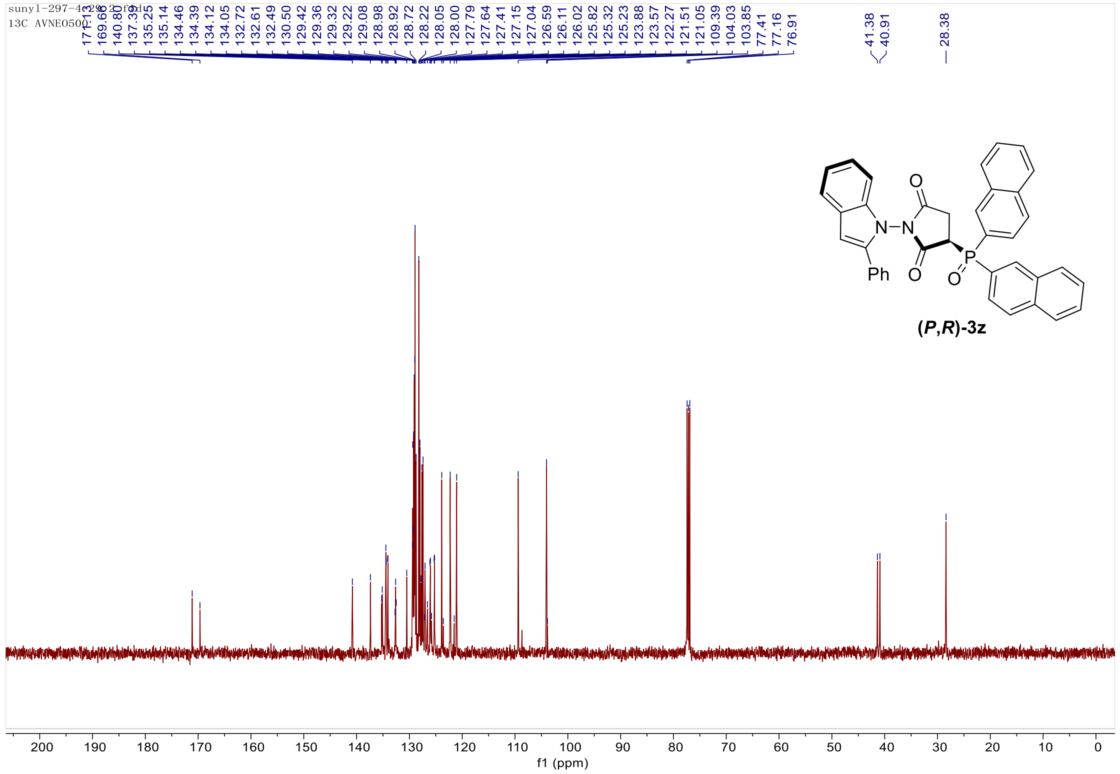
13C AVNEO500
171.13
169.66
140.80
137.39
135.25
135.14
134.46
134.39
134.12
134.05
132.72
132.61
132.49
130.50
129.42
129.36
129.32
129.22
129.08
128.98
128.92
128.72
128.22
128.05
128.00
127.79
127.64
127.41
127.15
127.04
126.59
126.11
126.02
125.82
125.32
125.23
123.88
123.57
122.27
121.51
121.05
109.39
104.03
103.85
77.41
77.16
76.91
41.38
40.91
28.38
O
N
N
P
O
O
Ph
(P,R)-3z
200 190 180 170 160 150 140 130 120 110 100 90 80 70 60 50 40 30 20 10 0
f1 (ppm)

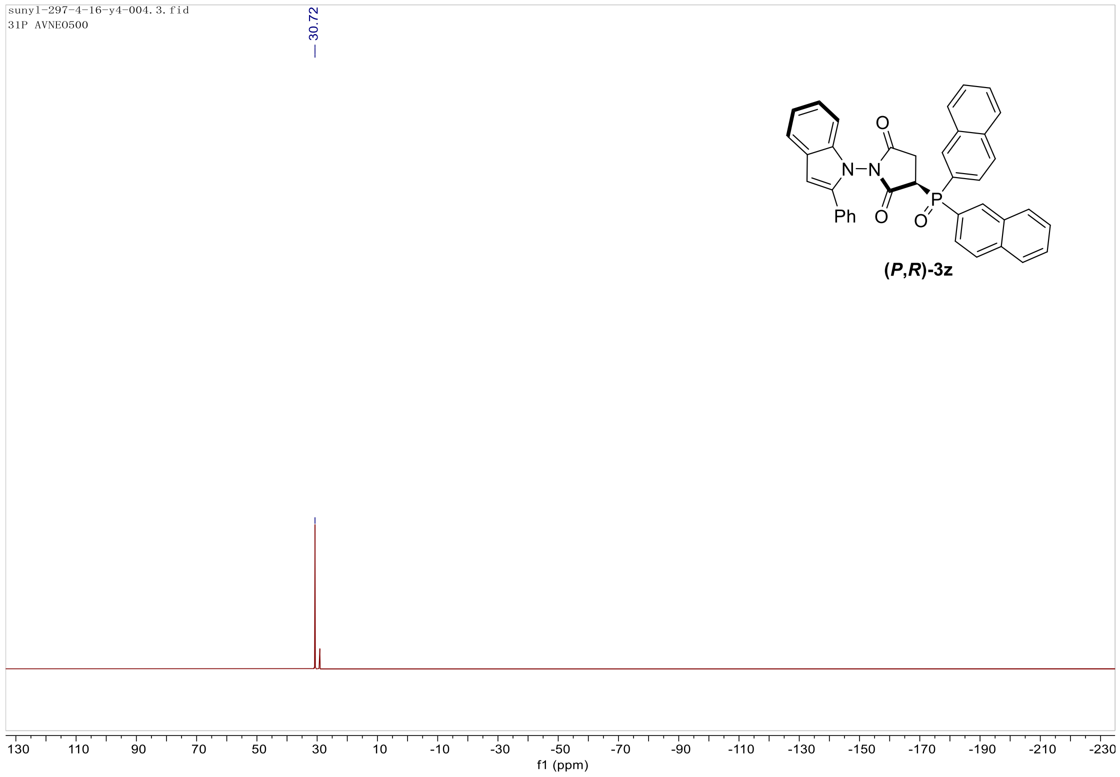
sunyl-297-4-16-y4-004.3.fid
31P AVNEO500
30.72
O
N
N
P
O
O
Ph
(P,R)-3z
130 110 90 70 50 30 10 -10 -30 -50 -70 -90 -110 -130 -150 -170 -190 -210 -230
f1 (ppm)

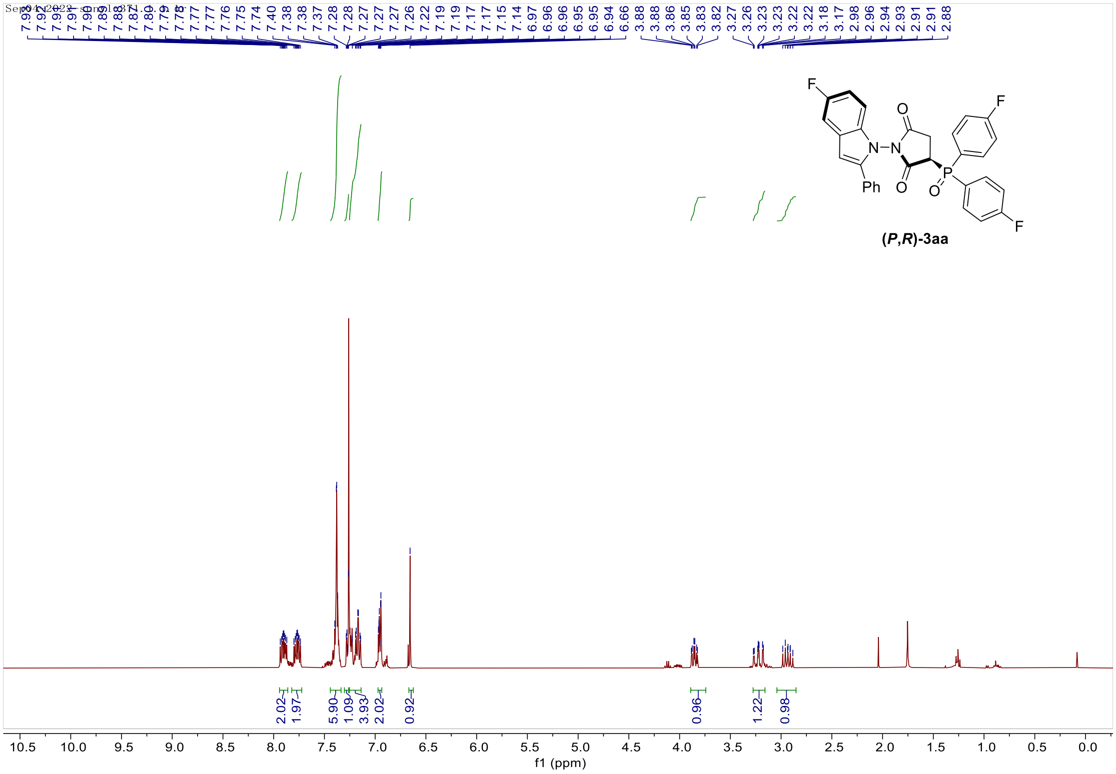
(P,R)-3aa
f1 (ppm)

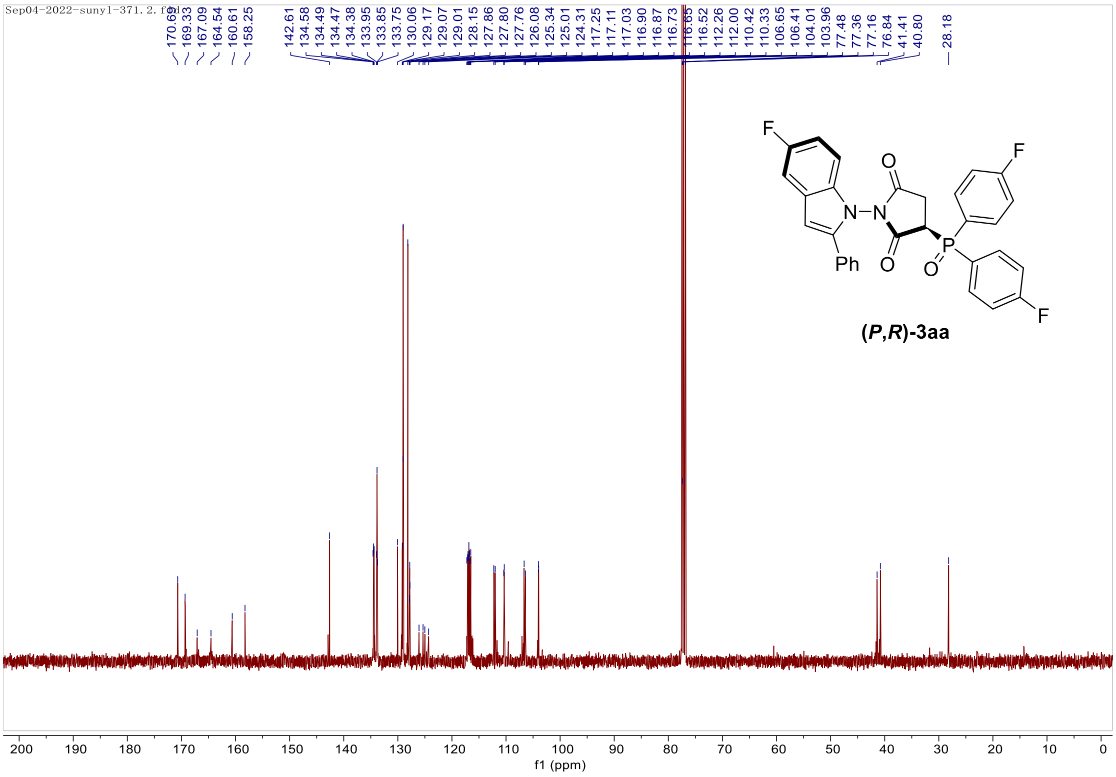
Sep04-2022-suny1-371.2.fid
(P,R)-3aa
f1 (ppm)

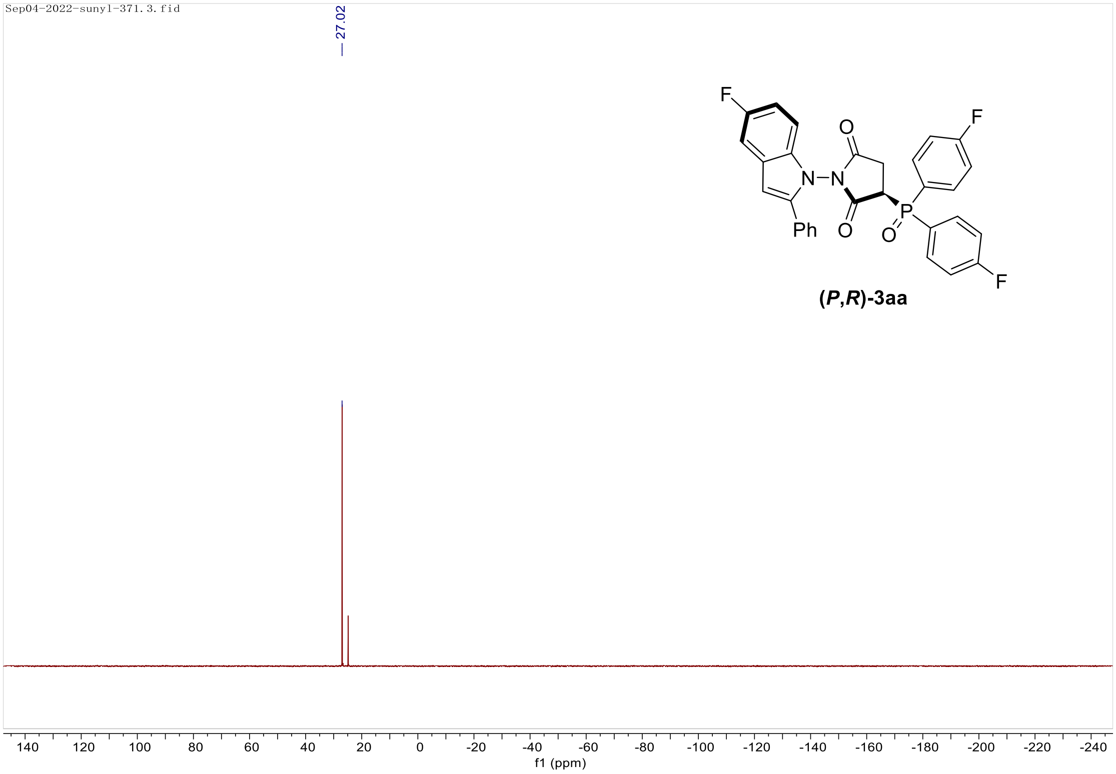

Sep04-2022-sunyl-371.3.fid
27.02
F
O
N–N
Ph
O
P
O
(P,R)-3aa
140
120
100
80
60
40
20
0
-20
-40
-60
-80
-100
-120
-140
-160
-180
-200
-220
-240
f1 (ppm)

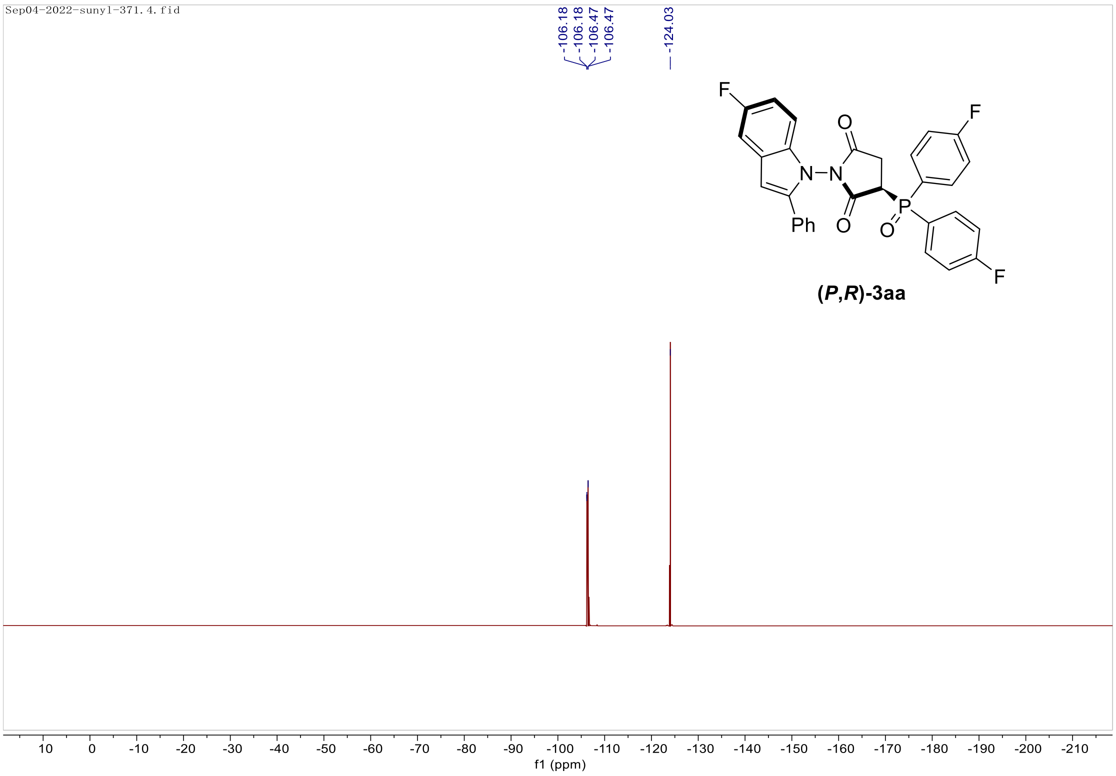

Sep04-2022-sunyl-371.4.fid
-106.18
-106.18
-106.47
-106.47
-124.03
F
O
N–N
Ph
O
P
O
(P,R)-3aa
10
0
-10
-20
-30
-40
-50
-60
-70
-80
-90
-100
-110
-120
-130
-140
-150
-160
-170
-180
-190
-200
-210
f1 (ppm)

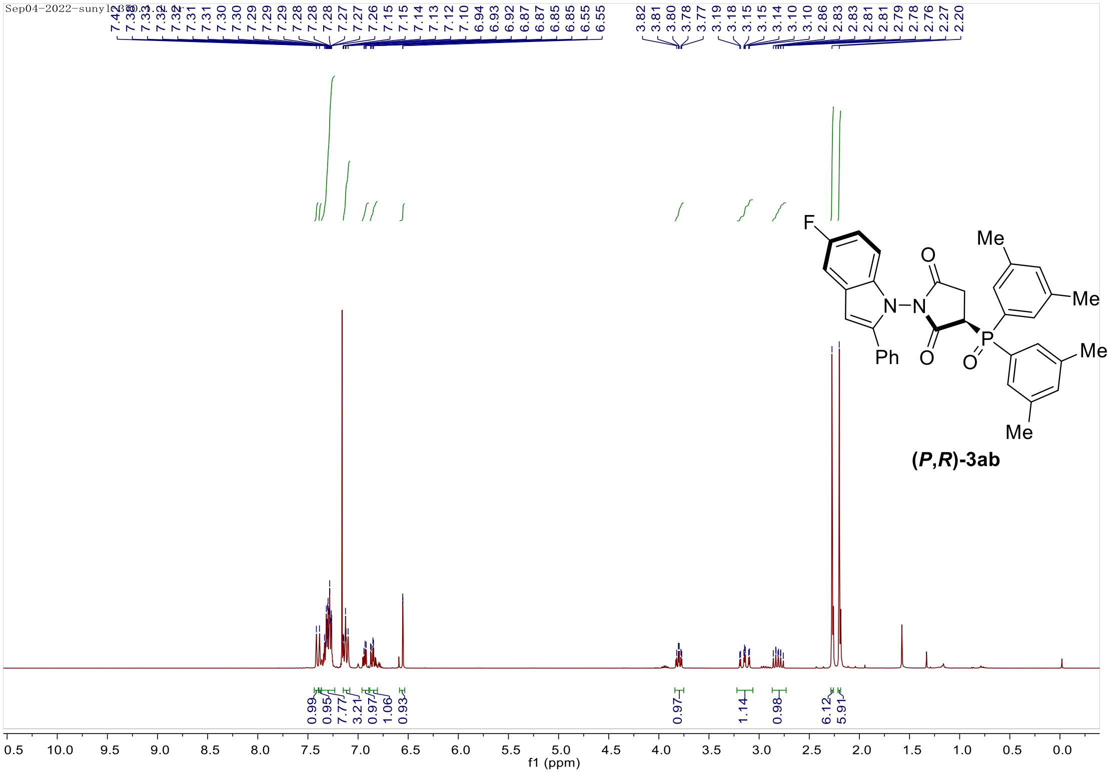

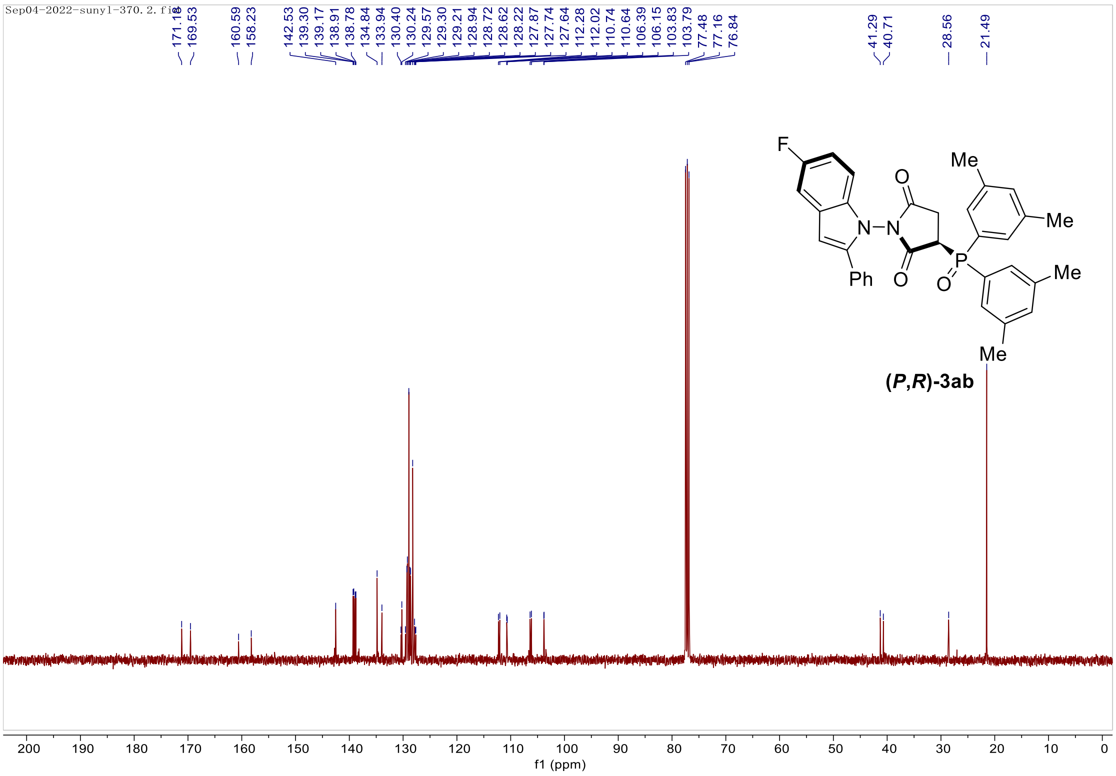

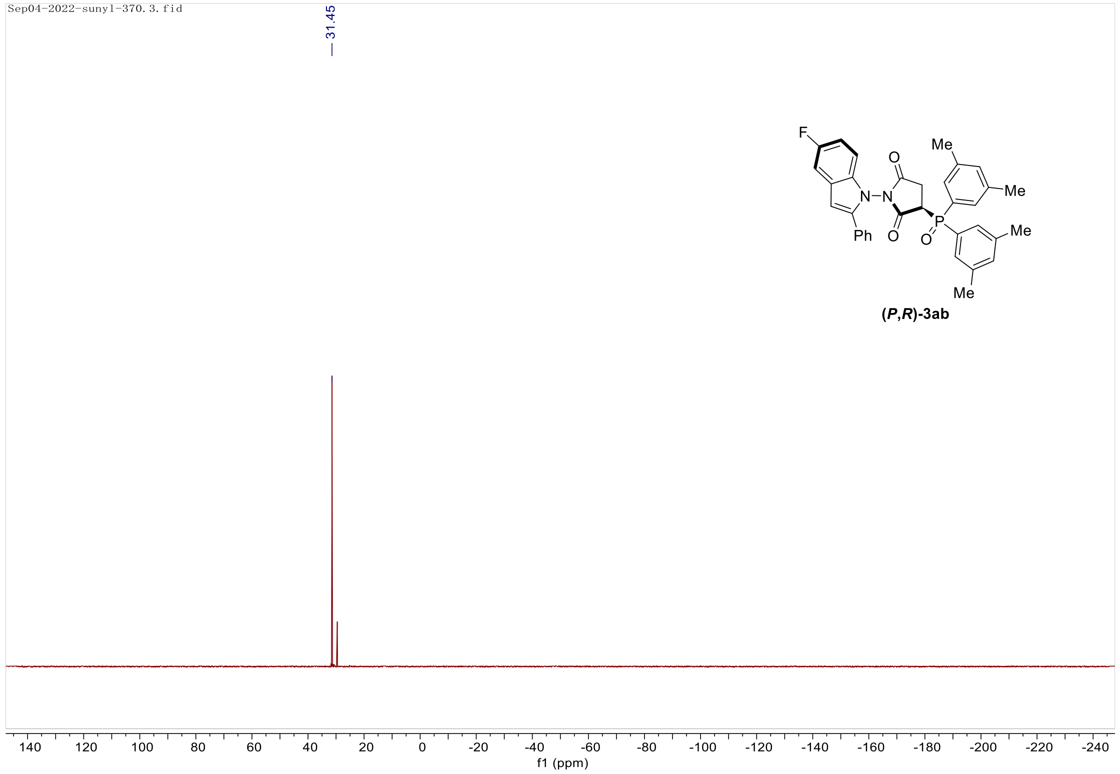
Sep04-2022-sunyl-370.3.fid
31.45
F
N
N
O
O
P
O
Me
Me
Me
Me
Ph
(P,R)-3ab
140 120 100 80 60 40 20 0 -20 -40 -60 -80 -100 -120 -140 -160 -180 -200 -220 -240
f1 (ppm)

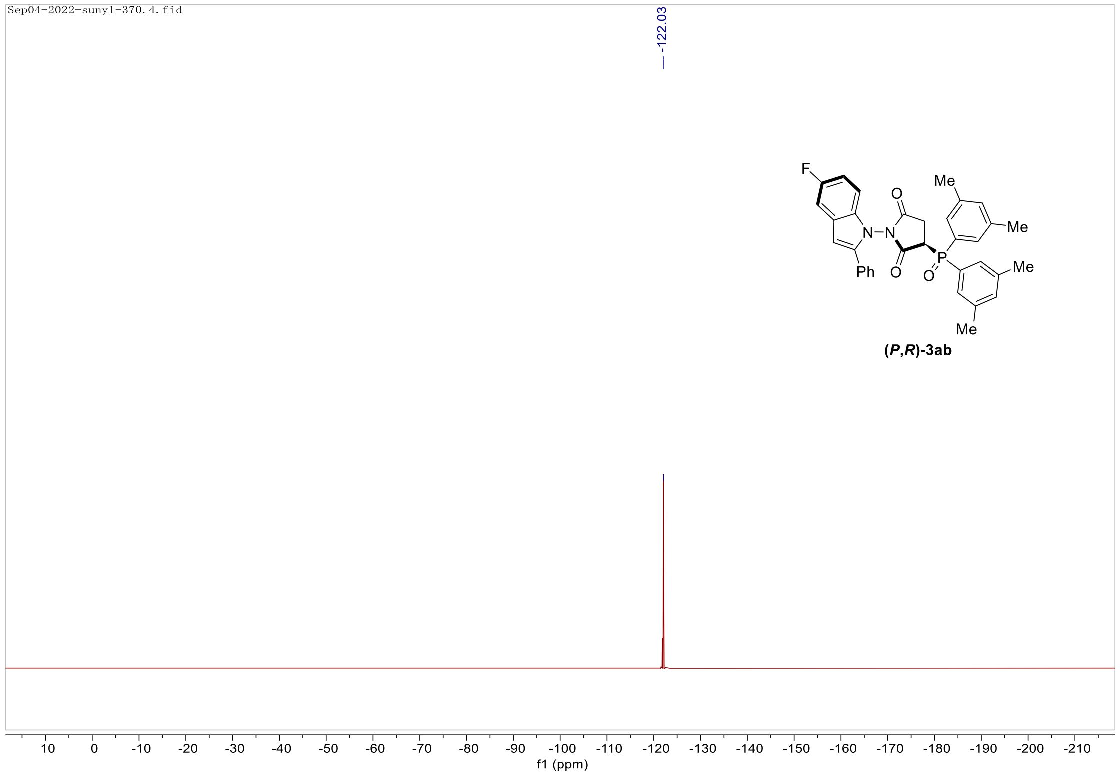
Sep04-2022-sunyl-370.4.fid
-122.03
F
N
N
O
O
P
O
Me
Me
Me
Me
Ph
(P,R)-3ab
10 0 -10 -20 -30 -40 -50 -60 -70 -80 -90 -100 -110 -120 -130 -140 -150 -160 -170 -180 -190 -200 -210
f1 (ppm)

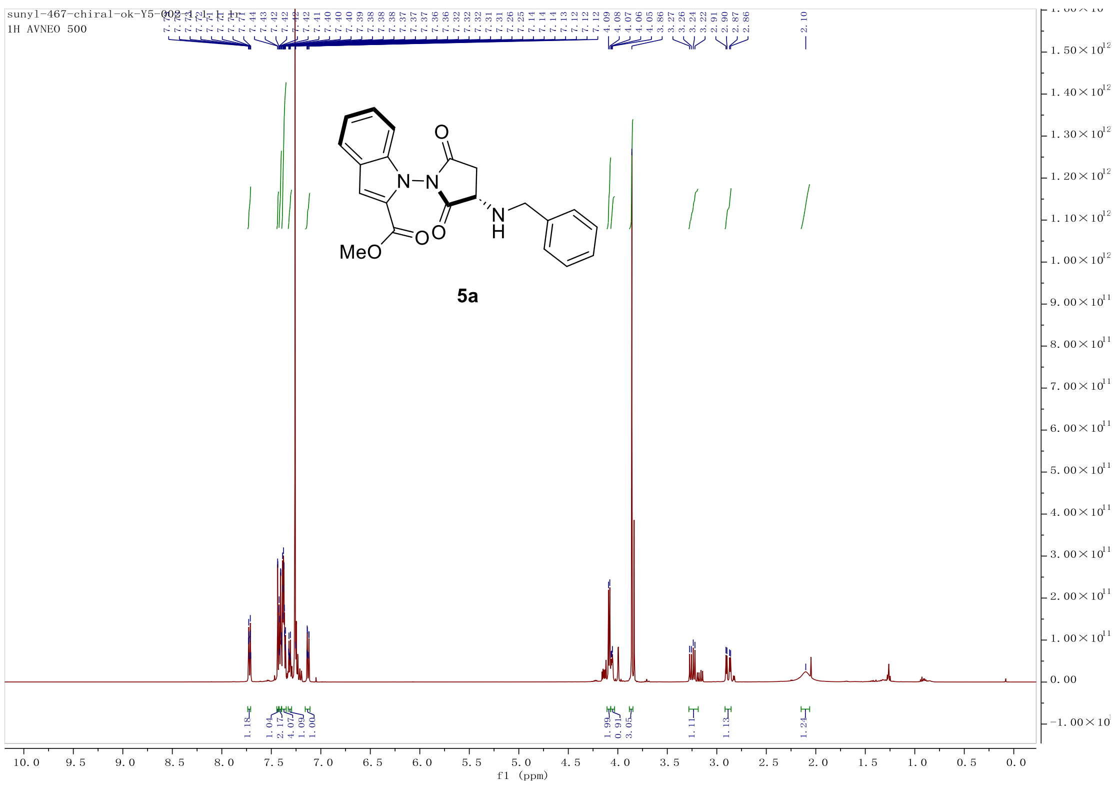
sunyl-467-chiral-ok-Y5-002
1H AVNEO 500
5a
MeO
f1 (ppm)

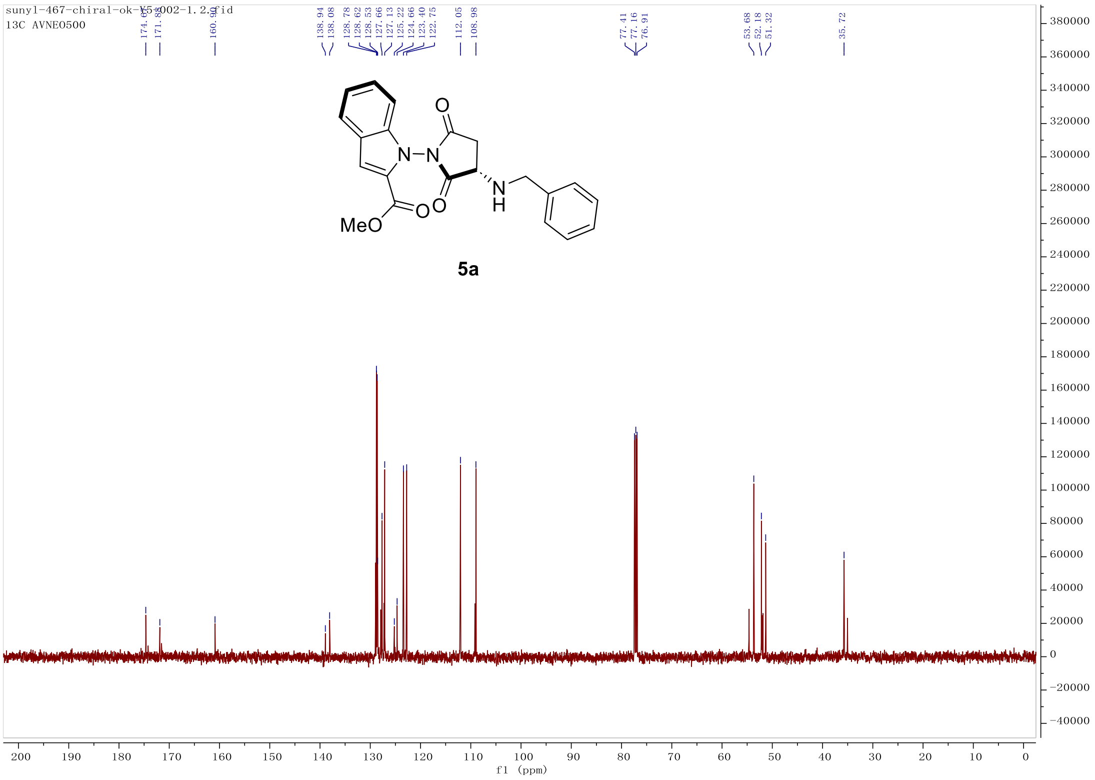
sunyl-467-chiral-ok-Y5-002-1.2.fid
13C AVNEO500
5a
MeO
f1 (ppm)

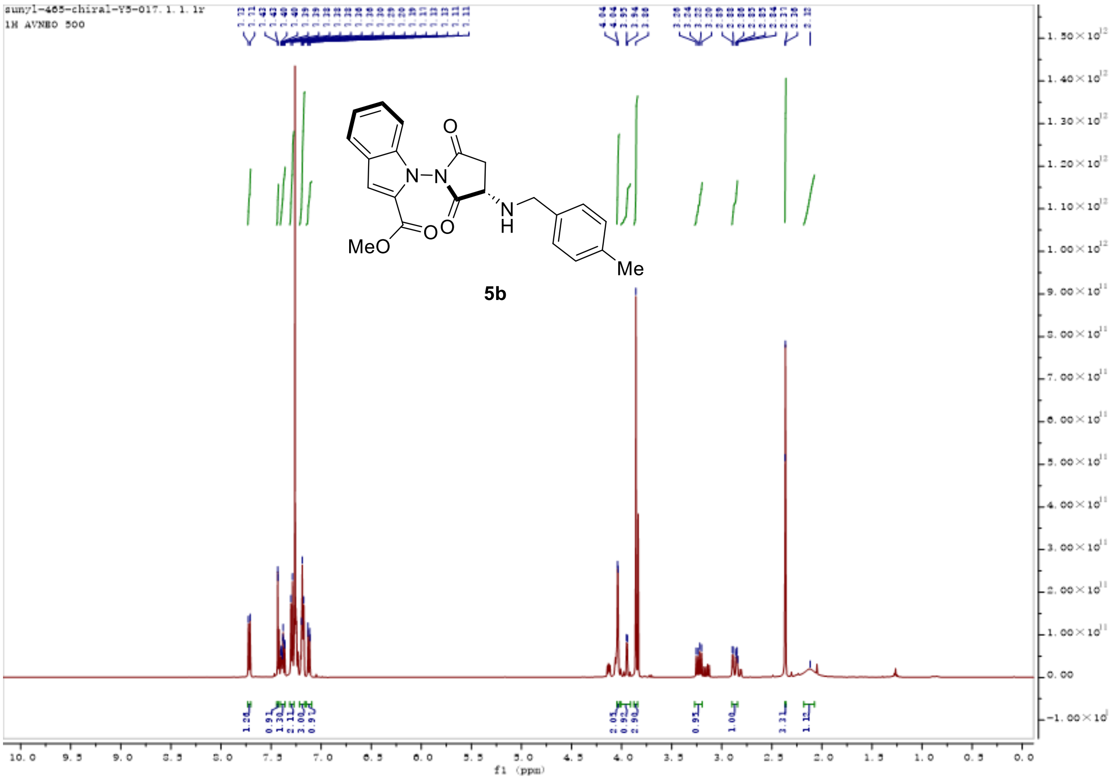

5b

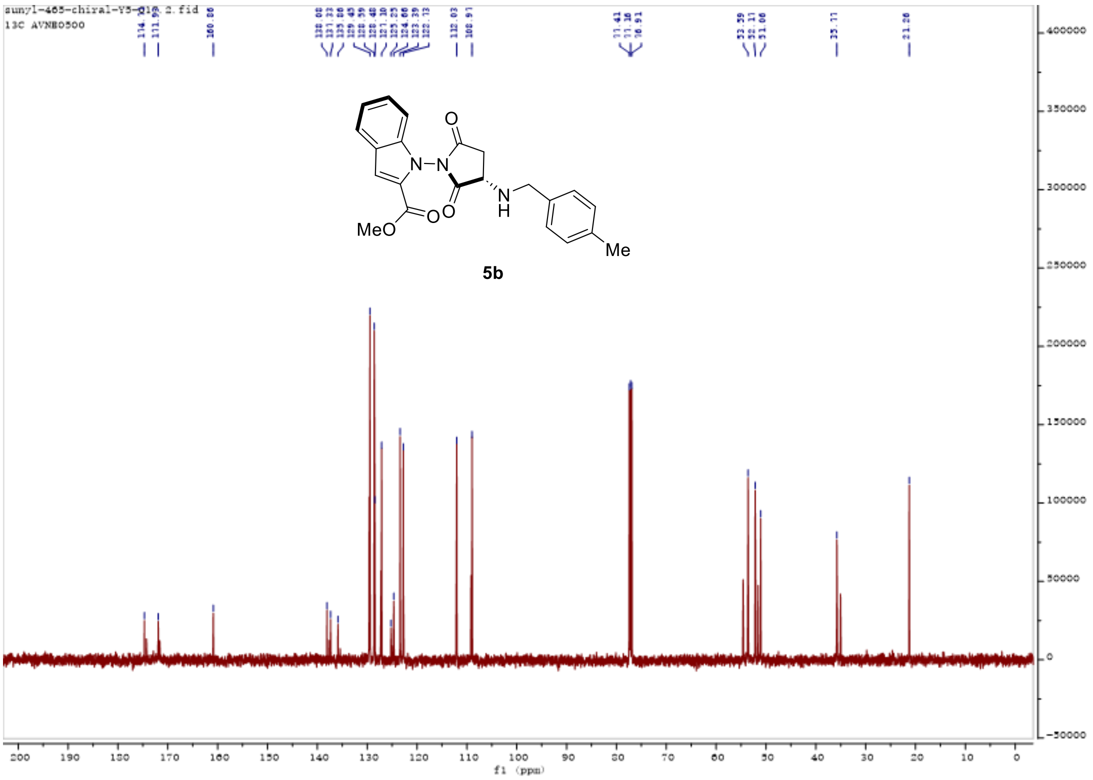

5b

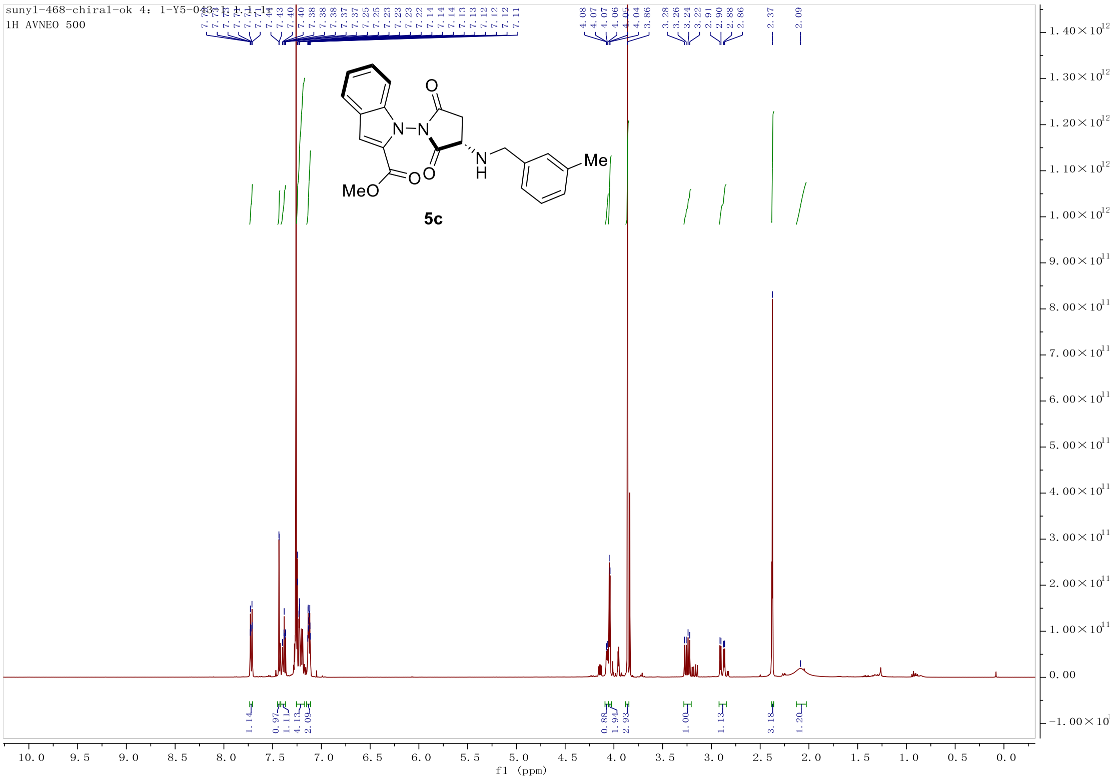
sunyl-468-chiral-ok 4: 1-Y5-043
1H AVNEO 500
5c
MeO
Me
f1 (ppm)

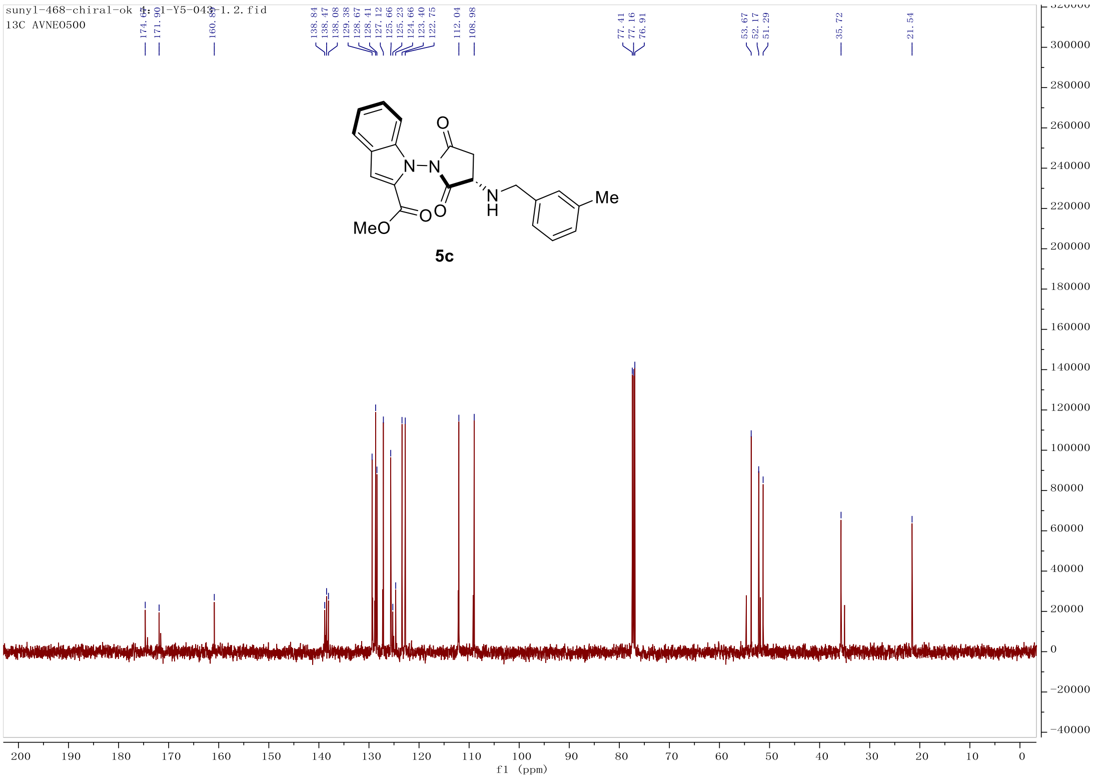
sunyl-468-chiral-ok 4: 1-Y5-043-1.2.fid
13C AVNEO500
5c
MeO
Me
f1 (ppm)

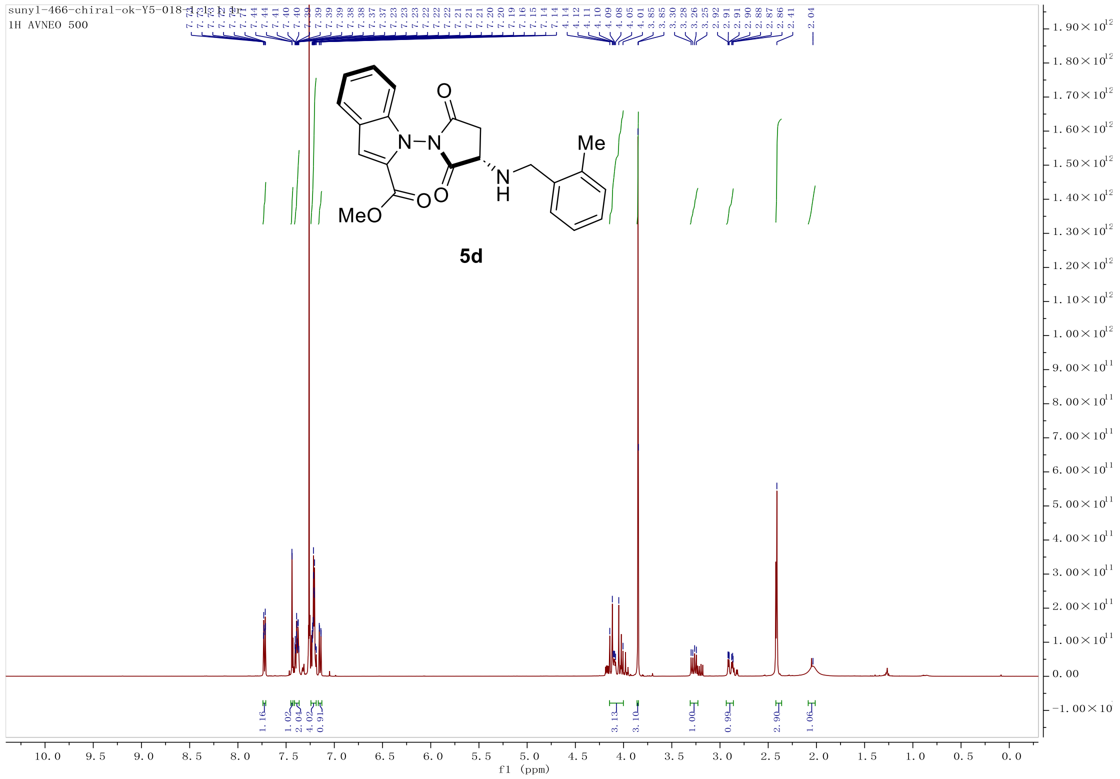
1H AVNEO 500
MeO
Me
5d
f1 (ppm)

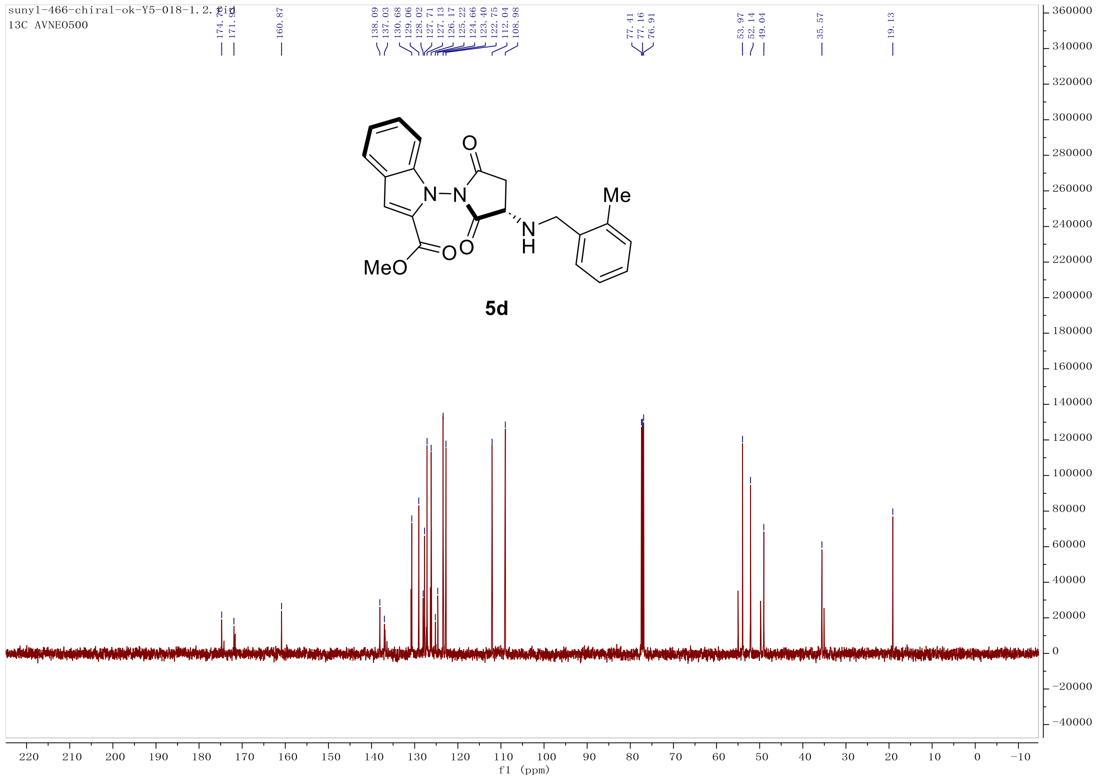
13C AVNEO500
160.87
138.09
137.03
130.68
129.06
128.02
127.71
127.13
126.17
125.22
124.66
123.40
122.75
112.04
108.98
77.41
77.16
76.91
53.97
52.14
49.04
35.57
19.13
MeO
Me
5d
f1 (ppm)

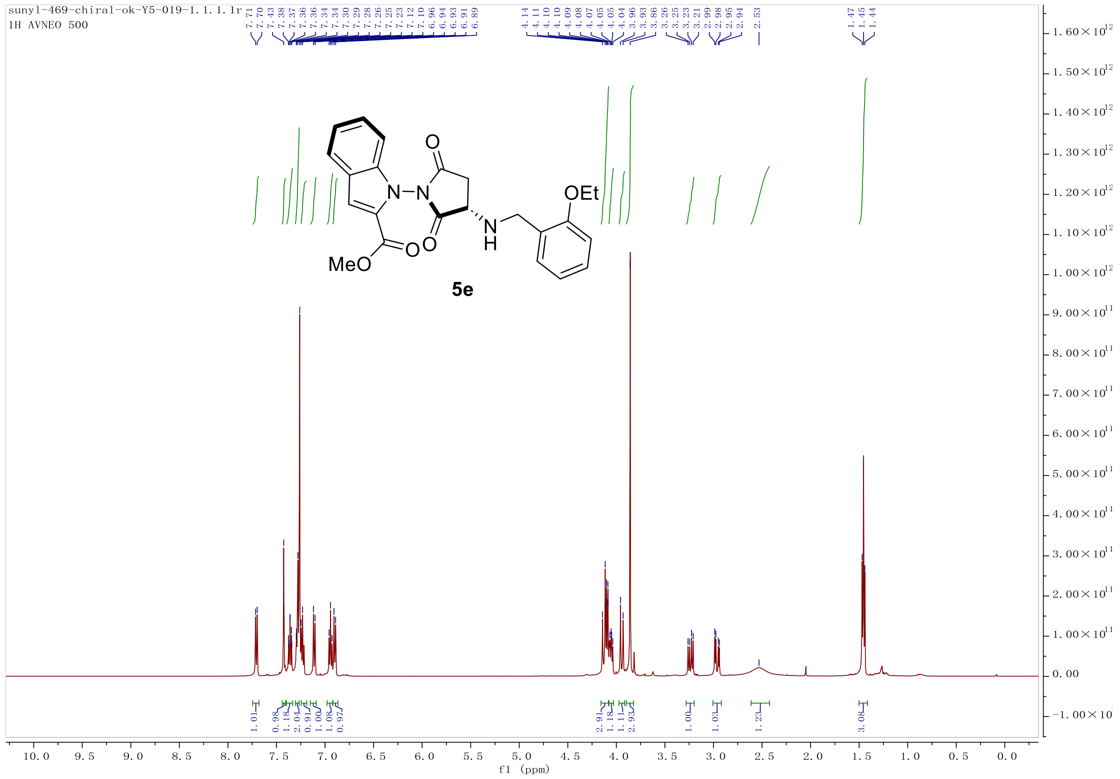

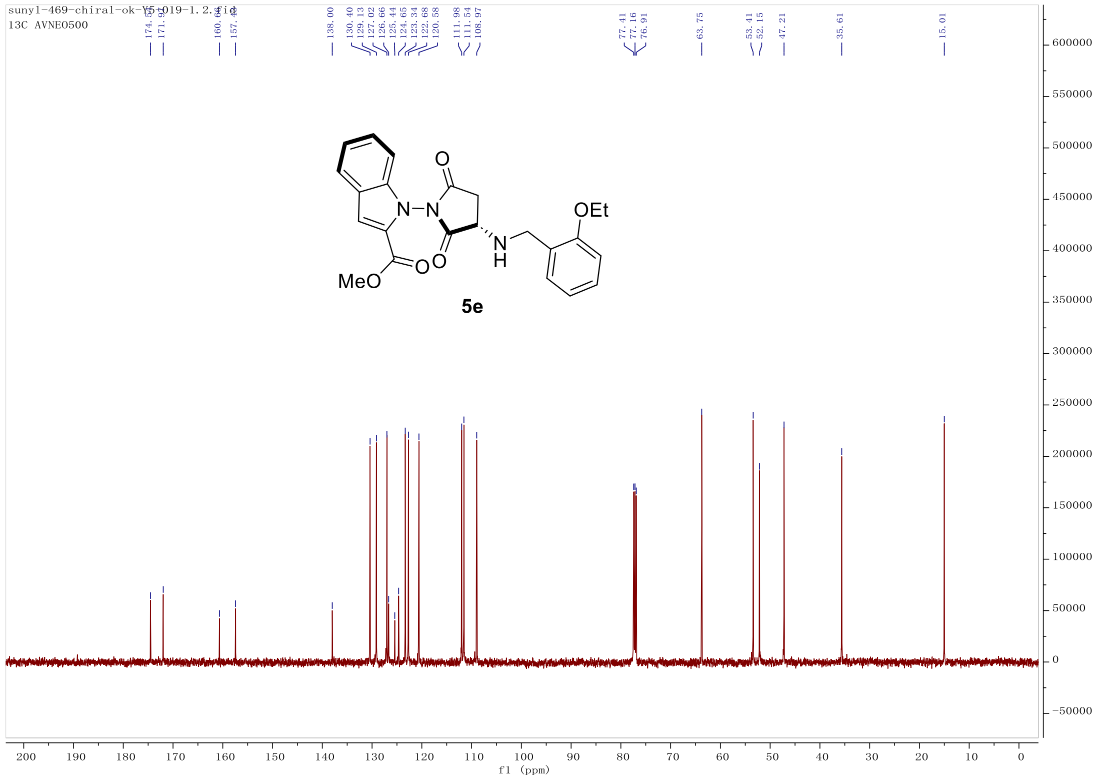

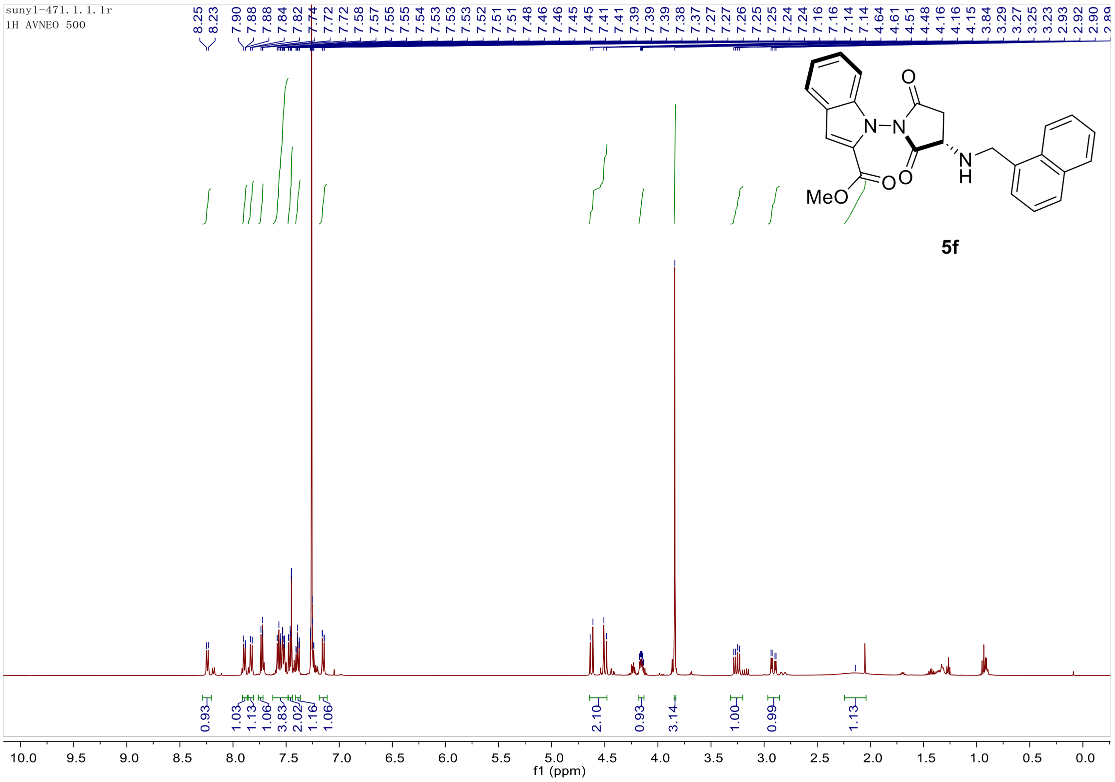
sunyl-471.1.1.1r
1H AVNEO 500
MeO
O
N
N
O
O
N
H
5f
f1 (ppm)

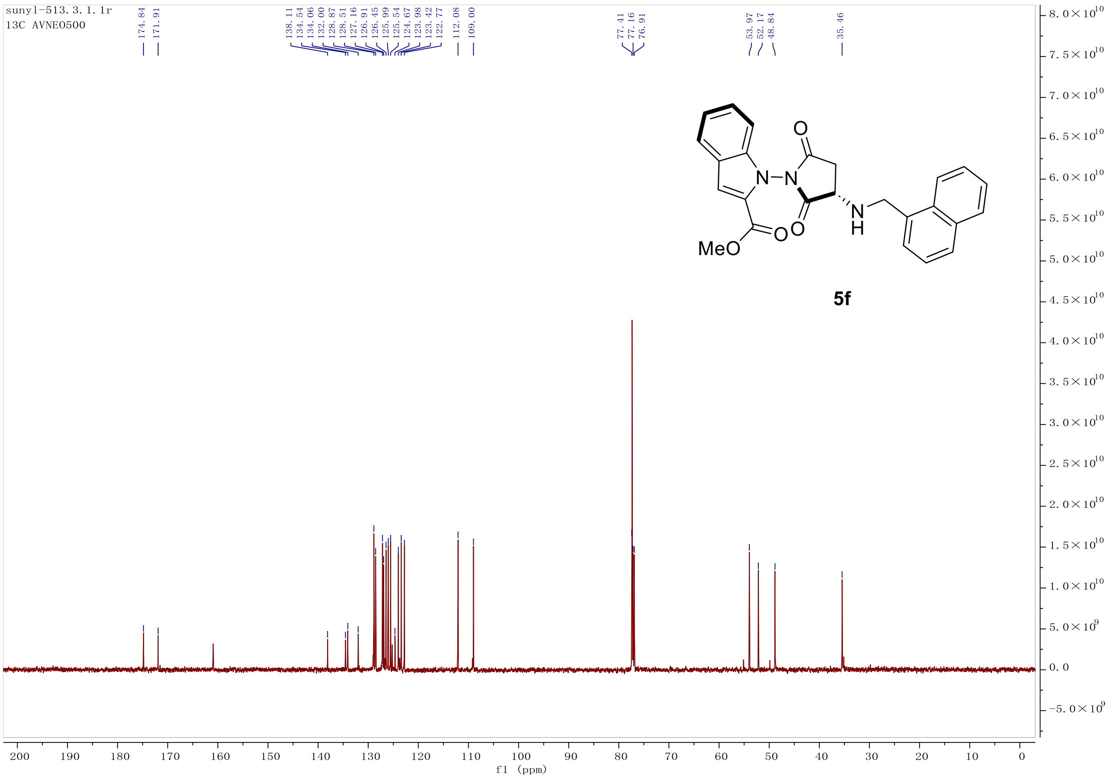
sunyl-513.3.1.1r
13C AVNEO500
174.84
171.91
77.41
77.16
76.91
53.97
52.17
48.84
35.46
MeO
5f
f1 (ppm)

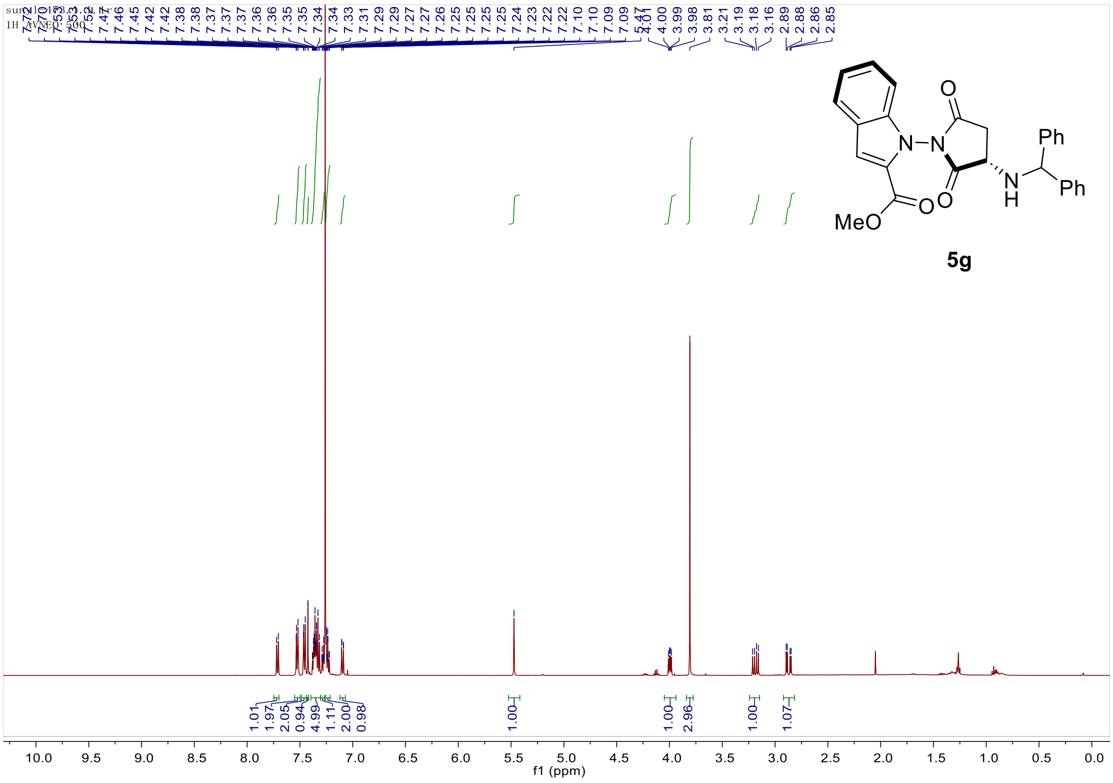

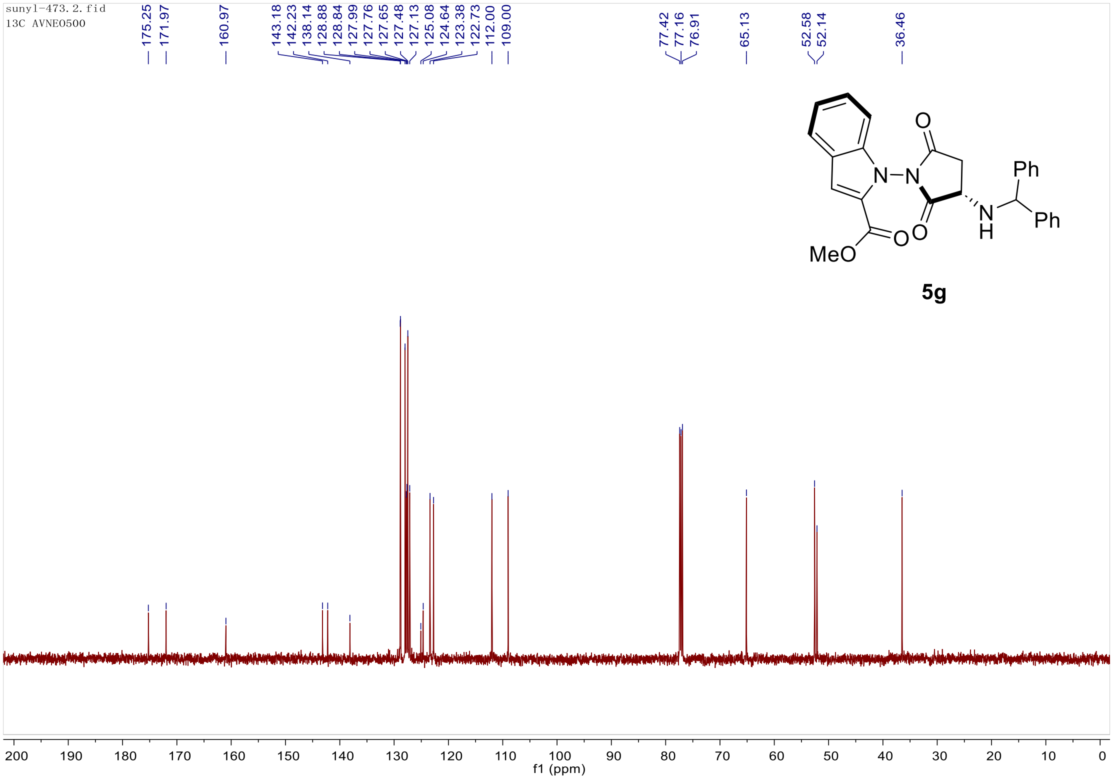

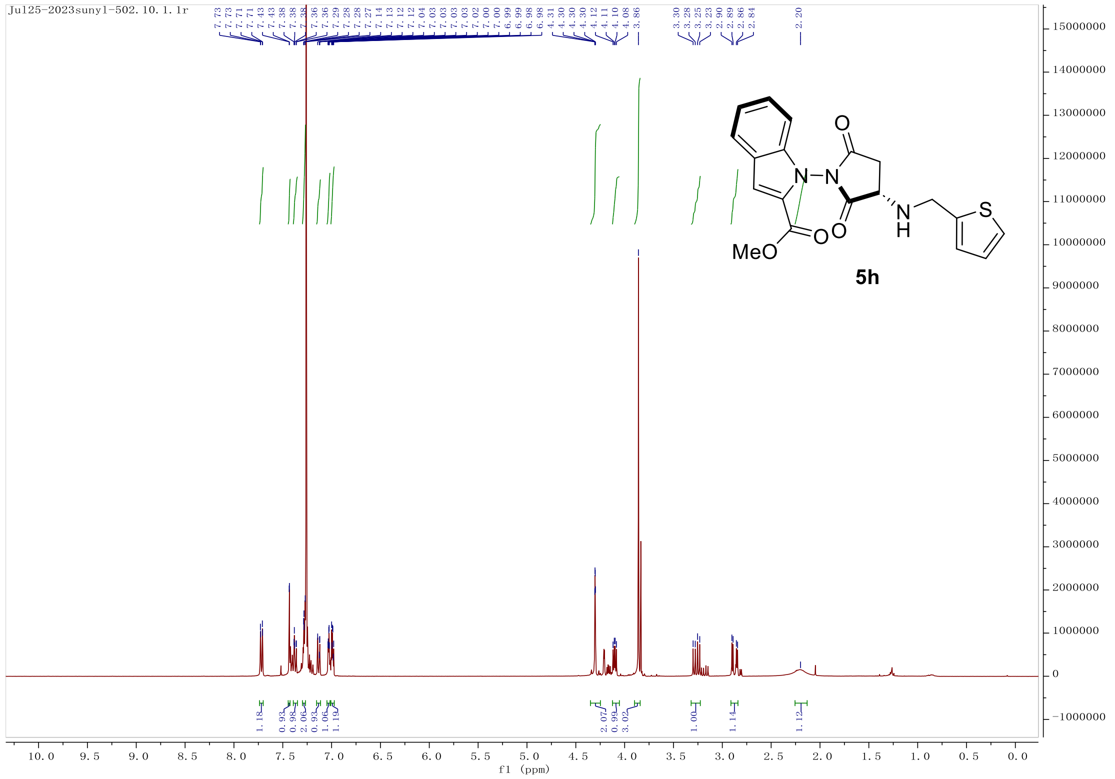
Jul25-2023sunyl-502.10.1.1r
MeO
5h
f1 (ppm)

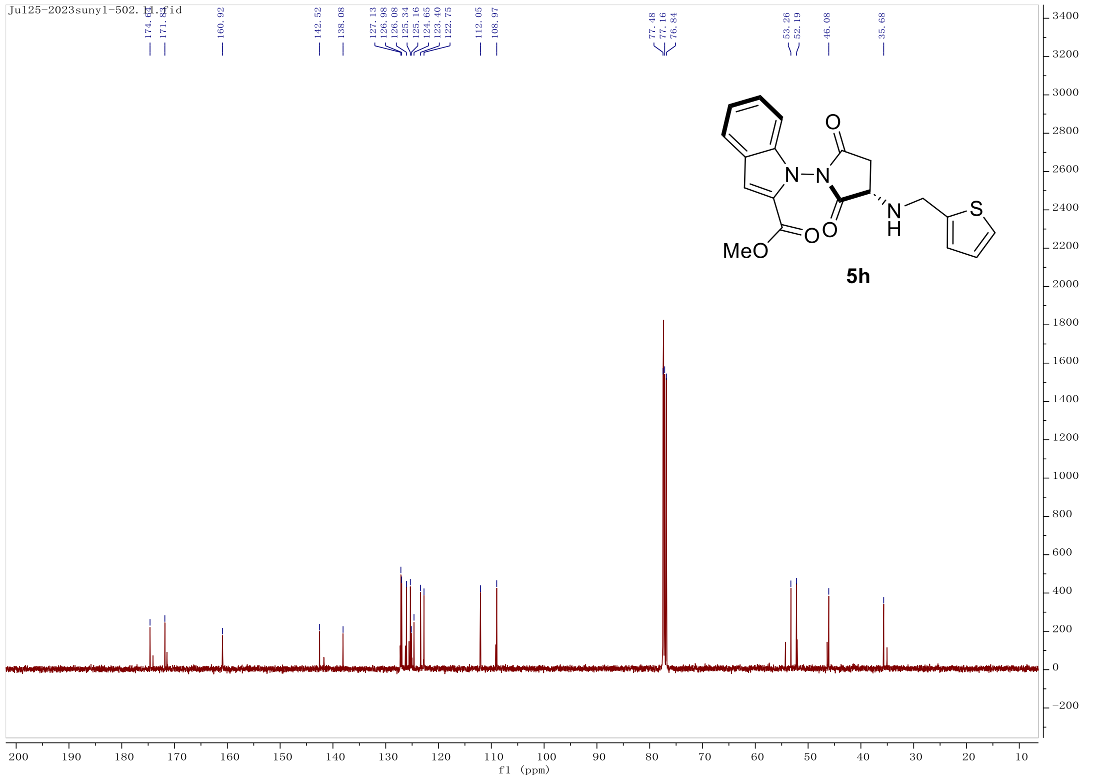
MeO
5h
f1 (ppm)

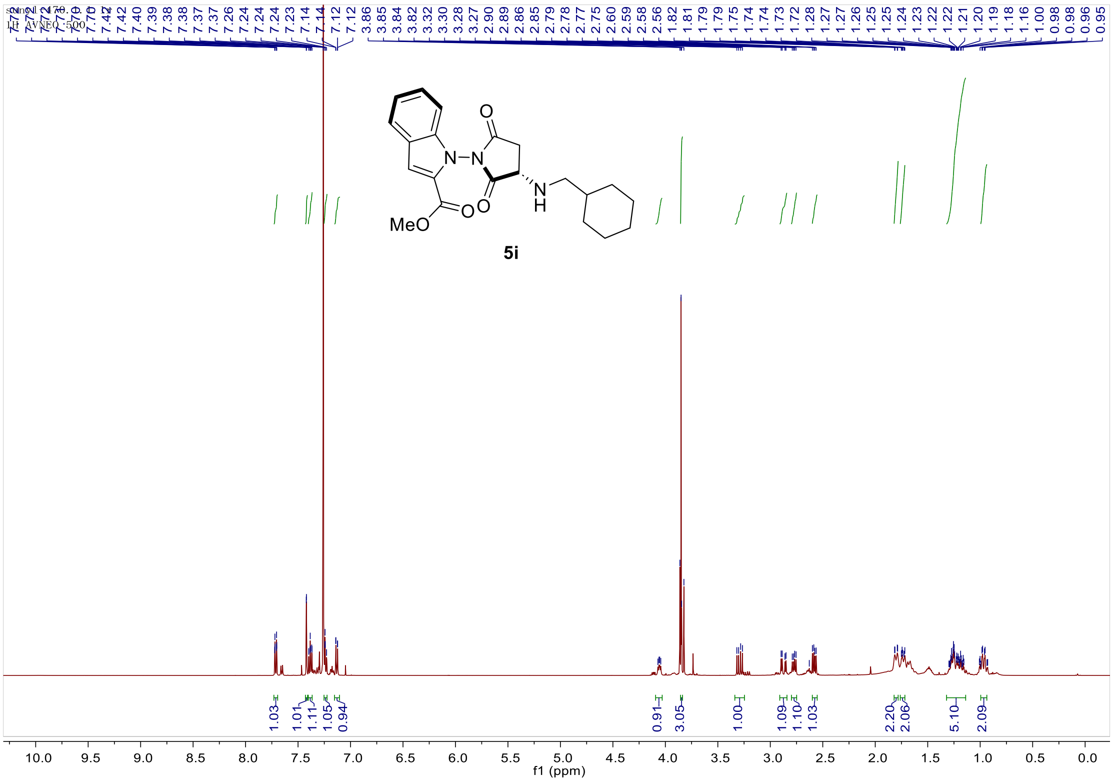

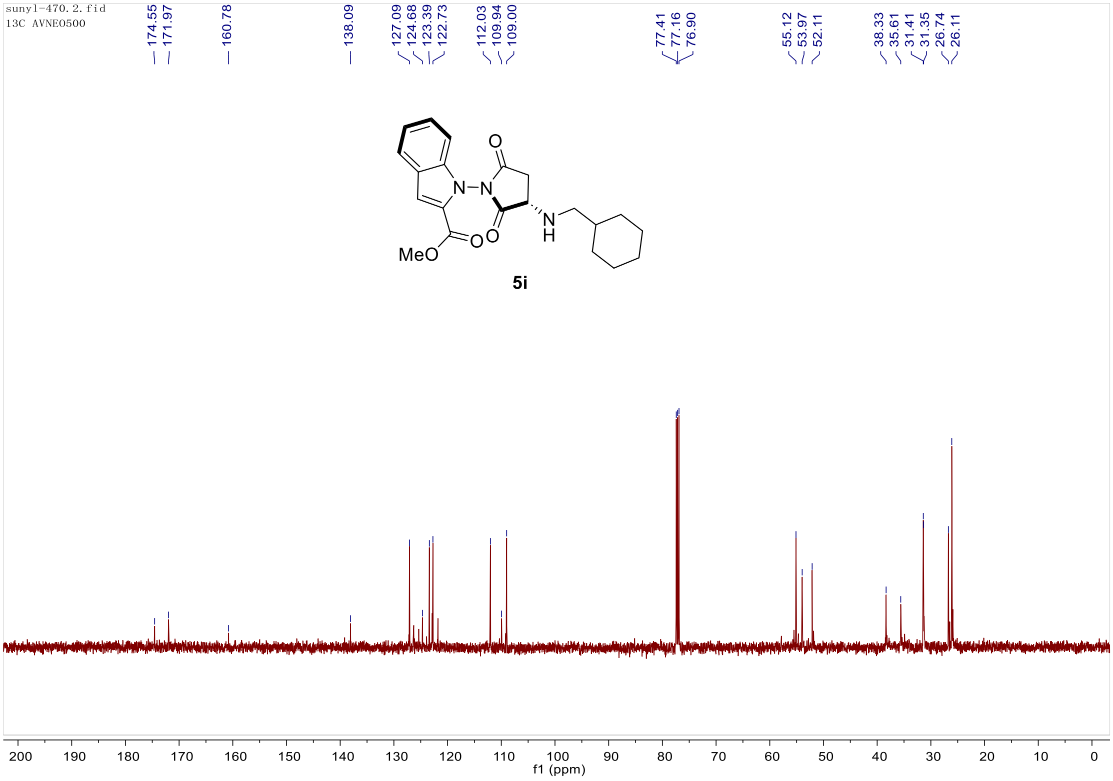

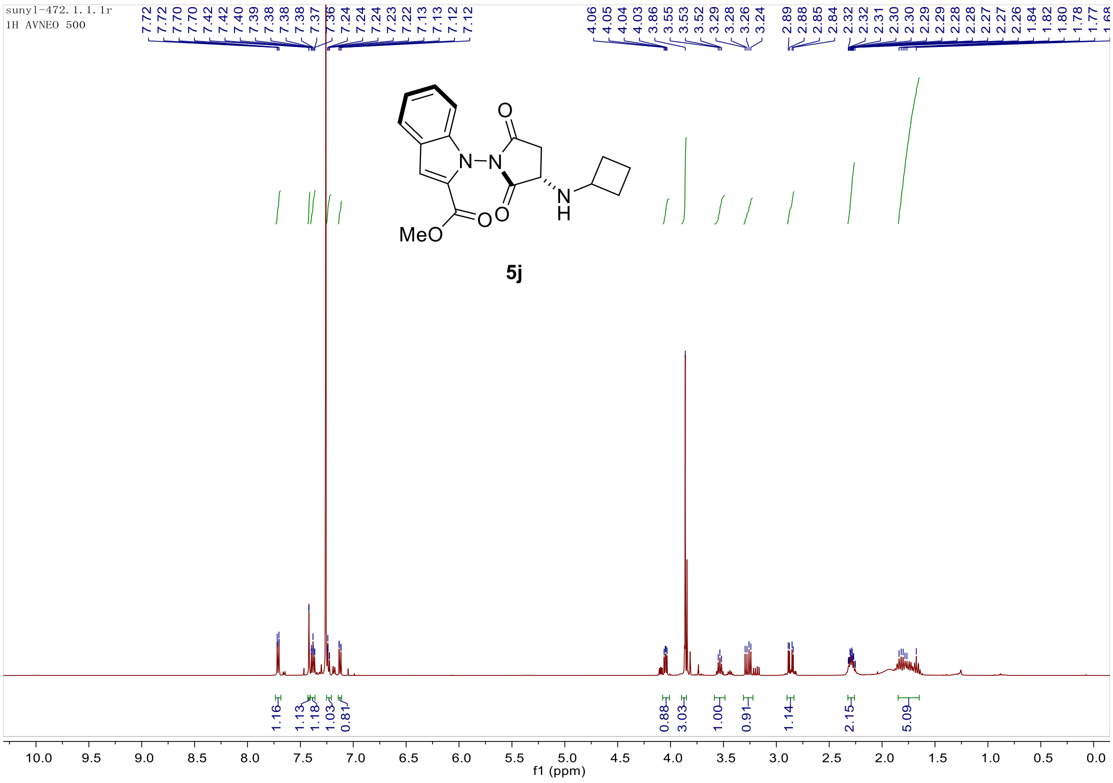

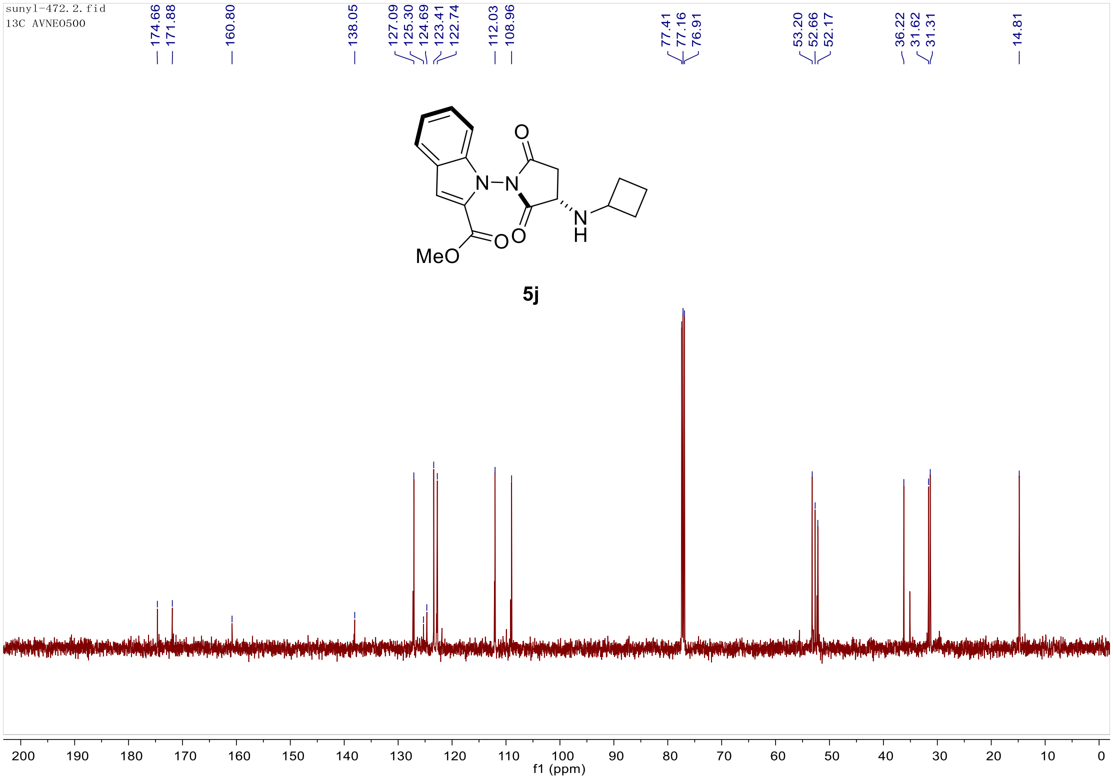

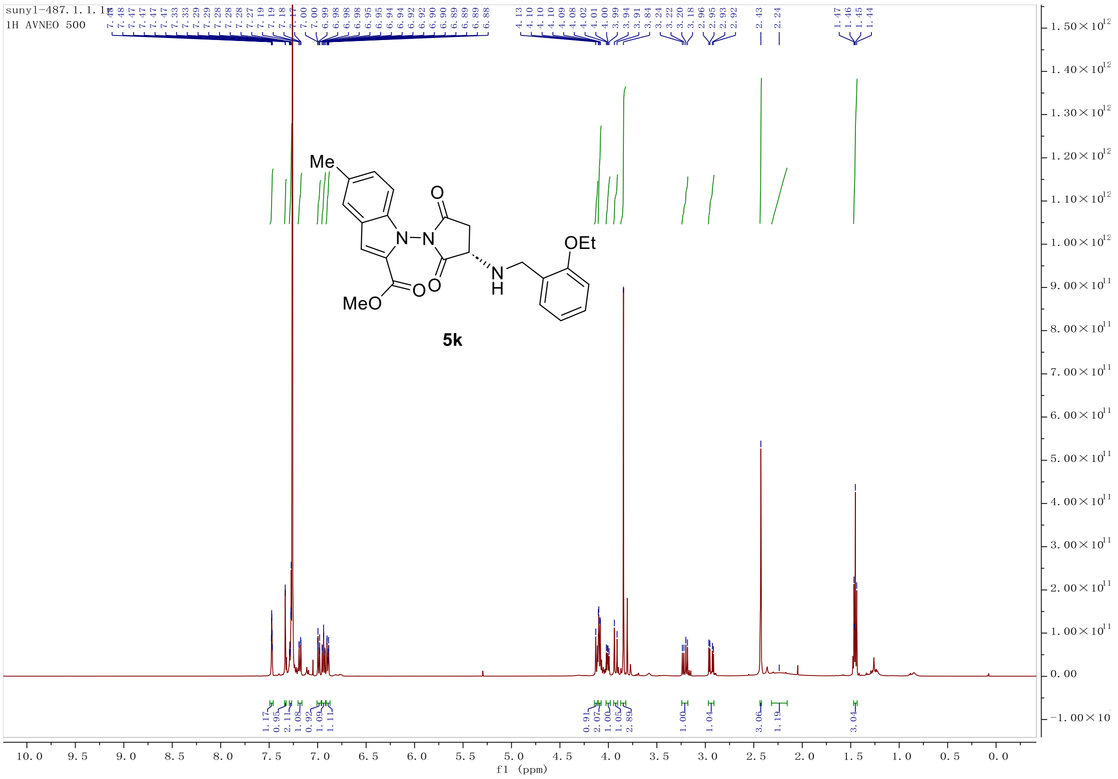

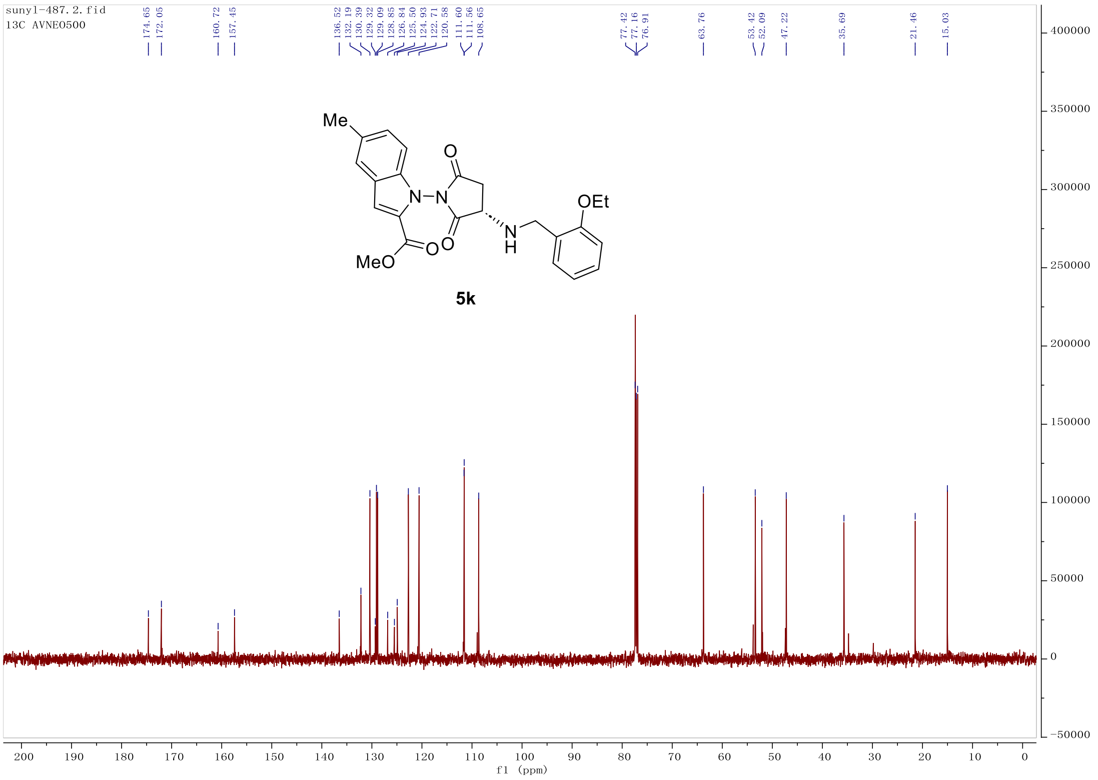

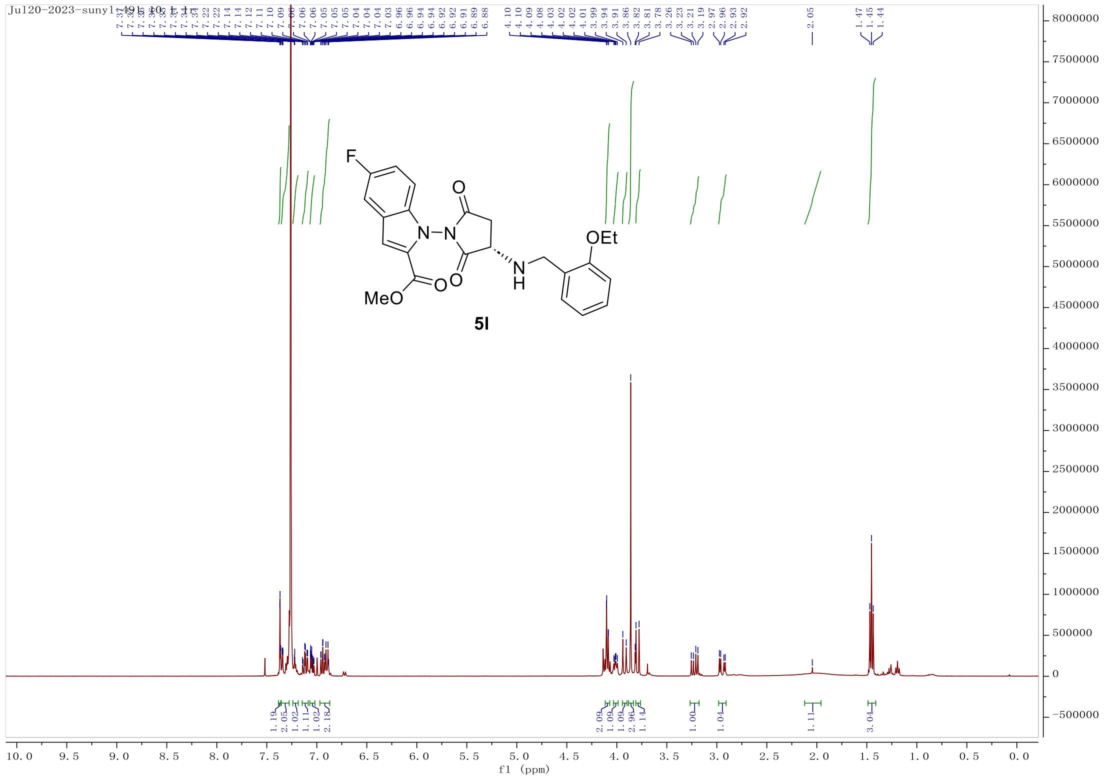
Jul20-2023-sunyl-491.10.fid
F
N
N
O
O
NH
OEt
MeO
O
5l
f1 (ppm)

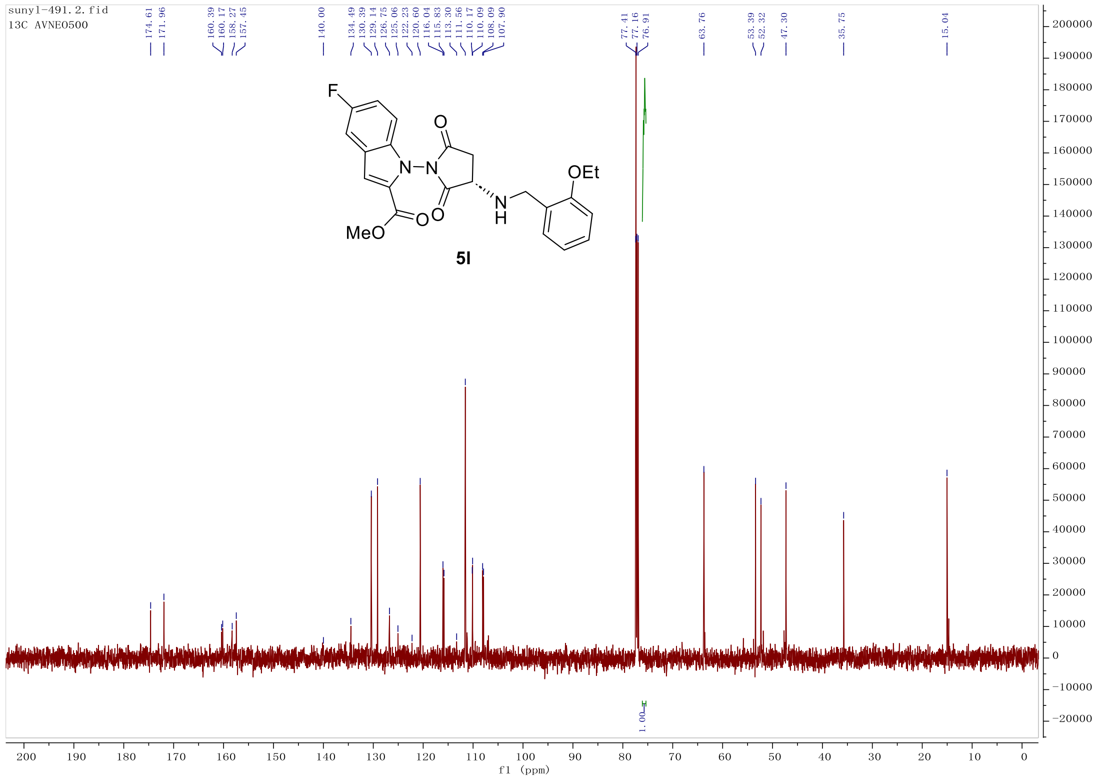
sunyl-491.2.fid
13C AVNEO500
174.61
171.96
160.39
160.17
158.27
157.45
140.00
134.49
130.39
129.14
126.75
125.06
122.23
120.60
116.04
115.83
113.30
111.56
110.17
110.09
108.09
107.90
77.41
77.16
76.91
63.76
53.39
52.32
47.30
35.75
15.04
F
N
N
O
O
NH
OEt
MeO
O
5l
f1 (ppm)

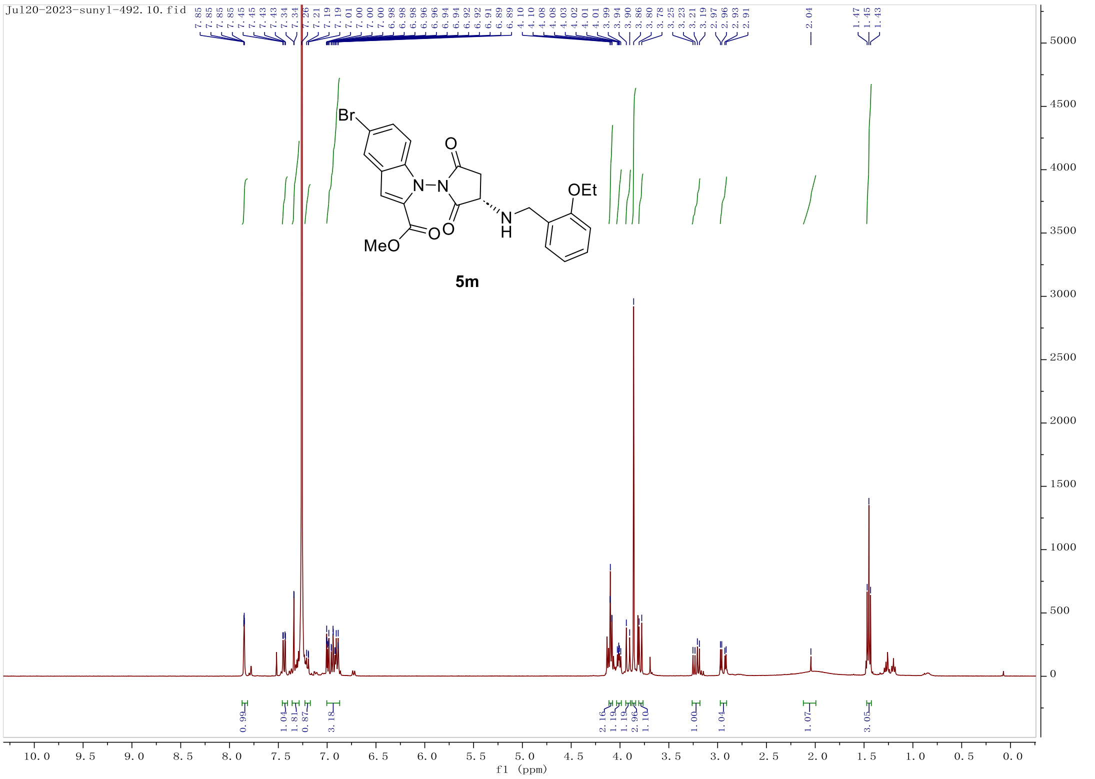
Jul20-2023-sunyl-492.10.fid
7.85 7.85 7.85 7.85 7.45 7.45 7.43 7.43 7.34 7.34 7.26 7.21 7.19 7.19 7.01 7.00 7.00 7.00 6.98 6.98 6.96 6.96 6.94 6.94 6.92 6.92 6.91 6.89 4.10 4.08 4.08 4.03 4.02 4.01 4.01 3.99 3.94 3.90 3.86 3.80 3.78 3.25 3.23 3.21 3.19 2.97 2.96 2.93 2.91 2.04 1.47 1.45 1.43
Br
O
N
N
O
OEt
N
H
MeO
O
5m
0.99 1.04 1.81 0.87 3.18 2.16 1.19 1.19 2.96 1.10 1.00 1.04 1.07 3.05
f1 (ppm)

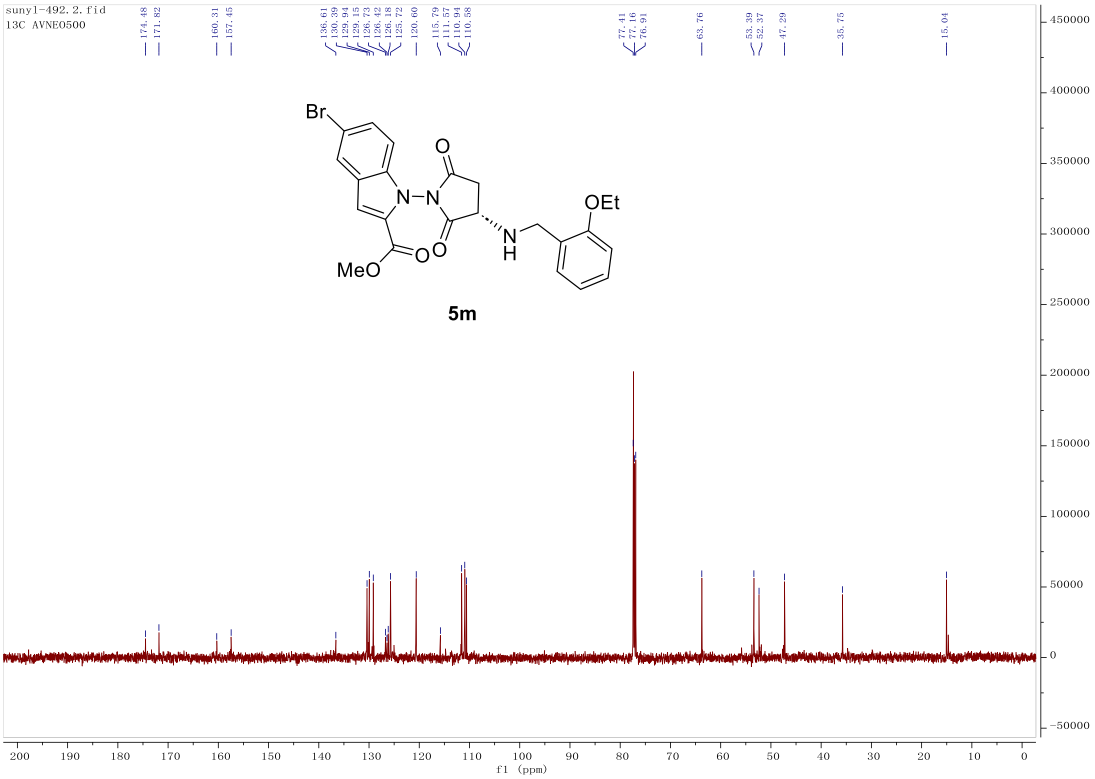
sunyl-492.2.fid
13C AVNEO500
174.48 171.82 160.31 157.45 136.61 130.39 129.94 129.15 126.73 126.42 126.18 125.72 120.60 115.79 111.57 110.94 110.58 77.41 77.16 76.91 63.76 53.39 52.37 47.29 35.75 15.04
Br
O
N
N
O
OEt
N
H
MeO
O
5m
f1 (ppm)

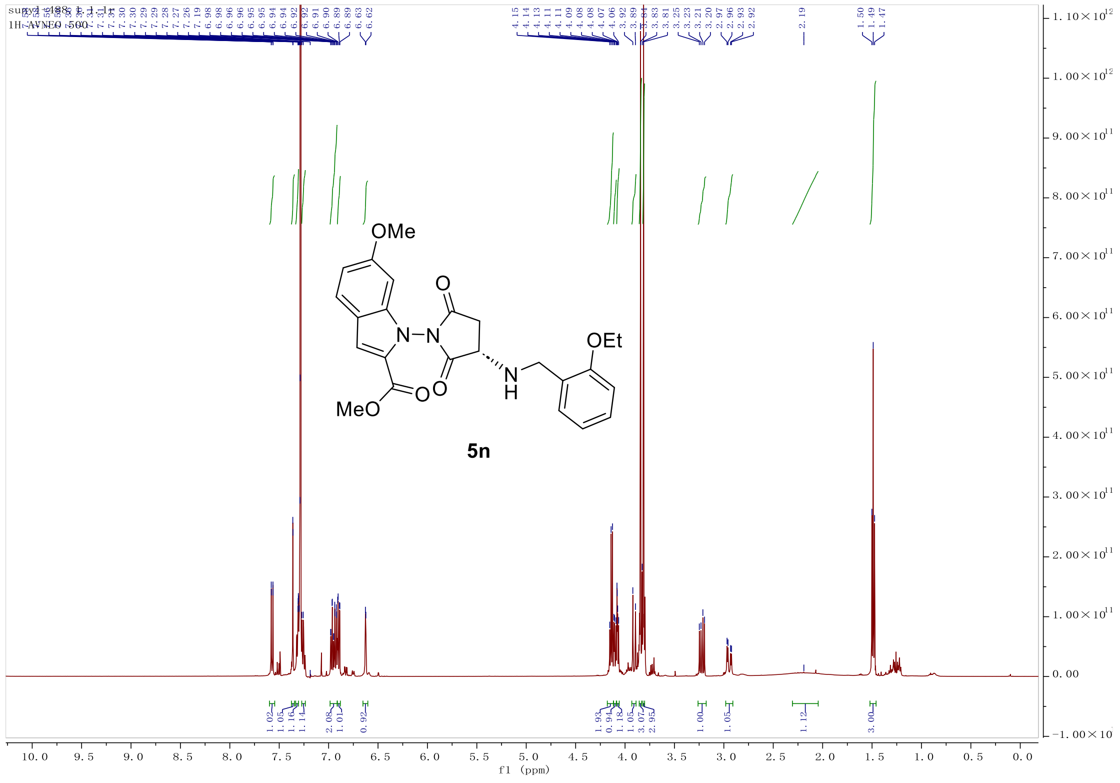

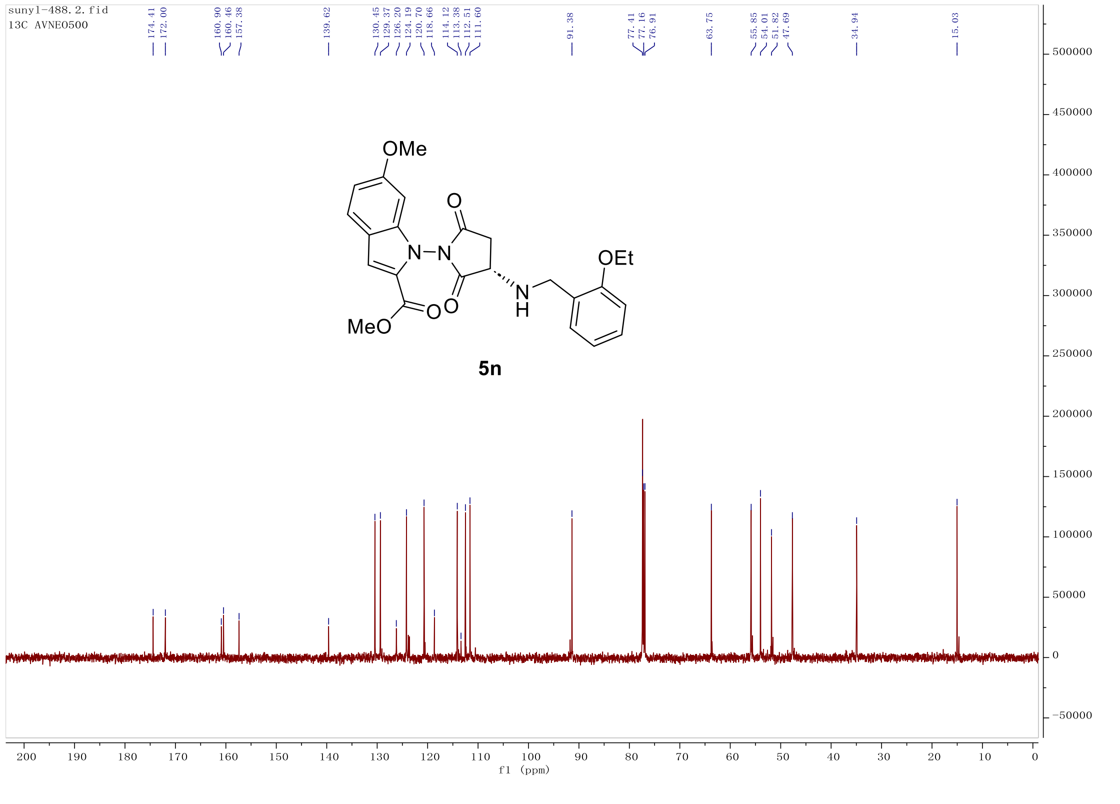

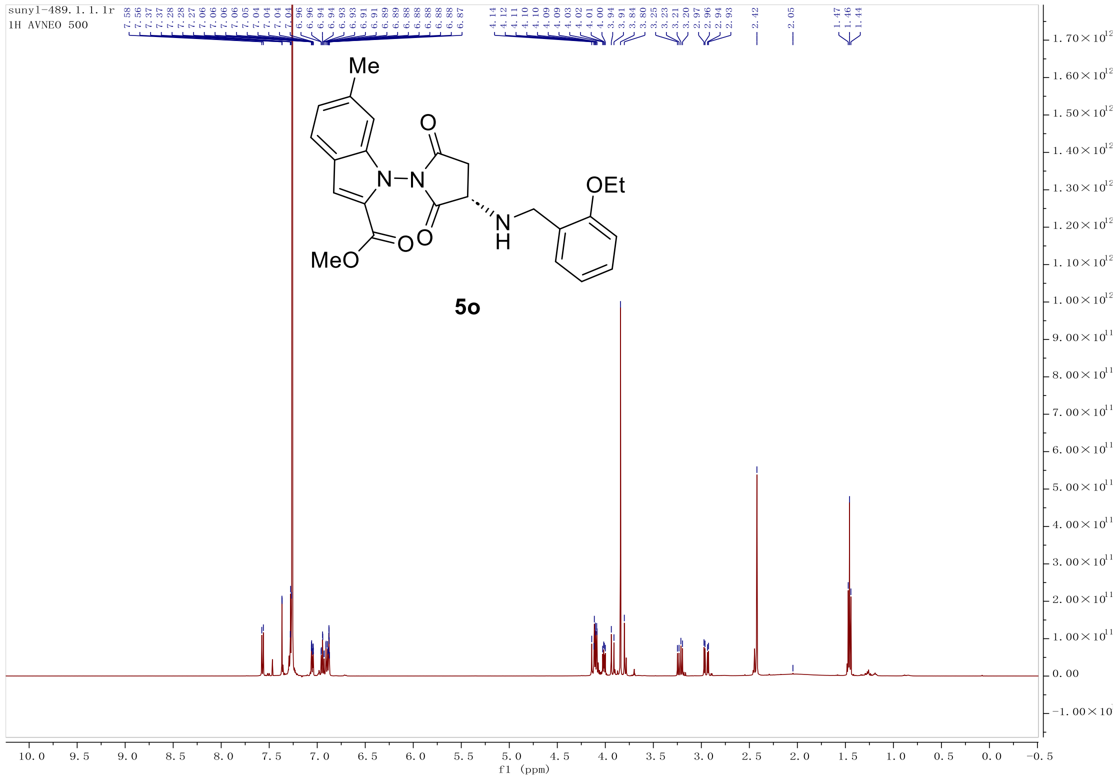

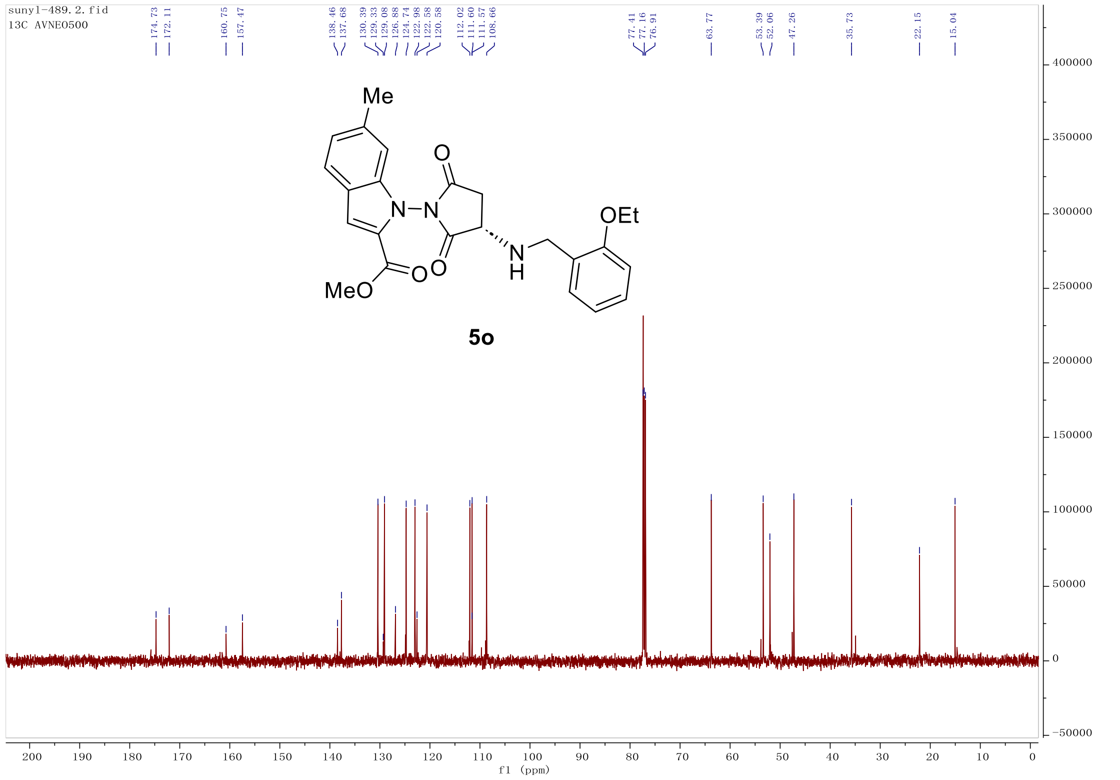

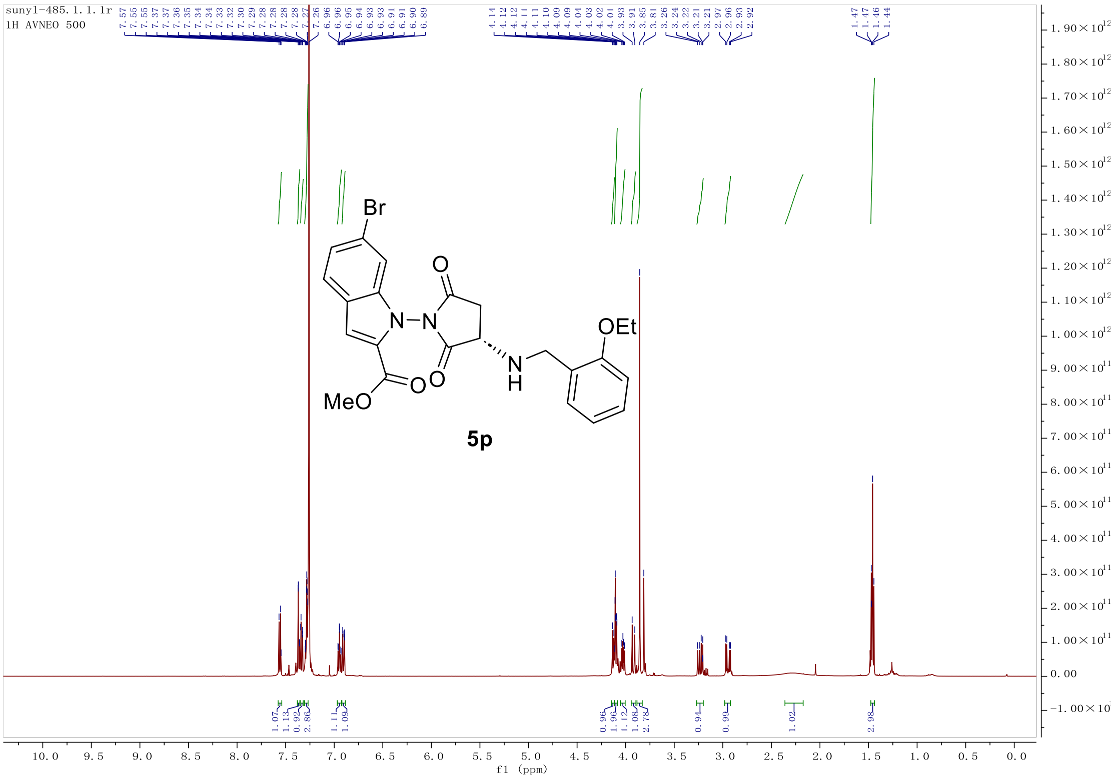

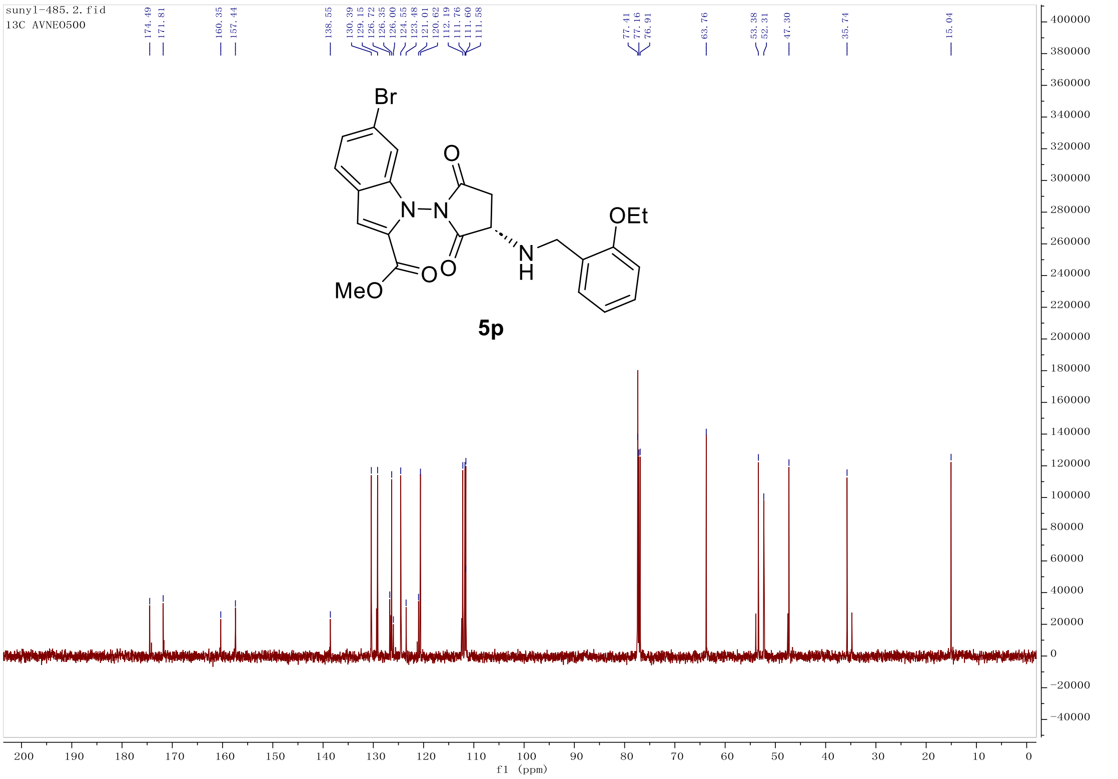

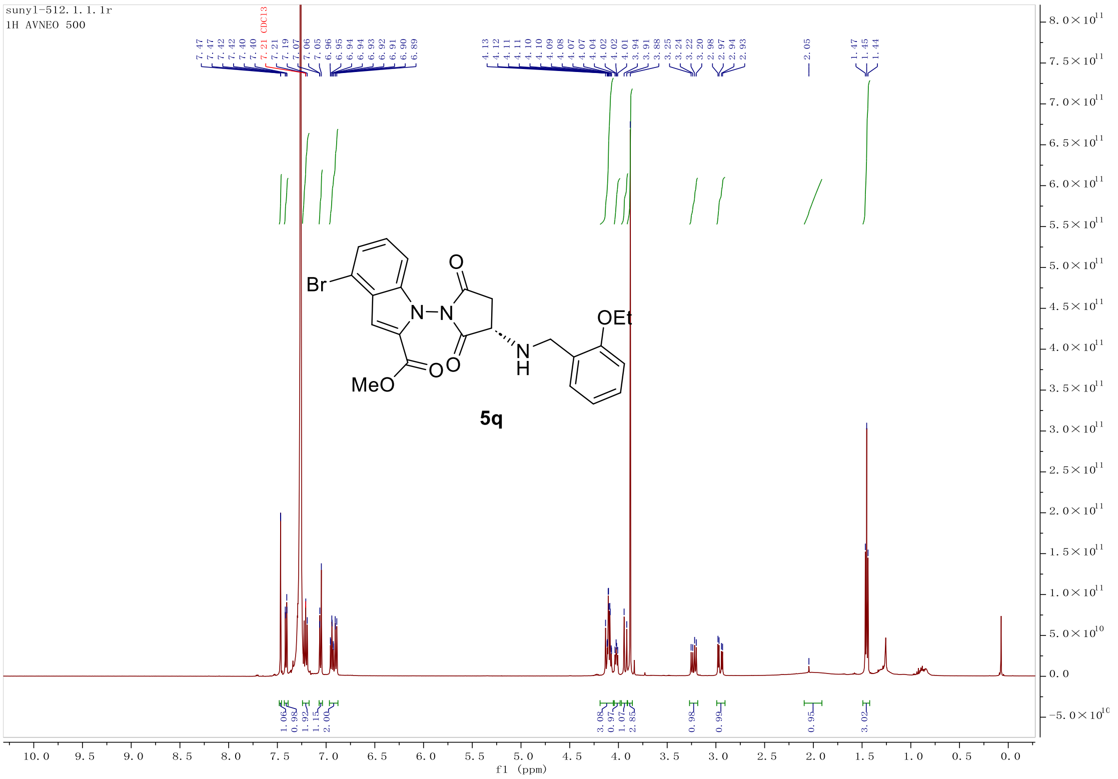
sunyl-512.1.1.1r
1H AVNEO 500
Br
N
N
O
O
OEt
N
H
MeO
O
5q
f1 (ppm)

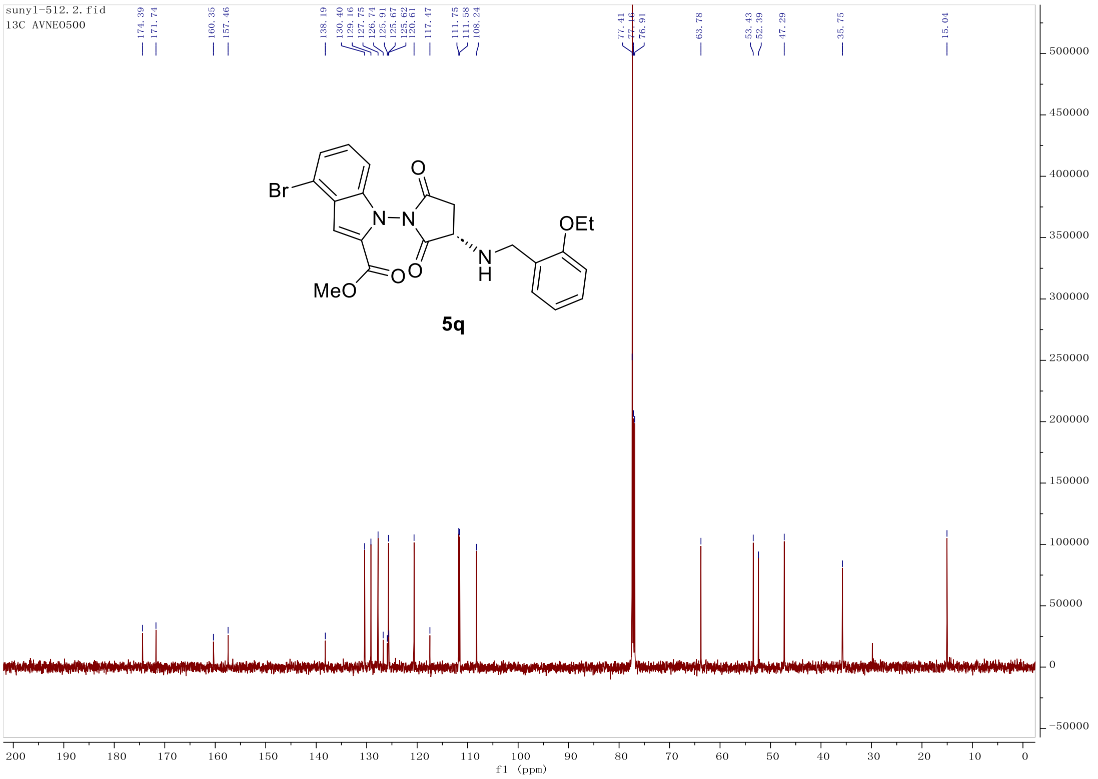
sunyl-512.2.fid
13C AVNEO500
Br
N
N
O
O
OEt
N
H
MeO
O
5q
f1 (ppm)

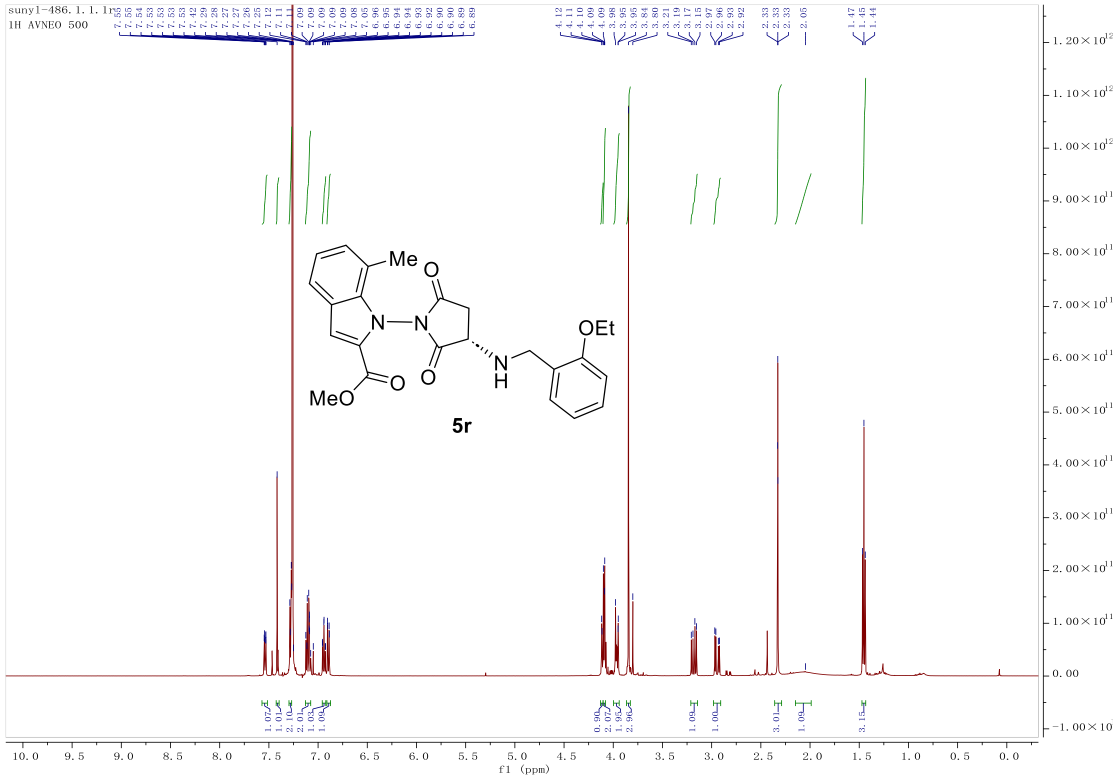

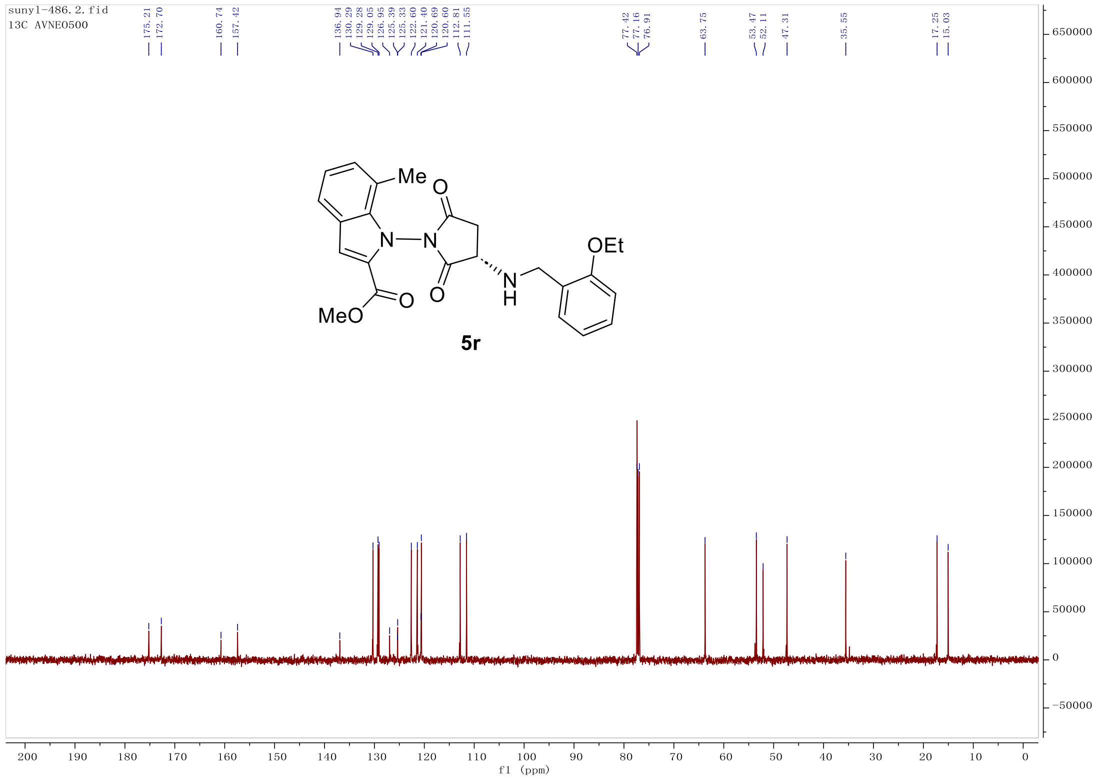

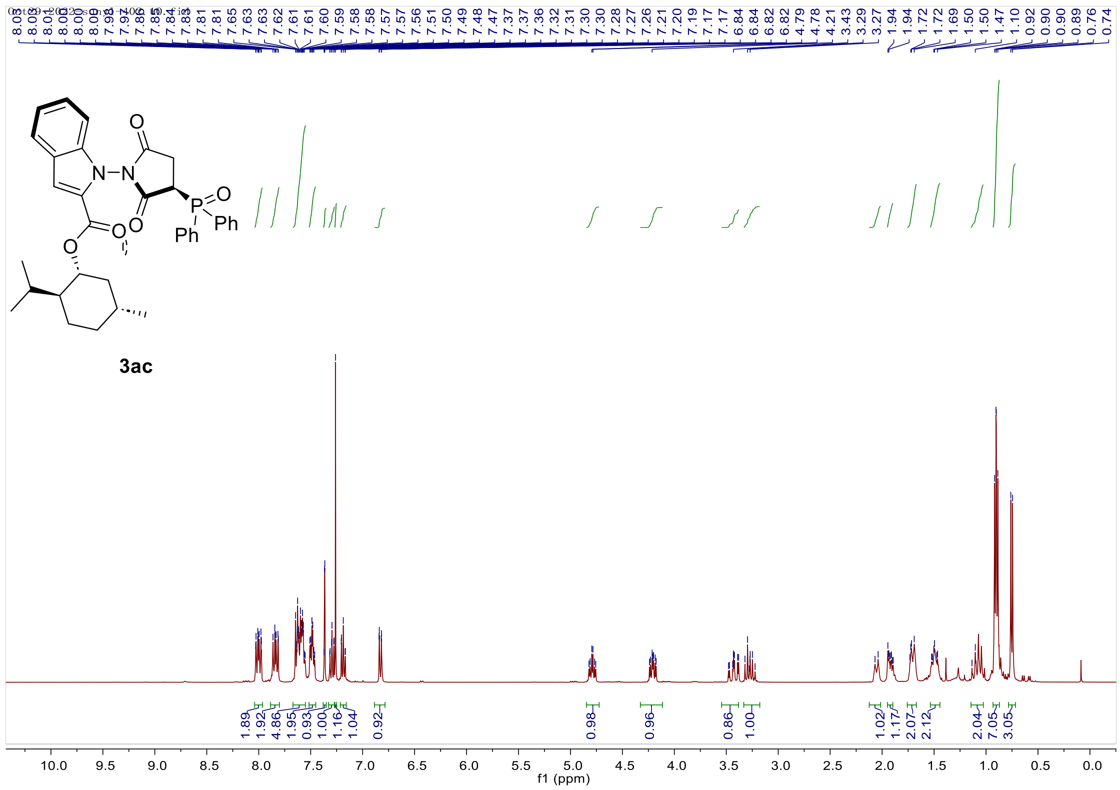

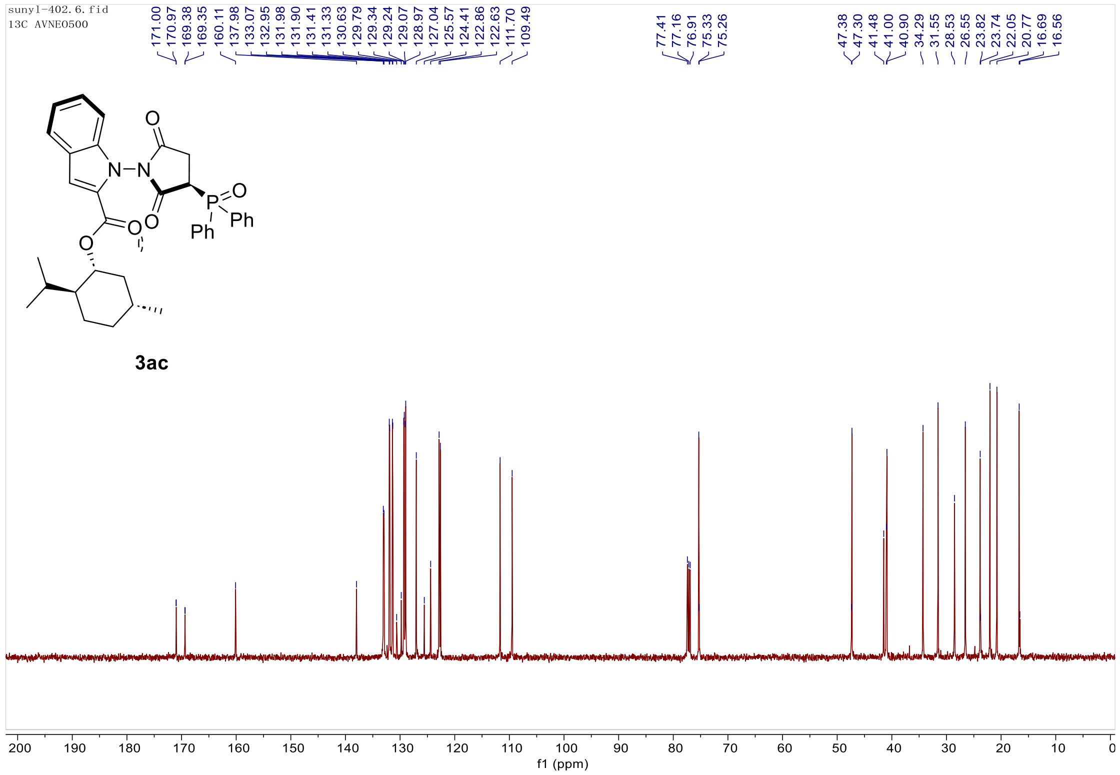

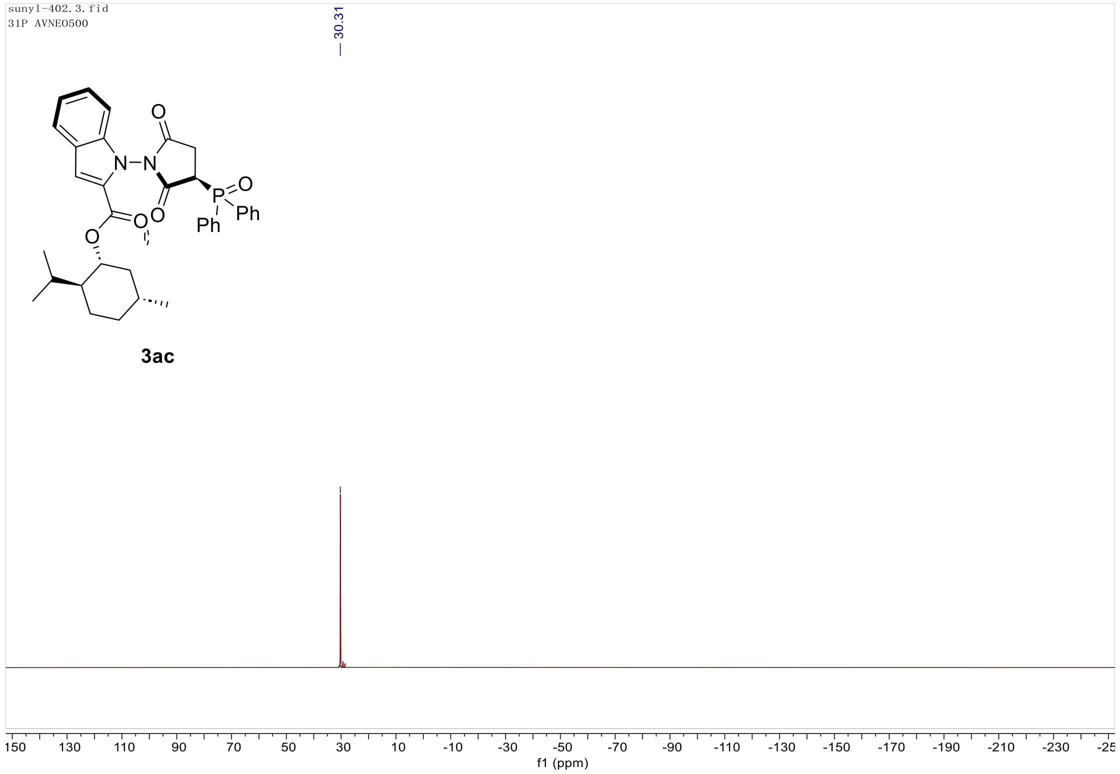
sunyl-402.3.fid
31P AVNEO500
30.31
3ac
f1 (ppm)

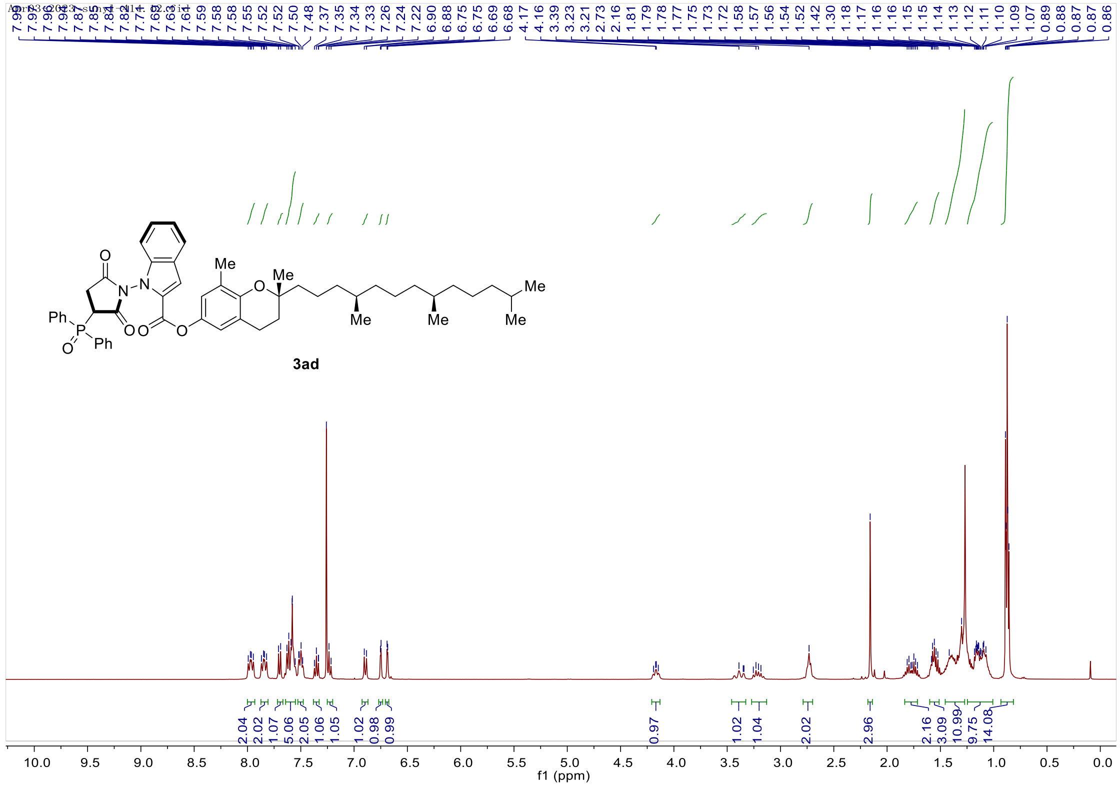
7.99 7.97 7.96 7.94 7.87 7.85 7.84 7.82 7.74 7.72 7.63 7.61 7.59 7.58 7.58 7.55 7.52 7.52 7.50 7.48 7.37 7.35 7.34 7.33 7.26 7.24 7.22 6.90 6.88 6.75 6.75 6.69 6.68
4.17 4.16 3.39 3.23 3.21 2.73 2.16 1.81 1.79 1.78 1.77 1.75 1.73 1.72 1.58 1.57 1.56 1.54 1.52 1.42 1.30 1.18 1.17 1.16 1.16 1.15 1.15 1.14 1.13 1.12 1.11 1.10 1.09 1.07 0.89 0.88 0.87 0.86
3ad
2.04 2.02 1.07 5.06 2.05 1.06 1.05 1.02 0.98 0.99 0.97 1.02 1.04 2.02 2.96 2.16 3.09 10.99 9.75 14.08
f1 (ppm)

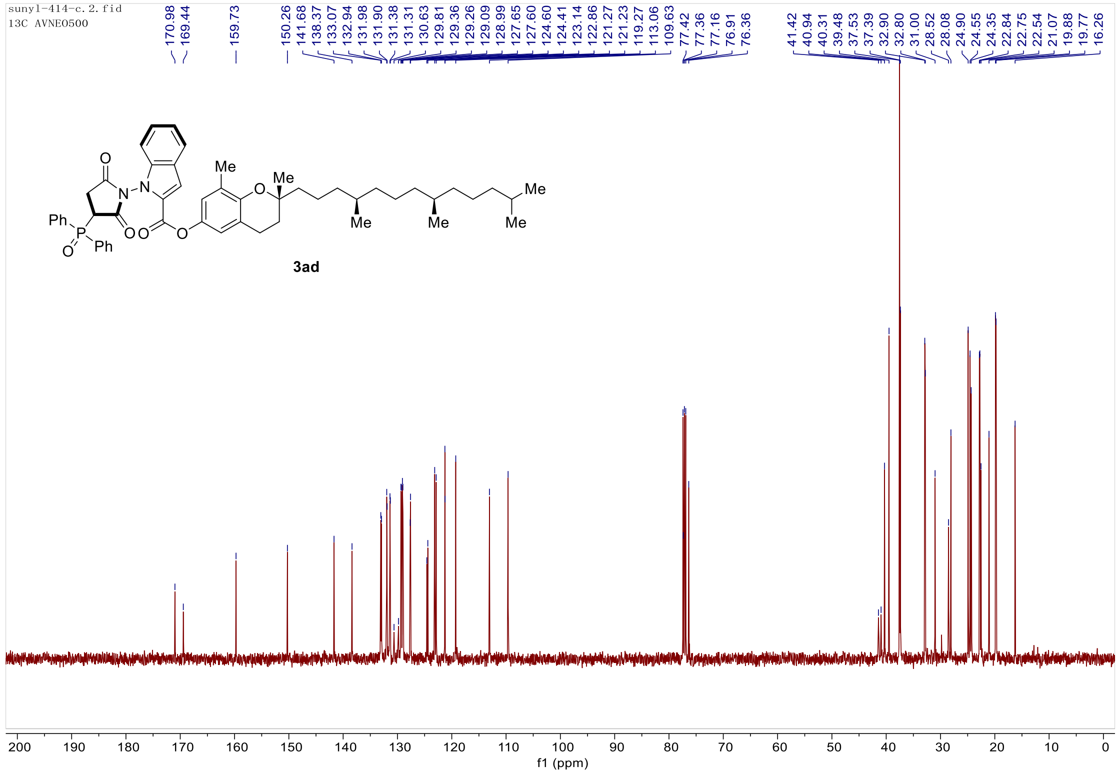
sunyl-414-c.2.fid
13C AVNEO500
170.98
169.44
159.73
150.26
141.68
138.37
133.07
132.94
131.98
131.90
131.38
131.31
130.63
129.81
129.36
129.26
129.09
128.99
127.65
127.60
124.60
124.41
123.14
122.86
121.27
121.23
119.27
113.06
109.63
77.42
77.36
77.16
76.91
76.36
41.42
40.94
40.31
39.48
37.53
37.39
32.90
32.80
31.00
28.52
28.08
24.90
24.55
24.35
22.84
22.75
22.54
21.07
19.88
19.77
16.26
Me
Me
Me
Me
Me
Me
O
O
O
O
O
N
N
Ph
P
Ph
O
3ad
f1 (ppm)

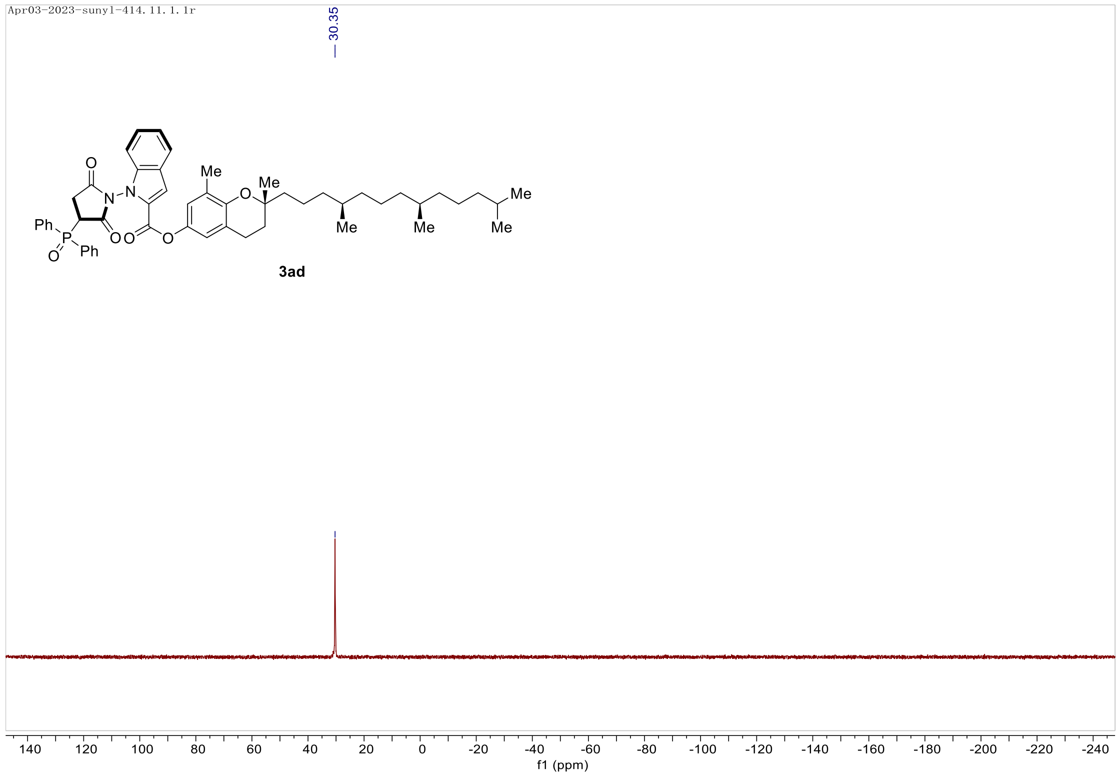
Apr03-2023-sunyl-414.11.1.1r
30.35
3ad
f1 (ppm)

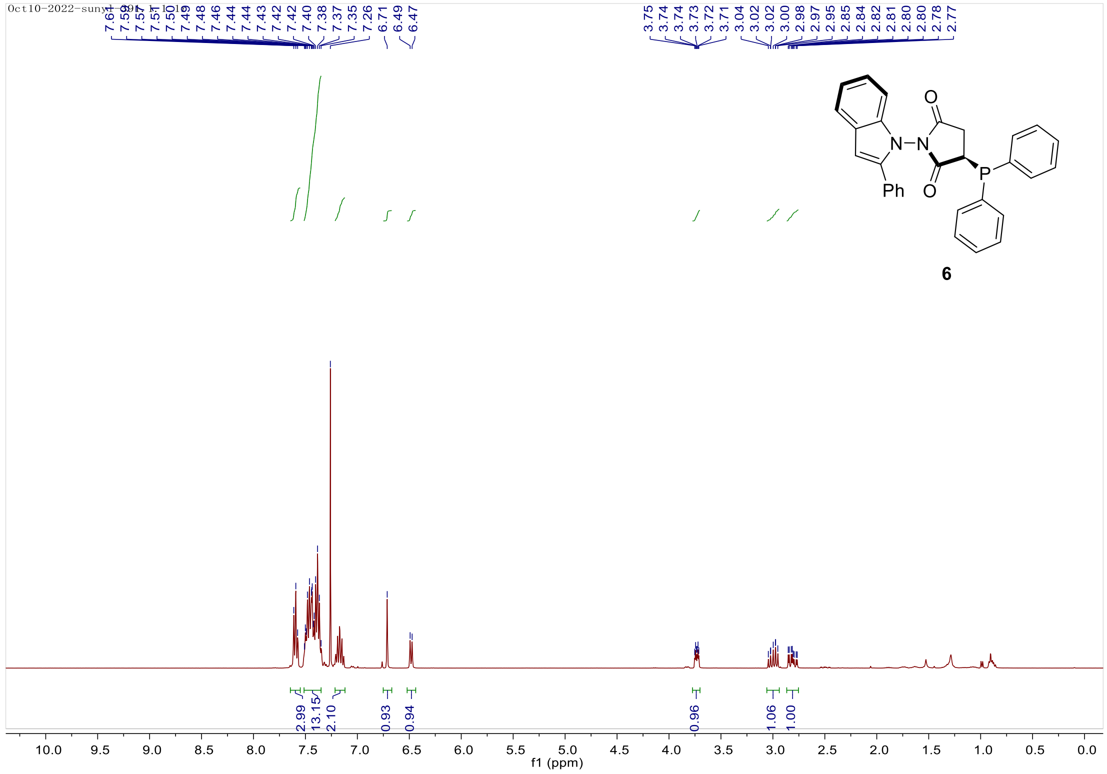
Oct10-2022-suny1-391
7.61
7.59
7.57
7.51
7.50
7.49
7.48
7.46
7.44
7.44
7.43
7.42
7.42
7.40
7.38
7.37
7.35
7.26
6.71
6.49
6.47
3.75
3.74
3.74
3.73
3.72
3.71
3.04
3.02
3.02
3.00
2.98
2.97
2.95
2.85
2.84
2.82
2.81
2.80
2.78
2.77
Ph
O
O
N
N
P
6
2.99
13.15
2.10
0.93
0.94
0.96
1.06
1.00
f1 (ppm)

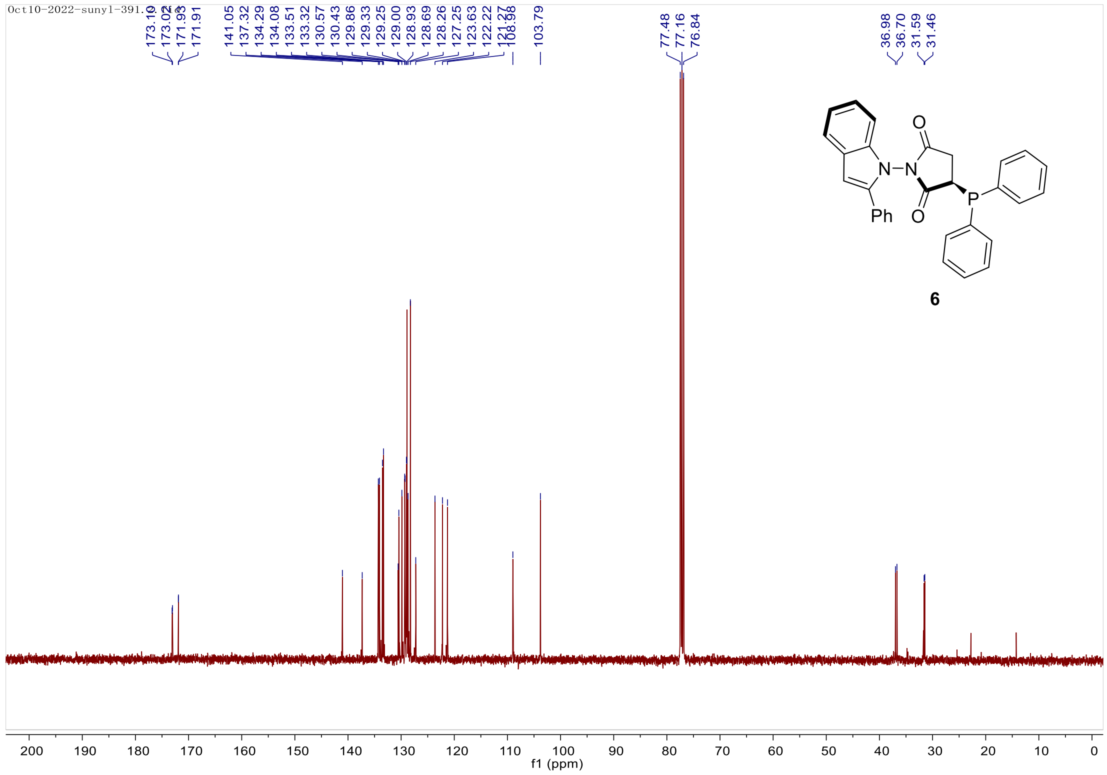
Oct10-2022-suny1-391
173.10
173.02
171.93
171.91
141.05
137.32
134.29
134.08
133.51
133.32
130.57
130.43
129.86
129.33
129.25
129.00
128.93
128.69
128.26
127.25
123.63
122.22
121.27
108.98
103.79
77.48
77.16
76.84
36.98
36.70
31.59
31.46
Ph
O
O
N
N
P
6
f1 (ppm)

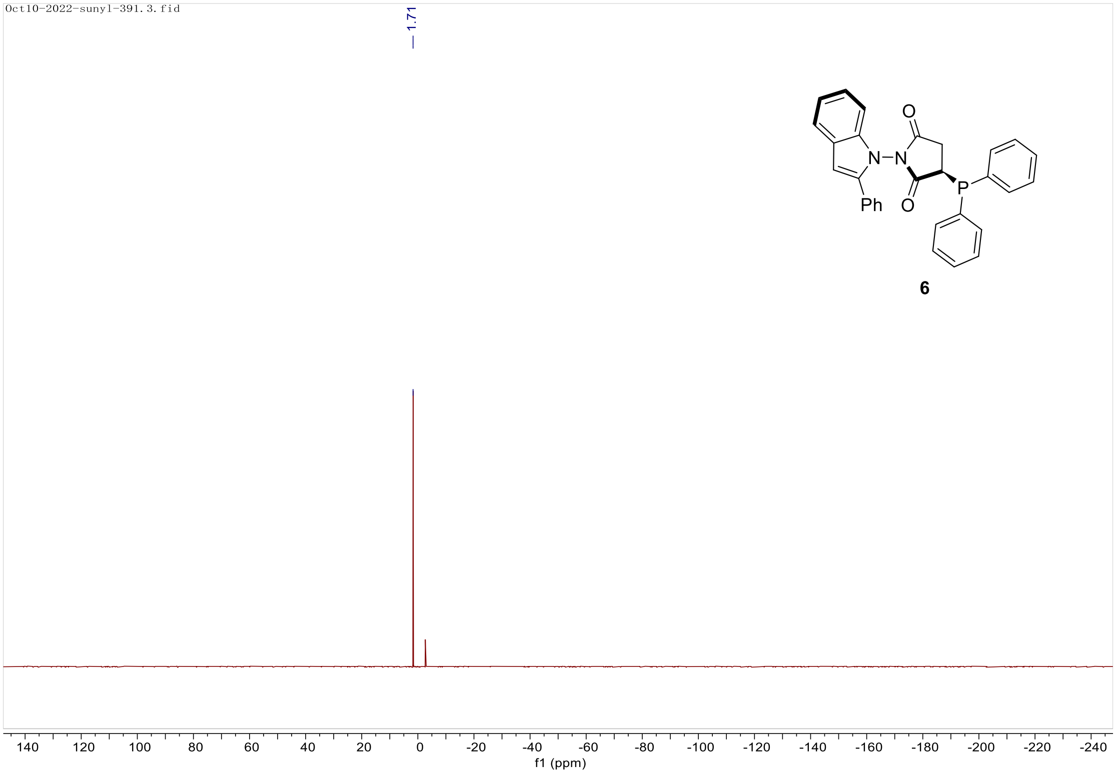
Oct10-2022-sunyl-391.3.fid
1.71
Ph
6
f1 (ppm)

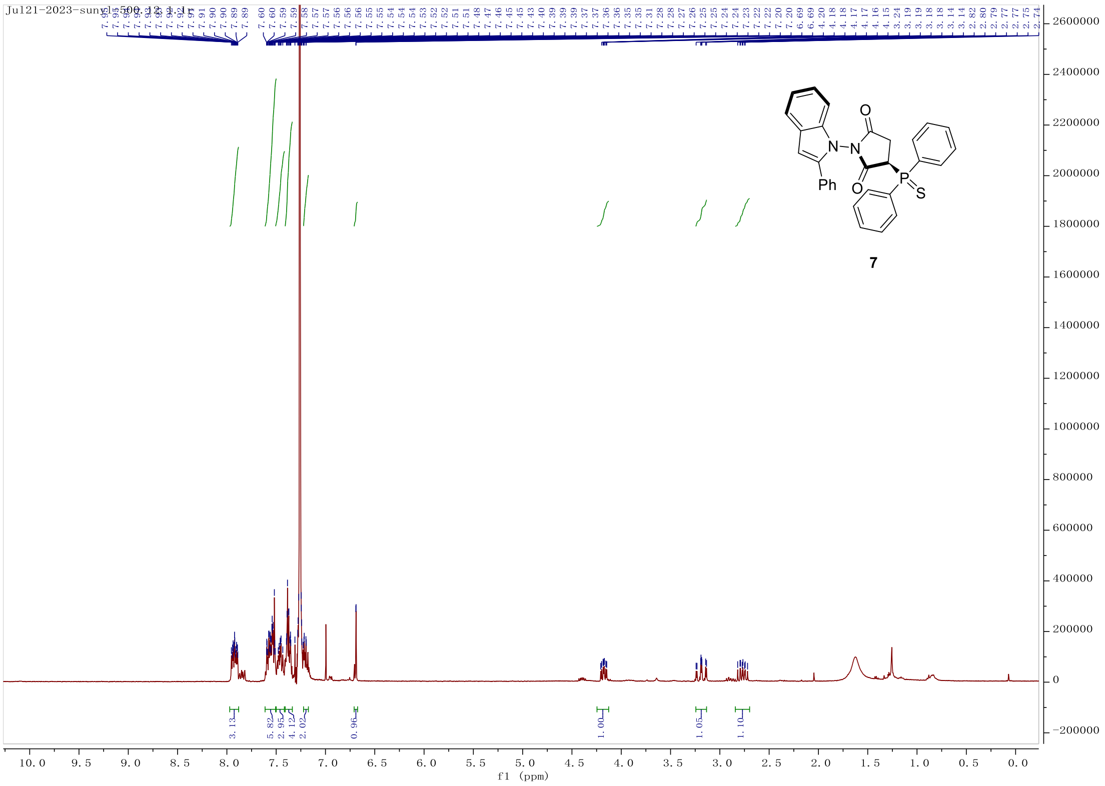
Ph
S
7
3.13
5.82
2.95
4.12
2.02
0.96
1.00
1.05
1.10
f1 (ppm)

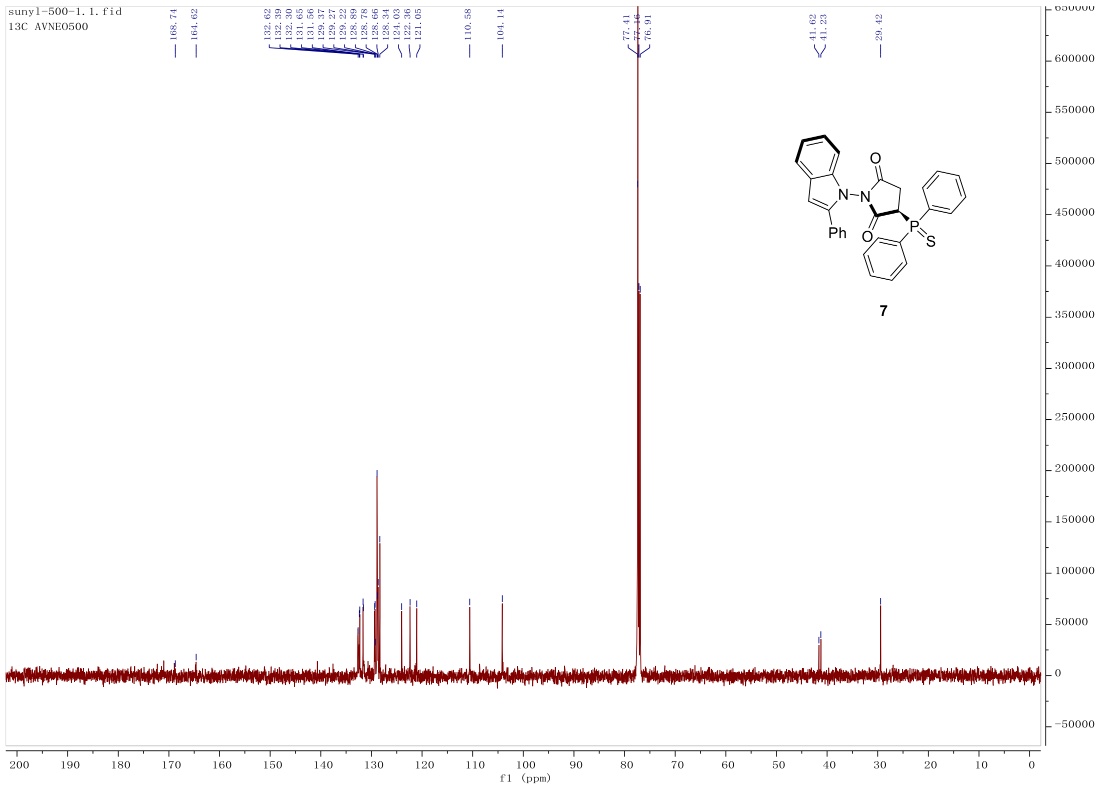

sunyl-500-1.1.fid
13C AVNEO500
168.74
164.62
132.62
132.39
132.30
131.65
131.56
129.37
129.27
129.22
128.89
128.78
128.66
128.34
124.03
122.56
121.05
110.58
104.14
77.41
77.16
76.91
41.62
41.23
29.42
N
N
O
O
P
S
Ph
7
f1 (ppm)

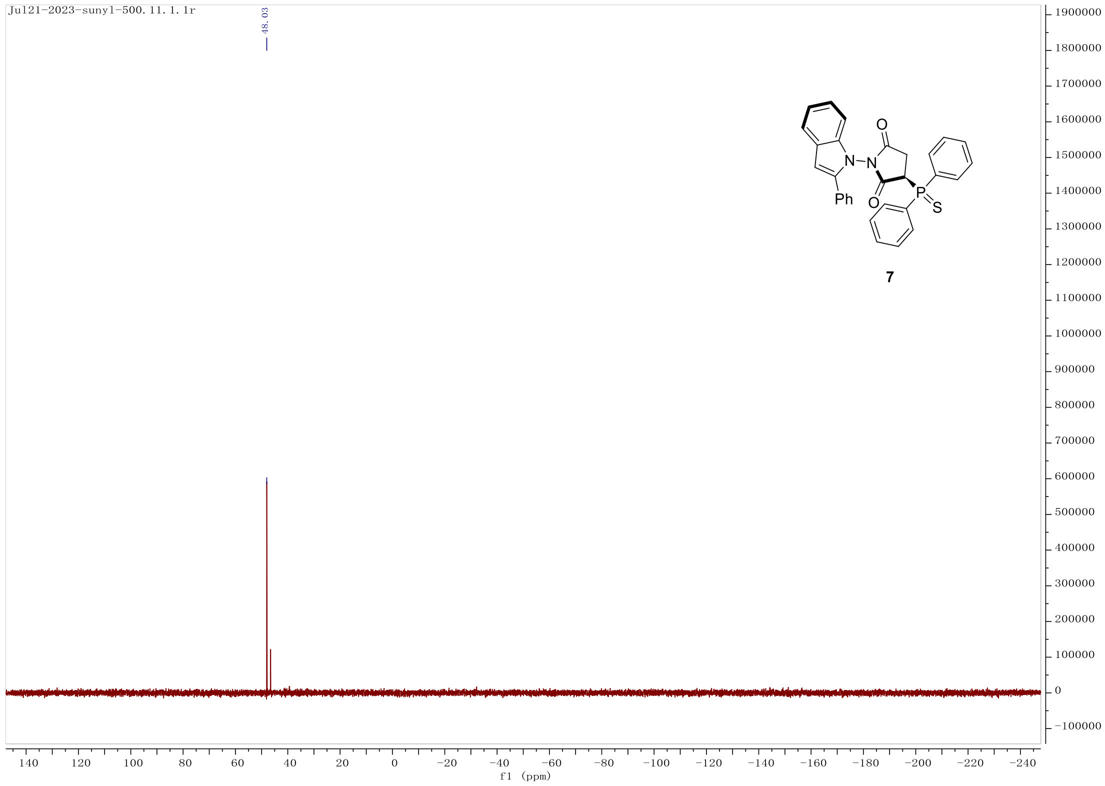

Jul21-2023-sunyl-500.11.1.1r
48.03
N
N
O
O
P
S
Ph
7
f1 (ppm)

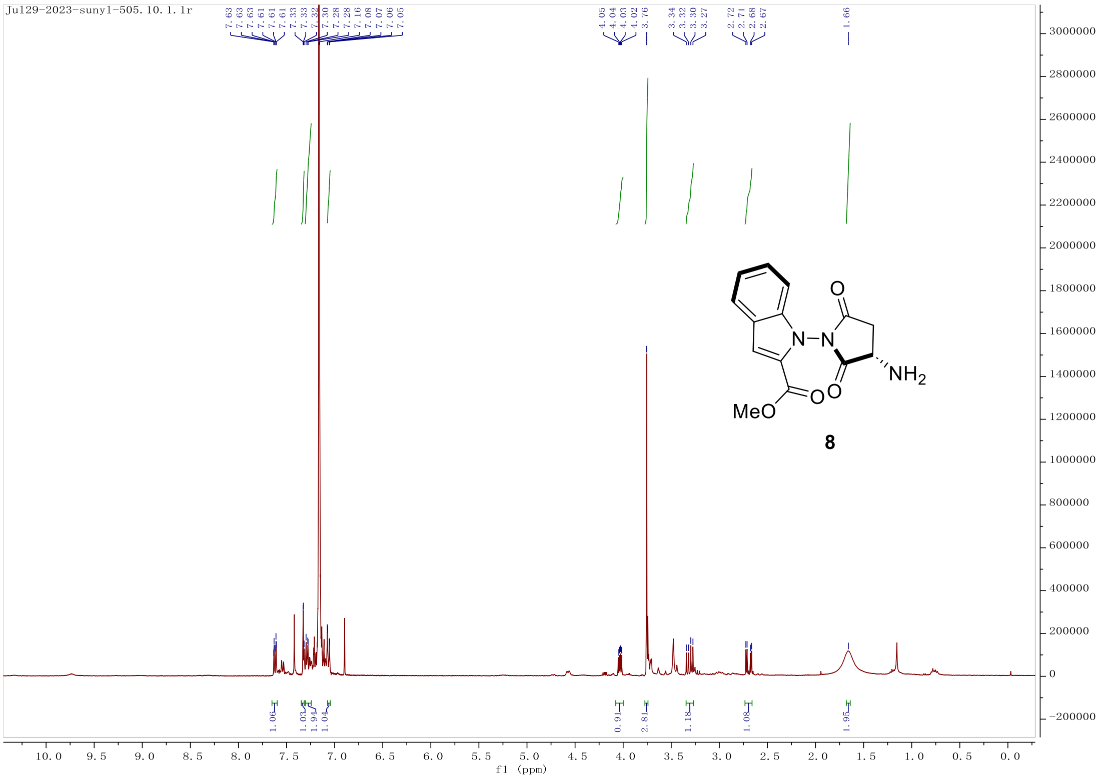

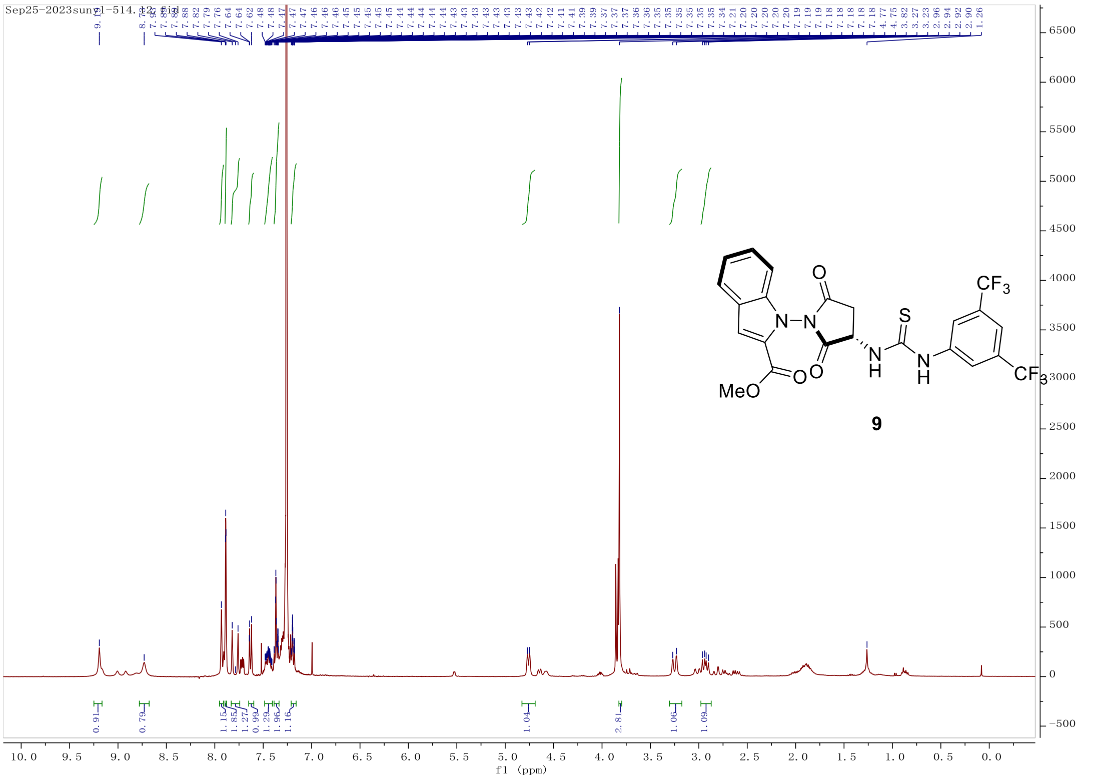

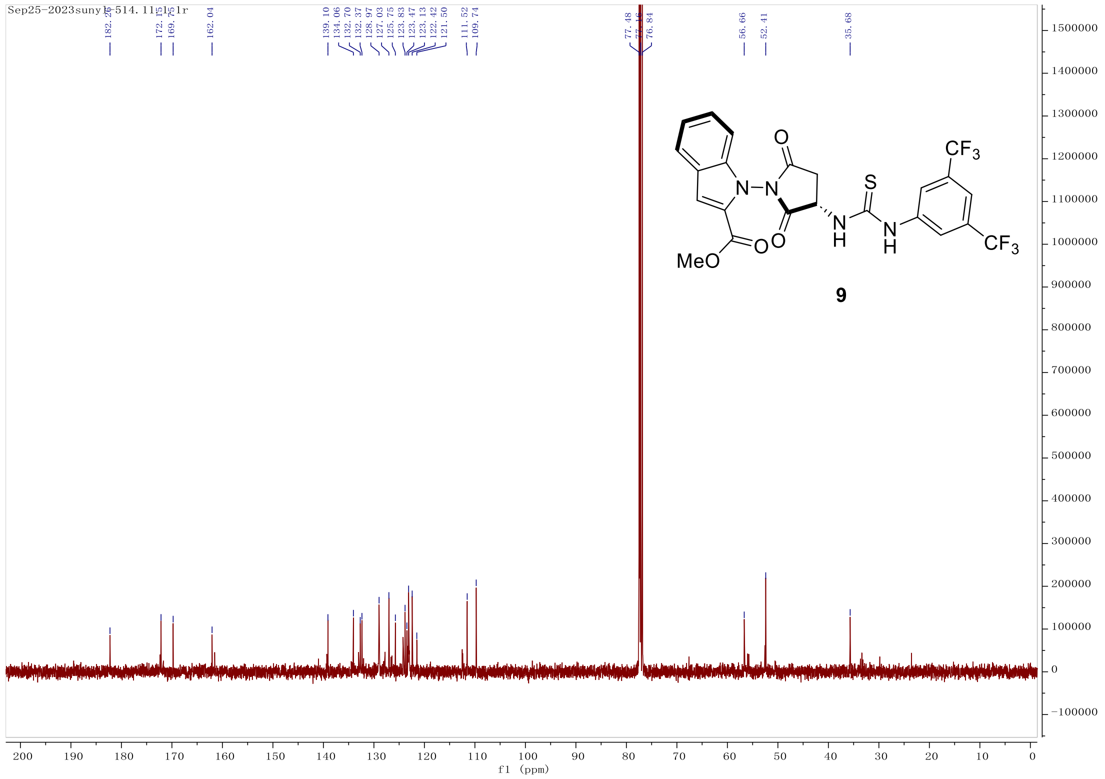
Sep25-2023sunyl-514.11.1.1r
182.23
172.15
169.75
162.04
139.10
134.06
132.70
132.37
128.97
127.03
125.75
123.83
123.47
123.13
122.42
121.50
111.52
109.74
77.48
77.16
76.84
56.66
52.41
35.68
CF3
S
O
N
N
H
N
H
CF3
MeO
O
O
9
f1 (ppm)

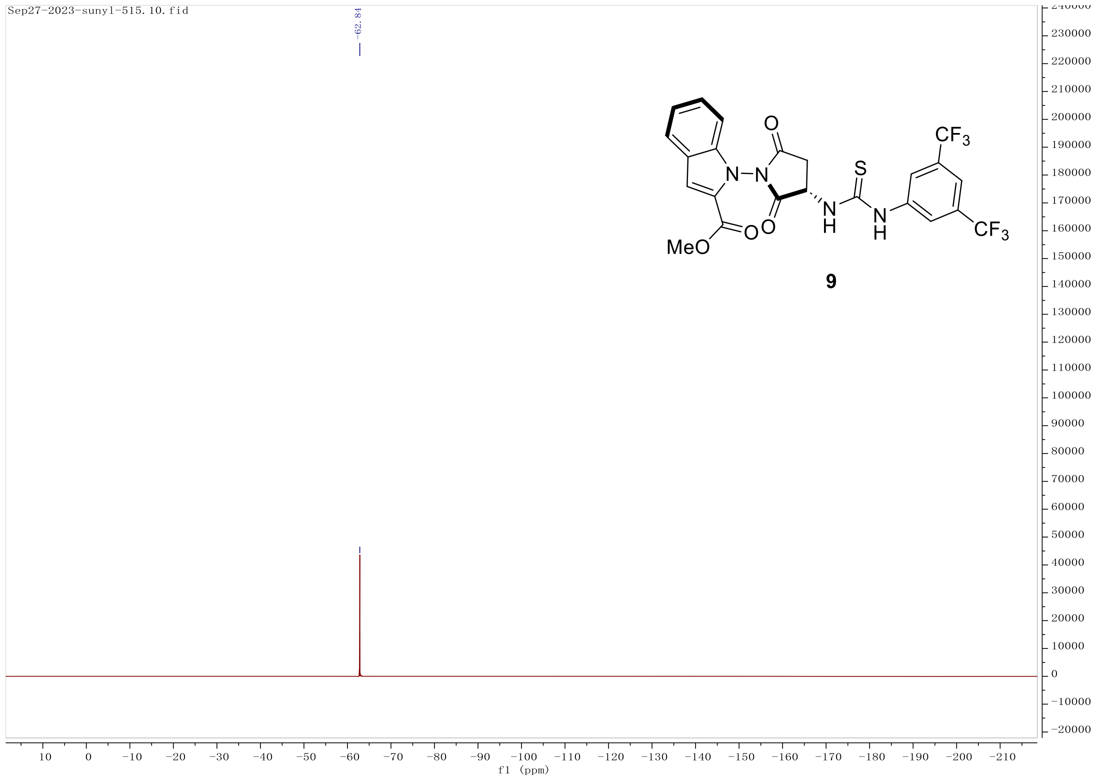
Sep27-2023-sunyl-515.10.fid
-62.84
CF3
S
O
N
N
H
N
H
CF3
MeO
O
O
9
f1 (ppm)
